# Asymmetric copper-catalyzed hydrophosphinylation of ethynylazaarenes to access *P*-chiral 2-azaaryl-ethylphosphine oxides†

**DOI:** 10.1039/d5sc00358j

**Published:** 2025-03-03

**Authors:** Jialiang Zhang, Jiajia Guo, Ruhui Xu, Di Zheng, Kai Lian, Zhaoxia Zhang, Shanshan Cao, Zhiyong Jiang

**Affiliations:** a School of Chemistry and Chemical Engineering, Henan Normal University Xinxiang Henan 453007 P. R. China caoshanshan@htu.edu.cn jiangzhiyong@htu.edu.cn; b International Scientific and Technological Cooperation Base of Chiral Chemistry, Henan University Kaifeng Henan 475004 P. R. China

## Abstract

We report a cost-effective approach for the enantioselective hydrophosphinylation of ethynylazaarenes utilizing a chiral copper catalytic platform. This strategy efficiently converts racemic secondary phosphine oxides (SPOs) into *P*-chiral tertiary phosphine oxides (TPOs) bearing functionalized olefin substituents with azaarene moieties, achieving high yields and exceptional enantioselectivities. These adducts serve as crucial intermediates in the development of valuable chiral 1,5-hybrid *P*,*N*-ligands. The facile introduction of diverse additional carbon-centered chirality through the transformation of the olefin moiety effectively enhances the enantioselectivity of asymmetric metal catalysis compared to ligands exhibiting solely *P*-chirality. Mechanistic investigations reveal that the interaction between the chiral Cu(i) complex and azaarenes promotes the kinetic resolution of SPOs. The robustness of this method is further demonstrated by its ability to incorporate deuterium atoms into the olefins, highlighting its potential relevance in pharmaceutical applications.

## Abstract 2

We report a cost-effective approach for the enantioselective hydrophosphinylation of ethynylazaarenes utilizing a chiral copper catalytic platform.

## Graphical abstract

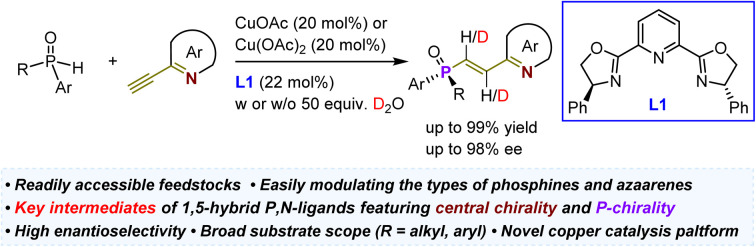

## Introduction

**Scheme 1:**
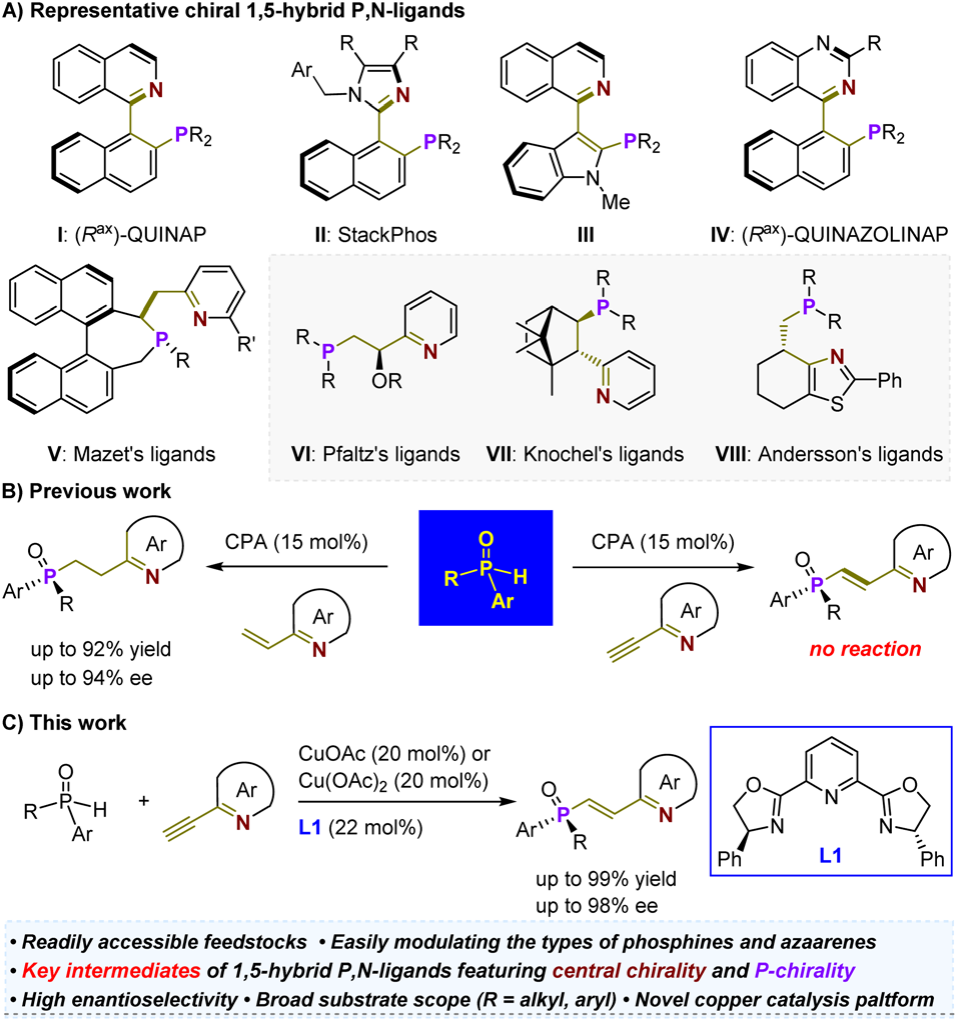
Outline of this work.

The development of readily accessible and highly efficient chiral ligands is a fundamental driving force behind the widespread application of asymmetric metal catalysis in the pharmaceutical and materials industries.^1–3^ Consequently, significant efforts have been dedicated to designing chiral *P*,*N*-ligands^4–13^ (*i.e.*, tertiary phosphines (*P*) and imine-containing azaarenes (*N*)) due to the soft phosphorus atom and hard nitrogen atom, which allow for interactions with various metals, featuring different coordination modes such as *N*-monodentate, *P*-monodentate, and bidentate in mononuclear or multinuclear complexes.^14^ Among them, a wide range of chiral 1,5-hybrid *P*,*N*-ligands has been devised (Scheme 1A).^15–22^ Most of these ligands are atropisomeric (*e.g.*, I–V), requiring labor-intensive synthesis from costly chiral feedstock chemicals.^15–17^ Only rare variants (*e.g.*, VI–VIII) with chirality located on simpler carbon chains have been developed, although they have demonstrated robust capabilities in enabling precise enantioselective reactions, particularly the significant hydrogenation of various olefins.^18–22^ This predicament may be attributed to the lack of efficient and generic catalytic methods. In this context, we recently developed a highly enantioselective hydrophosphinylation of vinylazaarenes catalyzed by a chiral phosphoric acid (CPA).^23^ This expedient approach allows for the precise synthesis of *P*-chiral azaarene-based tertiary phosphine oxides (TPOs) using readily available and air-stable secondary phosphine oxides (SPOs)^24–34^ as starting materials (Scheme 1B, left). The method's versatility in modulating both types of azaarenes and SPOs combined with direct reduction steps for TPOs paves the way for concise and modular synthetic approaches towards *P*-chiral^35^ 1,5-hybrid *P*,*N*-ligands. These entities have already shown promising potential as ligands in palladium-catalyzed asymmetric Tsuji–Trost allylation reactions. In this context, our objective is to investigate efficient techniques for facile assembly of central chirality elements on the ethylene scaffold similar to those observed in compounds V–VIII. This endeavor is crucial for further expanding the repertoire of patterns exhibited by such *P*-chiral ligands and addressing a broader range of challenging reactions *via* asymmetric metal catalysis.

Olefin has long been appreciated as a versatile functional group in organic synthesis due to its robust capacity for undergoing numerous classic chemical transformations, such as reduction, oxidation, epoxidation, dihydroxylation, and addition. These transformations have facilitated access to a diverse range of valuable derivatives from olefin-containing substrates. Hence, the asymmetric addition of racemic SPOs to 2-ethynylazaarenes to yield *P*-chiral 2-azaaryl-ethylphosphine oxides holds substantial synthetic value. However, our catalytic platform failed to initiate any reaction, indicating the insufficient activation ability of chiral Brønsted acid towards alkynyl groups. This can be attributed to the poor electron-withdrawing capability of azaarenes^36–39^ and the greater stability of triple bonds compared to double bonds (Scheme 1B, right). Notably, Zhang and colleagues have attempted this transformation using a palladium/Xiao-Phos catalytic system.^27^ Aside from the reliance on precious metals, a significant limitation is the narrow substrate scope, particularly as the *tert*-butyl substituent on the SPOs appears critical for achieving sufficient enantiofacial differentiation. Consequently, the exploration of a more economical and broadly applicable catalytic platform represents an important and challenging endeavor.

Here, we present the successful application of a Cu(OAc)_2_ and Ph-pybox L1 complex as an efficient chiral catalyst (Scheme 1C). This system demonstrates exceptional compatibility with various SPOs and ethynylazaarenes, facilitating the synthesis of diverse *P*-chiral products with high yields and enantioselectivities. CuOAc has also been found to be effective for this transformation. By employing sulfur-conjugate addition and dihydroxylation as representative derivatization reactions, we can introduce one or two central chirality elements onto the ethylene scaffold, significantly enhancing the diversity of such important *P*-chiral 1,5-hybrid *P*,*N*-ligands. Comprehensive investigations, including control experiments and density functional theory (DFT) calculations, have elucidated a kinetic resolution (KR) strategy and revealed a plausible mechanism for this asymmetric copper-catalyzed process. Notably, the interaction between the chiral Cu(i) complex and the nitrogen atom of azaarenes plays a crucial role in activating the transformation, facilitating sufficient enantiofacial differentiation. Understanding this mechanism is crucial for expanding the application of this catalytic platform in synthesizing enantiomerically enriched azaarene derivatives from simple azaarene feedstocks.^40–57^

## Results and discussion

Our study was commenced with the selection of racemic isopropyl(phenyl)phosphine oxide (1) and 2-ethynylpyridine (2) as model substrates (Table 1). The initial reaction was conducted using 10 mol% Co(OAc)_2_ and 11 mol% *tert*-butyl-pybox L2 in CH_2_Cl_2_ at 25 °C under a nitrogen atmosphere (entry 1). However, no reaction was observed. A similar outcome was noted when Fe(OAc)_2_ was employed in place of the cobalt salt (entry 2). We subsequently investigated the potential of inorganic salts derived from copper, a widely utilized and cost-effective transition metal. Our findings revealed that CuOAc afforded the desired product 3 in 65% yield with 15% enantiomeric excess (ee) (entry 3). This promising result prompted us to evaluate various chiral ligands, such as L1, L3, and L4 (entries 4–6). Notably, the pybox^58^ framework of ligand L1 provided the best outcome, yielding 3 in 76% with 55% ee (entry 4). In contrast, ligand L4, derived from a chiral diamine, resulted in a remarkably sluggish chemical conversion, producing 3 in only 22% yield (entry 6). We then assessed other commonly used Cu(i) salts, including CuI and CuCl, but observed extremely poor enantioselectivity (entries 7 and 8). Cu(OAc)_2_ as the Cu(ii) salt was subsequently examined (entry 9), resulting in a similar enantioselectivity to CuOAc but with a slightly improved yield of 78% for product 3. A range of solvents, including toluene, CH_3_CN, and DCE, was screened (entries 10–12), with DCE proving to be the most effective, resulting in 82% yield and 60% ee for 3 (entry 12). Lowering the temperature to 0 °C slowed the reaction, extending the time from 36 h to 72 h; however, this adjustment enhanced the ee to a promising 85% (entry 13). Additionally, increasing the catalyst loading to 20 mol% improved the yield from 56% to 64% and the ee to 89% (entry 14). To further enhance chemical conversion, we increased the equivalent of 2 to 2.0, leading to the adduct 3 being obtained in 92% yield with 96% ee (entry 15). This higher enantioselectivity suggests the probability for KR within the reaction system, as a higher concentration of the KR-favorable enantiomer can effectively diminish the participation of the unfavorable enantiomer in the addition reaction. Given the comparable enantioselectivity observed with CuOAc in entries 4 and 9, it was selected as the catalyst for the current conditions, resulting in the formation of adduct 3 with a yield of 82% and an ee of 92% (entry 16). This outcome suggests that the Cu(ii) complex serves as a more effective chiral catalyst. Importantly, the presence of copper salt was found to be indispensable for the progression of the reaction (entry 17). When L1 was omitted from the reaction mixture, negligible product formation was observed, highlighting that this chiral ligand significantly enhances the catalytic ability of Cu(OAc)_2_ (entry 18). Furthermore, conducting the transformation under a nitrogen atmosphere proved to be crucial for achieving high yields and enantioselectivity (entry 19).

**Table 1:** Optimization of the reaction conditions^a^

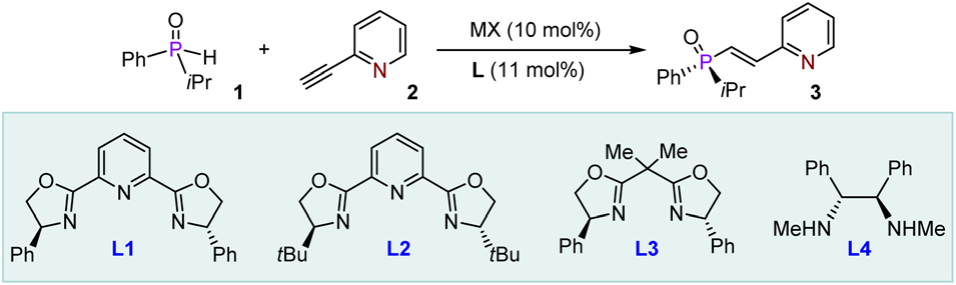						
Entry	MX	L	Solvent	*T* [°C]	Yield^b^ [%]	ee^c^ [%]
1	Co(OAc)_2_	L2	CH_2_Cl_2_	25	N.R.	N.A.
2	Fe(OAc)_2_	L2	CH_2_Cl_2_	25	N.R.	N.A.
3	CuOAc	L2	CH_2_Cl_2_	25	65	15
4	CuOAc	L1	CH_2_Cl_2_	25	76	55
5	CuOAc	L3	CH_2_Cl_2_	25	53	3
6	CuOAc	L4	CH_2_Cl_2_	25	22	11
7	CuI	L1	CH_2_Cl_2_	25	76	12
8	CuCl	L1	CH_2_Cl_2_	25	74	6
9	Cu(OAc)_2_	L1	CH_2_Cl_2_	25	78	55
10	Cu(OAc)_2_	L1	Toluene	25	45	6
11	Cu(OAc)_2_	L1	CH_3_CN	25	76	52
12	Cu(OAc)_2_	L1	DCE	25	82	60
13	Cu(OAc)_2_	L1	DCE	0	56	85
14^d^	Cu(OAc)_2_	L1	DCE	0	64	89
15^d^^e^	Cu(OAc)_2_	L1	DCE	0	92	96
16^d^^e^	CuOAc	L1	DCE	0	82	92
17^d^^e^	—	L1	DCE	0	N.R.	N.A.
18^d^^e^	Cu(OAc)_2_	—	DCE	0	Trace	N.A.
19^d^^e^^f^	Cu(OAc)_2_	L1	DCE	0	38	82

a Reaction conditions: 1 (0.1 mmol), 2 (0.1 mmol), solvent (1.0 mL), N_2_ atmosphere. Entries 1–12: *t* = 36 h. Entries 13–18: *t* = 72 h.

b Yield was isolated by flash column chromatography on silica gel.

c Determined by HPLC analysis on a chiral stationary phase.

d 20 mol% Cu(OAc)_2_ and 22 mol% L1 were used.

e 0.2 mmol 1 was used.

f In air. N.R. = no reaction. N.A. = not applicable. DCE = dichloroethane.

With the optimized reaction conditions in hand, we investigated the substrate scope for this asymmetric hydrophosphinylation of ethynylazaarenes using the chiral copper catalytic platform. Initially, a range of racemic alkyl–aryl-substituted SPOs were attempted to react with 2-ethynylpyridine 2 (Table 2). Consequently, corresponding products 3–22 were obtained in yields ranging from 72% to 99%, with ees between 86% and 96%. It was observed that introducing diverse electron-withdrawing or donating groups on the aromatic rings of SPOs (4–14) generally resulted in excellent enantioselectivities. The successful formation of products with fused aromatic (15) and heteroaromatic (16) rings as substituents further verifies the generality of this method. Importantly, various linear alkyls with higher steric hindrance (*e.g.*, 17) and lower steric hindrance (*e.g.*, 18 and 19), as well as cyclic alkyls (*e.g.*, 20–22), exhibited good tolerance towards the catalytic system. Notably, when iso-propyl was replaced by *tert*-butyl, the reaction became significantly sluggish, and almost no product could be obtained due to considerable steric hindrance.

**Table 2:** Reactions between aryl–alkyl-based SPOs with 2-ethynylpyridine^a^

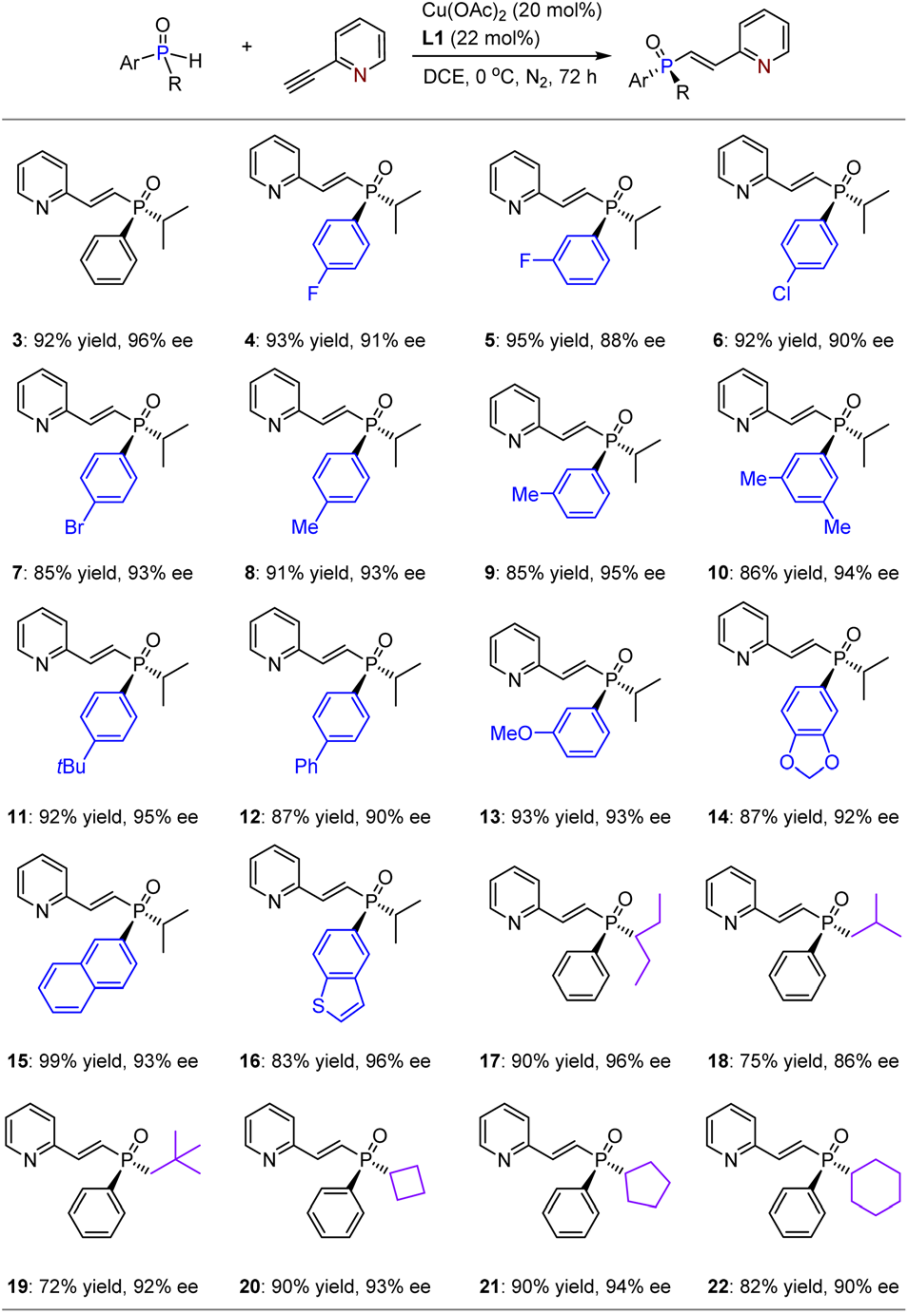

a The reaction was performed on a 0.10 mmol scale.

We subsequently conducted transformations of racemic isopropyl(phenyl)phosphine oxide (1) with various 2-ethynylpyridines, encompassing diverse electron-withdrawing or electron-donating groups on the 3-, 4-, and 5-positions of the pyridiyl rings (Table 3). The resulting products 23–40 were obtained in yields ranging from 81% to 96%, exhibiting ees between 86% and 96%. Notably, the presence of strong electron-withdrawing groups such as esters (24, 31, and 36) and nitrile (38) did not exert any influence on the chemo- and enantioselective outcomes. Furthermore, despite significant steric hindrance encountered when substituents were introduced at the 5-position, products 38–40 could still be synthesized with yields ranging from 84% to 96%, accompanied by ees between 86% and 93%. Importantly, this methodology proves effective for constructing bioactive molecular frameworks (*e.g.*, nikethamide, nerol, and gemfibrozil) based on *P*-chiral SPO derivatives (35–37), thereby robustly facilitating its application in the pharmaceutical industry, given that TPOs and their derivatives are also widely present in biologically important compounds. Encouraged by this success, further investigations were conducted on other azaarene-activated acetylenes. To our delight, in addition to isoquinoline, other significant azaarenes containing two nitrogen atoms on the aromatic rings exhibited compatibility as well. All products 42–44 could be synthesized with exceptional yields and ees. It is noteworthy that, despite the chiral copper complex having a relatively lower activation effect on 3-ethynyl-isoquinoline compared to 1-ethynyl-isoquinoline (41), it still provided product 42 with a yield of 76% and an ee of 98%, demonstrating the high efficiency of this catalytic platform in achieving enantiocontrol. Finally, diaryl-substituted SPOs were tested, resulting in product 45 being obtained with a yield of 78% and an ee of 72%. The relatively low enantioselectivity may be attributed to the potential remote enantiocontrol exerted by the chiral catalyst (*vide infra*), which complicates the differentiation of SPOs with two aryl groups that exhibit insufficient steric hindrance differences.

**Table 3:** Reactions between SPOs with ethynylazaarenes^a^

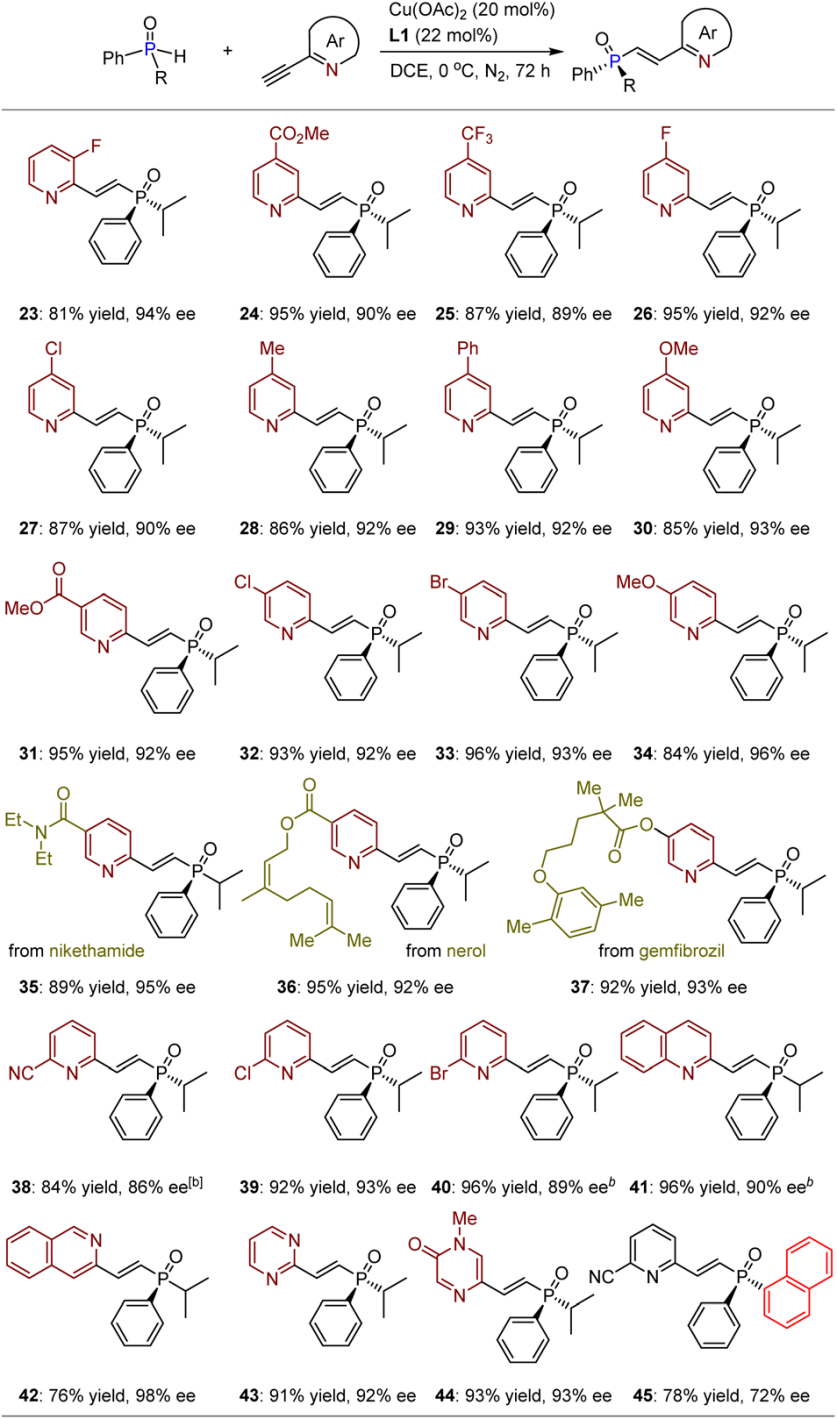

a The reaction was performed on a 0.10 mmol scale.

b *T* = −10 °C.

**Scheme 2:**
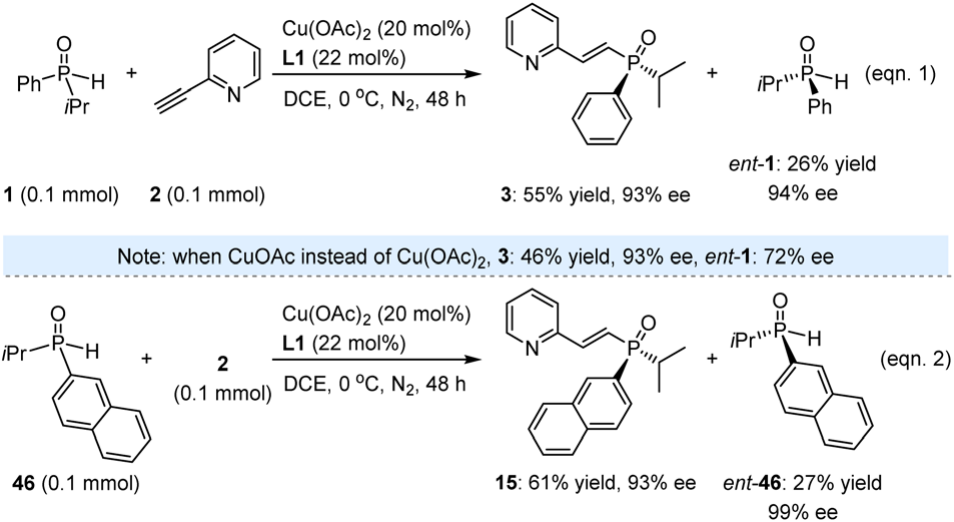
Investigation on kinetic resolution.

While the high yields and excellent ees of these valuable products underscore the successful execution of this task, we remain intrigued by the method's potential for KR. Beyond evaluating its applicability in synthesizing enantioenriched SPOs, this approach may also provide insights into the underlying mechanism. Accordingly, 1.0 equivalent of racemic 1 was reacted with 2-ethynylpyridine (2) under the established reaction conditions. The resulting product 3 was obtained in 55% yield with 93% ee after 48 h, while *ent*-1 was recovered in 26% yield with 94% ee (eqn (1), Scheme 2). The reaction proceeded sluggishly in the presence of CuOAc instead of Cu(OAc)_2_, resulting in the formation of *ent*-1 with an ee of only 72%. Additionally, we investigated SPO 46, which featured a 2-naphthyl substituent in place of phenyl, yielding product 45 in 61% yield with 93% ee, and *ent*-46 was recovered in 27% yield with 99% ee (eqn (2)). It is noteworthy that the moderate yields of the recovered SPOs can be attributed to several side reactions occurring within the catalytic system, leading to the formation of unknown byproducts. Nevertheless, the high enantioselectivity observed for both *ent*-1 and *ent*-46 underscores the feasibility of this method for accessing enantioenriched SPOs through kinetic resolution.

**Scheme 3:**
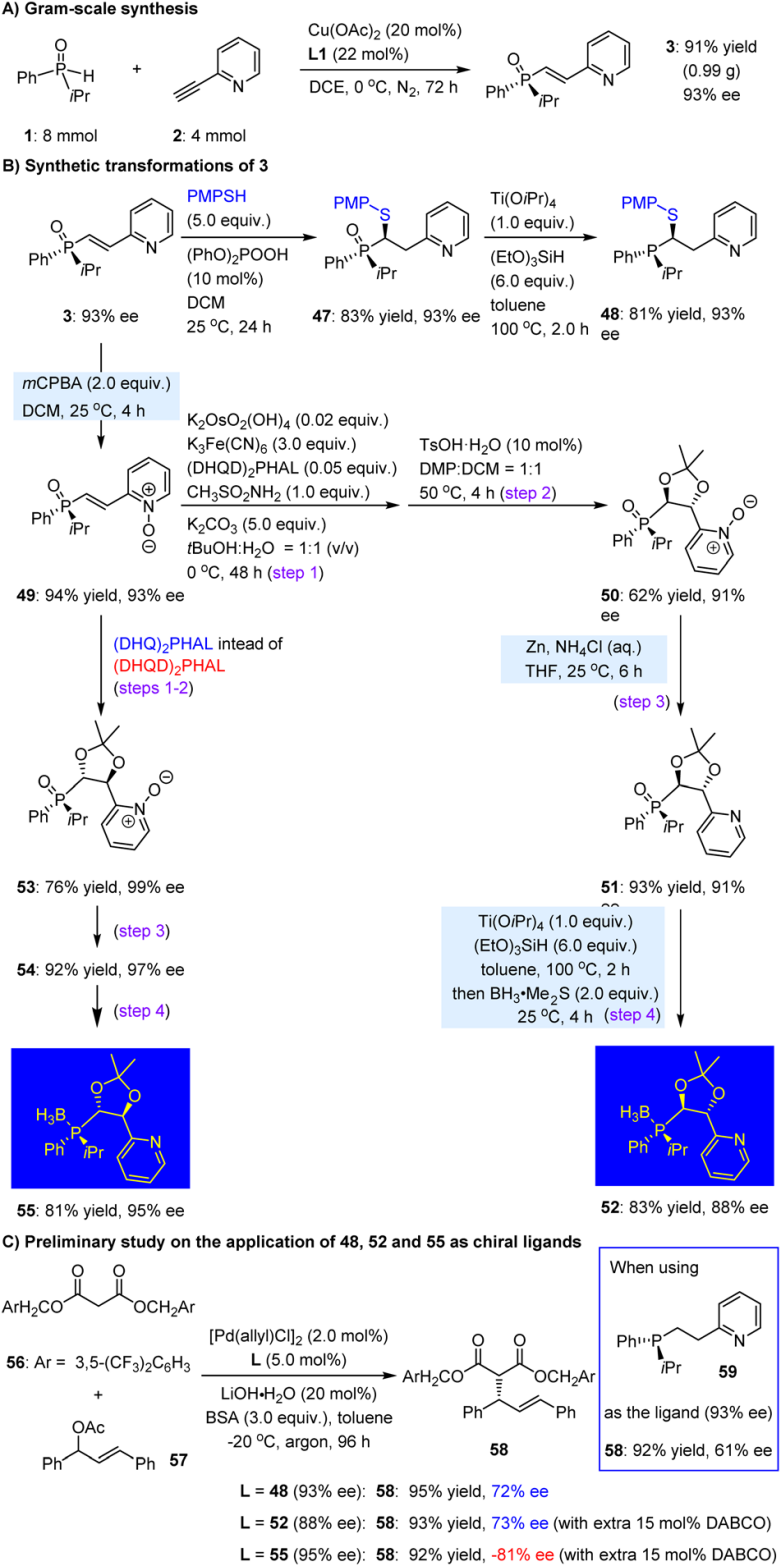
Synthetic utilities of the method and the obtained *P*-chiral secondary phosphine featuring central chirality.

We subsequently conducted a series of experiments to explore additional synthetic applications of this hydrophosphinylation strategy. As illustrated in Scheme 3A, the reaction of substrates 1 and 2 could be readily scaled up to 4.0 mmol, yielding product 3 in 91% yield (0.99 g) with 93% ee after 72 h. We then selected product 3 as a representative substrate for a sulfur-Michael reaction with diphenyl phosphate, utilizing 10 mol% of diphenylphosphate as the catalyst. This transformation occurred smoothly at 25 °C in dichloromethane, yielding the desired adduct 47 in 83% yield with 93% ee. Following reduction with (EtO)_3_SiH, the corresponding tertiary phosphine 48 was efficiently synthesized in 81% yield, with no loss in ee value (Scheme 3B).

In addition to constructing one stereocenter in the ethylene motif, we pursued dihydroxylation to introduce two secondary hydroxy groups, which could facilitate diverse transformations to create different chiral environments for the resulting *P*,*N*-ligands. To this end, 3 was first oxidized with *m*CPBA, yielding *N*-oxide 49 with high efficiency. This step was crucial for the subsequent dihydroxylation, as direct use of 3 led to poor chemoselectivity and no product formation. The Sharpless dihydroxylation^59^ of 49, using (DHQD)_2_PHAL as the chiral catalyst, was then performed, and the resulting product underwent further reaction with 2,2-dimethoxypropane (DMP) in the presence of 10 mol% TsOH, ultimately generating the protected dihydroxylation product 50. The *N*-oxide could be reduced smoothly by Zn/NH_4_Cl to yield pyridine derivative 51. The reduction using Ti(OiPr)_4_ and (EtO)_3_SiH was subsequently performed. Due to the susceptibility of the resulting tertiary phosphine to oxidation in the ambient atmosphere, a one-pot treatment with BH_3_·Me_2_S was investigated. This approach successfully yielded the *P*-chiral phosphine-BH_3_ adduct 52, featuring two tertiary carbon stereocenters, in 83% yield with 88% ee. Gratifyingly, when (DHQ)_2_PHAL was employed instead of (DHQD)_2_PHAL in the dihydroxylation reaction, product 53 was generated in 76% yield with 99% ee. It is worth noting that the dihydroxy groups in 53 exhibited an opposite absolute configuration compared to those in 50, thereby enhancing the diversity of this significant class of *N*,*P*-ligands. By following the same two-step reduction and protection sequence as employed for 50, the corresponding product 55 was obtained with a yield of 81% and ee of 95%.

To assess the potential of the newly formed carbon stereocenter in enhancing the enantioselectivity of *P*-chiral 1,5-*P*,*N* ligands, compound 48 was employed for the palladium-catalyzed Tsuji–Trost allylation of malonate nucleophile 56 with racemic 57 (Scheme 3C). Remarkably, initial tests utilizing our previously synthesized *P*-chiral molecule 59 (93% ee)^23^ yielded product 58 in a yield of 92% with an ee of 61%. Despite the flexible and unconstrained nature of the structure of 48, the ee value of product 58 improved to reach a level of 72%. Subsequently, we investigated chiral ligand derivative 52 in this reaction by employing DABCO as a cleavage agent for its borane protective group. It was found that the resulting product 58 exhibited enhanced enantioselectivity with an ee value of 73%, indicating a slight improvement when introducing one additional carbon-centered chirality on the carbon chain. Notably, as the epimer of 52, ligand 55 provided the enantiomer of 58 with 81% ee. The significance of incorporating carbon stereocenters into *P*-chiral 1,5-*P*,*N* ligands is further emphasized.

**Scheme 4:**
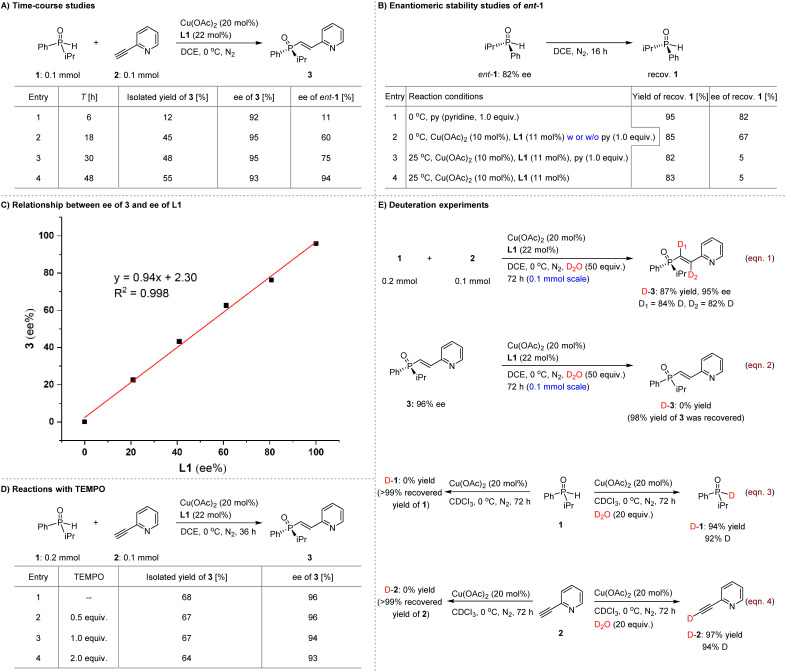
Mechanistic study.

To gain a deeper understanding of the KR event in the reaction system, we conducted the transformation of substrates 1 and 2 under the established reaction conditions, monitoring the ee values of both 3 and the recovered *ent*-1 over time (Scheme 4A). Notably, the ee of 3 remained at an excellent level, while its yield exhibited a progressive increase. Concurrently, the ee value of *ent*-1 continuously improved up to 48 hours. Importantly, the yields of both 3 and, particularly, 15 in this KR platform exceeded 50%, while maintaining excellent ees (Scheme 2). This observation suggests that SPO 1 could racemize in the reaction system, although the rate is slow, given the high ee of the recovered *ent*-1. In this context, *ent*-1, with an ee of 82%, was tested in the presence of 1.0 equivalent of pyridine (entry 1, Scheme 4B). The use of pyridine, rather than the pyridine-based olefin 2, was deliberate, aimed at creating a similar basic environment and preventing any reaction between *ent*-1 and 2 in the subsequent control experiments. The results indicated that *ent*-1 could be recovered in high yield while maintaining its ee. We then tested *ent*-1 under the established reaction conditions and found that the recovered *ent*-1 was obtained in 85% yield with an ee of 67% (entry 2). The addition of an additional 1.0 equivalent of pyridine yielded similar outcomes. However, at a temperature increase to 25 °C, the ee significantly decreased to only 5% (entry 3), even in the absence of pyridine (entry 4). The results suggest that the initial reaction of the copper complex with SPOs may form reaction intermediates, which will undergo addition to the alkynes. Meanwhile, these species can revert back to regenerate SPOs in an enantio-uncontrollable manner, resulting in racemization of the enantioenriched SPOs, particularly at room temperature. This is likely attributed to the significantly faster rate of conjugate addition compared to the putative reverse reaction, allowing for operational KR conditions. Moreover, racemization can occur for the kinetic-unfavorable enantiomer of SPOs, thereby ensuring high yield and ee values for the conjugate adducts in the KR-based reaction system (*i.e.*, 1 : 1 ratio of two substrates). Subsequently, the relationship between the ee of product 3 and the ee of L1 was investigated (Scheme 4C). The linear correlation result suggests that such a KR reaction should be solely mediated by a single molecule of the chiral copper complex.

As shown in Table 1, CuOAc exhibits comparable enantioselectivity to Cu(OAc)_2_. This observation leads us to hypothesize that the active species catalyzing the reaction may be the Cu(i) complex. This hypothesis was subsequently corroborated by a series of electron paramagnetic resonance (EPR) experiments, which demonstrated that both SPOs and alkynes can effectively reduce Cu(ii) to Cu(i) within the reaction system.^60^ In this context, we investigated the reactions between substrates 1 and 2 in the presence of various concentrations of TEMPO (2,2,6,6-tetramethylpiperidinyl-1-oxyl), ranging from 0.5 to 2.0 equivalents (Scheme 4D). Notably, the presence of TEMPO did not inhibit the reaction, resulting in yields and ees of product 3 that were consistent with those observed in the absence of the radical scavenger. These findings suggest that radical species do not play a critical role in these KR transformations.

We were also intrigued to investigate the source of the two hydrogen atoms incorporated into the double bond of the resulting products. To address this, we conducted the reaction of 1 with 2 in the presence of 50 equivalents of D_2_O (eqn (1), Scheme 4E). The product D-3 was obtained in 87% yield with 95% ee and exhibited high deuterium incorporation at both sp^2^ carbon atoms of the olefin moiety. Subsequently, we tested product 3 under the same reaction conditions but no deuterated derivative (D-3) was detected (eqn (2)). These results clearly indicate that protonation is responsible for the formation of the two new C–H bonds in the products. Furthermore, the ability of this method to precisely incorporate deuterium atoms using inexpensive D_2_O as a deuterium source underscores its significant potential applications in drug discovery. We then evaluated the reaction of 1 in the presence of 20 mol% Cu(OAc)_2_ in CDCl_3_ as the solvent, where it was recovered completely (left, eqn (3)). Upon adding 20 equivalents of D_2_O, D-1 was obtained in 94% yield with 92% deuterium incorporation (right, eqn (3)). Similar results were obtained for alkyne 2 (eqn (4)). Accordingly, in addition to facilitating single electron transfer, the presence of the Cu(ii) complex enables the efficient proton transfer between the two substrates, utilizing the protons present in the reaction system.

**Fig. 1:**
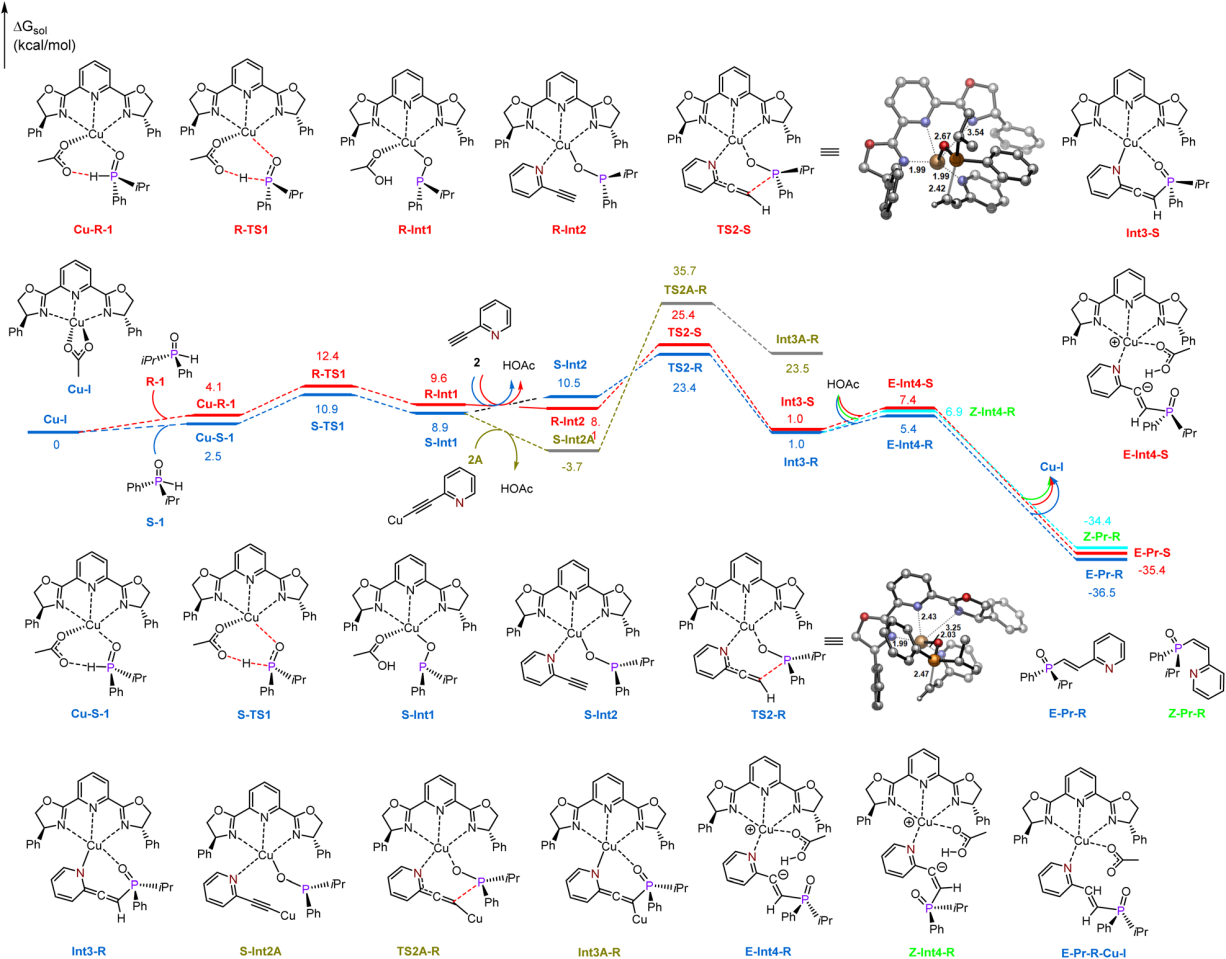
Free-energy profile for the Cu(i)-catalyzed hydrophosphorylation reaction between isopropyl(phenyl)phosphine oxide and 2-ethynylpyridine. The energies and bond distances are in kcal mol^−1^ and Å.

Based on these findings, DFT calculations were subsequently performed to elucidate the plausible mechanism underlying this intriguing reaction.^60^ As shown in Fig. 1, we chose the racemic isopropyl(phenyl)phosphine oxide (1) and 2-ethynylpyridine (2) as model substrates. All the pathways start with ligand-coordinated Cu(i) catalyst species Cu-I, which is derived from Cu(OAc)_2_ in the reaction. As previously mentioned, this transformation has been notably confirmed by EPR analysis.^60^ First, coordination of the pentavalent R-1 and S-1 to the copper center forms intermediates Cu-R-I and Cu-S-I. Tautomerization then generates two trivalent enantiomeric copper(i) phosphinous acids, R-Int1 and S-Int1, through transition states R-TS1 and S-TS1, which are endergonic by 9.6 and 8.9 kcal mol^−1^. The energy barriers for this step are 12.4 and 10.9 kcal mol^−1^. Subsequent ligand exchange with 2-ethynylpyridine 2 generates complexes R-Int2 and S-Int2, the direct nucleophilic addition of copper(i) phosphinous acids R-Int1 and S-Int1 to the C–C triple bond of 2-ethynylpyridine *via* transition states TS2-S and TS2-R with the energy barriers of 25.4 and 23.4 kcal mol^−1^, and the allenylic species Int3-S and Int3-R are generated.

Additionally, considering that terminal alkynes could undergo a deprotonation process to generate copper(i) acetylides, which is a reversible process (Fig. S6 in the ESI†), we also studied the nucleophilic addition of the copper(i) phosphinous acid S-Int1 to copper(i) acetylide *via* transition state TS2A-R., with an energy barrier of 39.4 kcal mol^−1^, which is 26.5 kcal mol^−1^ higher than that of the direct nucleophilic addition of 2-ethynylpyridine (TS2-R, 12.9 kcal mol^−1^), thus it could be excluded due to the high energy. Note that a similar behavior has been reported for copper, silver and nickel catalyzed reactions of terminal alkynes.^61–63^ Finally, with the coordination of HOAc to the copper center, the conformational change of the allenylic moiety could generate the vinyl anion complexes *E*-Int4-R, *Z*-Int4-R and *E*-Int-S, which are endergonic by 5.4, 6.9 and 7.4 kcal mol^−1^, and then undergo barrierless protonation, leading to the formation of final products *E*-Pr-R, *Z*-Pr-R and *E*-Pr-S, along with the Cu(i) catalyst species Cu-I.

The rate-determining step is the nucleophilic addition, and the enantioselectivity is also controlled in this step. The R-enantiomer Int3-R is generated by Re-face attack of the copper(i) phosphinous acid S-Int1*via* transition state TS2-R. Generation of the corresponding S-enantiomer Int3-S is accomplished by the corresponding Si-face attack. The energy barrier of R-enantiomer generation *via*TS2-R is 23.4 kcal mol^−1^, which is 2.0 kcal mol^−1^ lower than that of TS2-S, indicating that generation of R-enantiomer Int3-R is favorable. The calculated ee is 95.3%, which is in good agreement with the experimental value (96.0% ee). For the final protonation process, the energy of the less sterically hindered *E* isomer *E*-Int4-R is 5.4 kcal mol^−1^, which is 1.5 kcal mol^−1^ lower than that of *Z*-Int4-R. Therefore, the *E* isomer *E*-Pr-R is the major product.

**Fig. 2:**
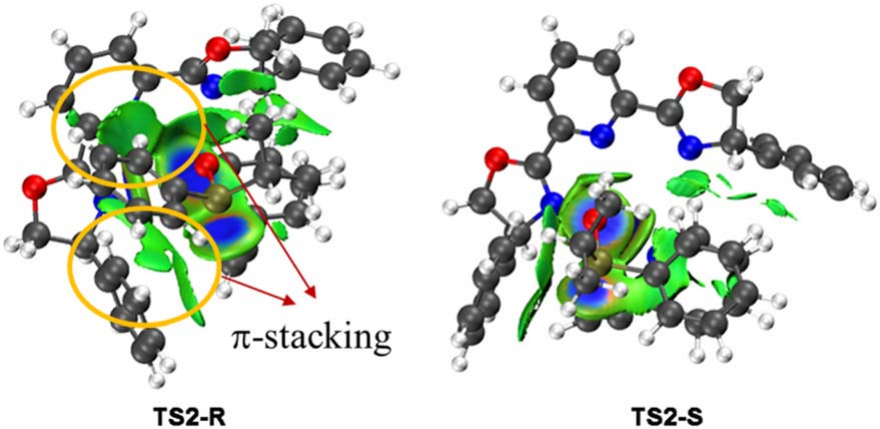
Non-covalent interaction (NCI) analysis of the two key transition states TS2-R and TS2-S (the blue, green, and red regions represent strong, weak, and repulsive interactions).

To investigate the origin of the enantioselectivity, NCI analysis of the nucleophilic addition transition states TS2-R and TS2-S was performed (Fig. 2). In transition states TS2-R and TS2-S, the N–Cu bond lengths are 2.03 and 1.99 Å, respectively, indicating that there are strong coordination interactions between the pyridine group of 2-ethynylpyridine and the copper center, which provide relative rigidity to the two transition states. Further, there are more π-stacking interactions between the phenyl group of the phosphinous acid moiety and the close dihydrooxazole and phenyl group of the chiral ligand in transition state TS2-R. Therefore, the coordination interactions between the pyridine group and the copper center lead to more rigid structures, and the enantioselectivity is mainly controlled by the π-stacking interaction of the nucleophilic addition transition state.

## Conclusions

In summary, we have developed an enantioselective hydrophosphinylation of ethynylazaarenes through the establishment of a chiral copper catalytic platform. This cost-effective method enables efficient reaction of various racemic SPOs, yielding *P*-chiral TPOs with one azaarene-functionalized olefin substituent in high yields and excellent enantioselectivities. In addition to its broad substrate scope, the facile modification of the olefin moiety renders these molecules valuable as significant chiral 1,5-hybrid *P*,*N*-ligands in asymmetric metal catalysis. The introduction of additional carbon stereocenters at the olefin position has been experimentally confirmed to significantly enhance enantioselectivity and even induce opposite enantiocontrol, leading to the formation of enantiomers with contrasting stereochemistry compared to species exhibiting solely *P*-chirality. Furthermore, this catalytic platform facilitates the kinetic resolution of SPOs. Mechanistic studies revealed that the interaction between the chiral copper complex and azaarenes is crucial for activating the transformation and achieving enantiofacial differentiation for remote SPOs. Additionally, the protonation process was observed for newly formed C–H bonds, which provides an important avenue for generating deuterated variants using inexpensive D_2_O as a deuterium source, highlighting the pharmaceutical significance of this method. We anticipate that this work will inspire rapid development of a broader array of *P*-chiral *P*,*N*-ligands, thereby advancing synthetic and pharmaceutical chemistry fields.

## Data availability

The data that support the findings of this study are available from the corresponding author upon reasonable request.

## Author contributions

Z. J. conceived and designed the experiments. J. Z. and R. X. conducted the experiments and prepared the ESI.† D. Z. and K. L. assisted in isolating the new compounds and analyzing the data. J. G. and S. C. performed the DFT calculations. Z. J. and S. C. authored the manuscript. J. Z. and J. G. contributed equally to this work. All authors engaged in discussions of the results and provided feedback on the manuscript.

## Conflicts of interest

There are no conflicts to declare.

## Supplementary Material

SC-OLF-D5SC00358J-s001

SC-OLF-D5SC00358J-s002
