# Supplementary material for: Asymmetric copper-catalyzed hydrophosphinylation of ethynylazaarenes to access P-chiral 2-azaaryl-ethylphosphine oxides
Source: Chem Sci. 2025 Mar 3;16(14):5957–66. doi: 10.1039/d5sc00358j (PMC11886619; doi:10.1039/d5sc00358j)
Supplement: SC-016-D5SC00358J-s001 [file SC-016-D5SC00358J-s001.pdf]

Supplementary Information (SI) for Chemical Science.
This journal is © The Royal Society of Chemistry 2025

Supporting Information

Asymmetric copper-catalyzed hydrophosphinylation of ethynylazaarenes to access P-chiral 2-azaaryl-ethylphosphine oxides

Jialiang Zhang,[a] Jiajia Guo,[a] Ruhui Xu,[a] Di Zheng,[a] Kai Lian,[a] Zhaoxia Zhang,[a] Shanshan Cao,[a]* and Zhiyong Jiang[a,b]*

[a]*Pingyuan Laboratory, School of Chemistry and Chemical Engineering, Henan Normal University, Xinxiang, Henan, P. R. China 453007*

[b]*College of Pharmacy, Henan University, Kaifeng, Henan, P. R. China 475004*

**E-mail: caoshanshan@htu.edu.cn (S.C.); jiangzhiyong@htu.edu.cn (Z.J.)*

Table of Contents

1. General information **S3-S4**

2. Optimization of reaction conditions **S5-S6**

3. General experimental procedures **S7**

4. Synthetic applications of enantioenriched tertiary phosphonates. **S8-S11**

5. Mechanism studies **S12-S21**

6. DFT calculation **S22-S104**

7. Determination of the absolute configurations **S105-S121**

8. Characterization of products **S122-S181**

9. Copies of NMR spectra **S182-S263**

10. References **S264-S265**

1. General information

General procedures and methods

Experiments involving moisture and/or air sensitive components were performed under a positive pressure of argon in oven-dried glassware equipped with a rubber septum inlet. Dried solvents and liquid reagents were transferred by oven-dried syringes or hypodermic syringes cooled to ambient temperature in a desiccator. Reaction mixtures were stirred in 10 mL sample vial with Teflon-coated magnetic stirring bars unless otherwise stated. Moisture in non-volatile reagents/compounds was removed in high *vacuo* by means of an oil pump and subsequent purging with nitrogen. Solvents were removed *in vacuo* under ~30 mmHg and heated with a water bath at 40 – 45 °C using rotary evaporator with aspirator. The condenser was cooled with running water at 0 °C.

All experiments were monitored by analytical thin layer chromatography (TLC). TLC was performed on pre-coated plates, 60 F_{254}. After elution, plate was visualized under UV illumination at 254 nm for UV active material. Further visualization was achieved by staining $Ce(SO_4)_2$ and phosphomolybdic acid solution. For those using the aqueous stains, the TLC plates were heated on a hot plate.

Columns for flash chromatography (FC) contained *silica gel* 200–300 mesh. Columns were packed as slurry of *silica gel* in petroleum ether and equilibrated solution using the appropriate solvent system. The elution was assisted by applying pressure of about 2 atm with an air pump.

Instrumentations

Proton nuclear magnetic resonance (^{1}H NMR) and carbon NMR (^{13}C NMR) were recorded in $CDCl_3$ otherwise stated. Chemical shifts are reported in parts per million (ppm), using the residual solvent signal as an internal standard: $CDCl_3$ (^{1}H NMR: δ 7.26, singlet; ^{13}C NMR: δ 77.16, triplet). Multiplicities were given as: *s* (singlet), *d* (doublet), *t* (triplet), *q* (quartet), *quintet*, *m* (multiplets), *dd* (doublet of doublets), *dt* (doublet of triplets), and *br* (broad). Coupling constants (*J*) were recorded in hertz (Hz). The number of proton atoms (*n*) for a given resonance was indicated by *n*H. The number of carbon atoms (*n*) for a given resonance was indicated by *n*C. HRMS (Analyzer: TOF) was reported in units of mass of charge ratio (m/z). Mass samples were dissolved in CH_3OH (HPLC Grade) unless otherwise stated. Optical rotations were recorded on a polarimeter with a sodium lamp of wavelength 589 nm and reported as follows:

$[\alpha]_{\lambda}^{T°C}$ (c = g/100 mL, solvent). Melting points were determined on a melting point apparatus. Enantiomeric excesses were determined by chiral High Performance Liquid Chromatography (HPLC) analysis. UV detection was monitored at 254 nm and 210 nm at the same time. HPLC samples were dissolved in HPLC grade isopropanol (IPA) unless otherwise stated.

Materials

All commercial reagents were purchased with the highest purity grade. They were used without further purification unless specified. All solvents used, mainly petroleum ether (PE) and ethyl acetate (EtOAc) were distilled. Anhydrous dichloromethane (CH_2Cl_2), CH_3CN, $CHCl_3$ were freshly distilled from CaH_2 and stored under N_2 atmosphere. Tetrahydrofuran (THF) was freshly distilled from sodium/benzophenone before use. All compounds synthesized were stored in a −20 ºC freezer and light-sensitive compounds were protected with aluminium foil.

2. Optimization of reaction conditions

Table S1. **Optimization of the reaction conditions.**[a]

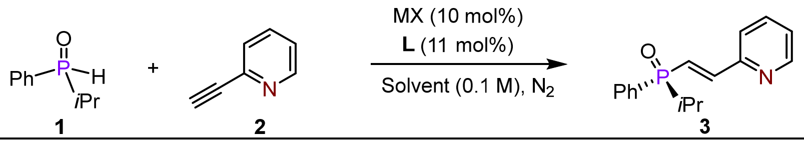

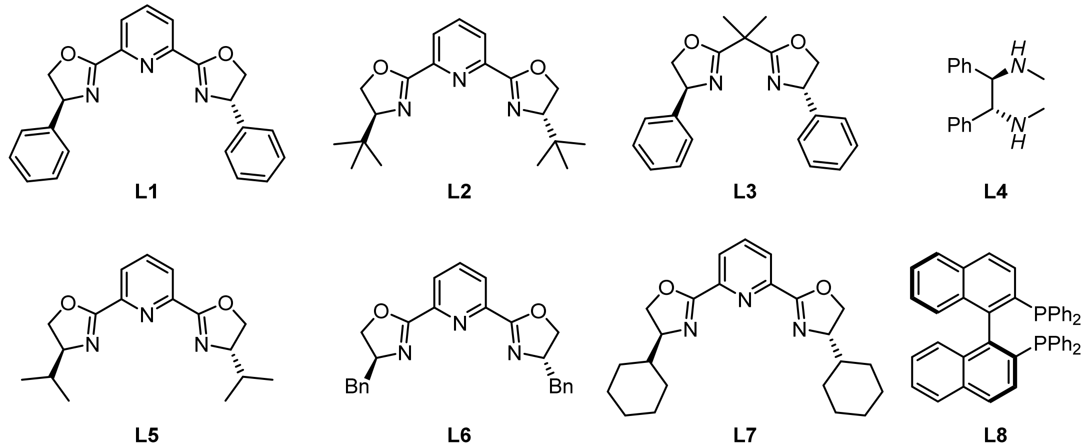

Entry	MX [mol%]	L [mol%]	Solvent	T [°C]	t [h]	Yield [%][b]	ee [%][c]
1	$Co(OAc)_2$ [10]	**L2** [11]	CH_2Cl_2	25	36	N.R.	N.A.
2	$Fe(OAc)_2$ [10]	**L2** [11]	CH_2Cl_2	25	36	N.R.	N.A.
3	CuOAc [10]	**L2** [11]	CH_2Cl_2	25	36	65	15
4	CuOAc [10]	**L1** [11]	CH_2Cl_2	25	36	76	55
5	CuOAc [10]	**L3** [11]	CH_2Cl_2	25	36	53	3
6	CuOAc [10]	**L4** [11]	CH_2Cl_2	25	36	22	11
7	CuOAc [10]	**L5** [11]	CH_2Cl_2	25	36	52	2
8	CuOAc [10]	**L6** [11]	CH_2Cl_2	25	36	57	8
9	CuOAc [10]	**L7** [11]	CH_2Cl_2	25	36	62	14
10	CuOAc [10]	**L8** [11]	CH_2Cl_2	25	36	17	0
11	CuI [10]	**L1** [11]	CH_2Cl_2	25	36	76	12
12	CuCl [10]	**L1** [11]	CH_2Cl_2	25	36	74	6
13	CuOTf [10]	**L1** [11]	CH_2Cl_2	25	36	36	2
14	CuTc [10]	**L1** [11]	CH_2Cl_2	25	36	65	54
15	$CuCl_2$ [10]	**L1** [11]	CH_2Cl_2	25	36	60	7
16	$Cu(OTf)_2$ [10]	**L1** [11]	CH_2Cl_2	25	36	27	5
17	$Cu(OAc)_2$ [10]	**L1** [11]	CH_2Cl_2	25	36	78	56
18	$Cu(acac)_2$ [10]	**L1** [11]	CH_2Cl_2	25	36	64	47
19	$Cu(OAc)_2$ [10]	**L1** [11]	PhCl	25	36	43	15
20	$Cu(OAc)_2$ [10]	**L1** [11]	CH_3Cl	25	36	62	38
21	$Cu(OAc)_2$ [10]	**L1** [11]	PhMe	25	36	45	6
22	$Cu(OAc)_2$ [10]	**L1** [11]	CH_3CN	25	36	76	52
23	$Cu(OAc)_2$ [10]	**L1** [11]	DME	25	36	47	4
24	$Cu(OAc)_2$ [10]	**L1** [11]	THF	25	36	24	3
25	$Cu(OAc)_2$ [10]	**L1** [11]	DCE	25	36	82	60
26	$Cu(OAc)_2$ [10]	**L1** [11]	DCE	10	48	74	76

27	$Cu(OAc)_2$ [10]	**L1** [11]	DCE	0	72	56	85
28	$Cu(OAc)_2$ [15]	**L1** [16]	DCE	0	72	58	87
29	$Cu(OAc)_2$ [20]	**L1** [22]	DCE	0	72	64	89
30[d]	$Cu(OAc)_2$ [20]	**L1** [22]	DCE	0	72	92	96
31[d]	CuOAc [20]	**L1** [22]	DCE	0	72	82	92
32[de]	$Cu(OAc)_2$ [20]	--	DCE	0	72	trace	N.A.
33[de]	--	**L1** [22]	DCE	0	72	N.R.	N.A.
34[d]	$Cu(OAc)_2$ [20]	**L1** [22]	DCE	0	72	38	82

[a] Reaction conditions: **1** (0.1 mmol), **2** (0.1 mmol), solvent (1.0 mL), N_2 atmosphere. [b] Yield was isolated by flash column chromatography on silica gel. [c] Determined by HPLC analysis on a chiral stationary phase. [d] 0.2 mmol **1** was used. [e] On air. N.R. = no reaction. N.A. = not applicable. DCE = dichloroethane.

3. General experimental procedures

General experimental procedure for P-Chiral 2-Azaaryl-Ethylphosphine Oxides.

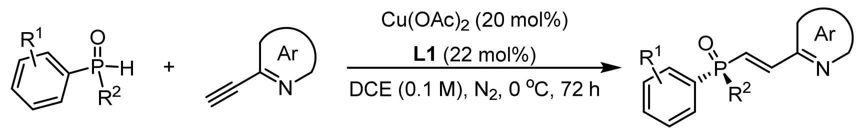

To a 10 mL Schlenk tube, $Cu(OAc)_2$ (0.02 mmol, 0.2 equiv.) and ligand **L1** (0.022 mmol, 0.22 equiv.) were added. Following this, DCE (1 mL) was introduced, and then alkyne (0.10 mmol, 1.0 equiv.) and SPO (0.20 mmol, 2.0 equiv.) were added. The reaction mixture was degassed twice using the freeze-pump-thaw method. The mixture was stirred under a nitrogen atmosphere at 0 °C for 72 hours. After the reaction, the solvent was removed under reduced pressure, and the residue was purified by flash chromatography on *silica gel* using a PE/EA solvent mixture in a ratio ranging from 5:1 to 1:2 to obtain the desired product.

4. Synthetic applications of enantioenriched tertiary phosphonates.

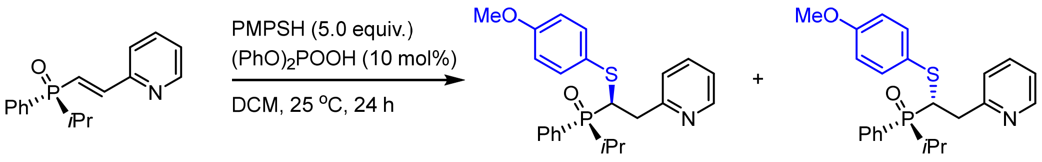

3: 93% ee **47**: 83% yield, 93% ee *ent*-**47**: 10% yield, 93% ee

__For 47__: To the solution of **3** (0.2 mmol, 1.0 equiv.) in CH_2Cl_2 (2 mL), $(PhO)_2POOH$ (0.02 mmol, 0.1 equiv.) and 4-methoxythiophenol (1.0 mmol, 5.0 equiv.) were added, the solution was stirred for 24 h at room temperature. The solvent was removed under reduced pressure, and the residue was purified by flash chromatography on silica gel (PE/EA = 5/1–1/1 ratio) to give the afforded product **47** and *ent*-**47**.

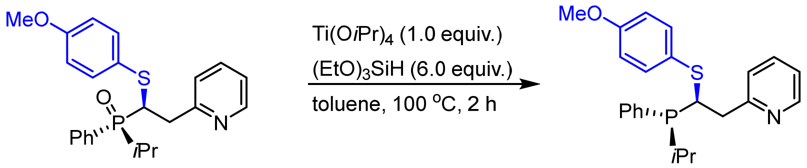

47: 93% ee **48**: 81% yield, 93% ee

__For 48__[1]: To a 10 mL vial equipped with a stirrer bar was added **47** (0.1 mmol) with the protection of N_2, $(EtO)_3SiH$ (0.6 mmol, 6.0 equiv.) and $Ti(O\textit{i}Pr)_4$ (0.1 mmol, 1.0 equiv.) was added after adding dry toluene (1.0 mL), and the reaction mixture was refluxed in oil bath at 100 °C . The reaction was monitored by TLC, after completion of the reaction, the mixture was directly loaded onto a short silica gel column, followed by gradient elution with petroleum ether/ethyl acetate (20/1–10/1 ratio) to affording the product **48**.

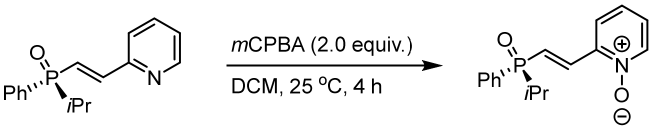

3: 93% ee **49**: 94% yield, 93% ee

__For 49__[2]: To a stirred solution of **3** (1 mmol) in DCM (20 mL) was added *m*CPBA (2.0 mmol, 2.0 equiv.) at 0 °C, and then the reaction warmed to room temperature for 4 h. After the reaction completed, the mixture was poured into water (20 mL) and saturated aq. sodium hydrogen carbonate (20 mL). The organic layer was separated and the aqueous phase was extracted with DCM (3 × 20 mL). The combined organic layers were dried (Na_2SO_4) and evaporated to give the crude product which was purified by flash chromatography with DCM/MeOH (100/1 – 20/1 ratio), removing the solvent in vacuo, afforded **49**.

.

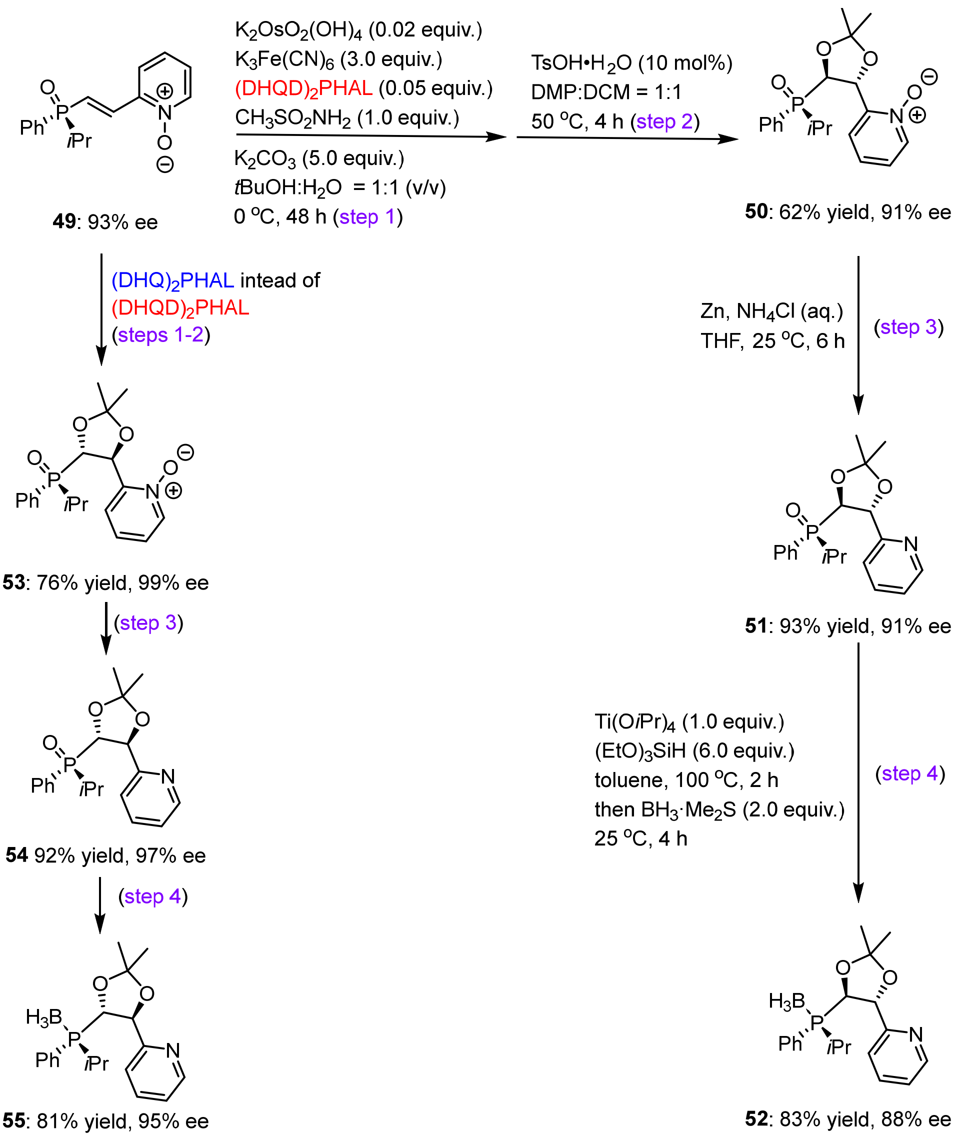

For 50[3]: A 10-mL round bottom flask filled with H_2O (2 mL) was cooled to 0 °C in an ice bath. Reagents $K_3[Fe(CN)_6]$ (0.6 mmol, 3 equiv.), K_2CO_3 (1.0 mmol, 5 equiv.), and $MeSO_2NH_2$ (0.2 mmol, 1.0 equiv.) were added, followed by $K_2[OsO_2(OH)_4]$ (0.004 mmol, 0.02 equiv.), $(DHQD)_2PHAL$ (0.001 mmol, 0.05 equiv.), **49** (0.2 mmol, 1 equiv.) and *t*BuOH (2 mL). The reaction mixture was stirred at 0 °C for 48 h. The solid was filtered off and washed with excess ethyl acetate. The organic layer was separated. The aqueous solution was concentrated to dryness, and the resulting solid was extracted with DCM. The above ethyl acetate and DCM solutions were combined and concentrated. and then the crude product was dissolved in DCM (2 mL) and DMP (2 mL), $TsOH{\cdot}H_2O$ (0.02 mmol, 0.1 equiv.) was added. The reaction mixture was stirred at 50 °C for 4 h. The solvent was removed under reduced pressure, and the residue

was purified by flash chromatography on silica gel DCM/MeOH (100/1 – 20/1 ratio) to give the **50**.

For 51: **50** (0.2 mmol) was dissolved in THF (2 mL). To this mixture was then added saturated NH_4Cl solution (2 mL) and zinc dust (1.0 mmol, 5.0 equiv.). This mixture was then stirred for 6 h. The deposit was then collected by filtration on Celite and washed with ethyl acetate (5 mL). The organic layer was then separated and the aqueous layer was extracted with ethyl acetate (3 x 5 mL). The combined organic layers dried (Na_2SO_4) and evaporated to give the crude product which was purified by flash chromatography with DCM/MeOH (100/1 – 40/1 ratio), removing the solvent in vacuo, afforded product **51**.

For 52: To a 10 mL vial equipped with a stirrer bar was added **51** (0.1 mmol) with the protection of N_2, $(EtO)_3SiH$ (0.6 mmol, 6.0 equiv.) and $Ti(O\textit{i}Pr)_4$ (0.1 mmol, 1.0 equiv.) was added after adding dry toluene (1.0 mL), and the reaction mixture was refluxed in oil bath at 100 °C for 2 h. Then $BH_3{\cdot}Me_2S$ (0.2 mmol, 2.0 equiv.) was added, after cool down to room temperature. The reaction was monitored by TLC, after completion of the reaction, the mixture was directly loaded onto a short silica gel column, followed by gradient elution with petroleum ether/ethyl acetate (20/1–10/1 ratio) to affording the product **52**.

For 53: according to step 1 and step 2.

For 54: according to step 3.

For 55: according to step 4.

Preliminary study on the use of P-chiral 1,5-P,N ligands[1]**.**

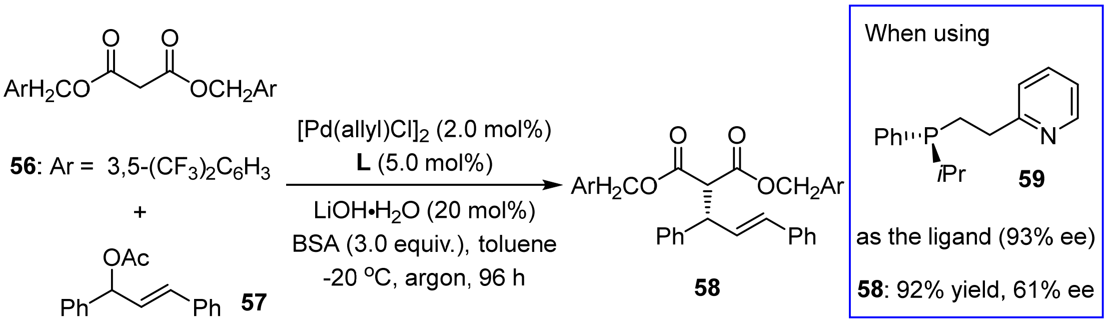

L = **48** (93% ee): **58**: 95% yield, 72% ee

L = **52** (88% ee): **58**: 93% yield, 73% ee (with extra 15 mol% DABCO)

L = **55** (95% ee): **58**: 92% yield, -81% ee (with extra 15 mol% DABCO)

48 as the chiral ligand: Chiral ligand **48** (5.0 mol%), $[Pd(\eta_3\text{-}C_3H_5)Cl]_2$ (2.0 mol%) and $LiOH{\bullet}H_2O$ (20 mol%) at room temperature under argon atmosphere were dissolved into toluene (2 mL). After stirring for 1 h, allylic ester **57** (0.1 mmol) was added, 20 min later, followed by BSA (0.3 mmol, 3.0 equiv.) and malonate **56** (0.3 mmol, 3.0 equiv.). The reaction mixture was stirred at –20 °C for 96 h. With the completion of the reaction monitored by TLC, water was added to the reaction mixture, DCM was employed to extract the product. The organic layer was dried over Na_2SO_4. The filtrate was concentrated and purified by column chromatography to afford **58**.

52 and 55 as the chiral ligand: To an oven-dried 10 mL vial charged with a stirring bar were added **52** or **55** (0.005 mmol, 0.05 equiv.), DABCO (0.015 mmol, 0.15 equiv.), and toluene (0.50 mL) in an argon-filled glovebox. The resulting solution was stirred for 24 h at room temperature, followed by the addition of $[Pd(\eta_3\text{-}C_3H_5)Cl]_2$ (2.0 mol%) and $LiOH{\bullet}H_2O$ (20 mol%). After stirring for 1 h, allylic ester **57** (0.1 mmol) in toluene (1.50 mL) was added, 20 min later, followed by BSA (0.3 mmol, 3.0 equiv.) and malonate **56** (0.3 mmol, 3.0 equiv.). The reaction mixture was stirred at –20 °C for 96 h. With the completion of the reaction monitored by TLC, water was added to the reaction mixture, DCM was employed to extract the product. The organic layer was dried over Na_2SO_4. The filtrate was concentrated and purified by column chromatography to afford **58**.

5. Mechanism studies

A) Time-course studies

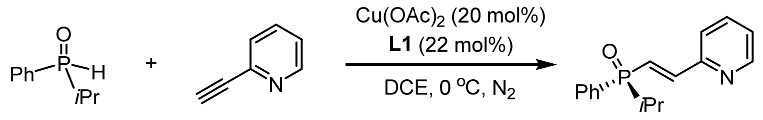

1: 0.1 mmol   2: 0.1 mmol   3

Entry	*T* [h]	Isolated yield of **3** [%]	ee of **3** [%]	ee of *ent*-**1** [%]
1	6	12	92	11
2	18	45	95	60
3	30	48	95	75
4	48	55	93	94

B) Enantiomeric stability studies of *ent*-1

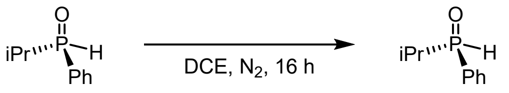

ent-**1**: 82% ee   recovered **1**

Entry	Reaction conditions	Yield of recov. **1** [%]	ee of recov. **1** [%]
1	0 °C, py (pyridine, 1.0 equiv.)	95	82
2	0 °C, $Cu(OAc)_2$ (10 mol%), **L1** (11 mol%) w or w/o py (1.0 equiv.)	85	67
3	25 °C, $Cu(OAc)_2$ (10 mol%), **L1** (11 mol%), py (1.0 equiv.)	82	5
4	25 °C, $Cu(OAc)_2$ (10 mol%), **L1** (11 mol%)	83	5

C) Relationship between ee of 3 and ee of L1

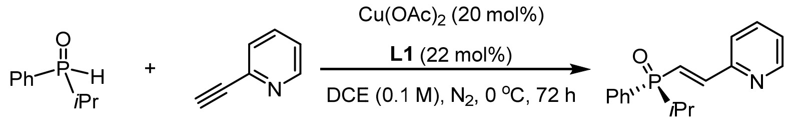

1: 0.1 mmol   2: 0.1 mmol   3

The ee of **L1** was determined by HPLC analysis: CHIRALPAK AD-H (4.6 mm i.d. × 150 mm); hexane/2-propanol = 70/30; flow rate 1.0 mL/min; 25 °C; 254 nm.

The ee of **3** was determined by HPLC analysis: CHIRALPAK IE (4.6 mm i.d. x 250 mm); hexane/2-propanol = 70/30; flow rate 1.0 mL/min; 25 °C; 254 nm

entry	1	2	3	4	5	6
ee (%) of **L1**	0	20.96	40.92	61.18	80.80	100
ee (%) of **3**	0	22.58	43.20	62.58	76.26	95.88

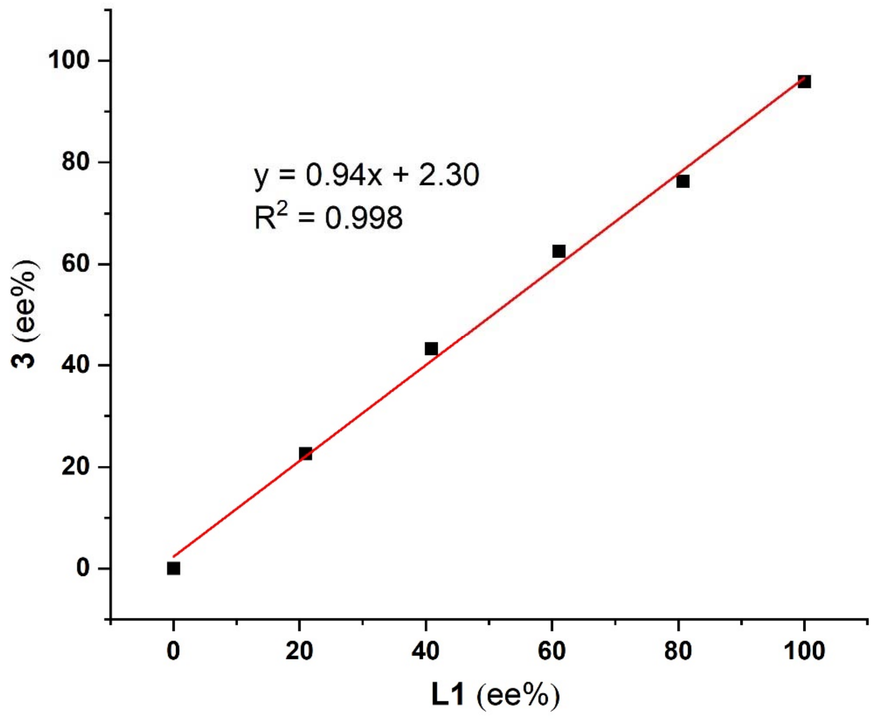

Fig. S1. Relationship between ee values of **3** and **L1**

HPLC spectra:

L1 0% ee

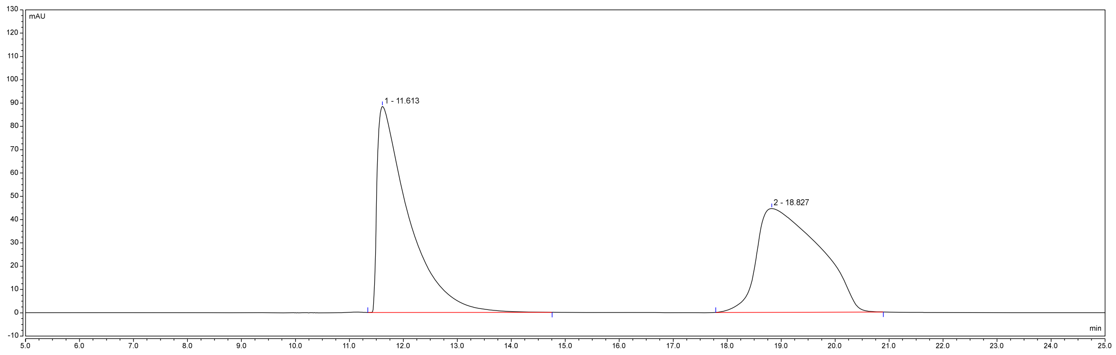

Entry	Retention Time	Area	Height	%Area
1	11.613	59.7858	88.37	49.92
2	18.827	59.9746	44.51	50.08

L1 20.96% ee

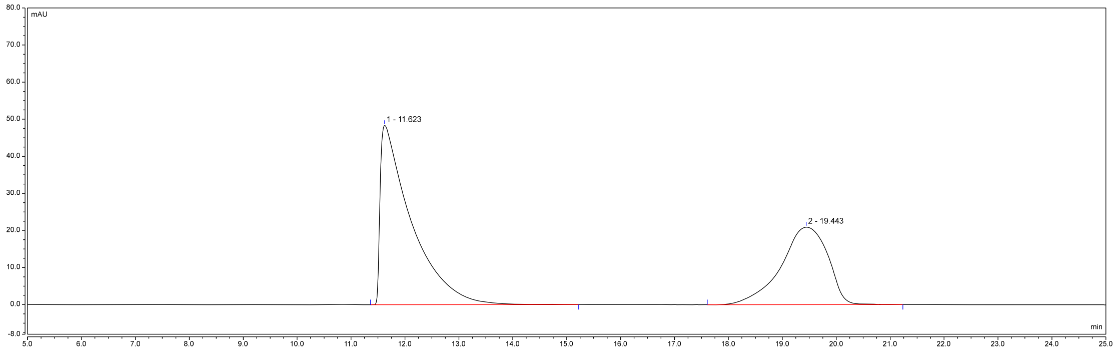

Entry	Retention Time	Area	Height	%Area
1	11.623	32.1705	48.36	60.48
2	19.443	21.0257	20.90	39.52

L1 40.92% ee

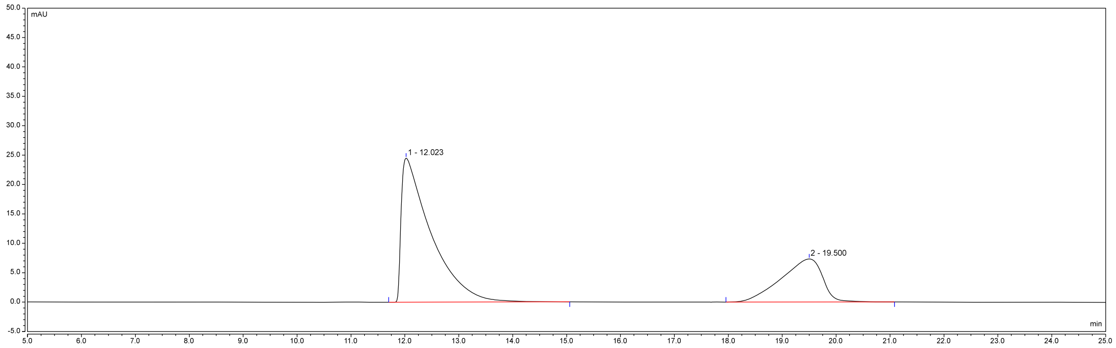

Entry	Retention Time	Area	Height	%Area
1	12.023	15.6123	24.49	70.46
2	19.500	6.5444	7.32	29.54

L1 61.18% ee

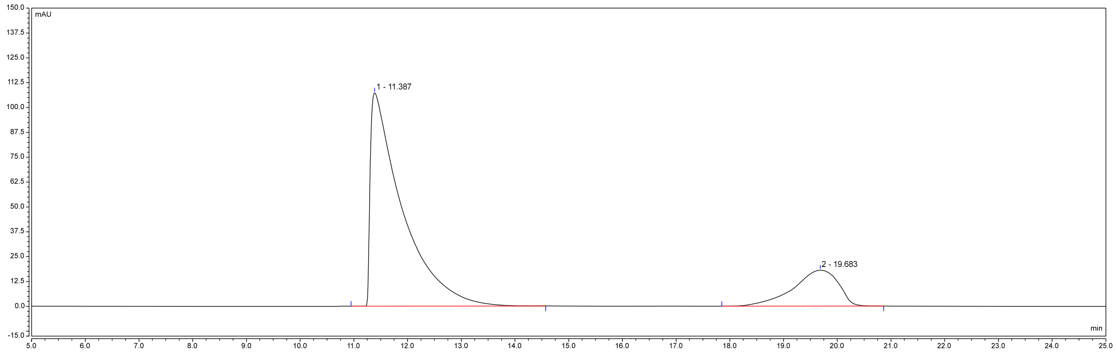

Entry	Retention Time	Area	Height	%Area
1	11.387	73.3374	107.30	80.59
2	19.683	17.6641	18.11	19.41

L1 80.80% ee

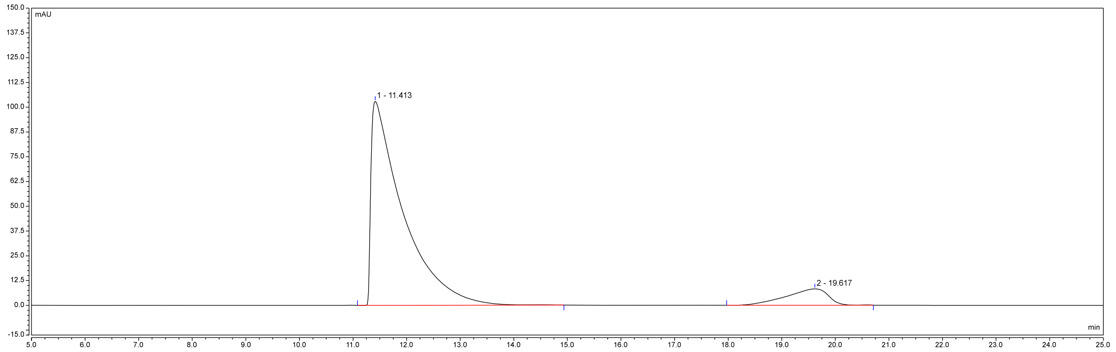

Entry	Retention Time	Area	Height	%Area
1	11.413	70.8458	102.93	90.40
2	19.617	7.5200	8.26	9.60

L1 100% ee

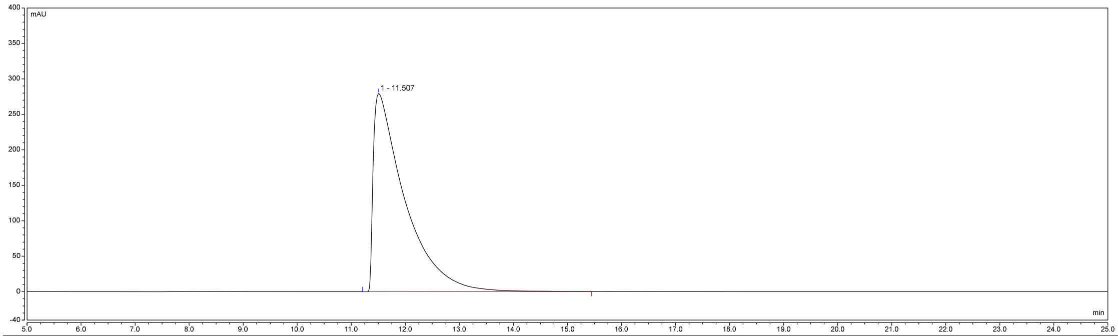

Entry	Retention Time	Area	Height	%Area
1	11.507	188.4142	279.14	100.00

3 0% ee

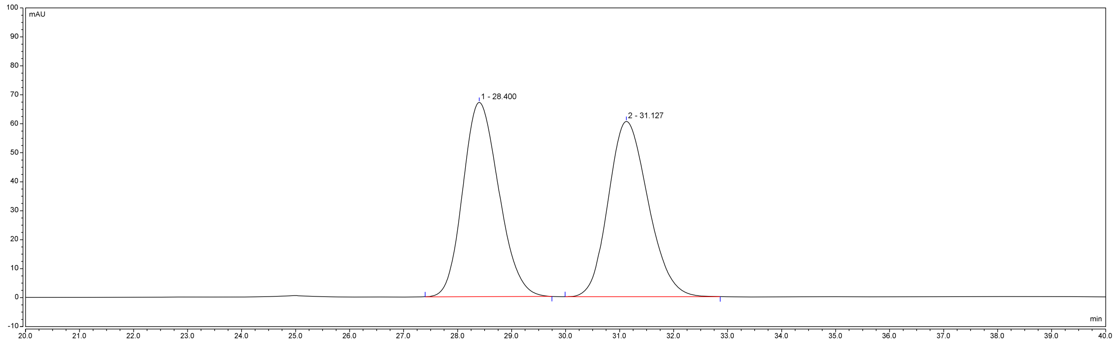

Entry	Retention Time	Area	Height	%Area
1	28.400	51.6754	67.03	49.99
2	31.127	51.6874	60.51	50.01

3 22.58% ee

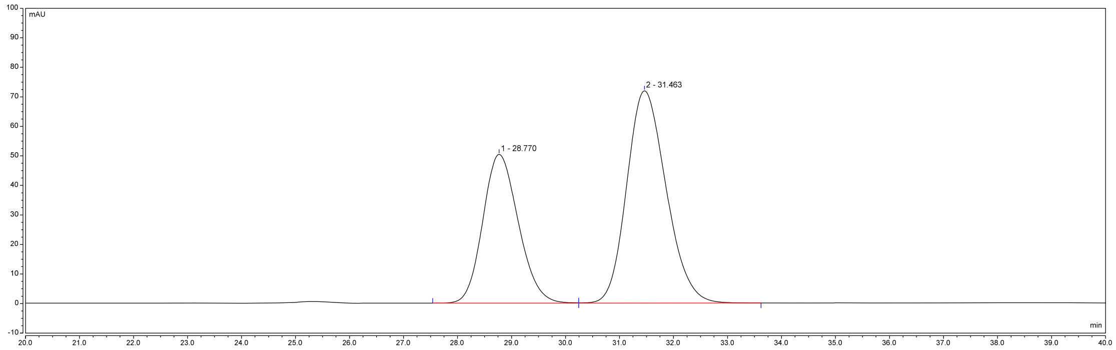

Entry	Retention Time	Area	Height	%Area
1	28.770	38.3987	50.37	38.71
2	31.463	60.7883	71.86	61.29

3 43.20% ee

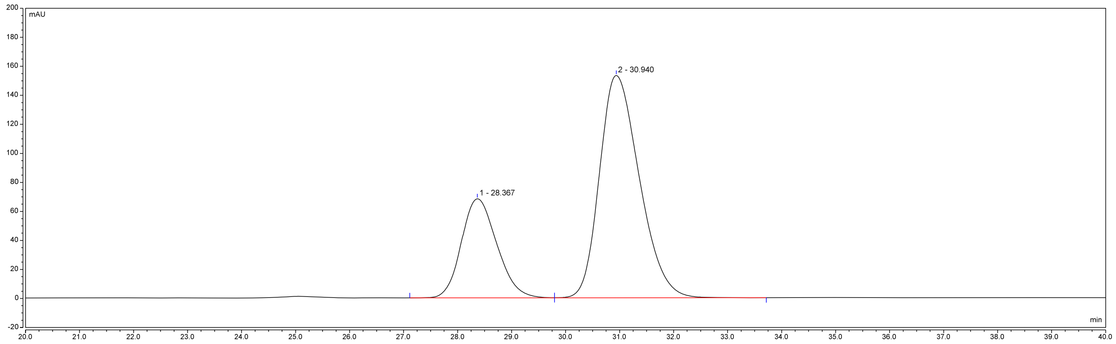

Entry	Retention Time	Area	Height	%Area
1	28.367	51.9127	68.28	28.40
2	30.940	130.8610	153.32	71.60

3 62.58% ee

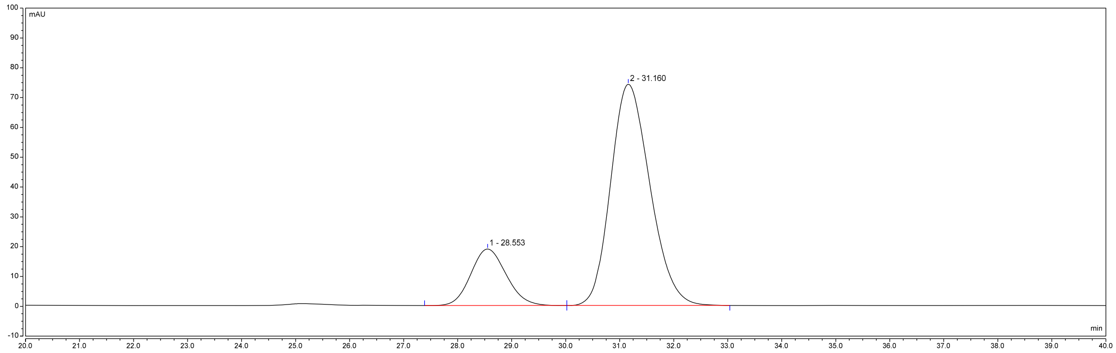

Entry	Retention Time	Area	Height	%Area
1	28.553	14.3730	18.94	18.71
2	31.160	62.4516	74.17	81.29

3 76.26% ee

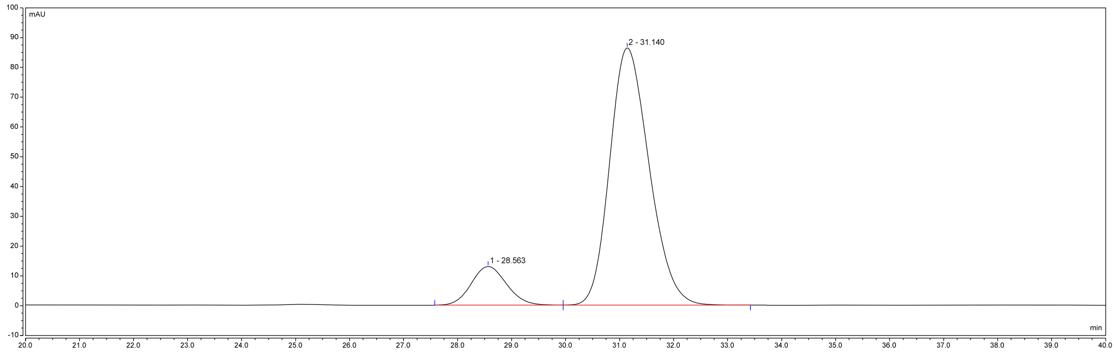

Entry	Retention Time	Area	Height	%Area
1	28.563	9.8416	12.97	11.87
2	31.140	73.0576	86.29	88.13

3 95.88% ee

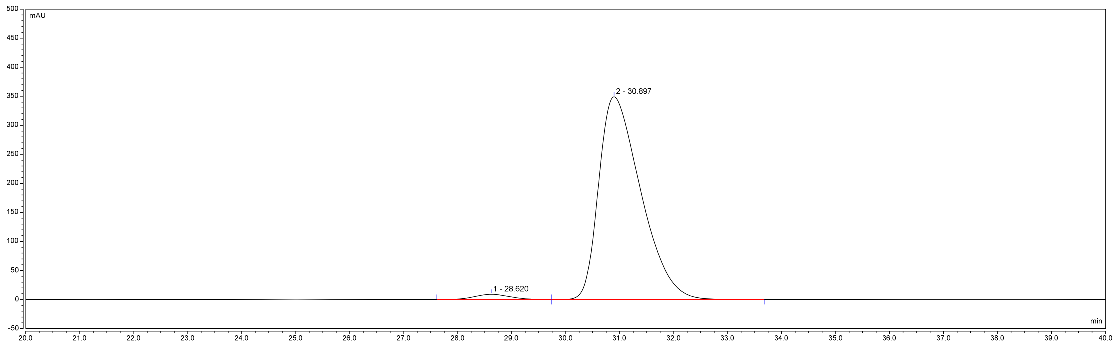

Entry	Retention Time	Area	Height	%Area
1	28.620	6.6059	8.88	2.06
2	30.897	313.3536	348.80	97.94

D) Reactions with TEMPO

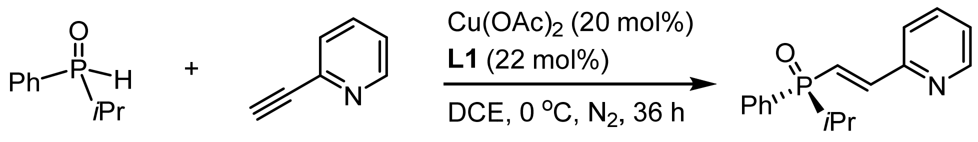

1: 0.2 mmol **2**: 0.1 mmol **3**

Entry	TEMPO	Isolated yield of **3** [%]	ee of **3** [%]
1	--	68	96
2	0.5 equiv.	67	96
3	1.0 equiv.	67	94
4	2.0 equiv.	64	93

E) Deuteration experiments

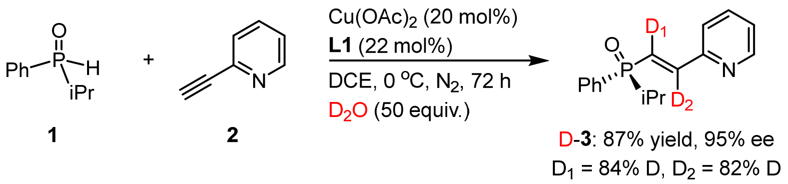

$Cu(OAc)_2$ (0.02 mmol, 0.2 equiv.), **L1** (0.022 mmol, 0.22 equiv.) was added into a 10 mL Schlenk tube, and DCE (1 mL) was added, followed by the addition of D_2O (50 equiv.), **2** (0.10 mmol, 1.0 equiv.) and **1** (0.20 mmol), immediately degassed twice by freeze-pump-thaw method. The reaction mixture was stirred under a nitrogen atmosphere at 0 °C for 72 h. The solvent was removed under reduced pressure, and the residue was purified by flash chromatography on silica gel (PE/EA = 5/1–1/2 ratio) to give D-**3**.

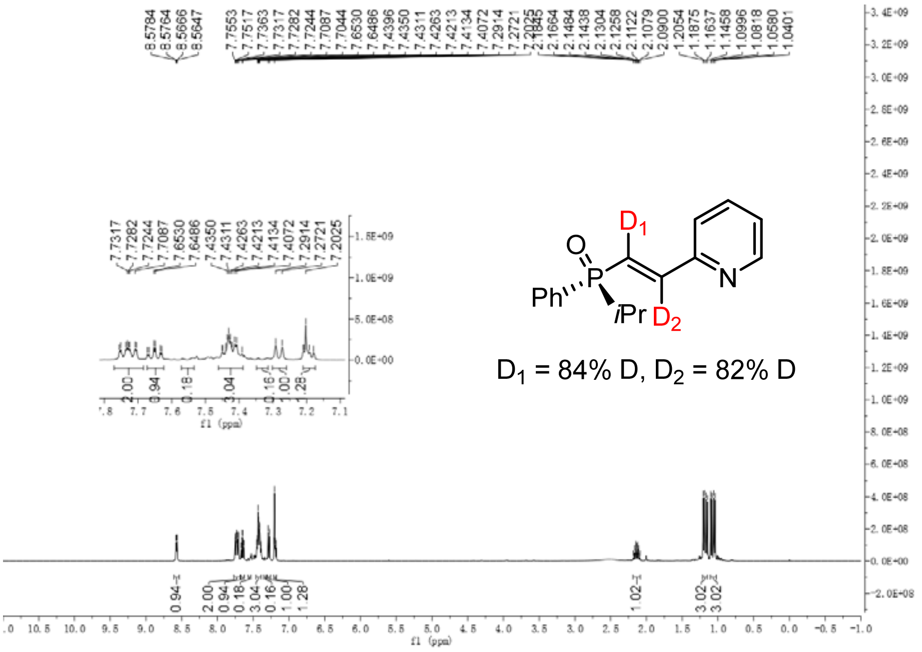

Fig. S2. ^{1}H NMR (600 MHz, $CDCl_3$) of D-**3**

II)

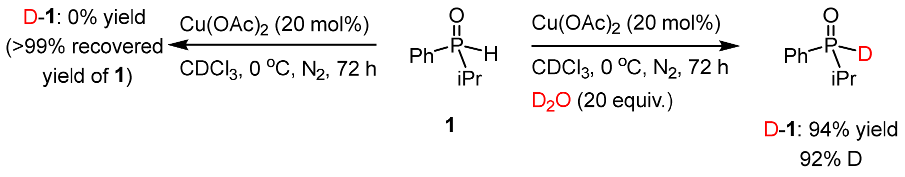

$Cu(OAc)_2$ (0.04 mmol, 0.2 equiv.), was added into a 10 mL Schlenk tube, and $CDCl_3$ (1 mL) was added, followed by the addition of D_2O (20 equiv.), and **1** (0.20 mmol), immediately degassed twice by freeze-pump-thaw method. The reaction mixture was stirred under a nitrogen atmosphere at 0 °C for 72 h. Reaction completed, D_2O was separated.

III)

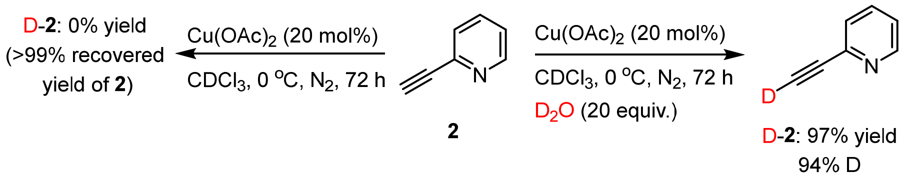

Cu(OAc)$_2$ (0.02 mmol, 0.2 equiv.), was added into a 10 mL Schlenk tube, and CDCl$_3$ (1 mL) was added, followed by the addition of D$_2$O (20 equiv.) and **2** (0.10 mmol), immediately degassed twice by freeze-pump-thaw method. The reaction mixture was stirred under a nitrogen atmosphere at 0 °C for 72 h. The solvent was removed under reduced pressure, and the residue was purified by flash chromatography on silica gel (PE/EA = 20/1) to give D-**2**.

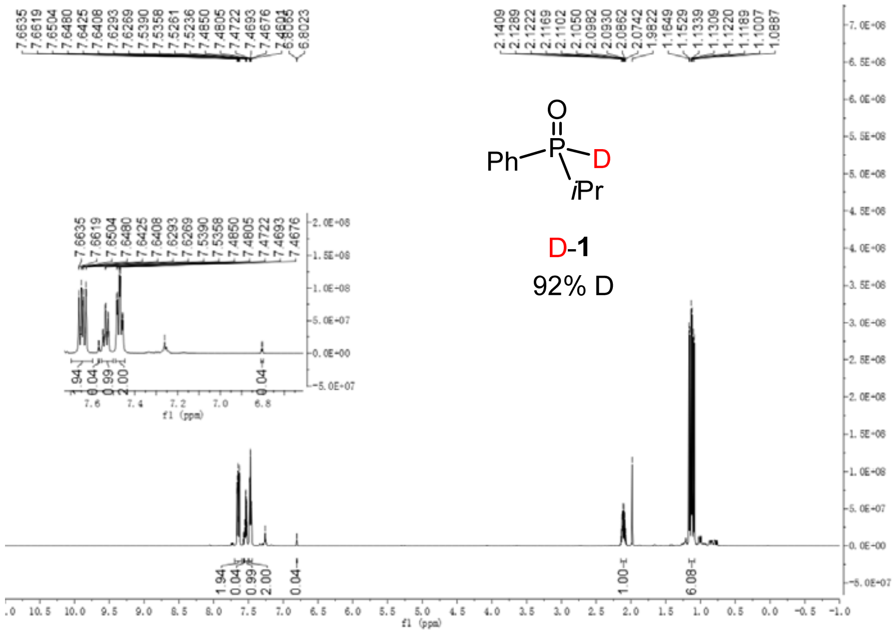

Fig. S3. ^{1}H NMR (600 MHz, CDCl$_3$) of D-**1**

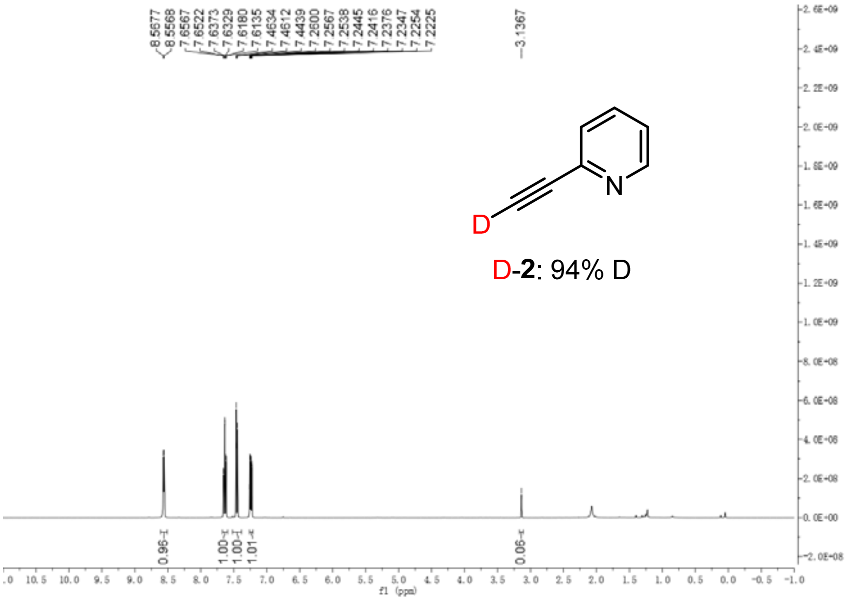

Fig. S4. ^{1}H NMR (400 MHz, $CDCl_3$) of D-**2**

F) X-band EPR spectrum of $Cu(OAc)_2$[4].

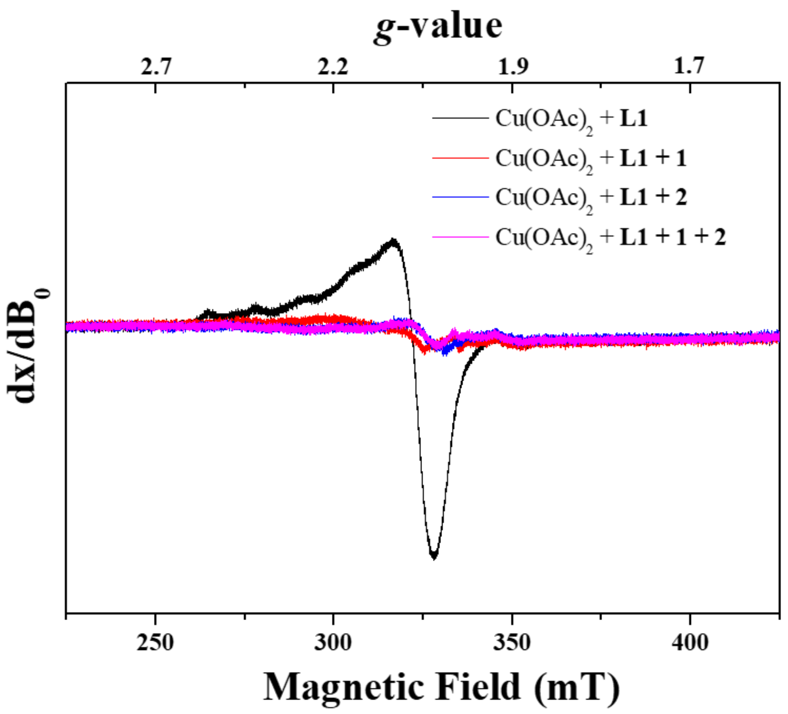

Fig. S5. X-band EPR spectrum of $Cu(OAc)_2$

1) In a nitrogen-filled glovebox, a mixture of $Cu(OAc)_2$ (0.1mmol), **L1** (0.11 mmol) in CH_3CN (1 mL) was stirred for 30 min. Then an aliquot (0.50 mL) was added into an EPR tube. The EPR tube containing the sample was then cooled with liquid nitrogen for an X-band EPR measurement at 90 K. Acquisition parameters: MW frequency = 9.388988 GHz; MW power = 20.00 mW; modulation amplitude = 0.1 mT; conversion time = 15.00 ms.

2) In a nitrogen-filled glovebox, a mixture of $Cu(OAc)_2$ (0.1mmol), **L1** (0.11 mmol) and **1** (0.2 mmol) in CH_3CN (1 mL) was stirred for 30 min. Then an aliquot (0.50 mL) was added into an EPR tube. The EPR tube containing the sample was then cooled with liquid nitrogen for an X-band EPR measurement at 90 K. Acquisition parameters: MW frequency = 9.388988 GHz; MW power = 20.00 mW; modulation amplitude = 0.1 mT; conversion time = 15.00 ms.

3) In a nitrogen-filled glovebox, a mixture of $Cu(OAc)_2$ (0.1mmol), **L1** (0.11 mmol) and **2** (0.2 mmol) in CH_3CN (1 mL) was stirred for 30 min. Then an aliquot (0.50 mL) was added into an EPR tube. The EPR tube containing the sample was then cooled with liquid nitrogen for an X-band EPR measurement at 90 K. Acquisition parameters: MW frequency = 9.388988 GHz; MW power = 20.00 mW; modulation amplitude = 0.1 mT; conversion time = 15.00 ms.

4) In a nitrogen-filled glovebox, a mixture of $Cu(OAc)_2$ (0.1mmol), **L1** (0.11 mmol), **1** and **2** (0.2 mmol) in CH_3CN (1 mL) was stirred for 30 min. Then an aliquot (0.50 mL) was added into an EPR tube. The EPR tube containing the sample was then cooled with liquid nitrogen for an X-band EPR measurement at 90 K. Acquisition parameters: MW frequency = 9.388988 GHz; MW power = 20.00 mW; modulation amplitude = 0.1 mT; conversion time = 15.00 ms.

6. DFT calculation

I. Computation Details

All the calculations in this work were performed on the basis of density functional theory (DFT) in the Gaussian G16 package (Revision B.01).[5] Geometry optimizations and frequencies were calculated with the B3LYP[6], dispersion-corrected with the D3 version of Grimme's dispersion with Becke-Johnson damping (B3LYP-D3(BJ))[7] and the basis set of SDD for Cu[8], 6-31G(d, p) for other atoms[9] Intrinsic reaction coordinate (IRC)[10] calculations were also carried out to inspect whether each of the transition structures actually connected the proposed reactant and product. The nature of the local minima was established with analytical frequencies calculations and the single point energies were subsequently obtained at the M06-2X functional and 6-31G(d, p) basis set with SMD (dichloroethane, Ɛ = 10.1) solvent model[11] and the temperature was set to 273.15 K in terms of the experiment temperature. Noncovalent interactions (NCI) analysis[12] was employed to gain more insights into the important noncovalent interactions that are present in the key transition states. The key 3D structures were prepared using the CYL view visualization program.[13]

II. Conformation search

Table S2. Conformers of the complexes **Cu-R-1** and **Cu-S-1**. All energies are given in kcal/mol. A lot of high energy conformations all were at least 9.0 kcal/mol higher in energy will not be discussed here. The **a-Cu-R-1** and **a-Cu-S-1** were called **Cu-R-1** and **Cu-S-1** in the main text.

	**ΔG≠**
**a-Cu-R-1**	4.1
**b-Cu-R-1**	6.4
**c-Cu-R-1**	6.5
**d-Cu-R-1**	7.3
**e-Cu-R-1**	7.9
**a-Cu-S-1**	2.5
**b-Cu-S-1**	5.6
**c-Cu-S-1**	6.1
**d-Cu-S-1**	6.3
**e-Cu-S-1**	8.4
**f-Cu-S-1**	8.8

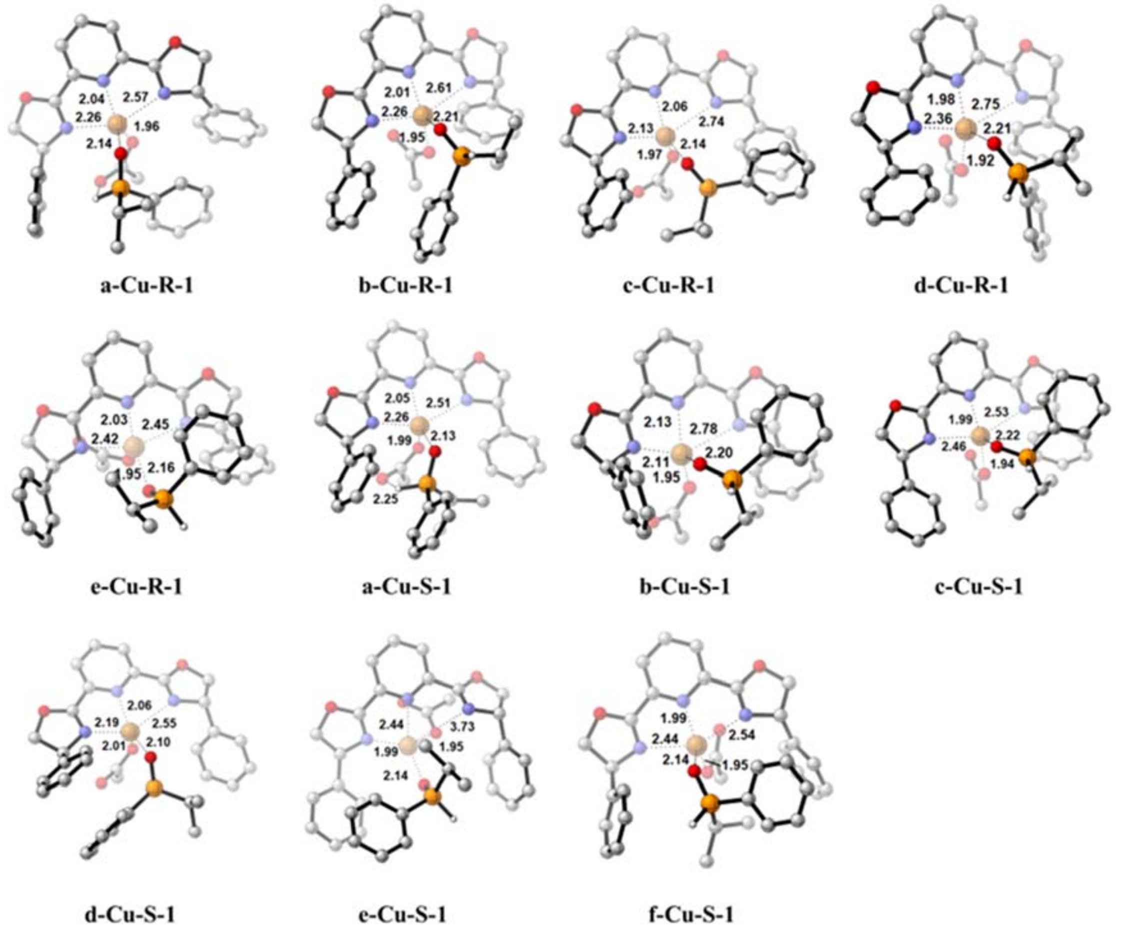

Figure S6. Different orientations of the substituents for the phosphinous acid moiety to compete with the low-lying complexes **Cu-R-1** and **Cu-S-1**. The bond distances are given in Angstroms. Hydrogen atoms are not involved in any noticeable interaction and are hidden for clarity.

III. The generation of copper(I) acetylides

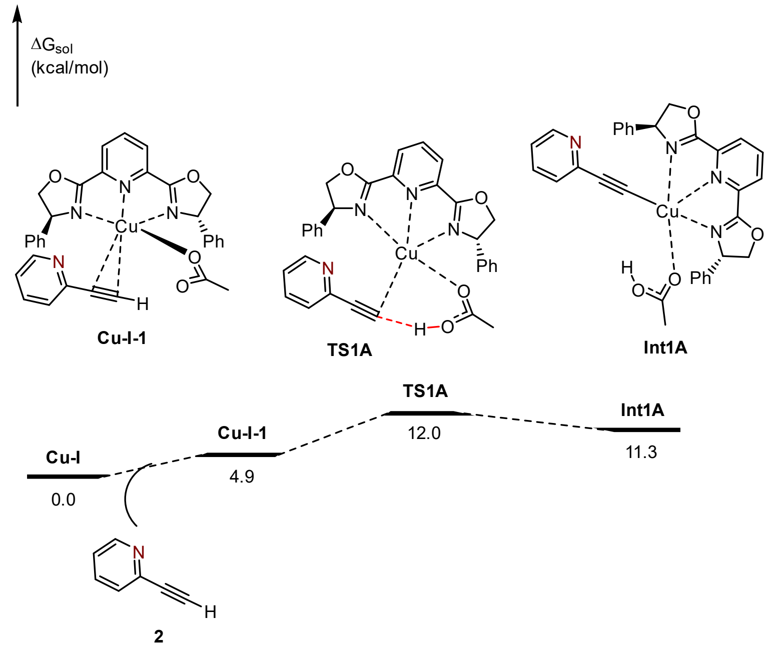

Figure S7. Free energy profiles of the deprotonation process of 2-ethynylpyridine.

IV. Tautomerization of double molecular pentavalent phosphorus oxide

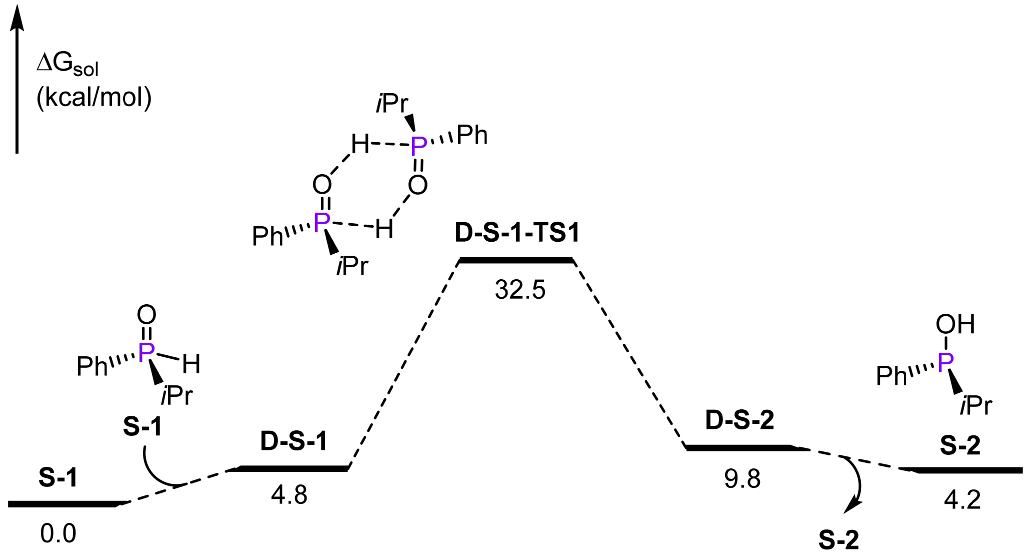

Figure S8. Free energy profiles of the tautomerization of double molecular pentavalent phosphorus oxide **S-1**.

V. The nucleophilic addition of copper(I) phosphinous oxide to the C-C triple bond of 2-ethynylpyridine

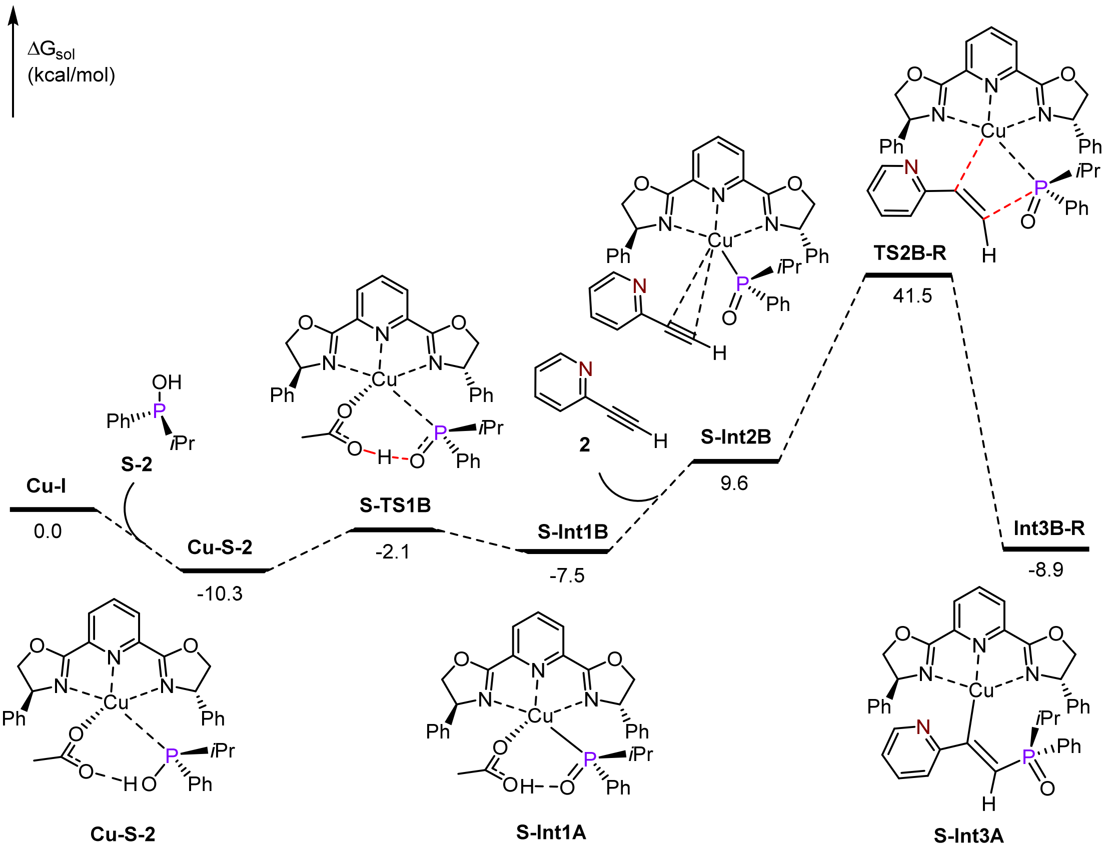

Figure S9. Free energy profiles of the nucleophilic addition of copper(I) phosphinous oxide to the C-C triple bond of 2-ethynylpyridine oxide.

VI. NCI analysis for the nucleophilic addition step

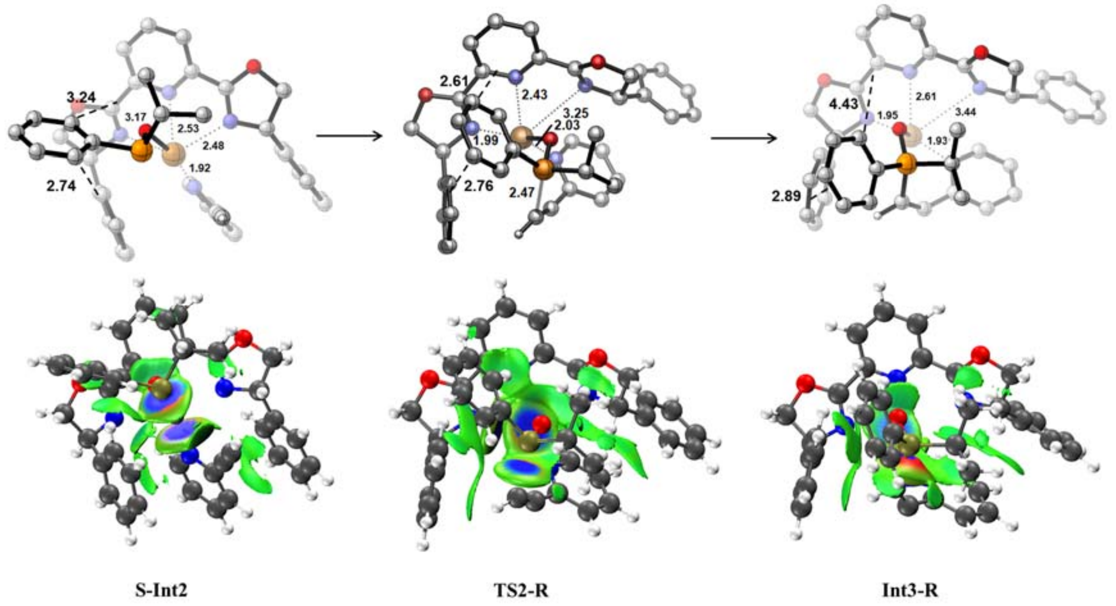

Figure S10. Non-covalent interaction (NCI) analysis for the **S-Int2**, **TS2-R** and **Int3-R** (the blue, green, and red regions represent strong, weak, and repulsive interactions).

VII. Distortion/interaction activation strain analysis

$$\Delta\Delta G^{\neq} = \Delta\Delta G^{\neq}_{dis} + \Delta\Delta G^{\neq}_{int}$$

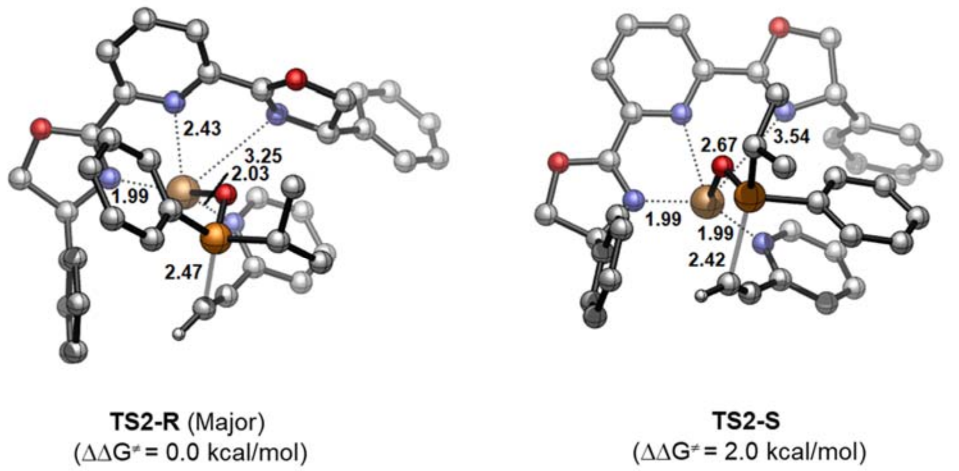

$\Delta\Delta G^{\neq}_{dis}$ = 0.0 kcal/mol	$\Delta\Delta G^{\neq}_{dis}$ = -1.55 kcal/mol
$\Delta\Delta G^{\neq}_{int}$ = 0.0 kcal/mol	$\Delta\Delta G^{\neq}_{int}$ = 3.54 kcal/mol

Figure S11. Distortion/interaction analyses of the transition states.

VIII. Cartesian coordination and energies for the all the calculated species were performed at the SMD/M062X/6-31G level in solvent phase.**

Cu-I

Sum of electronic and thermal Free Energies = -626.545964

C	-1.24968900	2.67602600	-0.25999800
C	1.00655900	2.74739400	0.26167300
C	1.00051100	4.14294500	0.26812700
C	-0.19058000	4.81528800	0.00456500
C	-1.33651700	4.06827800	-0.25842500
H	1.92087400	4.67151200	0.48272900
H	-0.22563600	5.89921700	0.00699900
H	-2.29113800	4.53622000	-0.46425000
N	-0.09678300	2.01062900	-0.00917800
C	2.22585300	1.98668600	0.56965300
C	3.64578000	0.37043200	1.08222000
C	4.41980400	1.70869900	0.89235700
H	3.61851200	0.09760300	2.14619400
H	5.04576900	1.99070300	1.73990900
H	5.01351000	1.70965500	-0.02668900
C	-2.41803300	1.83210500	-0.54374000
C	-3.73343200	0.09776300	-0.94814900
C	-4.58376900	1.40209200	-0.90215400
H	-3.66690500	-0.27256400	-1.98118400
H	-5.17706700	1.58459400	-1.79914700
H	-5.22452300	1.44401300	-0.01651800
N	-2.38595500	0.55741000	-0.57494800
N	2.27022100	0.71775300	0.69322000
O	3.36785100	2.70412000	0.75517400
O	-3.59472000	2.46413300	-0.79664000
C	4.23673800	-0.78736800	0.30780200
C	5.48589900	-1.29096900	0.69279300
C	3.57494000	-1.35377200	-0.78424500
C	6.07171400	-2.34303200	-0.00925700
H	5.99973200	-0.86499500	1.55204700
C	4.15924500	-2.41536300	-1.47886300
H	2.59221800	-1.00069600	-1.07244800
C	5.40708500	-2.90974900	-1.09928900
H	7.04029100	-2.72549700	0.29923200
H	3.62516000	-2.85428000	-2.31625700
H	5.85797200	-3.73501500	-1.64250000
C	-4.25380300	-1.02313500	-0.07066700
C	-5.61961600	-1.33332700	-0.09075000
C	-3.38972000	-1.77823300	0.72861000
C	-6.12093900	-2.37742600	0.68566000
H	-6.29943700	-0.76365800	-0.72062200
C	-3.89604900	-2.82347000	1.50341900
H	-2.32673000	-1.56572600	0.74428500
C	-5.25782000	-3.12556300	1.48834200

H	-7.18253400	-2.60582500	0.66243500
H	-3.21265900	-3.40075600	2.11897500
H	-5.64579600	-3.93910300	2.09428500
O	0.42548000	-1.58741400	-1.34032300
C	0.26045600	-2.35987400	-0.34020700
O	-0.11453600	-1.90907000	0.78355400
C	0.57702600	-3.83214600	-0.49550000
H	0.29767100	-4.18100800	-1.49218300
H	1.65926000	-3.96359400	-0.38460800
H	0.07275700	-4.42127400	0.27210300
Cu	-0.02779700	0.04896800	-0.11739400

S-1

Sum of electronic and thermal Free Energies = -767.008503

P	-1.14038600	-0.54090200	-0.80883200
O	-1.52583600	-1.99040900	-0.77419700
H	-1.25585600	0.11665100	-2.06641700
C	0.59602800	-0.21877300	-0.37739000
C	1.28719700	0.89737600	-0.86527800
C	1.24144500	-1.11141200	0.48786000
C	2.60563000	1.12950700	-0.47586100
H	0.80082400	1.58023100	-1.55642700
C	2.55973800	-0.87699700	0.87570300
H	0.70739500	-1.99148200	0.83188500
C	3.24001300	0.24454700	0.39790400
H	3.13956100	1.99393500	-0.85829500
H	3.05873400	-1.57134800	1.54458700
H	4.26763100	0.42448400	0.69858800
C	-2.15839200	0.53887300	0.28581600
H	-3.18404400	0.36432000	-0.06680700
C	-2.04633100	0.05328700	1.73634300
H	-1.02853300	0.19388000	2.11524600
H	-2.72631900	0.61925900	2.38092600
H	-2.29726900	-1.00751000	1.81411900
C	-1.81882500	2.02507800	0.13262900
H	-2.51239700	2.63208900	0.72314800
H	-0.80554500	2.23560300	0.48755400
H	-1.89072100	2.35776800	-0.90840600

R-1

Sum of electronic and thermal Free Energies = -767.008499

C	1.24095100	-1.11102400	-0.48845900
C	2.55924900	-0.87697700	-0.87650400
C	3.24012800	0.24401600	-0.39826700
C	2.60630200	1.12879500	0.47608100
C	1.28784800	0.89703800	0.86567900

C	0.59607500	-0.21852700	0.37736000
H	0.70649200	-1.99072000	-0.83281000
H	3.05778400	-1.57118700	-1.54588100
H	4.26776700	0.42363600	-0.69906500
H	3.14067500	1.99279300	0.85886700
H	0.80192000	1.57980000	1.55723200
P	-1.14031900	-0.54034000	0.80921100
O	-1.52581000	-1.98984600	0.77524600
H	-1.25559500	0.11774700	2.06652400
C	-2.15849600	0.53900300	-0.28571800
H	-3.18406300	0.36524900	0.06755300
C	-1.81825700	2.02518900	-0.13374500
H	-2.51204100	2.63202700	-0.72419600
H	-1.88936800	2.35859600	0.90711900
H	-0.80514000	2.23511700	-0.48945800
C	-2.04736100	0.05231400	-1.73594400
H	-1.02965200	0.19209800	-2.11540500
H	-2.29883500	-1.00841800	-1.81284400
H	-2.72731600	0.61816800	-2.38066300

Cu-S-1

Sum of electronic and thermal Free Energies = -2394.625825

P	1.46819100	-1.20952200	0.00960700
H	1.94935500	-0.40340200	1.05242700
C	0.59130500	-2.58444700	0.84824300
H	-0.31931100	-2.09617200	1.21027900
C	1.38304000	-3.11856000	2.04636200
H	0.78910300	-3.87133800	2.57571800
H	2.31604400	-3.59798200	1.73056600
H	1.61835500	-2.31456200	2.75049000
C	0.19762100	-3.66676600	-0.16190400
H	-0.43265500	-4.41709800	0.32342500
H	-0.37625500	-3.23690300	-0.98680100
H	1.08042200	-4.16809300	-0.57360800
C	2.96696500	-1.87743600	-0.76208500
C	4.11285000	-2.13736600	-0.00113700
C	2.98195000	-2.10234900	-2.14353600
C	5.26174500	-2.63309300	-0.61608300
H	4.11804900	-1.92485800	1.06367900
C	4.13386600	-2.59232500	-2.75721700
H	2.09597700	-1.86544200	-2.72404600
C	5.27270600	-2.86009400	-1.99368200
H	6.15138100	-2.82807500	-0.02474500
H	4.14678300	-2.76111400	-3.82999300
H	6.17033600	-3.23956600	-2.47318300

O	0.58751900	-0.49919700	-1.01431100
C	-3.14640200	2.27363800	-0.98689000
C	-1.37437000	3.59095300	-0.28358000
C	-2.11173700	4.76112900	-0.46243800
C	-3.42593600	4.65643900	-0.91967300
C	-3.95711800	3.39309300	-1.18250600
H	-1.65741600	5.71939300	-0.23968900
H	-4.02902400	5.54622200	-1.06532800
H	-4.97462500	3.26237300	-1.53075700
N	-1.87918700	2.36524700	-0.54112500
C	0.00006300	3.55468100	0.23013300
C	1.91412000	2.75187500	1.01501800
C	1.95388300	4.31093300	1.00745700
H	1.83069200	2.34750100	2.02962400
H	2.17920500	4.75520700	1.97779300
H	2.63741500	4.70426100	0.24879000
C	-3.59986100	0.89710200	-1.23321700
C	-3.64182100	-1.29991800	-1.50031900
C	-5.09368700	-0.73431400	-1.54650400
H	-3.33202800	-1.63291600	-2.49969800
H	-5.65447700	-1.01296900	-2.43984500
H	-5.66323900	-1.00005600	-0.65070800
N	-2.83299500	-0.12159400	-1.15632500
N	0.64166400	2.45692400	0.35006700
O	0.59636800	4.70433900	0.63786700
O	-4.90966000	0.70867700	-1.55453700
C	3.09300600	2.09978900	0.32847100
C	4.25123300	1.81196600	1.05887200
C	3.04936300	1.78394900	-1.03379500
C	5.35681000	1.23462000	0.43408500
H	4.27915000	2.02820000	2.12375300
C	4.15271800	1.19983700	-1.65618600
H	2.13210600	1.95259100	-1.58620200
C	5.31075400	0.92987100	-0.92736000
H	6.24800300	1.01037600	1.01310100
H	4.09785500	0.93151500	-2.70632300
H	6.15985400	0.45708300	-1.41020900
C	-3.46887200	-2.45337600	-0.53718000
C	-3.10742900	-2.23068700	0.79590300
C	-3.70635700	-3.76072500	-0.97619700
C	-2.98854500	-3.30948100	1.67331800
H	-2.86067800	-1.23325400	1.14301700
C	-3.59628900	-4.83607000	-0.09463900
H	-3.97108200	-3.93929600	-2.01596700

C	-3.23710000	-4.61169000	1.23591200
H	-2.69048000	-3.12427900	2.70121600
H	-3.78103600	-5.84627200	-0.44801600
H	-3.14368000	-5.44765600	1.92306200
O	-1.22755300	0.02031700	1.87436800
C	-0.39692600	0.00968300	2.85857500
O	0.79545400	0.36626000	2.82795800
C	-0.96513600	-0.57982700	4.15123800
H	-2.01824700	-0.31581500	4.27430200
H	-0.90084200	-1.67345100	4.09304400
H	-0.38467500	-0.25063100	5.01512800
Cu	-0.79569200	0.72744500	0.05612900

Cu-R-1

Sum of electronic and thermal Free Energies = -2394.623207

P	1.08069100	-1.74025600	-0.99463100
H	2.10446100	-1.80091000	-0.03963000
O	0.63412300	-0.35011000	-1.42694800
C	-1.05183400	3.60895000	-0.63353600
C	1.13091300	3.66037800	0.13963000
C	1.20128800	5.04484800	-0.00907400
C	0.08291100	5.72251100	-0.49347500
C	-1.06388700	4.99416800	-0.80930900
H	2.11596700	5.56281300	0.25450100
H	0.10337100	6.79945900	-0.62108100
H	-1.96073900	5.47459900	-1.18078500
N	0.02131000	2.94369500	-0.15942600
C	2.23487900	2.84263200	0.64982000
C	3.37328400	1.05930600	1.32573400
C	4.23507500	2.34533900	1.51658800
H	3.04014900	0.63601100	2.27732900
H	4.58278200	2.49681200	2.53945200
H	5.08422200	2.38441300	0.82748500
C	-2.22187900	2.77096200	-0.93046300
C	-3.57430300	1.05109800	-1.25983700
C	-4.38352900	2.38228300	-1.36223500
H	-3.50181400	0.57606100	-2.24782300
H	-4.91977000	2.50667500	-2.30476900
H	-5.07450200	2.50631000	-0.52347000
N	-2.22548500	1.49507400	-0.88310900
N	2.17826300	1.56697200	0.64308800
O	3.33238200	3.44292500	1.18226900
O	-3.37106700	3.41859200	-1.27463600
C	4.04416300	-0.03628000	0.53075600
C	4.41726400	-1.23104000	1.15119500

C	4.26347000	0.11917900	-0.84254300
C	5.02006900	-2.25170800	0.41412900
H	4.19027400	-1.37496200	2.20224900
C	4.86075600	-0.90092700	-1.58198200
H	3.93052800	1.02677300	-1.33638100
C	5.24551300	-2.08848600	-0.95382800
H	5.30088900	-3.17853700	0.90555400
H	5.02088900	-0.77240900	-2.64854700
H	5.70831300	-2.88432700	-1.52972200
C	-4.18609800	0.05861200	-0.29269100
C	-3.63114700	-0.17445100	0.96853300
C	-5.35705200	-0.61494100	-0.66576500
C	-4.25382200	-1.06331500	1.84705200
H	-2.68836700	0.27946800	1.25128600
C	-5.98019500	-1.49615800	0.21776700
H	-5.78286100	-0.45117100	-1.65364200
C	-5.42915300	-1.71909300	1.48240900
H	-3.79827900	-1.25166400	2.81429800
H	-6.88885900	-2.01038000	-0.08195300
H	-5.90809200	-2.40986800	2.17033800
O	-0.60923400	-0.00591600	1.78590300
C	0.12718400	-0.85739900	2.40733600
O	1.30730200	-1.15132600	2.13444900
C	-0.58650100	-1.57841900	3.55034600
H	-1.15626200	-0.86877800	4.15707800
H	-1.29623200	-2.29138300	3.11730600
H	0.12520400	-2.12256600	4.17405200
Cu	0.05268100	0.93245800	0.18844200
C	-0.26532400	-2.71803200	-0.28717500
C	-0.01971400	-3.72040400	0.65900500
C	-1.57387200	-2.46036400	-0.71201400
C	-1.07957900	-4.47920800	1.15426300
H	0.98630000	-3.87471700	1.03513800
C	-2.63144700	-3.21689700	-0.21146400
H	-1.75458000	-1.64115000	-1.39954800
C	-2.38283600	-4.22951100	0.71616500
H	-0.89206900	-5.25055800	1.89531000
H	-3.64727800	-2.99556300	-0.51953900
H	-3.20919000	-4.81063500	1.11495800
C	1.72915000	-2.69384200	-2.42936100
H	2.57025300	-2.08270300	-2.77995200
C	0.67178700	-2.77966900	-3.53580800
H	1.08749700	-3.26795700	-4.42358400
H	-0.19204800	-3.36476900	-3.20312000

H	0.32080700	-1.78385200	-3.81867800
C	2.25521000	-4.06839000	-2.00089900
H	2.70626500	-4.58302200	-2.85605400
H	3.01818500	-3.98067800	-1.22156600
H	1.44535600	-4.69818800	-1.61961800

S-TS1

Sum of electronic and thermal Free Energies = -2394.612364

P	1.37710600	-1.31869700	0.15663100
H	1.43260700	-0.43222100	1.61404000
C	0.33597200	-2.82148900	0.50524300
H	-0.56301000	-2.37122300	0.93800300
C	0.97117400	-3.73603000	1.55527200
H	0.26773200	-4.52439600	1.84672900
H	1.87469600	-4.21932500	1.16807100
H	1.24502300	-3.18177700	2.46032300
C	-0.05851900	-3.56307200	-0.77220300
H	-0.81247100	-4.32793900	-0.55607300
H	-0.47429000	-2.86496000	-1.50389000
H	0.81053800	-4.05257400	-1.22632600
C	2.90061700	-2.01914800	-0.57857100
C	3.94669600	-2.47848300	0.23095900
C	3.04236600	-2.06429900	-1.97117900
C	5.11173500	-2.98991600	-0.34137000
H	3.85743800	-2.41693200	1.31226500
C	4.20817900	-2.57090800	-2.54444600
H	2.23576600	-1.67690300	-2.58533000
C	5.24483100	-3.03584800	-1.73088400
H	5.91918700	-3.34235400	0.29446700
H	4.31209700	-2.60184500	-3.62582400
H	6.15400500	-3.42851900	-2.17755100
O	0.65915800	-0.53272100	-1.00512100
C	-3.08860000	2.21850600	-0.97109700
C	-1.31250200	3.56107800	-0.32905300
C	-2.07431400	4.71837300	-0.49557200
C	-3.39719500	4.59757000	-0.91833600
C	-3.91898100	3.32464000	-1.14786700
H	-1.62614700	5.68214100	-0.28563700
H	-4.01518200	5.47866900	-1.05299400
H	-4.94576300	3.17363700	-1.45832800
N	-1.79370700	2.32618800	-0.59647900
C	0.06479800	3.58509600	0.17992100
C	2.00554200	2.93606700	1.04560300
C	2.06194900	4.45932000	0.69634200
H	2.08441900	2.77015600	2.12401100

H	2.24635400	5.10047600	1.56104100
H	2.78530600	4.68187200	-0.09119400
C	-3.55033100	0.83872000	-1.16350800
C	-3.59586100	-1.37348200	-1.26813700
C	-5.02414700	-0.80157000	-1.51404100
H	-3.17666200	-1.76992600	-2.20247800
H	-5.49236000	-1.14473700	-2.43714300
H	-5.68958500	-0.98680300	-0.66528200
N	-2.82125700	-0.17875700	-0.90954900
N	0.64818700	2.54457300	0.63468200
O	0.73218500	4.76847200	0.18264500
O	-4.81444300	0.63710200	-1.61993900
C	3.06680100	2.10454200	0.35980600
C	4.12486300	1.56938500	1.09848200
C	3.00860900	1.87401700	-1.01869700
C	5.12605800	0.83170100	0.46578900
H	4.15245200	1.71640300	2.17454400
C	4.00754300	1.13666500	-1.64953900
H	2.16007300	2.23707400	-1.58940900
C	5.07204300	0.61913800	-0.91079300
H	5.93629600	0.40699100	1.05053500
H	3.93933700	0.93757700	-2.71393700
H	5.82812500	0.01382600	-1.39905000
C	-3.53715800	-2.45914200	-0.21746200
C	-3.13267800	-2.17666900	1.09068400
C	-3.90005500	-3.76665900	-0.56004100
C	-3.08852800	-3.19884500	2.04063300
H	-2.79871000	-1.17873900	1.35230600
C	-3.86350800	-4.78353700	0.39278400
H	-4.19592000	-3.99383600	-1.58173100
C	-3.45372300	-4.50082900	1.69787300
H	-2.74879300	-2.97419100	3.04723400
H	-4.14089200	-5.79591500	0.11447800
H	-3.41200300	-5.29354500	2.43890300
O	-0.91727600	0.10208500	1.87931300
C	-0.05684400	0.48138900	2.70272500
O	1.20710900	0.22834400	2.63068400
C	-0.47694900	1.35207600	3.87188700
H	0.20598300	1.24455400	4.71647600
H	-0.45054600	2.39492300	3.53447600
H	-1.50149800	1.12048200	4.16907700
Cu	-0.68434200	0.67999700	-0.29457700

R-TS1

Sum of electronic and thermal Free Energies = -2394.610016

P	0.32117600	-2.09314800	-0.01901200
H	0.79408100	-1.55444900	1.52054400
O	0.39699400	-0.95402500	-1.10275800
C	-0.27350900	3.65419300	-0.68446000
C	1.89212500	3.23091400	0.02120200
C	2.19020300	4.59384500	0.05666000
C	1.20138800	5.50816300	-0.30308700
C	-0.05670400	5.03158100	-0.67062100
H	3.17847900	4.91194300	0.36612900
H	1.40301900	6.57369000	-0.28425800
H	-0.86593700	5.69912800	-0.94072900
N	0.68937700	2.75732800	-0.37499100
C	2.85817900	2.20151000	0.42708700
C	3.75099200	0.24833500	0.98228500
C	4.85070700	1.36218000	1.01456100
H	3.62001800	-0.21284600	1.96561500
H	5.23709400	1.55537000	2.01899700
H	5.67745600	1.16914300	0.32906800
C	-1.57381000	3.05847500	-1.01494900
C	-3.20310000	1.58848100	-1.32609300
C	-3.74700000	3.03567800	-1.53905800
H	-3.15928600	1.05230700	-2.28414100
H	-4.21227700	3.20075900	-2.51180800
H	-4.43245900	3.33684800	-0.74186700
N	-1.82076500	1.81216900	-0.88514500
N	2.52714000	0.99183700	0.66540700
O	4.16164600	2.56311600	0.56127900
O	-2.56063400	3.87769600	-1.46074200
C	4.03332700	-0.84476200	-0.03067100
C	4.77109700	-1.96829000	0.35810200
C	3.61396500	-0.72971900	-1.35978100
C	5.10857500	-2.95198100	-0.57172700
H	5.07715700	-2.07556500	1.39581600
C	3.95013200	-1.71323400	-2.28957300
H	2.98762000	0.10356100	-1.65393800
C	4.70327800	-2.82227400	-1.90167000
H	5.67768800	-3.82138100	-0.25637600
H	3.60293500	-1.62187100	-3.31391900
H	4.95599400	-3.59082300	-2.62582400
C	-4.02079000	0.76769100	-0.35095700
C	-3.47777600	0.30582900	0.85124800
C	-5.35132800	0.46930400	-0.66841400
C	-4.26744400	-0.44303000	1.72442400
H	-2.44255700	0.51077100	1.09707600

C	-6.13879000	-0.27198000	0.21119200
H	-5.77385800	0.81217500	-1.61087900
C	-5.59575600	-0.73011700	1.41275400
H	-3.82986400	-0.81637300	2.64481200
H	-7.17019800	-0.49715100	-0.04452500
H	-6.20262600	-1.31804500	2.09499400
O	-0.34686000	0.56865900	1.86072000
C	0.45163100	0.10663200	2.70376700
O	1.09424100	-1.00611800	2.59333800
C	0.75425300	0.91118300	3.95491200
H	1.05594200	0.26498000	4.78120200
H	1.58584900	1.58792600	3.72513800
H	-0.10856200	1.51944100	4.23230000
Cu	0.23531800	0.80364800	-0.27693700
C	-1.42149600	-2.66607100	0.00230800
C	-1.93013600	-3.41777400	1.06978200
C	-2.27412000	-2.31065600	-1.05070700
C	-3.26202200	-3.83218400	1.06946800
H	-1.28518100	-3.67016800	1.90776600
C	-3.60648800	-2.72016900	-1.04870900
H	-1.87443000	-1.69564900	-1.85107800
C	-4.10101700	-3.48445700	0.00883300
H	-3.64966300	-4.41333800	1.90163500
H	-4.26679500	-2.42668100	-1.85926500
H	-5.14300000	-3.78968400	0.01743900
C	1.25077500	-3.54558400	-0.71704300
H	2.29334400	-3.21331600	-0.68219800
C	0.87135600	-3.80817700	-2.17573200
H	-0.16526000	-4.15389400	-2.25358900
H	0.96954300	-2.89438900	-2.76683500
H	1.51948800	-4.57828800	-2.61070800
C	1.10747200	-4.78870900	0.16634600
H	1.76647400	-5.58783400	-0.19151800
H	1.37426500	-4.57841800	1.20814300
H	0.08127700	-5.16935500	0.15147400

S-Int1

Sum of electronic and thermal Free Energies = -2394.615556

P	1.04463300	-1.67982700	0.14264900
H	1.29429800	-0.51462300	1.84028100
C	-0.33522900	-2.93046200	0.37701000
H	-1.11727500	-2.32354800	0.84654100
C	0.05982900	-4.04356000	1.34964700
H	-0.80755000	-4.66872800	1.59166500
H	0.83087100	-4.69071500	0.91731800

H	0.45147800	-3.63819200	2.28991600
C	-0.87458100	-3.46830300	-0.94834800
H	-1.79329700	-4.04438700	-0.78857100
H	-1.09269300	-2.64189100	-1.63104000
H	-0.14042000	-4.12029200	-1.43447200
C	2.31962100	-2.70321400	-0.70731200
C	3.28315400	-3.40595200	0.02686000
C	2.38441200	-2.72358000	-2.10753900
C	4.28572500	-4.12721100	-0.62269600
H	3.26163500	-3.36827900	1.11321500
C	3.38863000	-3.44000000	-2.75907300
H	1.65159600	-2.14829700	-2.66468100
C	4.34213600	-4.14360500	-2.01796000
H	5.03043200	-4.66491200	-0.04209200
H	3.43271200	-3.44772200	-3.84516300
H	5.12843800	-4.69584400	-2.52498400
O	0.51934000	-0.72500900	-1.02781700
C	-2.63826800	2.61311100	-1.08956100
C	-0.64890800	3.67448200	-0.55747600
C	-1.20395500	4.92857900	-0.82130400
C	-2.53192300	5.00555600	-1.23525200
C	-3.26689800	3.82777100	-1.36019700
H	-0.59223900	5.81322200	-0.69426900
H	-2.98933900	5.96634800	-1.44499100
H	-4.30775800	3.82977800	-1.65994000
N	-1.33849900	2.52410300	-0.72139300
C	0.72618300	3.53172200	-0.05815300
C	2.56124600	2.76418900	0.91579700
C	2.83813700	4.16948100	0.30256200
H	2.53753100	2.82118400	2.01177600
H	3.23468900	4.89885500	1.01045400
H	3.48879600	4.10878700	-0.57435500
C	-3.34389800	1.32865600	-1.17165500
C	-3.79502700	-0.83855500	-1.08648600
C	-5.08801700	-0.04160500	-1.43718800
H	-3.43651600	-1.39606200	-1.96144900
H	-5.58339500	-0.37131200	-2.35099800
H	-5.80102200	-0.03248100	-0.60733300
N	-2.81849300	0.22255700	-0.81060500
N	1.18840800	2.46620400	0.47349500
O	1.52830900	4.62806900	-0.13795400
O	-4.61748100	1.32258500	-1.64514200
C	3.59190400	1.73031200	0.52411000
C	4.80941300	1.70788200	1.21483200

C	3.38105400	0.83609400	-0.52926800
C	5.81231500	0.81004800	0.85282400
H	4.96837800	2.38930300	2.04786700
C	4.38650900	-0.06492800	-0.88759300
H	2.42024900	0.79885600	-1.03151100
C	5.60124400	-0.07863000	-0.20298800
H	6.75015100	0.79736400	1.40072200
H	4.20545500	-0.77484000	-1.68620700
H	6.37184900	-0.79136900	-0.48068100
C	-3.95983700	-1.80636200	0.06334500
C	-3.51849700	-1.48129900	1.34952100
C	-4.56566200	-3.04693000	-0.16443700
C	-3.68097500	-2.39455100	2.39257800
H	-3.00297800	-0.54314000	1.52077500
C	-4.73335700	-3.95434800	0.88048700
H	-4.89051700	-3.31192500	-1.16793800
C	-4.28791500	-3.62958000	2.16360600
H	-3.31477000	-2.14279500	3.38339100
H	-5.19772300	-4.91748800	0.69102700
H	-4.40590200	-4.33975700	2.97652800
O	-0.86915000	0.44833600	1.89605800
C	0.06035200	0.72139300	2.65774900
O	1.25076700	0.16113900	2.61903600
C	-0.06500400	1.78970000	3.71712100
H	-1.10130500	1.86361000	4.04975700
H	0.60201800	1.60074100	4.55963100
H	0.21500800	2.74430100	3.25774800
Cu	-0.53269100	0.73396000	-0.39389500

2

Sum of electronic and thermal Free Energies = -324.235438

C	-1.40571800	-1.20479500	0.00000000
C	0.55671900	-0.02131200	-0.00006100
C	-0.13213300	1.20351900	-0.00000900
C	-1.52250500	1.18712600	0.00001800
C	-2.17900600	-0.04218100	0.00000600
H	-1.88575800	-2.18134400	-0.00001500
H	0.42533200	2.13314600	-0.00000300
H	-2.08282200	2.11689500	0.00001900
H	-3.26199100	-0.10406100	0.00000800
N	-0.07085400	-1.21478700	0.00001600
C	1.99023200	-0.03089500	-0.00000600
C	3.19862700	-0.00221800	0.00003800
H	4.26391100	0.00341300	-0.00004100

HOAc

Sum of electronic and thermal Free Energies = -228.960513

C	-0.09196600	0.12530200	-0.00021700
O	-0.64274500	1.20273500	0.00002700
C	1.39582600	-0.11132000	0.00006800
H	1.91525300	0.84561100	0.00005500
H	1.67966500	-0.69354100	0.88136200
H	1.67995900	-0.69373400	-0.88100300
O	-0.77947500	-1.04540500	0.00001900
H	-1.72027400	-0.80087300	0.00012100

R-Int1

Sum of electronic and thermal Free Energies = -2394.614483

P	0.21851100	-2.15287400	0.08336900
H	0.79808000	-1.38854400	1.91123300
O	0.43033300	-1.03128900	-1.03316000
C	-0.17857100	3.56536200	-0.77763600
C	1.99717600	3.15146000	-0.09369900
C	2.31403500	4.50973500	-0.14304700
C	1.33010100	5.41614200	-0.53339700
C	0.05838100	4.93761000	-0.84729800
H	3.31355500	4.83005500	0.12515200
H	1.54627000	6.47795700	-0.58016200
H	-0.74711000	5.59997300	-1.14013000
N	0.78025600	2.67391700	-0.43954800
C	2.96273100	2.13520400	0.34374100
C	3.85738300	0.20925100	0.98560700
C	4.97437500	1.29822500	0.86324400
H	3.79996700	-0.18338500	2.00514300
H	5.42112600	1.56599400	1.82495000
H	5.75652100	1.03191000	0.15099500
C	-1.49455900	2.97551500	-1.05302700
C	-3.16133400	1.52785400	-1.24128800
C	-3.65877600	2.95796300	-1.61939700
H	-3.12267000	0.88564400	-2.13194100
H	-4.08238300	3.03379400	-2.62193800
H	-4.36522200	3.35539500	-0.88550000
N	-1.77522100	1.75415000	-0.81010600
N	2.62336600	0.95909500	0.71157100
O	4.27941900	2.47074700	0.35025800
O	-2.45463600	3.77727800	-1.58156700
C	4.04717600	-0.95414400	0.03112000
C	4.74675100	-2.08686300	0.46075000
C	3.58751900	-0.89429600	-1.28790800
C	5.00692500	-3.13630600	-0.42065700
H	5.08562800	-2.14891700	1.49205500

C	3.84638600	-1.94312100	-2.16904500
H	2.98777100	-0.05267900	-1.61165600
C	4.56182700	-3.06242900	-1.74183100
H	5.54685400	-4.01231700	-0.07391700
H	3.46508600	-1.89494700	-3.18391300
H	4.75270700	-3.88211400	-2.42780300
C	-4.00915300	0.83667200	-0.19379500
C	-3.44361100	0.31548200	0.97269800
C	-5.38277900	0.69113500	-0.42013600
C	-4.25187400	-0.34291900	1.89947200
H	-2.37928100	0.41182500	1.14723100
C	-6.18800300	0.03848000	0.51201300
H	-5.82714500	1.07933300	-1.33444300
C	-5.62150500	-0.48146000	1.67662600
H	-3.80016400	-0.76108400	2.79348100
H	-7.25276400	-0.06878400	0.32596000
H	-6.24366400	-0.99961600	2.40042100
O	-0.26026100	0.73140500	1.95387200
C	0.55798500	0.32094900	2.77635500
O	1.11546100	-0.87329500	2.74989200
C	1.05469100	1.18588400	3.91007500
H	1.96652600	1.68917800	3.56934800
H	0.31062100	1.94558100	4.15166800
H	1.30157700	0.58878000	4.78974500
Cu	0.33928800	0.73379400	-0.31883500
C	-1.57107000	-2.58875600	-0.06369000
C	-2.22937300	-3.32051600	0.93366500
C	-2.30822600	-2.12876300	-1.16266400
C	-3.59101100	-3.60360200	0.82593300
H	-1.67500900	-3.65937100	1.80594800
C	-3.67107300	-2.40458500	-1.26839700
H	-1.79062400	-1.53856400	-1.91295400
C	-4.31565900	-3.14307500	-0.27520200
H	-4.09186800	-4.16809000	1.60789600
H	-4.23748900	-2.03250900	-2.11798700
H	-5.38081400	-3.34086300	-0.34769100
C	1.03022800	-3.67233500	-0.65846700
H	2.09874200	-3.44338300	-0.57553200
C	0.68431600	-3.84537700	-2.13766800
H	-0.37208800	-4.10690500	-2.26251500
H	0.86857700	-2.91606200	-2.68230300
H	1.28677400	-4.64366300	-2.58887000
C	0.73748300	-4.92833600	0.16661800
H	1.33071000	-5.77578300	-0.19659800

H	0.97844000	-4.78221300	1.22594800
H	-0.31892800	-5.20769000	0.09857100

S-Int2

Sum of electronic and thermal Free Energies = -2489.884562

C	-1.33569300	-0.73061400	2.69382100
C	0.20000300	-2.29779900	2.02582000
C	-0.57477800	-3.35319800	2.51627600
C	-1.79371900	-3.04501000	3.11813400
C	-2.20167900	-1.71440700	3.18292100
H	-0.24215200	-4.37630100	2.38826200
H	-2.43420100	-3.83491500	3.49634400
H	-3.16662500	-1.43331700	3.58719800
N	-0.14602600	-1.01093500	2.15463900
C	1.38269100	-2.52702400	1.17595000
C	2.90776500	-2.27737700	-0.41765300
C	3.07564000	-3.67482000	0.25704900
H	2.55303200	-2.38356100	-1.44921400
H	3.00084000	-4.51081400	-0.44017100
H	4.00273200	-3.75276300	0.83219100
C	-1.72819000	0.69286000	2.62113400
C	-1.75189000	2.86390900	2.16767800
C	-3.21317700	2.34924500	2.30272500
H	-1.54862800	3.66349700	2.88751600
H	-3.74317900	2.79038000	3.15087900
H	-3.80571900	2.46810000	1.39400900
N	-0.92803700	1.68028000	2.51309300
N	1.83114600	-1.65388400	0.35874700
O	1.95921900	-3.75952400	1.18642000
O	-3.06908900	0.92621500	2.55215500
C	4.15443300	-1.42000300	-0.42345600
C	4.89497400	-1.24949600	-1.59575200
C	4.57281300	-0.77650000	0.74694900
C	6.04564900	-0.45969100	-1.59898700
H	4.55836700	-1.72354000	-2.51344400
C	5.72003800	0.01389000	0.74594000
H	3.97732200	-0.87572900	1.64892300
C	6.46181300	0.17224800	-0.42677300
H	6.61141800	-0.33352300	-2.51738100
H	6.02854400	0.51693800	1.65741900
H	7.35414700	0.79123800	-0.42841600
C	-1.33530600	3.32551200	0.78364700
C	-0.45980500	4.40508300	0.63858000
C	-1.69970000	2.58709700	-0.34842700
C	0.05209100	4.74008000	-0.61591600

H	-0.16345500	4.97540800	1.51570800
C	-1.19377300	2.92140800	-1.60400300
H	-2.34440800	1.72136200	-0.25597700
C	-0.31126200	3.99430800	-1.73900700
H	0.73655200	5.57767400	-0.71445500
H	-1.46538800	2.31460500	-2.46169600
H	0.09383400	4.24646800	-2.71457500
Cu	0.21618400	0.18673100	-0.04698700
P	-1.91507500	-0.86413200	-1.88245300
O	-1.40568900	-0.70411500	-0.36165900
C	-2.00303900	-2.75135500	-2.04860400
H	-2.54687800	-2.94996500	-2.98313900
C	-0.56744500	-3.27806400	-2.17997100
H	-0.55045800	-4.36550000	-2.32423500
H	-0.04223000	-2.81315200	-3.02232200
H	-0.00338100	-3.04792800	-1.26991400
C	-2.72417000	-3.41492100	-0.87333400
H	-3.77889300	-3.12966800	-0.83966800
H	-2.66587100	-4.50945900	-0.93643500
H	-2.26322100	-3.09905400	0.06711800
C	-3.73079600	-0.52853400	-1.68979900
C	-4.58125500	-0.49966300	-2.80385000
C	-4.26586900	-0.22937000	-0.42852100
C	-5.93457600	-0.19185300	-2.66184500
H	-4.17942200	-0.71305700	-3.79280000
C	-5.61655700	0.09175600	-0.28537300
H	-3.60749700	-0.25711800	0.43260500
C	-6.45640900	0.11043400	-1.40098500
H	-6.58250700	-0.17804800	-3.53452300
H	-6.01493600	0.32085200	0.70039700
H	-7.50788500	0.36146700	-1.29203000
H	1.26003600	-0.34445800	-4.04303800
C	2.02414600	2.10854900	1.10941900
C	2.47900500	1.53322200	-1.09845900
C	3.64180000	2.30876600	-1.09923400
C	3.98923500	3.00111000	0.05616700
C	3.16588300	2.90285500	1.17619200
H	1.33458900	1.99560100	1.94016800
H	4.25590000	2.34256100	-1.99028500
H	4.89141100	3.60382300	0.08101200
H	3.39841100	3.43054900	2.09469600
N	1.68681900	1.43325000	0.00003800
C	2.05528100	0.80624900	-2.25104100
C	1.66812100	0.19387500	-3.21945700

R-Int2

Sum of electronic and thermal Free Energies = -2489.888381

C	1.89436200	-0.18309700	2.67270900
C	0.02368400	1.14404400	2.67469000
C	0.64822800	2.11897900	3.46096100
C	1.96770200	1.90163700	3.85193000
C	2.61396100	0.73860300	3.44106100
H	0.11407000	3.02286300	3.72703500
H	2.49188200	2.63996600	4.44980300
H	3.65008000	0.54311100	3.68856500
N	0.62325600	0.00539700	2.30651500
C	-1.31509600	1.36836600	2.09588500
C	-3.06600700	1.34263400	0.74801600
C	-3.29862100	2.38311300	1.87658700
H	-2.85772200	1.84886600	-0.20121100
H	-3.55839400	3.37915700	1.51583100
H	-4.04263800	2.04567800	2.60604000
C	2.53035600	-1.40680400	2.14135800
C	2.92892400	-3.31093600	1.07618900
C	4.27796600	-2.63260600	1.44738300
H	2.85987000	-4.31223000	1.51347700
H	4.85519700	-3.20505200	2.17897000
H	4.90170700	-2.40671800	0.58110200
N	1.91299800	-2.43662500	1.70681500
N	-1.82169100	0.67376300	1.15505700
O	-2.00945000	2.46018400	2.54274600
O	3.88884400	-1.37769900	2.06127100
C	-4.19638700	0.36398400	0.53856700
C	-5.06078400	0.50301300	-0.55079500
C	-4.38082200	-0.70451100	1.42289500
C	-6.10081900	-0.40588900	-0.75136500
H	-4.90295000	1.31457400	-1.25546200
C	-5.41602600	-1.61558300	1.22321700
H	-3.68770800	-0.83359400	2.24818100
C	-6.28036800	-1.46826500	0.13583900
H	-6.76470100	-0.28828300	-1.60287500
H	-5.54342600	-2.44660900	1.91072800
H	-7.08600300	-2.17945100	-0.02120000
C	2.62775300	-3.39694300	-0.40712000
C	2.76764200	-2.26117900	-1.21498900
C	2.09893000	-4.56612500	-0.95890700
C	2.38126200	-2.29574900	-2.55421600
H	3.12723300	-1.32780900	-0.79404600
C	1.71207200	-4.60423500	-2.30007200

H	1.97914900	-5.44770300	-0.33354100
C	1.84992100	-3.46715100	-3.09824900
H	2.47121100	-1.39260900	-3.14956700
H	1.30142200	-5.51865400	-2.71849900
H	1.54179200	-3.49300200	-4.13940200
Cu	0.48188800	-0.46547900	-0.23609700
P	2.25863200	1.46908000	-1.60991000
O	1.93527800	0.70817000	-0.21867600
C	0.91199200	2.75602400	-1.68408900
C	0.58936000	3.39903500	-2.88693500
C	0.13896900	3.03833200	-0.54957400
C	-0.47746700	4.30009900	-2.95534000
H	1.16720800	3.17834700	-3.78170500
C	-0.92657600	3.93587500	-0.60948500
H	0.39214600	2.53377100	0.37456200
C	-1.24382500	4.56598300	-1.81652700
H	-0.71379300	4.79195300	-3.89542600
H	-1.49894600	4.15195400	0.28890600
H	-2.07620900	5.26223000	-1.86954300
C	3.68460200	2.58979000	-1.05905700
H	4.51360900	1.87708100	-0.94064800
C	4.05566400	3.59100800	-2.15593100
H	3.25645500	4.32690700	-2.29685200
H	4.96866500	4.13992400	-1.89600200
H	4.22535400	3.09374500	-3.11772300
C	3.43580200	3.26510900	0.29025500
H	4.34483200	3.75921500	0.65664400
H	2.65332600	4.02680300	0.20540300
H	3.11297900	2.53010100	1.03159800
H	-1.62590800	1.85481000	-3.05553400
C	-0.99396300	-2.86481800	0.19594100
C	-1.94165600	-1.42176600	-1.36640900
C	-3.04859000	-2.26210700	-1.51981000
C	-3.11280800	-3.43645400	-0.77826900
C	-2.06871700	-3.74356800	0.09214100
H	-0.15919800	-3.03311800	0.86854800
H	-3.84926300	-1.96552600	-2.18481300
H	-3.97202300	-4.09258300	-0.87061200
H	-2.08195800	-4.64564700	0.69376800
N	-0.92654200	-1.72943400	-0.51743400
C	-1.84457500	-0.18032800	-2.06144600
C	-1.76522800	0.89136600	-2.61678100

TS2-R

Sum of electronic and thermal Free Energies = -2489.864039

C	-1.24960400	-2.16728100	2.13336800
C	0.63534200	-2.71693400	0.93207400
C	0.21861500	-4.04708800	0.80708700
C	-0.96439000	-4.43610600	1.43706700
C	-1.72676800	-3.48225900	2.10920400
H	0.81006900	-4.74396800	0.22553400
H	-1.30559900	-5.46418200	1.37384300
H	-2.68168800	-3.72412400	2.56072200
N	-0.07208600	-1.80355700	1.60361000
C	1.83216000	-2.19145900	0.24442900
C	3.38679200	-0.84045400	-0.51397800
C	3.33562800	-2.11090900	-1.40611600
H	2.90518500	0.00108600	-1.02881300
H	3.19381800	-1.90266800	-2.46679200
H	4.21016500	-2.75426800	-1.26058500
C	-2.06577300	-1.02520400	2.55212400
C	-2.88036600	1.05982200	2.63512800
C	-3.88484200	0.06104400	3.28590500
H	-2.47559300	1.75251500	3.38003400
H	-4.10767400	0.28501500	4.33039300
H	-4.81430200	-0.02381100	2.71813500
N	-1.78443900	0.17788200	2.18607400
N	2.50273300	-1.17543300	0.61234800
O	2.16073400	-2.81582000	-0.92490400
O	-3.21109400	-1.22926000	3.23802900
C	4.78023600	-0.43360300	-0.10813600
C	5.53293500	0.39487800	-0.94657600
C	5.33821700	-0.87243300	1.09544800
C	6.82206600	0.78500300	-0.58684700
H	5.09337400	0.75720000	-1.87231300
C	6.62560500	-0.47821400	1.46132800
H	4.74159600	-1.49722900	1.75203700
C	7.37112700	0.35200400	0.62204000
H	7.39368900	1.43491500	-1.24304900
H	7.04546900	-0.81480800	2.40495000
H	8.37149800	0.66220000	0.90894600
C	-3.45533800	1.85517700	1.48466100
C	-3.91058300	1.19916700	0.33527500
C	-3.53361700	3.24772100	1.55614300
C	-4.45052700	1.92838000	-0.72160300
H	-3.81858900	0.12147100	0.24691900
C	-4.07751500	3.97991400	0.50032800
H	-3.16136800	3.76130000	2.43849100
C	-4.54099500	3.32031500	-0.63881300

H	-4.78393400	1.40375300	-1.61010600
H	-4.13163400	5.06231300	0.56518700
H	-4.96434200	3.88838900	-1.46205300
Cu	-0.20818300	0.55974700	1.04069200
P	-1.01605900	0.45647200	-1.99148200
O	-0.03798600	-0.09027400	-0.89016400
H	-1.97210100	3.17078300	-1.56372400
C	-0.12735300	0.11988700	-3.61354500
H	-0.83066600	0.38829200	-4.41414600
C	1.09573800	1.04267000	-3.68915600
H	1.63881000	0.89993300	-4.63091200
H	0.81159700	2.09612100	-3.60745900
H	1.78160700	0.82648500	-2.86312800
C	0.26693400	-1.35555300	-3.74424400
H	-0.61464700	-1.99588000	-3.83647500
H	0.90051600	-1.51798100	-4.62587000
H	0.81547700	-1.67980100	-2.85530200
C	-2.39561700	-0.76606900	-2.09431100
C	-3.46357500	-0.59996200	-2.98949100
C	-2.45239700	-1.81864200	-1.16919700
C	-4.55931500	-1.46232000	-2.95941600
H	-3.44102400	0.21601500	-3.70935200
C	-3.55332300	-2.67440100	-1.12783000
H	-1.61480600	-1.94688700	-0.49606300
C	-4.61310200	-2.49765500	-2.02047300
H	-5.37639700	-1.32596800	-3.66297200
H	-3.58030000	-3.48394400	-0.40270200
H	-5.47272600	-3.16082000	-1.98955700
C	2.46734400	1.70020300	1.68621200
C	1.28868300	2.76356600	-0.02524300
C	2.47980300	3.38270000	-0.46833600
C	3.66824100	3.13564300	0.19877000
C	3.66806100	2.29494000	1.31710300
H	2.42491300	0.99334700	2.50659400
H	2.43531600	4.04828300	-1.32273200
H	4.59233600	3.59232000	-0.14260500
H	4.57956100	2.06462100	1.85472800
N	1.31015000	1.90040100	1.03787400
C	0.04712400	3.01851300	-0.63578300
C	-1.00684800	2.82133500	-1.26304900

TS2-S

Sum of electronic and thermal Free Energies = -2489.860807

C	1.30984800	-3.46920400	0.18020200
C	-0.74604700	-3.21988300	1.16165100

C	-0.56965800	-4.37784100	1.93266100
C	0.60969000	-5.10396100	1.78291800
C	1.57236100	-4.65017100	0.88834200
H	-1.34577400	-4.68695600	2.62089300
H	0.77545700	-6.00837200	2.35919800
H	2.50922300	-5.17256400	0.73839700
N	0.17182300	-2.77096200	0.30006800
C	-1.99605100	-2.43967000	1.27758400
C	-3.62332400	-0.95533600	1.08812400
C	-4.06924300	-2.08711800	2.06056300
H	-3.47055000	-0.01964800	1.63735700
H	-4.36649200	-1.72887300	3.04756100
H	-4.86487200	-2.70871600	1.63807500
C	2.31072500	-2.88865600	-0.71866500
C	3.47517000	-1.45012800	-1.96706200
C	4.22286400	-2.81460300	-1.88698400
H	3.26142700	-1.18187500	-3.00501700
H	4.22437000	-3.35444300	-2.83854000
H	5.24094000	-2.72572400	-1.50651200
N	2.20168200	-1.73571800	-1.27736300
N	-2.30824400	-1.40609000	0.60108300
O	-2.88297800	-2.90419500	2.20904300
O	3.44967900	-3.59161000	-0.93541500
C	-4.60049100	-0.70205700	-0.03753400
C	-5.51596200	0.35078100	0.04180700
C	-4.61538500	-1.53243500	-1.16374500
C	-6.42536500	0.58122100	-0.99144600
H	-5.51074600	1.00073300	0.91275400
C	-5.52047700	-1.30372800	-2.19871300
H	-3.89524400	-2.34159300	-1.23423400
C	-6.42761600	-0.24407700	-2.11638000
H	-7.12512100	1.40840400	-0.92026800
H	-5.51364400	-1.94700000	-3.07355700
H	-7.12866600	-0.06222000	-2.92523400
C	4.23518400	-0.31514700	-1.31000400
C	4.18483000	-0.11788400	0.07325900
C	5.03575900	0.51949700	-2.09588100
C	4.92802700	0.90612000	0.65987000
H	3.50846700	-0.70521700	0.68237600
C	5.78308900	1.54010800	-1.50856700
H	5.06269400	0.38005300	-3.17361900
C	5.73001600	1.73568900	-0.12651800
H	4.86358600	1.06680400	1.73129800
H	6.39436400	2.18854600	-2.12900600

H	6.30217600	2.53631700	0.33256000
Cu	0.77872500	-0.42530300	-0.81706300
P	1.50895300	1.78653000	1.26459000
O	1.20658600	0.25594000	1.04531500
H	3.27539700	2.64356500	-0.85194900
C	-1.76956500	0.39850400	-2.06907700
C	-0.11038500	2.04129400	-1.96949800
C	-1.04791300	3.01937700	-2.36803500
C	-2.36248800	2.65494500	-2.59291000
C	-2.74078300	1.31322600	-2.45100200
H	-2.01568500	-0.64246700	-1.90451500
H	-0.71889700	4.04728900	-2.46060200
H	-3.09445700	3.40663700	-2.87377800
H	-3.76035200	0.98448700	-2.61145400
N	-0.49187400	0.73256600	-1.82079500
C	1.23810700	2.35831700	-1.70508100
C	2.22101000	2.48068900	-0.94500100
C	1.87319300	1.88749100	3.12232200
H	2.80548900	1.31211700	3.21500800
C	0.78673200	1.18770400	3.94291700
H	1.07546200	1.11121700	4.99931800
H	-0.15360300	1.74668100	3.88915900
H	0.60524700	0.18283200	3.55255300
C	2.12965800	3.32229100	3.58584300
H	2.46351700	3.34332500	4.63018600
H	2.89719100	3.81348100	2.97687900
H	1.21588600	3.92164600	3.51941100
C	-0.11323500	2.66791000	1.18315500
C	-0.15494700	4.06659700	1.10498700
C	-1.30917300	1.94708600	1.07985000
C	-1.36870400	4.73661100	0.95551200
H	0.77283600	4.63226200	1.13166800
C	-2.52206900	2.61547800	0.90928900
H	-1.26250100	0.86273900	1.08620600
C	-2.55655700	4.00959600	0.85296100
H	-1.38741400	5.82173700	0.89792100
H	-3.44032900	2.05247000	0.78165800
H	-3.50166300	4.52671000	0.71104500

Int3-R

Sum of electronic and thermal Free Energies = -2489.899688

C	-0.62896200	3.24571600	0.85913000
C	1.15408600	2.22419100	1.87041500
C	0.88227200	2.85773500	3.09161300
C	-0.17748200	3.75926400	3.14995100

C	-0.96260300	3.95841900	2.01641000
H	1.49101900	2.63501000	3.95921000
H	-0.40455300	4.28128600	4.07385200
H	-1.82417200	4.61510100	2.02276400
N	0.42855200	2.43358500	0.77191200
C	2.22917600	1.21844900	1.75533300
C	3.63458000	-0.31930000	1.03210700
C	3.73312300	-0.22068600	2.58292200
H	3.13768100	-1.25112800	0.73395700
H	3.56663300	-1.16657700	3.09959900
H	4.68028400	0.22122400	2.90830600
C	-1.51183400	3.22103000	-0.31506300
C	-2.61722800	2.47820300	-2.11236800
C	-3.27458500	3.78304900	-1.58173900
H	-2.31989000	2.58171500	-3.15829700
H	-3.12124300	4.63720100	-2.24711700
H	-4.33447700	3.67162100	-1.35213200
N	-1.40341000	2.37092800	-1.27286300
N	2.73062100	0.78268700	0.67096600
O	2.65815500	0.68552900	2.94130800
O	-2.57148800	4.05924200	-0.33753800
C	4.97323700	-0.25722000	0.33717600
C	5.70553400	-1.43141700	0.13647300
C	5.50270500	0.96032000	-0.09780300
C	6.94862100	-1.39228300	-0.49288100
H	5.28619400	-2.38354500	0.45153100
C	6.74303500	1.00107900	-0.73496800
H	4.92046900	1.86564300	0.03867400
C	7.46981000	-0.17409700	-0.93388300
H	7.50398600	-2.31200200	-0.65133300
H	7.13957200	1.95054200	-1.08280200
H	8.43291600	-0.14232100	-1.43437400
C	-3.48398700	1.24448000	-1.95621100
C	-3.90683500	0.83211600	-0.68582800
C	-3.88353500	0.51800100	-3.07878200
C	-4.74073800	-0.27407300	-0.54640700
H	-3.55684600	1.35333000	0.19883500
C	-4.71380000	-0.59576800	-2.94117900
H	-3.53634100	0.81760400	-4.06368500
C	-5.14891800	-0.98816200	-1.67587600
H	-5.05154200	-0.59380500	0.44205400
H	-5.00993900	-1.15897000	-3.82068000
H	-5.78888200	-1.85806700	-1.56377400
Cu	-0.11158300	0.90677800	-1.27799000

P	-1.23858800	-1.51706600	0.40808000
O	-0.96746800	-0.03111500	0.66220500
H	-2.64678600	-2.09210400	-1.54143300
C	2.45465800	-0.03772300	-2.24259400
C	0.73613200	-1.65585900	-2.19522600
C	1.72499400	-2.67096900	-2.39560900
C	3.05070100	-2.33221100	-2.52758900
C	3.43763600	-0.97496100	-2.49423600
H	2.70409600	1.01112400	-2.13216800
H	1.39399900	-3.70176100	-2.46569100
H	3.79778200	-3.10841100	-2.67398300
H	4.46913400	-0.66996900	-2.61443400
N	1.15394000	-0.34381000	-2.05102400
C	-0.63814800	-1.93365300	-2.24033500
C	-1.59110900	-1.95811200	-1.30420800
C	-2.67048900	-2.04722600	1.40631300
C	-3.40778800	-3.19670400	1.09253500
C	-3.08769600	-1.22695400	2.46251200
C	-4.54915400	-3.52037300	1.82675700
H	-3.10099100	-3.82330700	0.26089700
C	-4.22622000	-1.55308000	3.19855500
H	-2.52244900	-0.32414500	2.67238900
C	-4.96006600	-2.69868600	2.87879700
H	-5.12214000	-4.40787000	1.57438900
H	-4.54717800	-0.91313400	4.01566000
H	-5.85185600	-2.94871900	3.44613000
C	0.20898400	-2.51286800	0.98071400
H	1.01902500	-2.15990500	0.33222700
C	0.54287000	-2.16495000	2.43620600
H	-0.26281300	-2.48018400	3.10835600
H	1.45547600	-2.68519400	2.75004700
H	0.69266700	-1.09155900	2.56755100
C	0.02502000	-4.01770800	0.76714000
H	0.93943400	-4.55388000	1.04383300
H	-0.78868000	-4.40638300	1.38894800
H	-0.20234900	-4.24403400	-0.27719200

Int3-S

Sum of electronic and thermal Free Energies = -2489.899743

C	-1.09300300	3.62940000	0.21577300
C	0.96871900	3.33711300	1.17151000
C	0.88359500	4.56752100	1.84016600
C	-0.25521200	5.34767200	1.65608500
C	-1.26742600	4.87957000	0.82531000
H	1.69707600	4.88841700	2.47796700

H	-0.35077300	6.30702900	2.15416300
H	-2.17310800	5.44630700	0.64758700
N	0.00081900	2.87304200	0.37602500
C	2.17692000	2.49739700	1.32269600
C	3.72582800	0.92398400	1.20392300
C	4.25104800	2.09858800	2.08576400
H	3.57481500	0.02703200	1.81435600
H	4.52428200	1.79871800	3.09939500
H	5.08735900	2.62794000	1.62019300
C	-2.13932700	3.05380900	-0.63589300
C	-3.38724900	1.63356300	-1.83439900
C	-4.05368400	3.04085900	-1.80811300
H	-3.17412100	1.31518400	-2.85730900
H	-4.03041800	3.54087600	-2.78042900
H	-5.07241800	3.02668000	-1.41948400
N	-2.10524100	1.87191800	-1.13913100
N	2.40419500	1.38491200	0.74551600
O	3.12599800	3.00608200	2.16621000
O	-3.23164500	3.81282900	-0.89178200
C	4.64863800	0.57791200	0.05629600
C	5.53421900	-0.49761500	0.15930100
C	4.65322400	1.35339100	-1.10821000
C	6.40545800	-0.80369500	-0.88678000
H	5.53930100	-1.10418900	1.06105000
C	5.51904600	1.04826000	-2.15663100
H	3.95521500	2.17978600	-1.19811500
C	6.39776300	-0.03249400	-2.04924600
H	7.08222800	-1.64795600	-0.79655000
H	5.50213000	1.64761400	-3.06185700
H	7.06756100	-0.27415700	-2.86879200
C	-4.21290600	0.56779400	-1.14551200
C	-4.33625100	0.55012200	0.24747900
C	-4.90592300	-0.37444700	-1.90810700
C	-5.16664300	-0.38388100	0.86427100
H	-3.75850100	1.24135400	0.85115700
C	-5.72775100	-1.31752500	-1.29047200
H	-4.78610700	-0.38369600	-2.98753300
C	-5.86476000	-1.32005400	0.09799600
H	-5.26350500	-0.38470900	1.94575900
H	-6.25100000	-2.05375500	-1.89260300
H	-6.50322700	-2.05296600	0.58188500
Cu	-0.74090100	0.52160500	-0.87620200
P	-1.40643700	-1.83428200	0.95136800
O	-1.14167900	-0.32878700	1.12030400

H	-3.23967000	-2.36567200	-0.62309000
C	1.77347200	-0.52211300	-1.93996700
C	-0.03582800	-2.03540200	-1.88909200
C	0.86251300	-3.07954100	-2.25755100
C	2.20214300	-2.81932400	-2.42185600
C	2.68549900	-1.50171700	-2.27907200
H	2.08625100	0.50241100	-1.78183100
H	0.46029700	-4.07889900	-2.38003400
H	2.88496300	-3.62839200	-2.66853600
H	3.73072500	-1.25342600	-2.41378700
N	0.46348200	-0.76169700	-1.72188200
C	-1.43030300	-2.25906700	-1.76146500
C	-2.15239500	-2.27497400	-0.62769500
C	-2.48408300	-2.39744600	2.34499400
H	-3.38476200	-1.79051300	2.19475300
C	-1.84659700	-2.01638000	3.68623300
H	-2.52933400	-2.23628000	4.51483400
H	-0.92459500	-2.58484700	3.85001500
H	-1.59713700	-0.95274800	3.70900200
C	-2.87897600	-3.87642700	2.28372600
H	-3.64334200	-4.09309800	3.03858500
H	-3.28220600	-4.15034200	1.30430300
H	-2.02067900	-4.52134400	2.49282200
C	0.16405800	-2.75698200	1.06326400
C	0.21594900	-4.14733000	0.89024000
C	1.35046400	-2.03786300	1.24431100
C	1.43734600	-4.81529000	0.92595200
H	-0.69295300	-4.70017800	0.68044000
C	2.57339600	-2.70864200	1.25495400
H	1.29815700	-0.95670900	1.32234300
C	2.61948400	-4.09441600	1.10589100
H	1.47056500	-5.89211700	0.78759000
H	3.49721300	-2.14939900	1.35289600
H	3.57543300	-4.61046000	1.11019400

E-Int4-R

Sum of electronic and thermal Free Energies = -2718.853233

C	-2.42697100	3.65542600	0.13974300
C	-3.90329300	2.13693800	-0.74541800
C	-4.81698700	3.14822600	-1.07336800
C	-4.49453100	4.46494200	-0.75057200
C	-3.27215800	4.73602800	-0.14203500
H	-5.74257200	2.89368800	-1.57439400
H	-5.18157100	5.27126900	-0.98573500
H	-2.96288000	5.74503100	0.10185500

N	-2.74237500	2.38374100	-0.13203000
C	-4.15609000	0.73049200	-1.12398800
C	-3.97853300	-1.44553900	-1.49386400
C	-5.27507000	-0.91185500	-2.16955100
H	-3.24414200	-1.74602600	-2.24829800
H	-5.29714200	-1.07036800	-3.24941700
H	-6.18895400	-1.31538900	-1.72650900
C	-1.08590400	3.84833600	0.70844100
C	0.98001500	3.48572300	1.48935400
C	0.71811600	5.01342100	1.34707700
H	1.04028700	3.19419900	2.54406600
H	0.86381600	5.57745000	2.26918500
H	1.30882000	5.45543900	0.54061400
N	-0.25738500	2.89399800	0.94305800
N	-3.42434200	-0.26119100	-0.80872000
O	-5.24226600	0.51912100	-1.92552900
O	-0.68736400	5.10975800	0.97696500
C	-4.16577500	-2.60686800	-0.54101900
C	-3.27961800	-3.68632100	-0.58396000
C	-5.18224800	-2.59822200	0.42062400
C	-3.41837600	-4.75219100	0.30452100
H	-2.46224600	-3.67307200	-1.29853000
C	-5.32644800	-3.66468500	1.30655100
H	-5.85873400	-1.75020000	0.48765900
C	-4.44684400	-4.74783900	1.24617600
H	-2.71850000	-5.58113500	0.26535600
H	-6.12087400	-3.64846400	2.04682900
H	-4.55708600	-5.57744100	1.93796000
C	2.24131000	3.03321100	0.78866200
C	2.21967100	2.52327800	-0.51154100
C	3.46252600	3.14939200	1.46323800
C	3.40834300	2.12442300	-1.12489100
H	1.27779400	2.36884800	-1.02531700
C	4.65028600	2.76059800	0.84458600
H	3.48089600	3.52328400	2.48438600
C	4.62421400	2.24161700	-0.45195600
H	3.36448500	1.66020000	-2.10219700
H	5.59054700	2.83867100	1.38231300
H	5.54054000	1.89653400	-0.92005900
Cu	-0.68585600	1.00550800	0.69270100
P	2.93461600	-1.26058400	-0.46646100
O	2.33607100	-0.72384600	-1.75319500
H	3.32386200	0.38810500	1.32772000
C	2.43091500	-3.01608800	-0.18337800

H	1.38007300	-2.93408000	0.11790600
C	2.50762600	-3.79043100	-1.50361700
H	1.91331100	-3.29797000	-2.27554100
H	3.54242000	-3.85693200	-1.85911600
H	2.13406300	-4.81151400	-1.36814200
C	3.21671600	-3.69994000	0.93970500
H	4.26702000	-3.83204700	0.66085700
H	3.18155400	-3.11971000	1.86501900
H	2.79614700	-4.69024600	1.14507800
C	4.75889300	-1.22677800	-0.59406800
C	5.59745300	-1.26510800	0.52777800
C	5.32235300	-1.07969500	-1.86799700
C	6.98031200	-1.16144000	0.37628900
H	5.16671900	-1.35732700	1.51978700
C	6.70505300	-0.97852900	-2.01986200
H	4.65665800	-1.01946500	-2.72323600
C	7.53542500	-1.01766900	-0.89712000
H	7.62419500	-1.18519900	1.25079200
H	7.13564800	-0.86288600	-3.01052800
H	8.61204300	-0.93229900	-1.01362300
C	-2.02579300	-1.30860300	1.91161900
C	0.35348700	-1.28248900	2.02287900
C	0.28693200	-2.47213100	2.83903800
C	-0.92258300	-3.00491300	3.18453800
C	-2.12916700	-2.39496200	2.74375800
H	-2.90179100	-0.83913700	1.48172300
H	1.21684700	-2.90692000	3.18770100
H	-0.96063400	-3.90166800	3.79834200
H	-3.10045700	-2.79204800	3.00568700
N	-0.84352400	-0.77090700	1.50035700
C	1.51317600	-0.57922100	1.83090800
C	2.58789300	-0.37964500	1.08658700
C	-0.43160800	-0.43429500	-1.99029300
O	-0.45188100	0.70613900	-1.53634200
C	-0.15008300	-0.73789500	-3.43292800
H	0.93240400	-0.87975000	-3.49785900
H	-0.65356600	-1.65218400	-3.75391000
H	-0.44510400	0.10865900	-4.05430700
O	-0.66454900	-1.52005600	-1.25642400
H	-0.80581100	-1.23951500	-0.31186200

Z-Int4-R

Sum of electronic and thermal Free Energies = -2718.850725

C	1.04290300	3.31348800	-1.15803000
C	3.10647000	2.55392700	-0.52358000

C	3.65037900	3.84480100	-0.57067500
C	2.83020800	4.89485800	-0.97448900
C	1.49592300	4.63360100	-1.27475900
H	4.68644500	4.00161900	-0.29624200
H	3.22036900	5.90529300	-1.03859900
H	0.80947800	5.41939700	-1.56475100
N	1.83144900	2.29008300	-0.81981900
C	3.91983400	1.40447600	-0.07681400
C	4.63064400	-0.61321200	0.47422400
C	5.65121100	0.44579000	0.97610300
H	4.05958200	-1.02580700	1.31376600
H	5.86905500	0.38184900	2.04260900
H	6.58294300	0.42793700	0.40196500
C	-0.38706700	2.99534600	-1.30570700
C	-2.41320200	2.07242200	-1.05176800
C	-2.52603100	3.39862600	-1.84637800
H	-2.83373600	1.24542500	-1.62254600
H	-2.75709800	3.23828800	-2.90321400
H	-3.22326400	4.11227800	-1.40843400
N	-0.94671000	1.89258200	-0.94980000
N	3.67880400	0.18131000	-0.32388900
O	4.98595600	1.71528900	0.72551700
O	-1.18733900	3.97134500	-1.77123900
C	5.25748900	-1.74724200	-0.29866700
C	5.49320800	-2.97501500	0.32707500
C	5.63285000	-1.57917600	-1.63574700
C	6.10496200	-4.01848900	-0.36866400
H	5.18183400	-3.11635100	1.35832100
C	6.23963100	-2.62198100	-2.33416200
H	5.42590300	-0.63467200	-2.12963200
C	6.47997600	-3.84342100	-1.70127400
H	6.27976200	-4.96878400	0.12678200
H	6.51928200	-2.48450700	-3.37450200
H	6.94966000	-4.65650300	-2.24647500
C	-3.06270800	2.12106700	0.31716200
C	-2.35297300	2.51857400	1.45586000
C	-4.41163900	1.77526100	0.43181800
C	-2.99585800	2.56773000	2.69250500
H	-1.29533600	2.74328700	1.39755300
C	-5.05152100	1.82078500	1.66886500
H	-4.95630500	1.42121200	-0.43583500
C	-4.34457900	2.22275000	2.80247500
H	-2.43244100	2.85995400	3.57366900
H	-6.08096300	1.49078200	1.74395900

H	-4.83632400	2.24608000	3.77051700
Cu	-0.13424400	0.35372600	-0.16752000
P	-4.25809000	-1.73180300	0.43088600
O	-5.62959600	-1.14004100	0.65610100
H	-3.42498400	-0.43118200	2.33527100
C	-4.30913200	-3.58521600	0.49392100
H	-4.48799500	-3.79421600	1.55736200
C	-5.52716200	-4.05423400	-0.31515600
H	-6.43702100	-3.54760500	0.01239700
H	-5.38478300	-3.84261400	-1.38112600
H	-5.65958800	-5.13608000	-0.20469800
C	-3.03057100	-4.30313100	0.05095300
H	-2.82315000	-4.11410900	-1.00670700
H	-2.15567900	-4.00114400	0.62761900
H	-3.15712200	-5.38508100	0.17159800
C	-3.64522200	-1.31333700	-1.24835900
C	-2.31807200	-1.48378400	-1.66770100
C	-4.57718300	-0.75417000	-2.13422000
C	-1.92705900	-1.07941300	-2.94543800
H	-1.58362000	-1.90999000	-0.99725000
C	-4.18553700	-0.35277800	-3.41296400
H	-5.59956500	-0.62572000	-1.79178400
C	-2.85724200	-0.50996700	-3.81840400
H	-0.89252000	-1.20365200	-3.25125800
H	-4.91399400	0.08295600	-4.09105700
H	-2.54951400	-0.19485500	-4.81143900
C	1.49730400	-2.02947300	0.09462500
C	-0.52088600	-2.00657900	1.38493900
C	-0.09452400	-3.26749700	1.98133800
C	1.03549100	-3.88356700	1.54348400
C	1.84497900	-3.27918000	0.52790500
H	2.11862100	-1.47964100	-0.60135300
H	-0.72851700	-3.70901400	2.74211600
H	1.32637300	-4.84022400	1.96991900
H	2.72932600	-3.76538200	0.13654300
N	0.41284900	-1.32430300	0.55651400
C	-1.73426000	-1.48455200	1.60600000
C	-2.99877500	-1.14099600	1.62153400
C	1.74974800	1.37722300	2.53805000
O	0.82321800	1.92252900	1.96067900
C	2.75749400	2.11198500	3.38816800
H	3.01709500	1.52852000	4.27416500
H	3.66913200	2.25494900	2.79779800
H	2.35925700	3.08577400	3.67241400

O	2.01495300	0.07297500	2.45300700
H	1.38454100	-0.35175300	1.80749900

E-Int4-S

Sum of electronic and thermal Free Energies = -2718.850099

C	3.26917900	3.02426500	-0.12135800
C	4.18323000	1.06371200	0.65469500
C	5.41029100	1.68796600	0.91773400
C	5.54141800	3.04477500	0.62593000
C	4.45127900	3.73911900	0.10826100
H	6.22222600	1.11600100	1.34929900
H	6.47871700	3.55771800	0.81531200
H	4.49572400	4.79965500	-0.10793800
N	3.14310600	1.71425500	0.12450600
C	3.95100300	-0.35126000	1.01790000
C	3.03055000	-2.32802800	1.42166400
C	4.48902600	-2.31595900	1.96566900
H	2.31324200	-2.39370100	2.24599000
H	4.54181500	-2.45621700	3.04748600
H	5.14602200	-3.03905400	1.47835800
C	2.04553700	3.67574600	-0.60929500
C	-0.04829900	4.03577800	-1.31519100
C	0.68279200	5.38843300	-1.08003400
H	-0.20215900	3.85573300	-2.38562400
H	0.67939200	6.05353500	-1.94411900
H	0.29574300	5.91239900	-0.20181600
N	0.94634400	3.05221800	-0.84549800
N	2.86761800	-0.98788500	0.82224300
O	4.97698400	-0.98065600	1.66378400
O	2.06350500	5.01220100	-0.80422900
C	2.68530700	-3.41742700	0.42385600
C	1.39270400	-3.95335000	0.42030500
C	3.60127300	-3.84730500	-0.54191800
C	1.02486800	-4.90861800	-0.52511600
H	0.66071800	-3.59270200	1.13559400
C	3.24015100	-4.81634000	-1.47764100
H	4.59920500	-3.41877600	-0.57837300
C	1.95047900	-5.34966900	-1.47043000
H	0.00931500	-5.29023100	-0.52773500
H	3.96252300	-5.14581100	-2.21881700
H	1.66607400	-6.09487500	-2.20728300
C	-1.38336600	3.94549800	-0.61059500
C	-1.49613900	3.41855800	0.67881600
C	-2.52474600	4.42994300	-1.25971700
C	-2.73927300	3.37478200	1.31108400

H	-0.63395500	2.98511900	1.17118700
C	-3.76355400	4.40570500	-0.61962700
H	-2.44599700	4.81682400	-2.27294500
C	-3.87135000	3.87811300	0.66946700
H	-2.82440700	2.89184500	2.27679500
H	-4.64356200	4.78292600	-1.13226700
H	-4.83821700	3.84113500	1.16299600
Cu	0.75022000	1.12686100	-0.63242200
P	-3.42278100	-0.17936800	0.67486500
O	-2.84541800	0.30252800	1.99340200
H	-3.32840500	1.62119300	-0.97833300
C	1.33551300	-1.36592100	-1.98797400
C	-0.94919700	-0.68626000	-2.01155200
C	-1.18554400	-1.67793700	-3.03788800
C	-0.16322400	-2.45238600	-3.50066400
C	1.15337300	-2.28962700	-2.98356600
H	2.30116600	-1.22286600	-1.51947700
H	-2.18531000	-1.76017700	-3.44917500
H	-0.35553500	-3.19369700	-4.27252200
H	1.98166600	-2.89301400	-3.32930000
N	0.34109700	-0.59269800	-1.46471300
C	-1.88974000	0.23151500	-1.64551400
C	-2.84638100	0.64993700	-0.83772300
C	0.07272800	-0.10986000	2.04988200
O	0.42313700	0.97762800	1.59722600
C	-0.26758500	-0.32072400	3.49637200
H	-1.34207800	-0.12815000	3.57626000
H	-0.06136700	-1.34681900	3.80773600
H	0.27964200	0.39179000	4.11484900
O	-0.05310100	-1.20327800	1.31172300
H	0.15902400	-0.99387800	0.35872600
C	-5.25740300	0.07602300	0.66069000
H	-5.34539600	1.17160700	0.68537900
C	-5.87232900	-0.50086600	1.94035800
H	-6.93784700	-0.25315400	2.00405000
H	-5.78081400	-1.59273500	1.95318200
H	-5.36526100	-0.10867900	2.82502300
C	-5.94248000	-0.44700700	-0.60573400
H	-5.48285700	-0.04117900	-1.51180700
H	-5.88598100	-1.53885000	-0.65660100
H	-7.00233600	-0.16900200	-0.60747200
C	-3.14522500	-1.97160900	0.46541900
C	-3.31555900	-2.63178200	-0.75682600
C	-2.74512500	-2.70503100	1.58973200

C	-3.07875000	-4.00223100	-0.85831400
H	-3.61374600	-2.07011700	-1.63302900
C	-2.51682100	-4.07675300	1.49262300
H	-2.59964300	-2.17978900	2.52685700
C	-2.68128600	-4.72671200	0.26673700
H	-3.19441600	-4.49976300	-1.81636100
H	-2.20686600	-4.63874900	2.36909400
H	-2.50266600	-5.79578800	0.18995000

E-Pr-R

Sum of electronic and thermal Free Energies = -1091.298777

P	0.95113000	-0.86229100	-0.43834600
O	0.88893900	-1.34698000	-1.85997200
H	-0.67840500	0.16100200	1.19691100
C	1.51301300	-2.17875300	0.73256900
H	0.75131800	-2.95946400	0.60521500
C	2.86545000	-2.72327500	0.25350800
H	2.81368500	-3.03026900	-0.79341800
H	3.64623800	-1.96117500	0.34865100
H	3.16211000	-3.58662500	0.85801000
C	1.55297500	-1.75240400	2.20403500
H	2.29174000	-0.96175900	2.36565500
H	0.58155200	-1.39521300	2.55881000
H	1.83944700	-2.60362400	2.83030100
C	2.10859100	0.53353400	-0.22775200
C	2.11848800	1.37527000	0.89336500
C	3.03645500	0.74504300	-1.25573800
C	3.05206600	2.40604300	0.98917700
H	1.39652000	1.23686800	1.69162800
C	3.96988900	1.77617500	-1.15711000
H	3.00054800	0.10159200	-2.12877300
C	3.98033000	2.60497200	-0.03451500
H	3.05271600	3.05619700	1.85875900
H	4.68556700	1.93588400	-1.95777100
H	4.70602100	3.40907000	0.04106600
C	-5.28002200	0.14551200	-0.94799200
C	-3.09198400	0.05487000	-0.23798300
C	-3.42659700	0.68827000	0.97033100
C	-4.74751900	1.05155200	1.20101400
C	-5.70257000	0.77577300	0.22278000
H	-5.99440400	-0.08506300	-1.73577800
H	-2.66329200	0.89449400	1.71241300
H	-5.02847900	1.54302600	2.12743100
H	-6.74527200	1.04182500	0.35941200
N	-4.01509500	-0.20932200	-1.18489300

C	-1.72517000	-0.36922600	-0.57570500
C	-0.63453100	-0.26913700	0.19950800
H	-1.62359600	-0.80662200	-1.56626500

E-Pr-S

Sum of electronic and thermal Free Energies = -1091.296976

P	-1.19041000	-1.05820600	-0.62883000
O	-1.57144200	-1.48553900	-2.02141400
H	1.04452200	-2.01661600	-0.21603800
C	4.63036300	1.51370000	0.15648000
C	2.81539400	0.13548900	-0.16613600
C	3.64183500	-0.99941300	-0.19525700
C	5.01258300	-0.84048000	-0.03330900
C	5.52534000	0.44348800	0.15022800
H	4.99237200	2.53119800	0.28968600
H	3.21715700	-1.98402800	-0.35525300
H	5.67160800	-1.70289100	-0.05559300
H	6.58879400	0.61468300	0.27810700
N	3.31114800	1.37856700	0.00162700
C	1.35399000	0.07417200	-0.31730900
C	0.59304700	-1.03077500	-0.31888500
H	0.89392500	1.05368900	-0.41367900
C	-1.82828400	0.59995300	-0.21956500
C	-2.78434600	1.13114300	-1.09537400
C	-1.45300800	1.33109300	0.91760500
C	-3.36305700	2.37250400	-0.83383800
H	-3.05184900	0.56031900	-1.97864400
C	-2.03121100	2.57322600	1.17412600
H	-0.69928800	0.94178200	1.59288000
C	-2.98837400	3.09307700	0.30069700
H	-4.10199400	2.77911400	-1.51746000
H	-1.73066600	3.13727000	2.05168400
H	-3.43655200	4.06130900	0.50233500
C	-1.84821200	-2.21619400	0.65164000
H	-1.36894000	-3.16983600	0.38794900
C	-3.36358500	-2.36360500	0.46400300
H	-3.75699400	-3.13550900	1.13343600
H	-3.87444500	-1.42334100	0.69740700
H	-3.60457900	-2.63592100	-0.56590100
C	-1.47434500	-1.85252200	2.09245100
H	-1.97590500	-0.93233700	2.40529600
H	-1.79011700	-2.64920100	2.77403000
H	-0.39618200	-1.71138800	2.21679100

Z-Pr-R

Sum of electronic and thermal Free Energies = -1091.295383

P	1.38051400	-0.81153200	-0.49136700
O	1.32331700	-1.02169500	-1.98476800
H	1.23841400	-2.78810300	0.99030500
C	3.10935100	-0.81281600	0.15763100
H	3.45635100	-1.83880400	-0.02885800
C	3.95743100	0.15312900	-0.67938100
H	3.88074400	-0.08015700	-1.74347800
H	3.62510500	1.18616600	-0.53095400
H	5.00849600	0.09235800	-0.37937700
C	3.20442300	-0.51149200	1.65787400
H	2.87208600	0.50837200	1.87182600
H	2.59925800	-1.19564200	2.26023100
H	4.24296700	-0.60172200	1.99247300
C	0.65701400	0.79236000	-0.00622700
C	0.16841000	1.06409900	1.27801400
C	0.59287800	1.78263800	-0.99449700
C	-0.37794200	2.31273300	1.56842100
H	0.18580900	0.29213200	2.03954000
C	0.05094000	3.03277500	-0.69963900
H	0.94511900	1.54788000	-1.99378000
C	-0.43529100	3.29828200	0.58120600
H	-0.76753400	2.51324400	2.56166400
H	-0.00074800	3.79557700	-1.47061200
H	-0.86527400	4.26901700	0.80880700
C	-3.92637700	-0.51141200	0.66981600
C	-1.86370400	-1.41418400	0.21554000
C	-1.84262100	-0.83726500	-1.06349200
C	-2.91712400	-0.04174500	-1.44931000
C	-3.97793000	0.13868400	-0.56497200
H	-4.74873500	-0.41451900	1.37611800
H	-1.00697800	-1.01204500	-1.73294500
H	-2.92421900	0.42547000	-2.42902600
H	-4.83426100	0.75163000	-0.82598000
N	-2.90742600	-1.27983200	1.05920600
C	-0.75546700	-2.22684100	0.74565400
C	0.56148000	-2.07630700	0.52179900
H	-1.08168900	-3.00132200	1.43763700

2A

Sum of electronic and thermal Free Energies = -521.054733

C	-3.12670700	-1.20712300	0.00002100
C	-1.16262800	-0.01831600	-0.00003600
C	-1.86178100	1.20393900	0.00000500
C	-3.25157000	1.18342700	0.00002900
C	-3.90571400	-0.04818900	0.00003300

H	-3.60407700	-2.18567100	0.00002500
H	-1.30756000	2.13572600	0.00001300
H	-3.81457700	2.11212200	0.00005100
H	-4.98872100	-0.11416300	0.00005300
N	-1.79199500	-1.21352300	-0.00001100
C	0.26728000	-0.02010700	-0.00001400
C	1.49045500	0.00958800	0.00000700
Cu	3.29527200	-0.00250600	-0.00001100

S-Int2A

Sum of electronic and thermal Free Energies = -2686.729708

C	0.38681400	-0.00096800	-3.32917100
C	1.21714300	2.03264600	-2.65155600
C	2.27481200	1.95749200	-3.56366700
C	2.33213800	0.85895100	-4.42181800
C	1.38149900	-0.15430700	-4.29942100
H	3.02884400	2.73520100	-3.58384800
H	3.13100800	0.77497000	-5.15137600
H	1.42282000	-1.05516800	-4.90033700
N	0.27550700	1.09056500	-2.56066100
C	1.12049500	3.10015700	-1.63270100
C	0.45851800	4.36905000	0.05895600
C	1.99371900	4.53376600	-0.14277100
H	-0.05470100	5.33054200	-0.04604100
H	2.28100500	5.54587400	-0.44051100
H	2.57547300	4.22564100	0.72779100
C	-0.52068300	-1.08829500	-2.92535500
C	-1.79169800	-2.37332800	-1.62192100
C	-1.65361800	-3.01795900	-3.03797200
H	-1.29179200	-2.97759500	-0.85782100
H	-1.20384800	-4.01218900	-3.02318100
H	-2.60114300	-3.04575300	-3.58185300
N	-1.03981200	-1.12276900	-1.75683300
N	0.05044500	3.48016600	-1.05358200
O	2.31167200	3.63309400	-1.23493400
O	-0.75020500	-2.12762600	-3.76073000
C	0.04211700	3.72951300	1.36943100
C	-0.96441700	4.30097500	2.15114400
C	0.58728400	2.49561300	1.74801900
C	-1.42981700	3.64956800	3.29504800
H	-1.40074400	5.25129800	1.85251800
C	0.12108400	1.84340000	2.88846800
H	1.32566300	2.00169200	1.12242800
C	-0.89031200	2.41595300	3.66173300
H	-2.22038700	4.09849300	3.88926400

H	0.51341100	0.86396000	3.14134300
H	-1.26588500	1.89523200	4.53752000
C	-3.21569900	-2.11822700	-1.17872800
C	-3.84164300	-2.98948300	-0.28418300
C	-3.91440000	-1.00030200	-1.64704800
C	-5.15433400	-2.75715800	0.12661400
H	-3.28852100	-3.83545600	0.11289300
C	-5.22370400	-0.76321200	-1.23321000
H	-3.41454400	-0.29839800	-2.30668800
C	-5.84810900	-1.64324000	-0.34740100
H	-5.62820900	-3.43746700	0.82801900
H	-5.74963700	0.11783100	-1.58794700
H	-6.86568900	-1.45435600	-0.01830000
Cu	-0.39689300	0.31298700	-0.30518200
P	2.25423100	-1.15726300	-0.47020000
O	1.57495900	0.24972700	-0.15748300
C	3.96862100	-0.66749600	-1.11552300
H	3.75039700	-0.39659500	-2.15794000
C	4.55223100	0.56061000	-0.41781100
H	5.46776000	0.89660200	-0.92113200
H	3.83263700	1.38301900	-0.41969700
H	4.81425300	0.33470800	0.62203000
C	4.92625700	-1.86128300	-1.11770900
H	4.47572800	-2.74129800	-1.59173300
H	5.84909100	-1.62551900	-1.66067900
H	5.20856200	-2.14016500	-0.09653600
C	2.77374600	-1.71025600	1.25876900
C	2.96762700	-3.07936300	1.57194600
C	2.92767400	-0.76367800	2.30250700
C	3.35200300	-3.47417200	2.87257300
H	2.90800400	-3.82123500	0.77789300
C	3.32448100	-1.15913600	3.57400900
H	2.72235600	0.27583900	2.07175900
C	3.53996500	-2.51683400	3.86310700
H	3.50736300	-4.52675300	3.08969300
H	3.45475300	-0.41575000	4.35546100
H	3.83428200	-2.81952400	4.86310800
C	-2.77033300	1.98380000	0.26226500
C	-2.62260200	-0.00945100	1.46704200
C	-3.88678700	0.20929100	2.02965500
C	-4.59494500	1.35460000	1.68490800
C	-4.02805500	2.26230700	0.79000500
H	-2.26684100	2.65420900	-0.42734100
H	-4.28951400	-0.52326400	2.71844400

H	-5.57736500	1.53761100	2.10986100
H	-4.54392500	3.17276400	0.50501700
N	-2.08356300	0.87765800	0.57996300
C	-1.80072800	-1.12693100	1.77219900
C	-0.91361800	-1.96733100	1.91831000
Cu	0.84999900	-2.49997500	1.79040400

TS2A-R

Sum of electronic and thermal Free Energies = -2686.666915

C	-0.24065600	-1.16738800	3.19317400
C	1.84050800	-1.87366700	2.46987300
C	1.82134100	-2.98524500	3.31244200
C	0.68855000	-3.19230100	4.09779700
C	-0.36728100	-2.28118500	4.02543600
H	2.65512700	-3.67790000	3.31384600
H	0.62128100	-4.05848500	4.74739900
H	-1.27029100	-2.41056000	4.60981400
N	0.84314100	-0.96444600	2.42595000
C	2.85922300	-1.62458600	1.44536600
C	3.77816000	-0.77930400	-0.40453400
C	4.82611000	-1.62880800	0.37541700
H	3.60740500	-1.17816700	-1.40854300
H	5.13798500	-2.52387000	-0.16810800
H	5.70410200	-1.05829700	0.68265100
C	-1.28316500	-0.12303600	3.11868900
C	-2.38421800	1.78332500	2.87904300
C	-3.39303000	0.62564000	3.15733400
H	-2.37912100	2.49746300	3.71130400
H	-4.01161500	0.78259900	4.04352300
H	-4.02888400	0.41654000	2.29354400
N	-1.07244300	1.10562600	2.85281500
N	2.54985100	-0.97087800	0.38675400
O	4.13093600	-2.06348300	1.58625900
O	-2.55313900	-0.53529700	3.38849100
C	4.05341100	0.70573400	-0.50273100
C	3.88249400	1.37099200	-1.71710700
C	4.34940800	1.44370000	0.64949300
C	3.99367600	2.76087800	-1.77997700
H	3.60990900	0.80593200	-2.60231600
C	4.46638900	2.82979000	0.58768500
H	4.45825100	0.93590000	1.60458500
C	4.28057600	3.49231800	-0.62857600
H	3.83403900	3.27110200	-2.72446500
H	4.68594900	3.39501900	1.48850700
H	4.35195200	4.57467400	-0.67450900

C	-2.66559900	2.53853500	1.59783000
C	-2.48739700	1.90633100	0.36082600
C	-3.12478900	3.85702400	1.63619100
C	-2.77952200	2.58606700	-0.81882500
H	-2.08721400	0.89809000	0.31692700
C	-3.41156200	4.54005600	0.45232400
H	-3.25589800	4.35368500	2.59493900
C	-3.24102400	3.90367400	-0.77719800
H	-2.63060400	2.08512200	-1.77004400
H	-3.76585500	5.56622900	0.49222300
H	-3.46036000	4.43394200	-1.69944700
Cu	0.72643400	0.09138400	0.61180600
P	-1.15805100	-1.63674700	-1.23217600
O	-1.01832800	-0.93813100	0.16397100
C	-0.91730200	-3.48278400	-0.94652700
H	-1.22903400	-3.96865500	-1.88166300
C	0.56880800	-3.76693000	-0.69614900
H	0.74607600	-4.84222700	-0.57245100
H	1.19916000	-3.40485600	-1.51657700
H	0.90219700	-3.26145100	0.21358600
C	-1.78047500	-3.98889400	0.21321000
H	-2.84603800	-3.85595300	0.00901000
H	-1.59580900	-5.05422900	0.40180700
H	-1.54173600	-3.43120300	1.12459100
C	-2.95201600	-1.58388500	-1.63492100
C	-3.40269400	-1.90623300	-2.92325700
C	-3.88250300	-1.16228000	-0.67372600
C	-4.75761200	-1.82286900	-3.24122100
H	-2.68662600	-2.21463700	-3.68189400
C	-5.23637500	-1.07139900	-0.99388500
H	-3.52443300	-0.92189300	0.32141800
C	-5.67750600	-1.40098600	-2.27749600
H	-5.09691900	-2.07684000	-4.24169800
H	-5.95025100	-0.74480500	-0.24220400
H	-6.73174700	-1.32566800	-2.52788600
C	0.64545600	3.04956400	0.51481700
C	0.59958900	1.96616500	-1.55505000
C	0.39721700	3.20235900	-2.20538800
C	0.30751900	4.36285700	-1.45508400
C	0.43973800	4.29567800	-0.06436400
H	0.71653500	2.92777900	1.59020400
H	0.30893700	3.21532900	-3.28562800
H	0.13582900	5.31655400	-1.94582500
H	0.36155900	5.17903300	0.55831800

N	0.71878100	1.91467300	-0.19344500
C	0.69362300	0.74685800	-2.25027100
C	0.61679800	-0.43690300	-2.62578000
Cu	1.34020900	-1.79920500	-3.63235400

Int3A-R

Sum of electronic and thermal Free Energies = -2686.686459

C	0.50966300	3.61444200	0.09806100
C	2.57671500	2.64772300	0.48252400
C	3.04363000	3.78050300	1.14938500
C	2.17316700	4.85700500	1.30759000
C	0.88040000	4.76938400	0.78936700
H	4.04925100	3.79046300	1.55324400
H	2.49230300	5.74832200	1.83734800
H	0.16722100	5.57749600	0.89772900
N	1.34205200	2.57238300	-0.05805200
C	3.32390000	1.38972100	0.36726300
C	3.70407000	-0.77872900	0.02272200
C	5.05183600	-0.02662900	0.22491700
H	3.55063600	-1.53747900	0.79989400
H	5.58046400	-0.33794500	1.12877900
H	5.72018400	-0.09147400	-0.63541700
C	-0.82223100	3.46825600	-0.52486700
C	-2.51834200	2.87826000	-1.81923400
C	-3.03831000	3.74824600	-0.63381000
H	-2.63200800	3.41484100	-2.76892300
H	-3.58731100	4.63908200	-0.94465900
H	-3.63992800	3.16626000	0.06924100
N	-1.07352200	2.74861500	-1.54524100
N	2.70027500	0.28250600	0.22157400
O	4.67621000	1.37937800	0.39223500
O	-1.83129900	4.17414400	0.05561700
C	3.46544100	-1.40941200	-1.33122300
C	2.87360800	-2.67143800	-1.41132000
C	3.69729500	-0.68283600	-2.50507400
C	2.50949200	-3.19950000	-2.65093000
H	2.66604100	-3.22100800	-0.49685800
C	3.33874200	-1.21054100	-3.74292400
H	4.13192300	0.31237600	-2.44944900
C	2.73608100	-2.46921100	-3.81613900
H	2.02530500	-4.16942200	-2.70154200
H	3.51234600	-0.63559100	-4.64767800
H	2.43576000	-2.87261600	-4.77825600
C	-3.20385000	1.53503800	-1.94038300
C	-2.96706900	0.53664300	-0.98738400

C	-4.09131500	1.28777700	-2.99022500
C	-3.61596500	-0.69192100	-1.08794600
H	-2.25079000	0.70497000	-0.18949500
C	-4.74241800	0.05687200	-3.08944800
H	-4.26959000	2.05822500	-3.73686700
C	-4.50413200	-0.93424000	-2.13778100
H	-3.41381700	-1.46252900	-0.35237600
H	-5.42817200	-0.12746900	-3.91146900
H	-5.00130600	-1.89655200	-2.21773900
Cu	0.70537000	0.69433100	-0.59900600
P	-0.56574500	-0.61872100	2.07785400
O	-0.58745900	0.69021700	1.28214400
C	0.16050000	-0.33430700	3.74731400
H	0.34331300	-1.33287900	4.16802300
C	1.50268300	0.39221800	3.57481400
H	1.97748000	0.53734100	4.55146200
H	2.18540100	-0.17789900	2.93957000
H	1.34658400	1.37004900	3.11035400
C	-0.79103100	0.44928300	4.65817600
H	-1.72786000	-0.08625700	4.83069800
H	-0.31629900	0.62863600	5.62910600
H	-1.03157200	1.42322800	4.21851700
C	-2.27097000	-1.22164300	2.30022100
C	-2.57000900	-2.58970900	2.33480600
C	-3.30878800	-0.28356000	2.39208400
C	-3.89357000	-3.01245900	2.46334200
H	-1.76936100	-3.31497600	2.23608800
C	-4.62959100	-0.70874300	2.51953300
H	-3.07322000	0.77365100	2.33083700
C	-4.92292100	-2.07353200	2.55512100
H	-4.12190900	-4.07398500	2.48211400
H	-5.43070500	0.02201100	2.57664600
H	-5.95316700	-2.40487100	2.64579700
C	-0.32456300	-0.38907700	-3.13152200
C	-0.32831800	-1.86141000	-1.30121500
C	-1.06767900	-2.80767700	-2.07072400
C	-1.42633700	-2.50124500	-3.36385600
C	-1.04282400	-1.26932200	-3.92697000
H	-0.03221200	0.58920300	-3.49962100
H	-1.33499200	-3.75442100	-1.61467200
H	-1.99943800	-3.21480000	-3.94959500
H	-1.31283200	-0.99616600	-4.93976900
N	0.02048900	-0.64262900	-1.86178300
C	0.08244600	-2.08350300	-0.00546400

C	0.33690400	-1.93983800	1.24591300
Cu	1.71965500	-2.90151600	2.21753700

b-Cu-S-1

Sum of electronic and thermal Free Energies= -2394.620921

C	0.45449700	2.94631100	-0.70567100
C	-1.81099400	2.76519300	-1.12550500
C	-1.97469500	4.15051000	-1.13945400
C	-0.85480900	4.95149000	-0.92289600
C	0.37908700	4.34337600	-0.69574300
H	-2.95857800	4.57152500	-1.30995300
H	-0.94226300	6.03284600	-0.92497300
H	1.27578100	4.92257800	-0.51441700
N	-0.61629000	2.16469900	-0.92705100
C	-2.91593100	1.81566000	-1.28511600
C	-4.01176500	-0.12215500	-1.25446700
C	-4.98598600	1.03757500	-1.60634300
H	-3.99047800	-0.89432500	-2.02506000
H	-5.30691600	1.01046800	-2.65124900
H	-5.85648000	1.10129900	-0.95285600
C	1.72432400	2.24620400	-0.44555100
C	3.26984700	0.73004600	-0.01057000
C	3.92743800	2.14118000	-0.04423100
H	3.25257700	0.35804000	1.02172700
H	4.45344900	2.40331800	0.87499700
H	4.58845700	2.27122400	-0.90604500
N	1.87372400	0.98259600	-0.39215400
N	-2.70672400	0.55732600	-1.24547300
O	-4.19363100	2.25438400	-1.42060200
O	2.80187900	3.04607000	-0.18981600
C	-4.22092900	-0.77158600	0.10063000
C	-4.56348300	-0.00249100	1.21765100
C	-3.97965700	-2.13868800	0.25477600
C	-4.68317000	-0.59432500	2.47353400
H	-4.71967700	1.06821300	1.11304000
C	-4.11030100	-2.73284900	1.51051300
H	-3.64140800	-2.71424100	-0.60006100
C	-4.46136800	-1.96518500	2.62179800
H	-4.94891900	0.01212500	3.33463500
H	-3.93152000	-3.79850400	1.62022800
H	-4.56249100	-2.43058900	3.59826300
C	3.96554800	-0.29670300	-0.87849200
C	5.31665300	-0.57942100	-0.63864800
C	3.28846800	-0.98113400	-1.89218400
C	5.99001000	-1.52766700	-1.40723900

H	5.84480500	-0.06132700	0.15944000
C	3.96522100	-1.93681200	-2.65363300
H	2.23071600	-0.80784100	-2.06025600
C	5.31288900	-2.21055600	-2.42014100
H	7.03806100	-1.73674500	-1.21260100
H	3.42485600	-2.47154000	-3.42938900
H	5.83313700	-2.95391800	-3.01727200
O	0.13555300	-1.37585100	-2.04043800
C	-0.59673200	-2.39308600	-2.35418300
O	-1.78013000	-2.57433100	-2.02461900
C	0.14197400	-3.44820700	-3.17825400
H	0.57374700	-2.98990200	-4.07356400
H	0.97466400	-3.84792400	-2.58873300
H	-0.52660100	-4.26188400	-3.46366800
Cu	-0.68407200	0.02915000	-0.95091100
P	-0.22866000	-1.14381700	2.18224400
H	-0.98557500	-1.51794500	3.32329900
O	-1.06529100	-0.38858500	1.17616800
C	0.49891200	-2.74460700	1.66256600
H	0.88937100	-3.18055300	2.59544700
C	1.63865800	-2.57867800	0.65109700
H	2.05082500	-3.56250800	0.40352200
H	1.27686300	-2.12204300	-0.27216600
H	2.45402300	-1.96707800	1.04317300
C	-0.62650200	-3.64343900	1.12460300
H	-0.21712100	-4.62860800	0.87854700
H	-1.42257200	-3.78117400	1.86260300
H	-1.07077600	-3.21996000	0.21997400
C	1.10808600	-0.09572200	2.84020000
C	2.21921900	-0.59971000	3.52917400
C	1.01027500	1.27998600	2.59475100
C	3.22671700	0.26472500	3.95888000
H	2.31081400	-1.66461900	3.72059700
C	2.01829900	2.14252400	3.02370200
H	0.15384000	1.64913100	2.04167900
C	3.12829800	1.63477400	3.70273700
H	4.08995100	-0.13051200	4.48546700
H	1.94664500	3.20474800	2.81230700
H	3.91618500	2.30509300	4.03354900

c-Cu-S-1

Sum of electronic and thermal Free Energies= -2394.620090

C	0.76465100	2.77363900	-0.92183800
C	-1.54706000	2.87272300	-0.93588600
C	-1.52821200	4.26533800	-1.01614900

C	-0.30033200	4.92284600	-1.05948400
C	0.86604300	4.16318000	-1.01480500
H	-2.46752400	4.80319500	-1.05057300
H	-0.25408500	6.00437300	-1.12685300
H	1.84812500	4.61831300	-1.05074100
N	-0.42078600	2.12212400	-0.89240700
C	-2.80992300	2.12284300	-0.91707600
C	-4.31118300	0.50114100	-0.93461000
C	-5.02554500	1.87022300	-0.79527200
H	-4.44650200	0.11366300	-1.95578600
H	-5.81504100	2.04662400	-1.52632000
H	-5.40921200	2.03392200	0.21733400
C	1.95987500	1.92739600	-0.82842700
C	3.33727400	0.23402200	-0.45749800
C	4.14538300	1.45998500	-0.98169000
H	3.59032200	0.04370000	0.59186900
H	4.93781400	1.78254600	-0.30426100
H	4.54984400	1.29463400	-1.98371600
N	1.94294300	0.69688400	-0.49405200
N	-2.88668200	0.85604000	-0.81029500
O	-3.96563200	2.83733300	-1.03597900
O	3.15977500	2.52641200	-1.06234500
C	-4.72971000	-0.57973300	0.04012500
C	-6.02496400	-0.61589600	0.56584900
C	-3.82308600	-1.59520000	0.37310800
C	-6.41326900	-1.64507300	1.42592000
H	-6.74598700	0.15404000	0.30361100
C	-4.21627000	-2.62127700	1.23118000
H	-2.81941200	-1.58043300	-0.04031700
C	-5.50741900	-2.64984900	1.76291300
H	-7.42167400	-1.65902000	1.82923000
H	-3.50706100	-3.40354100	1.48360700
H	-5.80660300	-3.45152600	2.43195600
C	3.54818200	-1.03535200	-1.25140700
C	4.64573100	-1.85658800	-0.97027400
C	2.65466200	-1.40137900	-2.26138700
C	4.85160100	-3.03049500	-1.69492400
H	5.33295900	-1.58391700	-0.17196700
C	2.85298300	-2.58347300	-2.97541800
H	1.77408300	-0.80365600	-2.46648000
C	3.95187100	-3.39803400	-2.69853600
H	5.70492200	-3.66328900	-1.46809700
H	2.12825300	-2.85869300	-3.73487100
H	4.10438500	-4.31909400	-3.25370800

O	-0.69647200	-1.73878000	-0.87037900
C	-0.60694800	-2.11817900	-2.10771200
O	-0.38257800	-1.37887400	-3.07454500
C	-0.75034800	-3.62581400	-2.30198100
H	0.16188900	-4.11349300	-1.93943700
H	-1.58675500	-4.01349100	-1.71354400
H	-0.88626400	-3.86935700	-3.35697700
Cu	-0.53891400	0.16902900	-0.52063800
P	0.26232700	-0.53602300	2.65358700
H	-0.27420200	-0.60244500	3.96724800
O	-0.69222200	0.15434400	1.70338500
C	0.68445200	-2.29822600	2.35227600
H	1.13572000	-2.61889800	3.30465600
C	1.67949000	-2.51306600	1.20645100
H	1.89831300	-3.58186400	1.11314900
H	1.25055800	-2.17466200	0.26256700
H	2.62493600	-1.99506500	1.37473900
C	-0.61826300	-3.08191800	2.11973600
H	-0.40250700	-4.15528000	2.13462600
H	-1.37209200	-2.87710600	2.88740400
H	-1.02091700	-2.82012300	1.13873900
C	1.81390600	0.39709000	2.84964200
C	2.97757800	-0.15660600	3.40038600
C	1.83009600	1.71708600	2.38261800
C	4.14946100	0.59802200	3.46394800
H	2.97972200	-1.17835500	3.76747800
C	3.00274200	2.46859300	2.44202800
H	0.92529000	2.12758600	1.94888000
C	4.16443200	1.90756400	2.97726200
H	5.05067200	0.16250600	3.88444200
H	3.01336800	3.48420100	2.05888900
H	5.07969500	2.49052300	3.01971000

d-Cu-S-1

Sum of electronic and thermal Free Energies= -2394.619740

P	0.53624500	-2.01793000	-0.03738400
H	-0.05723200	-1.90168300	1.22987000
O	0.14910400	-0.93571300	-1.03724400
C	-2.79329400	2.78715000	-0.57980200
C	-0.72054600	3.67704400	-0.04426800
C	-1.26187600	4.94716900	0.15198800
C	-2.63522500	5.11768200	-0.03283300
C	-3.42023300	4.01978400	-0.39102700
H	-0.61936900	5.76620200	0.45344200
H	-3.09094900	6.09074900	0.11622900

H	-4.49288200	4.10321900	-0.51949200
N	-1.46886100	2.62079200	-0.42129000
C	0.68169900	3.31644300	0.20611000
C	2.48578600	2.05283700	0.50774900
C	2.80232800	3.53622300	0.89052800
H	2.48381600	1.40298600	1.39074900
H	3.09473000	3.66604500	1.93415700
H	3.54890400	3.99110300	0.23470600
C	-3.50965000	1.54585200	-0.91732900
C	-3.95600000	-0.56539700	-1.41846200
C	-5.27426900	0.17840400	-1.03765800
H	-3.95093000	-0.81102600	-2.48798300
H	-6.05313500	0.12093900	-1.79972500
H	-5.67098600	-0.15566100	-0.07435300
N	-2.91726800	0.44552300	-1.17987900
N	1.09811700	2.11808500	0.04833200
O	1.53971900	4.24353300	0.69532200
O	-4.87117600	1.56950800	-0.90105300
C	3.42020400	1.48331900	-0.54095700
C	4.79352400	1.42089000	-0.27604200
C	2.94396900	1.03003400	-1.77457900
C	5.68179300	0.94298800	-1.23783800
H	5.17108900	1.74525900	0.69090800
C	3.83436800	0.55181600	-2.73749700
H	1.87707900	1.03482900	-1.96258000
C	5.20422900	0.51376100	-2.47781000
H	6.74497100	0.90368300	-1.01880100
H	3.45157100	0.20572300	-3.69354600
H	5.89511200	0.14589600	-3.23102100
C	-3.71754500	-1.83905200	-0.63491400
C	-3.18889700	-1.78854800	0.66127400
C	-4.04361800	-3.07873900	-1.19366700
C	-3.00069400	-2.96760500	1.38286900
H	-2.87410000	-0.84480300	1.09539400
C	-3.86070300	-4.25714700	-0.46672000
H	-4.43674500	-3.12437300	-2.20650700
C	-3.34101500	-4.20351000	0.82756200
H	-2.57597900	-2.91549100	2.38142800
H	-4.11703400	-5.21324900	-0.91355300
H	-3.19027900	-5.11840900	1.39286000
O	-1.26757600	0.36028200	1.79599700
C	-0.39120700	0.22180400	2.72781000
O	0.84221000	0.15661300	2.56702100
C	-0.97830200	0.11868800	4.13669500

H	-1.49165000	1.05202000	4.39010000
H	-1.73007200	-0.67673100	4.17180800
H	-0.19627100	-0.07739000	4.87199900
Cu	-0.63032900	0.76277000	-0.07427700
C	2.30350600	-2.08986800	0.33594400
C	2.74407200	-2.02959300	1.66458500
C	3.23845100	-2.17923800	-0.70644400
C	4.10932100	-2.08916100	1.94503400
H	2.02792700	-1.86929100	2.46215000
C	4.59913000	-2.23972300	-0.41938800
H	2.90434200	-2.16591200	-1.73748400
C	5.03449400	-2.20074100	0.90685900
H	4.44938200	-2.03144400	2.97467400
H	5.31867200	-2.29001900	-1.22913400
H	6.09722100	-2.23868300	1.12822200
C	-0.01611100	-3.67431700	-0.60260200
H	-1.10013200	-3.54478300	-0.70500500
C	0.56709400	-4.03336800	-1.97353000
H	1.64450900	-4.21582200	-1.90896000
H	0.09706200	-4.94667900	-2.35313000
H	0.39245500	-3.23154000	-2.69615400
C	0.26138800	-4.74157100	0.46113500
H	-0.18853800	-4.47748700	1.42323100
H	-0.16285000	-5.70175900	0.14994800
H	1.33692200	-4.87825100	0.61321600

e-Cu-S-1

Sum of electronic and thermal Free Energies= -2395.547825

P	-0.11041804	-0.61377355	-2.55441049
H	-0.19922415	-0.59813901	-3.97165713
C	0.83007582	-2.15967626	-2.24323152
H	1.84704997	-1.90474007	-2.56123931
C	0.29466754	-3.29747019	-3.12246970
H	0.92393451	-4.18378249	-2.99448799
H	-0.72825504	-3.56770798	-2.84071033
H	0.29756835	-3.03827899	-4.18675423
C	0.85494375	-2.53587658	-0.75855422
H	1.89459659	-2.63002875	-0.43007447
H	0.37795832	-1.78251097	-0.13258305
H	0.35770555	-3.49574664	-0.58610138
C	-1.83876177	-0.90197821	-2.09459245
C	-2.77428706	-1.22808443	-3.08744872
C	-2.24315066	-0.80655867	-0.75299207
C	-4.10478142	-1.46648583	-2.74545175
H	-2.46556177	-1.28895460	-4.12783791

C	-3.57750424	-1.04443971	-0.42092450
H	-1.53081034	-0.53696603	0.02335325
C	-4.50470870	-1.37400817	-1.41084687
H	-4.82669700	-1.71532863	-3.51771274
H	-3.89297081	-0.95379950	0.61244108
H	-5.54225035	-1.55025736	-1.14220625
O	0.51289036	0.65077368	-2.00204223
C	1.11670900	-1.43193100	2.08876700
C	-0.82065600	-0.30710300	2.66950800
C	-1.03164400	-1.13385000	3.77285900
C	-0.11315900	-2.15176200	4.02408800
C	0.97611900	-2.30934800	3.16680900
H	-1.89897700	-0.97424500	4.40265700
H	-0.24541100	-2.81580100	4.87164300
H	1.71546000	-3.08557100	3.32061200
N	0.24512400	-0.43521800	1.84823900
C	-1.72709700	0.77287000	2.27163500
C	-2.59109200	2.44062200	1.08225700
C	-3.42163500	2.22775800	2.38566400
H	-2.00725900	3.36491400	1.10398700
H	-3.47945300	3.11479500	3.01802600
H	-4.42769000	1.84704900	2.18638800
C	2.24516600	-1.52436000	1.14826500
C	3.64287600	-1.19311000	-0.53313500
C	4.18143000	-2.34898000	0.37235000
H	3.37653700	-1.57358100	-1.52753300
H	4.27243400	-3.30665200	-0.14541100
H	5.13039200	-2.09733300	0.85222000
N	2.40205800	-0.77585400	0.12878500
N	-1.61470700	1.34523500	1.13359300
O	-2.69085800	1.20052100	3.12374000
O	3.17217600	-2.49060900	1.40547900
C	-3.41324000	2.40135300	-0.18545300
C	-2.79013700	2.21010400	-1.42106000
C	-4.80366700	2.56061700	-0.14011700
C	-3.54047000	2.19274600	-2.59850200
H	-1.71648700	2.04991100	-1.45341100
C	-5.55513900	2.54640400	-1.31432800
H	-5.29539800	2.66700600	0.82184400
C	-4.92439400	2.36498100	-2.54847100
H	-3.04463500	2.03778300	-3.55219300
H	-6.63365100	2.66579600	-1.26677300
H	-5.50986800	2.34895300	-3.46295100
C	4.63421100	-0.06045600	-0.71554300

C	4.38595600	1.22147300	-0.21892400
C	5.82054600	-0.30909700	-1.41712900
C	5.32015100	2.23970300	-0.42536700
H	3.44159500	1.44411200	0.26386600
C	6.75363800	0.70655900	-1.61657300
H	6.00882600	-1.29994300	-1.82503800
C	6.50484100	1.98827700	-1.11852500
H	5.10745300	3.23687100	-0.05209500
H	7.66839800	0.50132400	-2.16512800
H	7.22676300	2.78408800	-1.27712700
O	2.03490900	2.05733400	-0.22664000
C	1.79717600	3.32731400	-0.16734500
O	0.69625400	3.86402300	0.02022600
C	3.04602700	4.19073200	-0.35462400
H	3.67929000	4.10643200	0.53595200
H	3.63257600	3.82919800	-1.20452500
H	2.77720500	5.23828000	-0.49932600
Cu	0.68168900	0.69732700	0.17039000

f-Cu-S-1

Sum of electronic and thermal Free Energies= -2394.615741

C	-0.27274100	-3.34319400	-1.01185700
C	-2.48598200	-2.73110100	-0.72114500
C	-2.93580400	-3.98087800	-1.14673200
C	-1.99397800	-4.94683200	-1.49770900
C	-0.63988100	-4.62465800	-1.42527200
H	-4.00023100	-4.17947000	-1.18229500
H	-2.30954900	-5.93287900	-1.82115600
H	0.13176900	-5.33854700	-1.68598900
N	-1.17534200	-2.40771300	-0.65134600
C	-3.40386000	-1.67392000	-0.27549600
C	-4.20866200	0.17537200	0.64617500
C	-5.35218400	-0.58928400	-0.08811700
H	-4.34996500	0.12742500	1.73283200
H	-6.21086900	-0.81746600	0.54506400
H	-5.68556700	-0.06626600	-0.98984300
C	1.13283200	-2.91708600	-0.95625400
C	2.99476000	-1.71440500	-0.84259700
C	3.34641400	-3.23245900	-0.83937300
H	3.34212500	-1.23986200	-1.76701300
H	4.01537800	-3.53074200	-1.64932000
H	3.75671300	-3.56077200	0.11874300
N	1.51705800	-1.70164400	-0.85886000
N	-3.01456500	-0.61218800	0.31525900
O	-4.73882500	-1.84444900	-0.49366100

O	2.07185400	-3.89791500	-1.03443500
C	-4.05430900	1.62634800	0.24753300
C	-4.40007800	2.64810600	1.13507800
C	-3.54721900	1.95976000	-1.01399200
C	-4.25331900	3.98800500	0.76942700
H	-4.77731700	2.39428800	2.12239500
C	-3.40447400	3.29587400	-1.38291300
H	-3.21779100	1.17348300	-1.68328600
C	-3.75741600	4.31455200	-0.49309700
H	-4.52343700	4.77243600	1.47005400
H	-3.00615200	3.54257300	-2.36262800
H	-3.64214400	5.35522200	-0.78148100
C	3.57006900	-0.94669000	0.32733400
C	4.45358900	0.11420100	0.11274700
C	3.22467100	-1.30129600	1.63709400
C	4.99777500	0.81156600	1.19241800
H	4.70532600	0.40722900	-0.90136900
C	3.77177700	-0.60616200	2.71428100
H	2.49252800	-2.08297000	1.81669600
C	4.66092000	0.44939400	2.49652300
H	5.67837600	1.63852100	1.01178500
H	3.49075900	-0.88372700	3.72531500
H	5.08095800	0.99143800	3.33886300
O	-0.14490600	-0.25617500	2.06731200
C	0.17979100	-1.27484200	2.78943200
O	0.33521100	-2.43923900	2.38451800
C	0.39662300	-0.94271500	4.26675500
H	1.14078700	-0.14497300	4.35717100
H	-0.53423200	-0.56490500	4.70250300
H	0.72610700	-1.82371200	4.82038600
Cu	-0.57454000	-0.70810400	0.20759100
P	0.10694900	2.31748900	-0.68637900
H	-0.58962400	3.45860200	-1.15694400
O	-0.59707800	1.02612900	-1.05822100
C	1.71346000	2.43123900	-1.52994200
C	2.55552400	3.54468900	-1.41147200
C	2.08202300	1.36005200	-2.35204800
C	3.76355500	3.58246900	-2.10564600
H	2.27266100	4.38271600	-0.78067500
C	3.28421300	1.41063800	-3.05901000
H	1.42511800	0.49844400	-2.41041300
C	4.12608500	2.51694300	-2.93400200
H	4.41784700	4.44341800	-2.00803500
H	3.56555500	0.58460500	-3.70589800

H	5.06293800	2.55252900	-3.48216000
C	0.29839300	2.64664300	1.11465200
H	-0.44323200	1.97360000	1.56014000
C	1.66983600	2.21867300	1.64851800
H	2.47748000	2.84307900	1.25709900
H	1.67002900	2.30252200	2.73913100
H	1.87893800	1.17747200	1.40632800
C	-0.05548300	4.10358100	1.44435200
H	-1.06802500	4.36092200	1.11818400
H	-0.00022700	4.25607000	2.52686300
H	0.64280300	4.81088800	0.98113800

b-Cu-R-1

Sum of electronic and thermal Free Energies= -2394.619510

C	2.67064500	2.30549100	1.11342600
C	0.75482300	3.42059900	0.44073600
C	1.29028600	4.65448500	0.80518800
C	2.56912000	4.69307300	1.35998400
C	3.27602000	3.50038100	1.50597100
H	0.70933700	5.55443100	0.64149600
H	3.01265700	5.63668800	1.65874500
H	4.28582700	3.48003600	1.89771100
N	1.42617000	2.25402000	0.59278600
C	-0.55281100	3.26623600	-0.20106600
C	-2.26751600	2.30382500	-1.21161700
C	-2.42914600	3.85138100	-1.25363200
H	-1.95828600	1.92303700	-2.19402200
H	-2.58574100	4.25943600	-2.25231200
H	-3.21724500	4.19814200	-0.57715300
C	3.38155900	1.02116900	1.13232600
C	3.88533100	-1.10735900	0.81046900
C	5.18360700	-0.30525800	1.12926600
H	3.76767300	-1.93779600	1.51261500
H	5.60113400	-0.54752800	2.11177100
H	5.95307600	-0.40018600	0.36228200
N	2.81422300	-0.12103800	1.03223800
N	-1.11668400	2.12685900	-0.32171500
O	-1.15115700	4.35088000	-0.75883700
O	4.74102900	1.07745500	1.15647700
C	-3.50064000	1.56819600	-0.74518500
C	-4.55773100	1.34866300	-1.63524000
C	-3.61095400	1.12092700	0.57500100
C	-5.71832800	0.70413700	-1.20872100
H	-4.46772700	1.67632900	-2.66840300
C	-4.77226900	0.47597300	0.99957600

H	-2.76468400	1.23738900	1.24338900
C	-5.82832900	0.26827300	0.11304600
H	-6.53119900	0.53456400	-1.90893600
H	-4.84074500	0.11007300	2.01910600
H	-6.72498800	-0.24660300	0.44427000
C	3.83753000	-1.65610000	-0.60468600
C	4.35940500	-2.92814500	-0.86633600
C	3.30795500	-0.90529900	-1.65827300
C	4.36351600	-3.43938000	-2.16370000
H	4.75845700	-3.52401800	-0.04825200
C	3.30740500	-1.41756500	-2.95568600
H	2.84779300	0.05778200	-1.47396500
C	3.83666000	-2.68260400	-3.21291800
H	4.76788800	-4.42948100	-2.35338800
H	2.86562100	-0.82496900	-3.75007900
H	3.83043500	-3.08274700	-4.22260300
O	0.09438700	-0.78089200	-1.25481300
C	0.16843200	-0.35881300	-2.47680000
O	0.54981400	0.76417000	-2.83530600
C	-0.24280000	-1.40456900	-3.51265900
H	0.47759100	-2.22871400	-3.49052200
H	-1.22257200	-1.82551700	-3.26387600
H	-0.26833600	-0.97123500	-4.51394000
Cu	0.45622000	0.55305000	0.12742200
P	-1.10877200	-1.61582600	2.19456100
H	-1.61510800	-1.74910300	3.51526900
O	-0.62352900	-0.20995300	1.90259100
C	-2.51939500	-2.18754800	1.21675100
C	-3.60002000	-2.80514500	1.86041300
C	-2.54702200	-1.98602000	-0.17192800
C	-4.70253000	-3.23142300	1.12080100
H	-3.58809300	-2.94427500	2.93874900
C	-3.65697000	-2.40874000	-0.90047200
H	-1.70694600	-1.50315400	-0.66676200
C	-4.73114200	-3.02887500	-0.25979500
H	-5.54046400	-3.70732200	1.62138600
H	-3.68906000	-2.23801800	-1.97198200
H	-5.59591200	-3.34667300	-0.83482800
C	0.19552700	-2.90982300	2.16433800
H	-0.30139200	-3.80579600	2.56532300
C	1.32157400	-2.47711400	3.11549600
H	2.10109700	-3.24625000	3.14397500
H	1.76159400	-1.53907500	2.76838600
H	0.95872100	-2.33083200	4.13884400

C	0.70504300	-3.20088300	0.74678700
H	1.48577700	-3.96799000	0.78974200
H	-0.09642600	-3.56652100	0.10047100
H	1.12008000	-2.31017300	0.27462200

c-Cu-R-1

Sum of electronic and thermal Free Energies= -2394.619338

C	0.08711300	3.60066600	-0.73161800
C	2.32910800	3.08379900	-0.45736300
C	2.71758400	4.39464700	-0.73059100
C	1.72688700	5.33713800	-1.00410700
C	0.39009100	4.93754200	-1.00217300
H	3.76969200	4.65439200	-0.71990400
H	1.99130200	6.36788700	-1.21464600
H	-0.41348500	5.63511000	-1.20402900
N	1.03477700	2.68748500	-0.45021700
C	3.26006100	2.00314400	-0.12121100
C	3.96894300	-0.00360400	0.52129500
C	5.18514700	0.94845000	0.29668500
H	3.79677700	-0.21613300	1.58200400
H	5.83792200	1.04968300	1.16462900
H	5.77353900	0.66891200	-0.58227000
C	-1.29677700	3.09779800	-0.72668800
C	-3.09465100	1.81043200	-0.72575300
C	-3.52103300	3.30362500	-0.60772100
H	-3.36414300	1.41240800	-1.71281900
H	-4.23773300	3.62546600	-1.36508400
H	-3.90255200	3.54190600	0.38979900
N	-1.62582900	1.86640000	-0.65889400
N	2.83832700	0.81213400	0.07723800
O	4.58245800	2.25544500	0.03666000
O	-2.28396100	4.03710600	-0.80785300
C	4.07047000	-1.31739000	-0.21969500
C	4.86000300	-2.34548600	0.30748300
C	3.39321700	-1.52556700	-1.42474800
C	4.98416700	-3.56048900	-0.36538400
H	5.36429900	-2.19953500	1.25943300
C	3.51646300	-2.74211700	-2.09732300
H	2.73496200	-0.75304700	-1.80282300
C	4.31269400	-3.76135200	-1.57300600
H	5.59451300	-4.35273500	0.05804700
H	2.97897500	-2.89572900	-3.02867200
H	4.40141300	-4.70945000	-2.09518700
C	-3.71818200	0.91800500	0.32366600
C	-5.01506100	0.43247000	0.12531900

C	-3.03435800	0.59428600	1.49982800
C	-5.62772500	-0.36533900	1.09007300
H	-5.53920200	0.65977000	-0.79943200
C	-3.64565200	-0.21695800	2.45755500
H	-2.01567900	0.93040200	1.66214300
C	-4.94166900	-0.69573300	2.26023100
H	-6.63223600	-0.74205700	0.92057800
H	-3.09747800	-0.47098100	3.36022800
H	-5.41399700	-1.32482100	3.00941300
O	0.02652000	0.54733600	2.14077700
C	0.69587300	-0.21581200	2.93963000
O	1.75997600	-0.79858800	2.67793700
C	0.02773300	-0.43687300	4.29857800
H	-0.48010100	0.46900000	4.63861200
H	-0.73062800	-1.22292400	4.19379200
H	0.75850900	-0.76635600	5.03944400
Cu	0.71310300	0.79194000	0.30168400
P	-0.73088100	-1.91219200	-0.03769800
H	-1.04635300	-1.49277700	1.26902200
O	0.22244100	-0.99303700	-0.78155400
C	-2.32802400	-2.02483600	-0.89093700
C	-3.39884700	-2.76249300	-0.37328100
C	-2.46389000	-1.35705600	-2.11365000
C	-4.59661500	-2.83938900	-1.08083100
H	-3.30955900	-3.25998100	0.58824200
C	-3.66507300	-1.43522100	-2.81797900
H	-1.62572700	-0.77483800	-2.48217300
C	-4.72927000	-2.17879900	-2.30393200
H	-5.42968700	-3.40384800	-0.67371900
H	-3.77087600	-0.91847900	-3.76755200
H	-5.66456600	-2.23960900	-2.85269000
C	-0.10594200	-3.62211500	0.17953000
H	-0.88322000	-4.13794600	0.76305600
C	1.20829800	-3.59344800	0.97567200
H	1.51464800	-4.61736600	1.21440900
H	1.99789100	-3.13005800	0.38247900
H	1.13191700	-3.01865800	1.90207700
C	0.05986200	-4.31531500	-1.17928100
H	0.42462100	-5.33724800	-1.03464400
H	-0.88210200	-4.36159500	-1.73363000
H	0.79549000	-3.78006200	-1.78576000

d-Cu-R-1

Sum of electronic and thermal Free Energies= -2394.618117

C	0.37917900	3.21193800	1.04384600

C	-1.81113300	3.12322700	0.30571500
C	-2.05155500	4.43444200	0.71362500
C	-1.01887400	5.15380500	1.30922900
C	0.21428000	4.53036600	1.47749000
H	-3.03363800	4.86302800	0.55561400
H	-1.17149200	6.17649900	1.63633600
H	1.05359700	5.04197100	1.93093600
N	-0.60993800	2.50784800	0.44876200
C	-2.85936000	2.31409700	-0.32352500
C	-3.94090100	0.63328400	-1.27722400
C	-4.89997900	1.85364200	-1.11446400
H	-3.69033600	0.47977700	-2.33514100
H	-5.36319800	2.18442300	-2.04499700
H	-5.67023100	1.67231600	-0.35872000
C	1.67855900	2.54148400	1.20341300
C	3.36086300	1.10108100	1.21882600
C	3.87090800	2.50073300	1.68070500
H	3.54303300	0.35249300	1.99683200
H	4.16161000	2.52329200	2.73561400
H	4.68303000	2.88705300	1.06316700
N	1.90556200	1.29049600	1.07276800
N	-2.72032800	1.07546300	-0.59661000
O	-4.03581800	2.92220700	-0.63709700
O	2.72163900	3.36438000	1.51081500
C	-4.49877800	-0.65725400	-0.71639800
C	-5.58439700	-1.27099800	-1.35205400
C	-3.96268300	-1.23350400	0.43934200
C	-6.13753200	-2.44219200	-0.83584100
H	-5.99553600	-0.83553100	-2.26017800
C	-4.51739900	-2.40815800	0.95152100
H	-3.09921700	-0.77588600	0.90987100
C	-5.60460000	-3.01422300	0.32109600
H	-6.97904600	-2.90997800	-1.33840400
H	-4.09638000	-2.85092500	1.85024400
H	-6.03223300	-3.92783800	0.72356900
C	4.00327000	0.62910100	-0.07177800
C	5.20139300	-0.09361500	-0.02564900
C	3.43501100	0.93886400	-1.30997200
C	5.83382400	-0.48855900	-1.20475800
H	5.63805800	-0.35372300	0.93614500
C	4.05976700	0.53042800	-2.48926200
H	2.48303800	1.45246300	-1.38049600
C	5.26264600	-0.17608700	-2.44173300
H	6.76263700	-1.04972300	-1.15778900

H	3.57874600	0.76398200	-3.43307700
H	5.74856400	-0.49293400	-3.36001200
O	0.03814300	-0.45618300	-1.76727800
C	0.57086100	0.11388200	-2.79596500
O	0.84195900	1.32086900	-2.90662500
C	0.92085400	-0.85787900	-3.92203800
H	1.77102500	-1.47067200	-3.60213200
H	0.08772800	-1.53929300	-4.11483800
H	1.18766800	-0.31866800	-4.83289700
Cu	-0.42012400	0.66222800	-0.26638200
P	-0.40633500	-2.17157000	1.61798200
H	-1.46173400	-3.12024100	1.63220700
O	-0.85898400	-0.74457300	1.39244400
C	0.73628300	-2.87824700	0.41632000
C	0.61361400	-4.22026000	0.03146400
C	1.77702400	-2.09255400	-0.09527200
C	1.53931300	-4.77764900	-0.84921200
H	-0.20621700	-4.82551000	0.40944000
C	2.70227900	-2.65459300	-0.97224700
H	1.83868400	-1.04440400	0.16469500
C	2.58351200	-3.99426500	-1.34641300
H	1.44128100	-5.81542200	-1.15305600
H	3.50086700	-2.04053500	-1.37117100
H	3.30149900	-4.42794600	-2.03654900
C	0.36646000	-2.37727200	3.27608300
H	-0.39634700	-1.99797300	3.96948100
C	1.61098700	-1.48371800	3.37300300
H	2.00145000	-1.48973900	4.39589700
H	2.39852400	-1.85312400	2.70788300
H	1.37896000	-0.45486800	3.08572900
C	0.66504100	-3.84629500	3.59468700
H	1.06851000	-3.93825500	4.60823900
H	-0.23379300	-4.46939100	3.53614500
H	1.40449100	-4.25431900	2.89891200

e-Cu-R-1

Sum of electronic and thermal Free Energies= -2394.617018

P	-0.11414500	-2.33038000	-1.27868100
H	0.40383100	-3.10454500	-2.34908800
O	-0.10599400	-0.84554700	-1.55669200
C	1.06458400	1.08224600	2.50127600
C	-1.17765000	1.62281400	2.25401000
C	-1.25564000	2.07961500	3.56796500
C	-0.11564600	2.01205400	4.37126300
C	1.06582300	1.50436900	3.83132100

H	-2.19296000	2.47778300	3.93830100
H	-0.14630800	2.35744000	5.39906500
H	1.98076000	1.44559600	4.40878100
N	-0.03540700	1.13793100	1.71899400
C	-2.29408500	1.66371600	1.30014200
C	-3.46068000	1.39183000	-0.56237400
C	-4.03693100	2.58164500	0.25517400
H	-3.06261000	1.75700200	-1.51147500
H	-3.81280900	3.53238400	-0.22696700
H	-5.09881300	2.49522800	0.49239400
C	2.25025100	0.55979700	1.81659700
C	3.57056600	-0.31131200	0.26895900
C	4.38343700	-0.07019000	1.57789100
H	3.42497500	-1.38899500	0.11618400
H	4.86975200	-0.96525300	1.96840500
H	5.11308500	0.73730100	1.46912600
N	2.25671300	0.25924700	0.57605400
N	-2.30425300	0.94846800	0.24103500
O	-3.28901600	2.55315000	1.50922500
O	3.37884900	0.34894000	2.54585100
C	-4.39126400	0.22258000	-0.80655400
C	-4.34509600	-0.45707800	-2.02736900
C	-5.23997500	-0.25222600	0.19986500
C	-5.13180300	-1.59013000	-2.24186300
H	-3.67649900	-0.10001600	-2.80578100
C	-6.03218500	-1.37982100	-0.01156600
H	-5.26866500	0.25362400	1.16119100
C	-5.97836900	-2.05443700	-1.23376900
H	-5.08725700	-2.10656000	-3.19650600
H	-6.68786500	-1.73586200	0.77775400
H	-6.59519300	-2.93267300	-1.39970600
C	4.20053200	0.27446700	-0.97712300
C	3.53413700	1.22411500	-1.75880900
C	5.47881300	-0.15660400	-1.35513300
C	4.15237600	1.73416000	-2.90287300
H	2.53661200	1.56021100	-1.49598700
C	6.09331800	0.36000000	-2.49451900
H	5.99577100	-0.90553000	-0.75814600
C	5.42851000	1.31025000	-3.27299200
H	3.62212100	2.46666800	-3.50408100
H	7.08580600	0.01952100	-2.77578500
H	5.90297700	1.71260000	-4.16338100
O	0.37344800	2.32812700	-1.59469100
C	-0.38442600	3.37233000	-1.58533000

O	-1.35259300	3.57871200	-0.83383700
C	0.00459600	4.42159800	-2.62951100
H	1.02638600	4.76655700	-2.43990300
H	-0.00170400	3.97255700	-3.62786600
H	-0.67929600	5.27152200	-2.60107900
Cu	0.05847000	0.90277600	-0.29634200
C	-1.78223200	-3.04929100	-1.01195900
H	-2.36935200	-2.64698900	-1.84739200
C	-2.40797000	-2.54641900	0.29526000
H	-3.46505100	-2.82433700	0.32243800
H	-1.91315300	-2.99895000	1.16085300
H	-2.34650800	-1.45873000	0.37447300
C	-1.74473000	-4.58016700	-1.09510200
H	-1.13000000	-5.00220600	-0.29287400
H	-2.75707800	-4.98113100	-0.98472800
H	-1.34661400	-4.93471000	-2.05158900
C	0.94762400	-2.87380400	0.08959000
C	1.94703700	-3.83106200	-0.12913700
C	0.80856500	-2.29619700	1.36074300
C	2.79887400	-4.20915700	0.90986600
H	2.07016800	-4.27167000	-1.11459700
C	1.66334800	-2.67151700	2.39491100
H	0.06044100	-1.52958500	1.52451200
C	2.65941800	-3.62545300	2.17037500
H	3.57423100	-4.94809000	0.73287600
H	1.56457500	-2.20605500	3.37043500
H	3.32710100	-3.91181400	2.97747100

Cu-I-1

Sum of electronic and thermal Free Energies = -1951.848867

O	-1.45431000	0.54023000	2.20422600
C	-1.88853700	-0.36792300	3.01634100
O	-1.67642700	-1.59058100	2.94173900
C	-2.80332600	0.16602000	4.11741100
H	-2.87563200	-0.55045400	4.93757500
H	-2.45230300	1.13407700	4.48275300
H	-3.80500700	0.31460900	3.69694400
Cu	-0.25048200	0.10117000	0.71637500
C	2.67202300	0.18234200	-2.92558800
C	1.33521000	-1.17781300	-1.64657100
C	1.59923700	-2.27899600	-2.48048300
C	2.43960500	-2.10927300	-3.57313600
C	2.99623400	-0.85103700	-3.80516800
H	3.08430100	1.17878300	-3.07553600
H	1.14921900	-3.23814700	-2.25280400

H	2.66009700	-2.94463000	-4.23104300
H	3.65867800	-0.67063800	-4.64536500
N	1.86456800	0.04152000	-1.87199800
C	0.48346000	-1.35110300	-0.50062800
C	-0.20566800	-1.88774800	0.37902200
H	-0.77739800	-2.37403900	1.14950700
C	1.21224700	2.65840100	0.23020200
C	-0.89351000	2.70443900	-0.70616600
C	-0.75049500	4.04999900	-1.06203200
C	0.44680300	4.69593900	-0.77047400
C	1.45398000	3.98769800	-0.11978100
H	-1.56592300	4.55457500	-1.56537300
H	0.59249600	5.73580000	-1.04387900
H	2.40343100	4.44163700	0.13576400
N	0.06307500	2.02342900	-0.05957000
C	-2.11143100	1.95651700	-1.07977200
C	-3.53960600	0.40291800	-1.73890300
C	-4.30740800	1.71968800	-1.41923100
H	-3.54508200	0.20992100	-2.81935300
H	-4.93085600	2.08378000	-2.23744500
H	-4.90207900	1.63359000	-0.50550600
C	2.20008200	1.86474700	0.97575100
C	3.20860200	0.22908300	2.07939900
C	4.15580700	1.45196600	1.97376800
H	3.05765400	-0.06375200	3.12159500
H	4.26976300	1.97048000	2.93035100
H	5.13767200	1.22573900	1.55837600
N	1.93256600	0.75801000	1.54795900
N	-2.14871100	0.71380800	-1.35293900
O	-3.25410300	2.69341900	-1.17899500
O	3.46929900	2.35199300	1.05835300
C	-4.08635600	-0.81385000	-1.02821100
C	-3.70397100	-1.08467500	0.28892600
C	-4.98880700	-1.66579500	-1.67131200
C	-4.20460300	-2.20377000	0.95353600
H	-2.99546600	-0.43144600	0.78299500
C	-5.50782800	-2.77505800	-1.00144200
H	-5.28042400	-1.46716700	-2.70030100
C	-5.11265400	-3.04748000	0.30985300
H	-3.85379400	-2.40985100	1.95956900
H	-6.20872100	-3.43240900	-1.50816200
H	-5.50424000	-3.92060100	0.82397400
C	3.61180300	-0.99230300	1.27411500
C	3.20895200	-2.25957100	1.70373900

C	4.30585800	-0.87236800	0.06529200
C	3.50367400	-3.39176200	0.94502200
H	2.64452600	-2.35515900	2.62694800
C	4.61141300	-2.00290100	-0.68962600
H	4.58541000	0.10817200	-0.30650500
C	4.20959700	-3.26553100	-0.25195900
H	3.17958400	-4.36962600	1.28777700
H	5.13956100	-1.89409300	-1.63125400
H	4.44004000	-4.14573700	-0.84458300

TS1A

Sum of electronic and thermal Free Energies = -1951.837568

O	-1.50734600	0.19448100	2.24429400
C	-1.65494000	-0.90750900	2.78445500
O	-1.04892700	-2.00912700	2.40633400
C	-2.60874000	-1.09902600	3.93722700
H	-2.21343500	-1.82424600	4.65147900
H	-2.80598600	-0.14271300	4.42201100
H	-3.54781700	-1.49519400	3.53584800
Cu	-0.30447300	0.31631400	0.41676400
C	3.24508700	-1.07182800	-3.64294400
C	1.89543400	-1.95545400	-1.99755200
C	2.32405200	-3.26232400	-2.31447200
C	3.25257000	-3.44281400	-3.32975300
C	3.73350600	-2.32463100	-4.01560700
H	3.59301800	-0.17653800	-4.15648200
H	1.92946600	-4.09975100	-1.75043900
H	3.60044600	-4.44029500	-3.58305000
H	4.46171100	-2.41752400	-4.81442500
N	2.35568700	-0.86913900	-2.66687000
C	0.99027900	-1.73274300	-0.92566300
C	0.25278500	-1.54101000	0.04891500
H	-0.51369300	-1.83293700	1.52629800
C	0.59072400	3.10340100	-0.02317700
C	-1.43355400	2.68505100	-1.05133100
C	-1.51842100	4.00036700	-1.51369000
C	-0.48396600	4.88458200	-1.21719600
C	0.59130800	4.43007900	-0.45822500
H	-2.38066300	4.30169200	-2.09520300
H	-0.51776500	5.91059300	-1.56769000
H	1.41993300	5.07458700	-0.19238500
N	-0.39455000	2.23233400	-0.32140200
C	-2.48321100	1.69998800	-1.35127600
C	-3.64484600	-0.16610500	-1.57509400
C	-4.51333300	1.04062100	-2.01009300

H	-3.31925100	-0.72922900	-2.46245200
H	-4.95677000	0.94511800	-3.00166400
H	-5.28877200	1.28148900	-1.27569700
C	1.67223700	2.56410100	0.81269300
C	2.89603600	1.23576700	2.10200500
C	3.65935400	2.57774200	1.84612400
H	2.65453100	1.12587700	3.16461000
H	3.85123100	3.15128300	2.75593600
H	4.59104000	2.42990200	1.29633100
N	1.63290000	1.41497000	1.36631100
N	-2.43478500	0.47220900	-1.01931500
O	-3.57203500	2.14830300	-2.04036100
O	2.75996300	3.35299900	1.00668000
C	-4.26814200	-1.14230200	-0.59867100
C	-3.42057300	-1.96761200	0.15061000
C	-5.65188400	-1.27319200	-0.45289400
C	-3.95097500	-2.89216500	1.04774100
H	-2.34697200	-1.87520300	0.03087700
C	-6.18375500	-2.20416300	0.44305600
H	-6.32956200	-0.65703100	-1.03723400
C	-5.33519100	-3.01152400	1.19955200
H	-3.27371800	-3.50627200	1.63261300
H	-7.26097300	-2.29375100	0.54865900
H	-5.74893100	-3.73184500	1.89925900
C	3.67690800	0.01856000	1.65525800
C	4.34574000	-0.76100500	2.60311900
C	3.79280400	-0.30017300	0.29819600
C	5.13318000	-1.84125900	2.20318800
H	4.24643900	-0.52383800	3.65967000
C	4.58065400	-1.37821100	-0.10190800
H	3.24140300	0.26262100	-0.44754600
C	5.25452800	-2.14827700	0.84778000
H	5.64474900	-2.44288200	2.94874600
H	4.65492200	-1.62371000	-1.15586900
H	5.86321900	-2.99077800	0.53273700

Int1A

Sum of electronic and thermal Free Energies = -1951.838619

C	-0.36379800	-3.64822800	-0.50333700
C	1.84240400	-3.07644800	-0.88417000
C	2.19217600	-4.40657400	-1.12244400
C	1.19806700	-5.38038500	-1.07697600
C	-0.10125700	-4.99681800	-0.76167700
H	3.22353300	-4.64957700	-1.34472200
H	1.43475000	-6.42077400	-1.27282200

H	-0.91077000	-5.71263900	-0.69529500
N	0.58118500	-2.69070500	-0.59344700
C	2.85472500	-2.00865400	-0.94400100
C	3.88047600	-0.05068400	-1.01894200
C	4.93066300	-1.18286100	-0.86336300
H	3.92499800	0.36295600	-2.03722000
H	5.72081400	-1.17140200	-1.61486300
H	5.36916100	-1.20965500	0.13894700
C	-1.70148800	-3.22225500	-0.05807600
C	-3.37676000	-2.19685500	0.95143500
C	-3.91965200	-3.39022300	0.11903800
H	-3.40200300	-2.44673000	2.02154300
H	-4.55330200	-4.08020200	0.67744100
H	-4.43833800	-3.05041000	-0.78268800
N	-1.95038600	-2.14094300	0.57050300
N	2.59492500	-0.76441400	-0.90687400
O	4.15690800	-2.40162400	-1.04024100
O	-2.71784500	-4.10149500	-0.28911200
C	3.97310200	1.10091100	-0.03608700
C	5.17818600	1.45297000	0.57831700
C	2.82438600	1.85743300	0.22993200
C	5.23386000	2.53422700	1.46160200
H	6.08632000	0.89276400	0.37325800
C	2.87935000	2.93224800	1.11572100
H	1.88659300	1.59590000	-0.24747400
C	4.08385100	3.27322900	1.73741600
H	6.17653400	2.79398600	1.93420100
H	1.97132000	3.48751300	1.32895800
H	4.12578500	4.10984400	2.42863300
C	-4.14490700	-0.91257100	0.74390500
C	-3.65034600	0.11060300	-0.06680000
C	-5.39259300	-0.76018300	1.36214200
C	-4.38454200	1.28456000	-0.24971800
H	-2.67435900	0.01532000	-0.52534700
C	-6.13737800	0.40156400	1.16421100
H	-5.77773000	-1.54707100	2.00771800
C	-5.63138600	1.42679200	0.36074000
H	-3.95825300	2.08579600	-0.84851400
H	-7.10396100	0.51193000	1.64784100
H	-6.20501700	2.33888300	0.22158400
O	0.94011500	-0.57858800	1.82269700
C	0.78665400	0.46584200	2.44739700
O	0.04480500	1.48556200	2.03375800
C	1.46805900	0.74147400	3.76195400

H	1.80488300	-0.19361600	4.20867600
H	2.33564500	1.38016000	3.56367900
H	0.80152200	1.27885400	4.43970800
Cu	0.12568300	-0.73637900	-0.31826400
C	-2.66741400	5.14237100	-1.70008700
C	-1.09861600	3.51046900	-1.27122500
C	-0.07053900	4.47672000	-1.23327300
C	-0.38679900	5.81324800	-1.43745000
C	-1.71657800	6.16306100	-1.67817500
H	-3.71488400	5.37636700	-1.88455800
H	0.94886000	4.15723400	-1.04621200
H	0.39066900	6.57156400	-1.41085600
H	-2.01171300	7.19392000	-1.84389600
N	-2.38839800	3.85131000	-1.50480800
C	-0.78793900	2.13712500	-1.04731500
C	-0.47314600	0.96560700	-0.82851000
H	-0.27392600	1.32020200	1.10442900

S-1

Sum of electronic and thermal Free Energies = -767.008503

P	-1.14038600	-0.54090200	-0.80883200
O	-1.52583600	-1.99040900	-0.77419700
H	-1.25585600	0.11665100	-2.06641700
C	0.59602800	-0.21877300	-0.37739000
C	1.28719700	0.89737600	-0.86527800
C	1.24144500	-1.11141200	0.48786000
C	2.60563000	1.12950700	-0.47586100
H	0.80082400	1.58023100	-1.55642700
C	2.55973800	-0.87699700	0.87570300
H	0.70739500	-1.99148200	0.83188500
C	3.24001300	0.24454700	0.39790400
H	3.13956100	1.99393500	-0.85829500
H	3.05873400	-1.57134800	1.54458700
H	4.26763100	0.42448400	0.69858800
C	-2.15839200	0.53887300	0.28581600
H	-3.18404400	0.36432000	-0.06680700
C	-2.04633100	0.05328700	1.73634300
H	-1.02853300	0.19388000	2.11524600
H	-2.72631900	0.61925900	2.38092600
H	-2.29726900	-1.00751000	1.81411900
C	-1.81882500	2.02507800	0.13262900
H	-2.51239700	2.63208900	0.72314800
H	-0.80554500	2.23560300	0.48755400
H	-1.89072100	2.35776800	-0.90840600

D-S-1

Sum of electronic and thermal Free Energies = -1534.009419

P	-1.75769400	-1.36785500	0.19466600
O	-0.88163600	-1.62136300	1.39603100
H	-1.53368900	-2.18219600	-0.93520700
P	1.75814500	-1.36763800	-0.19486700
O	0.88208500	-1.62082100	-1.39630800
H	1.53416600	-2.18225300	0.93476400
C	-1.64388300	0.30637200	-0.49616900
C	-1.46722800	0.50124100	-1.87053100
C	-1.73125000	1.41473100	0.35663800
C	-1.41329400	1.79362600	-2.39091100
H	-1.33318200	-0.35660400	-2.52021700
C	-1.67679100	2.70441800	-0.16630400
H	-1.80554600	1.26454900	1.42819600
C	-1.52666500	2.89476700	-1.54095800
H	-1.27211200	1.94128400	-3.45732700
H	-1.73451100	3.55985300	0.49917400
H	-1.48141400	3.90084100	-1.94739400
C	-3.54904100	-1.66098000	0.52711800
H	-3.58359200	-2.72342000	0.80571000
C	-4.03819300	-0.82950900	1.71769200
H	-4.05364100	0.23601500	1.46737500
H	-5.05708100	-1.12081700	1.99329300
H	-3.39019100	-0.97077000	2.58665100
C	-4.39625300	-1.44354200	-0.73196800
H	-5.43842600	-1.72027200	-0.54219100
H	-4.37662300	-0.39365100	-1.03979000
H	-4.03921000	-2.04658700	-1.57347700
C	1.64395300	0.30644300	0.49629300
C	1.46777400	0.50110900	1.87073100
C	1.73032900	1.41492600	-0.35646400
C	1.41330100	1.79342600	2.39124600
H	1.33456300	-0.35684100	2.52045600
C	1.67534600	2.70453200	0.16659600
H	1.80431400	1.26482800	-1.42805300
C	1.52565700	2.89468900	1.54133100
H	1.27248300	1.94090300	3.45773500
H	1.73226800	3.56005500	-0.49883300
H	1.47995900	3.90071300	1.94784400
C	3.54952400	-1.66033000	-0.52746700
H	3.58437700	-2.72262300	-0.80655300
C	4.03833500	-0.82810700	-1.71767100
H	4.05319000	0.23732200	-1.46689800
H	5.05739000	-1.11876500	-1.99333700

H	3.39044800	-0.96933600	-2.58671800
C	4.39671900	-1.44322900	0.73169100
H	4.03991700	-2.04683600	1.57290100
H	5.43897600	-1.71951100	0.54172200
H	4.37675900	-0.39350200	1.04005400

D-S-1-TS1

Sum of electronic and thermal Free Energies = -1533.965146

P	-1.66568000	0.83434500	-0.00665700
O	-0.89349300	0.93857800	-1.38494600
H	-0.55627100	0.85216400	1.02671900
P	1.64664500	0.86253000	-0.03423800
O	0.91028400	0.96494500	1.35556500
H	0.27211100	0.90085700	-1.12877800
C	-2.67282600	-0.67403000	-0.01244100
C	-3.16685600	-1.21211100	1.18400600
C	-2.95390600	-1.30675400	-1.23031400
C	-3.95525800	-2.36104200	1.15798000
H	-2.92467900	-0.74403800	2.13381000
C	-3.74120200	-2.45685300	-1.25054400
H	-2.53753600	-0.89755700	-2.14491000
C	-4.24544900	-2.98096700	-0.05902200
H	-4.33458000	-2.77768900	2.08575900
H	-3.95717900	-2.94727400	-2.19467300
H	-4.85557400	-3.87876600	-0.07686800
C	-2.83810200	2.25324000	0.09807200
H	-2.17209200	3.12623700	0.10345400
C	-3.73078900	2.31159000	-1.14680600
H	-4.40142400	1.44718100	-1.18461100
H	-4.34681900	3.21632400	-1.12706900
H	-3.13011200	2.31991200	-2.05958300
C	-3.64098900	2.22649900	1.40383600
H	-4.25164200	3.13108900	1.48458500
H	-4.31598200	1.36579400	1.43158300
H	-2.99096400	2.18196900	2.28367500
C	2.65189300	-0.67325800	-0.01676500
C	3.20231900	-1.21008100	-1.18845000
C	2.84663100	-1.34052800	1.19930400
C	3.96031100	-2.37963800	-1.14044100
H	3.02892700	-0.71891300	-2.14233200
C	3.60193500	-2.51178800	1.24652900
H	2.38686400	-0.92748000	2.09192400
C	4.16395000	-3.03019500	0.07827300
H	4.38523000	-2.78819300	-2.05276400
H	3.75133000	-3.02296900	2.19342600

H	4.75024700	-3.94366700	0.11477400
C	2.93943900	2.20756400	-0.07457000
H	2.33701600	3.12652700	-0.07286700
C	3.78260200	2.17278200	-1.35203200
H	4.44252300	3.04595800	-1.39932800
H	4.41355800	1.27912400	-1.37944100
H	3.15888700	2.17480300	-2.25270600
C	3.79207100	2.17996600	1.19740900
H	4.42479500	1.28663400	1.21935100
H	4.44537700	3.05861500	1.24608300
H	3.15454800	2.16565100	2.08511600

D-S-2

Sum of electronic and thermal Free Energies = -1534.001259

P	1.75557300	1.44746200	-0.06228900
O	1.16294900	1.66970900	1.48330600
H	-0.20881500	1.86526900	-1.41624100
P	-1.75777600	1.44688400	0.06219700
O	-1.16522500	1.66995600	-1.48333500
H	0.20659300	1.86528800	1.41634000
C	1.81943800	-0.38533500	-0.18975700
C	2.08066600	-0.97229000	-1.43548000
C	1.55814700	-1.20942200	0.91193500
C	2.11786500	-2.35974000	-1.56976900
H	2.24471100	-0.34292900	-2.30676700
C	1.58075600	-2.59544400	0.77337000
H	1.32047400	-0.75052300	1.86505800
C	1.86898800	-3.17329700	-0.46396900
H	2.32733600	-2.80470300	-2.53806200
H	1.36232500	-3.22617700	1.62940800
H	1.88298400	-4.25388700	-0.56974800
C	3.54321800	1.87798400	0.27264200
H	3.50709500	2.95830800	0.46592900
C	4.39217900	1.62841800	-0.97914300
H	5.41548000	1.98293800	-0.81764100
H	4.44440200	0.55950200	-1.20870400
H	3.99178200	2.14603800	-1.85725100
C	4.11033200	1.17037400	1.50608900
H	4.16654600	0.08997700	1.34203000
H	5.12248700	1.53364900	1.71772000
H	3.48751600	1.35024800	2.38505000
C	-3.54594300	1.87489300	-0.27312100
H	-3.51105800	2.95505400	-0.46751400
C	-4.11228900	1.16547100	-1.50584900
H	-4.16734600	0.08516800	-1.34077700

H	-5.12483500	1.52744300	-1.71784700
H	-3.48968000	1.34516800	-2.38500100
C	-4.39447100	1.62544000	0.97899800
H	-5.41830000	1.97830100	0.81722000
H	-4.44513100	0.55667800	1.20964900
H	-3.99472300	2.14454200	1.85652000
C	-1.81900600	-0.38601300	0.18981700
C	-2.07936300	-0.97332600	1.43554500
C	-1.55639100	-1.20975100	-0.91183600
C	-2.11441100	-2.36082600	1.56987600
H	-2.24441600	-0.34419200	2.30680800
C	-1.57681600	-2.59579900	-0.77322900
H	-1.31945200	-0.75051700	-1.86498000
C	-1.86418800	-3.17403900	0.46412800
H	-2.32323200	-2.80608400	2.53817400
H	-1.35730800	-3.22621100	-1.62922100
H	-1.87645800	-4.25464300	0.56997200

S-2

Sum of electronic and thermal Free Energies = -767.005145

H	-1.60605500	-2.50641500	-1.27726100
P	-1.17923400	-0.36550400	-1.02600900
O	-1.51540300	-1.93660900	-0.50369400
C	-2.10079800	0.54252500	0.33404700
H	-3.15338500	0.34772700	0.08757600
C	-1.81701000	0.01222100	1.74106000
H	-0.78128000	0.21200700	2.03158200
H	-2.47215800	0.50331900	2.46991300
H	-1.98621700	-1.06555500	1.79884700
C	-1.84479300	2.05018000	0.22367900
H	-2.47617100	2.59442100	0.93389200
H	-0.80220600	2.28730400	0.45826600
H	-2.06220700	2.43117700	-0.77986100
C	0.55736300	-0.17689000	-0.44111200
C	1.29552500	0.93514600	-0.87135100
C	1.16886200	-1.11282900	0.40379500
C	2.60831900	1.12522000	-0.44055100
H	0.84363700	1.65183900	-1.55263800
C	2.48578600	-0.92907700	0.82362100
H	0.60278300	-1.98225100	0.72075700
C	3.20596300	0.19259200	0.40832500
H	3.16779100	1.99345100	-0.77602900
H	2.95087900	-1.66050600	1.47833000
H	4.23101600	0.33438700	0.73723100

Cu-S-2

Sum of electronic and thermal Free Energies = -2394.642884

C	0.26525800	3.14573000	-0.03943700
C	-1.88954800	2.85722000	-0.83109200
C	-2.17748700	4.21387300	-0.67412900
C	-1.18273500	5.05136400	-0.17068100
C	0.05986800	4.51280800	0.15335800
H	-3.16144500	4.58352800	-0.93460900
H	-1.37617700	6.10962300	-0.03095800
H	0.86205200	5.11830300	0.55684900
N	-0.69764900	2.32868600	-0.50245700
C	-2.88152300	1.89107100	-1.32387200
C	-3.88317800	0.01265500	-1.92102900
C	-4.92402200	1.17262000	-1.87697600
H	-3.87262400	-0.46227100	-2.90588800
H	-5.27771800	1.46687700	-2.86970300
H	-5.77081400	0.97015100	-1.22091800
C	1.52659600	2.47016300	0.29080100
C	3.06576600	0.90356100	0.52270800
C	3.36288800	2.09298000	1.49090700
H	2.84015800	0.00005900	1.09704100
H	3.19517900	1.82909100	2.53563100
H	4.35086200	2.53487000	1.35120900
N	1.82356100	1.30958600	-0.15490800
N	-2.59519400	0.70387800	-1.70051400
O	-4.17865700	2.28980200	-1.31822000
O	2.36468900	3.09474000	1.13965500
C	-4.11404100	-1.04786600	-0.86309300
C	-4.68639300	-2.27328000	-1.21855100
C	-3.77220000	-0.81307600	0.47332300
C	-4.91594400	-3.25194600	-0.25058200
H	-4.93893500	-2.46769600	-2.25802400
C	-3.98881600	-1.79464100	1.43865600
H	-3.28275400	0.10653100	0.77199300
C	-4.56451600	-3.01416500	1.07949500
H	-5.35104700	-4.20426300	-0.53866200
H	-3.66751600	-1.60964500	2.45674400
H	-4.71968500	-3.78418000	1.82878100
C	4.19126700	0.59346200	-0.44199700
C	5.41054600	0.13016000	0.06581400
C	4.03700700	0.72571600	-1.82295600
C	6.45709200	-0.19983400	-0.79167800
H	5.52942100	-0.00227200	1.13818200
C	5.08261300	0.38928100	-2.68514800
H	3.08740300	1.07305300	-2.21464700

C	6.29455500	-0.07492200	-2.17372500
H	7.39508300	-0.56521500	-0.38400700
H	4.94795300	0.48872400	-3.75845100
H	7.10679000	-0.33800600	-2.84473900
Cu	-0.33847400	0.30026800	-0.43654100
O	-1.64758100	0.27839000	2.73047500
C	-0.80115300	1.27668900	2.74289300
O	0.40262000	1.19529400	2.49907300
C	-1.45963900	2.61159500	3.05408400
H	-2.11113900	2.52438800	3.92721200
H	-0.70006200	3.37607500	3.22062300
H	-2.09010900	2.90747000	2.20811200
C	2.05928600	-2.82748600	1.60256900
C	3.38595100	-3.26232300	1.61795500
C	4.16714900	-3.17453200	0.46351200
C	3.62309100	-2.63909200	-0.70551000
C	2.30259300	-2.19314100	-0.71699700
C	1.50513100	-2.29515400	0.43178100
H	1.43562800	-2.87927100	2.48937000
H	3.81092600	-3.66944900	2.53159500
H	5.20226500	-3.50323100	0.47807700
H	4.23952500	-2.52765000	-1.59204600
H	1.90166600	-1.72798600	-1.61281500
P	-0.23831800	-1.71481100	0.44613800
O	-0.73058500	-1.83511000	1.93267400
H	-1.17707800	-0.67237200	2.40507800
C	-1.11824200	-3.05815000	-0.49928100
H	-2.16726200	-2.78517700	-0.36877800
C	-0.80100500	-3.03594300	-1.99589500
H	-1.45184300	-3.73635700	-2.53215900
H	-0.95533300	-2.03772200	-2.42145900
H	0.23464800	-3.33342400	-2.18966800
C	-0.89521300	-4.43084300	0.13891800
H	0.14262600	-4.75911900	0.01910700
H	-1.12013200	-4.39547100	1.20812400
H	-1.54492100	-5.18087400	-0.32755900

S-TS1B

Sum of electronic and thermal Free Energies = -2394.629674

O	1.71585600	1.84062800	-2.54037000
C	0.90123000	2.24037300	-1.71734200
O	-0.33078200	1.76332900	-1.61484400
C	1.21954000	3.31112600	-0.69232000
H	1.47877100	2.80877900	0.24497800
H	2.07670800	3.90341900	-1.01246600

H	0.35184400	3.95107500	-0.51412100
Cu	-0.75859900	0.24101800	0.19767700
P	-0.84507300	-1.17568500	-1.49506400
O	-0.77262400	-0.29195000	-2.80582100
H	-0.50484000	0.83690000	-2.33087500
C	-2.30542400	-2.31835400	-1.69943500
H	-2.07177900	-2.95207400	-2.56496000
C	-2.51836000	-3.20170000	-0.46558800
H	-1.68437800	-3.88785400	-0.29988400
H	-2.64020600	-2.58620500	0.43459300
H	-3.42672200	-3.80337200	-0.58215800
C	-3.55038100	-1.48155200	-2.00843700
H	-3.38671700	-0.83813100	-2.87406000
H	-4.40659800	-2.13506400	-2.21120200
H	-3.80918300	-0.83892500	-1.16135000
C	0.57545900	-2.33814400	-1.56956500
C	0.90083200	-3.18408600	-0.49894200
C	1.37774700	-2.35178100	-2.71826500
C	1.97951100	-4.06288900	-0.59182700
H	0.31803000	-3.14647800	0.41682900
C	2.46067800	-3.22768100	-2.80926300
H	1.13813100	-1.66269000	-3.52200700
C	2.75727600	-4.09293500	-1.75247600
H	2.22223000	-4.71293900	0.24371300
H	3.07414200	-3.23627200	-3.70591700
H	3.59786000	-4.77691700	-1.82651600
C	1.18252600	-0.98801100	2.27341800
C	-0.94441900	-0.43936000	3.00724100
C	-0.82342100	-1.23923100	4.14364300
C	0.36461100	-1.94979600	4.31849300
C	1.38482700	-1.82571100	3.37396700
H	-1.63995500	-1.30164600	4.85325600
H	0.49613700	-2.59059400	5.18392100
H	2.32580900	-2.35240000	3.47596900
N	0.03009500	-0.32807300	2.08430100
C	-2.14011800	0.33167500	2.65382200
C	-3.53131700	1.66838700	1.54237400
C	-4.27005900	0.98761500	2.71697900
H	-3.31058800	2.71376400	1.80334600
H	-4.80791200	1.67219100	3.37207100
H	-4.93423600	0.18460100	2.38389000
C	2.20919500	-0.75214600	1.24540500
C	3.38647500	0.10323100	-0.42371100
C	4.05176900	-1.21185100	0.06830000

H	3.12664800	0.05993800	-1.48212700
H	3.92754700	-2.03389800	-0.63791400
H	5.09981600	-1.09653600	0.34607900
N	2.12646800	0.15872400	0.35667800
N	-2.22877600	0.96193200	1.53718400
O	-3.19809100	0.36910900	3.49275800
O	3.30387700	-1.55662400	1.27476600
C	-4.20636000	1.65093700	0.18713100
C	-5.55224100	1.31319200	0.02236900
C	-3.44988100	2.01703400	-0.93453900
C	-6.13165500	1.32273700	-1.24921700
H	-6.16670100	1.04431500	0.87638900
C	-4.02686400	2.01777900	-2.20125900
H	-2.39974900	2.26838500	-0.83367200
C	-5.36975800	1.66998200	-2.36334400
H	-7.17749100	1.05419900	-1.36413800
H	-3.41540800	2.27300000	-3.06058600
H	-5.81781300	1.66573500	-3.35229400
C	4.22128300	1.34159900	-0.15316100
C	4.48901200	2.25974500	-1.17172000
C	4.73141300	1.57615300	1.13044100
C	5.26512800	3.39103800	-0.91119200
H	4.04036800	2.10919800	-2.14703900
C	5.50377600	2.70615900	1.39122200
H	4.52318900	0.87001000	1.93037500
C	5.77517200	3.61689200	0.36727700
H	5.46451700	4.10014900	-1.70942000
H	5.89322000	2.87715600	2.39071400
H	6.37716800	4.49834900	0.56795600

S-Int1B

Sum of electronic and thermal Free Energies = -2394.638293

C	-1.31491200	-2.56203000	1.70935500
C	0.94584300	-2.62807700	1.23549800
C	0.99703900	-4.00605500	1.45743900
C	-0.17481300	-4.66976300	1.81677300
C	-1.35395400	-3.93762400	1.94539100
H	1.94019200	-4.52675900	1.34588600
H	-0.16913400	-5.73986400	1.99437700
H	-2.29162300	-4.40435700	2.22154300
N	-0.19047800	-1.91265500	1.34896500
C	2.12518000	-1.85557100	0.82177700
C	3.43199100	-0.21623400	0.12000600
C	4.25789300	-1.53480000	0.22191600
H	3.29423600	0.05571900	-0.93307800

H	4.71733000	-1.83370800	-0.72034200
H	5.00669100	-1.50003100	1.01814000
C	-2.50723400	-1.71013200	1.81076600
C	-3.86330300	0.04266400	1.83356900
C	-4.69713200	-1.26506900	1.88008200
H	-3.88790000	0.53699600	2.81535700
H	-5.40375200	-1.31988100	2.70869000
H	-5.21247700	-1.46533800	0.93605600
N	-2.48107700	-0.44611000	1.64283700
N	2.11024700	-0.59462400	0.63762300
O	3.26919100	-2.54479300	0.57360900
O	-3.70009700	-2.31115100	2.06776700
C	4.04409100	0.95963300	0.85110700
C	5.37457500	1.30982500	0.58901700
C	3.30416300	1.71127300	1.76893000
C	5.96626700	2.38882400	1.24412000
H	5.95190600	0.74060400	-0.13634000
C	3.90124100	2.79066600	2.42435500
H	2.26326300	1.46560900	1.94825800
C	5.22963900	3.13149400	2.16880100
H	6.99911300	2.64953600	1.03212000
H	3.31949900	3.36666000	3.13775100
H	5.68791700	3.97170100	2.68202700
C	-4.24397500	1.05667500	0.77193900
C	-5.51922500	1.10361200	0.20339100
C	-3.27523700	1.97717500	0.35605600
C	-5.81248500	2.04365900	-0.78821000
H	-6.29454200	0.41197500	0.52144200
C	-3.56292700	2.90873100	-0.63799300
H	-2.28657400	1.93811800	0.79289400
C	-4.83417600	2.94125200	-1.21657800
H	-6.80501900	2.06688100	-1.22868000
H	-2.77939200	3.58648200	-0.95894500
H	-5.06123300	3.66116000	-1.99725600
O	-0.00653400	1.80761100	1.43087300
C	0.04625900	2.94463200	0.86150600
O	-0.21986200	3.20069700	-0.34445100
C	0.49231300	4.11618200	1.72579200
H	0.37705400	3.88787400	2.78685400
H	1.55021700	4.31285100	1.52256600
H	-0.06986900	5.01479000	1.46236500
Cu	-0.30736300	0.02316900	0.51206300
P	-0.50855800	0.08993200	-1.68696600
O	-0.68438000	1.60242900	-2.23575300

H	-0.51501700	2.25297300	-1.44940500
C	-1.78372600	-0.87050000	-2.64424900
H	-1.48358100	-1.92028500	-2.51469500
C	-3.17701800	-0.67455700	-2.03881000
H	-3.91374700	-1.28225200	-2.57621900
H	-3.19575200	-0.95868200	-0.98472700
H	-3.48849700	0.37106500	-2.10225700
C	-1.75264500	-0.51426400	-4.13496800
H	-0.77328300	-0.71571100	-4.57748400
H	-2.50271100	-1.09751200	-4.68092400
H	-1.97418600	0.54740800	-4.27330500
C	1.03196600	-0.50290300	-2.49023200
C	1.44003300	-1.83664000	-2.33803100
C	1.86135400	0.38847700	-3.18110800
C	2.65482400	-2.27154400	-2.86385800
H	0.81270800	-2.53543100	-1.79012600
C	3.07909800	-0.04709400	-3.70984400
H	1.53974900	1.41878000	-3.29277400
C	3.48023700	-1.37494700	-3.55043900
H	2.96189600	-3.30505200	-2.73260800
H	3.71553900	0.65229700	-4.24438700
H	4.42800300	-1.71196400	-3.96007700

S-Int2B

Sum of electronic and thermal Free Energies = -2489.886077

C	1.52740900	0.17856800	2.76645400
C	-0.68305000	0.86209300	2.68382700
C	-0.60258100	1.45096700	3.94586800
C	0.61559800	1.40534800	4.62157200
C	1.69843000	0.75753100	4.02561100
H	-1.47612700	1.93462100	4.36675400
H	0.71992000	1.86254700	5.59967200
H	2.66190700	0.68767600	4.51523400
N	0.36744400	0.25241600	2.08798600
C	-1.88492200	0.89580100	1.84839900
C	-3.19009200	0.69804900	0.05507300
C	-3.98897300	1.34167900	1.22693700
H	-3.03173000	1.42561500	-0.74444700
H	-4.36670800	2.33455600	0.98444800
H	-4.79831600	0.70566200	1.59322400
C	2.61427700	-0.55147900	2.09193700
C	3.79026900	-1.83377300	0.72328400
C	4.75492000	-1.05716200	1.67437000
H	3.80902100	-2.90677700	0.95143900
H	5.41477100	-1.69958900	2.26010600

H	5.34224600	-0.30658800	1.13941400
N	2.45651400	-1.32217800	1.08874100
N	-1.87780900	0.42149600	0.66045800
O	-3.01182700	1.48750800	2.30523100
O	3.86674300	-0.36039300	2.59178000
C	-3.79047600	-0.55867700	-0.52946200
C	-4.00212400	-0.65580600	-1.90573700
C	-4.09710500	-1.65135700	0.28993700
C	-4.52465400	-1.82607500	-2.45863800
H	-3.74440900	0.18563000	-2.54288100
C	-4.62553500	-2.81686200	-0.25850700
H	-3.90207700	-1.59765500	1.35741100
C	-4.84047000	-2.90580800	-1.63564000
H	-4.67755500	-1.89507000	-3.53119500
H	-4.84924600	-3.66221300	0.38388400
H	-5.24095200	-3.81883100	-2.06545700
C	4.11951400	-1.64233300	-0.74123400
C	3.52857400	-0.61096100	-1.47307800
C	5.06346400	-2.46615600	-1.36311700
C	3.86792500	-0.39494900	-2.80729000
H	2.77780400	0.01289500	-1.01088000
C	5.41440200	-2.24943600	-2.69649600
H	5.52084300	-3.28203400	-0.80753900
C	4.81811900	-1.21369800	-3.42028100
H	3.36618400	0.40911100	-3.33684100
H	6.14672300	-2.89549600	-3.17222100
H	5.08815300	-1.05392500	-4.46016200
Cu	0.18528400	0.11816800	-0.02151800
P	0.83661200	1.98319900	-1.10026300
O	1.36925600	1.71124600	-2.52275400
H	0.70950200	-0.37736900	-2.53466800
C	1.93948700	3.21798600	-0.21936500
H	1.41762400	3.48746000	0.70953000
C	3.28225100	2.57544600	0.14547900
H	3.93420000	3.30190900	0.64457200
H	3.15675600	1.72248800	0.81764600
H	3.79725500	2.22143200	-0.75243100
C	2.13648600	4.46800700	-1.08423900
H	1.18715300	4.97685500	-1.27321400
H	2.81626700	5.17798300	-0.59794800
H	2.56331300	4.18615900	-2.05130800
C	-0.73068700	2.95770100	-1.22015500
C	-1.32548000	3.58607600	-0.11476000
C	-1.41516100	2.95727500	-2.44283000

C	-2.57036400	4.20455800	-0.23001000
H	-0.81692700	3.59073800	0.84581600
C	-2.66759100	3.56555600	-2.55811300
H	-0.94111300	2.47599800	-3.29330300
C	-3.24991200	4.19055300	-1.45255800
H	-3.01078600	4.69868800	0.63221900
H	-3.18711300	3.55891900	-3.51263700
H	-4.22110500	4.66849600	-1.54270900
C	-2.00431100	-4.93827600	-0.46885400
C	-0.85898800	-2.95569800	-0.35188900
C	-0.86550400	-2.98528400	1.05380900
C	-1.47709400	-4.05666300	1.69370100
C	-2.06553000	-5.05739700	0.92111300
H	-2.45205000	-5.70052800	-1.10393100
H	-0.39162000	-2.18082100	1.60132800
H	-1.49566700	-4.10936600	2.77852900
H	-2.55685500	-5.91043900	1.37781600
N	-1.41747700	-3.92380500	-1.10617600
C	-0.24610400	-1.86847900	-1.06118600
C	0.25461900	-1.03453200	-1.80866800

TS2B-R

Sum of electronic and thermal Free Energies = -2489.835201

C	0.77552600	-1.92300400	-2.31544700
C	-1.40939000	-2.35464700	-1.66514000
C	-1.47756000	-3.48391400	-2.47698800
C	-0.35660800	-3.82894900	-3.23310500
C	0.78532900	-3.03140000	-3.16168100
H	-2.39021700	-4.06689400	-2.50631800
H	-0.37507900	-4.70472200	-3.87286400
H	1.67519500	-3.25446400	-3.73825700
N	-0.30333700	-1.58402300	-1.58098700
C	-2.50756000	-1.87027700	-0.82730000
C	-3.61021200	-0.64687400	0.67185700
C	-4.55024400	-1.73538100	0.08075400
H	-3.34475500	-0.86040800	1.71114200
H	-4.95770500	-2.40810400	0.83679900
H	-5.36621800	-1.33097200	-0.52350600
C	1.90114700	-0.99522800	-2.16808400
C	3.09119500	0.75050700	-1.49543500
C	3.75285500	0.11673900	-2.75070400
H	2.59319000	1.69616000	-1.73890600
H	3.89125600	0.81857900	-3.57396700
H	4.70276600	-0.37873200	-2.52859500
N	2.01119500	-0.20442600	-1.17000900

N	-2.37082800	-0.82945200	-0.09942900
O	-3.69094900	-2.52721800	-0.80040700
O	2.80034600	-0.89910000	-3.18465600
C	-4.07252600	0.79212500	0.58986500
C	-3.77190700	1.64784500	1.65569900
C	-4.70774600	1.30762300	-0.54417900
C	-4.12812200	2.99512400	1.59725800
H	-3.23105100	1.24584000	2.50760700
C	-5.06667100	2.65477000	-0.60232700
H	-4.91443400	0.66342600	-1.39462800
C	-4.78281200	3.50055600	0.47200600
H	-3.88869100	3.65250700	2.42779300
H	-5.56201900	3.04433100	-1.48683100
H	-5.06107600	4.54936300	0.42735900
C	4.01590200	0.99798000	-0.32758800
C	4.00674100	2.24970300	0.29576500
C	4.85140700	-0.00908800	0.16789200
C	4.83605300	2.49455700	1.39147600
H	3.32348100	3.01057000	-0.07164000
C	5.67899400	0.23477100	1.26276600
H	4.84573500	-0.99337100	-0.29362300
C	5.67530600	1.49018000	1.87505200
H	4.82091000	3.46848100	1.87192200
H	6.32149700	-0.55427000	1.64244200
H	6.31920100	1.68040100	2.72860600
Cu	-0.27864300	-0.04462200	-0.23312000
P	-0.20447000	-0.62691000	2.21894800
O	-1.55979200	-0.61678100	2.92092500
H	0.16611400	1.84677000	2.25819000
C	1.15214000	-0.38051000	3.49065300
H	1.20306600	-1.36823500	3.96747100
C	2.52479900	-0.03344800	2.91055800
H	3.23763400	0.15471900	3.72069300
H	2.93506100	-0.83885300	2.30145700
H	2.48781400	0.86212300	2.28622400
C	0.70730000	0.62878300	4.56024100
H	-0.32566600	0.44947800	4.86517800
H	1.35700000	0.54342400	5.43845100
H	0.78825300	1.66149200	4.20442600
C	0.13911900	-2.32555200	1.58316900
C	1.33494500	-2.69790400	0.94857200
C	-0.90812800	-3.25519200	1.66596700
C	1.47452200	-3.97359900	0.40686400
H	2.13036700	-1.97422500	0.82086000

C	-0.76716400	-4.52847100	1.11316600
H	-1.82327200	-2.95803600	2.16748400
C	0.42255000	-4.89157900	0.47982800
H	2.40254500	-4.24960500	-0.08631300
H	-1.58810800	-5.23751900	1.17624700
H	0.53037200	-5.88059600	0.04438200
C	1.00389100	4.60677900	-2.07001700
C	-0.11249700	3.08150500	-0.74805300
C	-1.34872000	3.71217700	-1.02326500
C	-1.36403300	4.82749900	-1.84257800
C	-0.16180400	5.29657700	-2.38835100
H	1.96086900	4.93600000	-2.47322500
H	-2.25727600	3.31298400	-0.58924600
H	-2.30344300	5.32989600	-2.05731900
H	-0.13051400	6.16438000	-3.03827800
N	1.05351400	3.52845400	-1.27680400
C	-0.08238400	1.90443500	0.06772800
C	0.03040800	1.44757100	1.26123100

Int3B-R

Sum of electronic and thermal Free Energies = -2489.915585

C	-0.81422400	3.15942500	-0.86763500
C	1.26682800	3.20306900	0.14524000
C	1.24206300	4.59393200	0.25287300
C	0.12570000	5.27473700	-0.22871200
C	-0.92218900	4.54975700	-0.79683300
H	2.07784000	5.11048200	0.70972300
H	0.06918900	6.35590900	-0.15857600
H	-1.80802900	5.03569600	-1.18710500
N	0.26870200	2.49564100	-0.42326100
C	2.35959800	2.35977800	0.64152000
C	3.50729600	0.51423300	1.13017500
C	4.34929500	1.77450500	1.46976400
H	3.36472500	-0.14685300	1.98738300
H	4.45258600	1.93137100	2.54704500
H	5.33015400	1.79601100	0.99453600
C	-1.87247000	2.31256600	-1.44195100
C	-3.11541600	0.55737500	-1.98327100
C	-3.71598500	1.84141100	-2.61991000
H	-2.85757800	-0.18363500	-2.74385300
H	-3.58565900	1.86321600	-3.70526500
H	-4.76324700	2.02092900	-2.37529600
N	-1.86313800	1.04211400	-1.37163200
N	2.19838000	1.10100400	0.77951900
O	3.56906400	2.89588900	0.93553000

O	-2.92258200	2.92828300	-2.05751000
C	3.99287600	-0.30536800	-0.05112000
C	3.88472900	-1.69887600	-0.01245100
C	4.46799700	0.31899500	-1.21084900
C	4.26660700	-2.45775200	-1.11938100
H	3.46457600	-2.16519200	0.87394100
C	4.85040000	-0.44167600	-2.31555300
H	4.53271300	1.40345000	-1.25780800
C	4.75221200	-1.83451200	-2.26986600
H	4.17205600	-3.53898000	-1.08639700
H	5.22087700	0.05095200	-3.20989200
H	5.04461700	-2.42870400	-3.13069300
C	-3.94147900	-0.11824300	-0.90247700
C	-3.75683900	-1.48653600	-0.66874100
C	-4.79597800	0.61203900	-0.06940600
C	-4.43949000	-2.11379700	0.37382000
H	-3.04008600	-2.04057800	-1.27252400
C	-5.47266200	-0.01732200	0.97628900
H	-4.92521700	1.68058800	-0.21983100
C	-5.29806200	-1.38394500	1.19786500
H	-4.28960300	-3.17434000	0.54979100
H	-6.13102500	0.56020200	1.61845200
H	-5.82332100	-1.87510500	2.01146800
Cu	0.40798400	0.40203600	-0.27278700
P	0.51641700	-1.97709400	2.08616400
O	1.96839900	-1.95459300	2.54010000
H	0.06898800	-3.47951500	0.16778600
C	-0.45985300	-3.17490900	3.10614700
H	-0.37521200	-2.78353900	4.12855500
C	-1.93884000	-3.23854100	2.71000700
H	-2.47109100	-3.95805400	3.34284100
H	-2.43316400	-2.26956100	2.81254600
H	-2.05028900	-3.55745800	1.66928900
C	0.21446300	-4.55385600	3.05712900
H	1.27379500	-4.47903100	3.31341200
H	-0.27077400	-5.24020100	3.76007200
H	0.13584400	-4.99558000	2.05773700
C	-0.25000300	-0.34049300	2.39274300
C	-1.44544000	0.08467800	1.79063900
C	0.41772400	0.52159800	3.27466500
C	-1.95476500	1.35658400	2.06657400
H	-1.97065600	-0.56472800	1.10269900
C	-0.08867000	1.79242300	3.54085000
H	1.34914200	0.18540800	3.71856200

C	-1.27452100	2.21349800	2.93166900
H	-2.87732300	1.66980800	1.59076300
H	0.43932600	2.45582100	4.22042500
H	-1.66894200	3.20507200	3.13680500
C	-1.20104100	-2.87027500	-3.67760700
C	0.08343900	-1.91515100	-2.01074700
C	1.11050300	-1.64109800	-2.94045200
C	0.95857900	-2.03327400	-4.26049800
C	-0.23158300	-2.65940800	-4.65158800
H	-2.14558800	-3.34678300	-3.93898700
H	2.00595900	-1.13430300	-2.59958000
H	1.75019200	-1.84773500	-4.98171700
H	-0.40194000	-2.96903500	-5.67757900
N	-1.06129300	-2.52876800	-2.38668000
C	0.24490200	-1.48889800	-0.61179300
C	0.20110200	-2.41253900	0.37271100

7. Determination of the absolute configurations

1)The absolute configurations of all products are determined to be (*R*)-enantiomers by X-ray structure analysis of compound **10.**

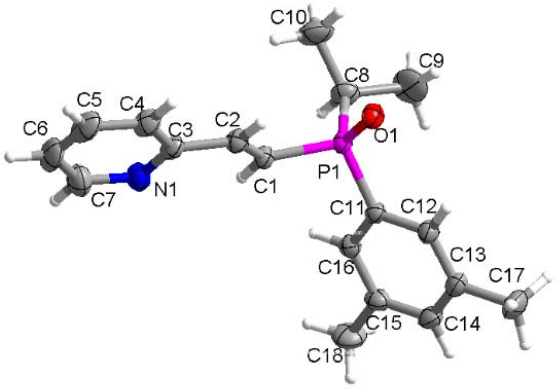

Fig. S12. X-ray single-crystal structure of **10** (CCDC 2391969)

Table 3 Crystal data and structure refinement for 10.

Identification code	**10**
Empirical formula	$C_{18}H_{22}NOP$
Formula weight	299.33
Temperature/K	293(2)
Crystal system	orthorhombic
Space group	$P2_12_12_1$
a/Å	9.9528(3)
b/Å	10.6897(3)
c/Å	16.8353(5)
α/°	90
β/°	90
γ/°	90
Volume/Å^3	1791.15(9)
Z	4
ρ_{calc}g/cm^3	1.110
μ/mm^{-1}	1.338
F(000)	640.0
Crystal size/mm^3	0.15 × 0.11 × 0.1
Radiation	CuKα (λ = 1.54184)
2Θ range for data collection/°	9.802 to 141.84
Index ranges	$-8 \le h \le 12$, $-12 \le k \le 12$, $-16 \le l \le 20$
Reflections collected	6722
Independent reflections	3378 [R_{int} = 0.0313, R_{sigma} = 0.0471]

Data/restraints/parameters	3378/0/194
Goodness-of-fit on F^2	1.058
Final R indexes [I>=2σ (I)]	R_1 = 0.0506, wR_2 = 0.1350
Final R indexes [all data]	R_1 = 0.0546, wR_2 = 0.1411
Largest diff. peak/hole / e $Å^{-3}$	0.35/-0.31
Flack parameter	0.00(2)

Table 4 Fractional Atomic Coordinates (×10^4) and Equivalent Isotropic Displacement Parameters ($Å^2$×10^3) for 10 U_{eq} is defined as 1/3 of of the trace of the orthogonalised U_{IJ} tensor.

Atom	*x*	*y*	*z*	U(eq)
P1	5943.9(8)	7314.2(8)	5396.5(5)	39.1(2)
O1	7297(3)	6772(3)	5538.8(17)	52.5(7)
N1	4505(4)	6704(4)	2799(2)	62.0(9)
C1	5380(3)	7116(3)	4394(2)	42.4(7)
C2	6076(4)	6452(4)	3879(2)	48.5(8)
C3	5643(4)	6177(4)	3064(2)	49.3(8)
C4	6362(5)	5354(5)	2599(3)	65.5(11)
C5	5911(7)	5049(5)	1853(3)	81.5(15)
C6	4759(7)	5555(6)	1593(3)	85.9(18)
C7	4074(6)	6398(6)	2071(3)	79.2(14)
C8	4643(5)	6612(4)	6011(3)	60.1(10)
C9	4910(9)	6913(8)	6882(3)	114(3)
C10	4584(7)	5201(5)	5856(4)	95.7(19)
C11	5860(3)	8977(3)	5600.0(18)	40.2(6)
C12	6953(4)	9541(4)	5957(2)	50.0(8)
C13	6918(4)	10808(4)	6141(2)	57.1(10)
C14	5760(5)	11474(4)	5977(2)	59.1(10)
C15	4651(4)	10925(4)	5623(2)	53.3(9)
C16	4705(3)	9652(3)	5438(2)	44.7(7)
C17	8115(6)	11429(5)	6539(4)	84.7(16)
C18	3378(6)	11660(5)	5447(4)	85.3(16)

Table 5 Anisotropic Displacement Parameters ($Å^2$×10^3) for 10. The Anisotropic displacement factor exponent takes the form: $-2\pi^2[h^2a^{*2}U_{11}+2hka^*b^*U_{12}+\ldots]$.

Atom	U_{11}	U_{22}	U_{33}	U_{23}	U_{13}	U_{12}
P1	39.4(4)	39.1(4)	38.8(4)	-5.0(3)	-1.1(3)	0.9(3)
O1	47.1(13)	52.6(14)	57.7(16)	-8.1(13)	-8.3(11)	6.3(11)
N1	72(2)	69(2)	45.6(17)	-7.7(16)	-0.2(15)	4.7(18)
C1	43.6(14)	41.5(17)	42.1(16)	-7.2(13)	-1.4(13)	1.0(13)
C2	47.9(17)	48.3(18)	49.3(18)	-5.5(15)	1.3(15)	2.7(16)

C3	58(2)	44.5(19)	45.7(18)	-7.4(14)	7.7(15)	-6.2(15)
C4	77(3)	58(3)	61(2)	-15(2)	15(2)	2(2)
C5	111(4)	71(3)	63(3)	-27(2)	29(3)	-13(3)
C6	120(5)	96(4)	42(2)	-21(2)	10(3)	-28(4)
C7	91(3)	95(4)	52(2)	-4(2)	-8(2)	-3(3)
C8	63(2)	57(2)	60(2)	5.5(19)	12.8(19)	-8.3(19)
C9	170(7)	117(6)	56(3)	9(3)	37(4)	-27(5)
C10	122(5)	59(3)	106(4)	13(3)	21(4)	-26(3)
C11	48.0(15)	36.2(15)	36.6(14)	-3.4(11)	3.4(13)	-2.0(14)
C12	49.7(18)	48(2)	51.9(19)	-7.9(16)	-7.7(16)	-1.8(15)
C13	68(2)	50(2)	53(2)	-7.4(18)	-6.4(18)	-12.8(19)
C14	86(3)	37.0(17)	55(2)	-5.9(16)	1(2)	-1.7(19)
C15	66(2)	44.5(19)	49.2(19)	1.6(16)	3.3(16)	11.5(18)
C16	47.8(15)	45.0(17)	41.4(16)	-0.6(15)	2.5(15)	0.3(14)
C17	91(4)	63(3)	99(4)	-15(3)	-26(3)	-22(3)
C18	88(3)	56(3)	111(4)	1(3)	-4(3)	25(2)

Table 6 Bond Lengths for 10.

Atom	Atom	Length/Å	Atom	Atom	Length/Å
P1	O1	1.485(3)	C6	C7	1.386(8)
P1	C1	1.790(3)	C8	C9	1.525(7)
P1	C8	1.819(4)	C8	C10	1.532(7)
P1	C11	1.812(3)	C11	C12	1.381(5)
N1	C3	1.341(6)	C11	C16	1.385(5)
N1	C7	1.339(6)	C12	C13	1.390(6)
C1	C2	1.319(5)	C13	C14	1.382(6)
C2	C3	1.467(5)	C13	C17	1.519(6)
C3	C4	1.379(6)	C14	C15	1.385(6)
C4	C5	1.373(7)	C15	C16	1.397(5)
C5	C6	1.341(9)	C15	C18	1.520(6)

Table 7 Bond Angles for 10.

Atom	Atom	Atom	Angle/°	Atom	Atom	Atom	Angle/°
O1	P1	C1	112.96(16)	C9	C8	P1	109.6(4)
O1	P1	C8	113.1(2)	C9	C8	C10	112.2(5)
O1	P1	C11	113.19(16)	C10	C8	P1	109.7(4)
C1	P1	C8	105.3(2)	C12	C11	P1	118.3(3)
C1	P1	C11	106.25(16)	C12	C11	C16	120.9(3)
C11	P1	C8	105.33(18)	C16	C11	P1	120.8(3)
C7	N1	C3	118.2(4)	C11	C12	C13	120.1(4)

C2	C1	P1	121.3(3)	C12	C13	C17	120.3(4)
C1	C2	C3	124.7(4)	C14	C13	C12	118.6(4)
N1	C3	C2	118.4(3)	C14	C13	C17	121.1(4)
N1	C3	C4	121.2(4)	C13	C14	C15	122.2(4)
C4	C3	C2	120.4(4)	C14	C15	C16	118.6(4)
C5	C4	C3	120.1(5)	C14	C15	C18	121.9(4)
C6	C5	C4	118.9(5)	C16	C15	C18	119.5(4)
C5	C6	C7	119.5(5)	C11	C16	C15	119.7(3)
N1	C7	C6	122.2(6)				

Table 8 Torsion Angles for 10.

A	B	C	D	Angle/°	A	B	C	D	Angle/°
P1	C1	C2	C3	175.8(3)	C5	C6	C7	N1	-1.8(9)
P1	C11	C12	C13	-178.4(3)	C7	N1	C3	C2	-176.5(4)
P1	C11	C16	C15	178.0(3)	C7	N1	C3	C4	0.8(7)
O1	P1	C1	C2	5.7(4)	C8	P1	C1	C2	-118.2(3)
O1	P1	C8	C9	65.2(5)	C8	P1	C11	C12	115.7(3)
O1	P1	C8	C10	-58.4(5)	C8	P1	C11	C16	-61.2(3)
O1	P1	C11	C12	-8.4(3)	C11	P1	C1	C2	130.4(3)
O1	P1	C11	C16	174.7(3)	C11	P1	C8	C9	-58.9(5)
N1	C3	C4	C5	-0.7(7)	C11	P1	C8	C10	177.5(4)
C1	P1	C8	C9	-171.0(4)	C11	C12	C13	C14	1.5(6)
C1	P1	C8	C10	65.4(4)	C11	C12	C13	C17	179.6(4)
C1	P1	C11	C12	-132.9(3)	C12	C11	C16	C15	1.3(5)
C1	P1	C11	C16	50.2(3)	C12	C13	C14	C15	-1.2(7)
C1	C2	C3	N1	4.9(6)	C13	C14	C15	C16	0.9(6)
C1	C2	C3	C4	-172.3(4)	C13	C14	C15	C18	179.7(5)
C2	C3	C4	C5	176.5(4)	C14	C15	C16	C11	-0.9(6)
C3	N1	C7	C6	0.5(8)	C16	C11	C12	C13	-1.6(6)
C3	C4	C5	C6	-0.6(8)	C17	C13	C14	C15	-179.3(4)
C4	C5	C6	C7	1.8(9)	C18	C15	C16	C11	-179.7(4)

Table 9 Hydrogen Atom Coordinates (Å×10^4) and Isotropic Displacement Parameters (Å^2×10^3) for 10.

Atom	*x*	*y*	*z*	U(eq)
H1	4580	7489	4235	51
H2	6901	6135	4043	58
H4	7155	5004	2791	79
H5	6395	4500	1534	98
H6	4422	5343	1096	103
H7	3290	6762	1880	95

H8	3776	6978	5861	72
H9A	5809	6667	7018	172
H9B	4282	6466	7209	172
H9C	4806	7796	6968	172
H10A	4462	5053	5298	144
H10B	3845	4845	6144	144
H10C	5408	4820	6028	144
H12	7715	9071	6074	60
H14	5725	12318	6108	71
H16	3968	9259	5207	54
H17A	8211	11112	7069	127
H17B	7975	12317	6558	127
H17C	8915	11251	6241	127
H18A	2838	11207	5072	128
H18B	3612	12461	5228	128
H18C	2880	11778	5929	128

2)The absolute configurations of **47** and *ent*-**47** are determined by NMR.

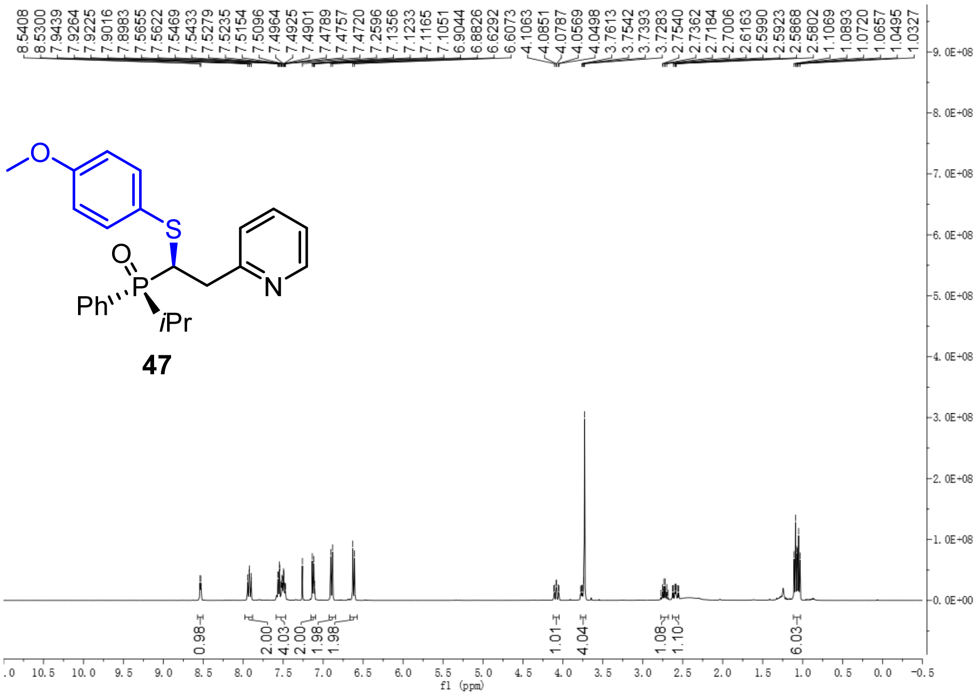

Fig. S13. ^{1}H NMR (400 MHz, $CDCl_3$) of **47**

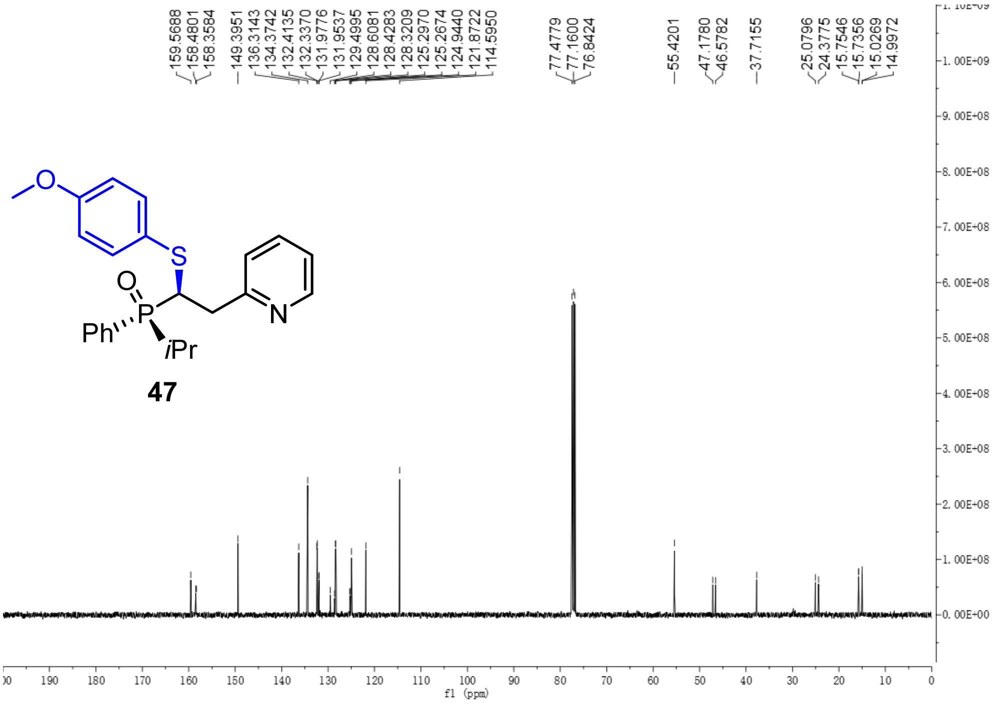

Fig. S14. ^{13}C NMR (101 MHz, $CDCl_3$) of **47**

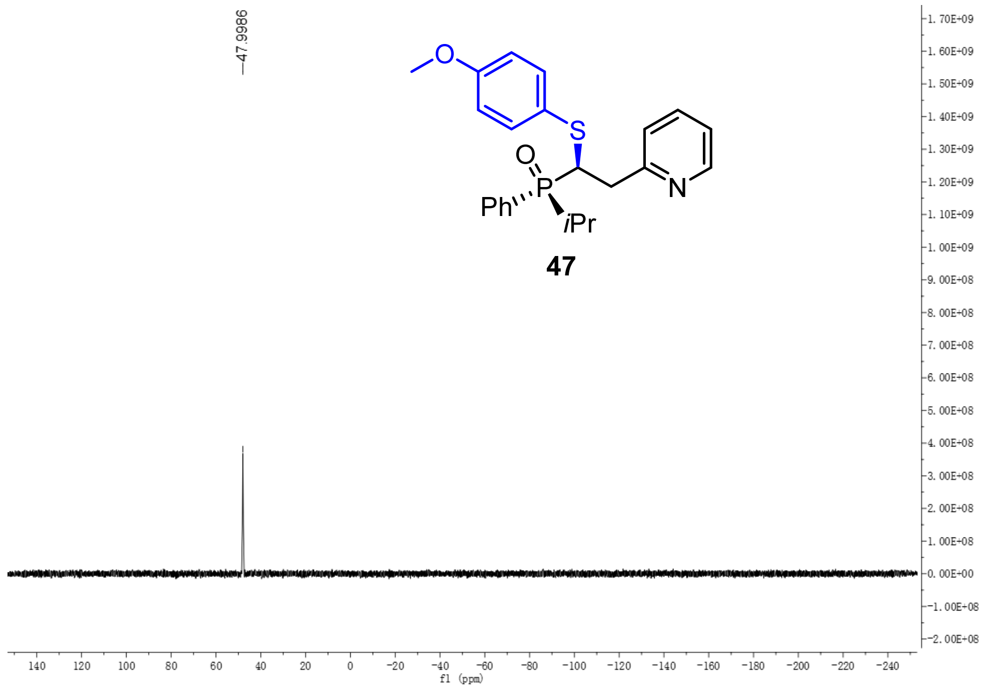

Fig. S15. ^{31}P NMR (162 MHz, $CDCl_3$) of **47**

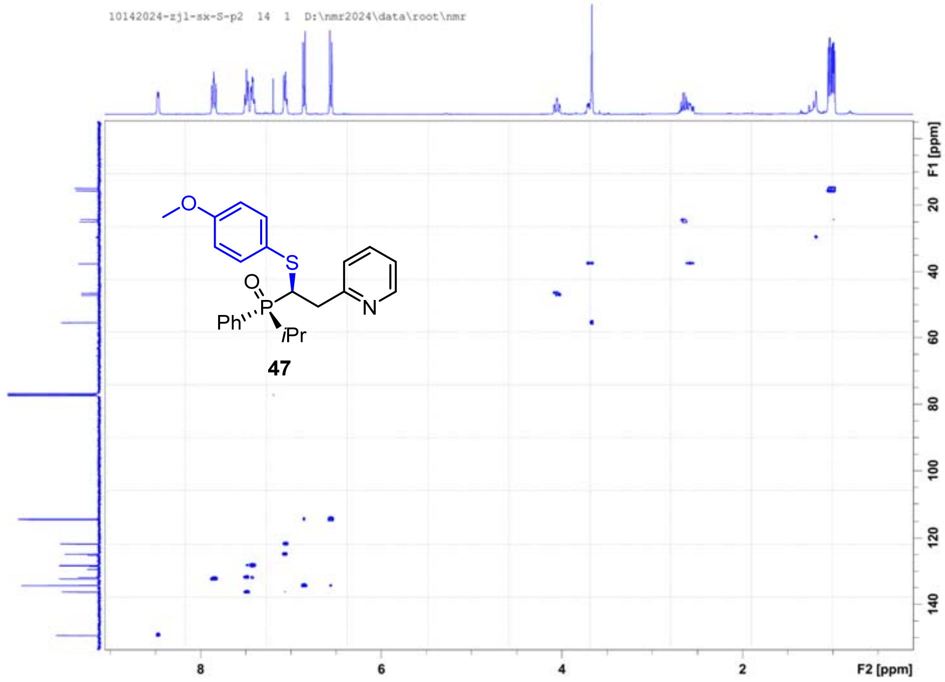

Fig. S16. ^{1}H- ^{13}C HSQC (400 MHz, $CDCl_3$) of **47**

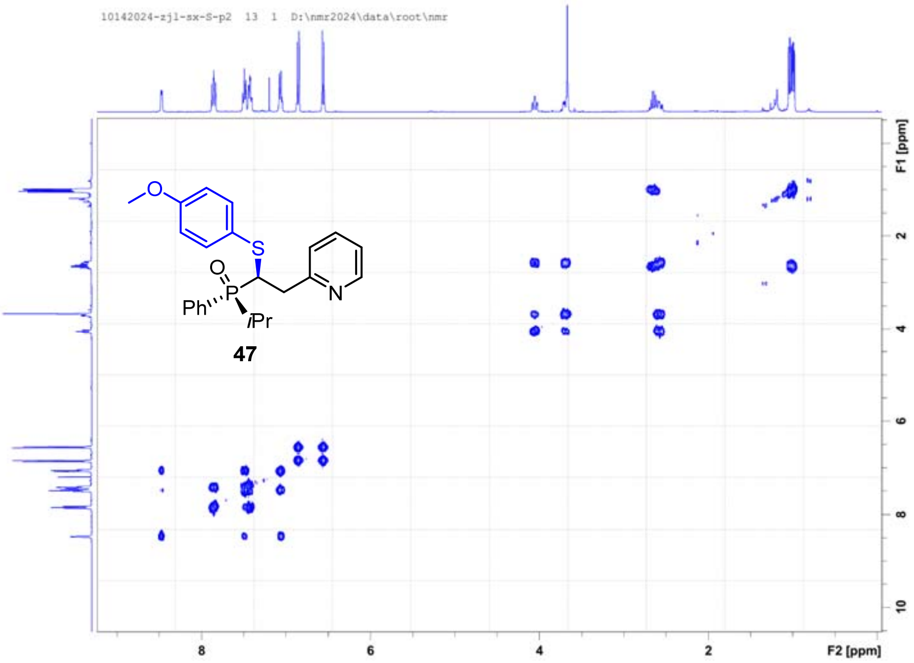

Fig. S17. ^{1}H- ^{1}H COSY (400 MHz, $CDCl_3$) of **47**

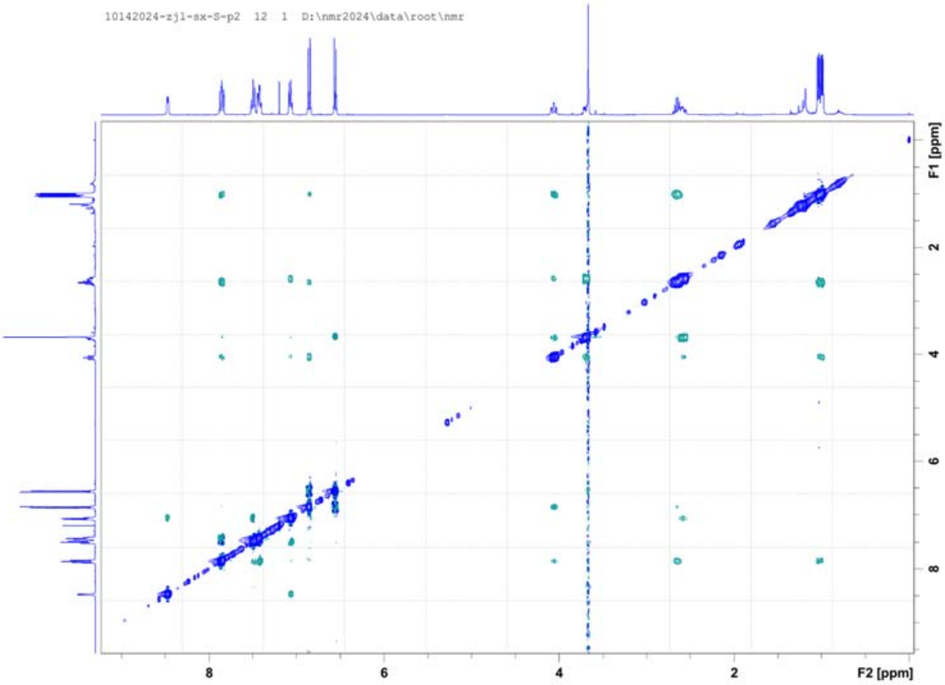

Fig. S18. ^{1}H- ^{1}H NOESY (400 MHz, $CDCl_3$) of **47**

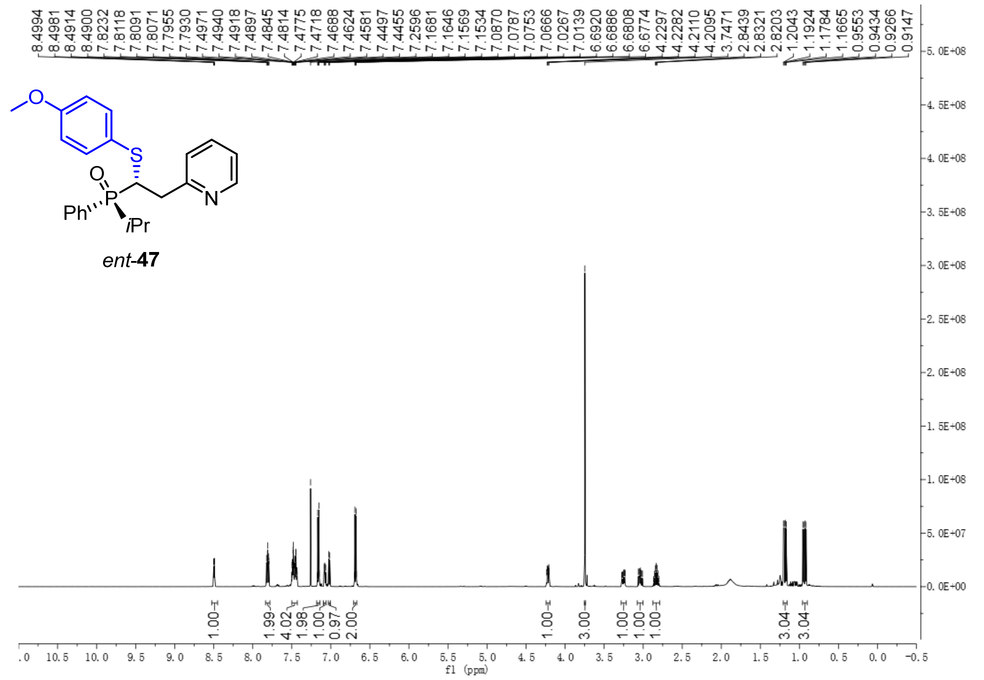

Fig. S19. ^{1}H NMR (600 MHz, $CDCl_3$) of *ent*-**47**

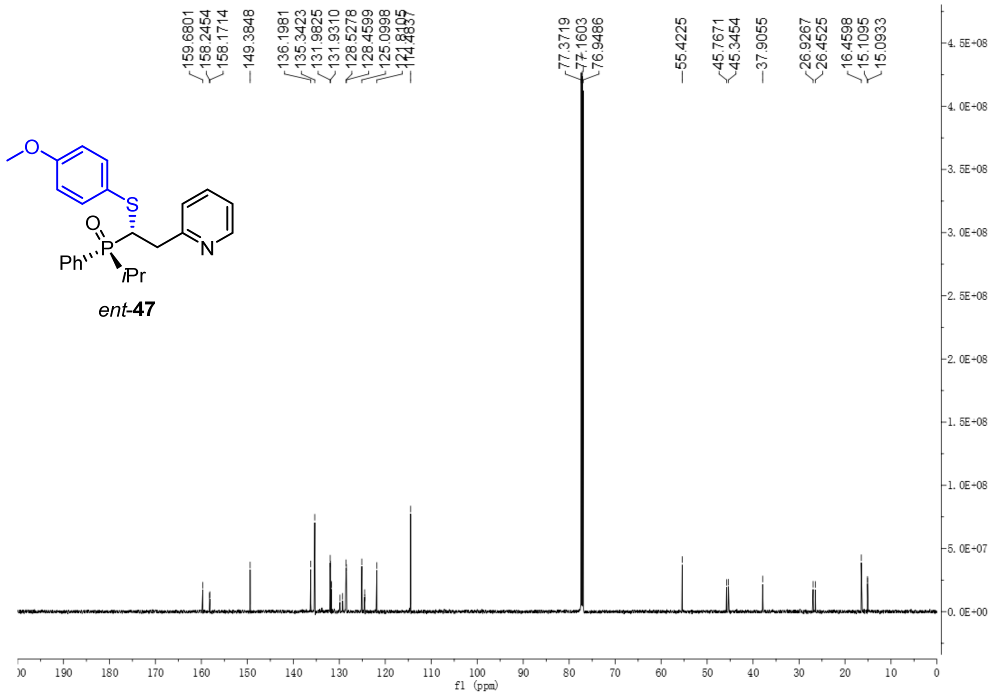

Fig. S20. ^{13}C NMR (151 MHz, $CDCl_3$) of *ent*-**47**

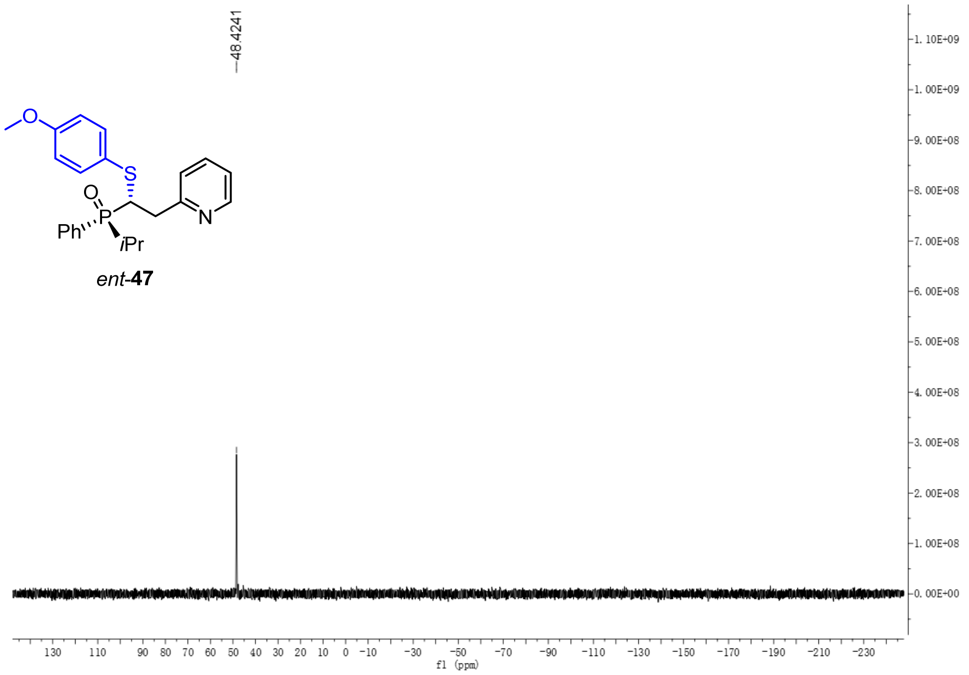

Fig. S21. ^{31}P NMR (243 MHz, $CDCl_3$) of *ent*-**47**

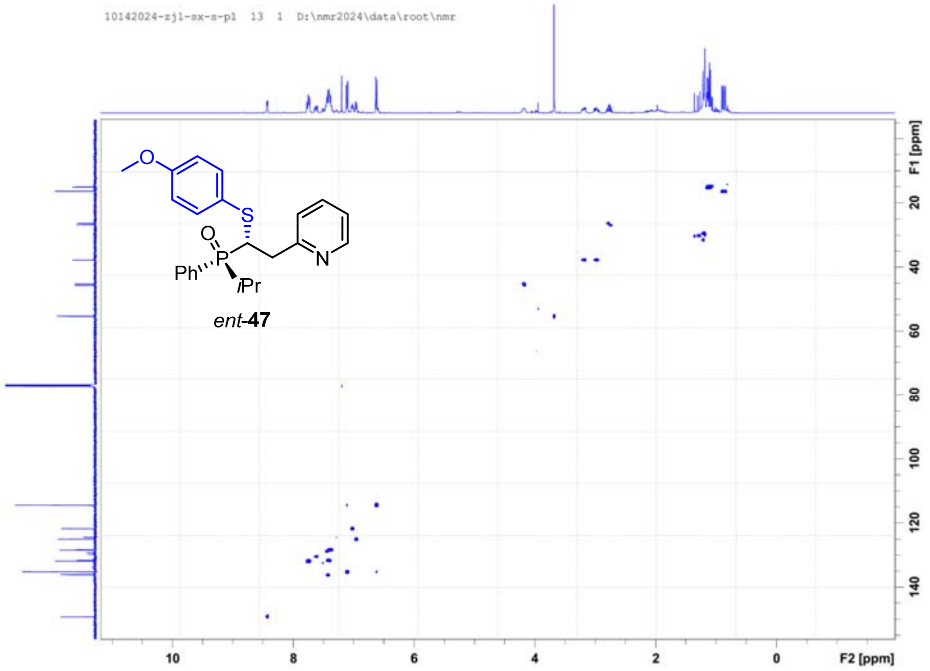

Fig. S22. ^{1}H- ^{13}C HSQC (400 MHz, $CDCl_3$) of *ent*-**47**

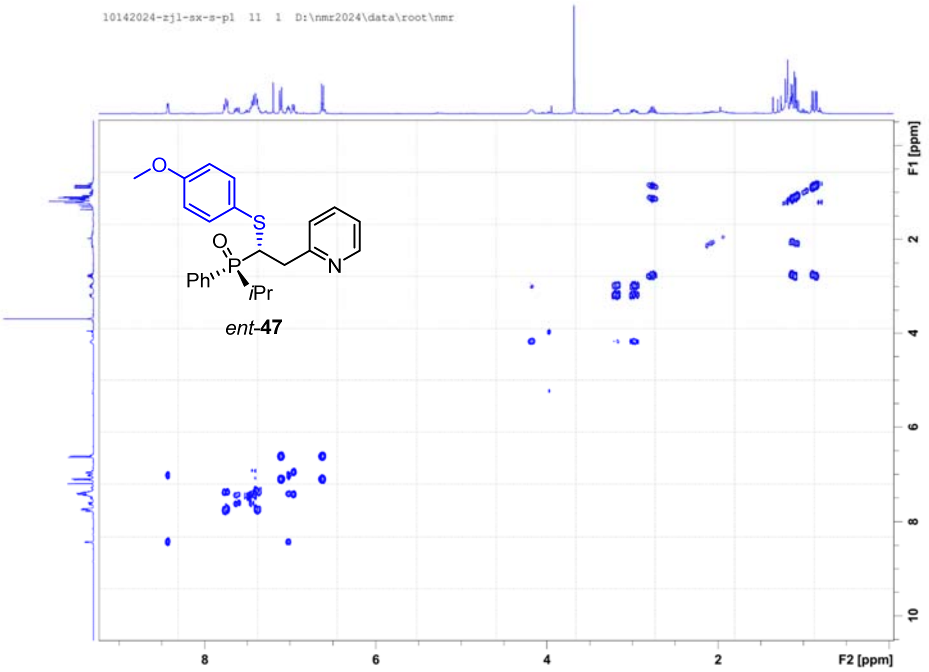

Fig. S23. ^{1}H- ^{1}H COSY (400 MHz, $CDCl_3$) of *ent*-**47**

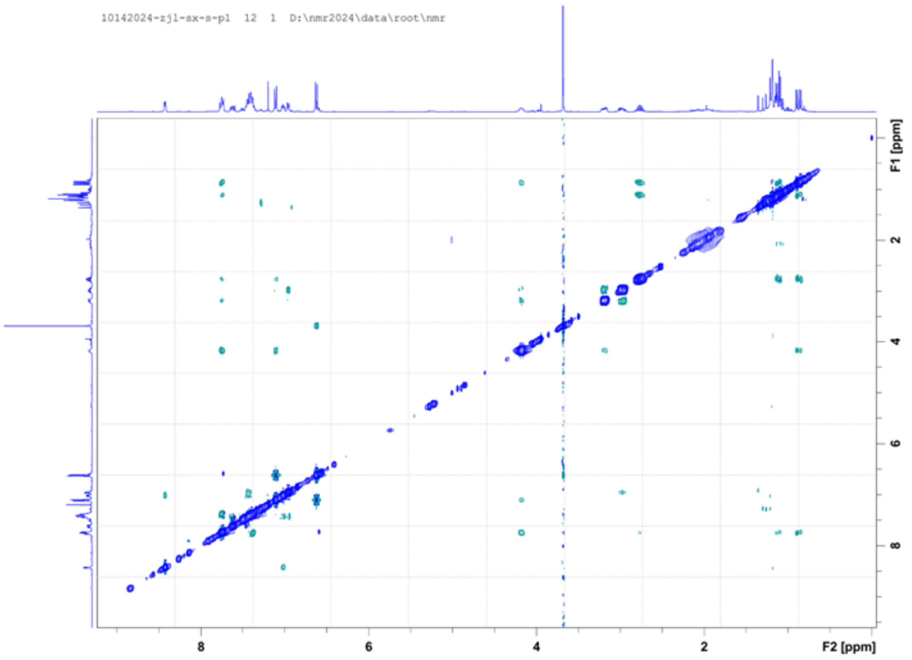

Fig. S24. ^{1}H- ^{1}H NOESY (400 MHz, $CDCl_3$) of *ent*-**47**

3)The absolute configurations of **50** and **53** are determined by NMR**.**

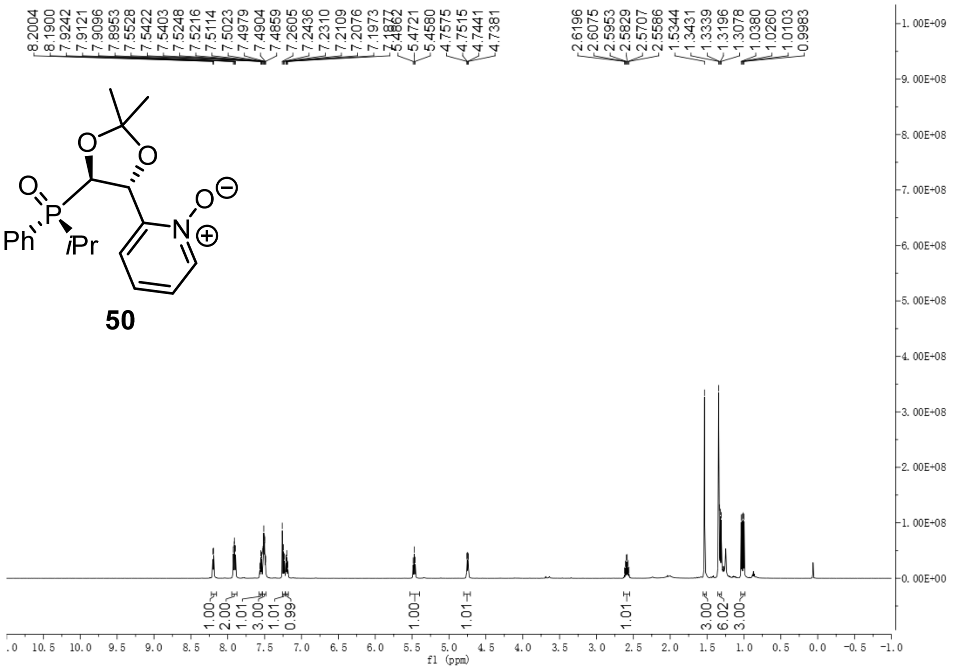

Fig. S25. ^{1}H NMR (600 MHz, $CDCl_3$) of **50**

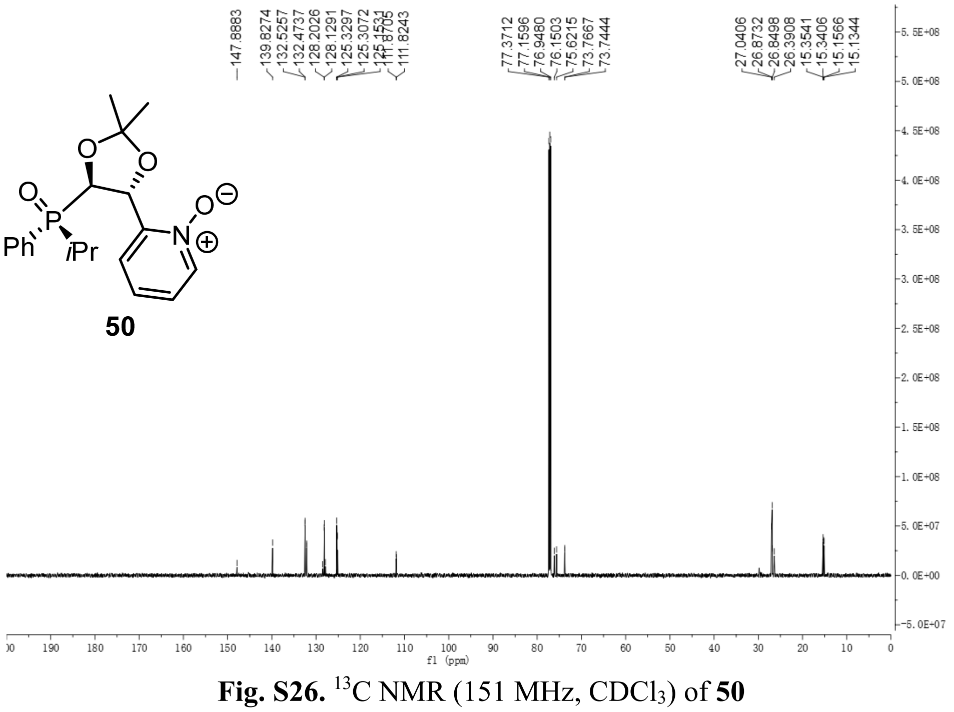

Fig. S26. ^{13}C NMR (151 MHz, $CDCl_3$) of **50**

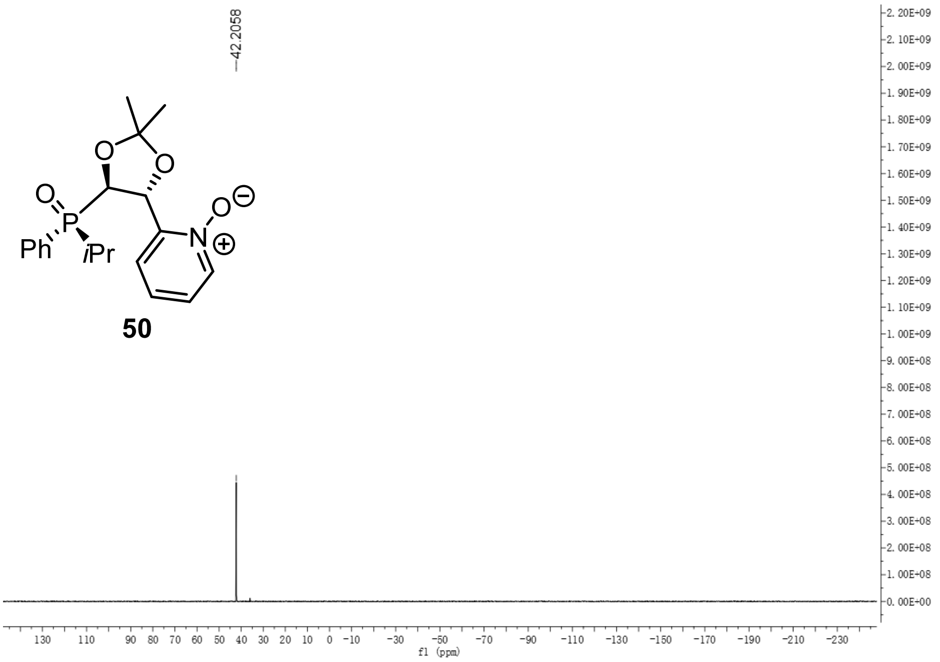

Fig. S27. ^{31}P NMR (243 MHz, $CDCl_3$) of **50**

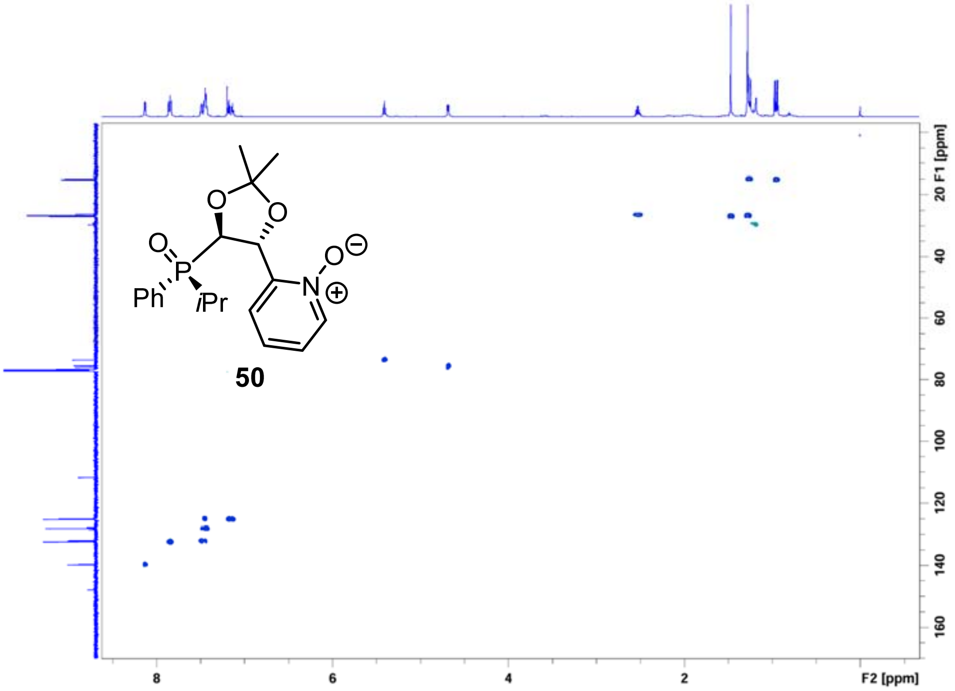

Fig. S28. ^{1}H- ^{13}C HSQC (600 MHz, $CDCl_3$) of **50**

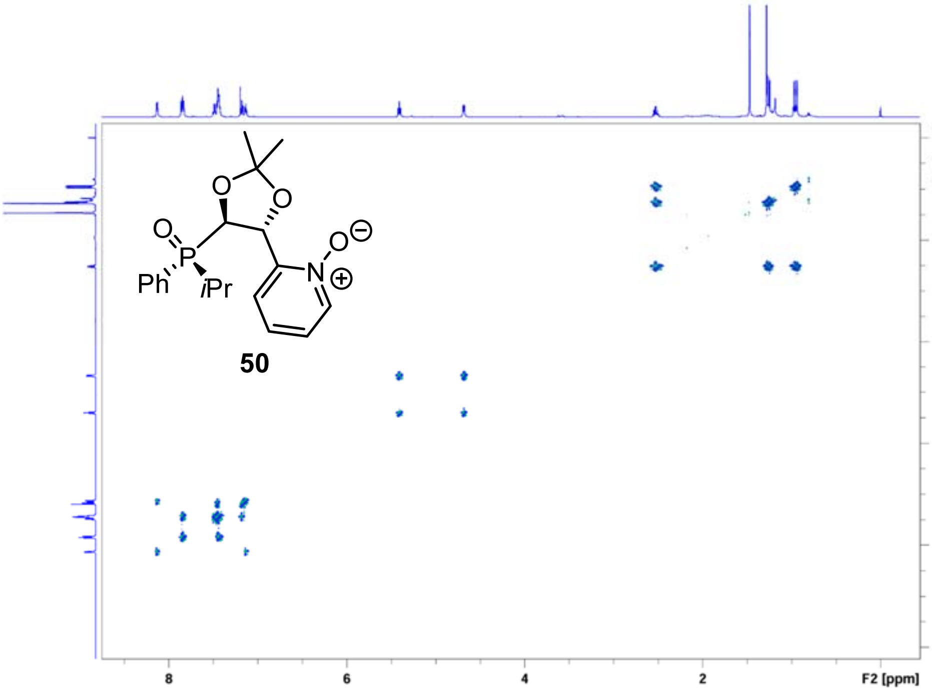

Fig. S29. ^{1}H- ^{1}H COSY (600 MHz, $CDCl_3$) of **50**

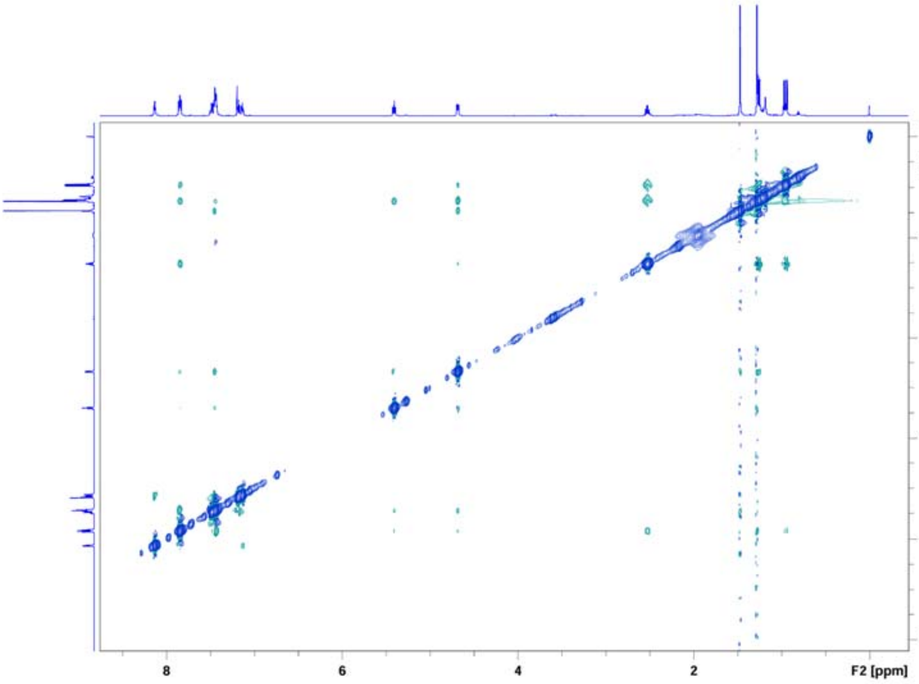

Fig. S30. ^{1}H- ^{1}H NOESY (600 MHz, $CDCl_3$) of **50**

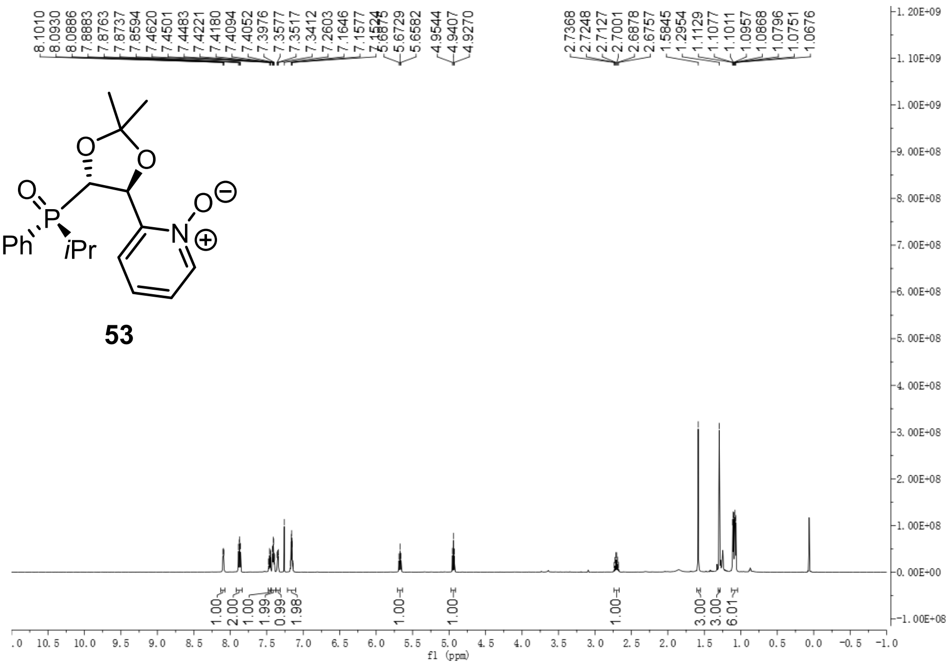

Fig. S31. ^{1}H NMR (600 MHz, $CDCl_3$) of **53**

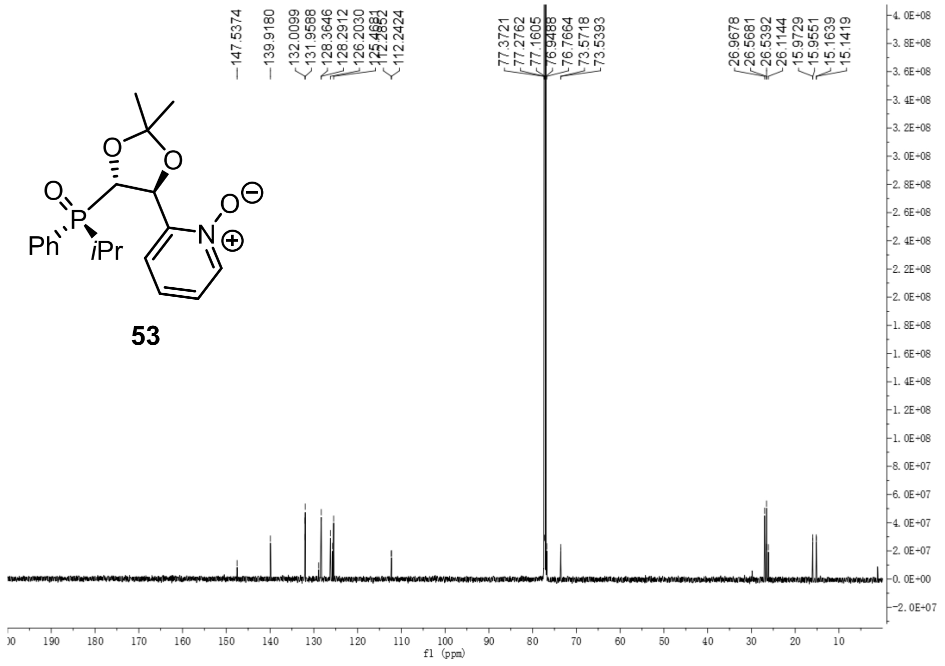

Fig. S32. ^{13}C NMR (151 MHz, $CDCl_3$) of **53**

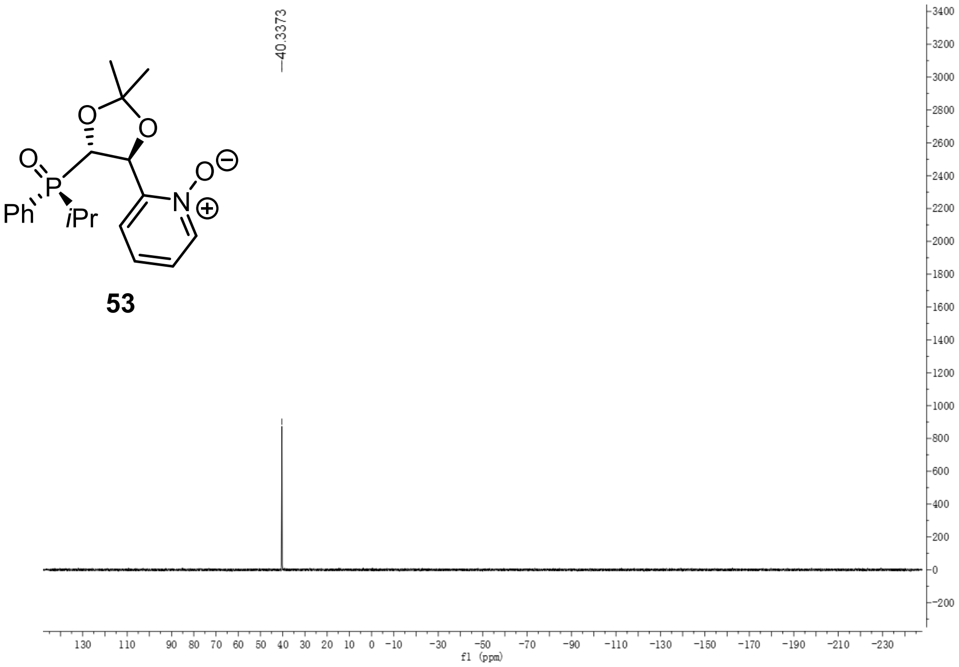

53

Fig. S33. ^{31}P NMR (243 MHz, $CDCl_3$) of **52**

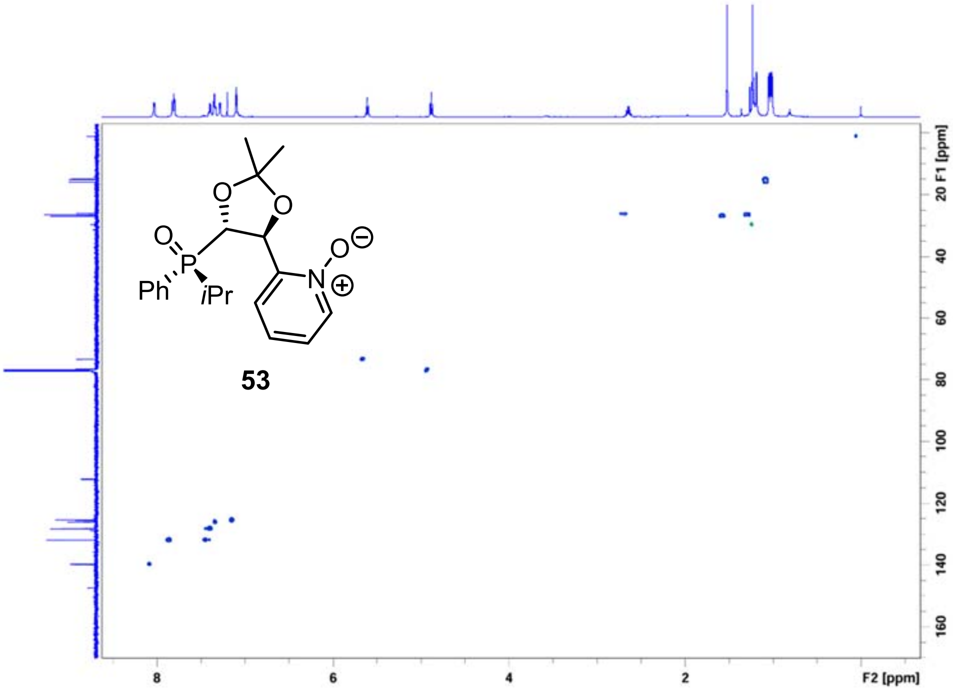

53

Fig. S34. ^{1}H- ^{13}C HSQC (600 MHz, $CDCl_3$) of **53**

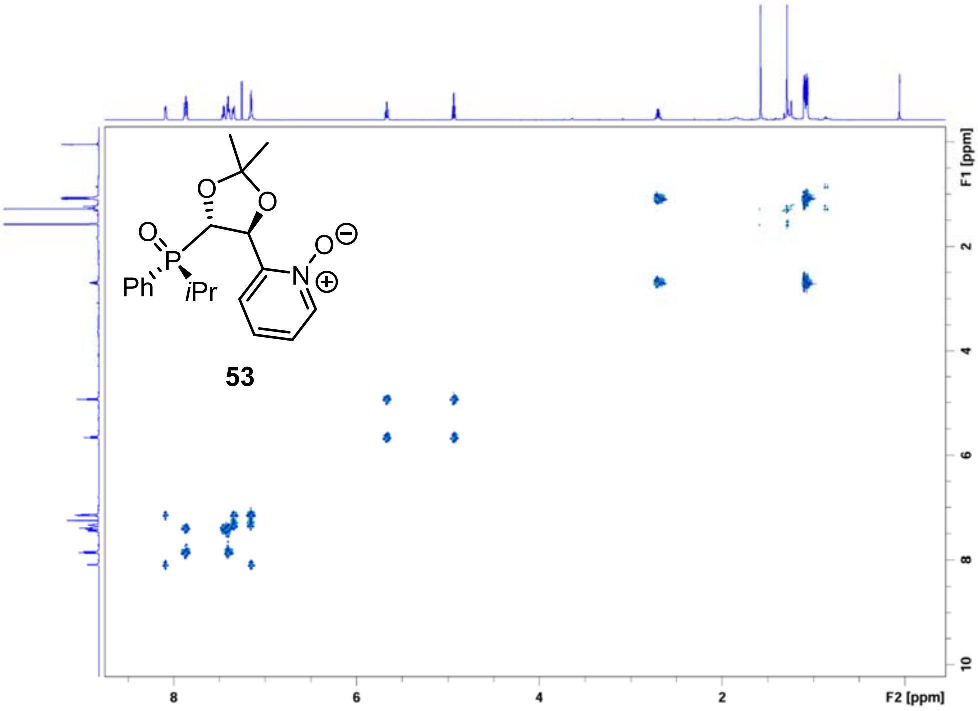

Fig. S35. ^{1}H- ^{1}H COSY (600 MHz, $CDCl_3$) of **53**

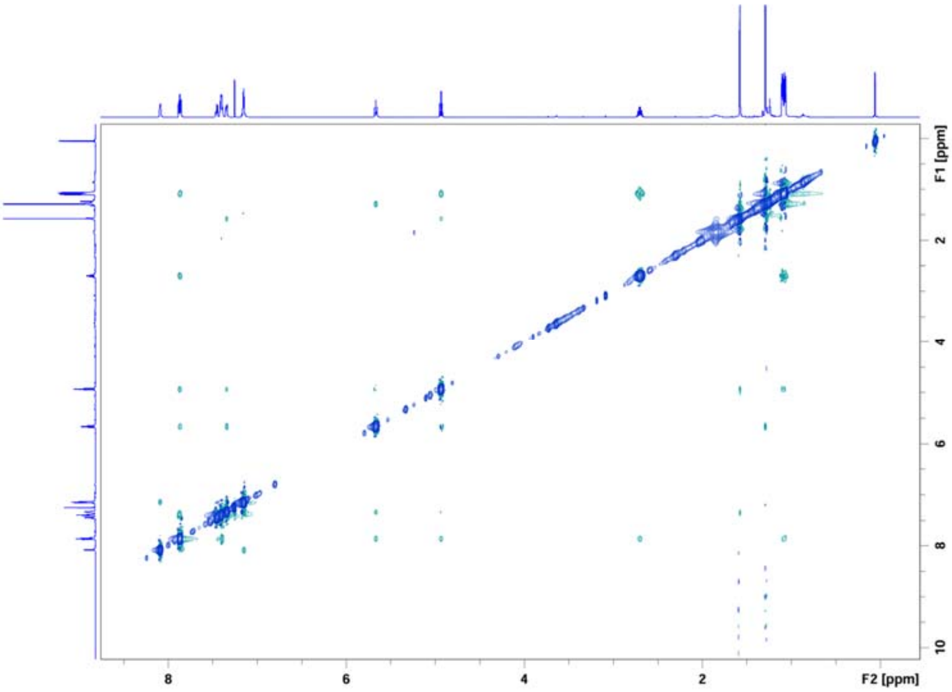

Fig. S36. ^{1}H- ^{1}H NOESY (600 MHz, $CDCl_3$) of **53**

8. Characterization of products

(*R*,*E*)-isopropyl(phenyl)(2-(pyridin-2-yl)vinyl)phosphine oxide (3):

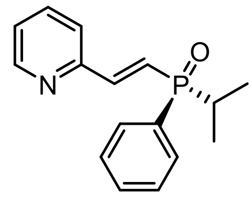

3

colorless oily liquid; 24.9 mg, 92% yield, 96% ee; $[\alpha]_D^{22}$ -20.6 (*c* 1.0, $CHCl_3$); ^{1}H NMR (600 MHz, $CDCl_3$) δ 8.60 (d, *J* = 2.0 Hz, 1H), 7.81 – 7.73 (m, 2H), 7.70 – 7.64 (m, 1H), 7.56 (t, *J* = 17.2 Hz, 1H), 7.49 – 7.42 (m, 3H), 7.37 (dd, *J* = 26.5, 16.8 Hz, 1H), 7.30 (d, *J* = 7.6 Hz, 1H), 7.23 – 7.20 (m, 1H), 2.17 (dq, *J* = 14.4, 7.1 Hz, 1H), 1.21 (dd, *J* = 16.6, 7.1 Hz, 3H), 1.11 (dd, *J* = 16.6, 7.1 Hz, 3H); ^{13}C NMR (101 MHz, $CDCl_3$) δ 152.9 (d, *J* = 16.5 Hz), 150.0, 146.3 (d, *J* = 2.8 Hz), 137.1, 132.0 (d, *J* = 97.2 Hz), 131.6 (d, *J* = 2.6 Hz), 130.8 (d, *J* = 8.7 Hz), 128.7 (d, *J* = 11.2 Hz), 124.8, 124.2, 122.7 (d, *J* = 93.0 Hz), 29.1 (d, *J* = 74.6 Hz), 15.4 (d, *J* = 2.0 Hz), 15.2 (d, *J* = 2.2 Hz); ^{31}P NMR (243 MHz, $CDCl_3$) δ 36.0; HRMS (ESI) m/z 272.1199 (M + H$^+$), calc. for $C_{16}H_{19}NOP$ 272.1199.

The ee was determined by HPLC analysis: CHIRALPAK IE (4.6 mm i.d. x 250 mm); Hexane/2-propanol = 70/30; flow rate 1.0 mL/min; 25 °C; 254 nm; retention time: 28.6 min (minor) and 30.9 min (major).

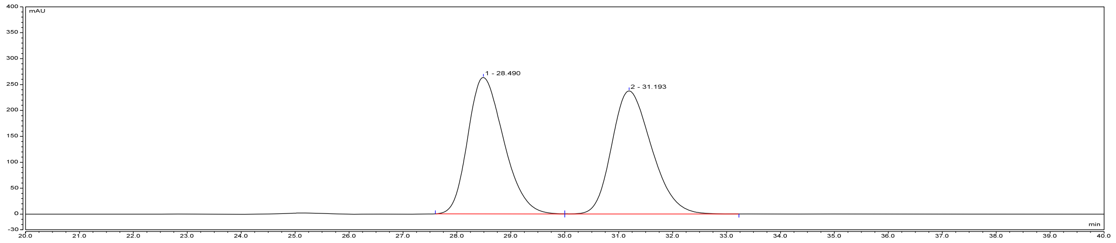

Entry	Retention Time	Area	Height	%Area
1	28.490	203.1791	263.07	50.01
2	31.193	203.1384	237.28	49.99

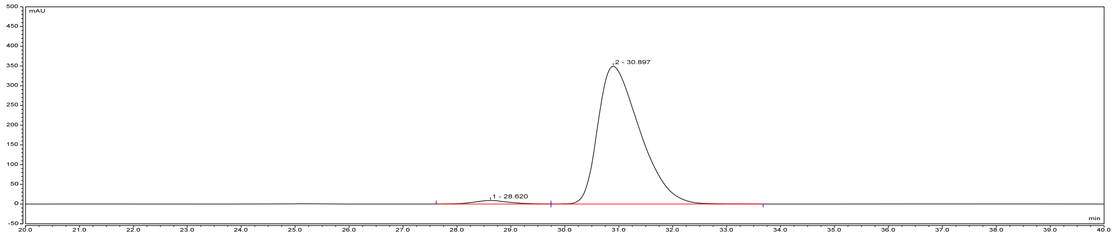

Entry	Retention Time	Area	Height	%Area
1	28.620	6.6059	8.88	2.06
2	30.897	313.3536	348.80	97.94

(*R*,*E*)-(4-fluorophenyl)(isopropyl)(2-(pyridin-2-yl)vinyl)phosphine oxide (4):

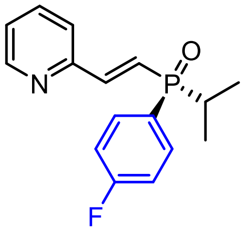

4

colorless oily liquid; 27.0 mg, 93% yield, 91% ee; $[\alpha]_D^{22}$ -18.6 (*c* 1.0, $CHCl_3$); ^1H NMR (600 MHz, $CDCl_3$) δ 8.62 (d, *J* = 4.3 Hz, 1H), 7.81 – 7.76 (m, 2H), 7.71 (t, *J* = 7.6 Hz, 1H), 7.57 (t, *J* = 17.2 Hz, 1H), 7.43 – 7.31 (m, 2H), 7.27 – 7.22 (m, 1H), 7.16 (dd, *J* = 8.6, 7.8 Hz, 2H), 2.16 (dq, *J* = 14.5, 7.1 Hz, 1H), 1.22 (dd, *J* = 16.8, 7.1 Hz, 3H), 1.12 (dd, *J* = 16.8, 7.1 Hz, 3H); ^{13}C NMR (151 MHz, $CDCl_3$) δ 164.9 (d, *J* = 252.8 Hz), 152.6 (d, *J* = 16.7 Hz), 149.8, 146.3, 137.3, 133.2 (d, *J* = 18.4 Hz), 127.7 (d, *J* = 102.1 Hz), 124.9, 124.3, 122.4 (d, *J* = 93.2 Hz), 116.0 (dd, *J* = 21.2, 12.3 Hz), 29.2 (d, *J* = 75.3 Hz), 15.3 (d, *J* = 1.9 Hz), 15.1 (d, *J* = 2.0 Hz); ^{31}P NMR (243 MHz, $CDCl_3$) δ 35.6; ^{19}F NMR (565 MHz, $CDCl_3$) δ -107.4; HRMS (ESI) m/z 290.1105 ($M + H^+$), calc. for $C_{16}H_{18}FNOP$ 290.1105.

The ee was determined by HPLC analysis: CHIRALPAK IC (4.6 mm i.d. x 250 mm); Hexane/2-propanol = 40/60; flow rate 1.0 mL/min; 25 °C; 254 nm; retention time: 9.2 min (minor) and 19.7 min (major).

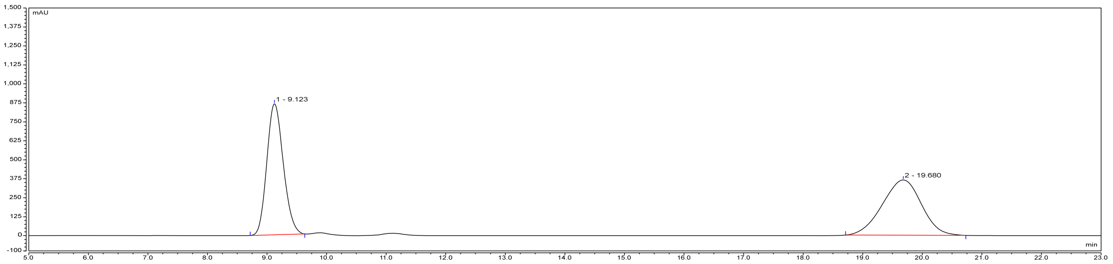

Entry	Retention Time	Area	Height	%Area
1	9.123	276.5011	863.31	49.98
2	19.680	276.7520	363.77	50.02

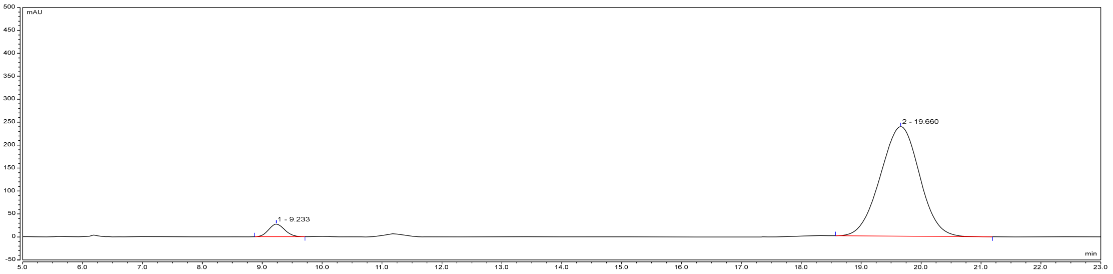

Entry	Retention Time	Area	Height	%Area
1	9.233	8.5023	27.32	4.49
2	19.660	180.8412	238.62	95.51

(*R*,*E*)-(3-fluorophenyl)(isopropyl)(2-(pyridin-2-yl)vinyl)phosphine oxide (5):

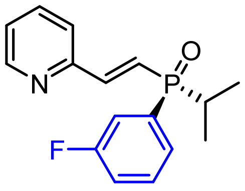

5

colorless oily liquid; 27.6 mg, 95% yield, 88% ee; $[\alpha]_D^{22}$ -18.4 (*c* 1.0, $CHCl_3$); ^{1}H NMR (600 MHz, $CDCl_3$) δ 8.62 (d, *J* = 4.2 Hz, 1H), 7.70 (dd, *J* = 11.2, 4.1 Hz, 1H), 7.61 – 7.42 (m, 4H), 7.35 (dd, *J* = 23.5, 12.8 Hz, 2H), 7.26 – 7.22 (m, 1H), 7.19 (dd, *J* = 12.2, 4.6 Hz, 1H), 2.17 (td, *J* = 14.6, 7.2 Hz, 1H), 1.23 (dd, *J* = 16.8, 7.2 Hz, 3H), 1.12 (dd, *J* = 16.8, 7.2 Hz, 3H); ^{13}C NMR (151 MHz, $CDCl_3$) δ 162.8 (dd, *J* = 250.0, 15.5 Hz), 152.7 (d, *J* = 16.7 Hz), 150.1, 146.9 (d, *J* = 2.9 Hz), 137.2, 134.9 (dd, *J* = 95.1, 5.3 Hz), 130.7 (dd, *J* = 13.1, 7.5 Hz), 126.5 (dd, *J* = 8.5, 3.1 Hz), 125.0, 124.4, 122.0 (d, *J* = 94.2 Hz), 118.8 (d, *J* = 23.6 Hz), 117.8 (dd, *J* = 22.1, 9.1 Hz), 29.1 (d, *J* = 74.9 Hz), 15.4 (d, *J* = 2.0 Hz), 15.1 (d, *J* = 2.4 Hz); ^{31}P NMR (243 MHz, $CDCl_3$) δ 35.3; ^{19}F NMR (565 MHz, $CDCl_3$) δ -111.1; HRMS (ESI) m/z 290.1105 (M + H^+), calc. for $C_{16}H_{18}FNOP$ 290.1105.

The ee was determined by HPLC analysis: CHIRALPAK IC (4.6 mm i.d. x 250 mm); Hexane/2-propanol = 40/60; flow rate 1.0 mL/min; 25 °C; 254 nm; retention time: 10.7 min (minor) and 14.2 min (major).

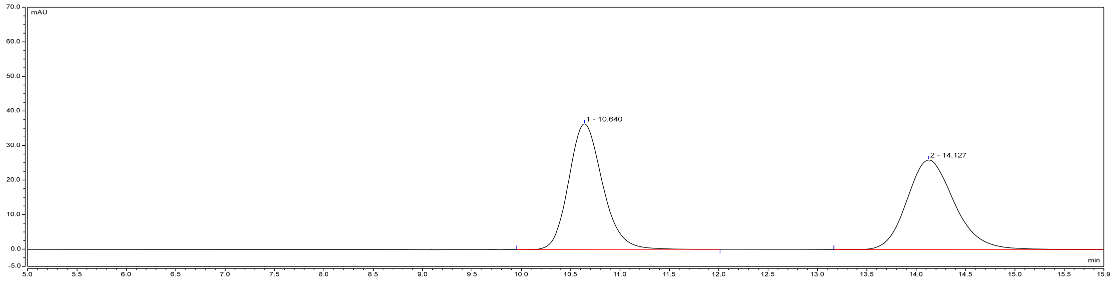

Entry	Retention Time	Area	Height	%Area
1	10.640	14.4443	36.36	50.04
2	14.127	14.4205	25.90	49.96

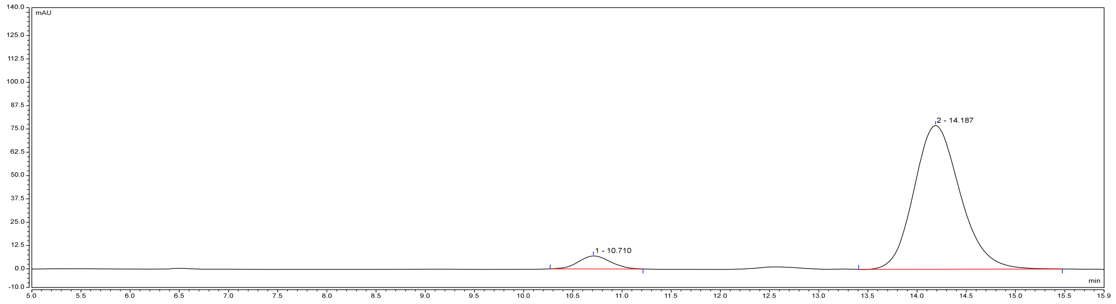

Entry	Retention Time	Area	Height	%Area
1	10.710	2.6039	6.96	5.86
2	14.187	41.8369	77.00	94.14

(*R*,*E*)-(4-chlorophenyl)(isopropyl)(2-(pyridin-2-yl)vinyl)phosphine oxide (6):

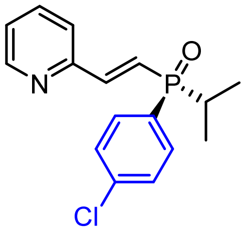

6

colorless oily liquid; 28.1 mg, 92% yield, 90% ee; $[\alpha]_D^{22}$ -15.6 (*c* 1.0, $CHCl_3$); ^1H NMR (600 MHz, $CDCl_3$) δ 8.66 – 8.58 (m, 1H), 7.77 – 7.68 (m, 3H), 7.57 (t, *J* = 17.2 Hz, 1H), 7.48 – 7.44 (m, 2H), 7.42 – 7.31 (m, 2H), 7.28 – 7.23 (m, 1H), 2.16 (dq, *J* = 14.5, 7.1 Hz, 1H), 1.23 (ddd, *J* = 16.8, 7.1, 1.5 Hz, 3H), 1.12 (ddd, *J* = 16.8, 7.1, 1.6 Hz, 3H); ^{13}C NMR (151 MHz, $CDCl_3$) δ 152.7 (d, *J* = 16.5 Hz), 150.1, 146.8 (d, *J* = 2.3 Hz), 138.2 (d, *J* = 3.2 Hz), 137.2, 132.3 (d, *J* = 9.3 Hz), 130.5 (d, *J* = 97.4 Hz), 129.1 (d, *J* = 11.9 Hz), 124.9, 124.4, 122.0 (d, *J* = 93.8 Hz), 29.2 (d, *J* = 75.0 Hz), 15.4 (d, *J* = 2.5 Hz), 15.2 (d, *J* = 2.1 Hz); ^{31}P NMR (243 MHz, $CDCl_3$) δ 35.7; HRMS (ESI) m/z 306.0810 (M + H^+), calc. for $C_{16}H_{18}ClNOP$ 306.0809. The ee was determined by HPLC analysis: CHIRALPAK IE (4.6 mm i.d. x 250 mm); Hexane/2-propanol = 40/60; flow rate 1.0 mL/min; 25 °C; 254 nm; retention time: 9.5 min (minor) and 24.8 min (major).

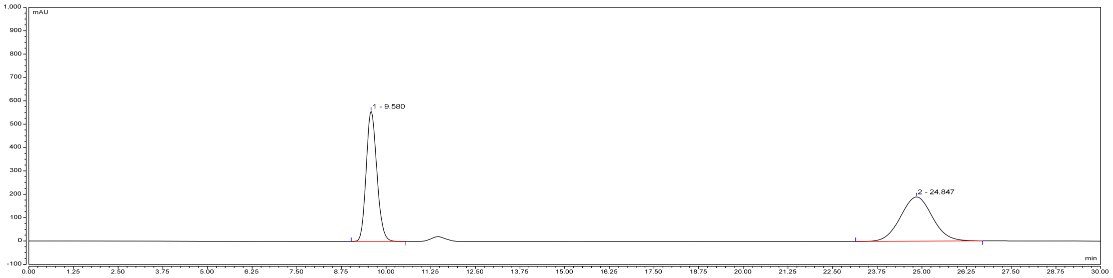

Entry	Retention Time	Area	Height	%Area
1	9.580	193.5195	556.66	49.74
2	24.847	195.5546	189.24	50.26

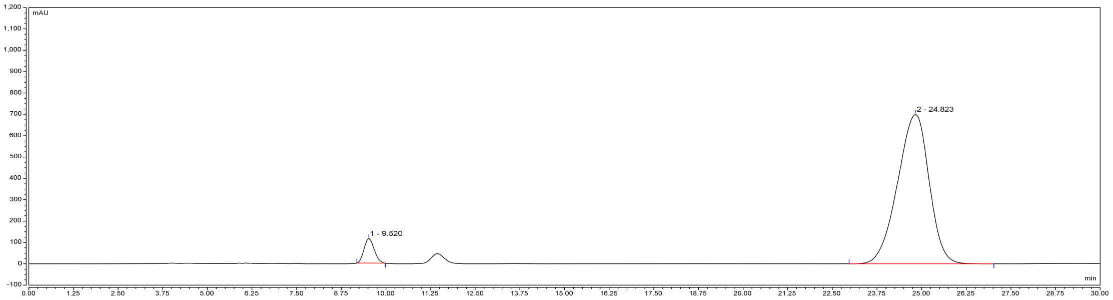

Entry	Retention Time	Area	Height	%Area
1	9.520	38.0606	115.03	5.06
2	24.823	714.3979	699.55	94.94

(*R*,*E*)-(4-bromophenyl)(isopropyl)(2-(pyridin-2-yl)vinyl)phosphine oxide (7):

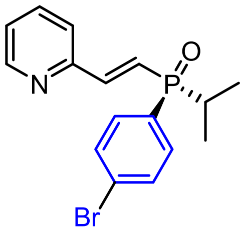
7

white solid; Mp 95.3-96.2 °C; 29.6 mg, 85% yield, 93% ee; $[\alpha]_D^{22}$ +10.4 (*c* 1.0, $CHCl_3$); ^{1}H NMR (600 MHz, $CDCl_3$) δ 8.60 (d, *J* = 4.6 Hz, 1H), 7.68 (td, *J* = 7.7, 1.7 Hz, 1H), 7.65 – 7.51 (m, 5H), 7.37 – 7.29 (m, 2H), 7.24 – 7.21 (m, 1H), 2.19 – 2.09 (m, 1H), 1.21 (dd, *J* = 16.8, 7.1 Hz, 3H), 1.10 (dd, *J* = 16.8, 7.1 Hz, 3H); ^{13}C NMR (151 MHz, $CDCl_3$) δ 152.7 (d, *J* = 16.5 Hz), 150.0, 146.8 (d, *J* = 3.0 Hz), 137.2, 132.4 (d, *J* = 9.2 Hz), 132.0 (d, *J* = 11.9 Hz), 131.0 (d, *J* = 96.9 Hz), 126.8 (d, *J* = 3.2 Hz), 124.9, 124.4, 122.0 (d, *J* = 94.2 Hz), 29.1 (d, *J* = 74.9 Hz), 15.3 (d, *J* = 2.4 Hz), 15.1 (d, *J* = 2.8 Hz); ^{31}P NMR (243 MHz, $CDCl_3$) δ 35.8; HRMS (ESI) m/z 350.0304 (M + H^+), calc. for $C_{16}H_{18}BrNOP$ 350.0304.

The ee was determined by HPLC analysis: CHIRALPAK IC (4.6 mm i.d. x 250 mm); Hexane/2-propanol = 50/50; flow rate 2.0 mL/min; 25 °C; 254 nm; retention time: 5.8 min (minor) and 16.1 min (major).

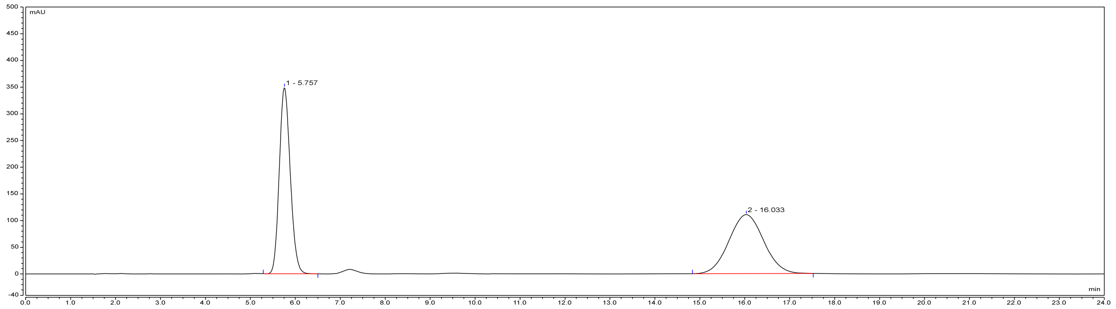

Entry	Retention Time	Area	Height	%Area
1	5.757	98.9447	348.65	49.97
2	16.033	99.0792	110.49	50.03

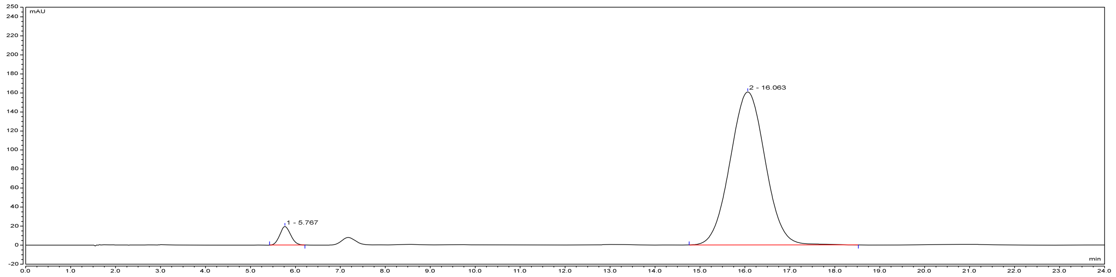

Entry	Retention Time	Area	Height	%Area
1	5.767	5.4607	19.37	3.62
2	16.063	145.4570	160.69	96.38

(*R*,*E*)-isopropyl(2-(pyridin-2-yl)vinyl)(*p*-tolyl)phosphine oxide (8):

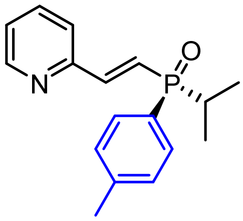

8

colorless oily liquid; 25.9 mg, 91% yield, 93% ee; $[\alpha]_D^{22}$ -21.6 (*c* 1.0, $CHCl_3$); ^{1}H NMR (600 MHz, $CDCl_3$) δ 8.62 (d, *J* = 3.9 Hz, 1H), 7.71 – 7.64 (m, 3H), 7.56 (t, *J* = 17.2 Hz, 1H), 7.41 – 7.31 (m, 2H), 7.28 (dd, *J* = 8.0, 2.3 Hz, 2H), 7.23 (ddd, *J* = 7.6, 4.8, 0.9 Hz, 1H), 2.38 (s, 3H), 2.20 – 2.14 (m, 1H), 1.22 (dd, *J* = 16.6, 7.2 Hz, 3H), 1.12 (dd, *J* = 16.6, 7.2 Hz, 3H); ^{13}C NMR (151 MHz, $CDCl_3$) δ 153.1 (d, *J* = 16.3 Hz), 150.0, 146.1 (d, *J* = 2.3 Hz), 142.1 (d, *J* = 2.8 Hz), 137.2, 130.9 (d, *J* = 8.9 Hz), 129.5 (d, *J* = 11.9 Hz), 128.6 (d, *J* = 99.3 Hz), 124.8, 124.2, 123.0 (d, *J* = 92.8 Hz), 29.2 (d, *J* = 74.8 Hz), 21.7, 15.5 (d, *J* = 2.5 Hz), 15.3 (d, *J* = 2.1 Hz); ^{31}P NMR (243 MHz, $CDCl_3$) δ 36.2; HRMS (ESI) m/z 286.1356 (M + H^+), calc. for $C_{17}H_{21}NOP$ 286.1357.

The ee was determined by HPLC analysis: CHIRALPAK AD-H (4.6 mm i.d. x 250 mm); Hexane/2-propanol = 80/20; flow rate 1.0 mL/min; 25 °C; 254 nm; retention time: 13.4 min (minor) and 15.4 min (major).

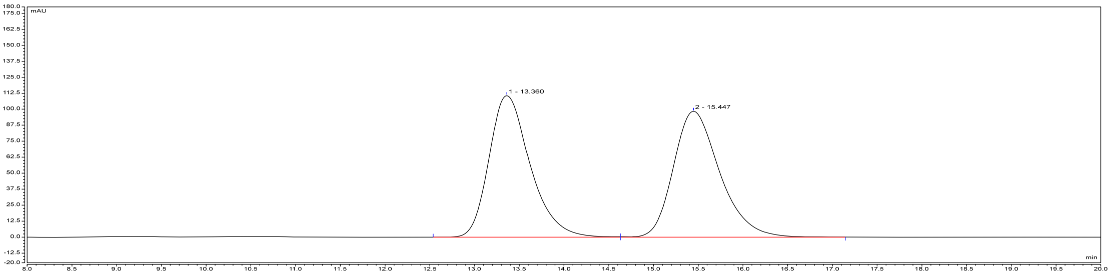

Entry	Retention Time	Area	Height	%Area
1	13.360	58.9391	110.77	49.97
2	15.447	59.0028	98.36	50.03

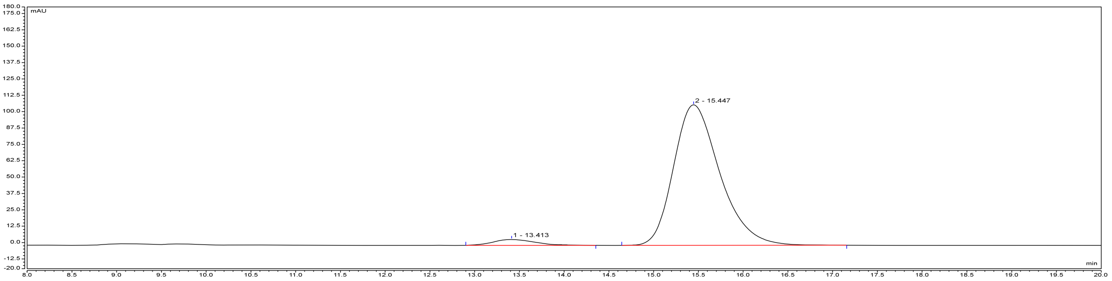

Entry	Retention Time	Area	Height	%Area
1	13.413	2.3518	4.49	3.53
2	15.447	64.1910	107.25	96.47

(*R*,*E*)-isopropyl(2-(pyridin-2-yl)vinyl)(*m*-tolyl)phosphine oxide (9):

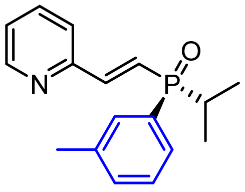

9

colorless oily liquid; 24.3 mg, 85% yield, 95% ee; $[\alpha]_D^{22}$ -19.2 (*c* 1.0, $CHCl_3$); ^1H NMR (600 MHz, $CDCl_3$) δ 8.63 (d, *J* = 4.5 Hz, 1H), 7.72 (t, *J* = 7.6 Hz, 1H), 7.63 (d, *J* = 11.4 Hz, 1H), 7.61 – 7.53 (m, 2H), 7.43 (dd, *J* = 25.6, 17.4 Hz, 1H), 7.37 – 7.33 (m, 2H), 7.30 (d, *J* = 7.4 Hz, 1H), 7.27 – 7.24 (m, 1H), 2.39 (s, 3H), 2.22 – 2.15 (m, 1H), 1.23 (dd, *J* = 16.6, 7.2 Hz, 3H), 1.13 (dd, *J* = 16.6, 7.2 Hz, 3H); ^{13}C NMR (151 MHz, $CDCl_3$) δ 152.8 (d, *J* = 16.3 Hz), 149.8, 145.8 (d, *J* = 9.3 Hz), 138.6 (d, *J* = 11.1 Hz), 137.5, 132.5 (d, *J* = 2.4 Hz), 132.0, 131.4 (d, *J* = 8.2 Hz), 128.6 (d, *J* = 12.1 Hz), 127.9 (d, *J* = 9.1 Hz), 125.0, 124.3, 29.1 (d, *J* = 74.1 Hz), 21.6, 15.5 (d, *J* = 2.1 Hz), 15.2 (d, *J* = 2.1 Hz); ^{31}P NMR (243 MHz, $CDCl_3$) δ 36.1; HRMS (ESI) m/z 273.1356 (M + H^+), calc. for $C_{17}H_{21}NOP$ 273.1357.

The ee was determined by HPLC analysis: CHIRALPAK IC (4.6 mm i.d. x 250 mm); Hexane/2-propanol = 40/60; flow rate 1.0 mL/min; 25 °C; 254 nm; retention time: 11.6 min (minor) and 20.0 min (major).

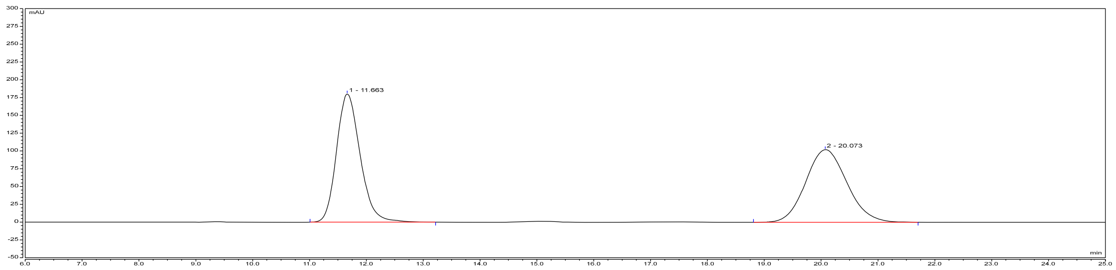

Entry	Retention Time	Area	Height	%Area
1	11.663	86.8915	180.10	50.36
2	20.073	85.6610	101.98	49.64

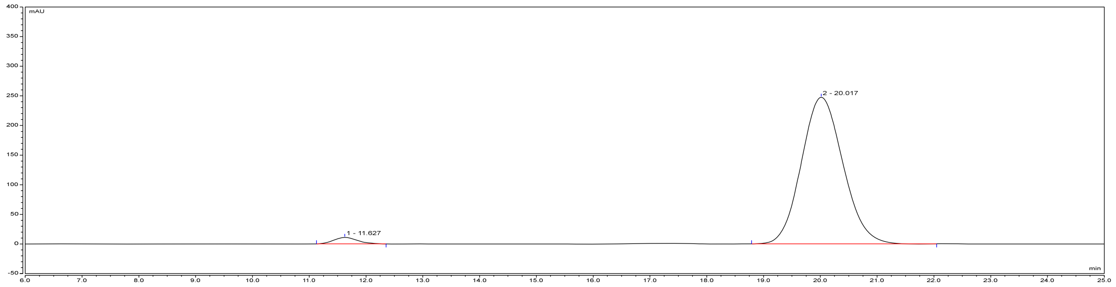

Entry	Retention Time	Area	Height	%Area
1	11.627	5.1425	10.82	2.39
2	20.017	209.9137	247.32	97.61

(*R*,*E*)-(3,5-dimethylphenyl)(isopropyl)(2-(pyridin-2-yl)vinyl)phosphine oxide (10):

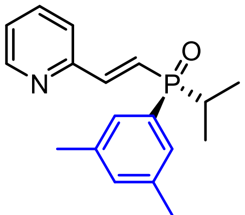

10

white solid; Mp 52.1-52.8 °C; 25.8 mg, 86% yield, 94% ee; $[\alpha]_D^{22}$ +4.2 (*c* 1.0, $CHCl_3$); ^{1}H NMR (600 MHz, $CDCl_3$) δ 8.62 (d, *J* = 4.1 Hz, 1H), 7.68 (td, *J* = 7.7, 1.7 Hz, 1H), 7.56 (t, *J* = 17.1 Hz, 1H), 7.40 – 7.30 (m, 4H), 7.23 (dd, *J* = 7.5, 4.8 Hz, 1H), 7.11 (s, 1H), 2.33 (s, 6H), 2.20 – 2.13 (m, 1H), 1.23 (dd, *J* = 16.6, 7.2 Hz, 3H), 1.12 (dd, *J* = 16.6, 7.2 Hz, 3H); ^{13}C NMR (151 MHz, $CDCl_3$) δ 153.1 (d, *J* = 16.4 Hz), 150.0, 146.0 (d, *J* = 2.7 Hz), 138.4 (d, *J* = 12.0 Hz), 137.2, 133.4 (d, *J* = 2.3 Hz), 131.6 (d, *J* = 96.8 Hz), 128.4 (d, *J* = 8.7 Hz), 124.9, 124.2, 123.1 (d, *J* = 92.4 Hz), 29.0 (d, *J* = 74.5 Hz), 21.4, 15.5 (d, *J* = 2.0 Hz), 15.2 (d, *J* = 2.1 Hz); ^{31}P NMR (243 MHz, $CDCl_3$) δ 36.1; HRMS (ESI) m/z 300.1512 (M + H^+), calc. for $C_{18}H_{23}NOP$ 300.1514.

The ee was determined by HPLC analysis: CHIRALPAK IC (4.6 mm i.d. x 250 mm); Hexane/2-propanol = 40/60; flow rate 1.0 mL/min; 25 °C; 254 nm; retention time: 8.5 min (minor) and 16.0 min (major).

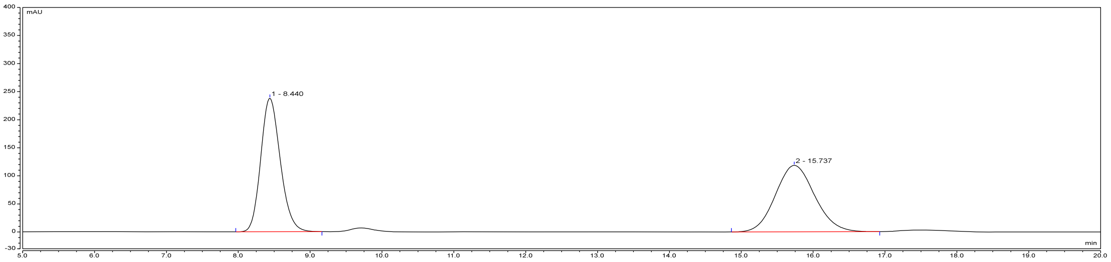

Entry	Retention Time	Area	Height	%Area
1	8.440	75.0753	237.79	50.02
2	15.737	75.0103	118.36	49.98

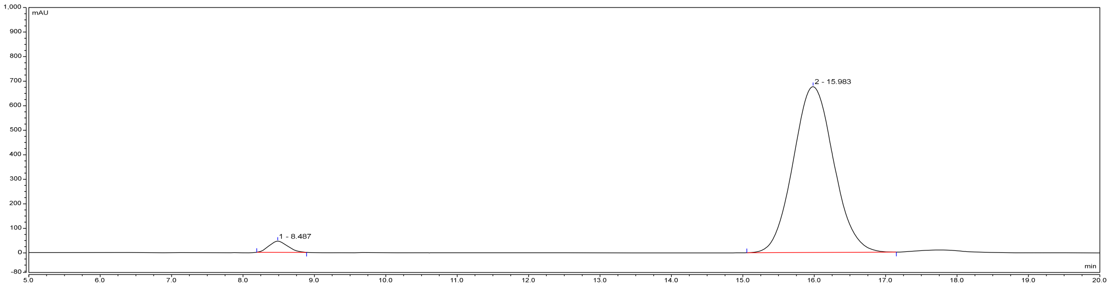

Entry	Retention Time	Area	Height	%Area
1	8.487	13.7465	45.27	3.01
2	15.983	443.4669	676.40	96.99

(*R*,*E*)-(4-(tert-butyl)phenyl)(isopropyl)(2-(pyridin-2-yl)vinyl)phosphine oxide (11):

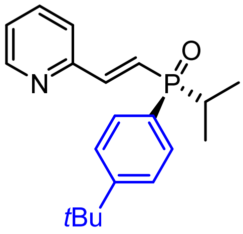

11

white solid; Mp 97.6-98.5 °C; 30.2 mg, 92% yield, 95% ee; $[\alpha]_D^{22}$ -29.3 (*c* 1.0, $CHCl_3$); ^1H NMR (600 MHz, $CDCl_3$) δ 8.58 (d, *J* = 4.7 Hz, 1H), 7.70 – 7.62 (m, 3H), 7.53 (t, *J* = 17.1 Hz, 1H), 7.44 (dd, *J* = 8.3, 2.3 Hz, 2H), 7.34 (dd, *J* = 26.2, 16.8 Hz, 1H), 7.29 (d, *J* = 7.7 Hz, 1H), 7.21 – 7.18 (m, 1H), 2.19 – 2.12 (m, 1H), 1.27 (s, 9H), 1.20 (dd, *J* = 16.6, 7.2 Hz, 3H), 1.11 (dd, *J* = 16.6, 7.2 Hz, 3H); ^{13}C NMR (151 MHz, $CDCl_3$) δ 155.0 (d, *J* = 2.2 Hz), 153.0 (d, *J* = 16.4 Hz), 145.0, 145.9 (d, *J* = 2.8 Hz), 137.1, 130.7 (d, *J* = 8.9 Hz), 128.6 (d, *J* = 99.7 Hz), 125.6 (d, *J* = 11.3 Hz), 124.7, 124.1, 123.1 (d, *J* = 92.7 Hz), 35.0, 31.2, 29.0 (d, *J* = 74.8 Hz), 15.5 (d, *J* = 2.0 Hz), 15.2 (d, *J* = 2.1 Hz); ^{31}P NMR (243 MHz, $CDCl_3$) δ 35.8; HRMS (ESI) m/z 328.1825 ($M + H^+$), calc. for $C_{20}H_{27}NOP$ 328.1825.

The ee was determined by HPLC analysis: CHIRALPAK AD-H (4.6 mm i.d. x 250 mm); Hexane/2-propanol = 80/20; flow rate 1.0 mL/min; 25 °C; 254 nm; retention time: 7.4 min (minor) and 9.5 min (major).

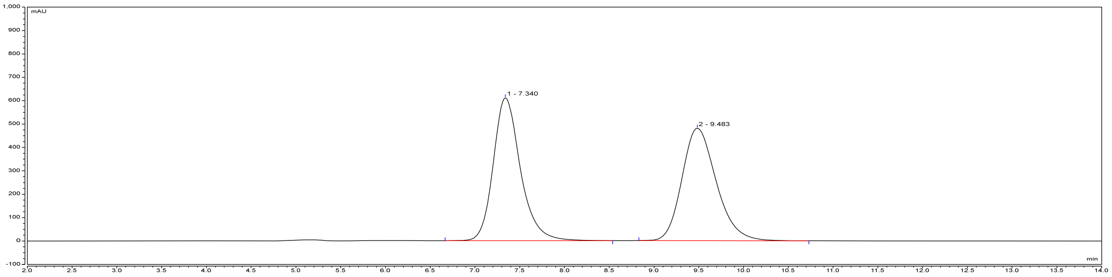

Entry	Retention Time	Area	Height	%Area
1	7.340	215.1346	610.80	50.18
2	9.483	213.5646	480.61	49.82

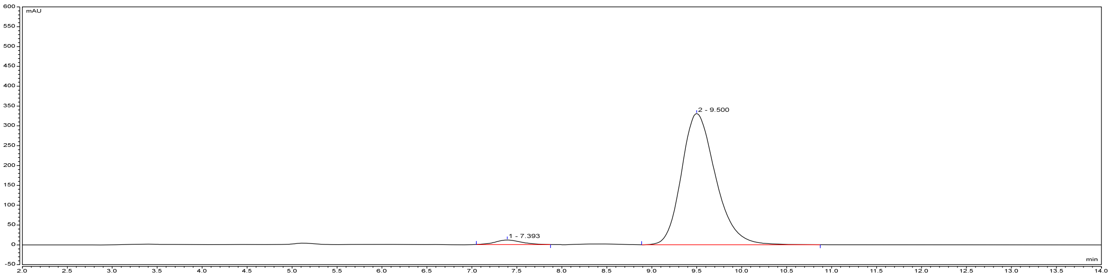

Entry	Retention Time	Area	Height	%Area
1	7.393	3.7910	11.42	2.55
2	9.500	145.0153	330.39	97.45

(*R*,*E*)-[1,1'-biphenyl]-4-yl(isopropyl)(2-(pyridin-2-yl)vinyl)phosphine oxide (12):

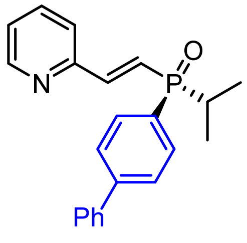

12

white solid; Mp 145.0-145.5 °C; 30.3 mg, 87% yield, 90% ee; $[\alpha]_D^{22}$ +67.8 (*c* 1.0, $CHCl_3$); ^1H NMR (400 MHz, $CDCl_3$) δ 8.62 (d, *J* = 4.6 Hz, 1H), 7.85 (dd, *J* = 10.4, 8.3 Hz, 2H), 7.71 – 7.65 (m, 3H), 7.61 – 7.55 (m, 3H), 7.48 – 7.30 (m, 5H), 7.25 – 7.20 (m, 1H), 2.28 – 2.15 (m, 1H), 1.26 (dd, *J* = 16.7, 7.1 Hz, 3H), 1.16 (dd, *J* = 16.7, 7.1 Hz, 3H); ^{13}C NMR (101 MHz, $CDCl_3$) δ 152.9 (d, *J* = 16.5 Hz), 150.0, 146.3 (d, *J* = 2.7 Hz), 144.4 (d, *J* = 2.6 Hz), 140.1, 137.1, 131.4 (d, *J* = 9.0 Hz), 130.6 (d, *J* = 97.9 Hz), 129.0, 128.1, 127.4 (d, *J* = 9.9 Hz), 127.3, 124.8, 124.2, 122.8 (d, *J* = 93.1 Hz), 29.2 (d, *J* = 74.7 Hz), 15.5 (d, *J* = 2.2 Hz), 15.2 (d, *J* = 2.6 Hz); ^{31}P NMR (162 MHz, $CDCl_3$) δ 35.9; HRMS (ESI) m/z 348.1512 (M + H^+), calc. for $C_{22}H_{23}NOP$ 348.1513.

The ee was determined by HPLC analysis: CHIRALPAK IC (4.6 mm i.d. x 250 mm); Hexane/2-propanol = 40/60; flow rate 1.0 mL/min; 25 °C; 254 nm; retention time: 17.0 min (minor) and 37.3 min (major).

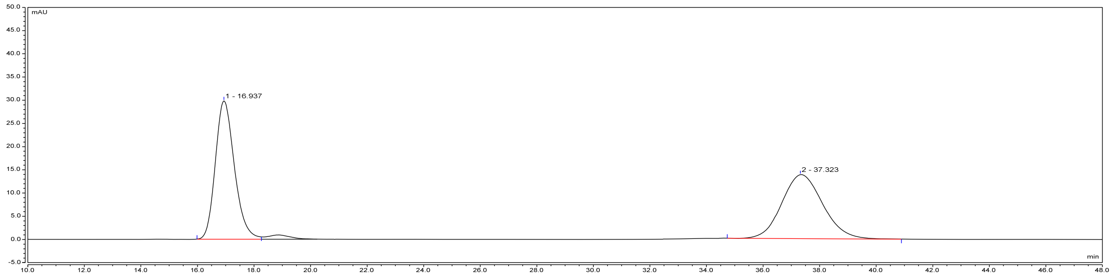

Entry	Retention Time	Area	Height	%Area
1	16.937	23.2909	29.79	50.12
2	37.323	23.1836	13.78	49.88

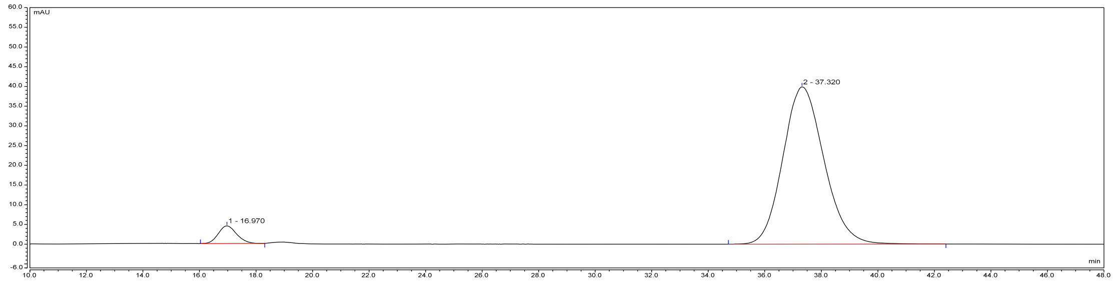

Entry	Retention Time	Area	Height	%Area
1	16.970	3.3544	4.46	4.77
2	37.320	67.0034	39.85	95.23

(*R*,*E*)-isopropyl(3-methoxyphenyl)(2-(pyridin-2-yl)vinyl)phosphine oxide (13):

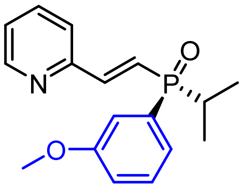

13

colorless oily liquid; 27.9 mg, 93% yield, 93% ee; $[\alpha]_D^{22}$ -54.6 (*c* 1.0, $CHCl_3$); ^1H NMR (600 MHz, $CDCl_3$) δ 8.61 (d, *J* = 4.6 Hz, 1H), 7.68 (t, *J* = 7.6 Hz, 1H), 7.56 (t, *J* = 17.2 Hz, 1H), 7.39 – 7.27 (m, 5H), 7.24 – 7.20 (m, 1H), 7.01 (d, *J* = 8.2 Hz, 1H), 3.82 (s, 3H), 2.16 (dq, *J* = 14.7, 7.3 Hz, 1H), 1.23 (dd, *J* = 16.7, 7.2 Hz, 3H), 1.12 (dd, *J* = 16.7, 7.2 Hz, 3H); ^{13}C NMR (151 MHz, $CDCl_3$) δ 159.8 (d, *J* = 13.6 Hz), 152.9 (d, *J* = 16.5 Hz), 150.0, 146.3 (d, *J* = 2.7 Hz), 137.2, 133.4 (d, *J* = 96.3 Hz), 129.9 (d, *J* = 13.3 Hz), 124.8, 124.2, 122.8 (d, *J* = 8.9 Hz), 122.7 (d, *J* = 93.3 Hz), 117.9 (d, *J* = 2.6 Hz), 115.7 (d, *J* = 9.4 Hz), 55.6, 29.1 (d, *J* = 74.6 Hz), 15.4 (d, *J* = 2.1 Hz), 15.2 (d, *J* = 2.1 Hz); ^{31}P NMR (243 MHz, $CDCl_3$) δ 36.1; HRMS (ESI) m/z 302.1305 ($M + H^+$), calc. for $C_{17}H_{21}NO_2P$ 302.1305.

The ee was determined by HPLC analysis: CHIRALPAK IE (4.6 mm i.d. x 250 mm); Hexane/2-propanol = 40/60; flow rate 1.0 mL/min; 25 °C; 254 nm; retention time: 16.2 min (minor) and 19.8 min (major).

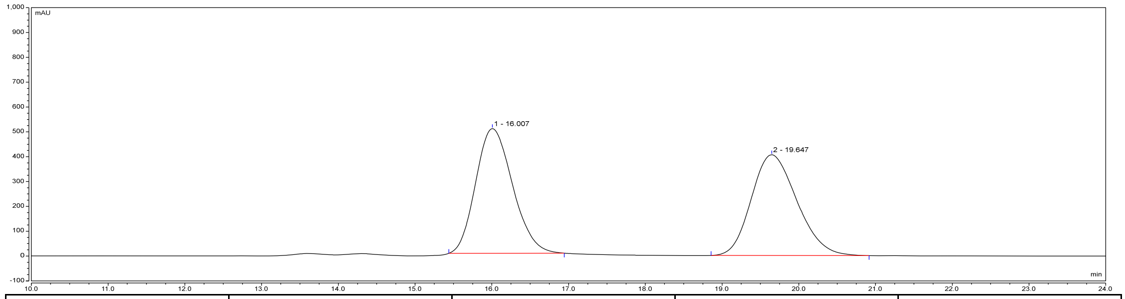

Entry	Retention Time	Area	Height	%Area
1	16.007	279.9908	501.97	50.10
2	19.647	278.9030	405.29	49.90

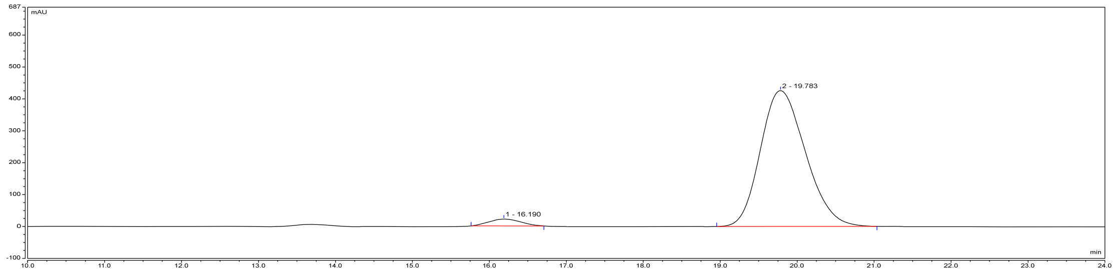

Entry	Retention Time	Area	Height	%Area
1	16.190	10.2670	21.51	3.43
2	19.783	288.6588	425.82	96.57

(*R*,*E*)-benzo[d][1,3]dioxol-5-yl(isopropyl)(2-(pyridin-2-yl)vinyl)phosphine oxide (14);

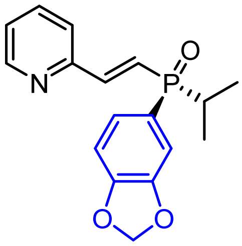

14

colorless oily liquid; 27.3 mg, 87% yield, 92% ee; $[\alpha]_D^{22}$ +5.2 (*c* 1.0, $CHCl_3$); ^{1}H NMR (600 MHz, $CDCl_3$) δ 8.61 – 8.51 (m, 1H), 7.68 – 7.63 (m, 1H), 7.50 (dd, *J* = 25.0, 9.3 Hz, 1H), 7.35 – 7.23 (m, 3H), 7.23 – 7.19 (m, 1H), 7.16 (d, *J* = 10.3 Hz, 1H), 6.86 (dd, *J* = 7.9, 2.2 Hz, 1H), 5.97 (s, 2H), 2.10 (dq, *J* = 14.3, 7.2 Hz, 1H), 1.20 – 1.15 (m, 3H), 1.09 (ddd, *J* = 16.7, 7.2, 1.9 Hz, 3H); ^{13}C NMR (151 MHz, $CDCl_3$) δ 152.8 (d, *J* = 16.5 Hz), 150.6 (d, *J* = 2.9 Hz), 145.0, 148.1 (d, *J* = 16.7 Hz), 146.0 (d, *J* = 2.9 Hz), 137.1, 126.0 (d, *J* = 9.6 Hz), 124.8 (d, *J* = 100.6 Hz), 124.7, 124.2, 122.7 (d, *J* = 93.5 Hz), 110.3 (d, *J* = 11.6 Hz), 108.9 (d, *J* = 14.2 Hz), 101.6, 29.2 (d, *J* = 75.5 Hz), 15.4 (d, *J* = 2.0 Hz), 15.2 (d, *J* = 2.1 Hz); ^{31}P NMR (243 MHz, $CDCl_3$) δ 36.3; HRMS (ESI) m/z 316.1098 (M + H$^+$), calc. for $C_{17}H_{19}NO_3P$ 316.1099.

The ee was determined by HPLC analysis: CHIRALPAK IG (4.6 mm i.d. x 250 mm); Hexane/2-propanol = 40/60; flow rate 1.0 mL/min; 25 °C; 254 nm; retention time: 13.3 min (major) and 15.5 min (minor).

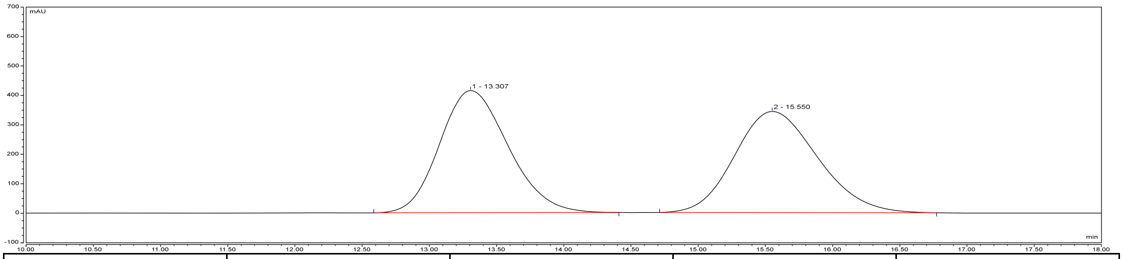

Entry	Retention Time	Area	Height	%Area
1	13.307	245.7027	414.60	49.99
2	15.550	245.8263	343.21	50.01

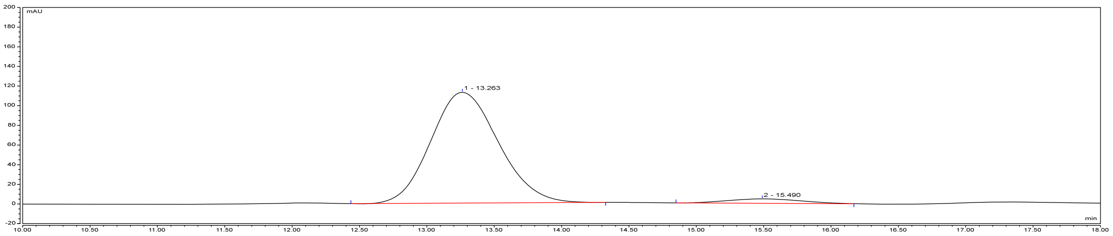

Entry	Retention Time	Area	Height	%Area
1	13.263	65.6440	112.81	95.87
2	15.490	2.8301	4.47	4.13

(*R*,*E*)-isopropyl(naphthalen-2-yl)(2-(pyridin-2-yl)vinyl)phosphine oxide (15):

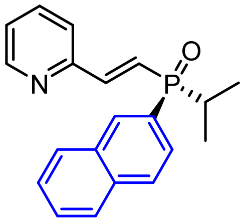

15

colorless oily liquid; 31.8 mg, 99% yield, 93% ee; $[\alpha]_D^{22}$ -100.8 (*c* 1.0, $CHCl_3$); ^1H NMR (600 MHz, $CDCl_3$) δ 8.63 (d, *J* = 3.6 Hz, 1H), 8.41 (d, *J* = 12.7 Hz, 1H), 7.95 – 7.88 (m, 2H), 7.85 (d, *J* = 7.8 Hz, 1H), 7.75 – 7.66 (m, 2H), 7.64 – 7.46 (m, 4H), 7.33 (d, *J* = 7.6 Hz, 1H), 7.26 – 7.22 (m, 1H), 2.32 – 2.22 (m, 1H), 1.28 (dd, *J* = 16.7, 7.1 Hz, 3H), 1.14 (dd, *J* = 16.7, 7.1 Hz, 3H); ^{13}C NMR (151 MHz, $CDCl_3$) δ 152.8 (d, *J* = 16.5 Hz), 149.9, 146.1, 137.4, 134.7 (d, *J* = 1.8 Hz), 132.9 (d, *J* = 7.7 Hz), 132.8 (d, *J* = 12.2 Hz), 129.0 (d, *J* = 97.4 Hz), 128.9, 128.5 (d, *J* = 11.1 Hz), 128.1, 127.9, 127.0, 125.7 (d, *J* = 9.9 Hz), 124.9, 124.3, 123.0 (d, *J* = 93.0 Hz), 29.5 (d, *J* = 74.7 Hz), 15.5 (d, *J* = 1.9 Hz), 15.2 (d, *J* = 2.0 Hz); ^{31}P NMR (243 MHz, $CDCl_3$) δ 36.2; HRMS (ESI) m/z 322.1356 (M + H^+), calc. for $C_{20}H_{21}NOP$ 322.1358. The ee was determined by HPLC analysis: CHIRALPAK IC (4.6 mm i.d. x 250 mm); Hexane/2-propanol = 40/60; flow rate 1.0 mL/min; 25 °C; 254 nm; retention time: 11.7 min (minor) and 20.1 min (major).

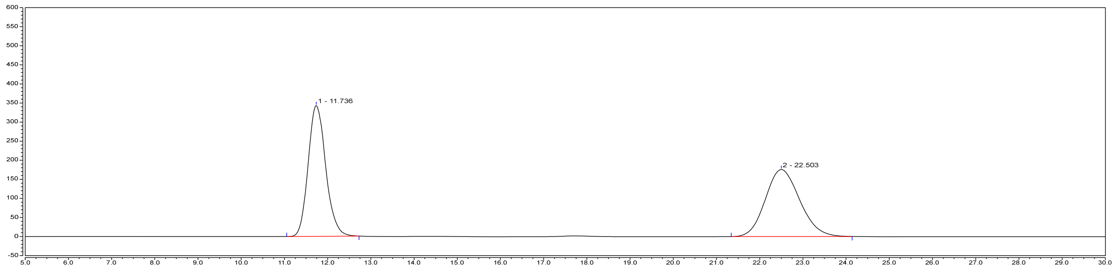

Entry	Retention Time	Area	Height	%Area
1	11.736	162.0558	342.92	50.00
2	22.503	162.0374	175.66	50.00

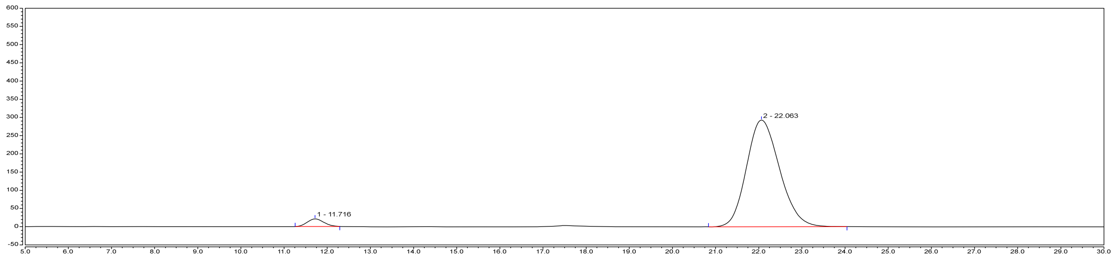

Entry	Retention Time	Area	Height	%Area
1	11.716	9.2190	20.91	3.42
2	22.063	260.2786	292.75	96.58

(*R*,*E*)-benzo[b]thiophen-5-yl(isopropyl)(2-(pyridin-2-yl)vinyl)phosphine oxide (16):

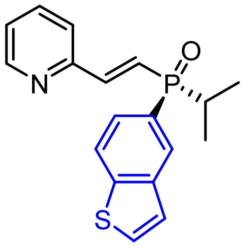

16

white solid; Mp 69.4-69.8 °C; 27.2 mg, 83% yield, 96% ee; $[\alpha]_D^{22}$ -27.6 (*c* 1.0, $CHCl_3$); ^1H NMR (600 MHz, $CDCl_3$) δ 8.63 (d, *J* = 4.4 Hz, 1H), 8.32 (d, *J* = 11.9 Hz, 1H), 7.98 (d, *J* = 8.2 Hz, 1H), 7.71 – 7.65 (m, 2H), 7.60 (t, *J* = 17.2 Hz, 1H), 7.51 (d, *J* = 5.4 Hz, 1H), 7.45 (dd, *J* = 26.5, 16.8 Hz, 1H), 7.40 (d, *J* = 5.4 Hz, 1H), 7.33 (d, *J* = 7.7 Hz, 1H), 7.25 – 7.22 (m, 1H), 2.28 – 2.21 (m, 1H), 1.26 (dd, *J* = 16.7, 7.2 Hz, 3H), 1.14 (dd, *J* = 16.7, 7.2 Hz, 3H); ^{13}C NMR (151 MHz, $CDCl_3$) δ 152.9 (d, *J* = 16.5 Hz), 150.0, 146.2 (d, *J* = 2.8 Hz), 143.0 (d, *J* = 2.9 Hz), 139.5 (d, *J* = 13.1 Hz), 137.2, 127.8, 127.8, 127.1 (d, *J* = 8.7 Hz), 125.2 (d, *J* = 10.7 Hz), 124.9, 124.3, 124.2, 123.0 (d, *J* = 12.5 Hz), 122.8 (d, *J* = 93.3 Hz), 29.3 (d, *J* = 74.8 Hz), 15.5 (d, *J* = 2.1 Hz), 15.3 (d, *J* = 2.1 Hz); ^{31}P NMR (243 MHz, $CDCl_3$) δ 36.9; HRMS (ESI) m/z 328.0920 (M + H^+), calc. for $C_{18}H_{19}NOPS$ 328.0919.

The ee was determined by HPLC analysis: CHIRALPAK IC (4.6 mm i.d. x 250 mm); Hexane/2-propanol = 40/60; flow rate 1.0 mL/min; 25 °C; 254 nm; retention time: 12.7 min (minor) and 29.8 min (major).

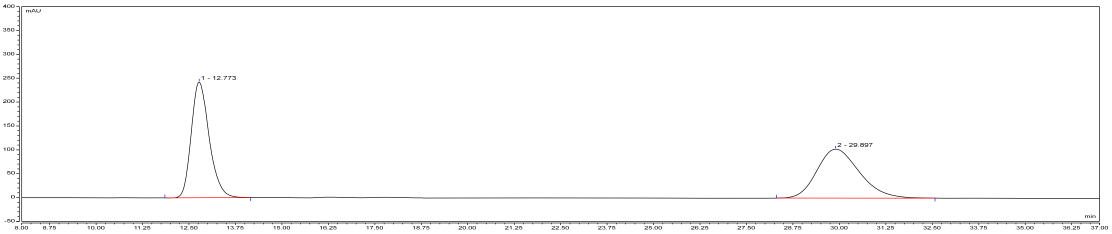

Entry	Retention Time	Area	Height	%Area
1	12.773	135.5780	242.20	49.98
2	29.897	135.6706	102.61	50.02

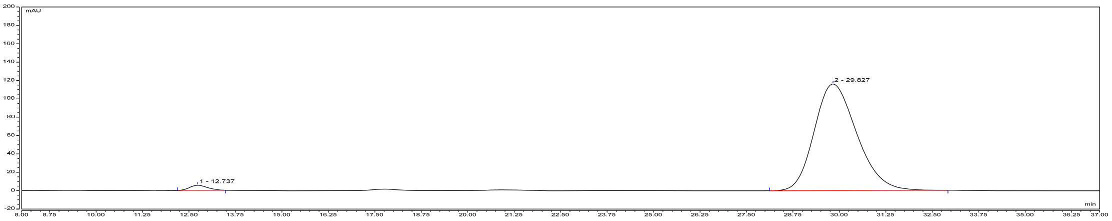

Entry	Retention Time	Area	Height	%Area
1	12.737	3.1298	5.59	1.99
2	29.827	153.8895	116.06	98.01

(*R*,*E*)-pentan-3-yl(phenyl)(2-(pyridin-2-yl)vinyl)phosphine oxide (17):

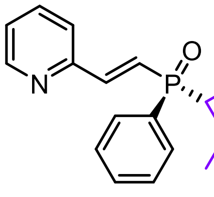

17

colorless oily liquid; 26.9 mg, 90% yield, 96% ee; $[\alpha]_D^{22}$ +7.2 (*c* 1.0, $CHCl_3$); ^1H NMR (600 MHz, $CDCl_3$) δ 8.60 (d, *J* = 4.5 Hz, 1H), 7.77 (ddd, *J* = 8.1, 5.9, 4.0 Hz, 2H), 7.69 (td, *J* = 7.6, 1.5 Hz, 1H), 7.54 (t, *J* = 17.2 Hz, 1H), 7.49 – 7.38 (m, 4H), 7.32 (d, *J* = 7.6 Hz, 1H), 7.23 (dd, *J* = 7.4, 4.9 Hz, 1H), 1.88 – 1.75 (m, 2H), 1.74 – 1.61 (m, 2H), 1.57 – 1.46 (m, 1H), 0.98 (t, *J* = 7.3 Hz, 3H), 0.89 (t, *J* = 7.5 Hz, 3H); ^{13}C NMR (151 MHz, $CDCl_3$) δ 152.8 (d, *J* = 16.3 Hz), 149.6, 145.2, 137.5, 132.9 (d, *J* = 96.4 Hz), 131.5 (d, *J* = 2.7 Hz), 130.7 (d, *J* = 8.7 Hz), 128.7, 128.6, 124.9, 124.2, 42.1 (d, *J* = 72.5 Hz), 19.9 (d, *J* = 44.6 Hz), 12.7 (d, *J* = 9.9 Hz), 12.5 (d, *J* = 9.6 Hz); ^{31}P NMR (243 MHz, $CDCl_3$) δ 35.2. HRMS (ESI) m/z 300.1512 (M + H^+), calc. for $C_{18}H_{23}NOP$ 300.1514.

The ee was determined by HPLC analysis: CHIRALPAK IC (4.6 mm i.d. x 250 mm); Hexane/2-propanol = 40/60; flow rate 1.0 mL/min; 25 °C; 254 nm; retention time: 13.4 min (minor) and 14.8 min (major).

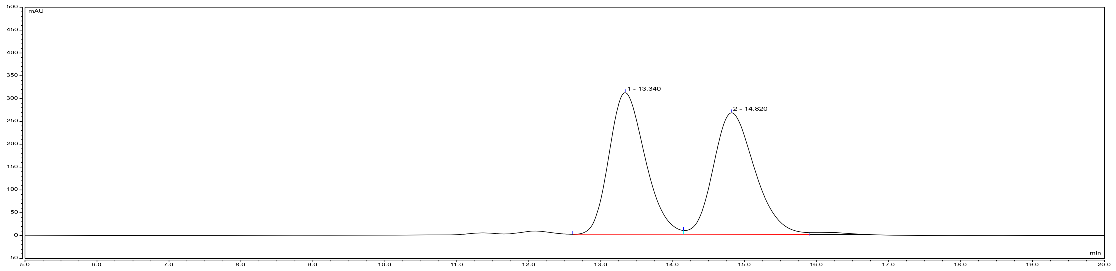

Entry	Retention Time	Area	Height	%Area
1	13.340	180.1933	310.17	50.40
2	14.820	177.3098	266.27	49.60

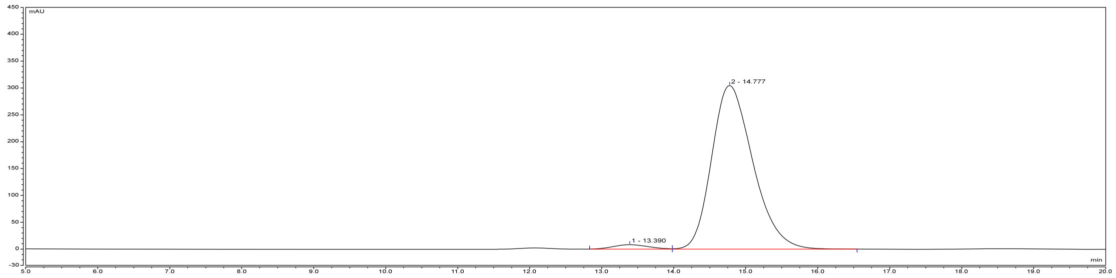

Entry	Retention Time	Area	Height	%Area
1	13.390	4.6135	8.26	2.23
2	14.777	202.4405	304.49	97.77

(*R*,*E*)-isobutyl(phenyl)(2-(pyridin-2-yl)vinyl)phosphine oxide (18):

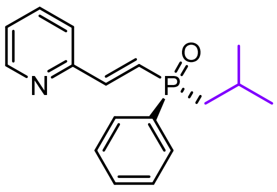

18

colorless oily liquid; 21.4 mg, 75% yield, 86% ee; $[\alpha]_D^{22}$ +18.8 (*c* 1.0, $CHCl_3$); ^{1}H NMR (400 MHz, $CDCl_3$) δ 8.62 (d, *J* = 4.3 Hz, 1H), 7.79 (ddd, *J* = 11.4, 7.6, 1.7 Hz, 2H), 7.70 (td, *J* = 7.7, 1.6 Hz, 1H), 7.56 – 7.44 (m, 4H), 7.37 – 7.21 (m, 3H), 2.23 – 2.09 (m, 1H), 2.01 (dd, *J* = 11.3, 6.6 Hz, 2H), 1.07 (d, *J* = 6.6 Hz, 3H), 1.00 (d, *J* = 6.6 Hz, 3H); ^{13}C NMR (101 MHz, $CDCl_3$) δ 153.1 (d, *J* = 17.2 Hz), 150.0, 144.8, 137.2, 133.6 (d, *J* = 99.5 Hz), 131.7 (d, *J* = 2.6 Hz), 130.4 (d, *J* = 9.2 Hz), 128.8 (d, *J* = 11.6 Hz), 125.4 (t, *J* = 50.1 Hz), 124.7, 124.2, 39.9 (d, *J* = 72.7 Hz), 24.8 (d, *J* = 9.0 Hz), 24.7 (d, *J* = 8.9 Hz), 23.8 (d, *J* = 3.7 Hz); ^{31}P NMR (162 MHz, $CDCl_3$) δ 29.0; HRMS (ESI) m/z 286.1356 (M + H$^+$), calc. for $C_{17}H_{21}NOP$ 286.1355.

The ee was determined by HPLC analysis: CHIRALPAK IC (4.6 mm i.d. x 250 mm); Hexane/2-propanol = 40/60; flow rate 1.0 mL/min; 25 °C; 254 nm; retention time: 13.9 min (minor) and 26.0 min (major).

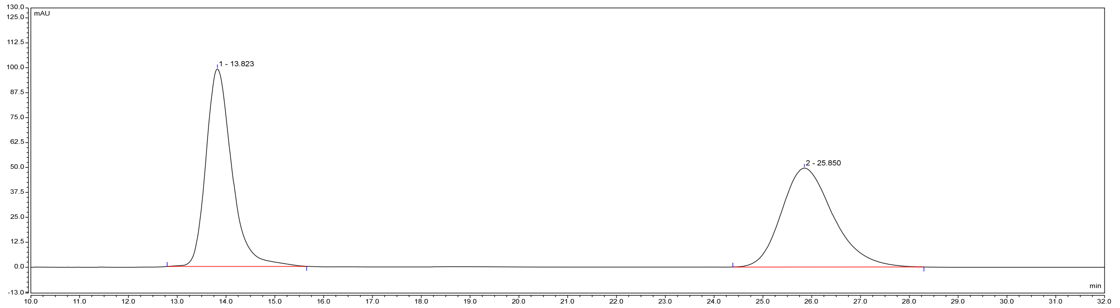

Entry	Retention Time	Area	Height	%Area
1	13.823	60.9427	99.02	50.04
2	25.850	60.8507	49.52	49.96

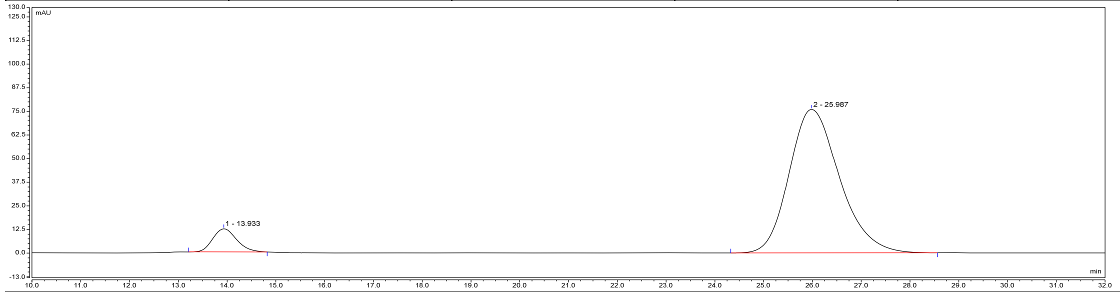

Entry	Retention Time	Area	Height	%Area
1	13.933	6.9096	12.28	7.06
2	25.987	90.9293	75.95	92.94

(*R*,*E*)-neopentyl(phenyl)(2-(pyridin-2-yl)vinyl)phosphine oxide (19):

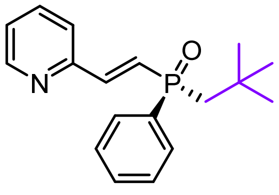

19

white solid; Mp 81.8-82.5 °C; 21.5 mg, 72% yield, 92% ee; $[\alpha]_D^{22}$ +15.6 (*c* 1.0, $CHCl_3$); ^1H NMR (600 MHz, $CDCl_3$) δ 8.57 (d, *J* = 4.2 Hz, 1H), 7.76 (ddd, *J* = 11.3, 7.6, 1.6 Hz, 2H), 7.65 (td, *J* = 7.7, 1.7 Hz, 1H), 7.47 – 7.40 (m, 4H), 7.36 – 7.27 (m, 2H), 7.19 (dd, *J* = 7.1, 4.9 Hz, 1H), 2.08 (d, *J* = 11.1 Hz, 2H), 1.07 (s, 9H); ^{13}C NMR (151 MHz, $CDCl_3$) δ 153.0 (d, *J* = 17.3 Hz), 149.8, 143.6, 137.2, 134.6 (d, *J* = 99.0 Hz), 131.4 (d, *J* = 2.2 Hz), 130.3 (d, *J* = 8.9 Hz), 128.7 (d, *J* = 11.8 Hz), 127.1 (d, *J* = 94.6 Hz), 124.6, 124.0, 44.5 (d, *J* = 71.9 Hz), 32.2 (d, *J* = 4.4 Hz), 31.7 (d, *J* = 6.9 Hz); ^{31}P NMR (243 MHz, $CDCl_3$) δ 27.0; HRMS (ESI) m/z 300.1512 ($M + H^+$), calc. for $C_{18}H_{23}NOP$ 300.1514.

The ee was determined by HPLC analysis: CHIRALPAK AD-H (4.6 mm i.d. x 250 mm); Hexane/2-propanol = 95/5; flow rate 1.0 mL/min; 25 °C; 254 nm; retention time: 31.2 min (minor) and 35.1 min (major).

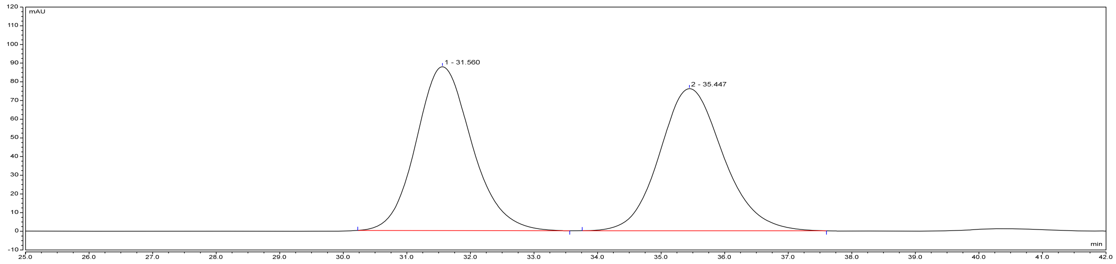

Entry	Retention Time	Area	Height	%Area
1	31.560	89.2937	87.69	50.26
2	35.447	88.3824	76.06	49.74

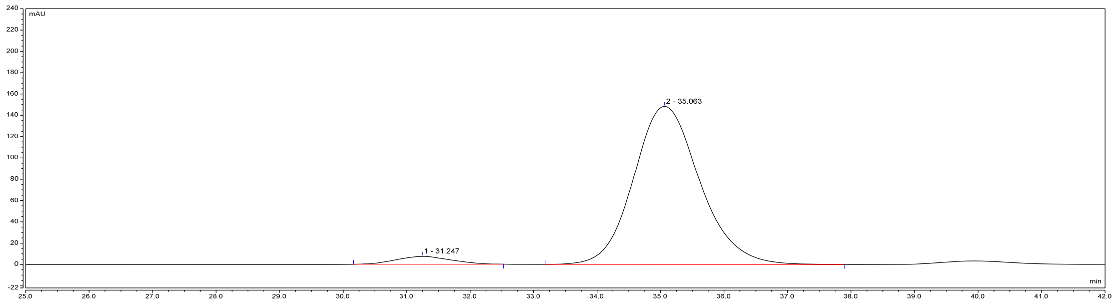

Entry	Retention Time	Area	Height	%Area
1	31.247	7.3009	7.33	3.91
2	35.063	179.3632	148.26	96.09

(*R*,*E*)-cyclobutyl(phenyl)(2-(pyridin-2-yl)vinyl)phosphine oxide (20):

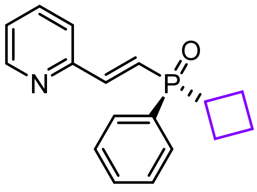

20

colorless oily liquid; 25.6 mg, 90% yield, 93% ee; $[\alpha]_D^{22}$ +18.7 (*c* 1.0, $CHCl_3$); ^1H NMR (600 MHz, $CDCl_3$) δ 8.59 (d, *J* = 4.4 Hz, 1H), 7.77 – 7.66 (m, 3H), 7.54 – 7.40 (m, 4H), 7.33 (d, *J* = 7.8 Hz, 1H), 7.29 – 7.21 (m, 2H), 3.03 (p, *J* = 8.8 Hz, 1H), 2.51 – 2.32 (m, 2H), 2.22 – 2.14 (m, 1H), 2.13 – 2.04 (m, 2H), 2.03 – 1.96 (m, 1H); ^{13}C NMR (151 MHz, $CDCl_3$) δ 152.9 (d, *J* = 16.7 Hz), 149.8, 145.5, 137.4, 132.1 (d, *J* = 98.9 Hz), 131.7 (d, *J* = 2.7 Hz), 130.6 (d, *J* = 8.9 Hz), 128.7 (d, *J* = 11.1 Hz), 124.6, 124.2, 123.5 (d, *J* = 94.3 Hz), 33.4 (d, *J* = 74.9 Hz), 21.4 (d, *J* = 5.3 Hz), 21.2 (d, *J* = 5.4 Hz), 20.1 (d, *J* = 15.2 Hz); ^{31}P NMR (243 MHz, $CDCl_3$) δ 30.1; HRMS (ESI) m/z 284.1199 (M + H^+), calc. for $C_{17}H_{19}NOP$ 284.1200.

The ee was determined by HPLC analysis: CHIRALPAK IE (4.6 mm i.d. x 250 mm); Hexane/2-propanol = 40/60; flow rate 1.0 mL/min; 25 °C; 254 nm; retention time: 16.3 min (minor) and 19.0 min (major).

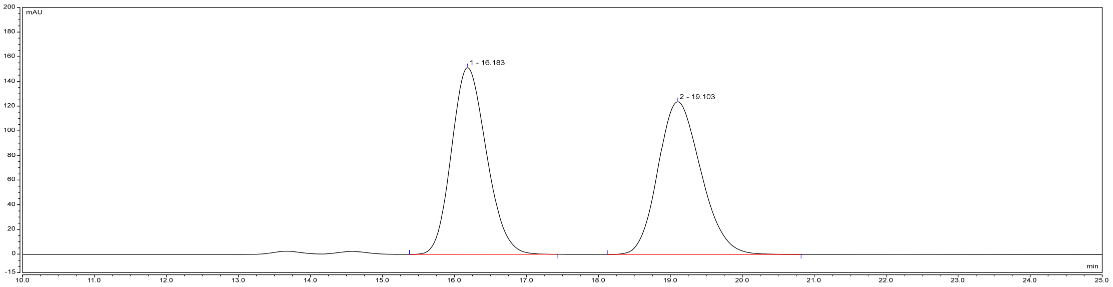

Entry	Retention Time	Area	Height	%Area
1	16.183	85.0905	151.28	49.88
2	19.103	85.5042	123.70	50.12

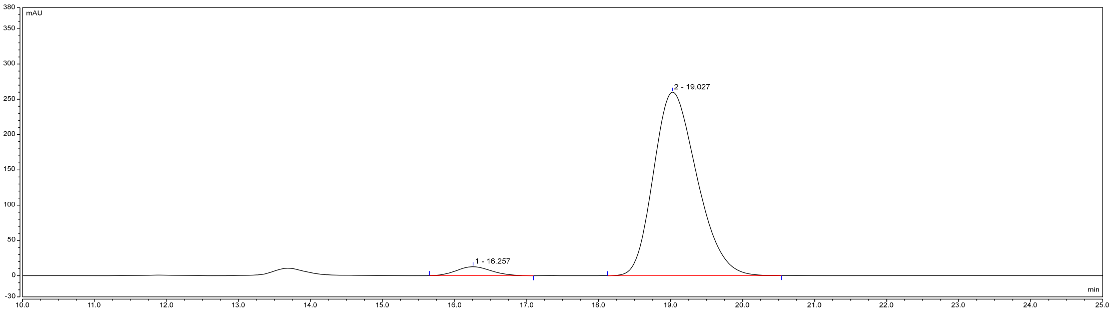

Entry	Retention Time	Area	Height	%Area
1	16.257	6.9786	12.58	3.70
2	19.027	181.4586	260.01	96.30

(*R*,*E*)-cyclopentyl(phenyl)(2-(pyridin-2-yl)vinyl)phosphine oxide (21):

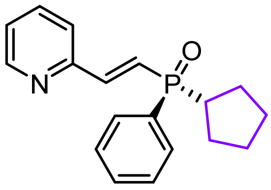

21

white solid; Mp 71.2-72.0 °C; 26.7 mg, 90% yield, 94% ee; $[\alpha]_D^{22}$+18.6 (*c* 1.0, $CHCl_3$); ^{1}H NMR (600 MHz, $CDCl_3$) δ 8.56 (d, *J* = 1.5 Hz, 1H), 7.81 – 7.71 (m, 2H), 7.66 – 7.60 (m, 1H), 7.55 – 7.38 (m, 4H), 7.33 – 7.24 (m, 2H), 7.20 – 7.15 (m, 1H), 2.45 – 2.34 (m, 1H), 1.88 – 1.81 (m, 2H), 1.78 – 1.60 (m, 4H), 1.58 – 1.48 (m, 2H); ^{13}C NMR (151 MHz, $CDCl_3$) δ 152.9 (d, *J* = 16.7 Hz), 149.9, 145.5 (d, *J* = 2.8 Hz), 137.1, 132.8 (d, *J* = 98.2 Hz), 131.5 (d, *J* = 1.5 Hz), 130.6 (d, *J* = 8.8 Hz), 128.6 (d, *J* = 11.2 Hz), 124.6, 124.1, 123.7 (d, *J* = 94.2 Hz), 38.5 (d, *J* = 77.2 Hz), 26.8 (d, *J* = 9.1 Hz), 26.6 (d, *J* = 9.0 Hz), 26.3 (d, *J* = 27.3 Hz); ^{31}P NMR (243 MHz, $CDCl_3$) δ 33.5; HRMS (ESI) m/z 298.1356 (M + H^+), calc. for $C_{18}H_{21}NOP$ 298.1356.

The ee was determined by HPLC analysis: CHIRALPAK AD-H (4.6 mm i.d. x 250 mm); Hexane/2-propanol = 90/10; flow rate 1.0 mL/min; 25 °C; 254 nm; retention time: 25.7 min (major) and 31.0 min (minor).

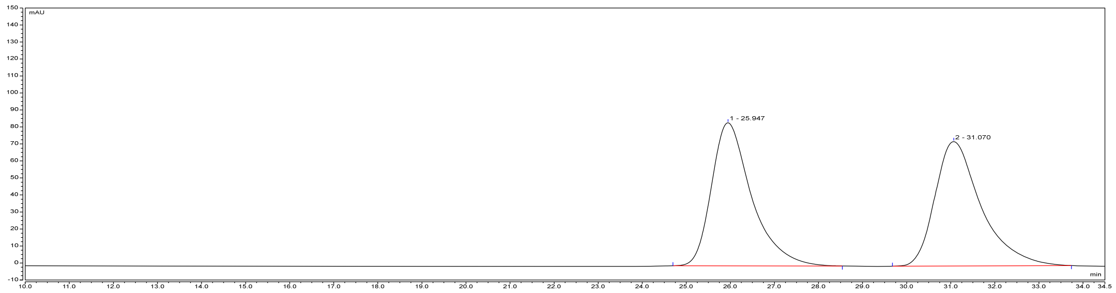

Entry	Retention Time	Area	Height	%Area
1	25.947	88.6597	84.13	50.03
2	31.070	88.5596	73.25	49.97

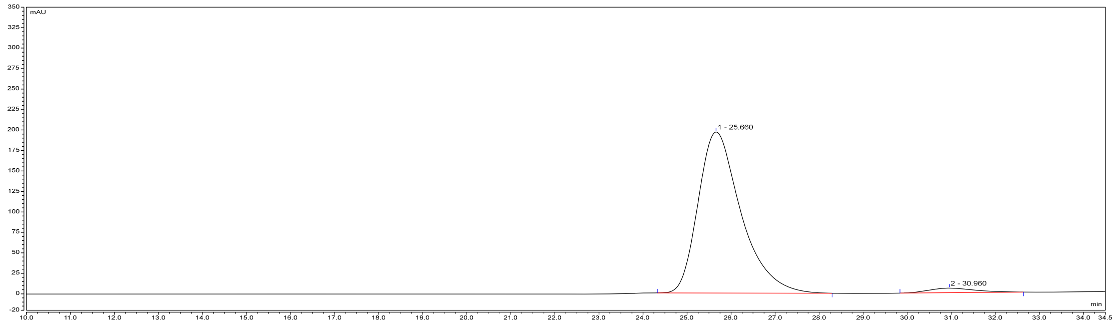

Entry	Retention Time	Area	Height	%Area
1	25.660	221.5964	196.54	97.06
2	30.960	6.7026	5.60	2.94

(*R*,*E*)-cyclohexyl(phenyl)(2-(pyridin-2-yl)vinyl)phosphine oxide (22):

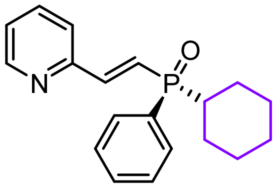

22

white solid; Mp 63.6-64.0 °C; 25.5 mg, 82% yield, 90% ee; $[\alpha]_D^{22}$+18.6 (*c* 1.0, $CHCl_3$); ^{1}H NMR (600 MHz, $CDCl_3$) δ 8.59 (s, 1H), 7.79 – 7.72 (m, 2H), 7.70 – 7.62 (m, 1H), 7.53 (td, *J* = 17.3, 2.1 Hz, 1H), 7.48 – 7.41 (m, 3H), 7.35 (ddd, *J* = 26.5, 16.8, 2.0 Hz, 1H), 7.30 – 7.26 (m, 1H), 7.21 – 7.17 (m, 1H), 1.95 – 1.83 (m, 2H), 1.80 – 1.68 (m, 3H), 1.63 (s, 1H), 1.47 – 1.31 (m, 2H), 1.24 – 1.12 (m, 3H); ^{13}C NMR (151 MHz, $CDCl_3$) δ 152.8 (d, *J* = 16.4 Hz), 149.9, 146.0 (d, *J* = 2.4 Hz), 137.1, 131.8 (d, *J* = 97.2 Hz), 131.5 (d, *J* = 1.9 Hz), 130.7 (d, *J* = 8.7 Hz), 128.6 (d, *J* = 11.2 Hz), 124.7, 124.1, 122.7 (d, *J* = 93.1 Hz), 39.0 (d, *J* = 74.8 Hz), 26.3 (d, *J* = 7.5 Hz), 26.2 (d, *J* = 7.3 Hz), 25.7, 25.0 (d, *J* = 2.1 Hz), 24.6 (d, *J* = 2.2 Hz); ^{31}P NMR (243 MHz, $CDCl_3$) δ 33.4; HRMS (ESI) m/z 312.1509 (M + H^+), calc. for $C_{19}H_{23}NOP$ 312.1509. The ee was determined by HPLC analysis: CHIRALPAK AD-H (4.6 mm i.d. x 250 mm); Hexane/2-propanol = 80/20; flow rate 1.0 mL/min; 25 °C; 254 nm; retention time: 9.7 min (major) and 11.1min (minor).

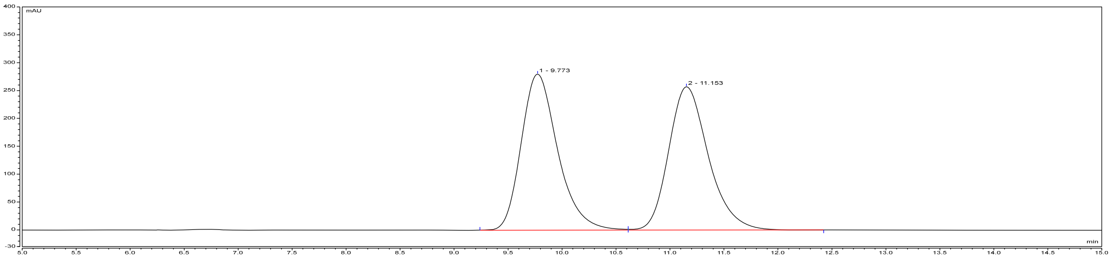

Entry	Retention Time	Area	Height	%Area
1	9.773	109.6384	279.78	50.05
2	11.153	109.4149	256.85	49.95

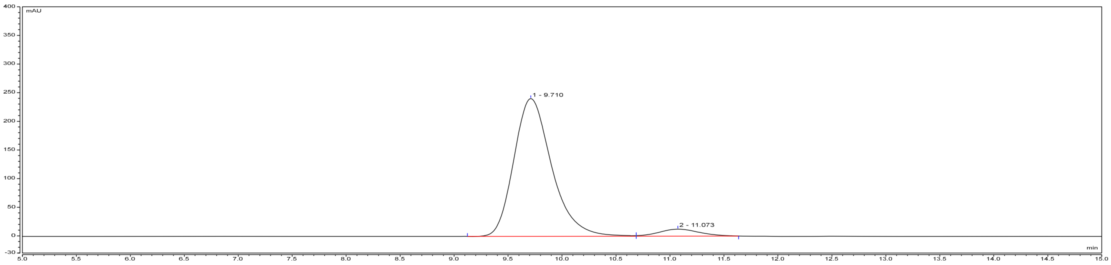

Entry	Retention Time	Area	Height	%Area
1	9.710	97.6030	240.40	94.91
2	11.073	5.2292	12.23	5.09

(*R*,*E*)-(2-(3-fluoropyridin-2-yl)vinyl)(isopropyl)(phenyl)phosphine oxide (23):

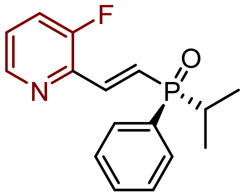

23

white solid; Mp 79.0-80.0 °C; 23.3 mg, 81% yield, 94% ee; $[\alpha]_D^{22}$ -17.6 (*c* 1.0, $CHCl_3$); ^1H NMR (400 MHz, $CDCl_3$) δ 8.26 (d, *J* = 4.5 Hz, 1H), 7.71 (td, *J* = 17.4, 1.3 Hz, 1H), 7.65 – 7.59 (m, 2H), 7.38 – 7.28 (m, 4H), 7.27 – 7.21 (m, 1H), 7.14 – 7.09 (m, 1H), 2.09 – 1.97 (m, 1H), 1.08 (dd, *J* = 16.7, 7.2 Hz, 3H), 0.97 (dd, *J* = 16.7, 7.2 Hz, 3H); ^{13}C NMR (101 MHz, $CDCl_3$) δ 157.7 (d, *J* = 264.3 Hz), 145.6 (d, *J* = 5.1 Hz), 141.6 (dd, *J* = 16.8, 11.3 Hz), 138.3 (d, *J* = 3.7 Hz),131.8 (d, *J* = 97.3 Hz), 131.7 (d, *J* = 2.7 Hz), 130.9 (d, *J* = 8.7 Hz), 128.7 (d, *J* = 11.2 Hz), 125.7 (d, *J* = 4.1 Hz), 125.2 (d, *J* = 3.4 Hz), 124.1 (d, *J* = 18.8 Hz), 29.1 (d, *J* = 74.6 Hz), 15.5 (d, *J* = 2.4 Hz), 15.2 (d, *J* = 2.6 Hz); ^{31}P NMR (162 MHz, $CDCl_3$) δ 35.8; ^{19}F NMR (376 MHz, $CDCl_3$) δ -124.2; HRMS (ESI) m/z 290.1105 (M + H^+), calc. for $C_{16}H_{18}FNOP$ 290.1106.

The ee was determined by HPLC analysis: CHIRALPAK IE (4.6 mm i.d. x 250 mm); Hexane/2-propanol = 40/60; flow rate 1.0 mL/min; 25 °C; 254 nm; retention time: 10.8 min (major) and 12.4 min (minor).

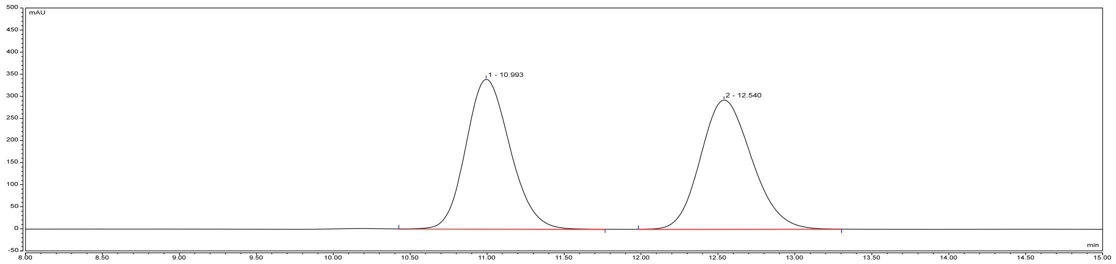

Entry	Retention Time	Area	Height	%Area
1	10.993	112.7650	338.91	49.96
2	12.540	112.9494	292.34	50.04

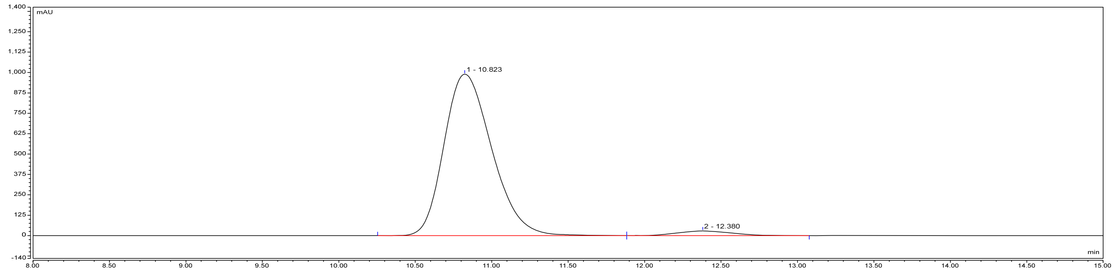

Entry	Retention Time	Area	Height	%Area
1	10.823	358.4248	990.19	96.82
2	12.380	11.7550	28.84	3.18

methyl (*R*,*E*)-2-(2-(isopropyl(phenyl)phosphoryl)vinyl)isonicotinate (24):

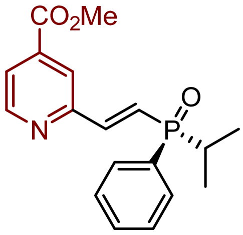

24

colorless oily liquid; 31.3 mg, 95% yield, 90% ee; $[\alpha]_D^{22}$ +16.7 (*c* 1.0, $CHCl_3$); ^1H NMR (400 MHz, $CDCl_3$) δ 8.75 (d, *J* = 4.9 Hz, 1H), 7.87 (s, 1H), 7.81 – 7.73 (m, 3H), 7.61 (t, *J* = 17.2 Hz, 1H), 7.53 – 7.39 (m, 4H), 3.94 (s, 3H), 2.30 – 2.11 (m, 1H), 1.23 (dd, *J* = 16.7, 7.1 Hz, 3H), 1.12 (dd, *J* = 16.7, 7.1 Hz, 3H); ^{13}C NMR (101 MHz, $CDCl_3$) δ 165.2, 154.0 (d, *J* = 16.7 Hz), 150.6, 145.2 (d, *J* = 2.1 Hz), 138.8, 131.8 (d, *J* = 2.6 Hz), 131.6 (d, *J* = 97.5 Hz), 130.9 (d, *J* = 8.7 Hz), 128.8 (d, *J* = 11.2 Hz), 124.6 (d, *J* = 91.7 Hz), 123.5, 123.3, 53.0, 29.1 (d, *J* = 74.5 Hz), 15.4 (d, *J* = 2.5 Hz), 15.2 (d, *J* = 2.3 Hz); ^{31}P NMR (162 MHz, $CDCl_3$) δ 35.8; HRMS (ESI) m/z 330.1254 (M + H^+), calc. for $C_{18}H_{21}NO_3P$ 330.1255.

The ee was determined by HPLC analysis: CHIRALPAK IC (4.6 mm i.d. x 250 mm); Hexane/2-propanol = 50/50; flow rate 2.0 mL/min; 25 °C; 254 nm; retention time: 24.4 min (major) and 36.2 min (minor).

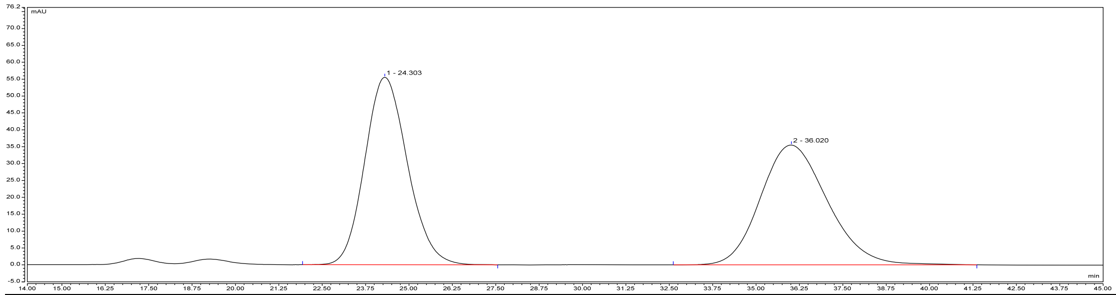

Entry	Retention Time	Area	Height	%Area
1	24.303	79.3146	55.50	50.03
2	36.020	79.2309	35.49	49.97

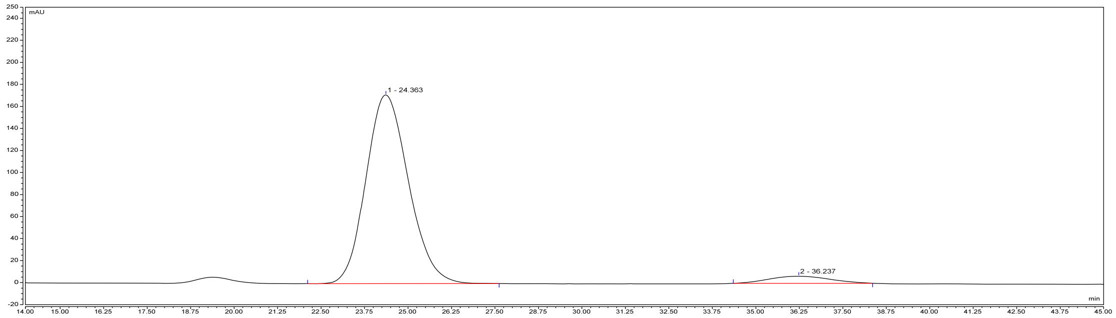

Entry	Retention Time	Area	Height	%Area
1	24.363	243.4377	171.42	94.94
2	36.237	12.9697	6.56	5.06

(*R*,*E*)-isopropyl(phenyl)(2-(4-(trifluoromethyl)pyridin-2-yl)vinyl)phosphine oxide (25):

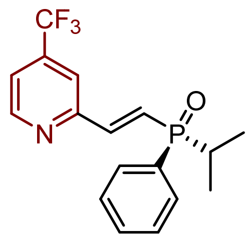

25

colorless oily liquid; 29.6 mg, 87% yield, 89% ee; $[\alpha]_D^{22}$ -27.0 (*c* 1.0, $CHCl_3$); ^1H NMR (400 MHz, $CDCl_3$) δ 8.78 (d, *J* = 4.9 Hz, 1H), 7.81 – 7.72 (m, 2H), 7.60 (t, *J* = 14.7 Hz, 1H), 7.52 – 7.40 (m, 6H), 2.19 (dq, *J* = 14.9, 7.3 Hz, 1H), 1.26 – 1.19 (m, 3H), 1.15 – 1.07 (m, 3H); ^{13}C NMR (101 MHz, $CDCl_3$) δ 154.3 (d, *J* = 16.7 Hz), 150.9, 144.8 (d, *J* = 2.9 Hz), 139.5 (q, *J* = 34.2 Hz), 131.8 (d, *J* = 2.5 Hz), 131.5 (d, *J* = 97.4 Hz), 130.8 (d, *J* = 8.7 Hz), 128 (d, *J* = 11.4 Hz), 126.0, 125.0, 122.6 (d, *J* = 273.3 Hz), 119.7 (dq, *J* = 21.7, 3.3 Hz), 29.0 (d, *J* = 74.7 Hz), 15.4 (d, *J* = 2.6 Hz), 15.1 (d, *J* = 2.6 Hz); ^{31}P NMR (162 MHz, $CDCl_3$) δ 35.6; ^{19}F NMR (376 MHz, $CDCl_3$) δ -65.0; HRMS (ESI) m/z 340.1073 ($M + H^+$), calc. for $C_{17}H_{18}F_3NOP$ 340.1073.

The ee was determined by HPLC analysis: CHIRALPAK IC (4.6 mm i.d. x 250 mm); Hexane/2-propanol = 40/60; flow rate 1.0 mL/min; 25 °C; 254 nm; retention time: 9.1 min (major) and 10.1min (minor).

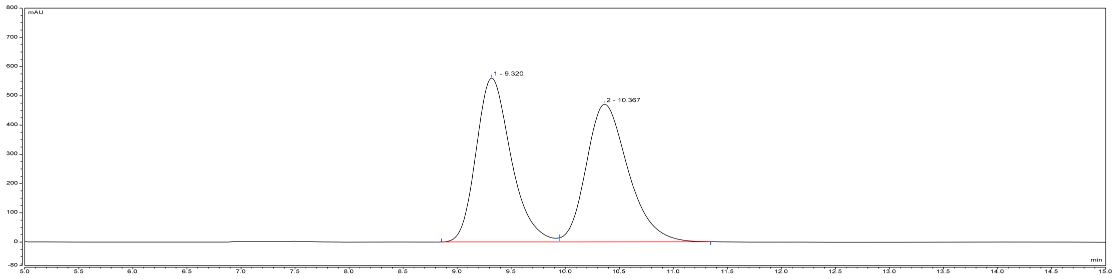

Entry	Retention Time	Area	Height	%Area
1	9.320	210.1016	560.21	49.86
2	10.367	211.2535	470.45	50.14

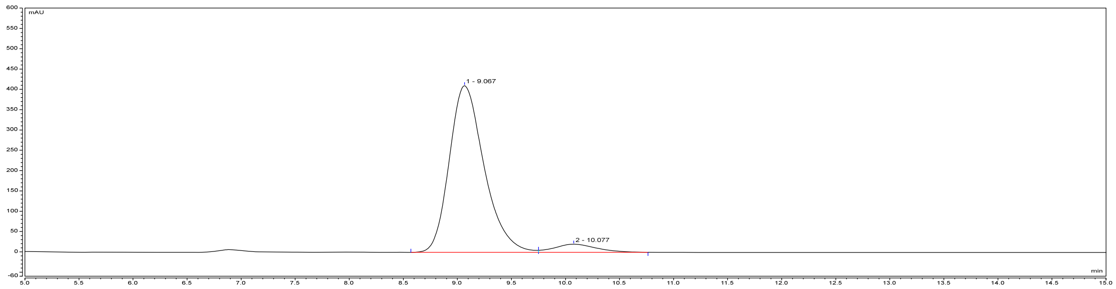

Entry	Retention Time	Area	Height	%Area
1	9.067	152.8741	410.04	94.52
2	10.077	8.8667	19.90	5.48

(*R*,*E*)-(2-(4-fluoropyridin-2-yl)vinyl)(isopropyl)(phenyl)phosphine oxide (26):

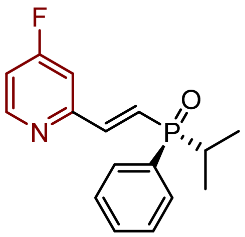

26

colorless oily liquid; 27.4 mg, 95% yield, 92% ee; $[\alpha]_D^{22}$ -8.6 (*c* 1.0, $CHCl_3$); ^1H NMR (600 MHz, $CDCl_3$) δ 8.56 (dd, *J* = 8.5, 5.5 Hz, 1H), 7.78 – 7.72 (m, 2H), 7.55 – 7.37 (m, 5H), 7.05 (dd, *J* = 9.3, 2.4 Hz, 1H), 6.98 – 6.95 (m, 1H), 2.21 – 2.14 (m, 1H), 1.21 (dd, *J* = 16.7, 7.2 Hz, 3H), 1.11 (dd, *J* = 16.7, 7.2 Hz, 3H); ^{13}C NMR (151 MHz, $CDCl_3$) δ 169.3 (d, *J* = 263.7 Hz), 156.0 (dd, *J* = 16.6, 6.8 Hz), 152.2 (d, *J* = 7.4 Hz), 145.0, 131.8 (d, *J* = 2.8 Hz), 131.6 (d, *J* = 97.6 Hz), 130.8 (d, *J* = 8.8 Hz), 128.8 (d, *J* = 11.2 Hz), 124.8 (d, *J* = 91.5 Hz), 112.4 (d, *J* = 17.2 Hz), 111.7 (d, *J* = 16.2 Hz), 29.0 (d, *J* = 74.6 Hz), 15.4 (d, *J* = 2.3 Hz), 15.1 (d, *J* = 2.7 Hz); ^{31}P NMR (243 MHz, $CDCl_3$) δ 35.8; ^{19}F NMR (565 MHz, $CDCl_3$) δ -101.6; HRMS (ESI) m/z 290.1105 ($M + H^+$), calc. for $C_{16}H_{17}FNOP$ 290.1105.

The ee was determined by HPLC analysis: CHIRALPAK IC (4.6 mm i.d. x 250 mm); Hexane/2-propanol = 40/60; flow rate 1.0 mL/min; 25 °C; 254 nm; retention time: 11.3 min (minor) and 16.4 min (major).

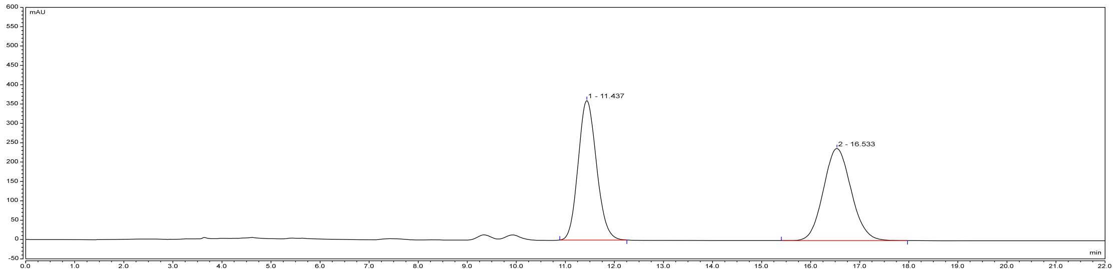

Entry	Retention Time	Area	Height	%Area
1	11.437	156.5268	360.40	50.06
2	16.533	156.1633	237.81	49.94

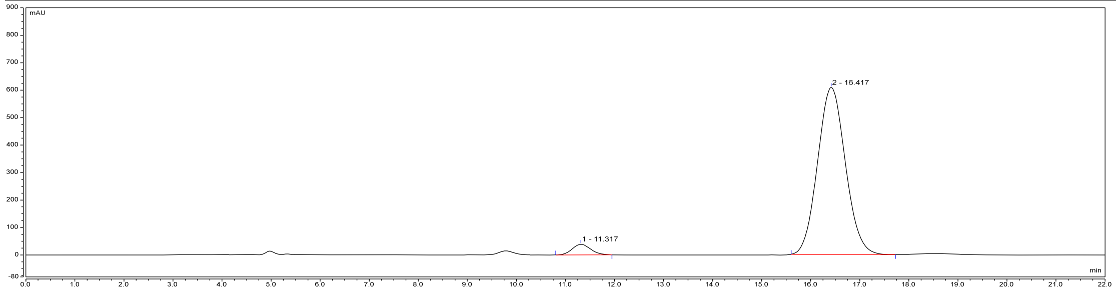

Entry	Retention Time	Area	Height	%Area
1	11.317	16.6835	38.45	4.01
2	16.417	399.3247	608.27	95.99

(*R*,*E*)-(2-(4-chloropyridin-2-yl)vinyl)(isopropyl)(phenyl)phosphine oxide (27):

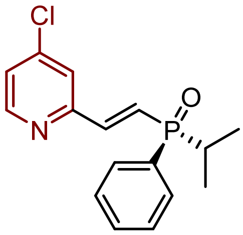

27

colorless oily liquid; 26.5 mg, 87% yield, 90% ee; $[\alpha]_D^{22}$ +16.7 (*c* 1.0, $CHCl_3$); ^1H NMR (400 MHz, $CDCl_3$) δ 8.65 – 8.38 (m, 1H), 7.93 – 7.64 (m, 2H), 7.58 – 7.35 (m, 5H), 7.32 – 7.29 (m, 1H), 7.25 – 7.19 (m, 1H), 2.52 – 2.03 (m, 1H), 1.20 (ddd, *J* = 16.7, 7.1, 2.6 Hz, 3H), 1.10 (ddd, *J* = 16.7, 7.1, 2.6 Hz, 3H); ^{13}C NMR (101 MHz, $CDCl_3$) δ 154.3 (d, *J* = 16.6 Hz), 150.5, 145.3, 144.7 (d, *J* = 2.6 Hz), 131.8 (d, *J* = 2.7 Hz), 131.6 (d, *J* = 97.4 Hz), 130.8 (d, *J* = 8.7 Hz), 128.7 (d, *J* = 11.3 Hz), 125.2 (d, *J* = 91.2 Hz), 124.8, 124.2, 29.0 (d, *J* = 74.6 Hz), 15.4 (d, *J* = 2.5 Hz), 15.1 (d, *J* = 2.6 Hz); ^{31}P NMR (162 MHz, $CDCl_3$) δ 35.6; HRMS (ESI) m/z 306.0810 (M + H^+), calc. for $C_{16}H_{18}ClNOP$ 306.0810.

The ee was determined by HPLC analysis: CHIRALPAK IC (4.6 mm i.d. x 250 mm); Hexane/2-propanol = 40/60; flow rate 1.0 mL/min; 25 °C; 254 nm; retention time: 13.5 min (minor) and 18.1 min (major).

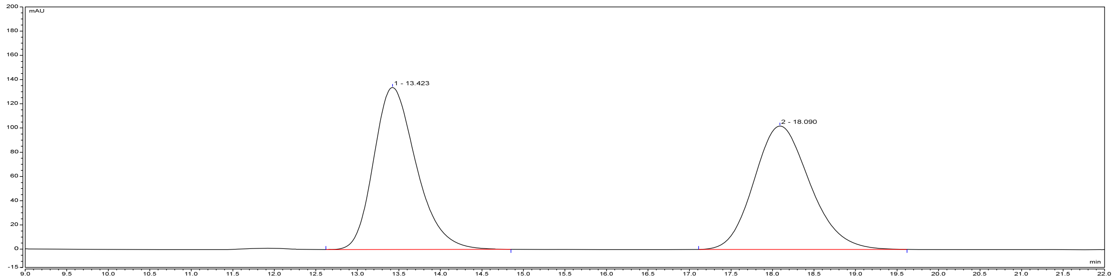

Entry	Retention Time	Area	Height	%Area
1	13.423	79.4860	133.68	49.95
2	18.090	79.6310	101.88	50.05

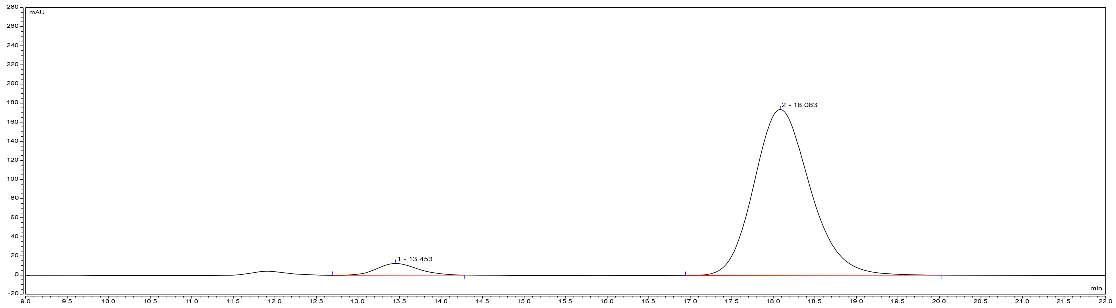

Entry	Retention Time	Area	Height	%Area
1	13.453	6.9338	12.28	4.86
2	18.083	135.6569	173.59	95.14

(*R*,*E*)-isopropyl(2-(4-methylpyridin-2-yl)vinyl)(phenyl)phosphine oxide (28):

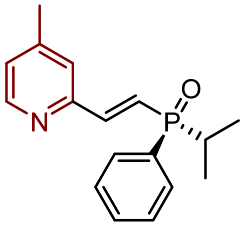

28

colorless oily liquid; 24.6 mg, 86% yield, 92% ee; $[\alpha]_D^{22}$ +14.4 (*c* 1.0, $CHCl_3$); ^1H NMR (600 MHz, $CDCl_3$) δ 8.44 (d, *J* = 5.0 Hz, 1H), 7.79 – 7.73 (m, 2H), 7.53 – 7.47 (m, 1H), 7.46 – 7.33 (m, 4H), 7.12 (s, 1H), 7.04 (d, *J* = 4.9 Hz, 1H), 2.31 (s, 3H), 2.19 – 2.12 (m, 1H), 1.19 (dd, *J* = 16.7, 7.2 Hz, 3H), 1.09 (dd, *J* = 16.7, 7.2 Hz, 3H); ^{13}C NMR (151 MHz, $CDCl_3$) δ 152.5 (d, *J* = 16.5 Hz), 149.4, 148.7, 146.1, 131.9 (d, *J* = 97.0 Hz), 131.6 (d, *J* = 2.8 Hz), 130.8 (d, *J* = 8.7 Hz), 128.6 (d, *J* = 11.1 Hz), 125.9, 125.1, 122.8 (d, *J* = 93.0 Hz), 29.1 (d, *J* = 74.6 Hz), 21.0, 15.4 (d, *J* = 2.1 Hz), 15.1 (d, *J* = 2.1 Hz); ^{31}P NMR (243 MHz, $CDCl_3$) δ 36.1; HRMS (ESI) m/z 286.1356 (M + H^+), calc. for $C_{17}H_{21}NOP$ 286.1356.

The ee was determined by HPLC analysis: CHIRALPAK IG (4.6 mm i.d. x 250 mm); Hexane/2-propanol = 40/60; flow rate 1.0 mL/min; 25 °C; 254 nm; retention time: 8.9 min (major) and 10.3 min (minor).

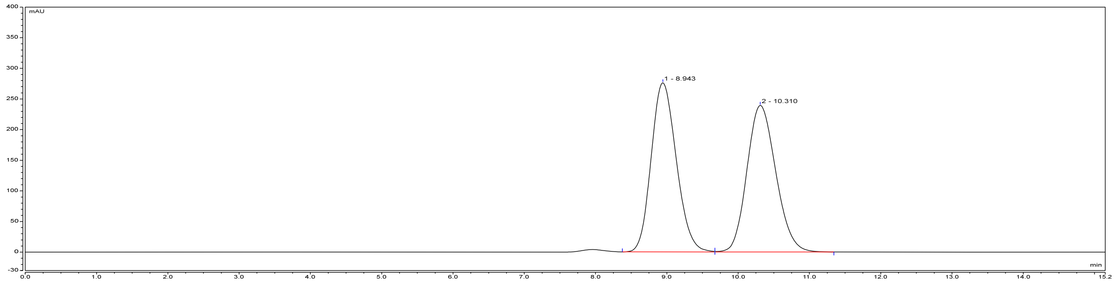

Entry	Retention Time	Area	Height	%Area
1	8.943	112.6527	276.03	50.13
2	10.310	112.0810	239.12	49.87

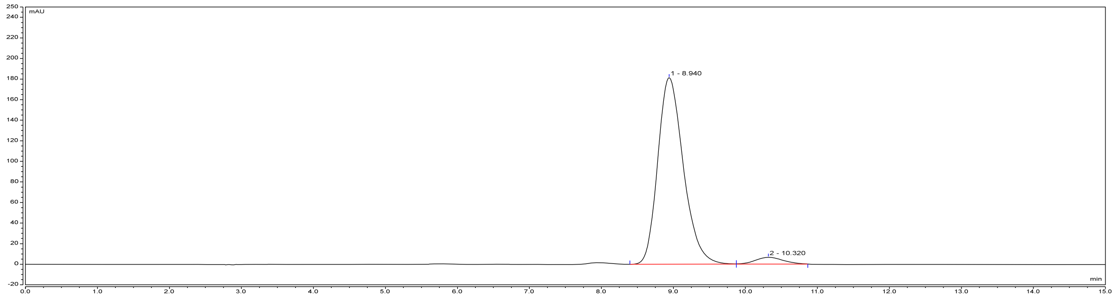

Entry	Retention Time	Area	Height	%Area
1	8.940	74.6627	181.25	96.18
2	10.320	2.9639	6.60	3.82

(*R*,*E*)-isopropyl(phenyl)(2-(4-phenylpyridin-2-yl)vinyl)phosphine oxide (29):

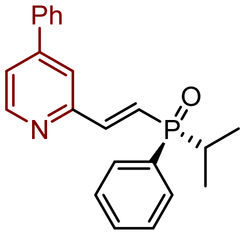

29

white solid; Mp 92.5-93.2 °C; 32.4 mg, 93% yield, 92% ee; $[\alpha]_D^{22}$ +64.4 (*c* 1.0, $CHCl_3$); ^{1}H NMR (600 MHz, $CDCl_3$) δ 8.65 (d, *J* = 5.0 Hz, 1H), 7.83 – 7.76 (m, 2H), 7.65 (t, *J* = 17.2 Hz, 1H), 7.59 (d, *J* = 7.7 Hz, 2H), 7.54 (s, 1H), 7.51 – 7.40 (m, 8H), 2.20 (dq, *J* = 14.5, 7.5 Hz, 1H), 1.24 (dd, *J* = 16.7, 7.1 Hz, 3H), 1.13 (dd, *J* = 16.7, 7.1 Hz, 3H); ^{13}C NMR (151 MHz, $CDCl_3$) δ 153.3 (d, *J* = 16.4 Hz), 150.2, 149.9, 146.2, 137.6, 131.8 (d, *J* = 97.3 Hz), 131.7 (d, *J* = 2.1 Hz), 130.8 (d, *J* = 8.6 Hz), 129.5, 129.3, 128.7 (d, *J* = 11.1 Hz), 127.0, 123.6, 122.9, 122.0, 29.19 (d, *J* = 74.5 Hz), 15.4 (d, *J* = 2.0 Hz), 15.2 (d, *J* = 2.6 Hz); ^{31}P NMR (243 MHz, $CDCl_3$) δ 36.2; HRMS (ESI) m/z 348.1512 (M + H^+), calc. for $C_{22}H_{23}NOP$ 348.1514.

The ee was determined by HPLC analysis: CHIRALPAK IC (4.6 mm i.d. x 250 mm); Hexane/2-propanol = 50/50; flow rate 2.0 mL/min; 25 °C; 254 nm; retention time: 12.3 min (major) and 20.8 min (minor).

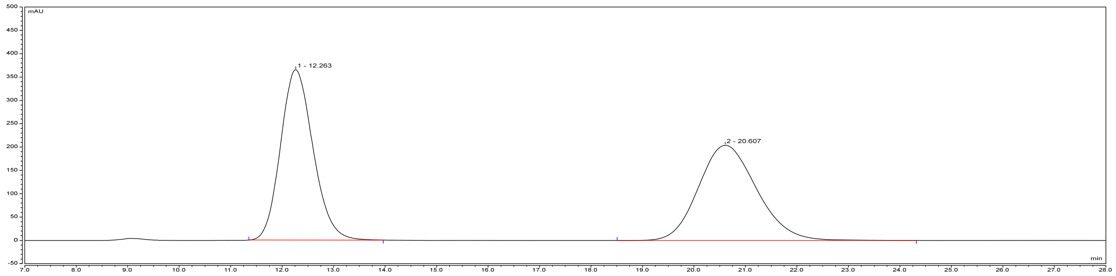

Entry	Retention Time	Area	Height	%Area
1	12.263	267.9361	365.00	50.05
2	20.607	267.4207	203.96	49.95

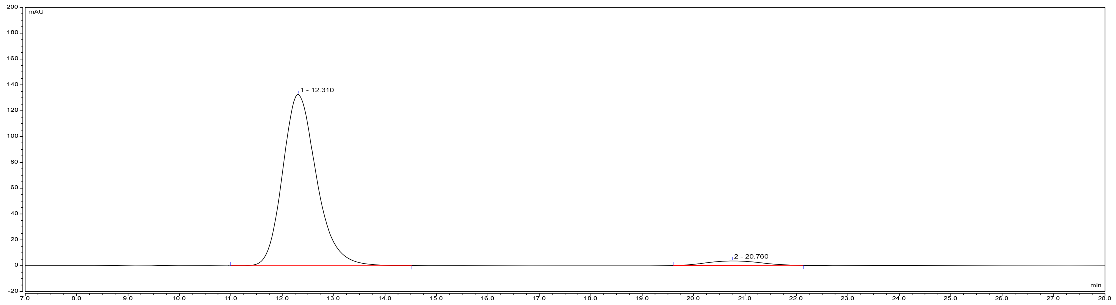

Entry	Retention Time	Area	Height	%Area
1	12.310	101.0981	132.55	95.96
2	20.760	4.2558	3.56	4.04

(*R*,*E*)-isopropyl(2-(4-methoxypyridin-2-yl)vinyl)(phenyl)phosphine oxide (30):

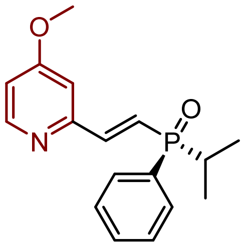

30

white solid; Mp 90.0-91.2 °C; 25.7 mg, 85% yield, 93% ee; $[\alpha]_D^{22}$ +46.2 (*c* 1.0, $CHCl_3$); ^1H NMR (600 MHz, $CDCl_3$) δ 8.41 (d, *J* = 5.6 Hz, 1H), 7.82 – 7.68 (m, 2H), 7.53 – 7.41 (m, 4H), 7.34 (dd, *J* = 26.5, 16.8 Hz, 1H), 6.83 (d, *J* = 2.4 Hz, 1H), 6.73 (dd, *J* = 5.6, 2.4 Hz, 1H), 3.81 (s, 3H), 2.21 – 2.11 (m, 1H), 1.20 (dd, *J* = 16.7, 7.2 Hz, 3H), 1.09 (dd, *J* = 16.7, 7.2 Hz, 3H); ^{13}C NMR (151 MHz, $CDCl_3$) δ 166.6, 154.5 (d, *J* = 16.5 Hz), 151.1, 146.4 (d, *J* = 2.9 Hz), 131.9 (d, *J* = 97.0 Hz),131.6 (d, *J* = 2.2 Hz), 130.8 (d, *J* = 8.7 Hz), 128.7 (d, *J* = 11.1 Hz), 122.9 (d, *J* = 92.8 Hz), 111.4, 109.8, 55.4, 29.0 (d, *J* = 74.7 Hz), 15.4 (d, *J* = 2.0 Hz), 15.1 (d, *J* = 2.3 Hz); ^{31}P NMR (243 MHz, $CDCl_3$) δ 36.0; HRMS (ESI) m/z 302.1305 (M + H^+), calc. for $C_{17}H_{21}NO_2P$ 302.1304.

The ee was determined by HPLC analysis: CHIRALPAK IE (4.6 mm i.d. x 250 mm); Hexane/2-propanol = 40/60; flow rate 1.0 mL/min; 25 °C; 254 nm; retention time: 15.3 min (minor) and 17.8 min (major).

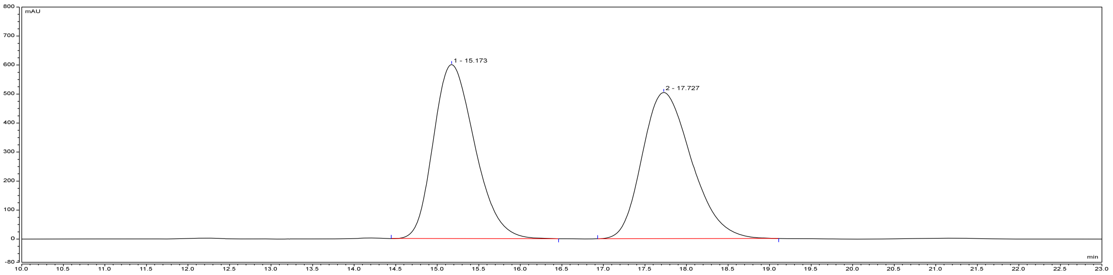

Entry	Retention Time	Area	Height	%Area
1	15.173	346.1259	599.48	49.95
2	17.727	346.8020	503.83	50.05

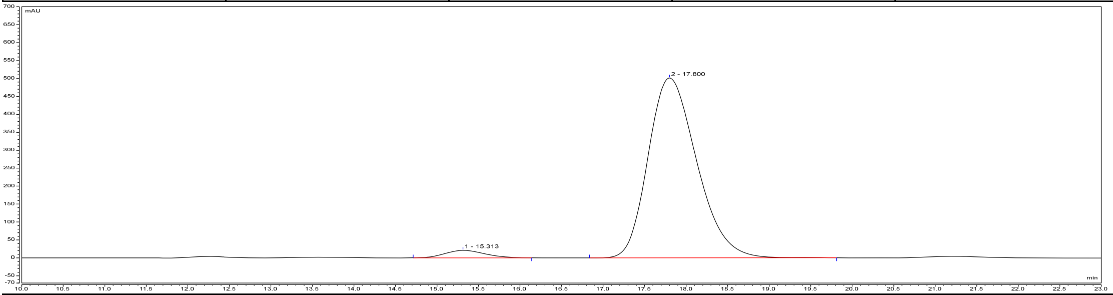

Entry	Retention Time	Area	Height	%Area
1	15.313	11.5282	20.80	3.28
2	17.800	340.2972	501.26	96.72

methyl (*R*,*E*)-6-(2-(isopropyl(phenyl)phosphoryl)vinyl)nicotinate (31):

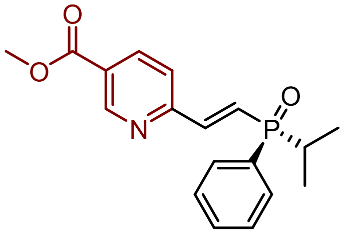

31

white solid; Mp 125.0-125.5 °C; 31.2 mg, 95% yield, 92% ee; $[\alpha]_D^{22}$ -79.0 (*c* 1.0, $CHCl_3$); ^1H NMR (400 MHz, $CDCl_3$) δ 9.18 (d, *J* = 1.9 Hz, 1H), 8.27 (dd, *J* = 8.0, 2.0 Hz, 1H), 7.77 (ddd, *J* = 9.5, 7.6, 1.5 Hz, 2H), 7.67 – 7.42 (m, 5H), 7.37 (d, *J* = 8.0 Hz, 1H), 3.92 (s, 3H), 2.27 – 2.05 (m, 1H), 1.22 (dd, *J* = 16.7, 7.2 Hz, 3H), 1.11 (dd, *J* = 16.7, 7.2 Hz, 3H); ^{13}C NMR (101 MHz, $CDCl_3$) δ 165.4, 156.2 (d, *J* = 16.5 Hz), 151.1, 145.1 (d, *J* = 2.8 Hz), 138.4, 131.8 (d, *J* = 2.7 Hz), 131.6 (d, *J* = 97.3 Hz), 130.8 (d, *J* = 8.7 Hz), 128.8 (d, *J* = 11.5 Hz), 126.2 (d, *J* = 51.5 Hz), 125.5, 124.0, 52.6, 29.1 (d, *J* = 74.4 Hz), 15.4 (d, *J* = 2.6 Hz), 5.1 (d, *J* = 2.4 Hz); ^{31}P NMR (162 MHz, $CDCl_3$) δ 35.7; HRMS (ESI) m/z 330.1254 (M + H^+), calc. for $C_{18}H_{21}NO_3P$ 330.1254.

The ee was determined by HPLC analysis: CHIRALPAK IC (4.6 mm i.d. x 250 mm); Hexane/2-propanol = 40/60; flow rate 1.0 mL/min; 25 °C; 254 nm; retention time: 22.9 min (minor) and 28.0 min (major).

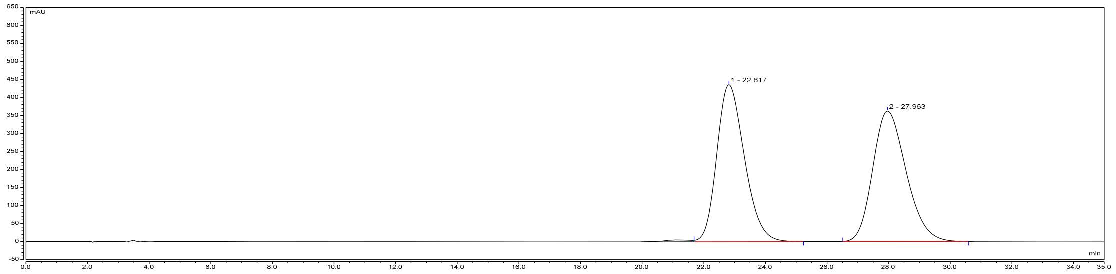

Entry	Retention Time	Area	Height	%Area
1	22.817	453.2406	435.61	49.93
2	27.963	454.5371	361.97	50.07

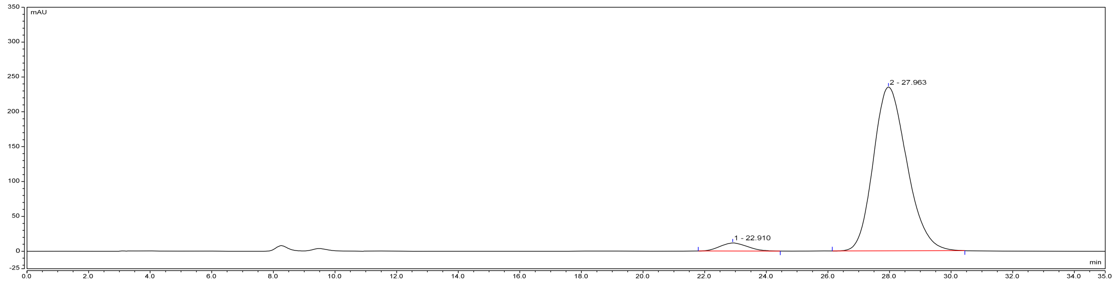

Entry	Retention Time	Area	Height	%Area
1	22.910	11.9811	11.59	3.88
2	27.963	296.4238	234.80	96.12

(*R*,*E*)-(2-(5-chloropyridin-2-yl)vinyl)(isopropyl)(phenyl)phosphine oxide (32):

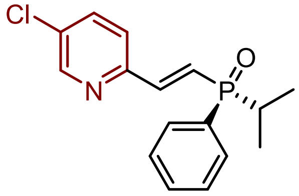

32

white solid; Mp 69.9-70.5 °C; 28.3 mg, 93% yield, 92% ee; $[\alpha]_D^{22}$ -61.6 (*c* 1.0, $CHCl_3$); ^1H NMR (400 MHz, $CDCl_3$) δ 8.53 (s, 1H), 7.83 – 7.69 (m, 2H), 7.63 (dd, *J* = 8.3, 2.4 Hz, 1H), 7.55 – 7.41 (m, 4H), 7.34 (dd, *J* = 26.0, 16.8 Hz, 1H), 7.24 (d, *J* = 8.3 Hz, 1H), 2.27 – 2.07 (m, 1H), 1.20 (dd, *J* = 16.7, 7.1 Hz, 3H), 1.10 (dd, *J* = 16.7, 7.1 Hz, 3H); ^{13}C NMR (101 MHz, $CDCl_3$) δ 151.1 (d, *J* = 16.7 Hz), 149.0, 144.9 (d, *J* = 2.9 Hz), 136.6, 132.4, 131.8 (d, *J* = 97.3 Hz), 131.7 (d, *J* = 2.7 Hz), 130.8 (d, *J* = 8.7 Hz), 128.7 (d, *J* = 11.3 Hz), 125.1, 123.5 (d, *J* = 92.4 Hz), 29.1 (d, *J* = 74.6 Hz), 15.4 (d, *J* = 2.5 Hz), 15.1 (d, *J* = 2.3 Hz); ^{31}P NMR (162 MHz, $CDCl_3$) δ 35.8; HRMS (ESI) m/z 306.0810 (M + H^+), calc. for $C_{16}H_{18}ClNOP$ 306.0810.

The ee was determined by HPLC analysis: CHIRALPAK IE (4.6 mm i.d. x 250 mm); Hexane/2-propanol = 40/60; flow rate 1.0 mL/min; 25 °C; 254 nm; retention time: 13.6 min (minor) and 16.1 min (major).

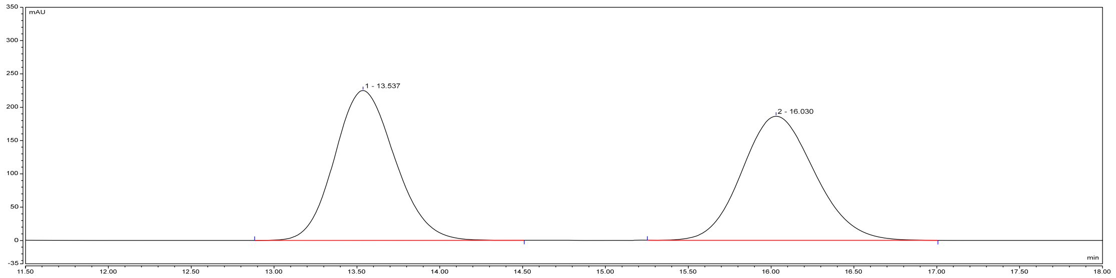

Entry	Retention Time	Area	Height	%Area
1	13.537	94.9207	224.65	49.99
2	16.030	94.9595	186.10	50.01

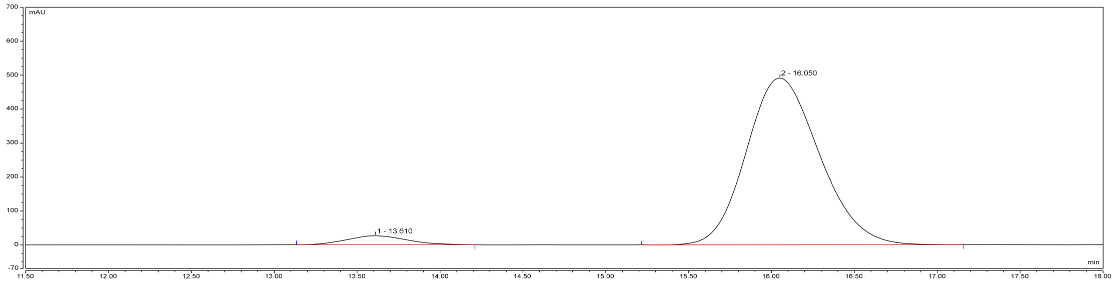

Entry	Retention Time	Area	Height	%Area
1	13.610	11.0228	26.39	4.20
2	16.050	251.2679	491.33	95.80

(*R*,*E*)-(2-(5-bromopyridin-2-yl)vinyl)(isopropyl)(phenyl)phosphine oxide (33):

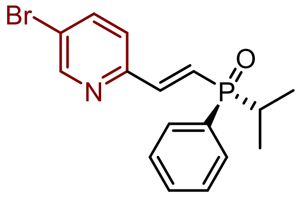

33

white solid; Mp 80.0-80.5 °C; 33.4 mg, 96% yield, 93% ee; $[\alpha]_D^{22}$ -34.6 (*c* 1.0, $CHCl_3$); ^1H NMR (600 MHz, $CDCl_3$) δ 8.62 (s, 1H), 7.81 – 7.70 (m, 3H), 7.53 – 7.42 (m, 4H), 7.35 (ddd, *J* = 26.0, 16.8, 1.0 Hz, 1H), 7.18 (d, *J* = 8.2 Hz, 1H), 2.20 – 2.12 (m, 1H), 1.20 (ddd, *J* = 16.7, 7.1, 1.3 Hz, 3H), 1.09 (ddd, *J* = 16.7, 7.1, 1.4 Hz, 3H); ^{13}C NMR (151 MHz, $CDCl_3$) δ 151.4 (d, *J* = 16.6 Hz), 151.2, 145.0 (d, *J* = 3.0 Hz), 139.6, 131.7 (d, *J* = 2.2 Hz), 131.6 (d, *J* = 97.6 Hz), 130.8 (d, *J* = 8.6 Hz), 128.7 (d, *J* = 11.1 Hz), 125.5, 123.6 (d, *J* = 92.3 Hz), 121.2, 29.0 (d, *J* = 74.7 Hz), 15.4 (d, *J* = 2.6 Hz), 15.1 (d, *J* = 2.1 Hz); ^{31}P NMR (243 MHz, $CDCl_3$) δ 35.9; HRMS (ESI) m/z 350.0304 (M + H^+), calc. for $C_{16}H_{18}BrNOP$ 350.0302.

The ee was determined by HPLC analysis: CHIRALPAK AD-H (4.6 mm i.d. x 250 mm); Hexane/2-propanol = 90/10; flow rate 1.0 mL/min; 25 °C; 254 nm; retention time: 26.4 min (minor) and 30.8 min (major).

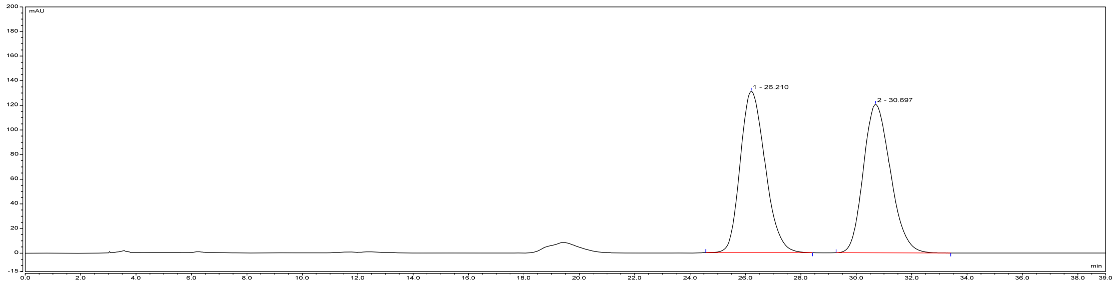

Entry	Retention Time	Area	Height	%Area
1	26.210	137.1183	130.98	50.01
2	30.697	137.0398	120.45	49.99

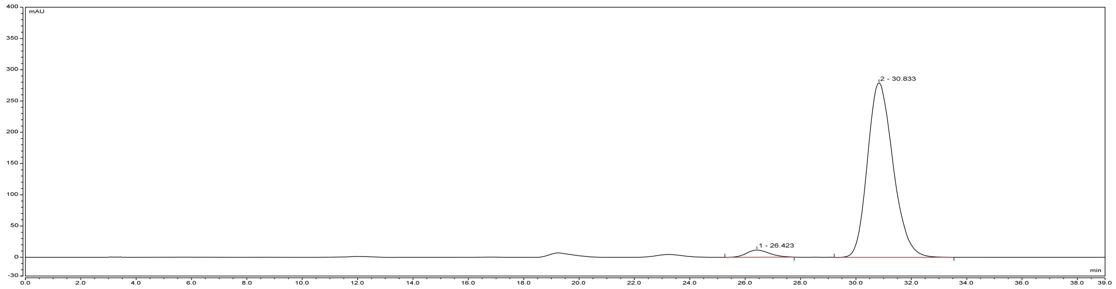

Entry	Retention Time	Area	Height	%Area
1	26.423	10.8239	11.47	3.50
2	30.833	298.7734	278.94	96.50

(*R*,*E*)-isopropyl(2-(5-methoxypyridin-2-yl)vinyl)(phenyl)phosphine oxide (34):

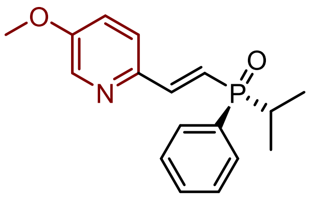

34

colorless oily liquid; 25.2 mg, 84% yield, 96% ee; $[\alpha]_D^{22}$ -59.2 (*c* 1.0, $CHCl_3$); ^1H NMR (600 MHz, $CDCl_3$) δ 8.28 (d, *J* = 2.7 Hz, 1H), 7.76 – 7.72 (m, 2H), 7.51 – 7.40 (m, 4H), 7.24 (d, *J* = 8.5 Hz, 1H), 7.19 – 7.10 (m, 2H), 3.81 (s, 3H), 2.13 (dq, *J* = 14.8, 7.4 Hz, 1H), 1.18 (dd, *J* = 16.7, 7.2 Hz, 3H), 1.08 (dd, *J* = 16.7, 7.2 Hz, 3H); ^{13}C NMR (151 MHz, $CDCl_3$) δ 156.1, 145.8 (d, *J* = 2.6 Hz), 145.7 (d, *J* = 15.7 Hz), 132.2 (d, *J* = 96.8 Hz), 131.5 (d, *J* = 2.2 Hz), 130.8 (d, *J* = 8.7 Hz), 128.6 (d, *J* = 11.1 Hz), 128.5, 125.4, 119.8, 119.4 (d, *J* = 94.9 Hz), 55.7, 29.1 (d, *J* = 74.7 Hz), 15.4 (d, *J* = 2.0 Hz), 15.2 (d, *J* = 2.1 Hz); ^{31}P NMR (243 MHz, $CDCl_3$) δ 36.3; HRMS (ESI) m/z 302.1305 (M + H^+), calc. for $C_{17}H_{21}NO_2P$ 302.1305.

The ee was determined by HPLC analysis: CHIRALPAK IC (4.6 mm i.d. x 250 mm); Hexane/2-propanol = 50/50; flow rate 2.0 mL/min; 25 °C; 254 nm; retention time: 9.1 min (minor) and 20.9 min (major).

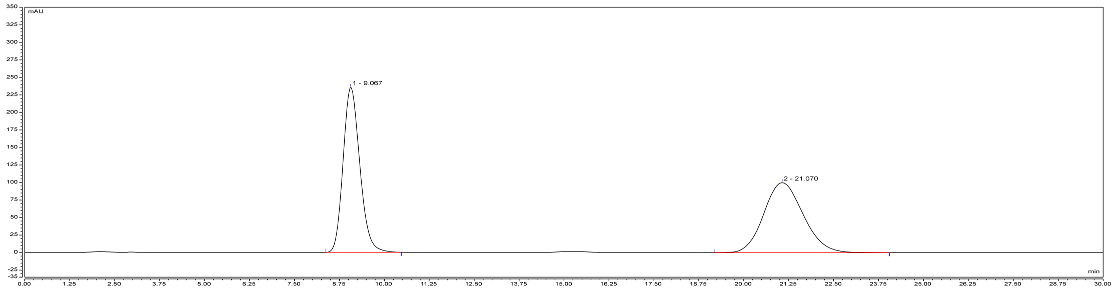

Entry	Retention Time	Area	Height	%Area
1	9.067	127.1981	235.40	50.14
2	21.070	126.4881	99.78	49.86

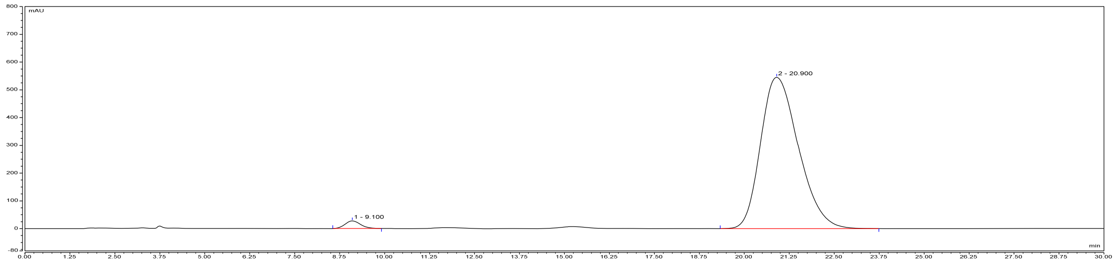

Entry	Retention Time	Area	Height	%Area
1	9.100	13.5345	27.46	1.99
2	20.900	665.6473	545.51	98.01

(*R*,*E*)-N,N-diethyl-6-(2-(isopropyl(phenyl)phosphoryl)vinyl)nicotinamide (35):

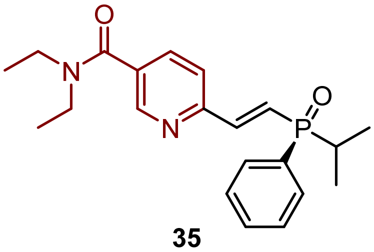

35

white solid; Mp 109.0-109.5 °C; 32.9 mg, 89% yield, 95% ee; $[\alpha]_D^{22}$ -21.6 (*c* 1.0, $CHCl_3$); ^1H NMR (600 MHz, $CDCl_3$) δ 8.62 (d, *J* = 2.0 Hz, 1H), 7.79 – 7.74 (m, 2H), 7.71 (dd, *J* = 7.9, 2.2 Hz, 1H), 7.57 (t, *J* = 17.1 Hz, 1H), 7.52 – 7.37 (m, 4H), 7.35 (d, *J* = 7.9 Hz, 1H), 3.53 (d, *J* = 6.4 Hz, 2H), 3.24 (d, *J* = 6.3 Hz, 2H), 2.18 (qd, *J* = 14.3, 7.2 Hz, 1H), 1.26 – 1.19 (m, 6H), 1.14 – 1.08 (m, 6H); ^{13}C NMR (151 MHz, $CDCl_3$) δ 168.2, 153.4 (d, *J* = 16.5 Hz), 147.6, 145.4 (d, *J* = 2.2 Hz), 135.5, 133.1, 131.8 (d, *J* = 2.1 Hz), 31.7 (d, *J* = 97.2 Hz), 130.9 (d, *J* = 8.7 Hz), 128.7 (d, *J* = 11.3 Hz), 124.2, 124.1 (d, *J* = 92.2 Hz), 43.5, 39.7, 29.1 (d, *J* = 74.6 Hz), 15.4 (d, *J* = 2.0 Hz), 15.2 (d, *J* = 2.5 Hz), 14.4, 12.9; ^{31}P NMR (243 MHz, $CDCl_3$) δ 36.0; HRMS (ESI) m/z 371.1883 (M + H^+), calc. for $C_{21}H_{28}FN_2O_2P$ 371.1884.

The ee was determined by HPLC analysis: CHIRALPAK AD-H (4.6 mm i.d. x 250 mm); Hexane/2-propanol = 80/20; flow rate 1.0 mL/min; 25 °C; 254 nm; retention time: 16.0 min (major) and 23.7 min (minor).

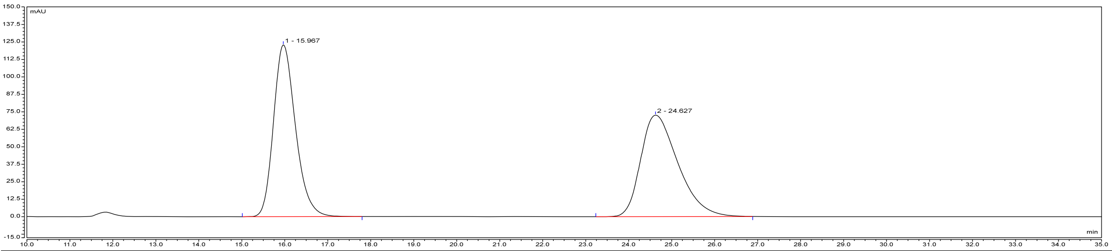

Entry	Retention Time	Area	Height	%Area
1	15.967	72.8743	122.80	49.99
2	24.627	72.9042	72.58	50.01

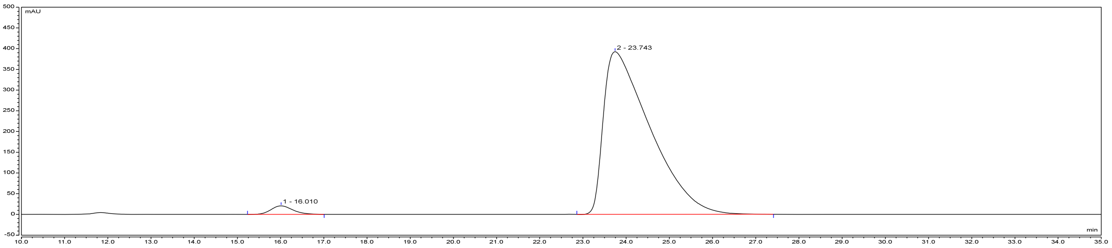

Entry	Retention Time	Area	Height	%Area
1	16.010	11.9980	20.59	2.38
2	23.743	493.0616	392.48	97.62

(*E*)-3,7-dimethylocta-2,6-dien-1-yl 6-((*E*)-2-((*R*) isopropyl(phenyl)phosphoryl)vinyl)nicotinate (36):

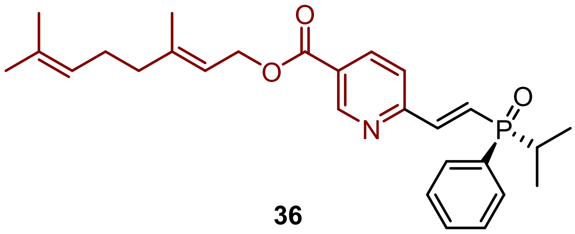

36

white solid; Mp 152.2-152.8 °C; 42.9 mg, 95% yield, 92% ee; $[\alpha]_D^{22}$ -55.0 (*c* 1.0, $CHCl_3$); ^{1}H NMR (600 MHz, $CDCl_3$) δ 9.19 (d, *J* = 1.6 Hz, 1H), 8.28 (dd, *J* = 8.0, 2.1 Hz, 1H), 7.78 (dd, *J* = 10.3, 7.7 Hz, 2H), 7.65 – 7.45 (m, 5H), 7.37 (d, *J* = 8.0 Hz, 1H), 5.46 (t, *J* = 7.2 Hz, 1H), 5.09 (t, *J* = 7.0 Hz, 1H), 4.82 (d, *J* = 7.3 Hz, 2H), 2.24 – 2.15 (m, 3H), 2.13 – 2.07 (m, 3H), 1.78 (s, 3H), 1.65 (s, 3H), 1.58 (s, 3H), 1.24 (dd, *J* = 16.7, 7.2 Hz, 4H), 1.13 (dd, *J* = 16.7, 7.2 Hz, 3H); ^{13}C NMR (151 MHz, $CDCl_3$) δ 165.0, 156.1 (d, *J* = 16.4 Hz), 151.2, 145.2 (d, *J* = 2.9 Hz), 143.6, 138.4, 132.4, 131.8 (d, *J* = 2.2 Hz), 131.6 (d, *J* = 97.5 Hz), 130.9 (d, *J* = 8.7 Hz), 128.8 (d, *J* = 11.2 Hz), 126.3, 125.8 (d, *J* = 91.0 Hz), 124.0, 123.6, 118.8, 62.2, 32.4, 29.1 (d, *J* = 74.7 Hz), 26.8, 25.8, 23.7, 17.8, 15.5 (d, *J* = 2.0 Hz), 15.2 (d, *J* = 2.1 Hz); ^{31}P NMR (243 MHz, $CDCl_3$) δ 35.8; HRMS (ESI) m/z 452.2350 (M + H^+), calc. for $C_{27}H_{35}NO_3P$ 452.2350.

The ee was determined by HPLC analysis: CHIRALPAK AD-H (4.6 mm i.d. x 250 mm); Hexane/2-propanol = 80/20; flow rate 1.0 mL/min; 25 °C; 254 nm; retention time: 8.5 min (major) and 10.9 min (minor).

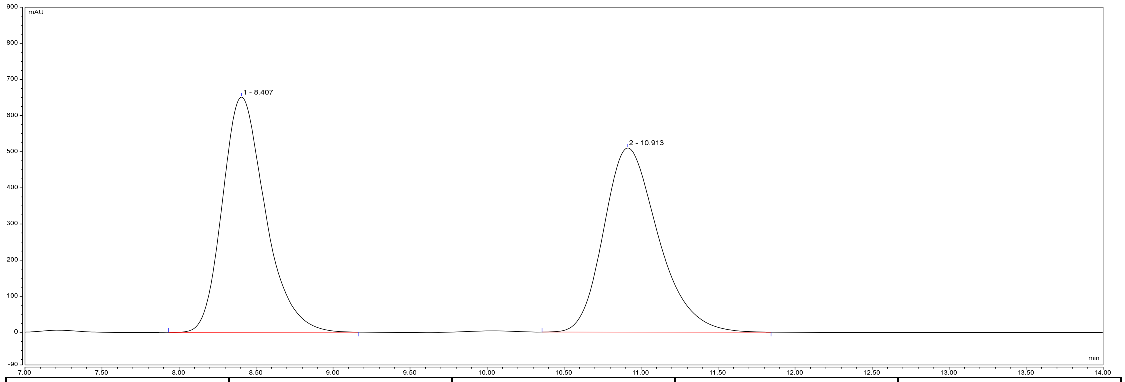

Entry	Retention Time	Area	Height	%Area
1	8.407	210.3042	651.13	50.08
2	10.913	209.6710	509.56	49.92

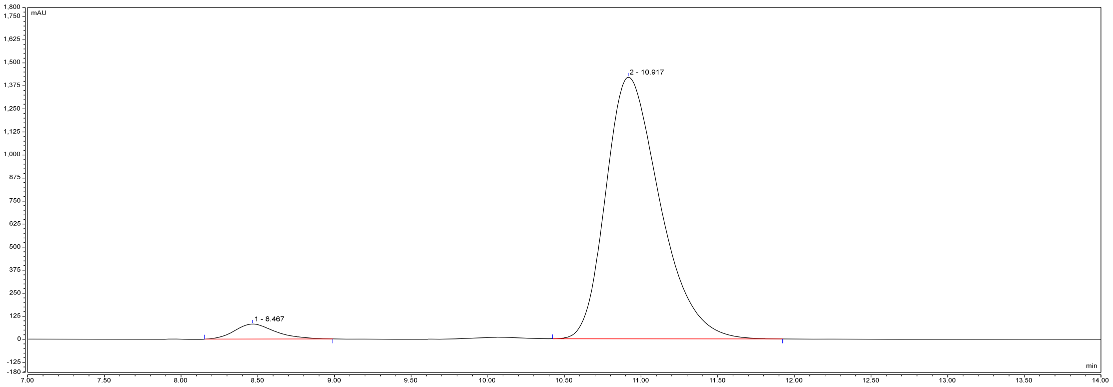

Entry	Retention Time	Area	Height	%Area
1	8.467	24.8491	81.31	4.10
2	10.917	581.1361	1419.01	95.90

(*R*,*E*)-6-(2-(isopropyl(phenyl)phosphoryl)vinyl)pyridin-3-yl 5-(2,5-dimethylphenoxy)-2,2-dimethylpentanoate (37):

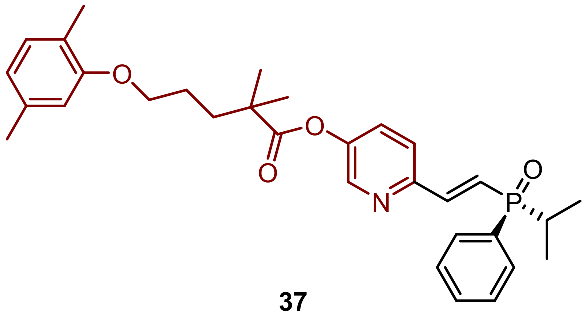

37

colorless oily liquid; 47.7 mg, 92% yield, 93% ee; $[\alpha]_D^{22}$ -26.4 (*c* 1.0, $CHCl_3$); ^1H NMR (600 MHz, $CDCl_3$) δ 8.38 (d, J = 2.6 Hz, 1H), 7.78 (ddd, J = 10.9, 8.0, 1.4 Hz, 2H), 7.58 (t, J = 17.1 Hz, 1H), 7.52 – 7.44 (m, 3H), 7.39 (dd, J = 8.4, 2.6 Hz, 1H), 7.37 – 7.29 (m, 2H), 6.98 (d, J = 7.5 Hz, 1H), 6.65 (d, J = 7.5 Hz, 1H), 6.60 (s, 1H), 3.97 (t, J = 5.8 Hz, 2H), 2.28 (s, 3H), 2.23 – 2.17 (m, 1H), 2.15 (s, 3H), 1.91 – 1.80 (m, 4H), 1.37 (s, 6H), 1.23 (dd, J = 16.7, 7.2 Hz, 3H), 1.13 (dd, J = 16.7, 7.2 Hz, 3H); ^{13}C NMR (151 MHz, $CDCl_3$) δ 175.8, 156.8, 150.2 (d, J = 16.7 Hz), 147.4, 145.3 (d, J = 2.9 Hz), 143.6, 136.5, 131.8 (d, J = 97.6 Hz), 131.7 (d, J = 2.6 Hz), 130.8 (d, J = 8.7 Hz), 130.4, 129.8, 128.7 (d, J = 11.3 Hz), 124.9, 123.6, 122.6 (d, J = 93.2 Hz), 120.9, 112.0, 67.6, 42.7, 37.1 29.0 (d, J = 74.7 Hz), 25.3, 25.1, 21.4, 15.8, 15.4 (d, J = 2.0 Hz), 15.1 (d, J = 2.2 Hz); ^{31}P NMR (243 MHz, $CDCl_3$) δ 36.3; HRMS (ESI) m/z 520.2612 (M + H^+), calc. for $C_{16}H_{19}NOP$ 520.2614.

The ee was determined by HPLC analysis: CHIRALPAK IE (4.6 mm i.d. x 250 mm); Hexane/2-propanol = 40/60; flow rate 1.0 mL/min; 25 °C; 254 nm; retention time: 20.7 min (minor) and 27.4 min (major).

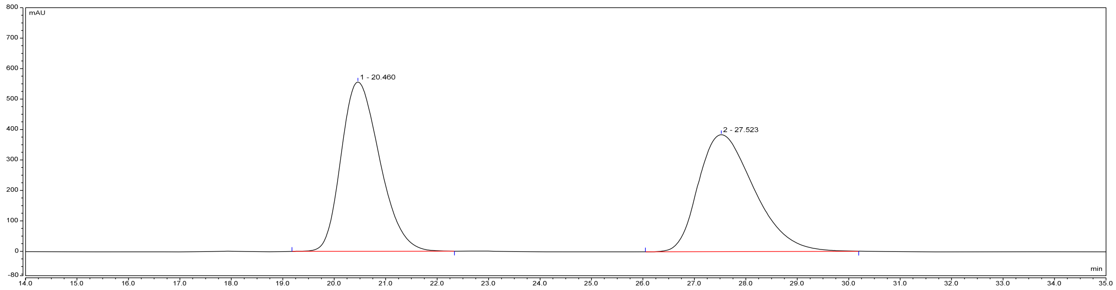

Entry	Retention Time	Area	Height	%Area
1	20.460	482.4665	555.76	49.97
2	27.523	482.9790	384.17	50.03

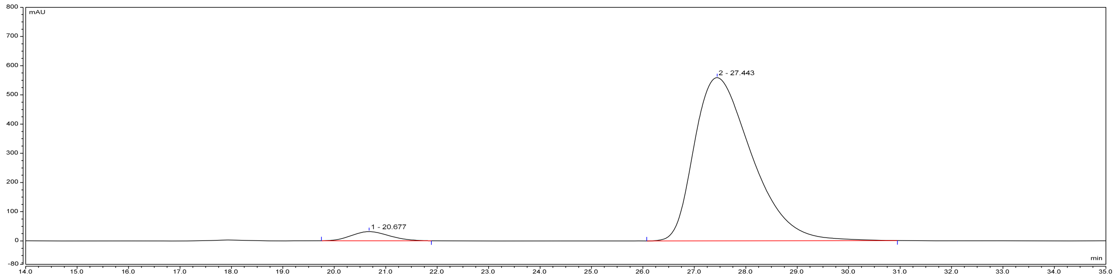

Entry	Retention Time	Area	Height	%Area
1	20.677	26.4651	31.27	3.53
2	27.443	723.8323	558.61	96.47

(*R*,*E*)-6-(2-(isopropyl(phenyl)phosphoryl)vinyl)picolinonitrile (38):

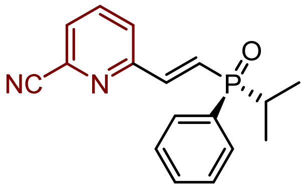

38

white solid; Mp 122.4-122.8 °C; 24.8 mg, 84% yield, 86% ee; $[\alpha]_D^{22}$ -65.6 (*c* 1.0, $CHCl_3$); ^{1}H NMR (400 MHz, $CDCl_3$) δ 7.83 (td, *J* = 7.8, 1.1 Hz, 1H), 7.79 – 7.73 (m, 2H), 7.60 (d, *J* = 7.7 Hz, 1H), 7.57 – 7.55 (m, 1H), 7.54 – 7.43 (m, 5H), 2.20 (dq, *J* = 14.7, 7.3 Hz, 1H), 1.22 (ddd, *J* = 16.8, 7.2, 1.2 Hz, 3H), 1.12 (ddd, *J* = 16.8, 7.2, 1.2 Hz, 3H); ^{13}C NMR (101 MHz, $CDCl_3$) δ 154.4 (d, *J* = 16.6 Hz), 143.9 (d, *J* = 2.9 Hz), 138.4, 134.1, 131.9 (d, *J* = 2.7 Hz), 131.4 (d, *J* = 97.7 Hz), 130.8 (d, *J* = 8.7 Hz), 128.8 (d, *J* = 11.2 Hz), 128.2, 127.4, 126.4 (d, *J* = 90.5 Hz), 117.1, 29.0 (d, *J* = 74.5 Hz), 15.4 (d, *J* = 2.3 Hz), 15.1 (d, *J* = 2.3 Hz); ^{31}P NMR (162 MHz, $CDCl_3$) δ 35.7; HRMS (ESI) m/z 297.1152 (M + H^+), calc. for $C_{16}H_{18}BrNOP$ 297.1152.

The ee was determined by HPLC analysis: CHIRALPAK IC (4.6 mm i.d. x 250 mm); Hexane/2-propanol = 40/60; flow rate 1.0 mL/min; 25 °C; 254 nm; retention time: 12.8 min (minor) and 21.7 min (major).

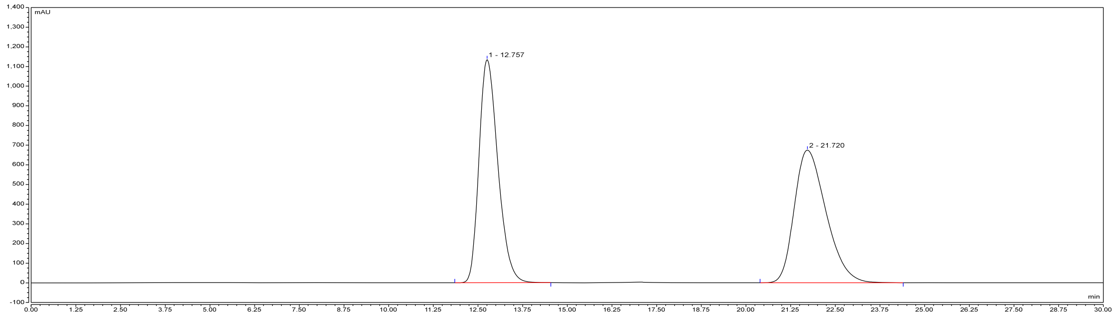

Entry	Retention Time	Area	Height	%Area
1	12.757	695.0159	1134.21	50.04
2	21.720	693.7752	674.56	49.96

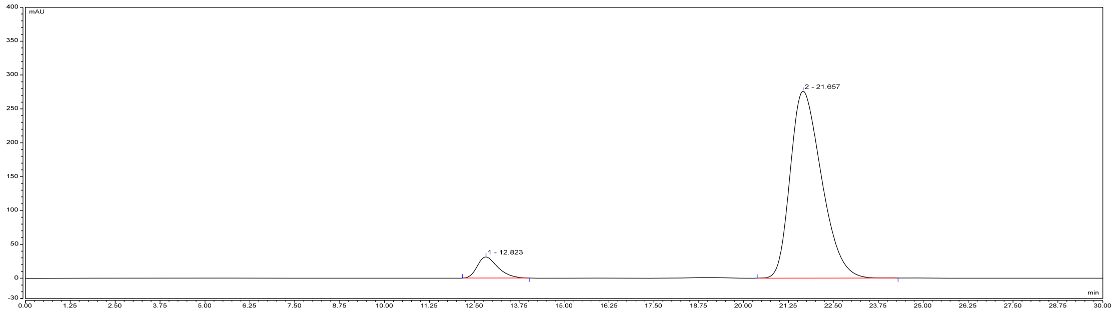

Entry	Retention Time	Area	Height	%Area
1	12.823	20.5796	31.20	6.88
2	21.657	278.7548	275.85	93.12

(*R*,*E*)-(2-(6-chloropyridin-2-yl)vinyl)(isopropyl)(phenyl)phosphine oxide (39):

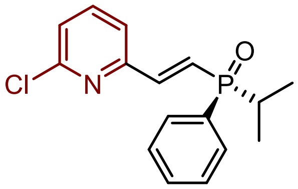

39

white solid; Mp 72.4-72.8 °C; 28.2 mg, 92% yield, 93% ee; $[\alpha]_D^{22}$ -58.6 (*c* 1.0, $CHCl_3$); ^{1}H NMR (400 MHz, $CDCl_3$) δ 7.85 – 7.71 (m, 2H), 7.63 (t, *J* = 7.7 Hz, 1H), 7.57 – 7.32 (m, 5H), 7.22 (dd, *J* = 12.6, 7.7 Hz, 2H), 2.17 (dq, *J* = 14.6, 7.3 Hz, 1H), 1.21 (dd, *J* = 16.7, 7.2 Hz,3H), 1.10 (dd, *J* = 16.7, 7.2 Hz, 3H); ^{13}C NMR (101 MHz, $CDCl_3$) δ 153.5 (d, *J* = 16.8 Hz), 151.8, 144.5 (d, *J* = 2.9 Hz), 139.7, 131.8 (d, *J* = 2.8 Hz), 131.7 (d, *J* = 97.5 Hz), 130.8 (d, *J* = 8.7 Hz), 128.7 (d, *J* = 11.5 Hz), 124.6 (d, *J* = 91.6 Hz), 124.8, 123.2, 29.0 (d, *J* = 74.4 Hz), 15.4 (d, *J* = 2.5 Hz), 15.1 (d, *J* = 2.6 Hz); ^{31}P NMR (162 MHz, $CDCl_3$) δ 35.9; HRMS (ESI) m/z 306.0810 (M + H^+), calc. for $C_{16}H_{18}ClNOP$ 306.0806. The ee was determined by HPLC analysis: CHIRALPAK IC (4.6 mm i.d. x 250 mm); Hexane/2-propanol = 40/60; flow rate 1.0 mL/min; 25 °C; 254 nm; retention time: 11.2 min (minor) and 34.0 min (major).

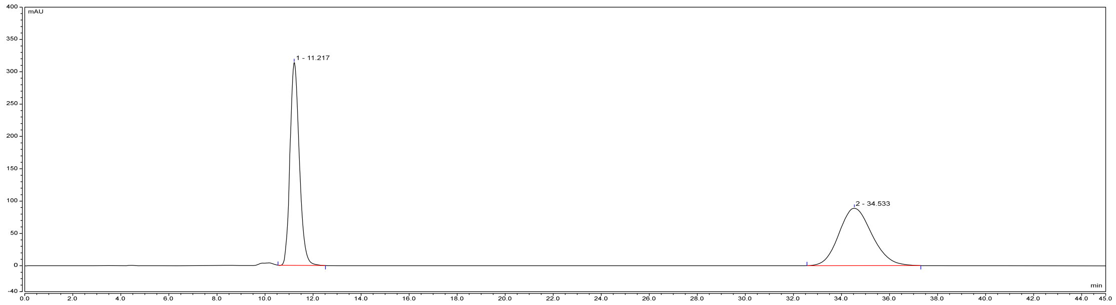

Entry	Retention Time	Area	Height	%Area
1	11.217	140.8302	313.63	49.89
2	34.533	141.4242	88.86	50.11

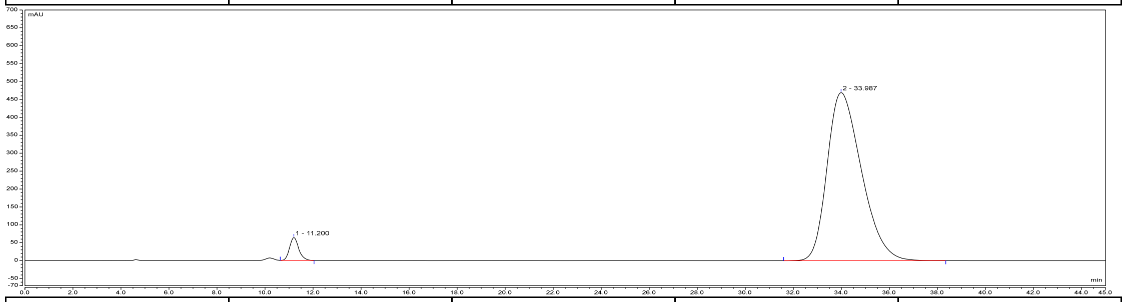

Entry	Retention Time	Area	Height	%Area
1	11.200	28.0670	63.43	3.49
2	33.987	775.2930	469.14	96.51

(*R*,*E*)-(2-(6-bromopyridin-2-yl)vinyl)(isopropyl)(phenyl)phosphine oxide (40):

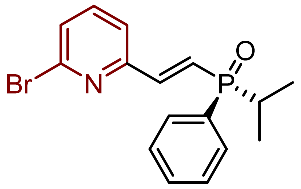

40

white solid; Mp 85.2-86.0 °C; 33.4 mg, 96% yield, 89% ee; $[\alpha]_D^{22}$ -136.3 (*c* 1.0, $CHCl_3$); ^{1}H NMR (600 MHz, $CDCl_3$) δ 7.81 – 7.73 (m, 2H), 7.57 – 7.31 (m,7H), 7.24 (d, *J* = 7.5 Hz, 1H), 2.21 – 2.14 (m, 1H), 1.22 (dd, *J* = 16.7, 7.1 Hz, 3H), 1.10 (dd, *J* = 16.7, 7.1 Hz, 3H); ^{13}C NMR (151 MHz, $CDCl_3$) δ 154.1 (d, *J* = 16.6 Hz), 144.4 (d, *J* = 2.9 Hz), 142.6, 139.3, 131.8 (d, *J* = 2.2 Hz), 131.7 (d, *J* = 97.2 Hz), 130.8 (d, *J* = 8.8 Hz), 128.7, 128.7 (d, *J* = 32.1 Hz), 124.8 (d, *J* = 91.6 Hz), 123.5, 29.0 (d, *J* = 74.7 Hz), 15.4 (d, *J* = 2.7 Hz), 15.1 (d, *J* = 2.7 Hz); ^{31}P NMR (243 MHz, $CDCl_3$) δ 35.8; HRMS (ESI) m/z 350.0304 (M + H^+), calc. for $C_{16}H_{18}BrNOP$ 350.0302.

The ee was determined by HPLC analysis: CHIRALPAK AD-H (4.6 mm i.d. x 250 mm); Hexane/2-propanol = 90/10; flow rate 1.0 mL/min; 25 °C; 254 nm; retention time: 12.5 min (minor) and 14.1 min (major).

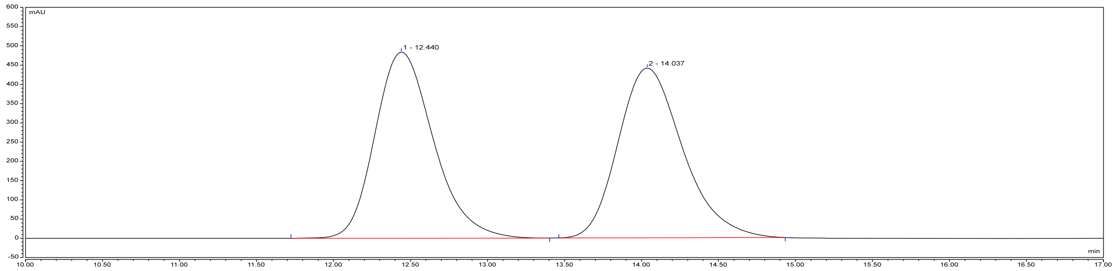

Entry	Retention Time	Area	Height	%Area
1	12.440	212.8953	483.35	49.82
2	14.037	214.4554	440.94	50.18

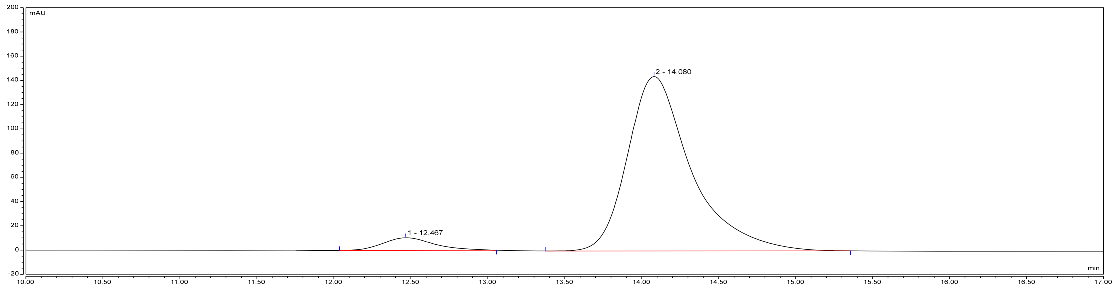

Entry	Retention Time	Area	Height	%Area
1	12.467	4.0051	10.44	5.48
2	14.080	69.0591	143.94	94.52

(*R*,*E*)-isopropyl(phenyl)(2-(quinolin-2-yl)vinyl)phosphine oxide (41):

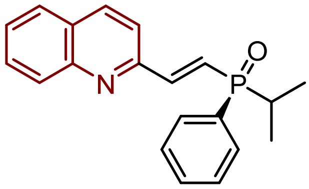

41

yellow solid; Mp 88.3-88.5 °C; 30.8 mg, 96% yield, 90% ee; $[\alpha]_D^{22}$ -63.2 (*c* 1.0, $CHCl_3$); ^{1}H NMR (600 MHz, $CDCl_3$) δ 8.16 (d, *J* = 8.4 Hz, 1H), 8.09 (d, *J* = 8.5 Hz, 1H), 7.84 (ddd, *J* = 10.9, 7.9, 1.5 Hz, 2H), 7.81 – 7.70 (m, 3H), 7.56 – 7.46 (m, 6H), 2.31 – 2.22 (m, 1H), 1.29 (dd, *J* = 16.7, 7.1 Hz, 3H), 1.16 (dd, *J* = 16.7, 7.1 Hz, 3H); ^{13}C NMR (151 MHz, $CDCl_3$) δ 153.1 (d, *J* = 16.8 Hz), 148.3, 146.8 (d, *J* = 2.9 Hz), 137.1, 131.9 (d, *J* = 2.2 Hz), 131.8 (d, *J* = 97.0 Hz), 131.0 (d, *J* = 8.6 Hz), 130.1, 129.8, 128.8 (d, *J* = 11.7 Hz), 128.3, 127.7, 127.2, 124.5 (d, *J* = 92.3 Hz), 121.6, 29.0 (d, *J* = 74.2 Hz), 15.5 (d, *J* = 2.7 Hz), 15.3 (d, *J* = 2.1 Hz); ^{31}P NMR (243 MHz, $CDCl_3$) δ 36.2; HRMS (ESI) m/z 322.1356 (M + H^+), calc. for $C_{20}H_{21}NOP$ 322.1355.

The ee was determined by HPLC analysis: CHIRALPAK IE (4.6 mm i.d. x 250 mm); Hexane/2-propanol = 40/60; flow rate 1.0 mL/min; 25 °C; 254 nm; retention time: 17.8 min (major) and 24.7 min (minor).

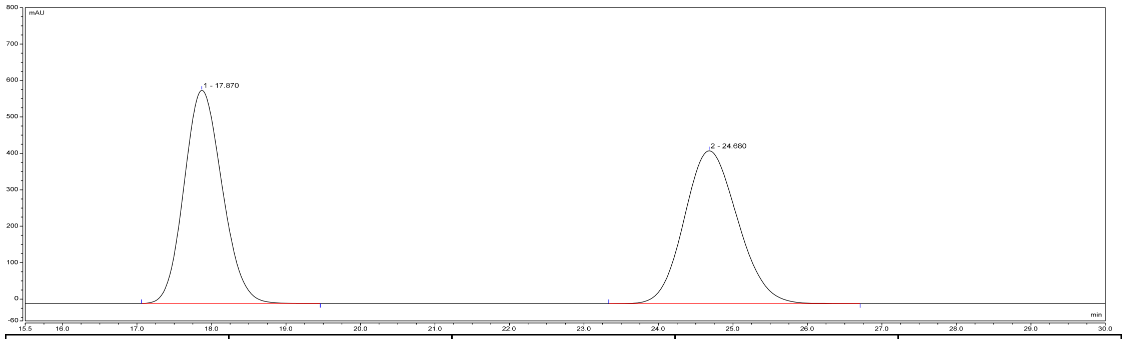

Entry	Retention Time	Area	Height	%Area
1	17.870	347.2697	585.09	49.94
2	24.680	348.1396	419.42	50.06

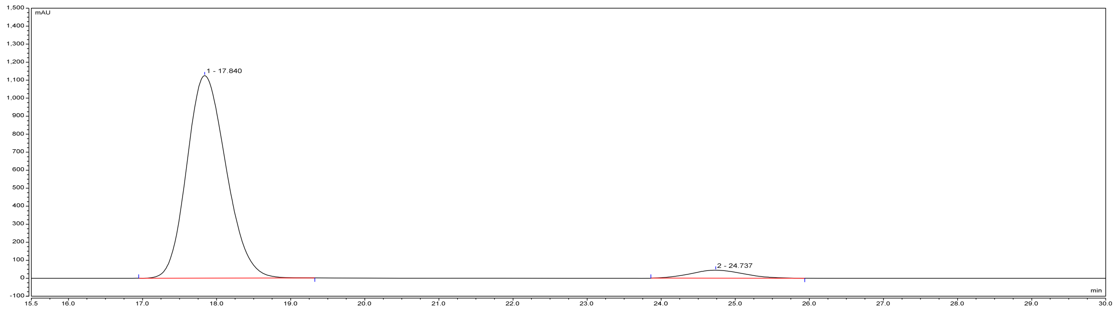

Entry	Retention Time	Area	Height	%Area
1	17.840	691.5149	1124.28	94.97
2	24.737	36.6614	44.45	5.03

(*R*,*E*)-isopropyl(2-(isoquinolin-3-yl)vinyl)(phenyl)phosphine oxide (42):

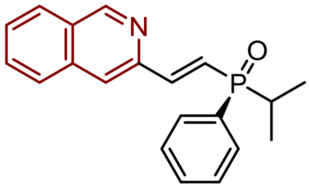

42

yellow oily liquid; 24.4 mg, 76% yield, 98% ee; $[\alpha]_D^{22}$ +46.8 (*c* 1.0, $CHCl_3$); ^{11}H NMR (600 MHz, $CDCl_3$) δ 9.26 (s, 1H), 7.99 (d, *J* = 8.1 Hz, 1H), 7.87 – 7.80 (m, 3H), 7.76 – 7.68 (m, 3H), 7.66 – 7.54 (m, 2H), 7.53 – 7.45 (m, 3H), 2.22 (dp, *J* = 21.5, 7.1 Hz, 1H), 1.25 (dd, *J* = 16.7, 7.1 Hz, 3H), 1.15 (dd, *J* = 16.7, 7.1 Hz, 3H); ^{13}C NMR (151 MHz, $CDCl_3$) δ 152.5, 146.3, 145.8, 136.5, 132.1 (d, *J* = 97.1 Hz), 131.7 (d, *J* = 2.0 Hz), 131.0 (d, *J* = 8.6 Hz), 128.8, 128.7, 128.6, 128.1, 127.5, 122.6, 122.3, 121.7, 29.3 (d, *J* = 74.7 Hz), 15.5 (d, *J* = 1.5 Hz), 15.3 (d, *J* = 2.0 Hz); ^{31}P NMR (243 MHz, $CDCl_3$) δ 36.5; HRMS (ESI) m/z 322.1356 ($M + H^+$), calc. for $C_{20}H_{21}NOP$ 322.1356.

The ee was determined by HPLC analysis: CHIRALPAK AD-H (4.6 mm i.d. x 250 mm); Hexane/2-propanol = 90/10; flow rate 1.0 mL/min; 25 °C; 254 nm; retention time: 30.4 min (major) and 35.0 min (minor).

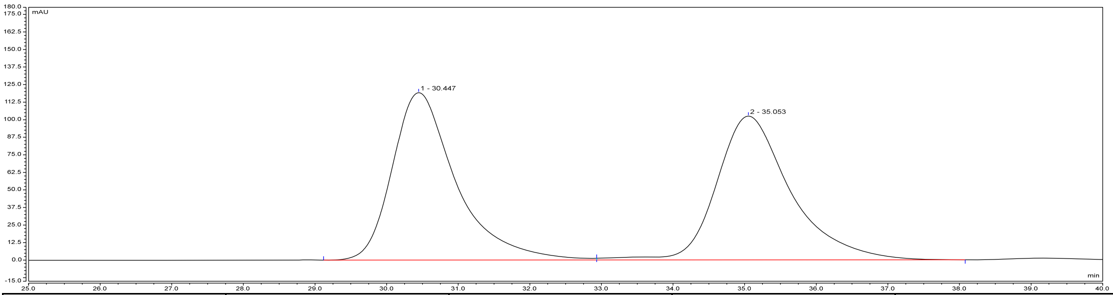

Entry	Retention Time	Area	Height	%Area
1	30.447	122.9108	119.16	50.06
2	35.053	122.6342	102.40	49.94

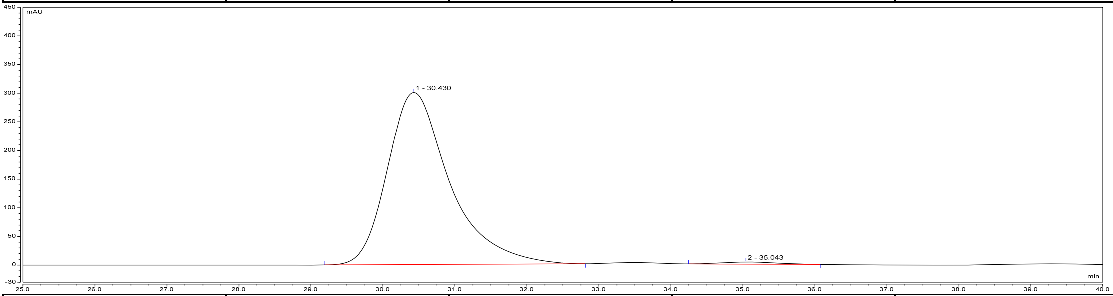

Entry	Retention Time	Area	Height	%Area
1	30.430	301.8335	300.40	98.93
2	35.043	3.2596	3.69	1.07

(*R*,*E*)-isopropyl(phenyl)(2-(pyrimidin-2-yl)vinyl)phosphine oxide (43):

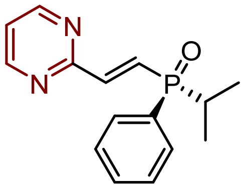

43

colorless oily liquid; 24.8 mg, 91% yield, 92% ee; $[\alpha]_D^{22}$ +23.4 (*c* 1.0, $CHCl_3$); ^{1}H NMR (600 MHz, $CDCl_3$) δ 8.74 (d, *J* = 4.9 Hz, 2H), 7.82 – 7.74 (m, 2H), 7.68 – 7.55 (m, 2H), 7.54 – 7.46 (m, 3H), 7.19 (t, *J* = 4.9 Hz, 1H), 2.25 – 2.19 (m, 1H), 1.26 (dd, *J* = 16.7, 7.2 Hz, 3H), 1.13 (dd, *J* = 16.7, 7.2 Hz, 3H); ^{13}C NMR (151 MHz, $CDCl_3$) δ 162.8 (d, *J* = 18.5 Hz), 157.4, 146.2 (d, *J* = 3.3 Hz), 131.8 (d, *J* = 2.8 Hz), 131.4 (d, *J* = 97.5 Hz), 131.0 (d, *J* = 8.7 Hz), 129.1 (d, *J* = 91.6 Hz), 128.8 (d, *J* = 11.2 Hz), 120.2, 29.0 (d, *J* = 74.7 Hz), 15.5 (d, *J* = 2.8 Hz), 15.2 (d, *J* = 2.1 Hz); ^{31}P NMR (243 MHz, $CDCl_3$) δ 35.3; HRMS (ESI) m/z 273.1152 (M + H^+), calc. for $C_{15}H_{18}N_2OP$ 273.1152.

The ee was determined by HPLC analysis: CHIRALPAK AD-H (4.6 mm i.d. x 250 mm); Hexane/2-propanol = 70/30; flow rate 1.0 mL/min; 25 °C; 254 nm; retention time: 11.3 min (major) and 17.4 min (minor).

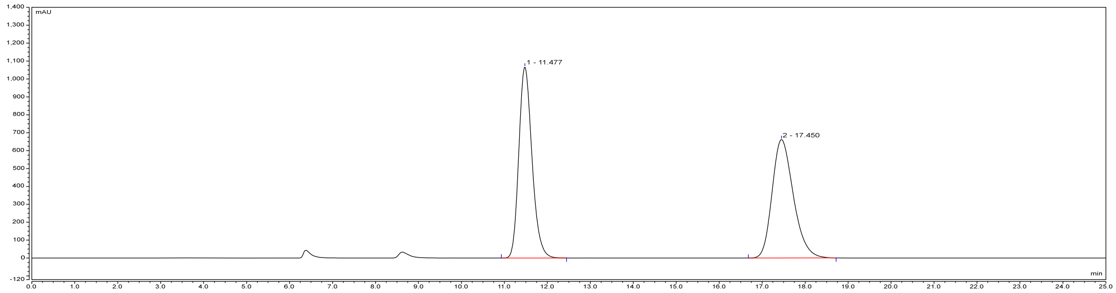

Entry	Retention Time	Area	Height	%Area
1	11.477	379.1231	1066.34	50.01
2	17.450	378.9887	661.20	49.99

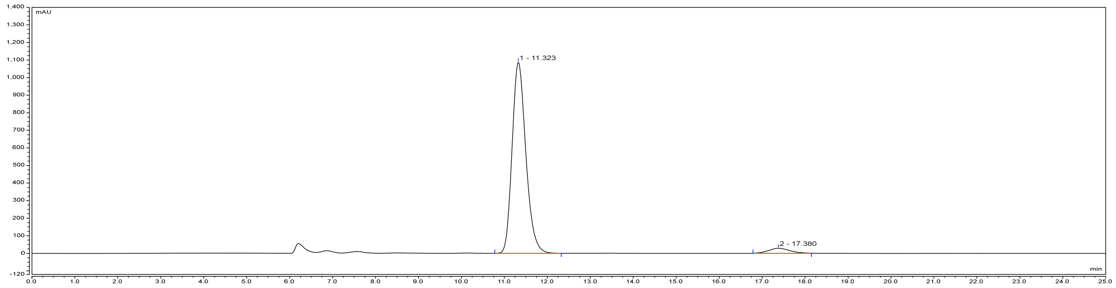

Entry	Retention Time	Area	Height	%Area
1	11.323	402.4324	1086.96	96.20
2	17.380	15.9056	28.46	3.80

(*R,E*)-5-(2-(isopropyl(phenyl)phosphoryl)vinyl)-1-methylpyrazin-2(1*H*)-one (44):

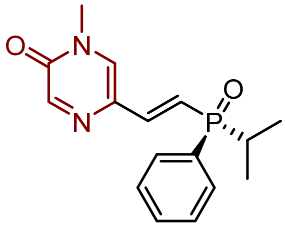

44

colorless oily liquid; 28.1 mg, 93% yield, 93% ee; $[\alpha]_D^{22}$ -104.6 (*c* 1.0, $CHCl_3$); ^1H NMR (600 MHz, $CDCl_3$) δ 8.13 (s, 1H), 7.74 – 7.69 (m, 2H), 7.50 – 7.42 (m, 3H), 7.30 – 7.23 (m, 2H), 6.97 (dd, *J* = 26.6, 16.6 Hz, 1H), 3.49 (s, 3H), 2.17 – 2.06 (m, 1H), 1.15 (dd, *J* = 16.8, 7.2 Hz, 3H), 1.07 (dd, *J* = 16.6, 7.1 Hz,3H); ^{13}C NMR (151 MHz, $CDCl_3$) δ 156.1, 148.8, 141.4 (d, *J* = 3.3 Hz), 132.1 (d, *J* = 97.4 Hz), 131.6 (d, *J* = 2.1 Hz), 131.0, 130.7 (d, *J* = 8.7 Hz), 130.5 (d, *J* = 17.7 Hz), 128.7 (d, *J* = 11.5 Hz), 117.7 (d, *J* = 95.9 Hz), 37.2, 29.2 (d, *J* = 74.8 Hz), 15.4 (d, *J* = 1.9 Hz), 15.1 (d, *J* = 2.1 Hz); ^{31}P NMR (243 MHz, $CDCl_3$) δ 36.6; HRMS (ESI) m/z 303.1257 (M + H^+), calc. for $C_{16}H_{20}N_2O_2P$ 303.1257.

The ee was determined by HPLC analysis: CHIRALPAK IE (4.6 mm i.d. x 250 mm); Hexane/2-propanol = 40/60; flow rate 1.0 mL/min; 25 °C; 254 nm; retention time: 16.9 min (minor) and 27.6 min (major).

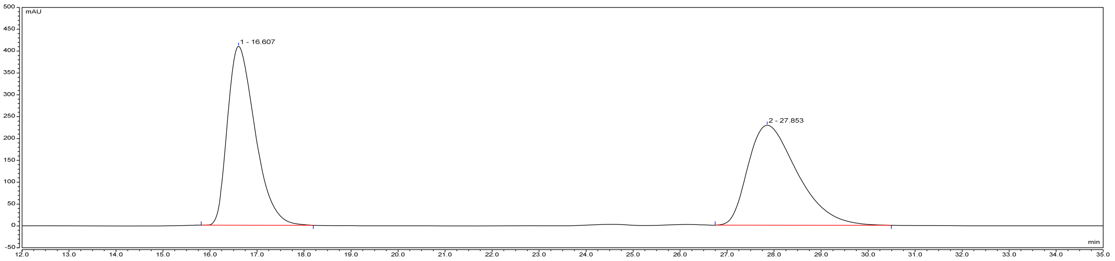

Entry	Retention Time	Area	Height	%Area
1	16.607	278.3407	409.01	50.00
2	27.853	278.3005	228.69	50.00

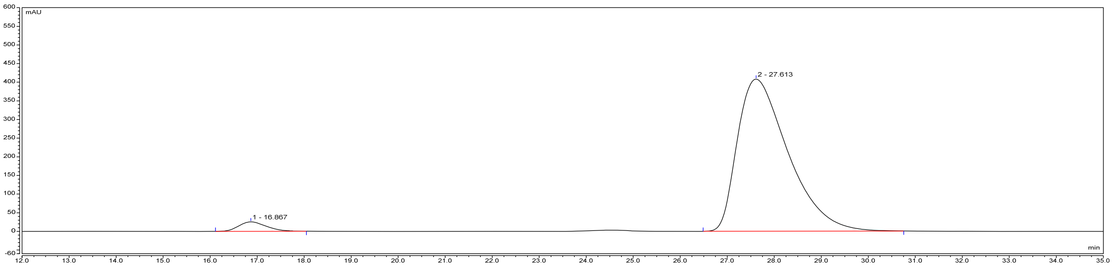

Entry	Retention Time	Area	Height	%Area
1	16.867	17.3306	25.44	3.29
2	27.613	510.1210	407.75	96.71

(*R*,*E*)-6-(2-(naphthalen-1-yl(phenyl)phosphoryl)vinyl)picolinonitrile (45):

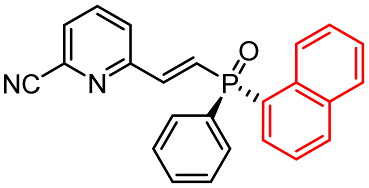

45

white solid; Mp 186.3-187.0 °C; 29.8 mg, 78% yield, 72% ee; $[\alpha]_D^{22}$ +95.2 (*c* 1.0, $CHCl_3$); ^{1}H NMR (600 MHz, $CDCl_3$) δ 8.42 (d, *J* = 8.3 Hz, 1H), 8.03 (d, *J* = 8.2 Hz, 1H), 7.96 (dd, *J* = 15.7, 7.0 Hz, 1H), 7.87 (d, *J* = 8.0 Hz, 1H), 7.85 – 7.77 (m, 2H), 7.75 (dd, *J* = 12.4, 7.2 Hz, 2H), 7.68 (dd, *J* = 18.3, 16.9 Hz, 1H), 7.58 (d, *J* = 7.6 Hz, 1H), 7.55 – 7.42 (m, 7H); ^{13}C NMR (151 MHz, $CDCl_3$) δ 154.5 (d, *J* = 18.1 Hz), 143.7 (d, *J* = 4.3 Hz), 138.3, 134.1, 133.9 (d, *J* = 9.3 Hz), 133.6 (d, *J* = 2.8 Hz), 133.3 (d, *J* = 55.2 Hz), 133.2 (d, *J* = 10.6 Hz), 132.9 (d, *J* = 41.6 Hz), 132.1 (d, *J* = 2.6 Hz), 131.1 (d, *J* = 10.3 Hz), 129.1, 128.9 (d, *J* = 12.3 Hz), 128.7, 128.7, 128.2, 128.0, 127.4 (d, *J* = 26.5 Hz), 126.7 (d, *J* = 5.7 Hz), 126.6, 124.6 (d, *J* = 14.1 Hz), 117.0; ^{31}P NMR (243 MHz, $CDCl_3$) δ 24.7; HRMS (ESI) m/z 381.1151 (M + H^+), calc. for $C_{24}H_{18}N_2OP$ 381.1151.

The ee was determined by HPLC analysis: CHIRALPAK IC (4.6 mm i.d. x 250 mm); Hexane/2-propanol = 50/50; flow rate 2.0 mL/min; 25 °C; 254 nm; retention time: 11.6 min (major) and 14.4 min (minor).

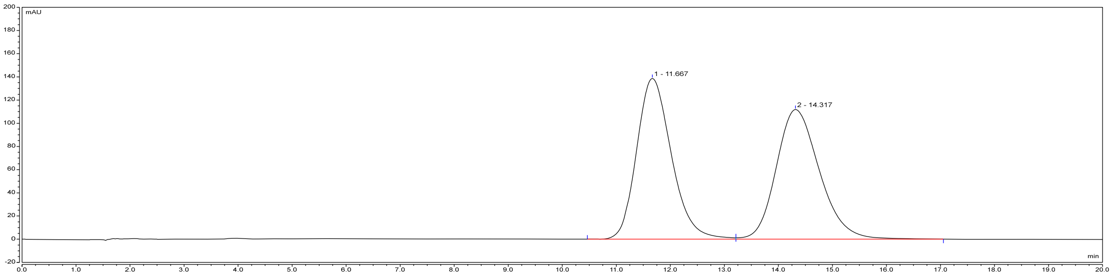

Entry	Retention Time	Area	Height	%Area
1	11.667	103.3173	138.53	49.96
2	14.317	103.4863	111.79	50.04

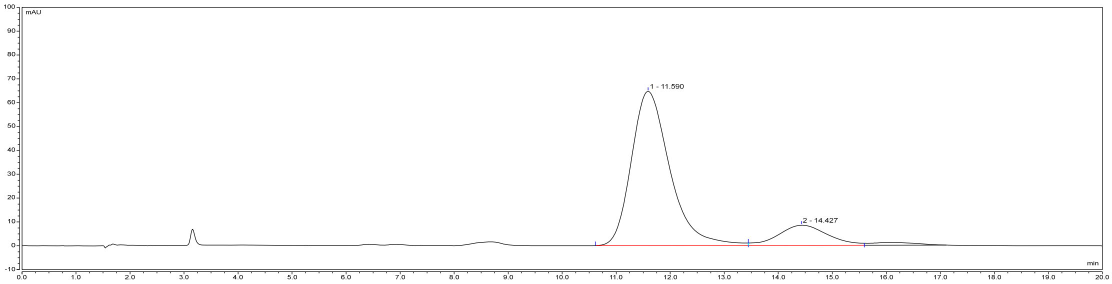

Entry	Retention Time	Area	Height	%Area
1	11.590	53.2152	64.63	85.82
2	14.427	8.7949	8.43	14.18

(*R*)-isopropyl(phenyl)phosphine oxide (*ent*-1):

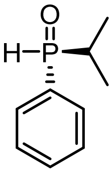

ent-**1**

colorless oily liquid; 4.4 mg, 26% yield, 94% ee; $[\alpha]_D^{22}$ +30.2 (*c* 1.0, $CHCl_3$); ^1H NMR (600 MHz, $CDCl_3$) δ 7.68 – 7.61 (m, 2H), 7.53 (td, *J* = 7.3, 1.4 Hz, 1H), 7.49 – 7.45 (m, 2H), 7.19 (dd, *J* = 457.2, 2.4 Hz, 1H), 2.14 – 2.06 (m, 1H), 1.13 (ddd, *J* = 20.2, 18.3, 7.2 Hz, 6H), ^{13}C NMR (151 MHz, $CDCl_3$) δ 132.5 (d, *J* = 2.8 Hz), 130.4 (d, *J* = 10.0 Hz), 129.7 (d, *J* = 93.1 Hz), 128.8 (d, *J* = 12.1 Hz), 28.7 (d, *J* = 69.1 Hz), 15.2, 14.6, ^{31}P NMR (243 MHz, $CDCl_3$) δ 39.5; HRMS (ESI) m/z 169.0777 (M + H^+), calc. for $C_9H_{14}OP$ 169.0778.

The ee was determined by HPLC analysis: CHIRALPAK IA (4.6 mm i.d. x 250 mm); Hexane/2-propanol = 90/10; flow rate 1.0 mL/min; 25 °C; 210 nm; retention time: 13.3 min (minor) and 14.4 min (major).

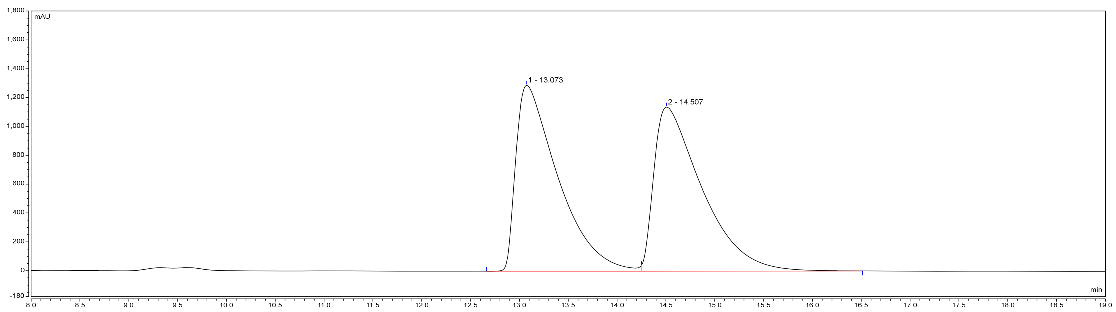

Entry	Retention Time	Area	Height	%Area
1	13.073	645.2550	1287.29	49.90
2	14.507	647.9343	1136.68	50.10

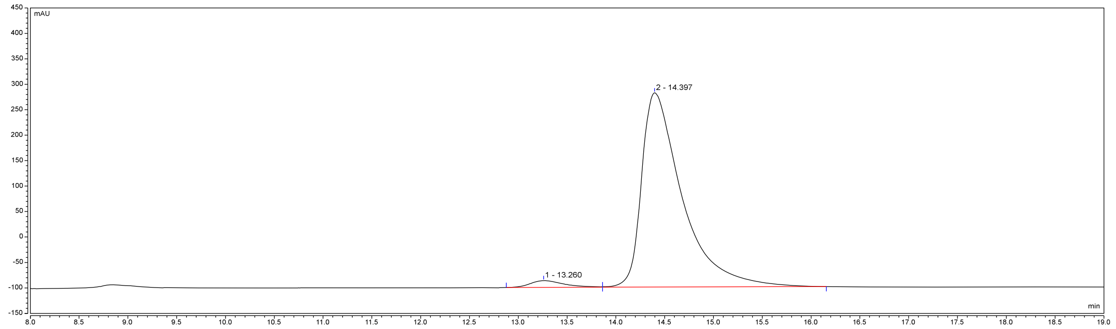

Entry	Retention Time	Area	Height	%Area
1	13.260	5.2816	13.33	2.79
2	14.397	184.3287	381.69	97.21

(*R*)-isopropyl(naphthalen-2-yl)phosphine oxide (*ent*-46):

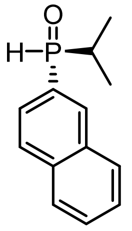

ent-**46**

colorless oily liquid; 5.9 mg, 27% yield, 99% ee; $[\alpha]_D^{22}$ -140.1 (*c* 1.0, $CHCl_3$); ^{1}H NMR (600 MHz, $CDCl_3$) δ 8.25 (d, *J* = 14.5 Hz, 1H), 7.96 – 7.89 (m, 2H), 7.86 (d, *J* = 8.1 Hz, 1H), 7.64 – 7.52 (m, 3H), 7.35 (dd, *J* = 457.6, 2.2 Hz, 1H), 2.22 – 2.14 (m, 1H), 1.17 (ddd, *J* = 33.1, 18.3, 7.2 Hz, 6H); ^{13}C NMR (151 MHz, $CDCl_3$) δ 135.1 (d, *J* = 2.3 Hz), 132.6 (d, *J* = 13.1 Hz), 132.5 (d, *J* = 9.4 Hz), 128.8, 128.7 (d, *J* = 11.9 Hz), 128.3, 128.0, 127.2, 126.9 (d, *J* = 92.9 Hz), 124.9 (d, *J* = 11.4 Hz), 28.8 (d, *J* = 69.2 Hz), 15.3, 14.6 (d, *J* = 1.6 Hz); ^{31}P NMR (243 MHz, $CDCl_3$) δ 39.1; HRMS (ESI) m/z 219.0933 (M + H^+), calc. for $C_{13}H_{16}OP$ 219.0936.

The ee was determined by HPLC analysis: CHIRALPAK IA (4.6 mm i.d. x 250 mm); Hexane/2-propanol = 90/10; flow rate 1.0 mL/min; 25 °C; 210 nm; retention time: 20.1 min (minor) and 22.5 min (major).

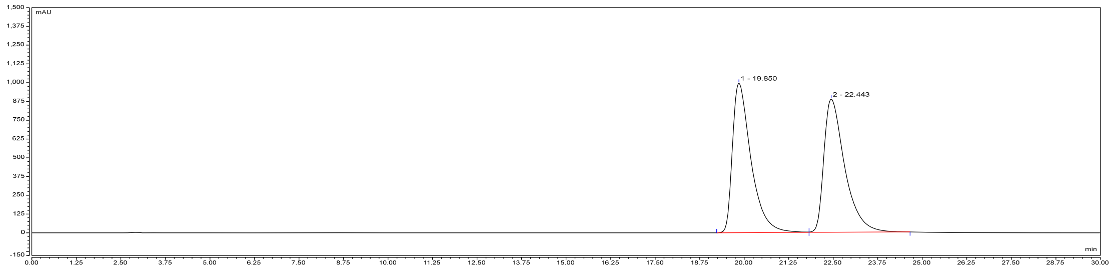

Entry	Retention Time	Area	Height	%Area
1	19.850	599.9935	995.24	49.99
2	22.443	600.3529	886.47	50.01

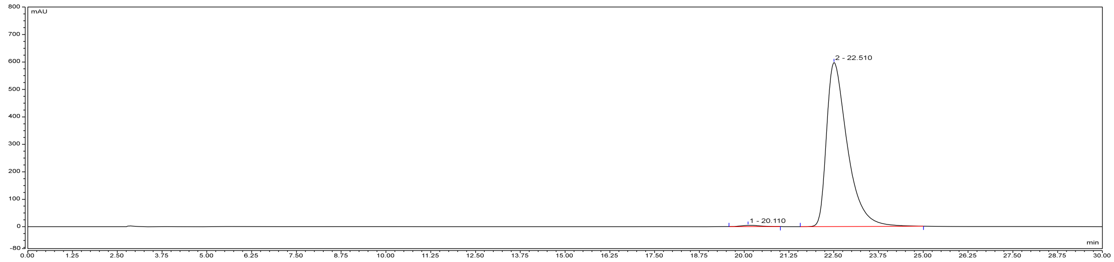

Entry	Retention Time	Area	Height	%Area
1	20.110	2.8985	4.97	0.71
2	22.510	402.6001	597.06	99.29

(*S*)-isopropyl((*S*)-1-((4-methoxyphenyl)thio)-2-(pyridin-2-yl)ethyl)(phenyl)phosphine oxide (47):

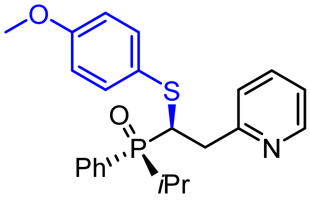

47

white solid; Mp 84.5-84.9 °C; 68.3 mg, 83% yield, 93% ee; $[\alpha]_D^{22}$ -28.4 (*c* 1.0, $CHCl_3$); ^{1}H NMR (400 MHz, $CDCl_3$) δ 8.54 (d, *J* = 4.3 Hz, 1H), 7.97 – 7.87 (m, 2H), 7.59 – 7.46 (m, 4H), 7.15 – 7.09 (m, 2H), 6.89 (d, *J* = 8.7 Hz, 2H), 6.62 (d, *J* = 8.8 Hz, 2H), 4.08 (td, *J* = 11.5, 2.9 Hz, 1H), 3.80 – 3.71 (m, 4H), 2.73 (dq, *J* = 14.2, 7.1 Hz, 1H), 2.59 (ddd, *J* = 14.5, 11.9, 4.9 Hz, 1H), 1.12 – 1.03 (m, 6H); ^{13}C NMR (101 MHz, $CDCl_3$) δ 159.6, 58.4 (d, *J* = 12.2 Hz), 149.4, 136.3, 134.4, 132.4 (d, *J* = 7.7 Hz), 132.0 (d, *J* = 2.4 Hz), 129.0 (d, *J* = 89.7 Hz), 128.4 (d, *J* = 10.8 Hz), 125.3 (d, *J* = 3.0 Hz) 124.9, 121.9, 114.6, 55.4, 46.9 (d, *J* = 60.4 Hz), 37.7, 24.7 (d, *J* = 70.6 Hz), 15.8 (d, *J* = 1.9 Hz), 15.0 (d, *J* = 3.0 Hz); ^{31}P NMR (162 MHz, $CDCl_3$) δ 48.0; HRMS (ESI) m/z 412.1495 (M + H^+), calc. for $C_{23}H_{27}NO_2PS$ 412.1496.

The ee was determined by HPLC analysis: CHIRALPAK IG (4.6 mm i.d. x 250 mm); Hexane/2-propanol = 40/60; flow rate 1.0 mL/min; 25 °C; 254 nm; retention time: 7.6 min (major) and 18.8 min (minor).

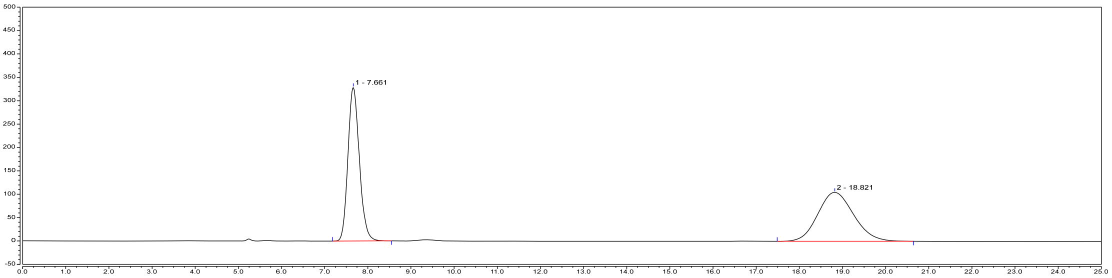

Entry	Retention Time	Area	Height	%Area
1	7.661	100.5722	328.56	50.08
2	18.821	100.2425	104.63	49.92

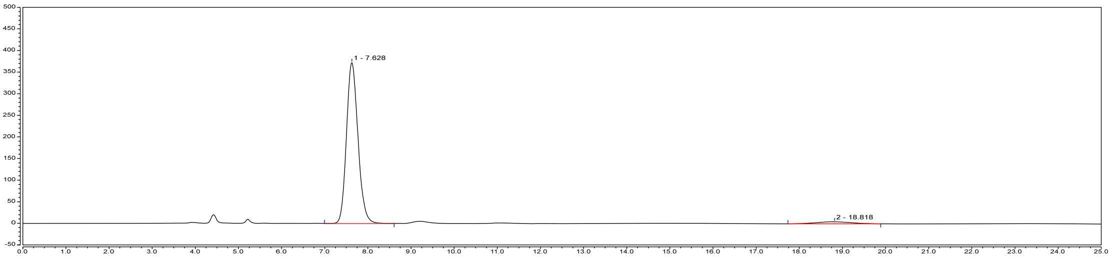

Entry	Retention Time	Area	Height	%Area
1	7.628	115.1116	372.50	96.43
2	18.818	4.2677	4.67	3.57

(*S*)-isopropyl((*R*)-1-((4-methoxyphenyl)thio)-2-(pyridin-2-yl)ethyl)(phenyl)phosphine oxide (*ent*-47):

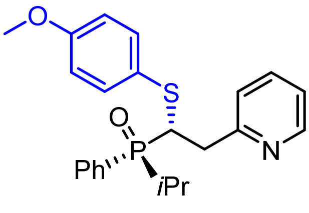

ent-**47**

white solid; Mp 84.8-85.4 °C; 8.2 mg, 10% yield, 93% ee; $[\alpha]_D^{22}$ -5.6 (*c* 1.0, $CHCl_3$); ^{1}H NMR (600 MHz, $CDCl_3$) δ 8.49 (dd, *J* = 4.8, 0.8 Hz, 1H), 7.85 – 7.77 (m, 2H), 7.51 – 7.41 (m, 4H), 7.19 – 7.14 (m, 2H), 7.08 (dd, *J* = 7.1, 5.1 Hz, 1H), 7.02 (d, *J* = 7.7 Hz, 1H), 6.70 – 6.67 (m, 2H), 4.22 (ddd, *J* = 11.2, 4.5, 3.6 Hz, 1H), 3.75 (s, 3H), 3.25 (ddd, *J* = 14.8, 7.2, 3.5 Hz, 1H), 3.04 (ddd, *J* = 14.9, 11.2, 5.9 Hz, 1H), 2.87 – 2.80 (m, 1H), 1.19 (dd, *J* = 15.6, 7.1 Hz, 3H), 0.94 (dd, *J* = 17.2, 7.1 Hz, 3H); ^{13}C NMR (151 MHz, $CDCl_3$) δ 159.7, 158.2 (d, *J* = 11.2 Hz), 149.4, 136.2, 135.3, 132.0 (d, *J* = 7.8 Hz), 131.8 (d, *J* = 2.8 Hz), 129.6 (d, *J* = 84.8 Hz), 128.5 (d, *J* = 10.2 Hz), 125.1, 124.5 (d, *J* = 2.9 Hz), 121.8,

114.5, 55.4, 45.6 (d, J = 63.6 Hz), 37.9, 26.7(d, J = 71.6 Hz), 16.5, 15.1 (d, J = 2.5 Hz); ^{31}P NMR (243 MHz, $CDCl_3$) δ 48.4; HRMS (ESI) m/z 412.1495 (M + H^+), calc. for $C_{23}H_{27}NO_2PS$ 412.1495.

The ee was determined by HPLC analysis: CHIRALPAK IG (4.6 mm i.d. x 250 mm); Hexane/2-propanol = 40/60; flow rate 1.0 mL/min; 25 °C; 254 nm; retention time: 9.5 min (minor) and 11.2 min (major).

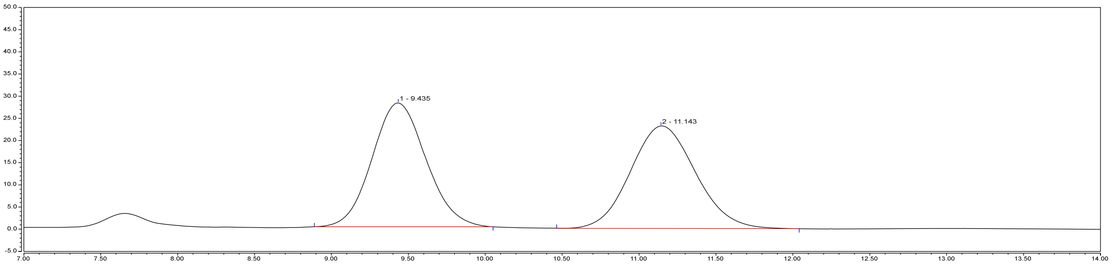

Entry	Retention Time	Area	Height	%Area
1	9.435	11.3675	27.92	50.06
2	11.143	11.3409	23.11	49.94

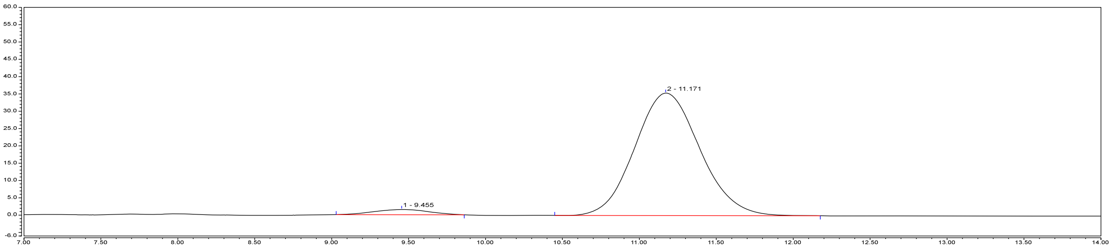

Entry	Retention Time	Area	Height	%Area
1	9.455	0.6208	1.53	3.44
2	11.171	17.4139	35.38	96.56

2-((*S*)-2-((*R*)-isopropyl(phenyl)phosphaneyl)-2-((4-methoxyphenyl)thio)ethyl)pyridine (48):

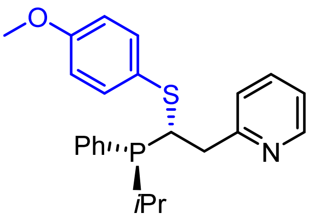

48

colorless oily liquid; 32.0 mg, 81% yield, 93% ee; $[\alpha]_D^{22}$ +30.1 (*c* 1.0, $CHCl_3$); ^{1}H NMR (600 MHz, $CDCl_3$) δ 8.49 (s, 1H), 7.75 – 7.62 (m, 2H), 7.54 – 7.34 (m, 4H), 7.12 (d, J = 7.4 Hz, 2H), 7.07 – 7.03 (m, 2H), 6.69 (d, J = 7.8 Hz, 2H), 3.93 (s, 1H), 3.75 (s, 3H), 3.21 – 3.11 (m, 1H), 2.93 (dd, J = 23.2, 11.9 Hz, 1H), 2.77 (d, J = 6.5 Hz, 1H), 1.21 – 1.15 (m, 3H), 1.01 – 0.95 (m, 3H); ^{13}C NMR (151 MHz, $CDCl_3$) δ 159.6 (d, J = 8.2 Hz), 159.106, 149.4, 136.1, 135.4 (d, J = 19.6 Hz), 134.4, 133.1 (d, J = 17.2 Hz),

129.8, 128.1 (d, J = 7.6 Hz), 126.8, 124.6, 121.5, 114.5, 55.4, 45.7 (d, J = 31.4 Hz), 42.9 (d, J = 16.8 Hz), 22.6 (d, J = 8.7 Hz), 19.5 (d, J = 19.7 Hz), 19.2 (d, J = 17.5 Hz); ^{31}P NMR (243 MHz, $CDCl_3$) δ 8.0; HRMS (ESI) m/z 396.1545 (M + H^+), calc. for $C_{23}H_{27}NOPS$ 396.1546. The ee was determined by HPLC analysis: CHIRALPAK OD-H (4.6 mm i.d. x 250 mm); Hexane/2-propanol = 95/5; flow rate 1.0 mL/min; 25 °C; 254 nm; retention time: 7.4 min (major) and 11.1 min (minor).

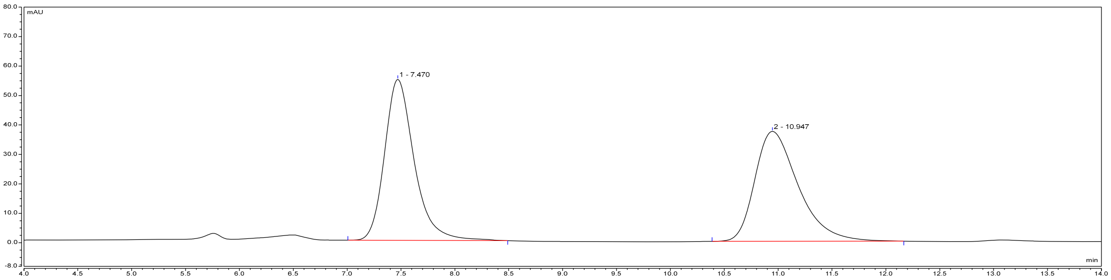

Entry	Retention Time	Area	Height	%Area
1	7.470	17.4349	54.62	50.01
2	10.947	17.4246	37.31	49.99

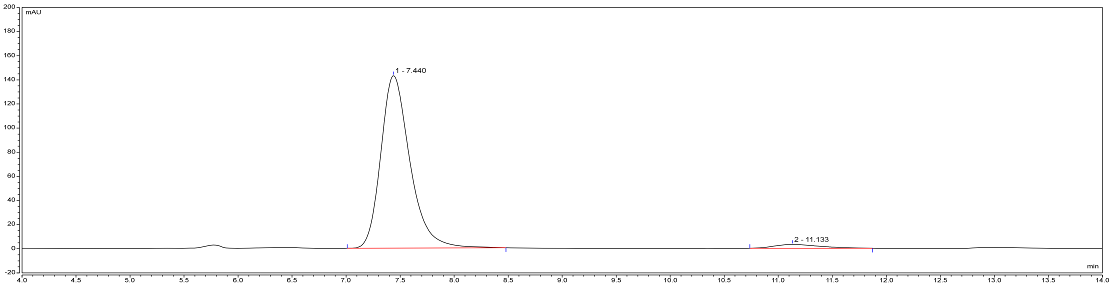

Entry	Retention Time	Area	Height	%Area
1	7.440	44.6578	142.93	96.63
2	11.133	1.5562	3.22	3.37

(*R*,*E*)-2-(2-(isopropyl(phenyl)phosphoryl)vinyl)pyridine 1-oxide (49):

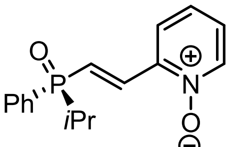

49

white solid; Mp 161.3-161.8 °C; 269.9 mg, 94% yield, 93% ee; $[\alpha]_D^{22}$ -24.3 (c 1.0, $CHCl_3$); ^{1}H NMR (400 MHz, $CDCl_3$) δ 8.24 – 8.08 (m, 2H), 7.81 – 7.73 (m, 2H), 7.58 – 7.41 (m, 5H), 7.25 – 7.15 (m, 2H), 2.26 – 2.16 (m, 1H), 1.23 (dd, J = 16.7, 7.1 Hz, 3H), 1.11 (dd, J = 16.7, 7.2 Hz, 3H); ^{13}C NMR (101 MHz, $CDCl_3$) δ 144.4 (d, J = 16.2 Hz), 141.0, 136.9 (d, J = 4.1 Hz), 131.8 (d, J = 2.5 Hz), 131.4 (d, J = 97.5 Hz), 131.0 (d, J = 8.7 Hz), 128.8 (d, J = 11.5 Hz), 128.3, 127.4, 125.4, 125.1, 28.9 (d, J = 74.2 Hz), 15.5 (d, J = 2.6 Hz), 15.2 (d, J = 2.3 Hz); ^{31}P NMR

(162 MHz, $CDCl_3$) δ 36.0; HRMS (ESI) m/z 288.1148 (M + H^+), calc. for $C_{16}H_{19}NO_2P$ 288.1143.

The ee was determined by HPLC analysis: CHIRALPAK AD-H (4.6 mm i.d. x 250 mm); Hexane/2-propanol = 80/20; flow rate 1.0 mL/min; 25 °C; 254 nm; retention time: 12.7 min (minor) and 15.6 min (major).

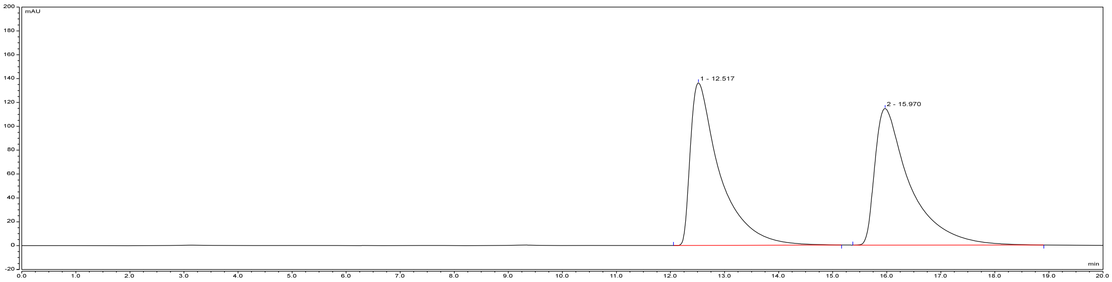

Entry	Retention Time	Area	Height	%Area
1	12.517	86.0599	136.23	49.97
2	15.970	86.1699	114.47	50.03

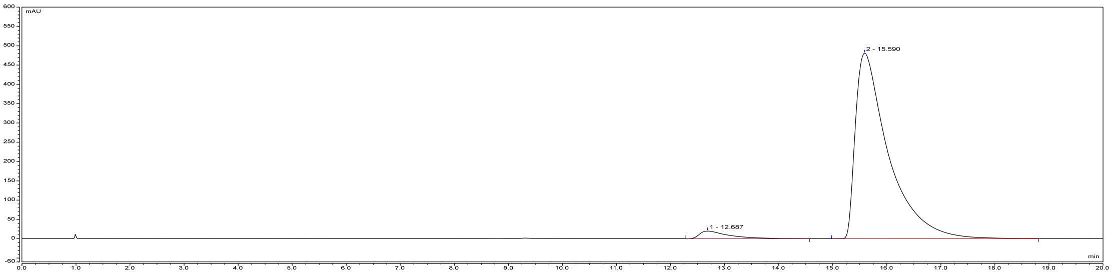

Entry	Retention Time	Area	Height	%Area
1	12.687	12.1924	19.76	3.47
2	15.590	339.2101	480.54	96.53

2-((4*R*,5*R*)-5-((*S*)-isopropyl(phenyl)phosphoryl)-2,2-dimethyl-1,3-dioxolan-4-yl)pyridine 1-oxide (50):

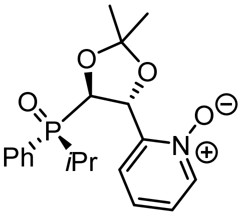

50

white solid; Mp 164.2-164.8 °C; 44.8 mg, 62% yield, 91% ee; $[\alpha]_D^{22}$ +4.3 (*c* 1.0, $CHCl_3$); ^1H NMR (600 MHz, $CDCl_3$) δ 8.20 (d, *J* = 6.3 Hz, 1H), 7.95 – 7.88 (m, 2H), 7.57 – 7.54 (m, 1H), 7.53 – 7.47 (m, 3H), 7.26 – 7.22 (m, 1H), 7.21 – 7.18 (m, 1H), 5.47 (t, *J* = 8.5 Hz, 1H), 4.75 (dd, *J* = 8.0, 3.6 Hz, 1H), 2.59 (dq, *J* = 14.6, 7.2 Hz, 1H), 1.53 (s, 3H), 1.36 – 1.30 (m, 6H), 1.02 (dd, *J* = 16.6, 7.2 Hz, 3H); ^{13}C NMR (151 MHz, $CDCl_3$) δ 147.9, 139.8, 132.5 (d, *J* = 7.8 Hz), 132.1 (d, *J* = 2.1 Hz), 128.2 (d, *J* = 91.5 Hz), 128.2 (d, *J* = 11.1 Hz),

125.3, 125.3, 152.1, 111.8 (d, J = 7.0 Hz), 75.9 (d, J = 79.8 Hz), 73.8 (d, J = 3.4 Hz), 27.0, 26.9, 26.6 (d, J = 69.3 Hz), 15.4 (d, J = 2.0 Hz), 15.2 (d, J = 3.3 Hz); ^{31}P NMR (243 MHz, $CDCl_3$) δ 42.2; HRMS (ESI) m/z 362.1516 (M + H^+), calc. for $C_{19}H_{25}NO_4P$ 362.1512.

The ee was determined by HPLC analysis: CHIRALPAK IG (4.6 mm i.d. x 250 mm); Hexane/2-propanol = 40/60; flow rate 1.0 mL/min; 25 °C; 254 nm; retention time: 10.8 min (major) and 12.7 min (minor).

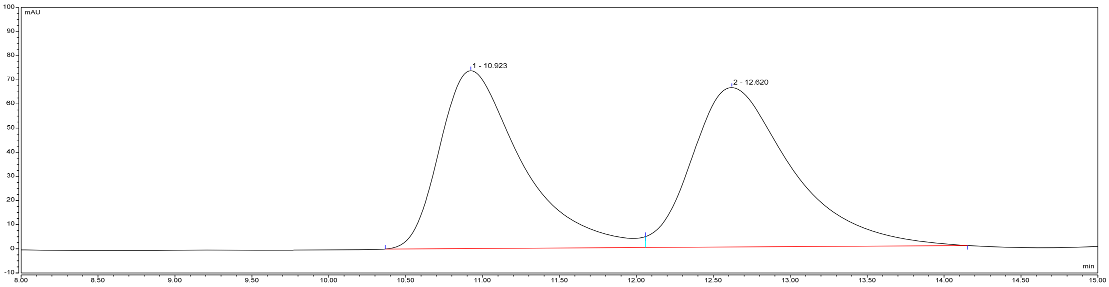

Entry	Retention Time	Area	Height	%Area
1	10.923	45.7724	73.66	48.05
2	12.620	49.4972	66.09	51.95

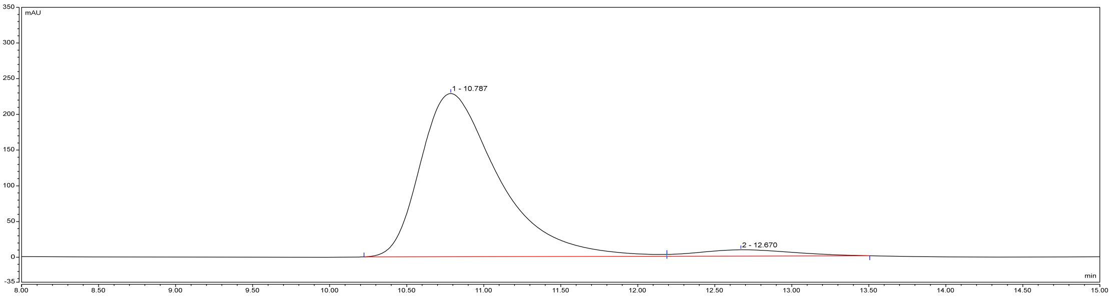

Entry	Retention Time	Area	Height	%Area
1	10.787	134.6657	228.44	95.52
2	12.670	6.3143	8.86	4.48

(*S*)-((4*R*,5*R*)-2,2-dimethyl-5-(pyridin-2-yl)-1,3-dioxolan-4-yl)(isopropyl)(phenyl)phosphine oxide (51):

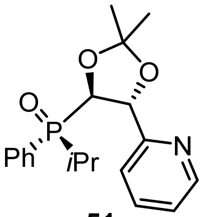

51

white solid; Mp 122.1-122.8 °C; 64.2 mg, 93% yield, 91% ee; $[\alpha]_D^{22}$ +5.5 (c 1.0, $CHCl_3$); ^{1}H NMR (600 MHz, $CDCl_3$) δ 8.63 (d, J = 4.4 Hz, 1H), 7.93 – 7.86 (m, 2H), 7.66 (td, J = 7.7, 1.8 Hz, 1H), 7.61 – 7.56 (m, 1H), 7.54 – 7.49 (m, 2H), 7.41 (d, J = 7.8 Hz, 1H), 7.20 (ddd, J = 7.5, 4.8, 0.9 Hz, 1H), 4.95 (dd, J = 8.5, 4.1 Hz, 1H), 4.89 (dd, J = 10.2, 8.6 Hz, 1H), 2.44 (dq, J =

14.3, 7.1 Hz, 1H), 1.49 (s, 3H), 1.37 – 1.31 (m, 6H), 0.99 (dd, J = 16.5, 7.3 Hz, 3H); ^{13}C NMR (151 MHz, $CDCl_3$) δ 156.9 (d, J = 2.7 Hz), 149.8, 136.7, 132.5 (d, J = 7.8 Hz), 132.2 (d, J = 2.2 Hz), 128.4 (d, J = 10.9 Hz), 128.4 (d, J = 90.8 Hz), 123.4, 123.2, 111.8 (d, J = 8.5 Hz), 79.9 (d, J = 3.3 Hz), 75.5 (d, J = 83.0 Hz), 26.6, 26.3 (d, J = 69.0 Hz), 15.0 (d, J = 3.3 Hz), 14.9 (d, J = 3.0 Hz); ^{31}P NMR (243 MHz, $CDCl_3$) δ 42.4; HRMS (ESI) m/z 346.1567 (M + H^+), calc. for $C_{19}H_{25}NO_4P$ 346.1565.

The ee was determined by HPLC analysis: CHIRALPAK AD-H (4.6 mm i.d. x 250 mm); Hexane/2-propanol = 90/10; flow rate 1.0 mL/min; 25 °C; 254 nm; retention time: 8.1 min (major) and 10.3 min (minor).

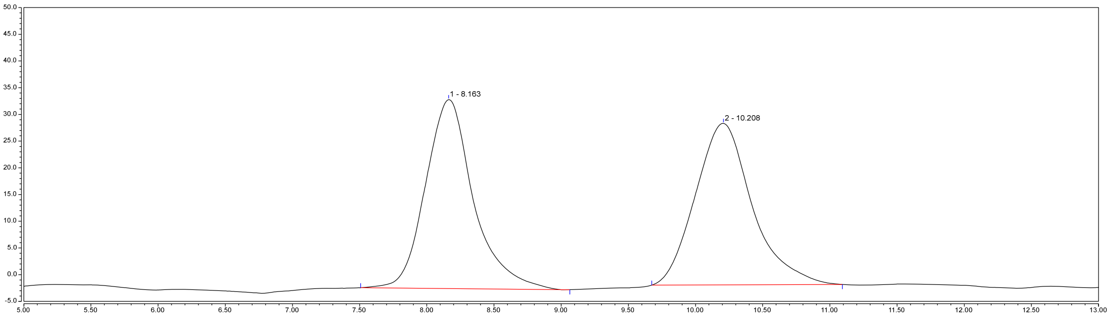

Entry	Retention Time	Area	Height	%Area
1	8.163	14.6567	35.38	49.72
2	10.208	14.8213	30.24	50.28

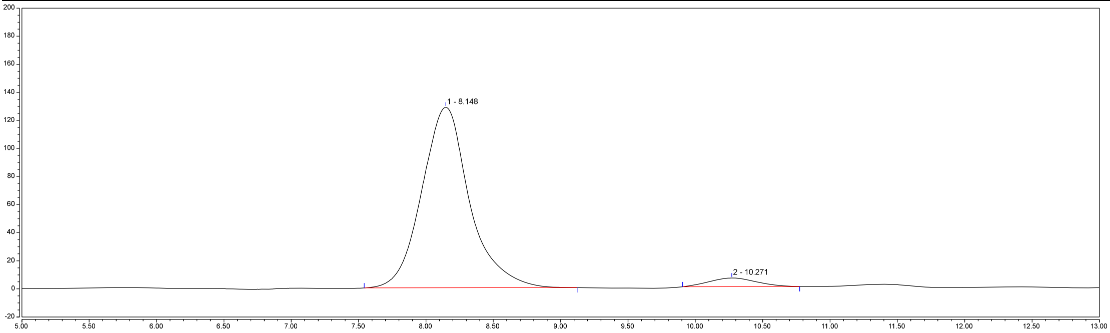

Entry	Retention Time	Area	Height	%Area
1	8.148	52.8549	128.70	95.48
2	10.271	2.5005	6.19	4.52

2-((4*R*,5*R*)-5-((*R*)-isopropyl(phenyl)phosphaneyl)-2,2-dimethyl-1,3-dioxolan-4-yl)pyridine-borane (52):

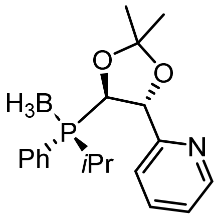

52

colorless oily liquid; 28.5 mg, 83% yield, 88% ee; $[\alpha]_D^{22}$ +8.0 (*c* 1.0, $CHCl_3$); ^{1}H NMR (600 MHz, $CDCl_3$) δ 8.65 (d, *J* = 4.4 Hz, 1H), 7.94 (t, *J* = 8.5 Hz, 2H), 7.66 (t, *J* = 7.1 Hz, 1H), 7.58 (t, *J* = 7.1 Hz, 1H), 7.51 (t, *J* = 6.8 Hz, 2H), 7.27 (d, *J* = 7.7 Hz, 1H), 7.24 (dd, *J* = 7.1, 5.1 Hz, 1H), 5.20 – 5.08 (m, 1H), 4.58 (t, *J* = 9.2 Hz, 1H), 2.81 – 2.70 (m, 1H), 1.55 (s, 3H), 1.34 (dd, *J* = 16.0, 7.0 Hz, 3H), 1.31 (s, 3H), 0.95 (dd, *J* = 16.7, 7.1 Hz, 3H), 0.59 – -0.19 (br, 3H); ^{13}C NMR (101 MHz, $CDCl_3$) δ 155.8 (d, *J* = 1.2 Hz), 149.8, 136.7, 134.4 (d, *J* = 8.2 Hz), 132.2 (d, *J* = 2.5 Hz), 128.6 (d, *J* = 9.6 Hz), 124.6, 124.4 (d, *J* = 50.3 Hz), 123.8, 111.7 (d, *J* = 5.9 Hz), 80.8 (d, *J* = 6.4 Hz), 74.9 (d, *J* = 37.8 Hz), 26.9, 26.8, 22.0 (d, *J* = 36.9 Hz), 16.8 (d, *J* = 1.8 Hz), 16.4 (d, *J* = 2.1 Hz); ^{31}P NMR (162 MHz, $CDCl_3$) δ 26.4 (d, *J* = 54.4 Hz); HRMS (ESI) m/z 344.1945 (M + H^+), calc. for $C_{23}H_{27}NOPS$ 344.1946.

The ee was determined by HPLC analysis: CHIRALPAK OD-H (4.6 mm i.d. x 250 mm); Hexane/2-propanol = 95/5; flow rate 1.0 mL/min; 25 °C; 254 nm; retention time: 5.0 min (major) and 10.4 min (minor).

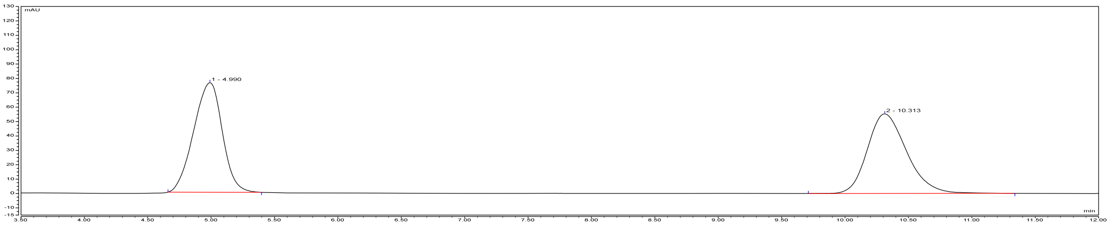

Entry	Retention Time	Area	Height	%Area
1	4.990	19.9815	76.15	50.19
2	10.313	19.8313	55.34	49.81

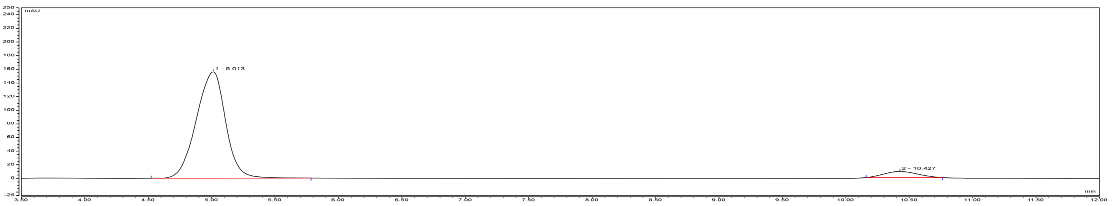

Entry	Retention Time	Area	Height	%Area
1	5.013	41.6836	155.91	93.84
2	10.427	2.7376	8.96	6.16

2-((4*S*,5*S*)-5-((*S*)-isopropyl(phenyl)phosphoryl)-2,2-dimethyl-1,3-dioxolan-4-yl)pyridine 1-oxide (53):

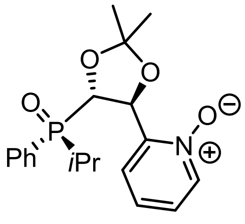

53

white solid; Mp 163.5-164.2 °C; 54.9 mg, 76% yield, 99% ee; $[\alpha]_D^{22}$ -25.5 (*c* 1.0, $CHCl_3$); ^1H NMR (600 MHz, $CDCl_3$) δ 8.11 – 8.07 (m, 1H), 7.90 – 7.85 (m, 2H), 7.49 – 7.44 (m, 1H), 7.41 (td, *J* = 7.4, 2.6 Hz, 2H), 7.35 (dd, *J* = 6.8, 3.1 Hz, 1H), 7.19 – 7.13 (m, 2H), 5.67 (t, *J* = 8.8 Hz, 1H), 4.94 (t, *J* = 8.2 Hz, 1H), 2.71 (dq, *J* = 14.7, 7.2 Hz, 1H), 1.58 (s, 3H), 1.30 (s, 3H), 1.09 (ddd, *J* = 11.5, 7.1, 3.8 Hz, 6H); ^{13}C NMR (151 MHz, $CDCl_3$) δ 147.5, 139.9, 132.0(d, *J* = 7.7 Hz), 132.0, 128.9, 128.3 (d, *J* = 11.1 Hz), 126.2, 125.7, 125.5, 112.3 (d, *J* = 6.5 Hz), 77.0 (d, *J* = 76.9 Hz), 73.6 (d, *J* = 4.9 Hz), 27.0, 26.5, 26.3 (d, *J* = 68.5 Hz), 16.0 (d, *J* = 2.7 Hz), 15.2 (d, *J* = 3.3 Hz); ^{31}P NMR (243 MHz, $CDCl_3$) δ 40.3; HRMS (ESI) m/z 362.1516 (M + H^+), calc. for $C_{19}H_{25}NO_4P$ 362.1515.

The ee was determined by HPLC analysis: CHIRALPAK IG (4.6 mm i.d. x 250 mm); Hexane/2-propanol = 40/60; flow rate 1.0 mL/min; 25 °C; 254 nm; retention time: 8.1 min (major) and 27.3 min (minor).

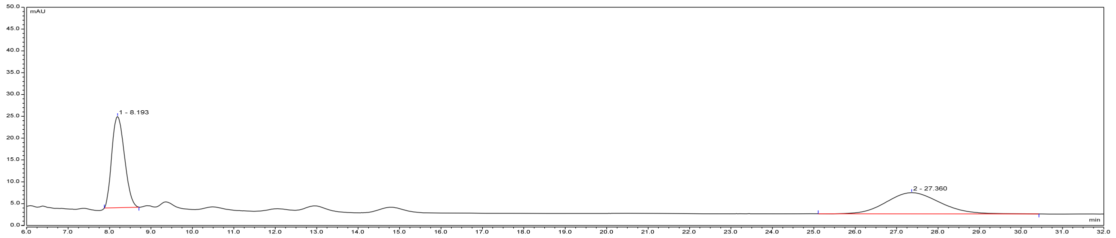

Entry	Retention Time	Area	Height	%Area
1	8.193	7.4302	20.87	50.54
2	27.360	7.2714	4.85	49.46

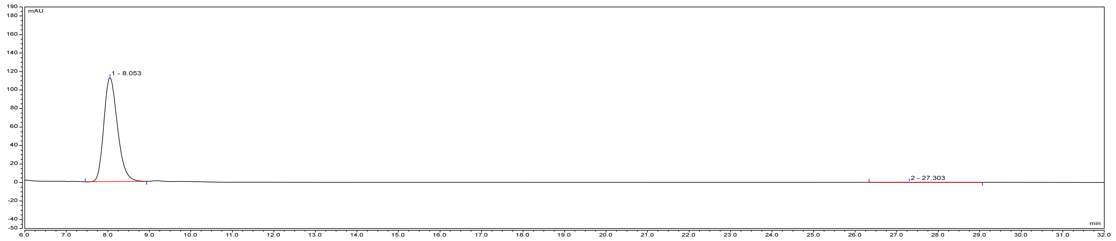

Entry	Retention Time	Area	Height	%Area
1	8.053	42.7307	112.49	99.47
2	27.303	0.2295	0.18	0.53

(*S*)-((4*S*,5*S*)-2,2-dimethyl-5-(pyridin-2-yl)-1,3-dioxolan-4-yl)(isopropyl)(phenyl)phosphine oxide (54):

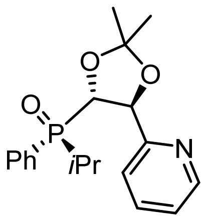

54

white solid; Mp 121.5-122.2 °C; 63.5 mg, 92% yield, 97% ee; $[\alpha]_D^{22}$ -21.5 (*c* 1.0, $CHCl_3$); ^1H NMR (400 MHz, $CDCl_3$) δ 8.43 (d, *J* = 4.3 Hz, 1H), 7.56 – 7.48 (m, 2H), 7.38 (td, *J* = 7.7, 1.8 Hz, 1H), 7.33 (td, *J* = 7.5, 1.3 Hz, 1H), 7.22 (td, *J* = 7.5, 2.7 Hz, 2H), 7.06 – 7.01 (m, 2H), 5.35 – 5.30 (m, 1H), 4.94 (dd, *J* = 13.2, 8.7 Hz, 1H), 2.49 – 2.39 (m, 1H), 1.58 (s, 3H), 1.54 (s, 3H), 1.21 (dd, *J* = 15.8, 7.1 Hz, 3H), 1.10 (dd, *J* = 17.1, 7.2 Hz, 3H); ^{13}C NMR (101 MHz, $CDCl_3$) δ 156.2 (d, *J* = 2.2 Hz), 149.6, 136.5, 131.6 (d, *J* = 2.5 Hz), 131.5 (d, *J* = 8.0 Hz), 129.0 (d, *J* = 87.8 Hz), 128.1 (d, *J* = 10.8 Hz), 123.5, 123.2, 112.3 (d, *J* = 6.9 Hz), 79.1 (d, *J* = 3.8 Hz, 78.3, 27.5 (d, *J* = 69.4 Hz), 26.9, 26.6, 16.1 (d, *J* = 1.7 Hz), 15.1 (d, *J* = 3.5 Hz); ^{31}P NMR (162 MHz, $CDCl_3$) δ 38.1; HRMS (ESI) m/z 346.1567 (M + H^+), calc. for $C_{19}H_{25}NO_4P$ 346.1564.

The ee was determined by HPLC analysis: CHIRALPAK AD-H (4.6 mm i.d. x 250 mm); Hexane/2-propanol = 80/20; flow rate 1.0 mL/min; 25 °C; 254 nm; retention time: 5.4 min (major) and 9.1 min (minor).

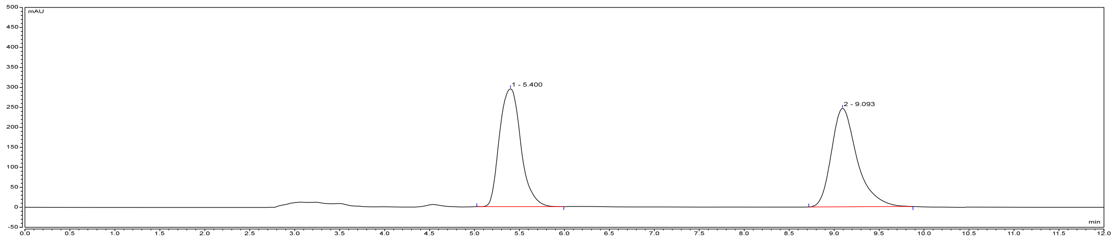

Entry	Retention Time	Area	Height	%Area
1	5.400	81.1917	294.70	49.89
2	9.093	81.5488	246.18	50.11

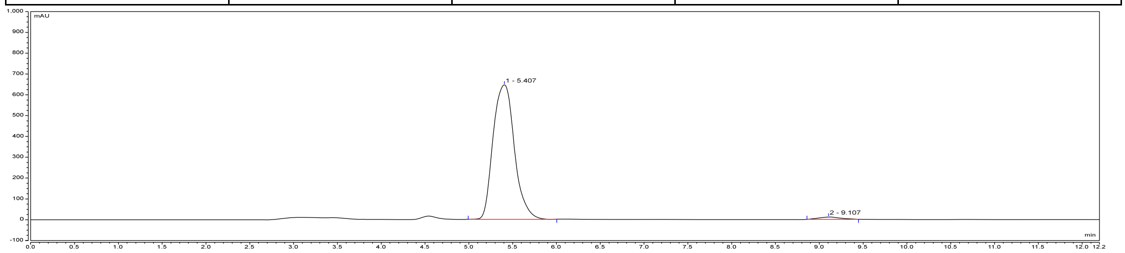

Entry	Retention Time	Area	Height	%Area
1	5.407	182.1385	645.86	98.33
2	9.107	3.0919	11.06	1.67

2-((4*S*,5*S*)-5-((*R*)-isopropyl(phenyl)phosphaneyl)-2,2-dimethyl-1,3-dioxolan-4-yl)pyridine-borane (55):

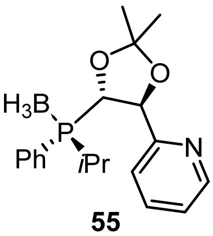

55

colorless oily liquid; Mp 178.5-179.0 °C; 27.8 mg, 81% yield, 95% ee; $[\alpha]_D^{22}$ +28.1 (*c* 1.0, $CHCl_3$); ^1H NMR (600 MHz, $CDCl_3$) δ 8.47 (d, *J* = 4.3 Hz, 1H), 7.64 – 7.51 (m, 2H), 7.43 – 7.39 (m, 1H), 7.30 (td, *J* = 7.4, 1.2 Hz, 1H), 7.19 (td, *J* = 7.8, 1.9 Hz, 2H), 7.08 (dd, *J* = 7.0, 5.2 Hz, 1H), 7.04 (d, *J* = 7.7 Hz, 1H), 5.25 (t, *J* = 9.0 Hz, 1H), 5.13 (d, *J* = 8.7 Hz, 1H), 2.57 – 2.47 (m, 1H), 1.60 (s, 3H), 1.53 (s, 3H), 1.24 (dd, *J* = 16.2, 7.0 Hz, 3H), 1.04 (dd, *J* = 15.4, 7.1 Hz, 3H), 0.87 – 0.41 (br, 3H); ^{13}C NMR (151 MHz, $CDCl_3$) δ 155.6, 149.2, 135.3, 133.4 (d, *J* = 7.8 Hz), 131.4 (d, *J* = 2.0 Hz), 128.3 (d, *J* = 9.0 Hz), 125.5, 125.2 (d, *J* = 4.0 Hz), 123.9 (d, *J* = 131.2 Hz), 112.4 (d, *J* = 6.8 Hz), 80.5 (d, *J* = 7.5 Hz), 76.2 (d, *J* = 45.9 Hz), 32.3, 27.1, 26.8, 26.5, 24.2, 23.9, 23.6, 17.1 (d, *J* = 2.8 Hz); ^{31}P NMR (243 MHz, $CDCl_3$) δ 25.2 (d, *J* = 65.0 Hz); HRMS (ESI) m/z 344.1945 (M + H^+), calc. for $C_{23}H_{27}NOPS$ 344.1946.

The ee was determined by HPLC analysis: CHIRALPAK OD-H (4.6 mm i.d. x 250 mm); Hexane/2-propanol = 95/5; flow rate 1.0 mL/min; 25 °C; 254 nm; retention time: 5.4 min (minor) and 7.1 min (major).

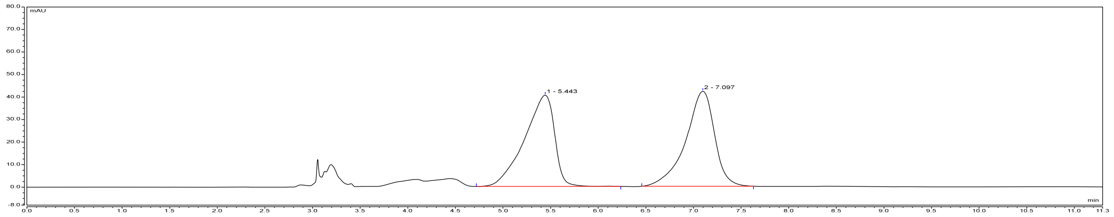

Entry	Retention Time	Area	Height	%Area
1	5.443	14.8101	40.53	49.98
2	7.097	14.8217	42.20	50.02

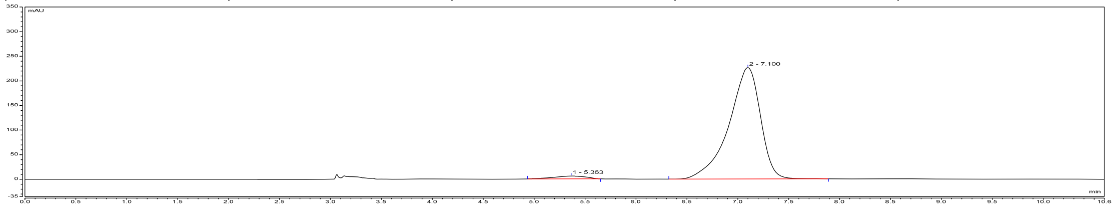

Entry	Retention Time	Area	Height	%Area
1	5.363	2.0839	5.70	2.50
2	7.100	81.1458	226.13	97.50

bis(3,5-bis(trifluoromethyl)benzyl) (*S*,*E*)-2-(1,3-diphenylallyl)malonate (58):

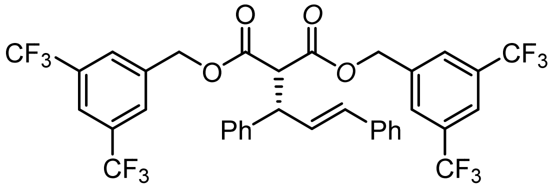

58

white solid; Mp 89.2-90.6 °C; 69.6 mg, 93% yield, 73% ee; $[\alpha]_D^{22}$ -4.3 (*c* 1.0, $CHCl_3$); ^1H NMR (400 MHz, $CDCl_3$) δ 7.88 – 7.70 (m, 4H), 7.55 (s, 2H), 7.25 – 7.11 (m, 10H), 6.41 (d, *J* = 15.8 Hz, 1H), 6.27 (dd, *J* = 15.7, 8.5 Hz, 1H), 5.31 – 5.15 (m, 2H), 5.01 (s, 2H), 4.28 (dd, *J* = 10.9, 8.6 Hz, 1H), 4.11 (d, *J* = 11.0 Hz, 1H); ^{13}C NMR (101 MHz, $CDCl_3$) δ 167.2, 166.9, 139.5, 137.6, 137.4, 136.3, 132.4, 132.4 (dd, *J* = 33.5, 7.8 Hz), 131.7 (dd, *J* = 33.6, 7.7 Hz), 128.9, 128.7, 128.4 (d, *J* = 18.8 Hz), 127.8, 127.8 (d, *J* = 32.0 Hz), 126.3, 124.5 (d, *J* = 2.8 Hz), 122.6 – 122.4 (m), 121.8 (d, *J* = 2.9 Hz), 65.8, 65.6, 57.6, 49.7; ^{19}F NMR (376 MHz, $CDCl_3$) δ -62.9; HRMS (ESI) m/z 749.1556 (M + H^+), calc. for $C_{36}H_{25}F_{12}O_4$ 749.1552.

The ee was determined by HPLC analysis: CHIRALPAK OD-H (4.6 mm i.d. x 250 mm); Hexane/2-propanol = 80/20; flow rate 1.0 mL/min; 25 °C; 254 nm; retention time: 4.5 min (minor) and 5.5 min (major).

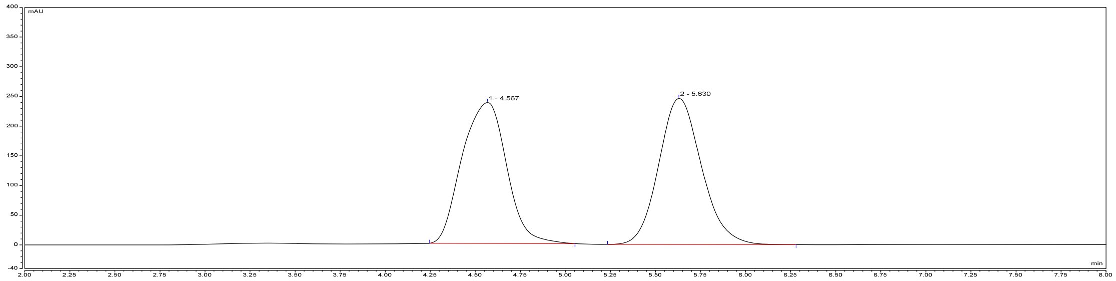

Entry	Retention Time	Area	Height	%Area
1	4.567	67.8384	237.25	50.04
2	5.630	67.7169	246.10	49.96

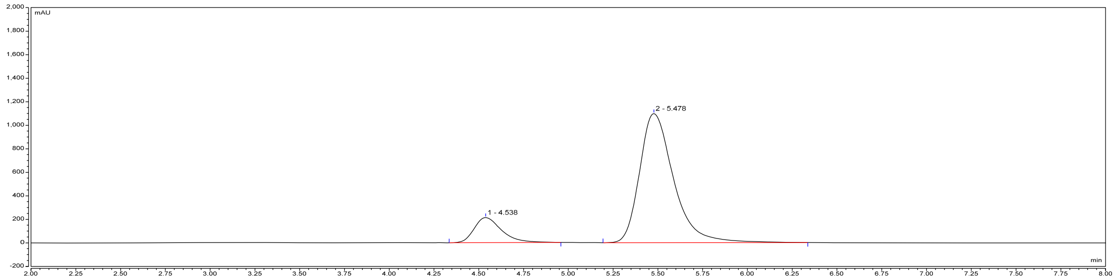

Entry	Retention Time	Area	Height	%Area
1	4.538	37.6076	213.43	13.48
2	5.478	241.3067	1098.14	86.52

bis(3,5-bis(trifluoromethyl)benzyl) (*R*,*E*)-2-(1,3-diphenylallyl)malonate (*ent*-58):

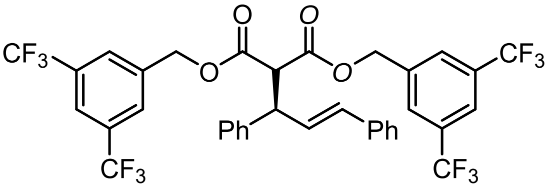

ent-**58**

white solid; Mp 89.2-90.6 °C; 68.1 mg, 91% yield, 81% ee; $[\alpha]_D^{22}$ +4.8 (*c* 1.0, $CHCl_3$). The ee was determined by HPLC analysis: CHIRALPAK OD-H (4.6 mm i.d. x 250 mm); Hexane/2-propanol = 80/20; flow rate 1.0 mL/min; 25 °C; 254 nm; retention time: 4.6 min (major) and 5.6 min (minor).

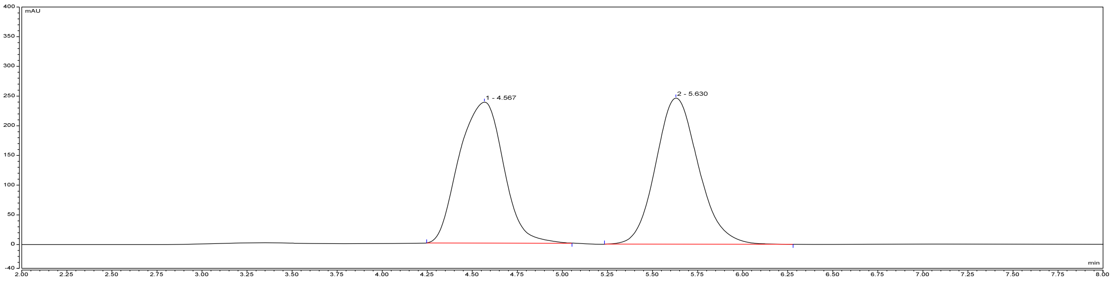

Entry	Retention Time	Area	Height	%Area
1	4.567	67.8384	237.25	50.04
2	5.630	67.7169	246.10	49.96

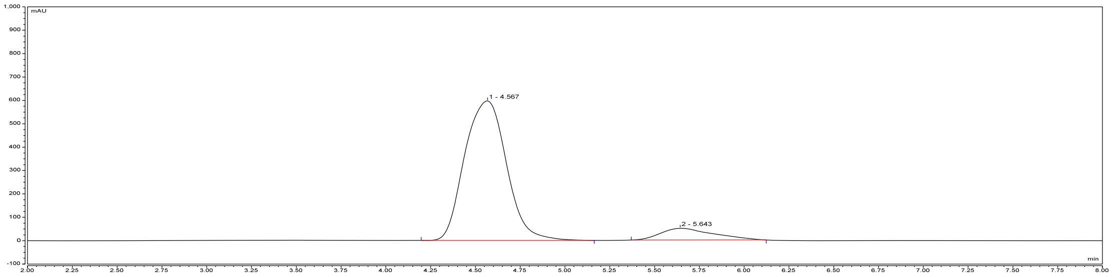

Entry	Retention Time	Area	Height	%Area
1	4.567	161.4837	596.37	90.44
2	5.643	17.0711	50.33	9.56

(*R*)-2-(2-(isopropyl(phenyl)phosphaneyl)ethyl)pyridine (59):

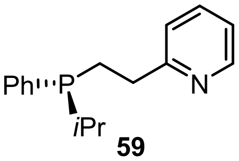

59

colorless oily liquid; 93% ee; $[\alpha]_D^{22}$ +24.3 (c 1.0, $CHCl_3$); ^{1}H NMR (600 MHz, $CDCl_3$) δ 8.50 (d, J = 4.3 Hz, 1H), 7.57 – 7.51 (m, 3H), 7.38 – 7.31 (m, 3H), 7.09 – 7.03 (m, 2H), 2.89 – 2.81 (m, 1H), 2.77 – 2.70 (m, 1H), 2.24 – 2.14 (m, 2H), 1.97 – 1.88 (m, 1H), 1.12 (dd, *J* = 14.6, 7.0 Hz, 3H), 0.88 (dd, *J* = 14.1, 7.0 Hz, 3H); ^{13}C NMR (151 MHz, $CDCl_3$) δ 162.2 (d, *J* = 12.3 Hz), 149.3, 137.0 (d, *J* = 15.7 Hz), 136.4, 133.5 (d, *J* = 18.9 Hz), 129.1, 128.4 (d, *J* = 6.9 Hz), 122.8, 121.2, 34.9 (d, *J* =

16.0 Hz), 27.3 (d, J = 8.5 Hz), 25.6 (d, J = 13.8 Hz), 19.7 (d, J = 6.7 Hz), 19.6 (d, J = 9.4 Hz); ^{31}P NMR (243 MHz, $CDCl_3$) δ -5.9 (d, J = 5.0 Hz); HRMS (ESI) m/z 258.1406 (M + H^+), calc. for $C_{16}H_{21}NP$ 258.1402.

The ee was determined by HPLC analysis: CHIRALPAK OD-H (4.6 mm i.d. x 250 mm); Hexane/2-propanol = 98/2; flow rate 1.0 mL/min; 25 °C; 210 nm; retention time: 7.4 min (major) and 11.0 (minor) min.

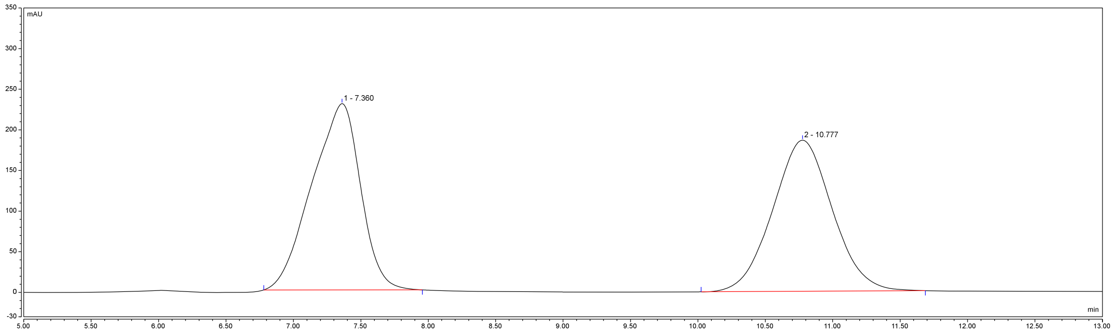

Entry	Retention Time	Area	Height	%Area
1	7.360	96.2015	229.26	50.29
2	10.777	95.1102	185.91	49.71

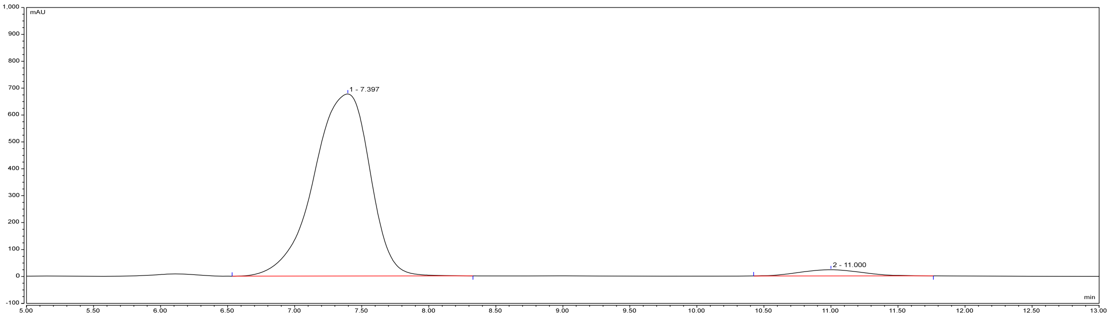

Entry	Retention Time	Area	Height	%Area
1	7.397	328.8305	676.96	96.41
2	11.000	12.2619	22.80	3.59

(*R*,*E*)-isopropyl(phenyl)(2-(pyridin-2-yl)vinyl-1,2-d2)phosphine oxide (D-**3**)**:**

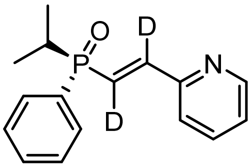

D-**3**

colorless oily liquid; 25.1 mg, 92% yield, 95% ee; $[\alpha]_D^{22}$ -18.5 (*c* 1.0, $CHCl_3$); ^{1}H NMR (400 MHz, $CDCl_3$) δ 8.57 (dd, J = 4.7, 0.8 Hz, 1H), 7.73 (ddd, J = 10.9, 7.8, 1.6 Hz, 2H), 7.65 (td, J = 7.7, 1.8 Hz, 1H), 7.47 – 7.38 (m, 3H), 7.28 (d, J = 7.7 Hz, 1H), 7.22 – 7.16 (m, 1H), 2.20 – 2.07 (m, 1H), 1.18 (dd, J = 16.7, 7.2 Hz, 3H), 1.07 (dd, J = 16.7, 7.2 Hz, 3H); HRMS (ESI) m/z 274.1324 (M + H^+), calc. for $C_{16}H_{19}NOP$ 274.1322.

The ee was determined by HPLC analysis: CHIRALPAK IE (4.6 mm i.d. x 250 mm); Hexane/2-propanol = 70/30; flow rate 1.0 mL/min; 25 °C; 254 nm; retention time: 28.5 min (minor) and 30.8 min (major).

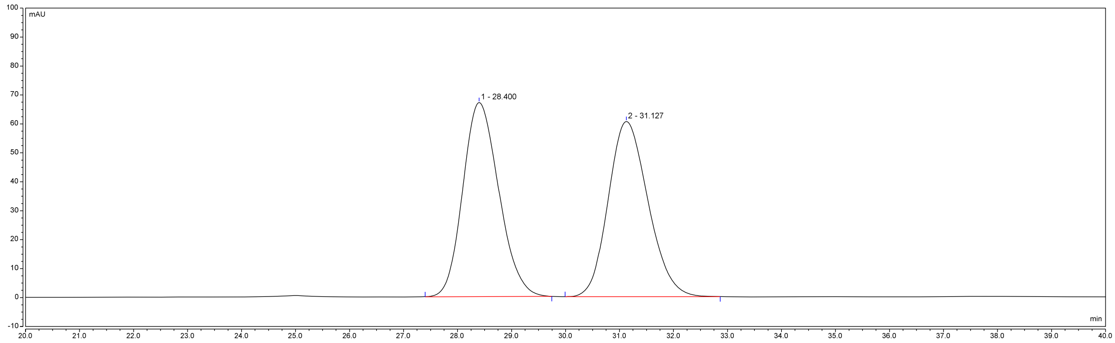

Entry	Retention Time	Area	Height	%Area
1	28.400	51.6754	67.03	49.99
2	31.127	51.6874	60.51	50.01

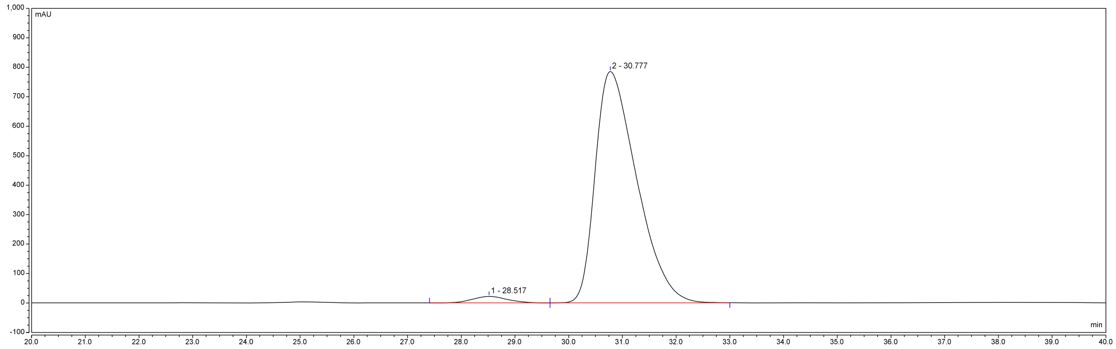

Entry	Retention Time	Area	Height	%Area
1	28.517	16.2837	21.69	2.27
2	30.777	701.8910	784.83	97.73

9. Copies of NMR spectra

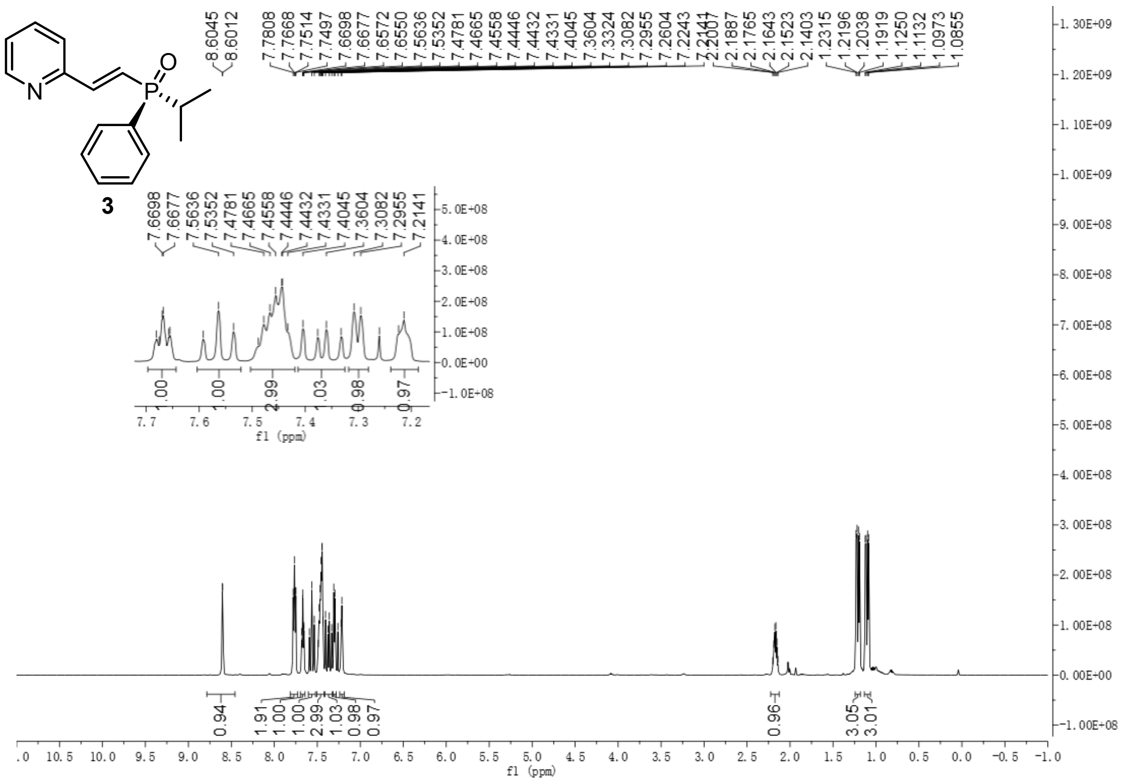

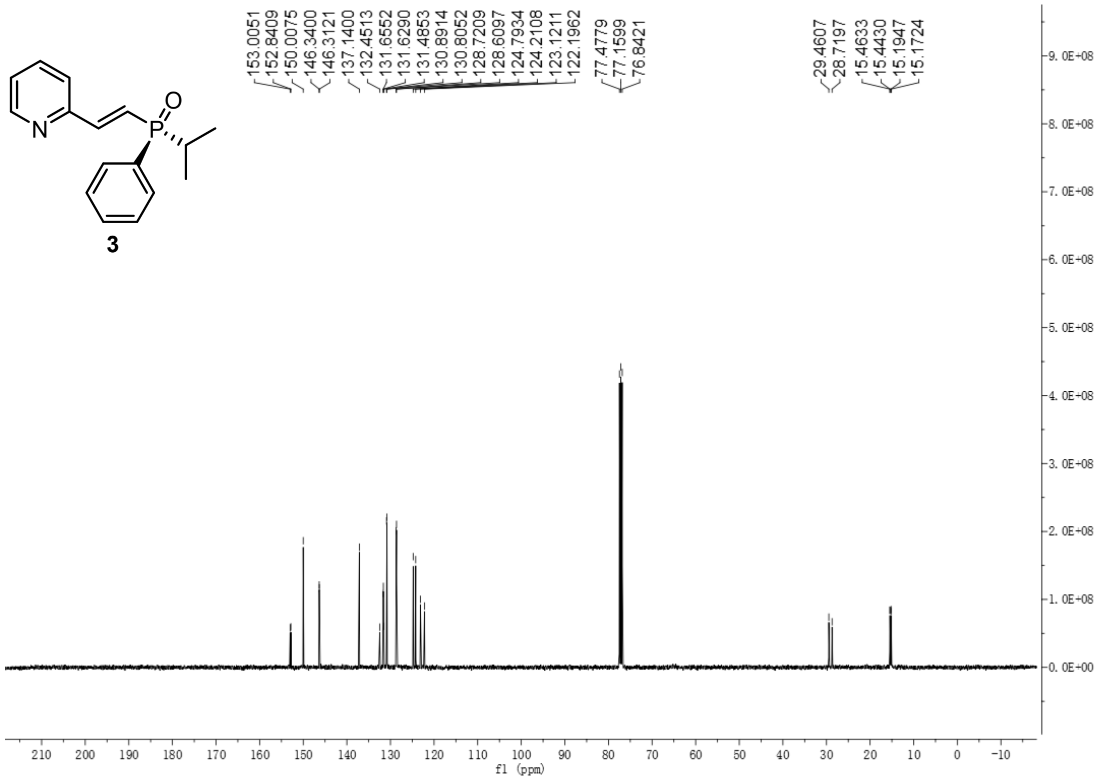

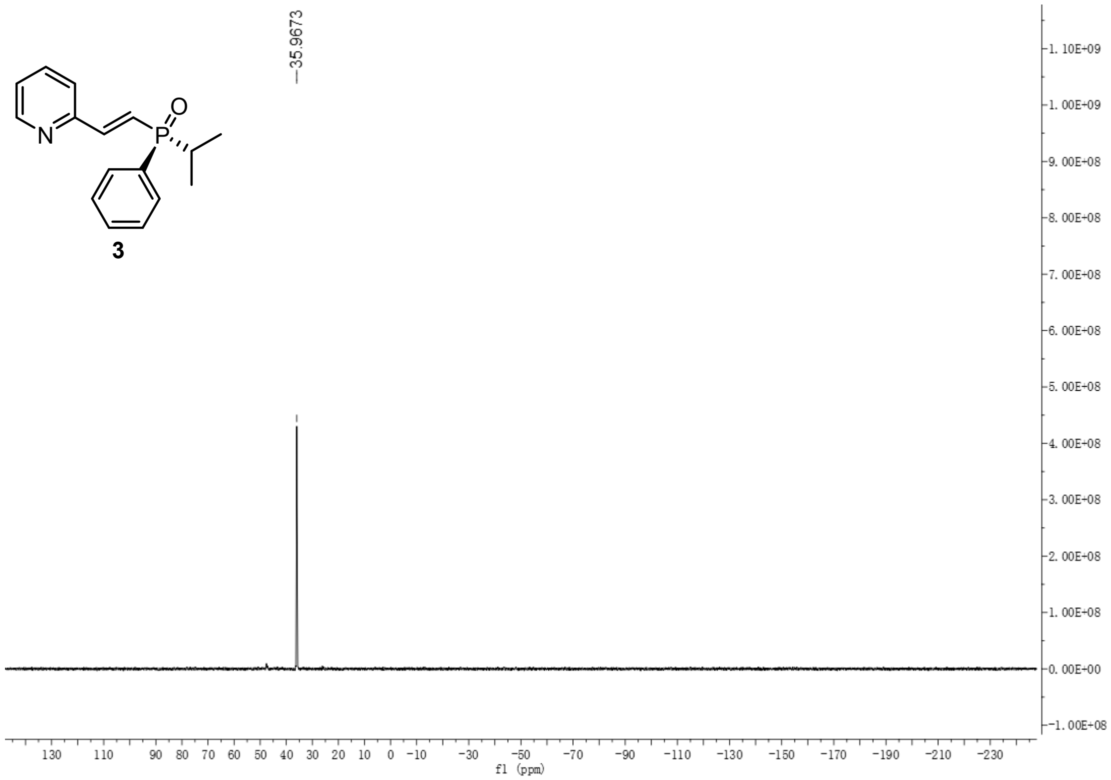

3

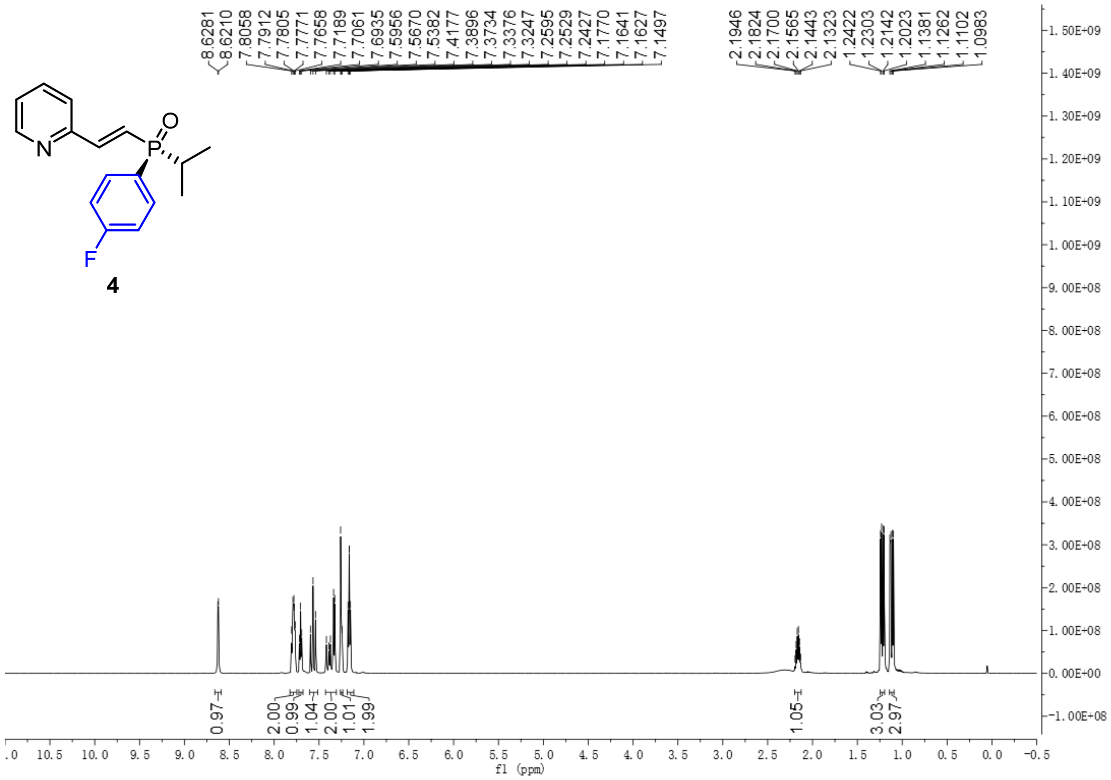

4

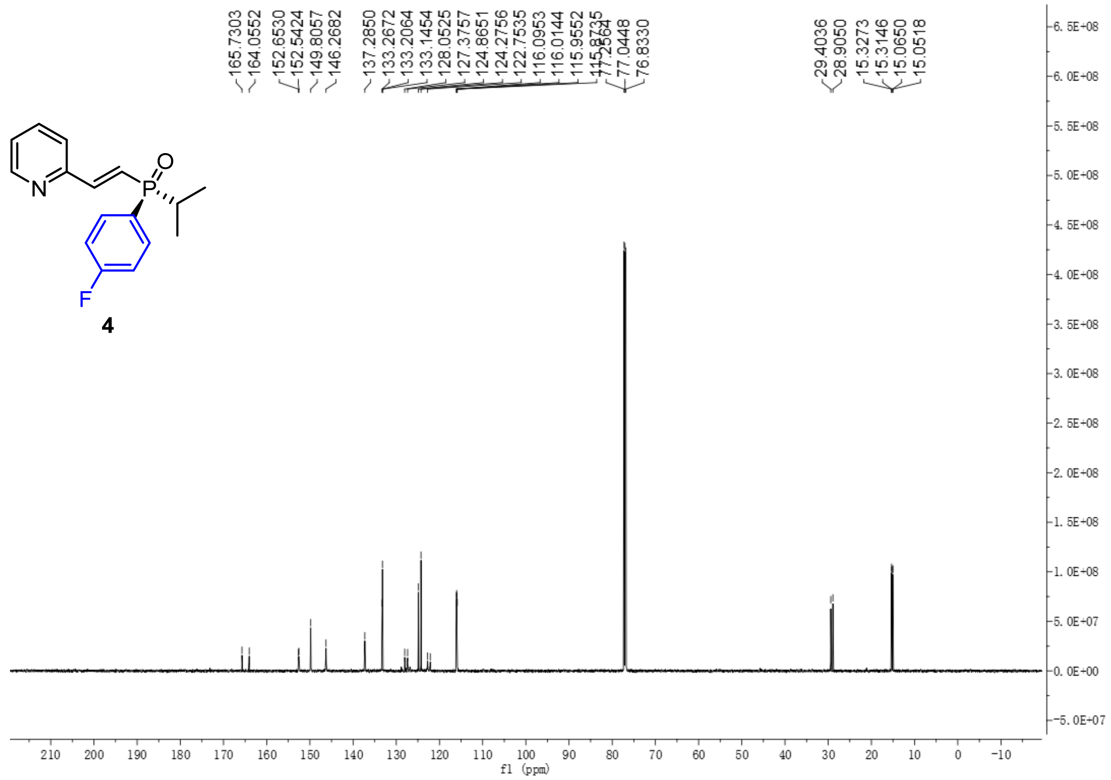

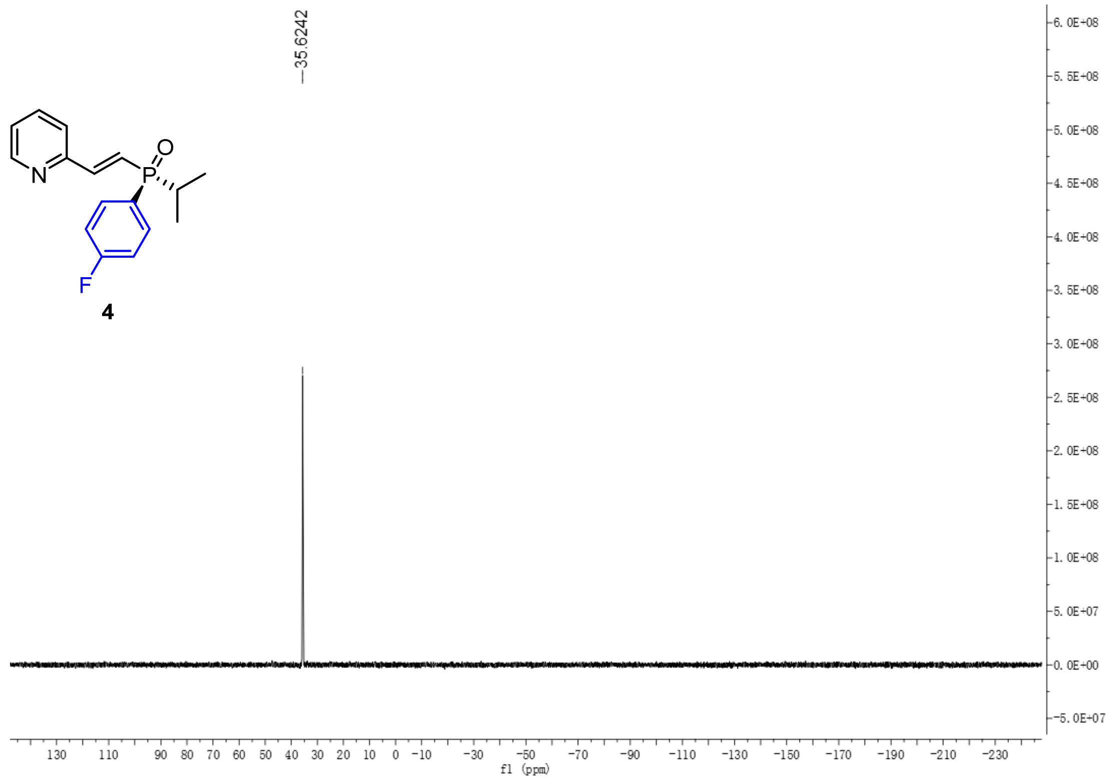

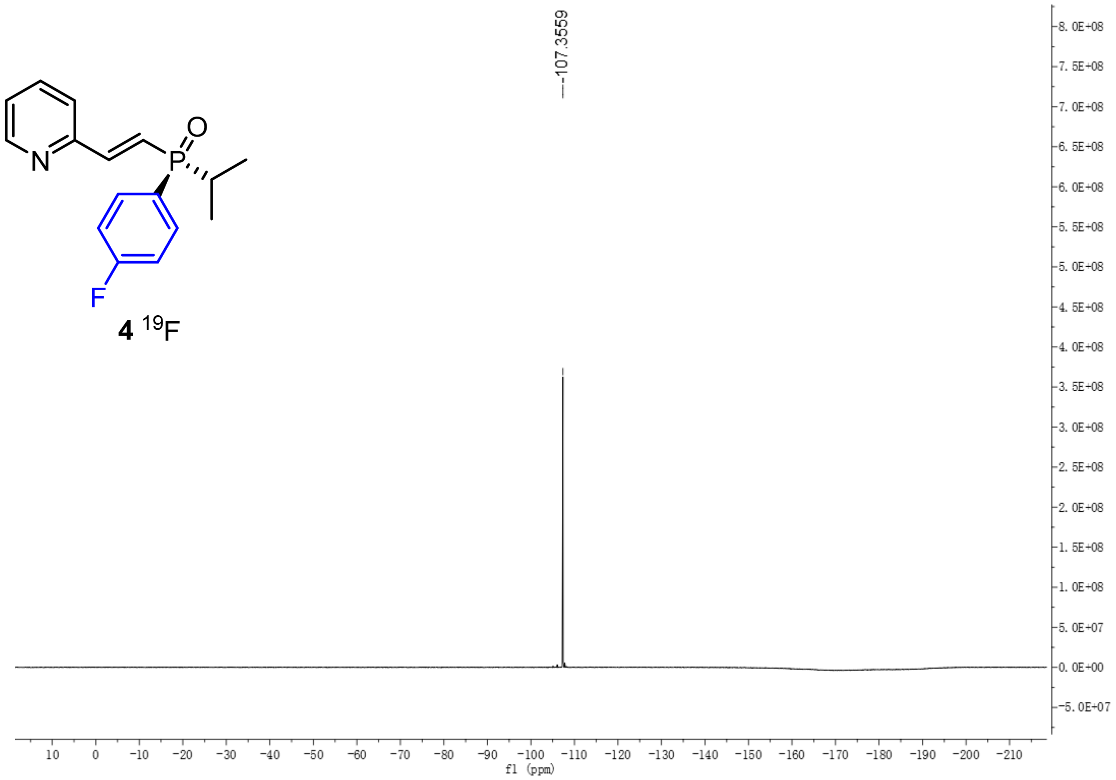
-107.3559
4 19F
fl (ppm)

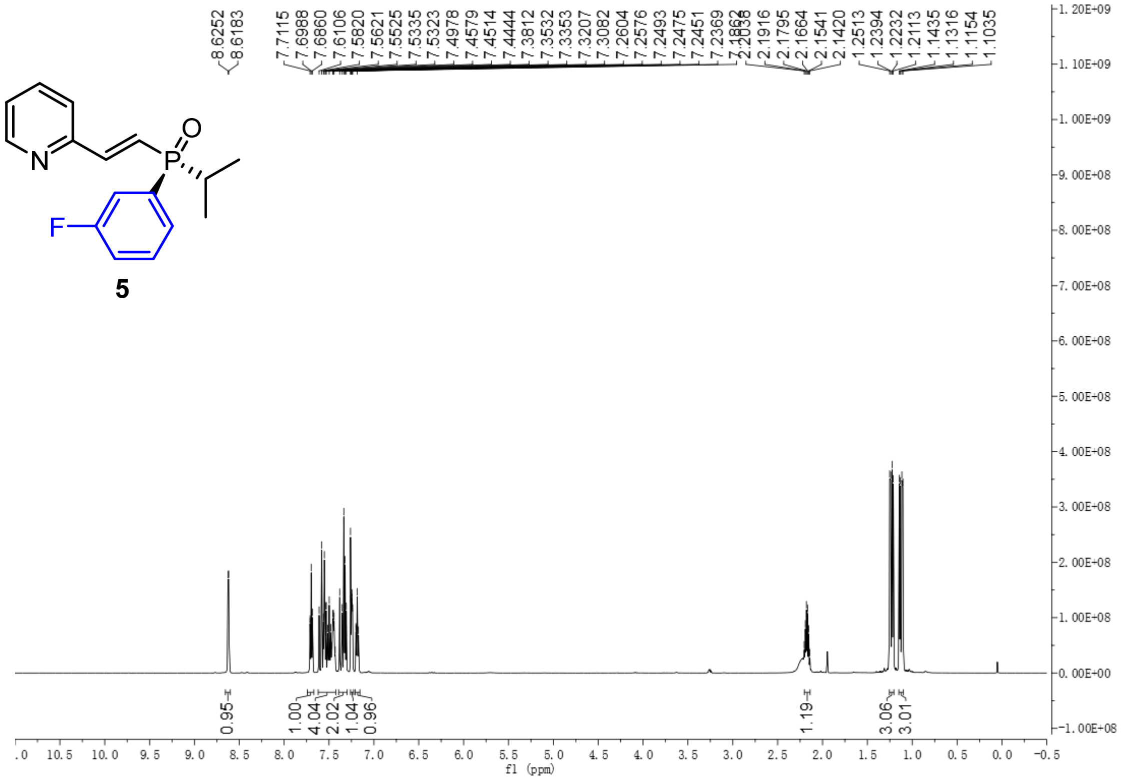
8.6252
8.6183
7.7115
7.6988
7.6860
7.6106
7.5820
7.5621
7.5525
7.5335
7.5323
7.4978
7.4579
7.4514
7.4444
7.3812
7.3532
7.3353
7.3207
7.3082
7.2604
7.2576
7.2493
7.2475
7.2451
7.2369
2.1916
2.1795
2.1664
2.1541
2.1420
1.2513
1.2394
1.2232
1.2113
1.1435
1.1316
1.1154
1.1035
5
0.95
1.00
4.04
2.02
1.04
0.96
1.19
3.06
3.01
fl (ppm)

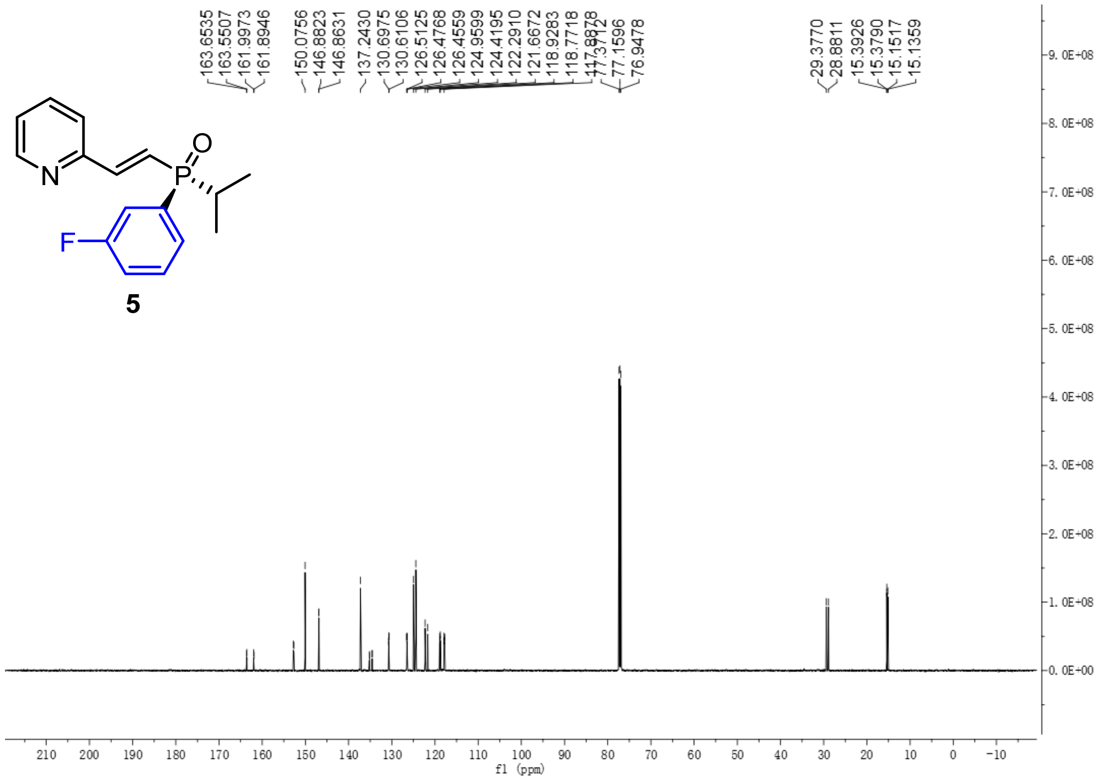
163.6535
163.5507
161.9973
161.8946
150.0756
146.8823
146.8631
137.2430
130.6975
130.6106
126.5125
126.4768
126.4559
124.9599
124.4195
122.2910
121.6672
118.9283
118.7718
77.1596
76.9478
29.3770
28.8811
15.3926
15.3790
15.1517
15.1359
5
fl (ppm)

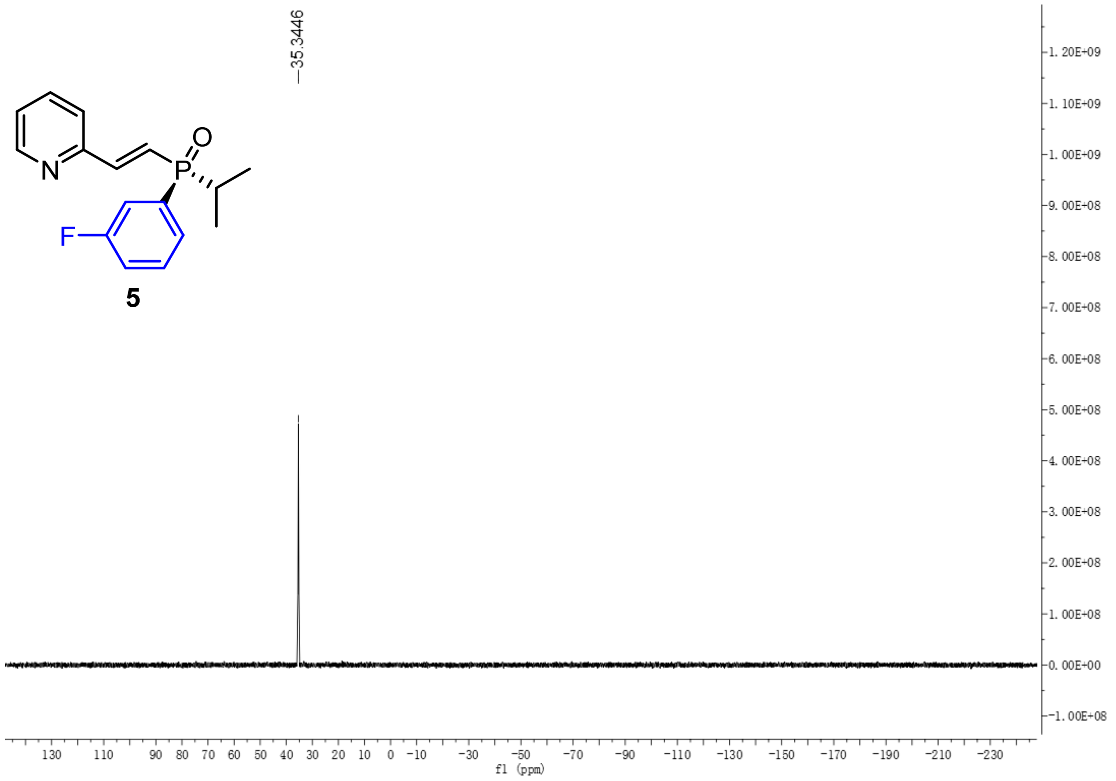
35.3446
5
fl (ppm)

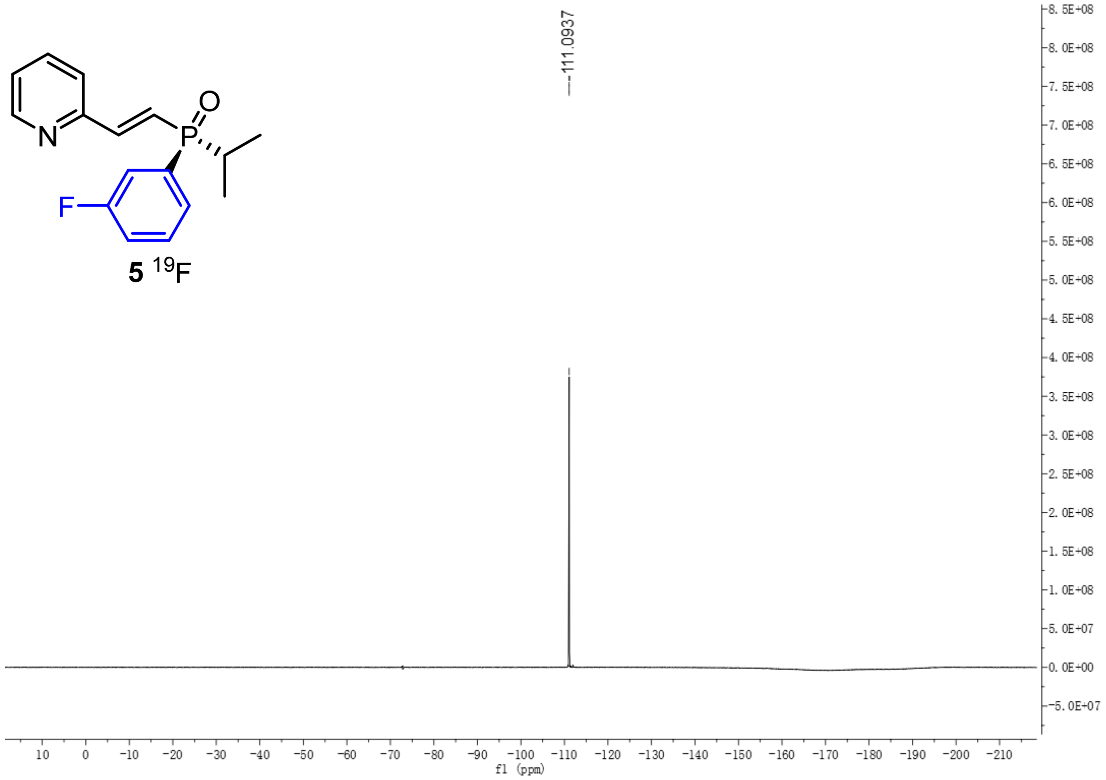

5 19F
-111.0937
f1 (ppm)

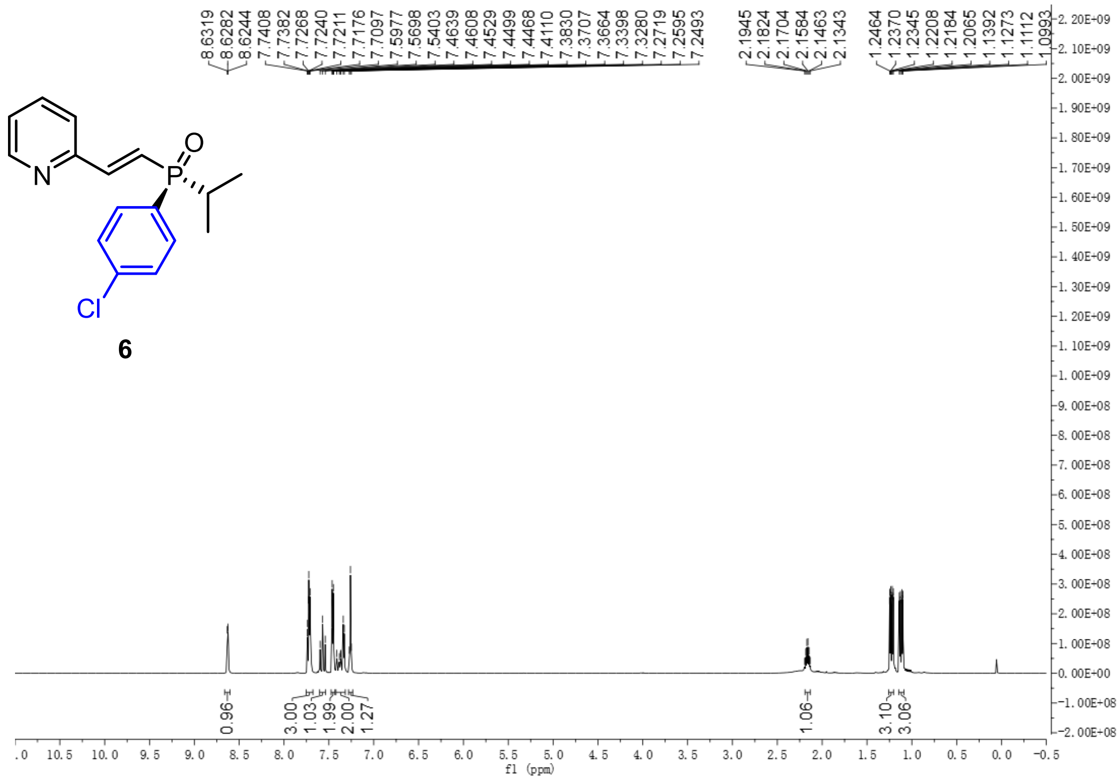

6
f1 (ppm)

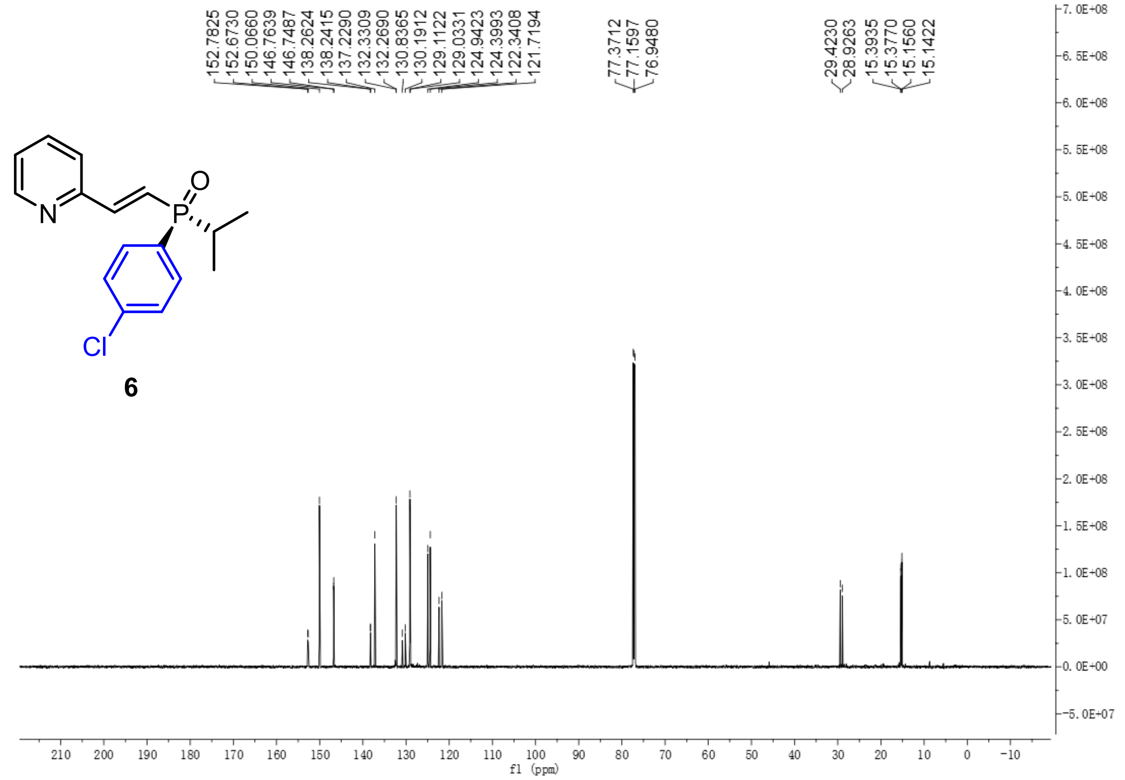

152.7825
152.6730
150.0660
146.7639
146.7487
138.2624
138.2415
137.2290
132.3309
132.2690
130.8365
130.1912
129.1122
129.0331
124.9423
124.3993
122.3408
121.7194
77.3712
77.1597
76.9480
29.4230
28.9263
15.3935
15.3770
15.1560
15.1422
N
O
P
Cl
6
f1 (ppm)

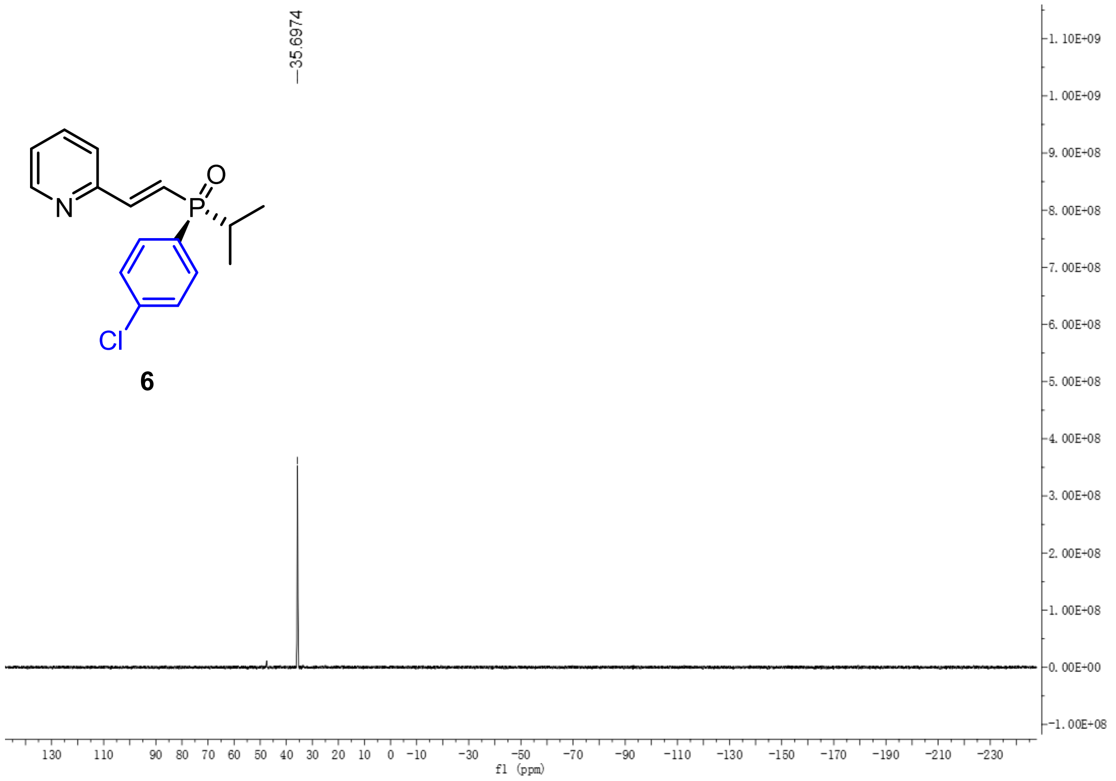

35.6974
N
O
P
Cl
6
f1 (ppm)

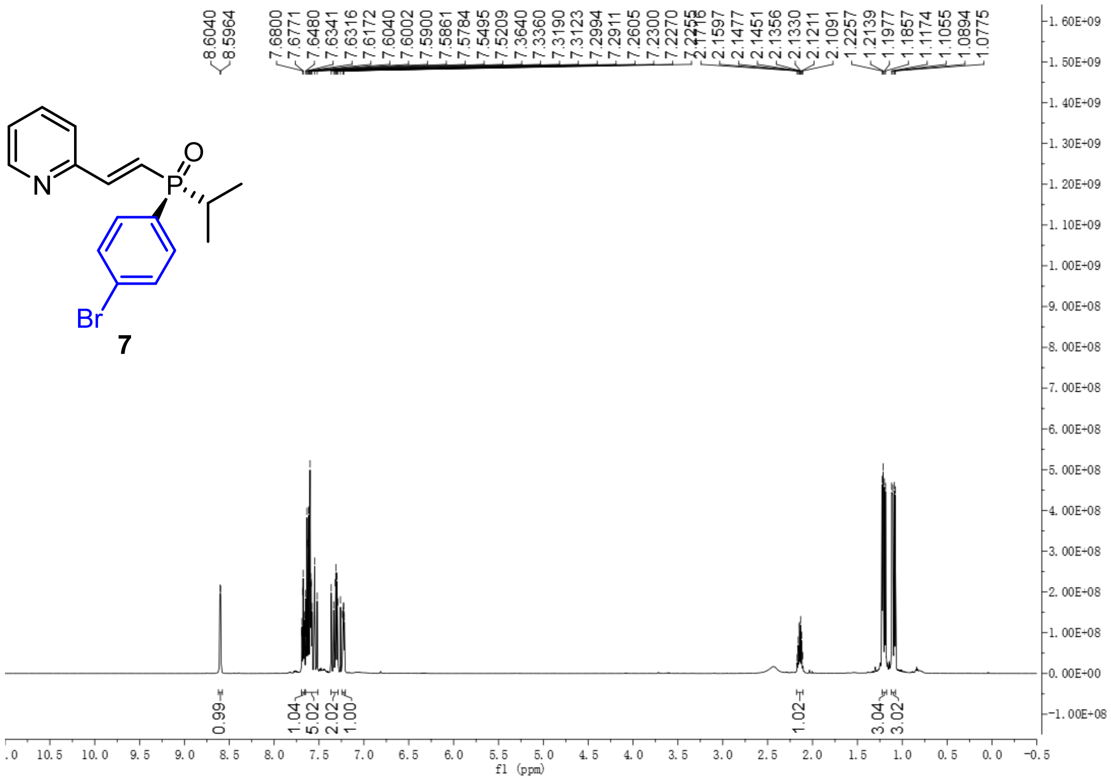
8.6040
8.5964
7.6800
7.6771
7.6480
7.6341
7.6316
7.6172
7.6040
7.6002
7.5900
7.5861
7.5784
7.5495
7.5209
7.3640
7.3360
7.3190
7.3123
7.2994
7.2911
7.2605
7.2300
7.2270
2.1597
2.1477
2.1451
2.1356
2.1330
2.1211
2.1091
1.2257
1.2139
1.1977
1.1857
1.1174
1.1055
1.0894
1.0775
O
P
N
Br
7
0.99
1.04
5.02
2.02
1.00
1.02
3.04
3.02
f1 (ppm)

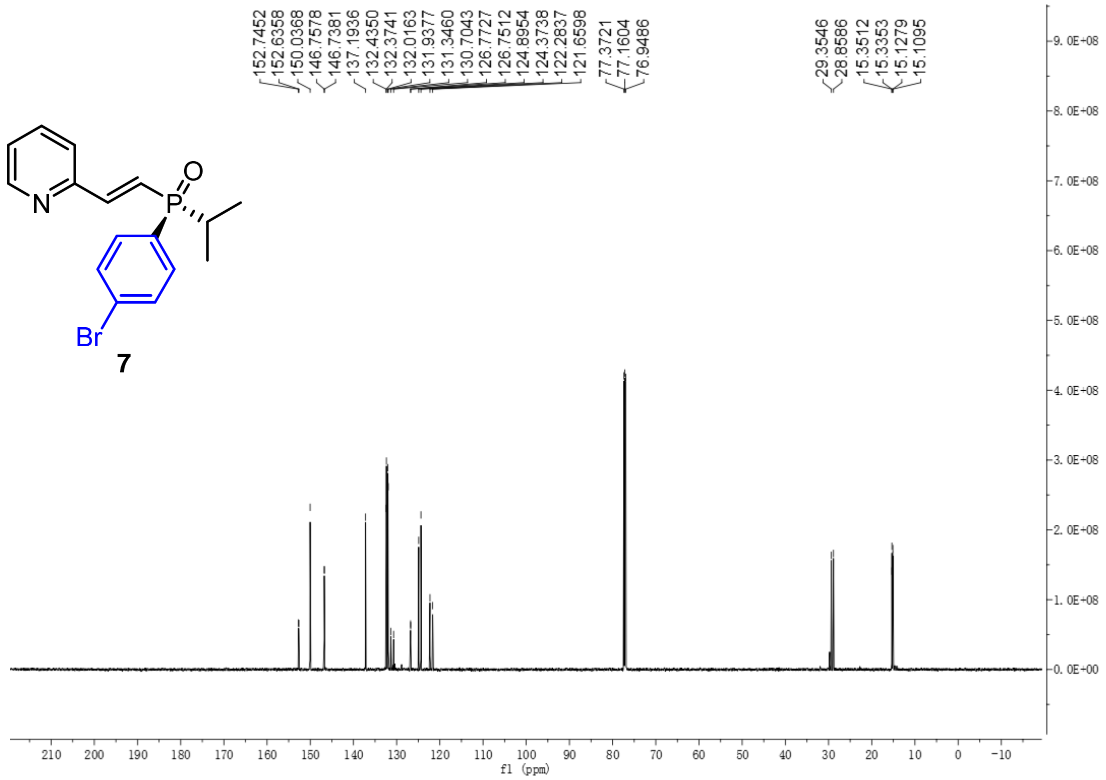
152.7452
152.6358
150.0368
146.7578
146.7381
137.1936
132.4350
132.3741
132.0163
131.9377
131.3460
130.7043
126.7727
126.7512
124.8954
124.3738
122.2837
121.6598
77.3721
77.1604
76.9486
29.3546
28.8586
15.3512
15.3353
15.1279
15.1095
O
P
N
Br
7
f1 (ppm)

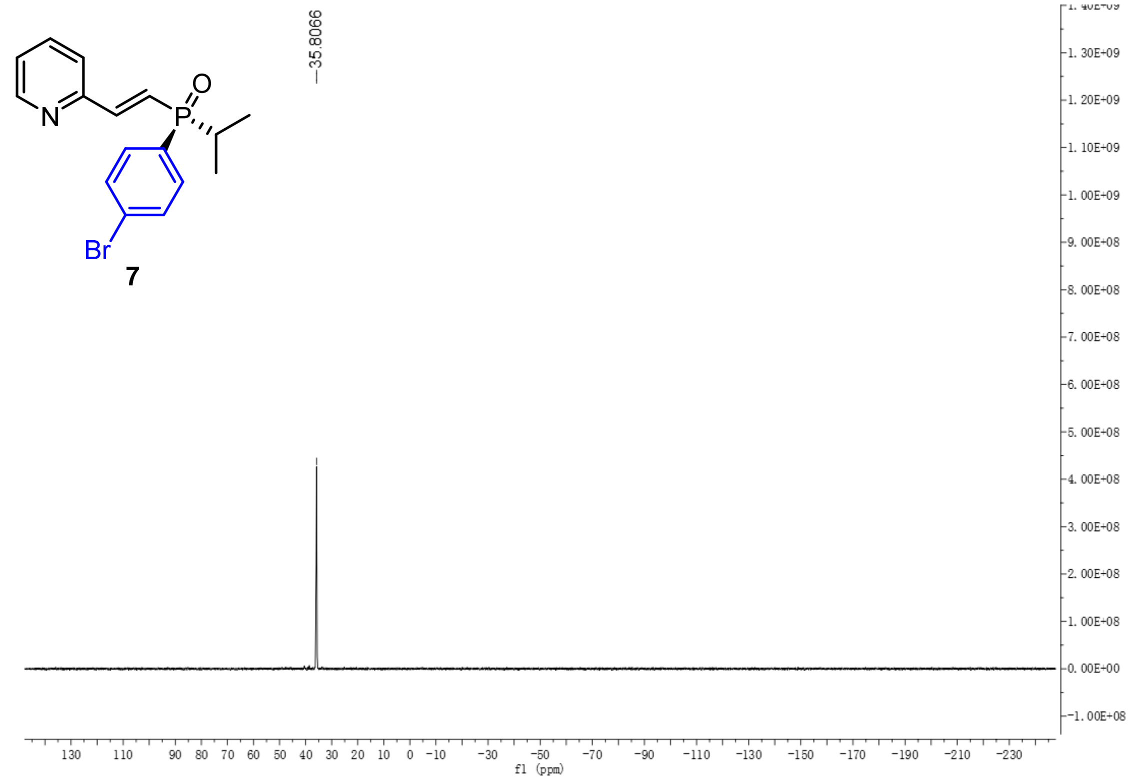
-35.8066
fl (ppm)

7

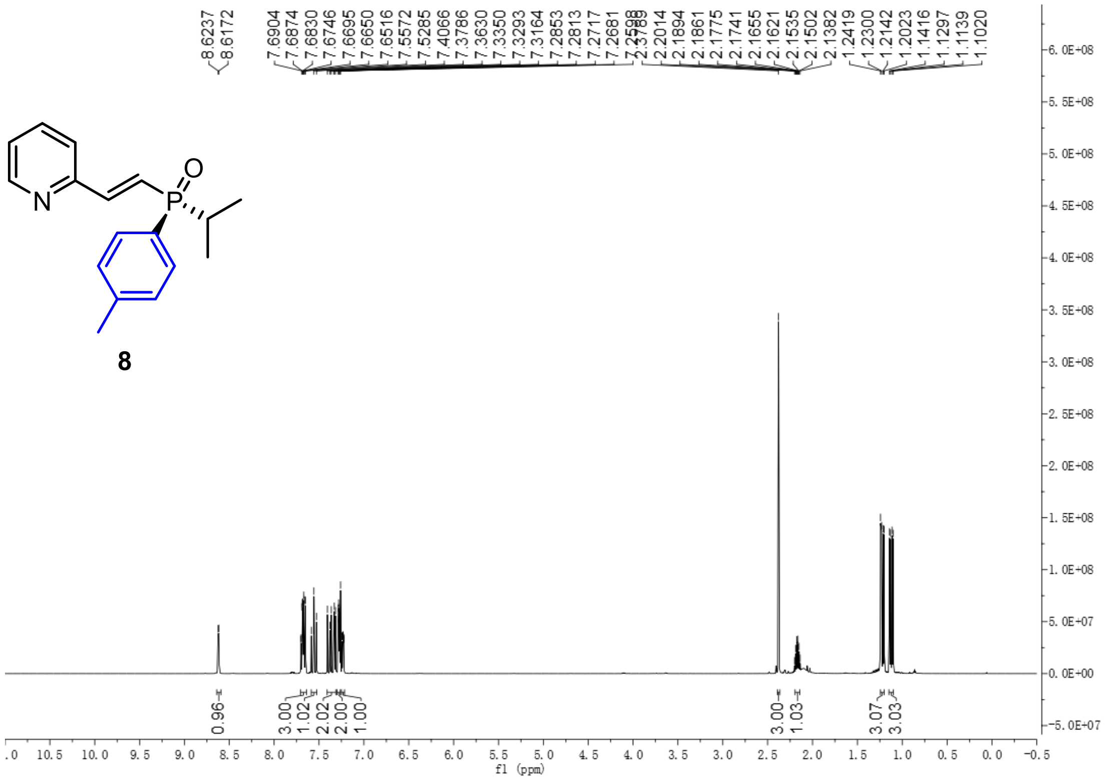
8.6237
8.6172
7.6904
7.6874
7.6830
7.6746
7.6695
7.6650
7.6516
7.5572
7.5285
7.4066
7.3786
7.3630
7.3350
7.3293
7.3164
7.2853
7.2813
7.2717
7.2681
2.2014
2.1894
2.1861
2.1775
2.1741
2.1655
2.1621
2.1535
2.1502
2.1382
1.2419
1.2300
1.2142
1.2023
1.1416
1.1297
1.1139
1.1020
0.96
3.00
1.02
2.02
2.00
1.00
3.00
1.03
3.07
3.03
fl (ppm)

8

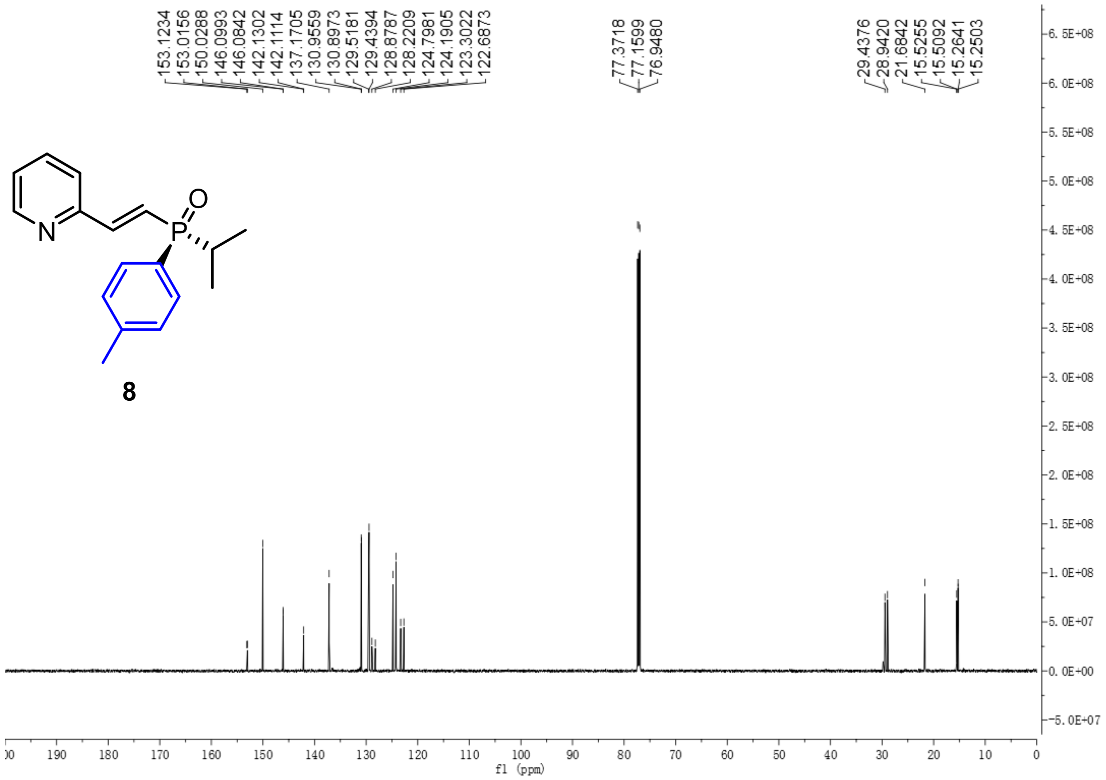

153.1234
153.0156
150.0288
146.0993
146.0842
142.1302
142.1114
137.1705
130.9559
130.8973
129.5181
129.4394
128.8787
128.2209
124.7981
124.1905
123.3022
122.6873
77.3718
77.1599
76.9480
29.4376
28.9420
21.6842
15.5255
15.5092
15.2641
15.2503
8
fl (ppm)

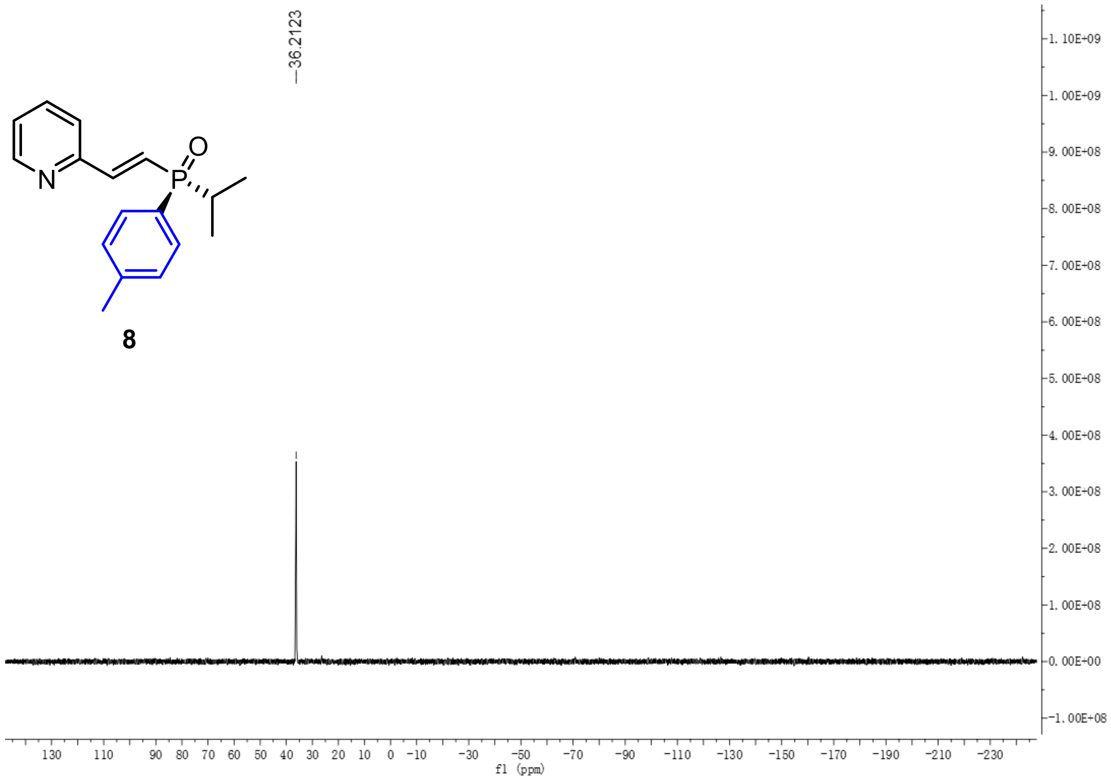

36.2123
8
fl (ppm)

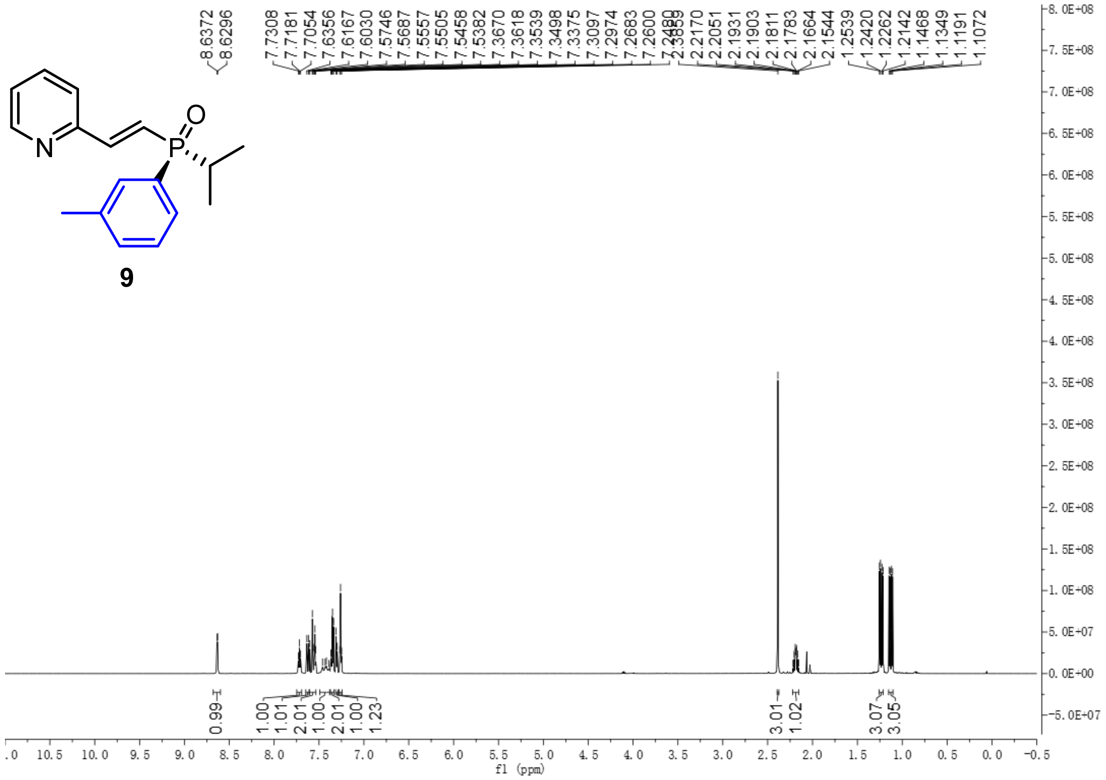

9
8.6372
8.6296
7.7308
7.7181
7.7054
7.6356
7.6167
7.6030
7.5746
7.5687
7.5557
7.5505
7.5458
7.5382
7.3670
7.3618
7.3539
7.3498
7.3375
7.3097
7.2974
7.2683
7.2600
2.2170
2.2051
2.1931
2.1903
2.1811
2.1783
2.1664
2.1544
1.2539
1.2420
1.2262
1.2142
1.1468
1.1349
1.1191
1.1072
0.99
1.00
1.01
2.01
1.00
2.01
1.00
1.23
3.01
1.02
3.07
3.05
f1 (ppm)

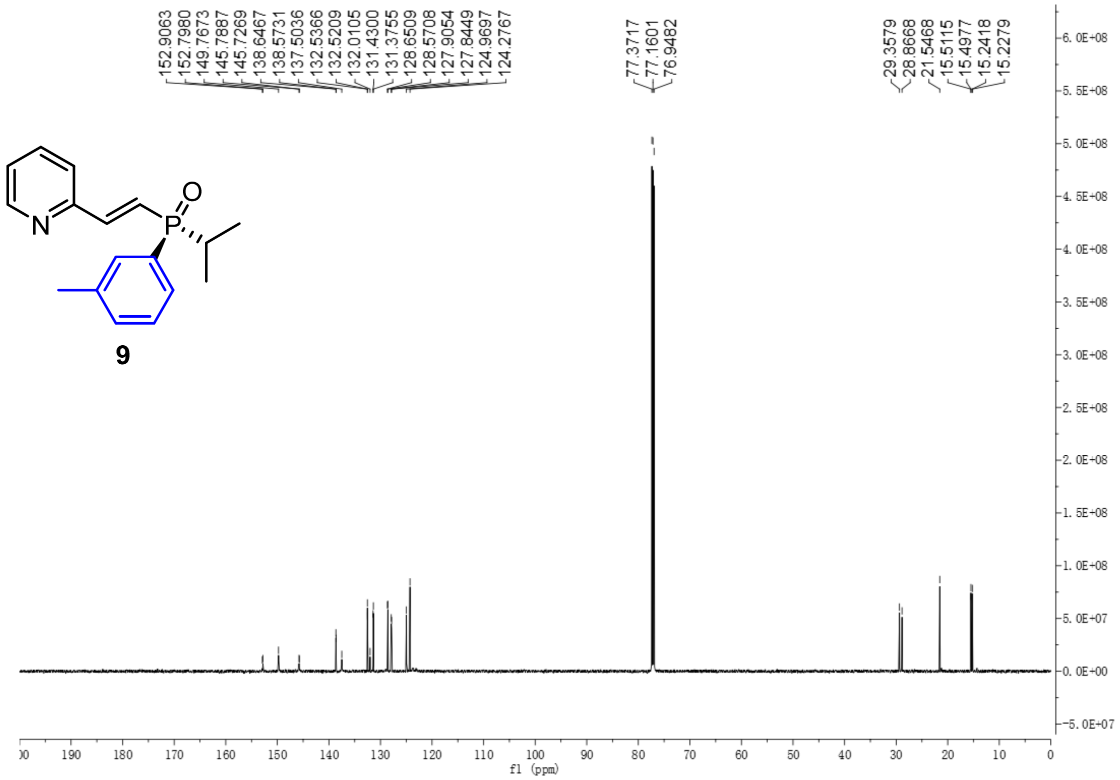

9
152.9063
152.7980
149.7673
145.7887
145.7269
138.6467
138.5731
137.5036
132.5366
132.5209
132.0105
131.4300
131.3755
128.6509
128.5708
127.9054
127.8449
124.9697
124.2767
77.3717
77.1601
76.9482
29.3579
28.8668
21.5468
15.5115
15.4977
15.2418
15.2279
f1 (ppm)

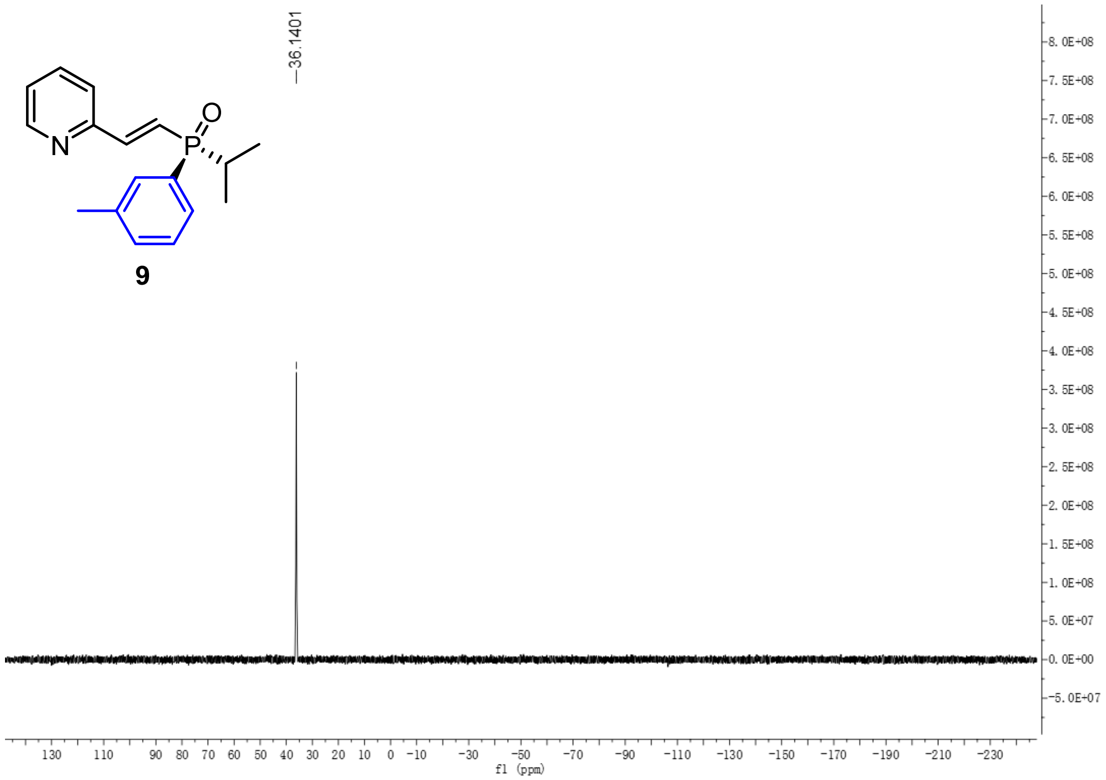

9

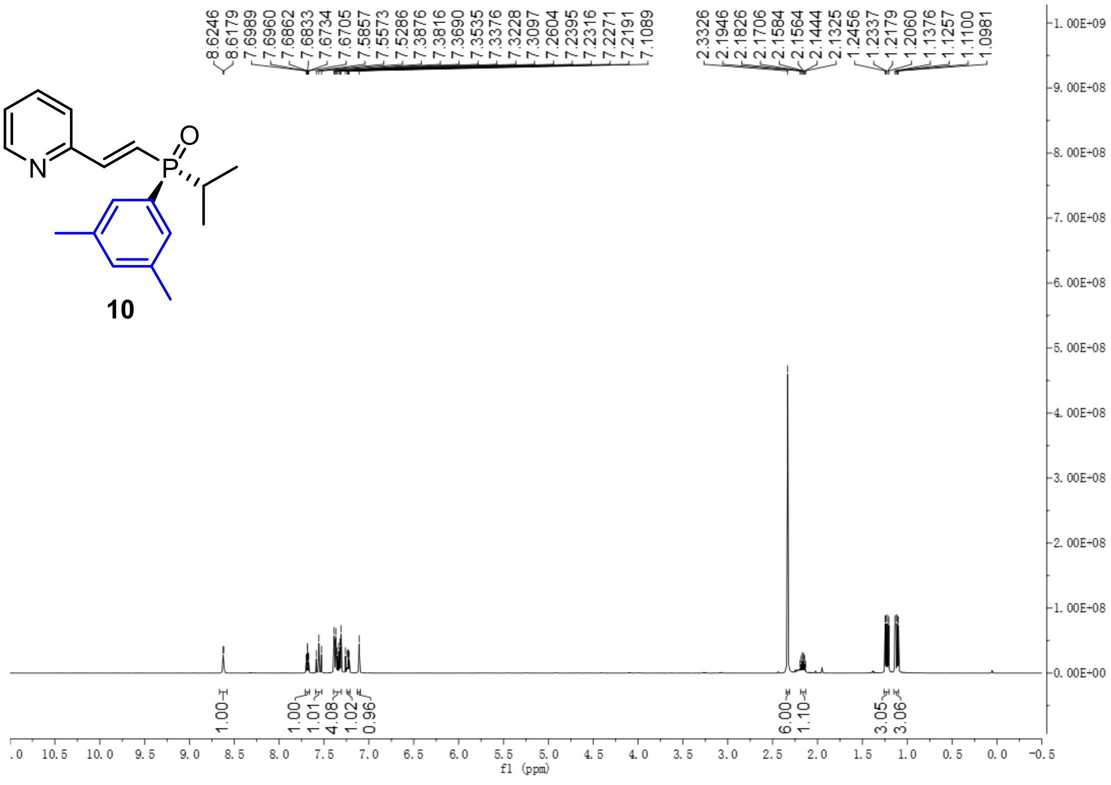

10

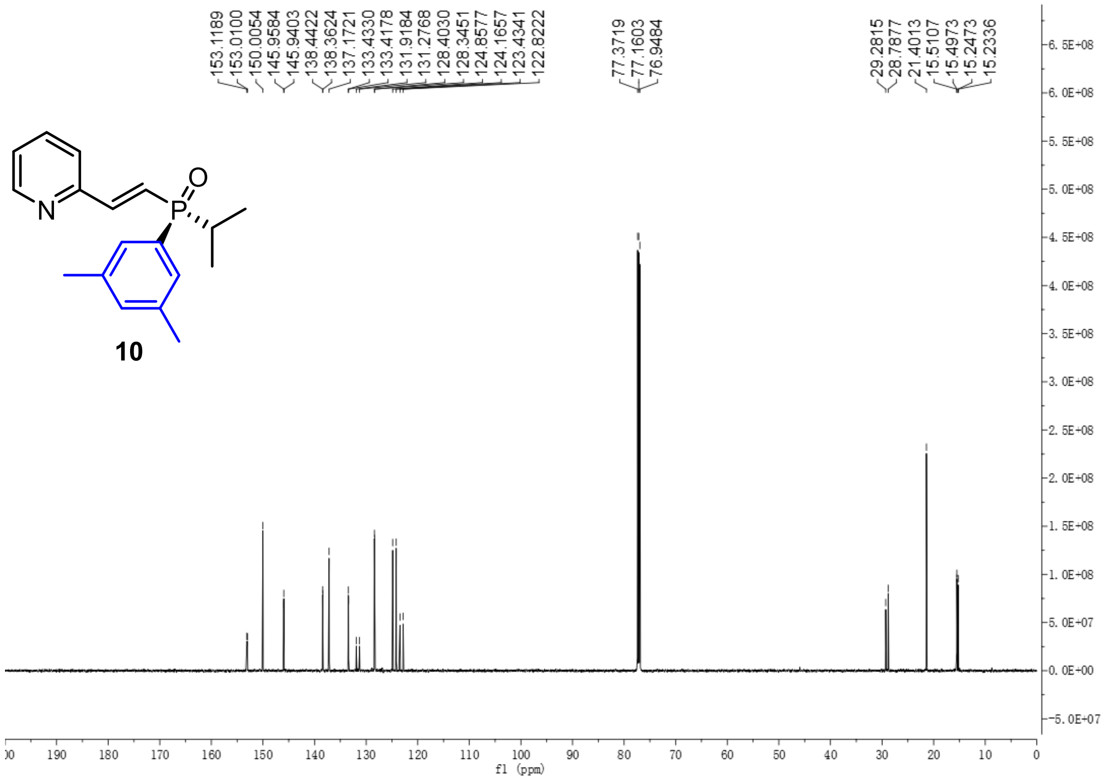
153.1189
153.0100
150.0054
145.9584
145.9403
138.4422
138.3624
137.1721
133.4330
133.4178
131.9184
131.2768
128.4030
128.3451
124.8577
124.1657
123.4341
122.8222
77.3719
77.1603
76.9484
29.2815
28.7877
21.4013
15.5107
15.4973
15.2473
15.2336
N
O
P
10
fl (ppm)

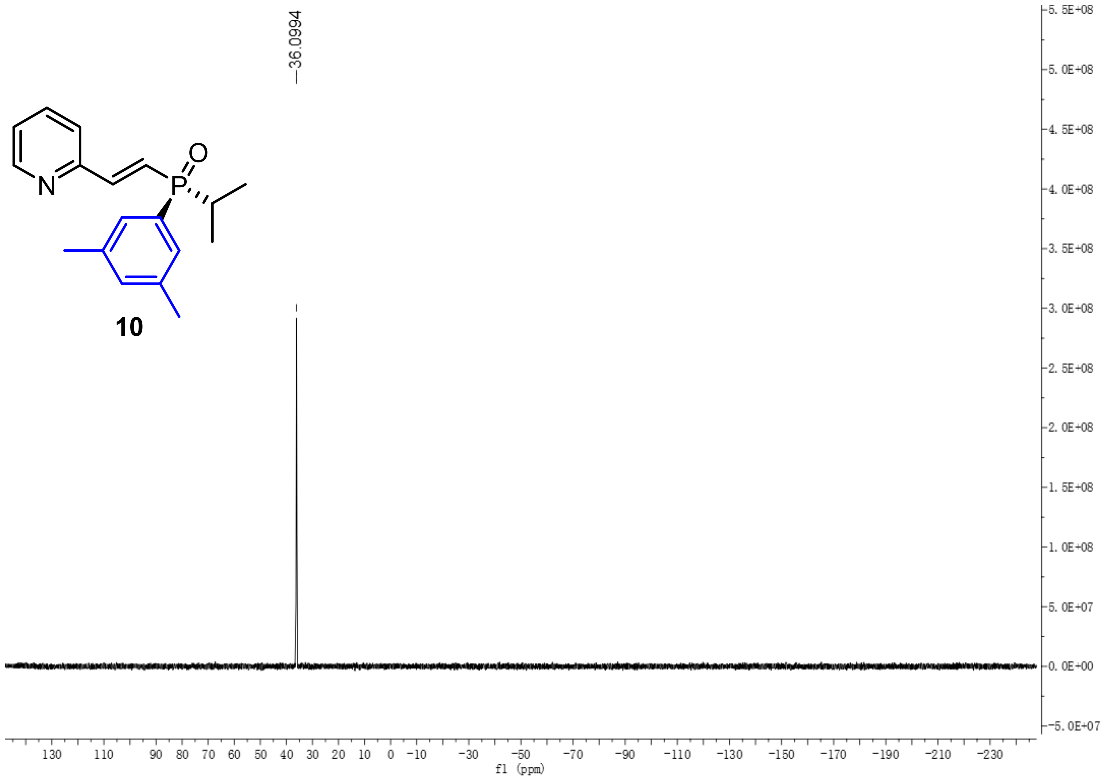
36.0994
N
O
P
10
fl (ppm)

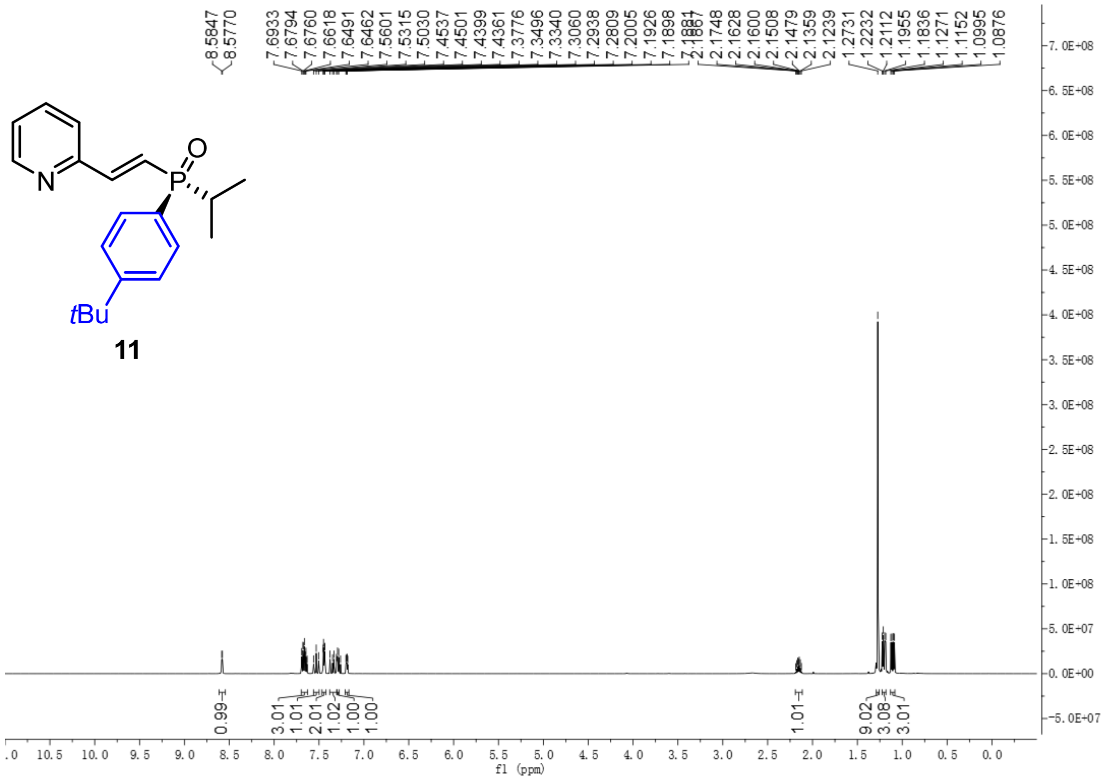
8.5847
8.5770
7.6933
7.6794
7.6760
7.6618
7.6491
7.6462
7.5601
7.5315
7.5030
7.4537
7.4501
7.4399
7.4361
7.3776
7.3496
7.3340
7.3060
7.2938
7.2809
7.2005
7.1926
7.1898
2.1748
2.1628
2.1600
2.1508
2.1479
2.1359
2.1239
1.2731
1.2232
1.2112
1.1955
1.1836
1.1271
1.1152
1.0995
1.0876
tBu
11
0.99
3.01
1.01
2.01
1.02
1.00
1.00
1.01
9.02
3.08
3.01
f1 (ppm)

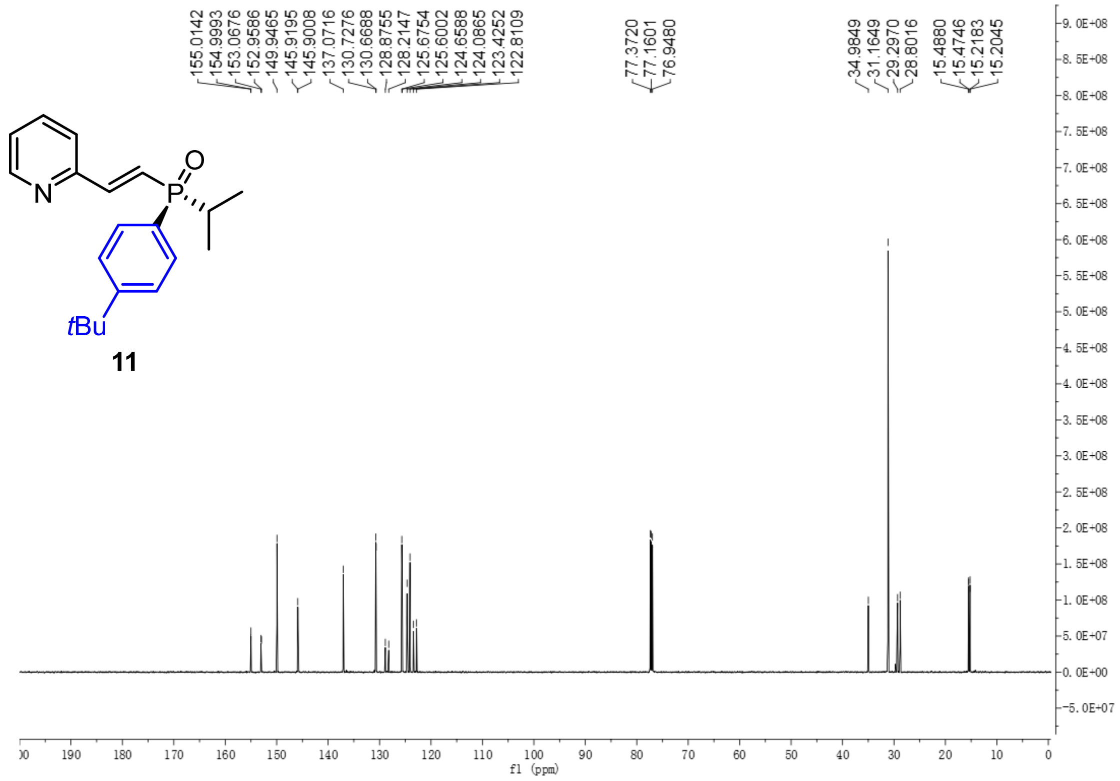
155.0142
154.9993
153.0676
152.9586
149.9465
145.9195
145.9008
137.0716
130.7276
130.6688
128.8755
128.2147
125.6754
125.6002
124.6588
124.0865
123.4252
122.8109
77.3720
77.1601
76.9480
34.9849
31.1649
29.2970
28.8016
15.4880
15.4746
15.2183
15.2045
tBu
11
f1 (ppm)

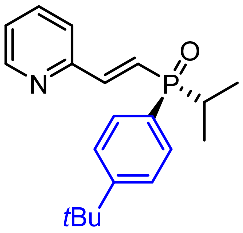

11

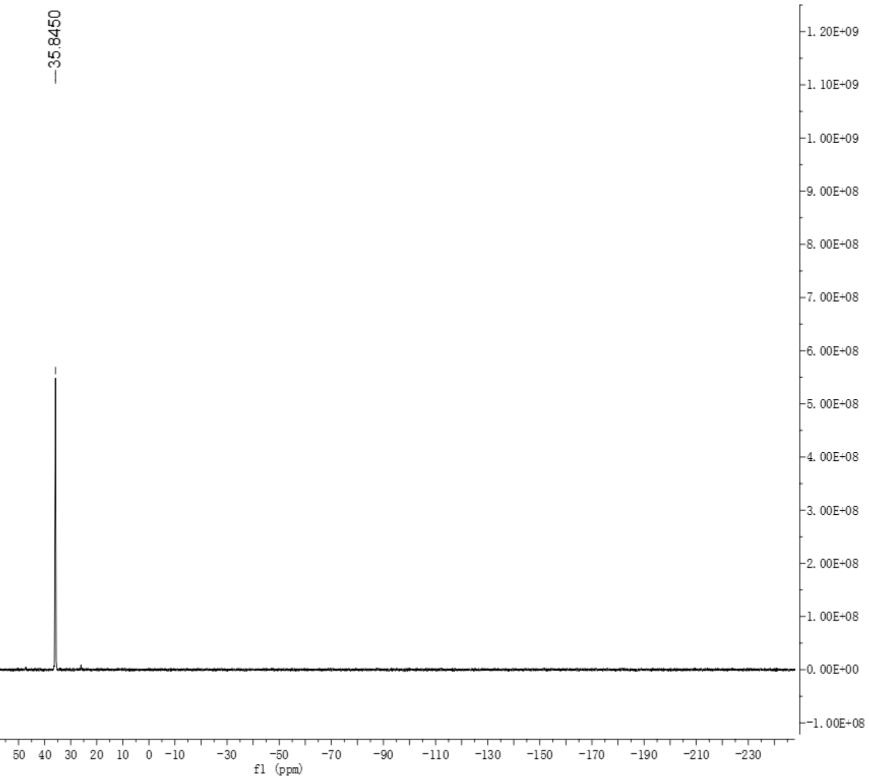

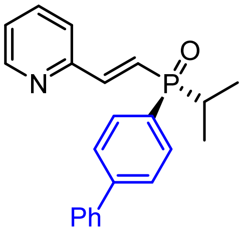

12

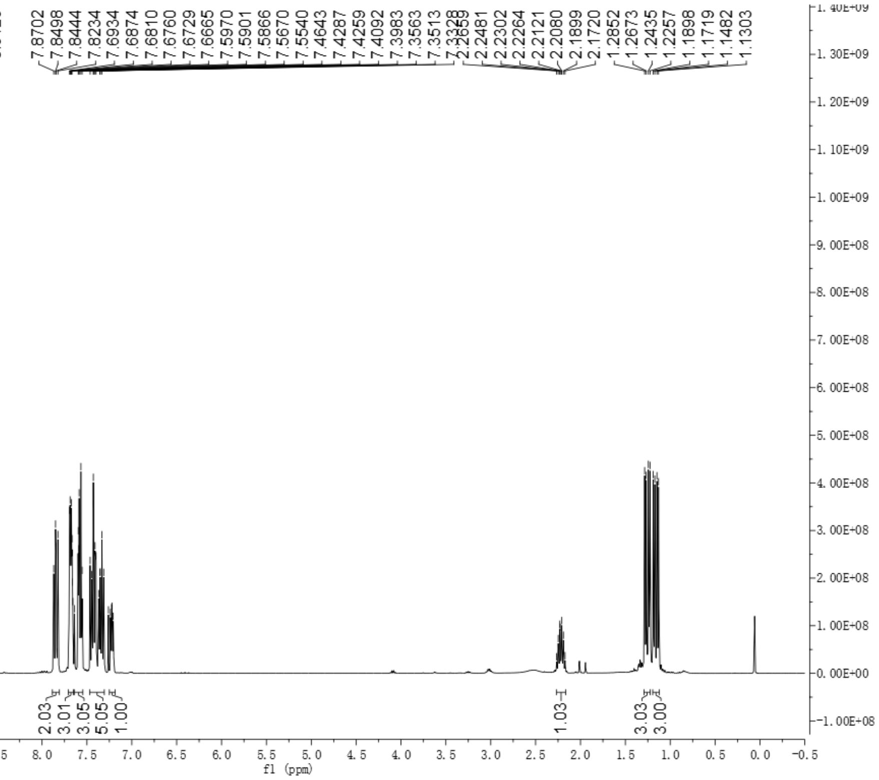

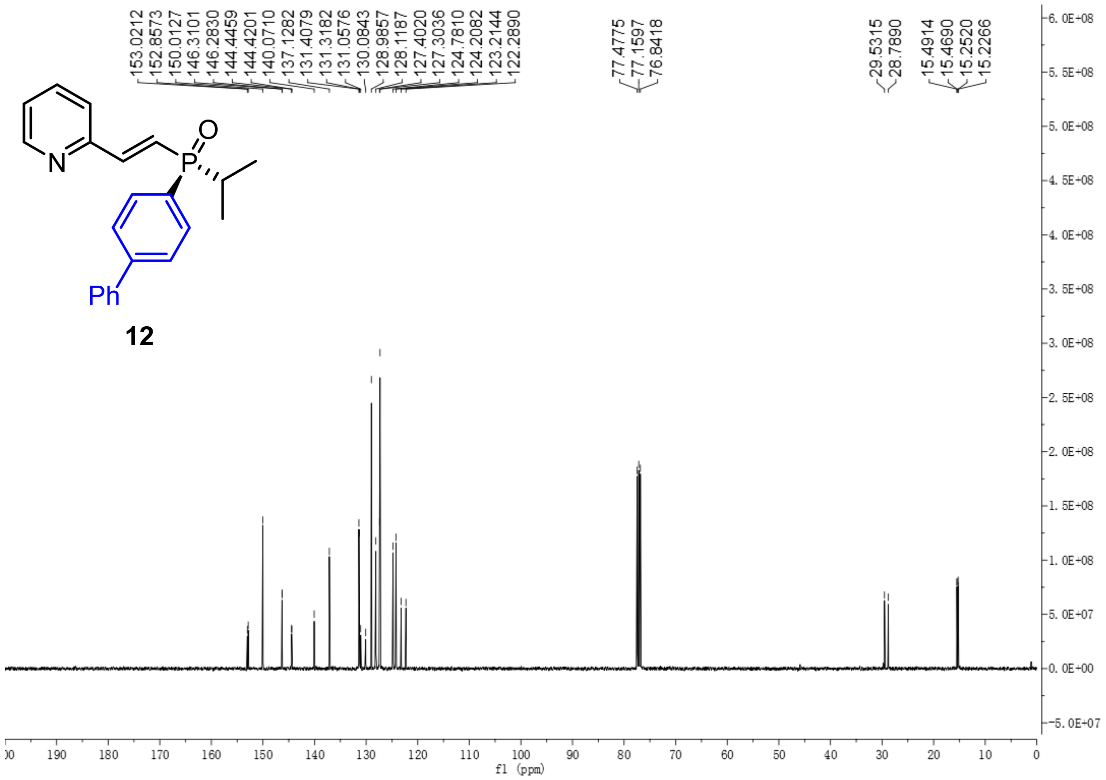

153.0212
152.8573
150.0127
146.3101
146.2830
144.4459
144.4201
140.0710
137.1282
131.4079
131.3182
131.0576
130.0843
128.9857
128.1187
127.4020
127.3036
124.7810
124.2082
123.2144
122.2890
77.4775
77.1597
76.8418
29.5315
28.7890
15.4914
15.4690
15.2520
15.2266
Ph
12
f1 (ppm)

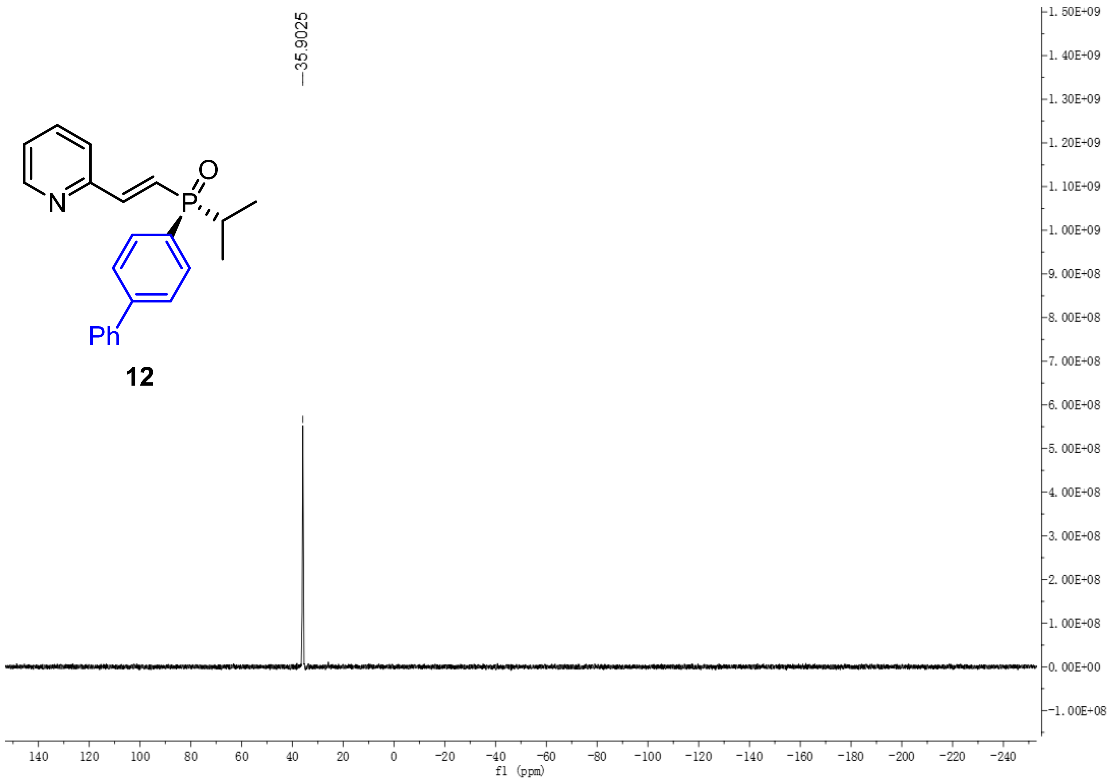

35.9025
Ph
12
f1 (ppm)

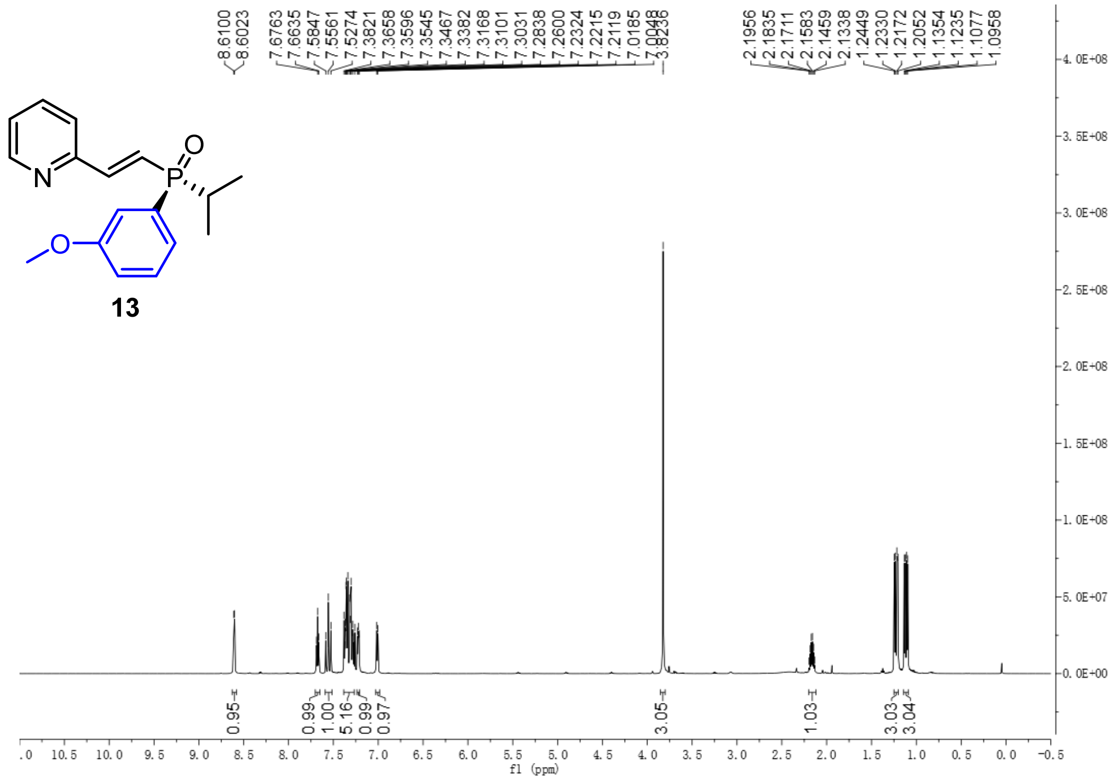
13
8.6100
8.6023
7.6763
7.6635
7.5847
7.5561
7.5274
7.3821
7.3658
7.3596
7.3545
7.3467
7.3382
7.3168
7.3101
7.3031
7.2838
7.2600
7.2324
7.2215
7.2119
7.0185
7.0048
3.8236
2.1956
2.1835
2.1711
2.1583
2.1459
2.1338
1.2449
1.2330
1.2172
1.2052
1.1354
1.1235
1.1077
1.0958
0.95
0.99
1.00
5.16
0.99
0.97
3.05
1.03
3.03
3.04
f1 (ppm)

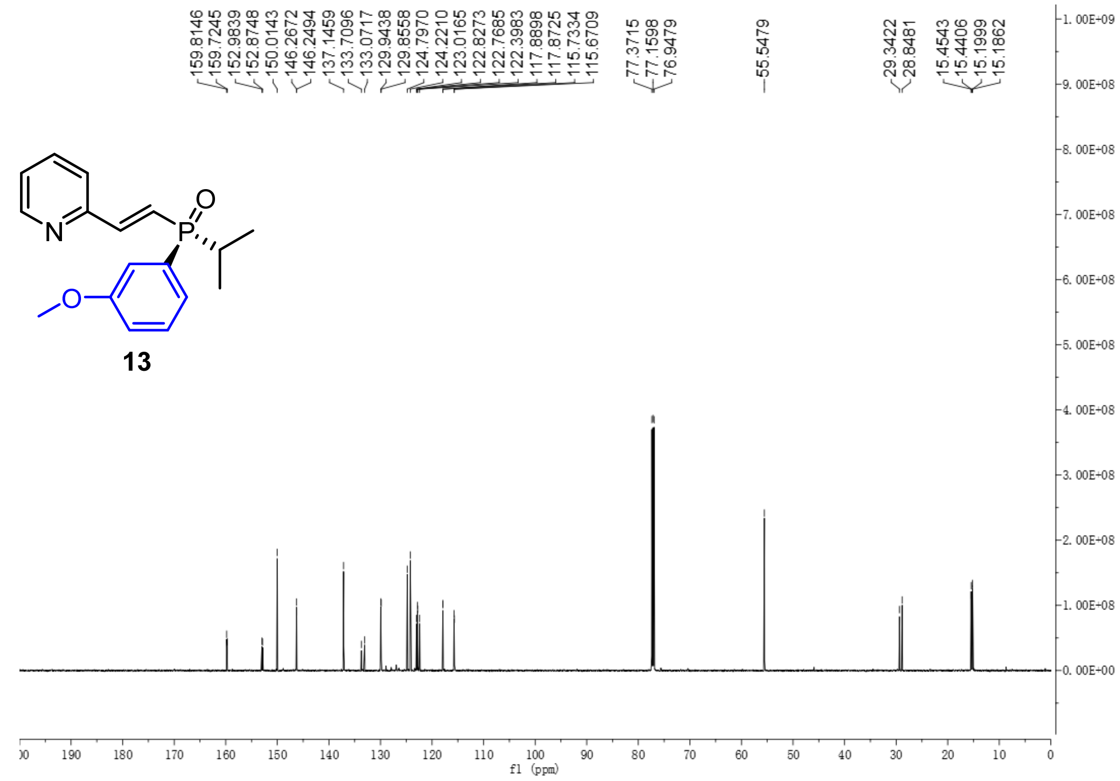
13
159.8146
159.7245
152.9839
152.8748
150.0143
146.2672
146.2494
137.1459
133.7096
133.0717
129.9438
129.8558
124.7970
124.2210
123.0165
122.8273
122.7685
122.3983
117.8898
117.8725
115.7334
115.6709
77.3715
77.1598
76.9479
55.5479
29.3422
28.8481
15.4543
15.4406
15.1999
15.1862
f1 (ppm)

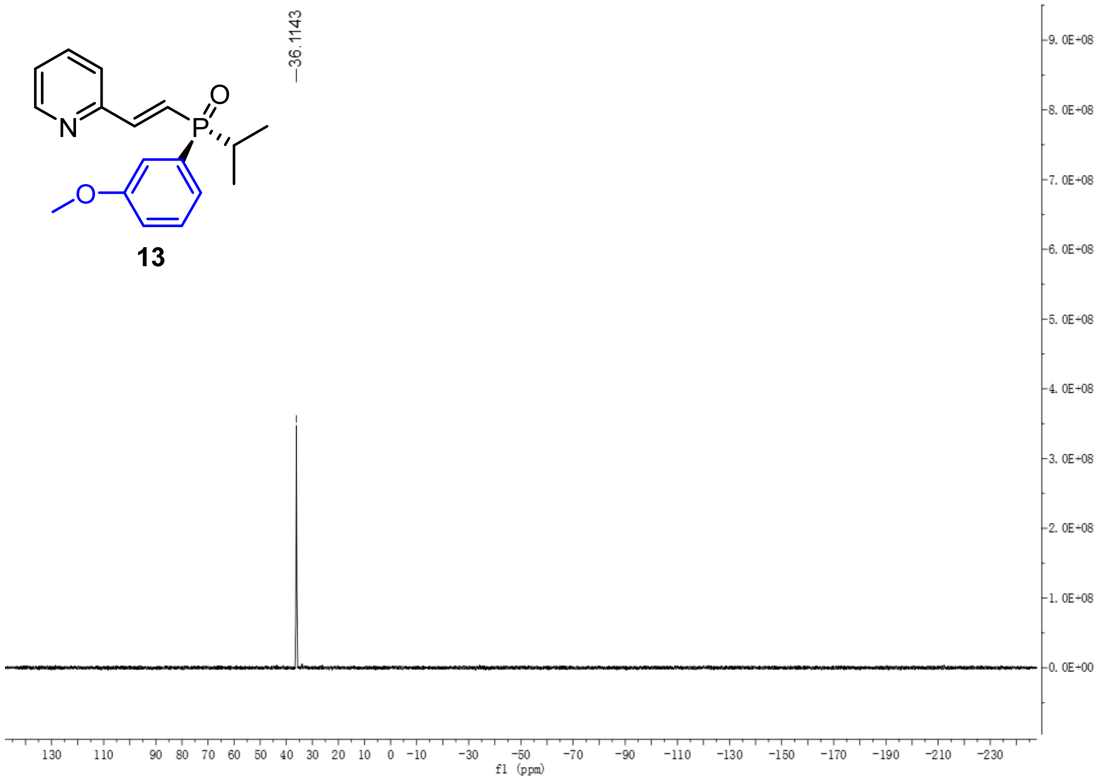

13
—36.1143
f1 (ppm)

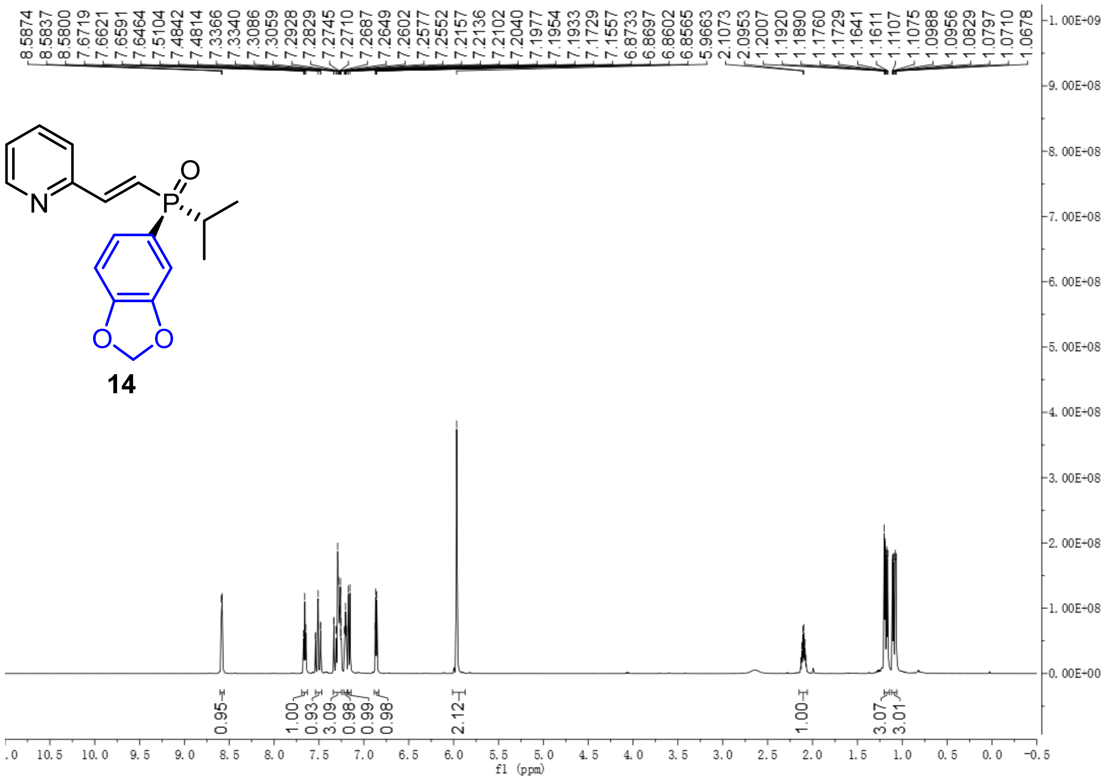

14
8.5874 8.5837 8.5800 7.6719 7.6621 7.6591 7.6464 7.5104 7.4842 7.4814 7.3366 7.3340 7.3086 7.3059 7.2928 7.2829 7.2745 7.2710 7.2687 7.2649 7.2602 7.2577 7.2552 7.2157 7.2136 7.2102 7.2040 7.1977 7.1954 7.1933 7.1729 7.1557 6.8733 6.8697 6.8602 6.8565 5.9663 2.1073 2.0953 1.2007 1.1920 1.1890 1.1760 1.1729 1.1641 1.1611 1.1107 1.1075 1.0988 1.0956 1.0829 1.0797 1.0710 1.0678
0.95 1.00 0.93 3.09 0.98 0.99 0.98 2.12 1.00 3.07 3.01
f1 (ppm)

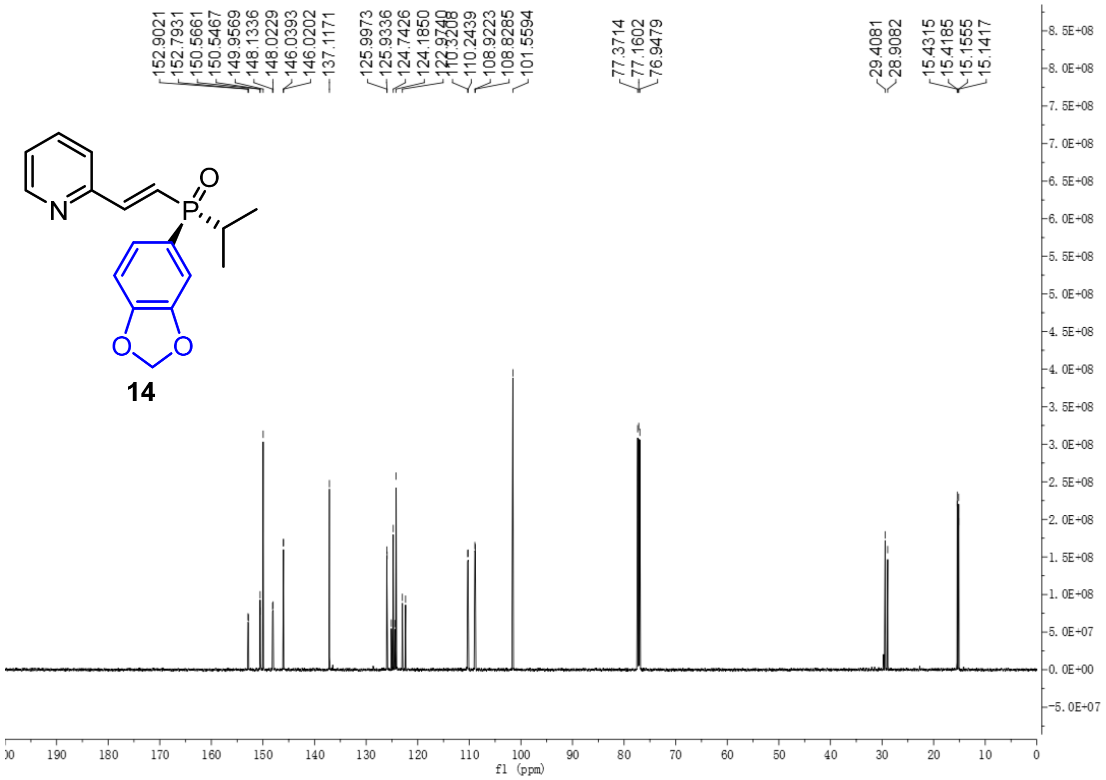

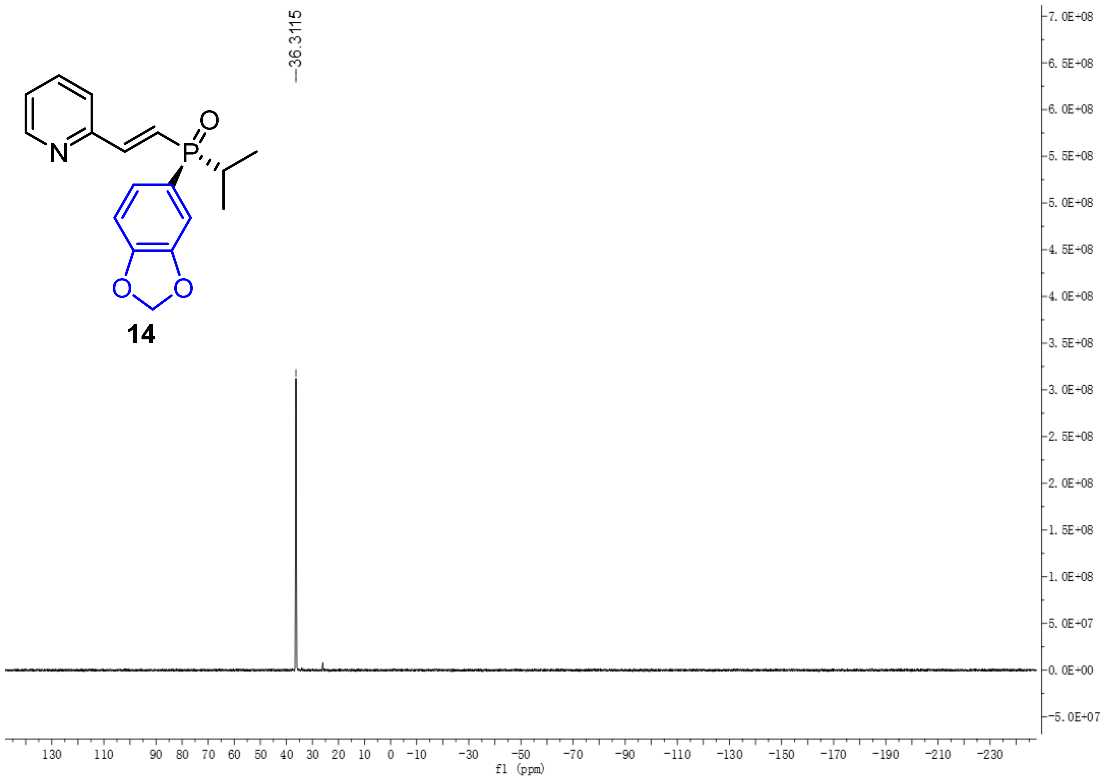

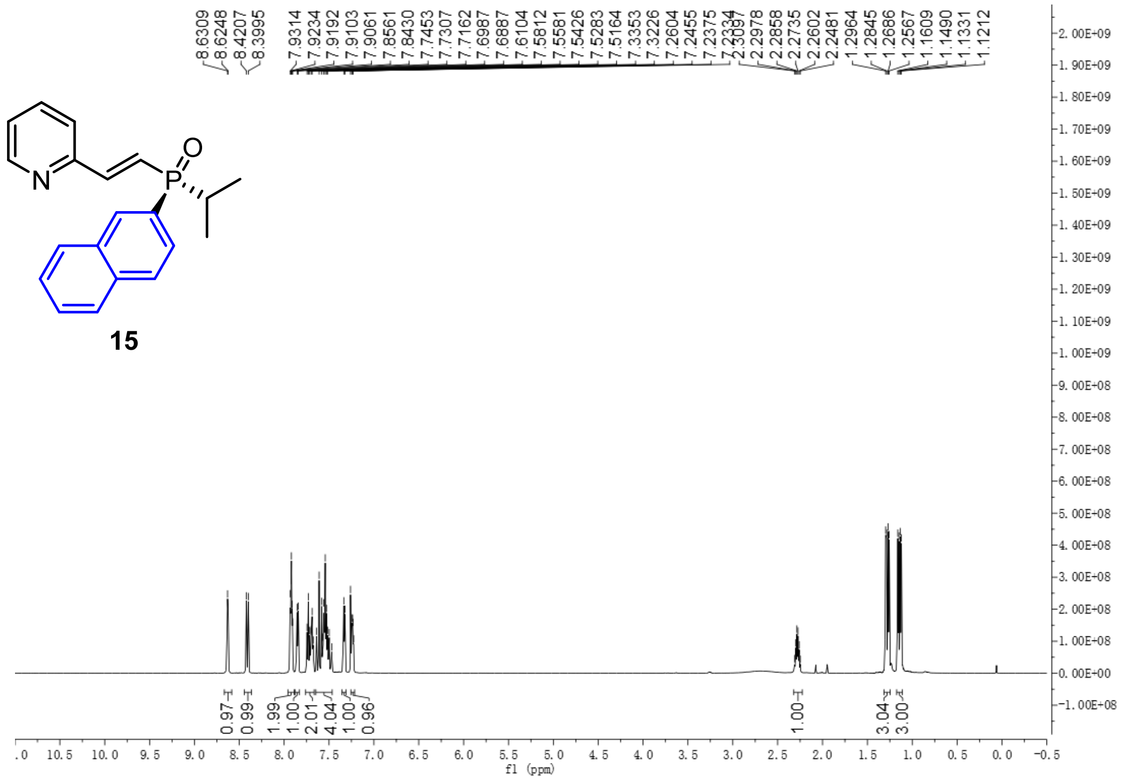

15

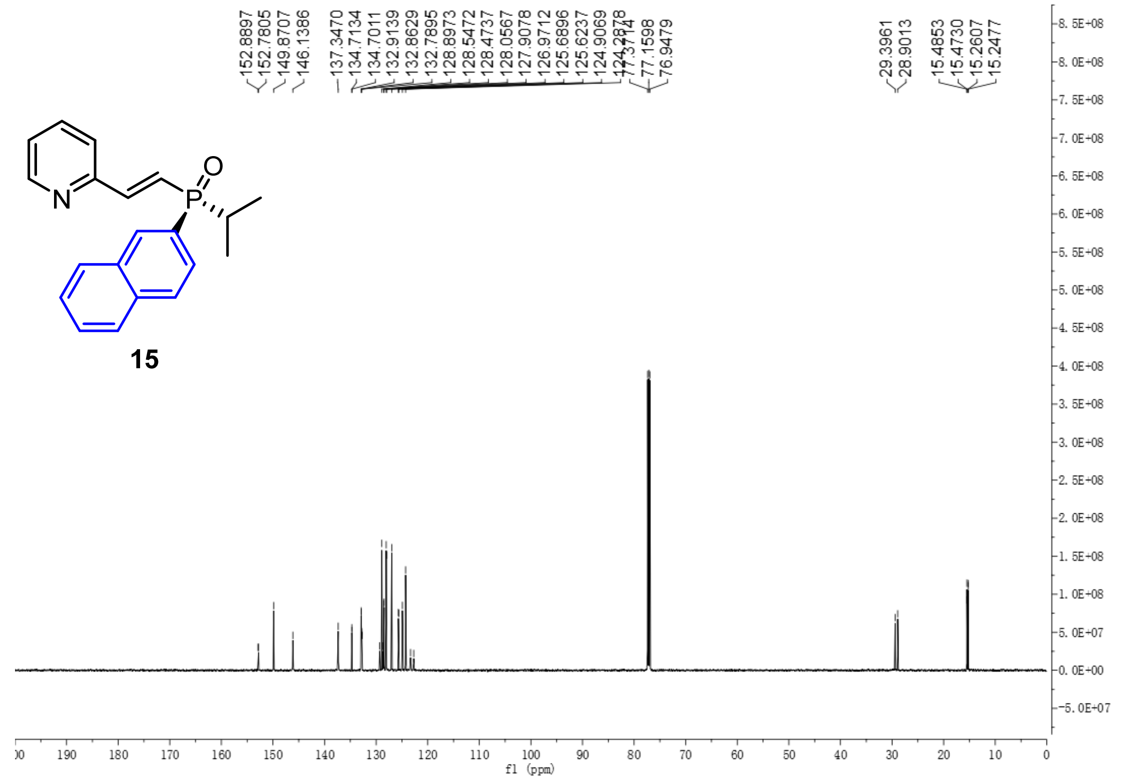

15

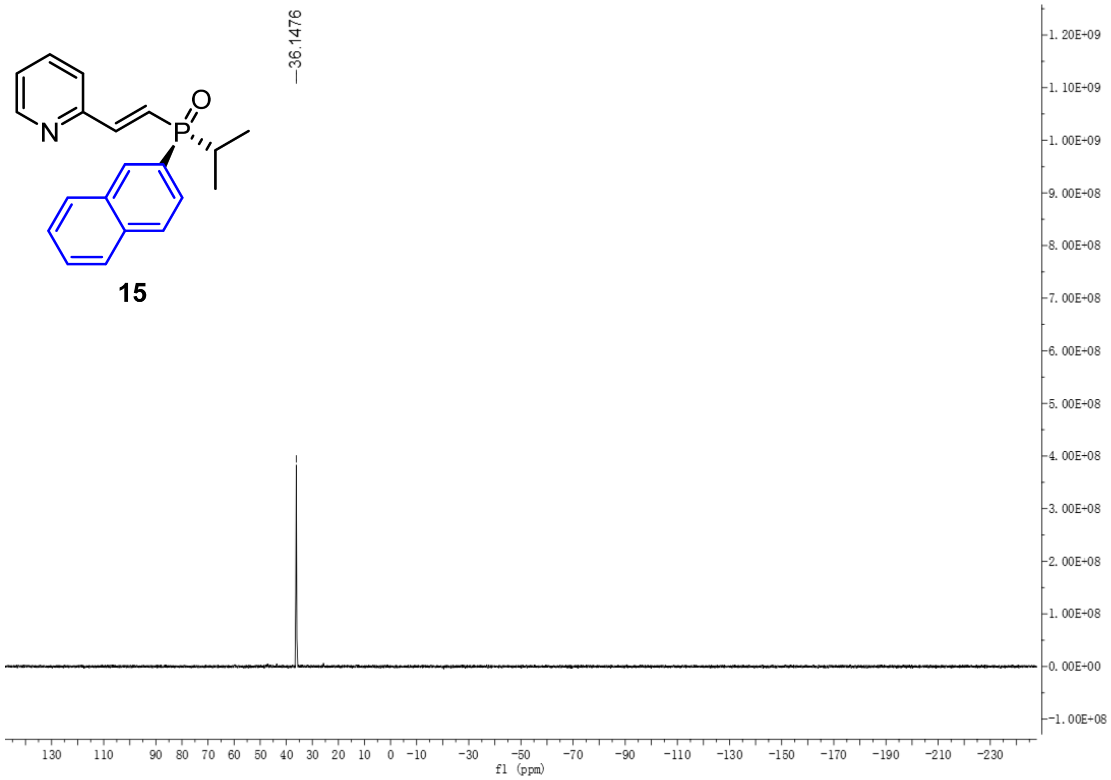

15
—36.1476
fl (ppm)

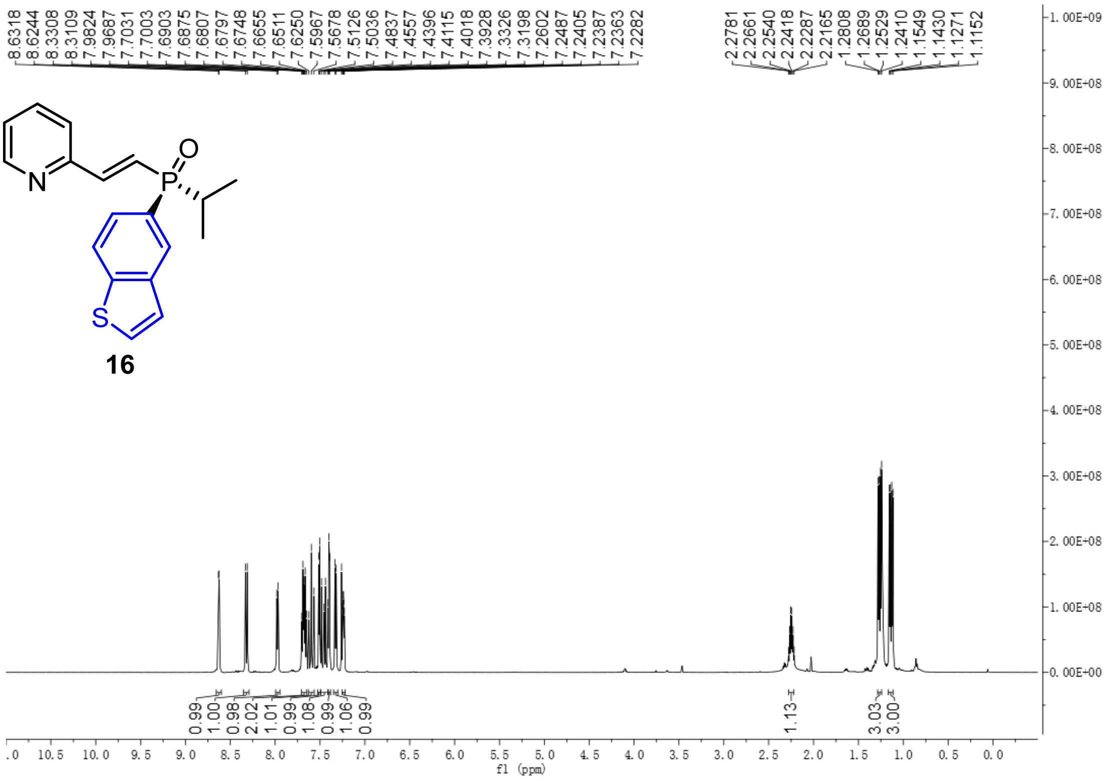

16
8.6318 8.6244 8.3308 8.3109 7.9824 7.9687 7.7031 7.7003 7.6903 7.6875 7.6807 7.6797 7.6748 7.6655 7.6511 7.6250 7.5967 7.5678 7.5126 7.5036 7.4837 7.4557 7.4396 7.4115 7.4018 7.3928 7.3326 7.3198 7.2602 7.2487 7.2405 7.2387 7.2363 7.2282
2.2781 2.2661 2.2540 2.2418 2.2287 2.2165 1.2808 1.2689 1.2529 1.2410 1.1549 1.1430 1.1271 1.1152
0.99 1.00 0.98 2.02 1.01 0.99 1.08 0.99 1.06 0.99
1.13
3.03 3.00
fl (ppm)

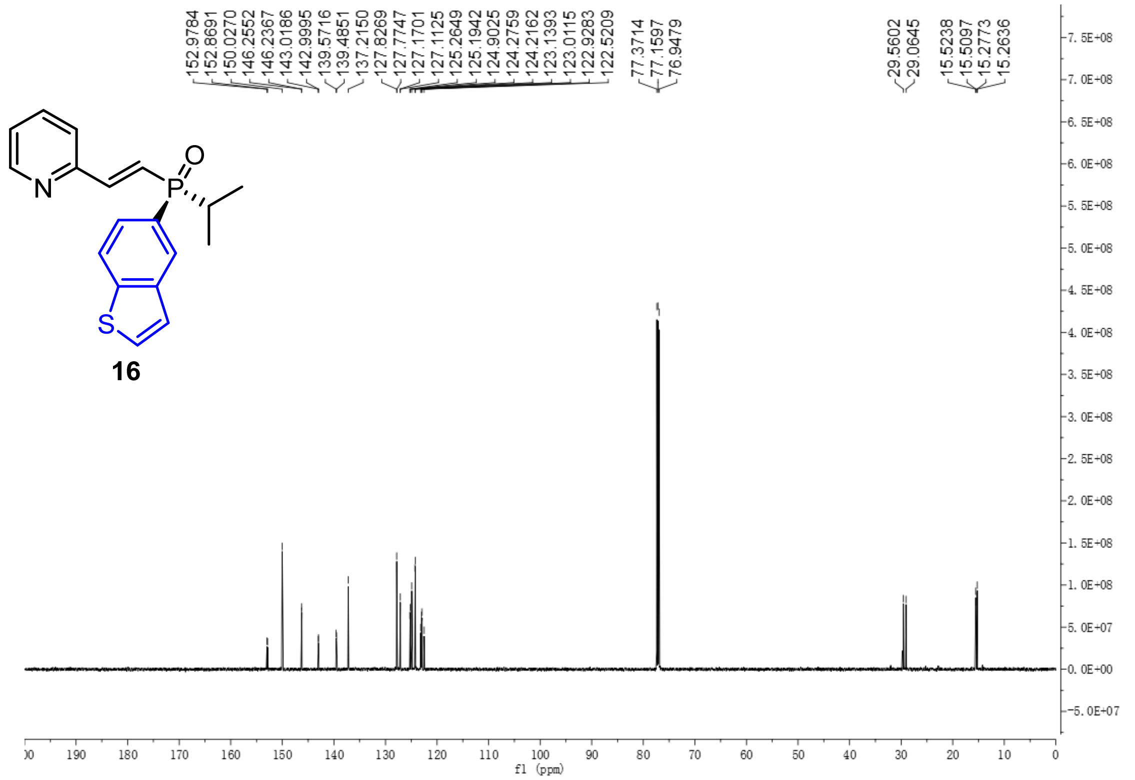
152.9784
152.8691
150.0270
146.2552
146.2367
143.0186
142.9995
139.5716
139.4851
137.2150
127.8269
127.7747
127.1701
127.1125
125.2649
125.1942
124.9025
124.2759
124.2162
123.1393
123.0115
122.9283
122.5209
77.3714
77.1597
76.9479
29.5602
29.0645
15.5238
15.5097
15.2773
15.2636
16
f1 (ppm)

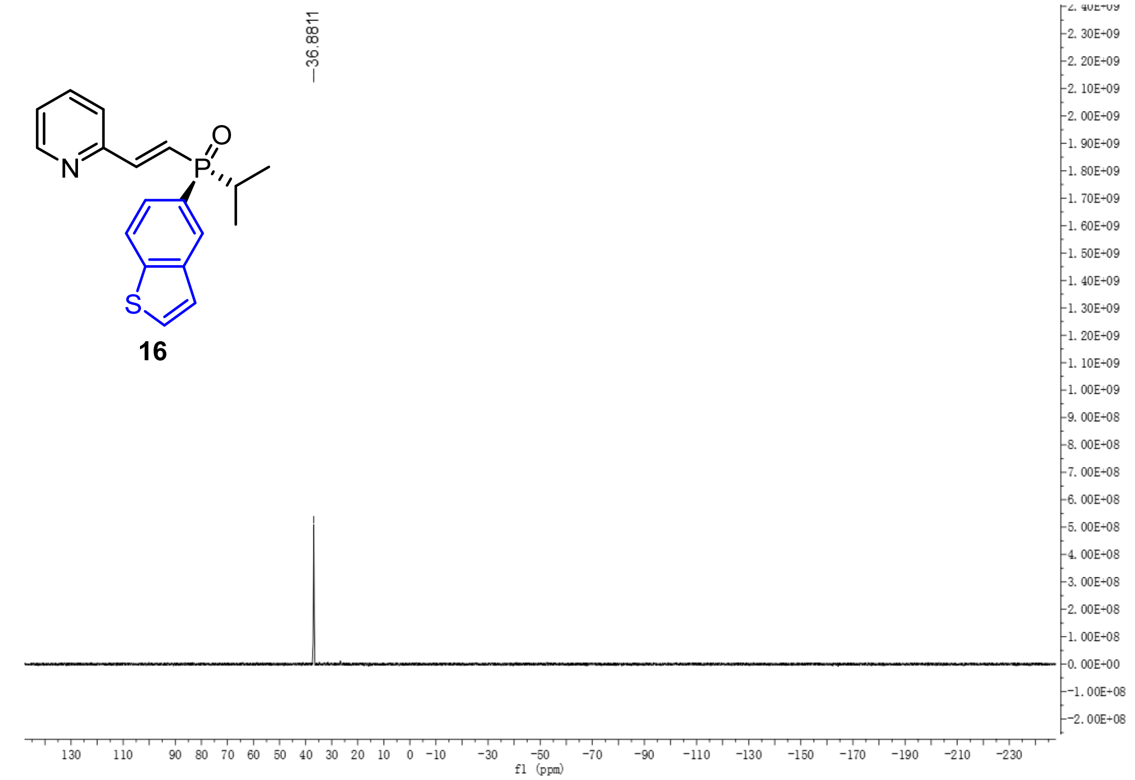
36.8811
16
f1 (ppm)

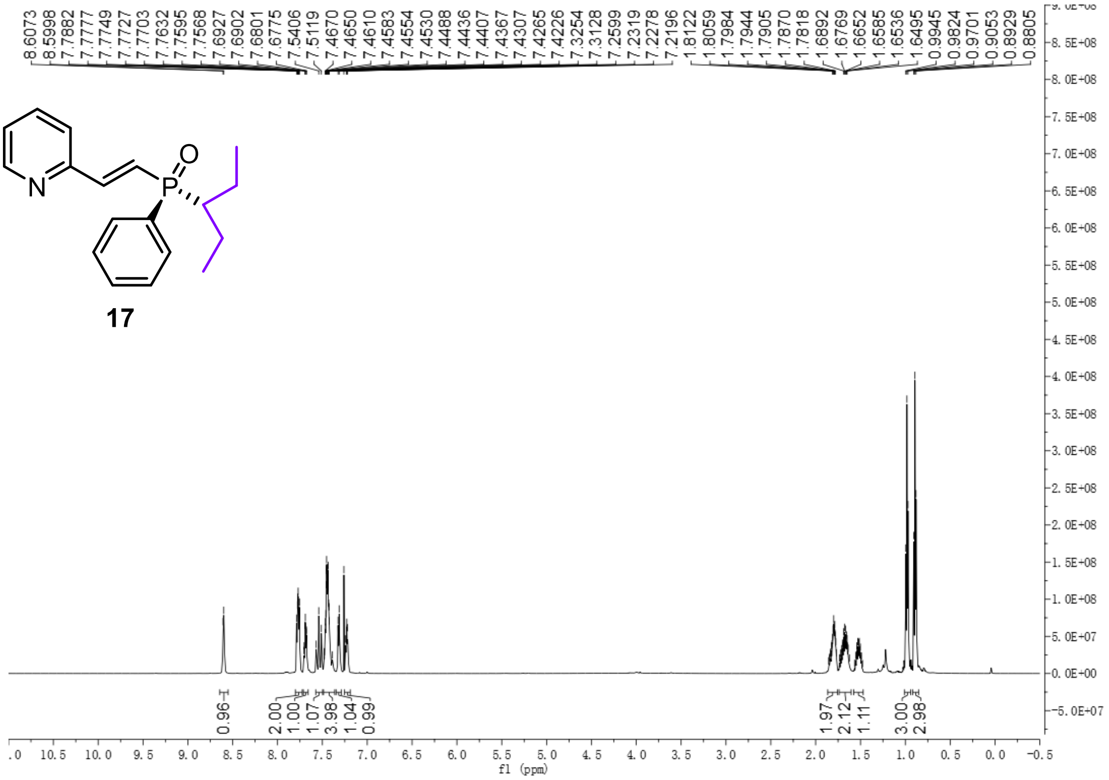
8.6073
8.5998
7.7882
7.7777
7.7749
7.7727
7.7703
7.7632
7.7595
7.7568
7.6927
7.6902
7.6801
7.6775
7.5406
7.5119
7.4670
7.4650
7.4610
7.4583
7.4554
7.4530
7.4488
7.4436
7.4407
7.4367
7.4307
7.4265
7.4226
7.3254
7.3128
7.2599
7.2319
7.2278
7.2196
1.8122
1.8059
1.7984
1.7944
1.7905
1.7870
1.7818
1.6892
1.6769
1.6652
1.6585
1.6536
1.6495
0.9945
0.9824
0.9701
0.9053
0.8929
0.8805
O
P
N
17
0.96
2.00
1.00
1.07
3.98
1.04
0.99
1.97
2.12
1.11
3.00
2.98
f1 (ppm)

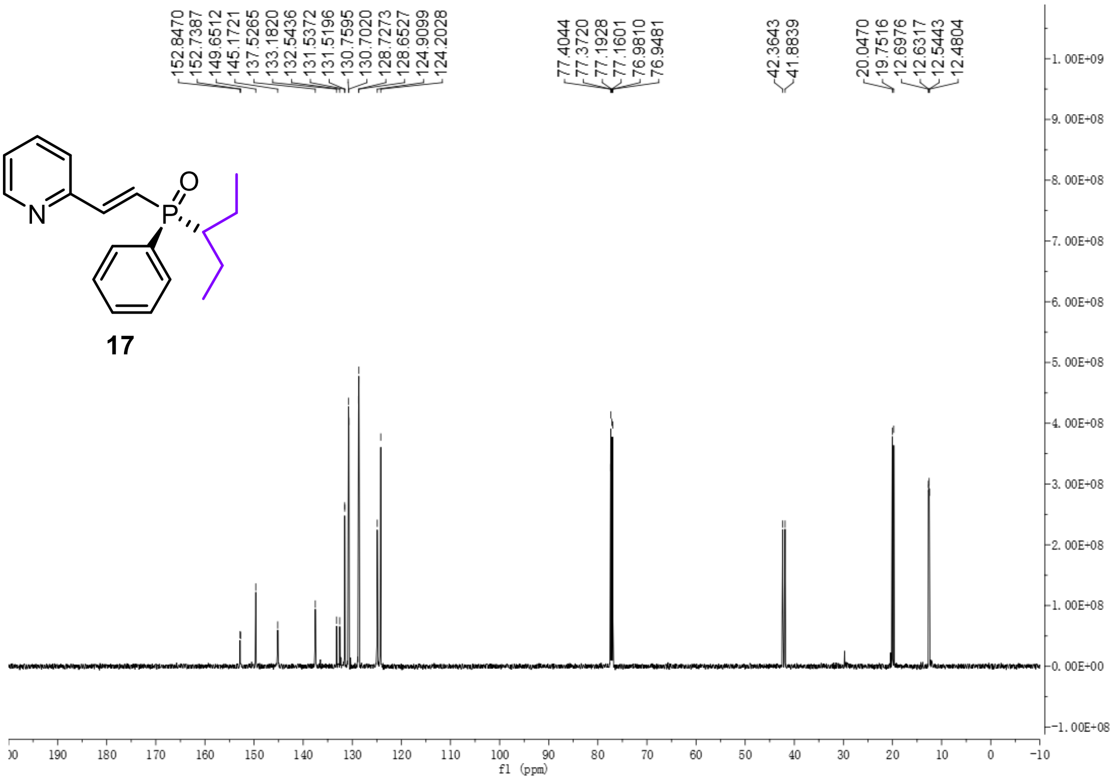
152.8470
152.7387
149.6512
145.1721
137.5265
133.1820
132.5436
131.5372
131.5196
130.7595
130.7020
128.7273
128.6527
124.9099
124.2028
77.4044
77.3720
77.1928
77.1601
76.9810
76.9481
42.3643
41.8839
20.0470
19.7516
12.6976
12.6317
12.5443
12.4804
O
P
N
17
f1 (ppm)

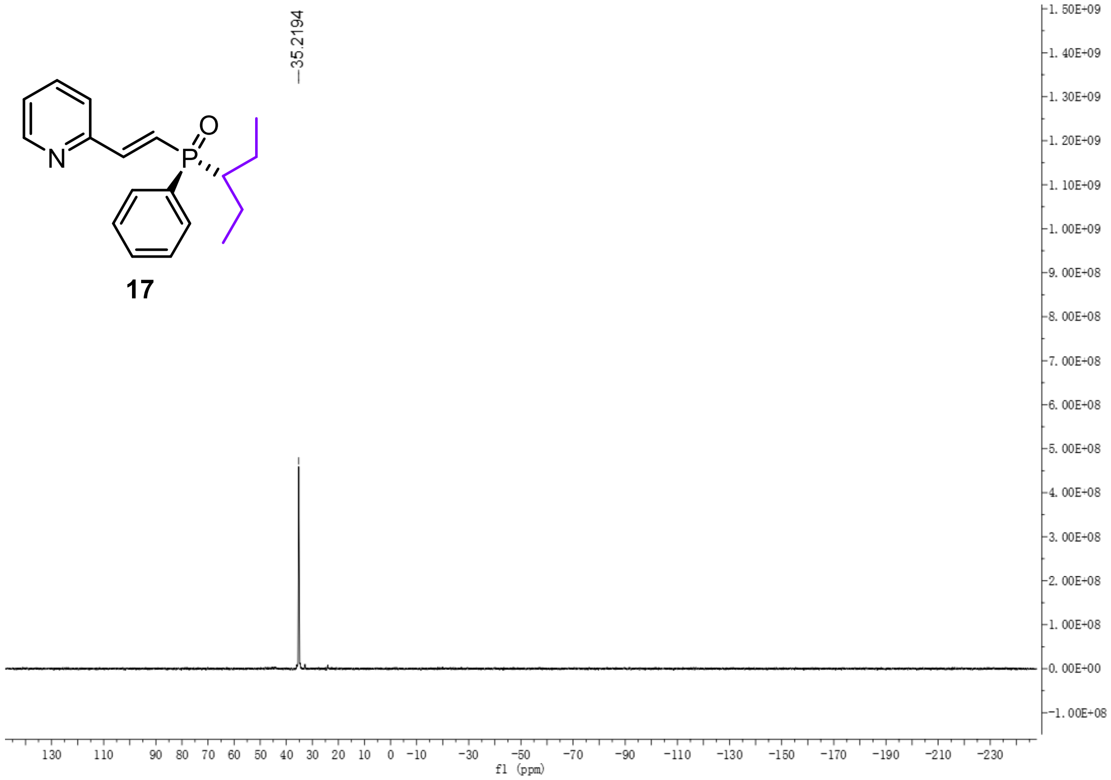
35.2194
f1 (ppm)

17

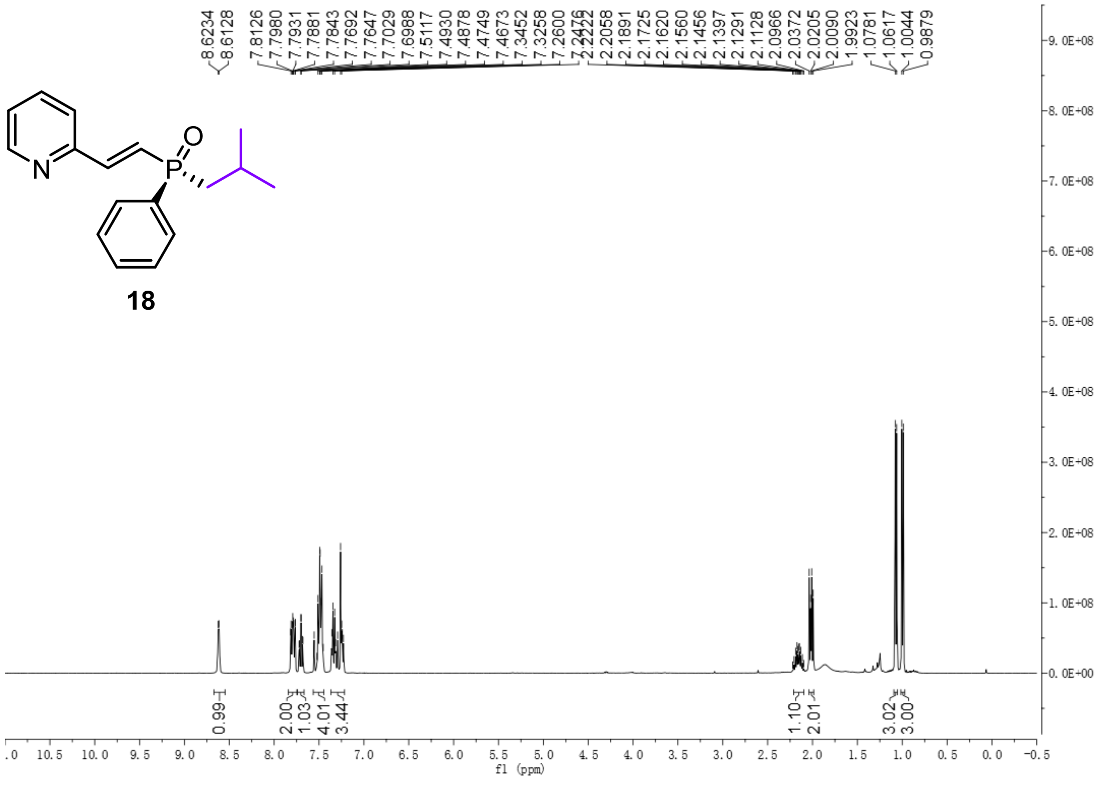
8.6234
8.6128
7.8126
7.7980
7.7931
7.7881
7.7843
7.7692
7.7647
7.7029
7.6988
7.5117
7.4930
7.4878
7.4749
7.4673
7.3452
7.3258
7.2600
2.2058
2.1891
2.1725
2.1620
2.1560
2.1456
2.1397
2.1291
2.1128
2.0966
2.0372
2.0205
2.0090
1.9923
1.0781
1.0617
1.0044
0.9879
0.99
2.00
1.03
4.01
3.44
1.10
2.01
3.02
3.00
f1 (ppm)

18

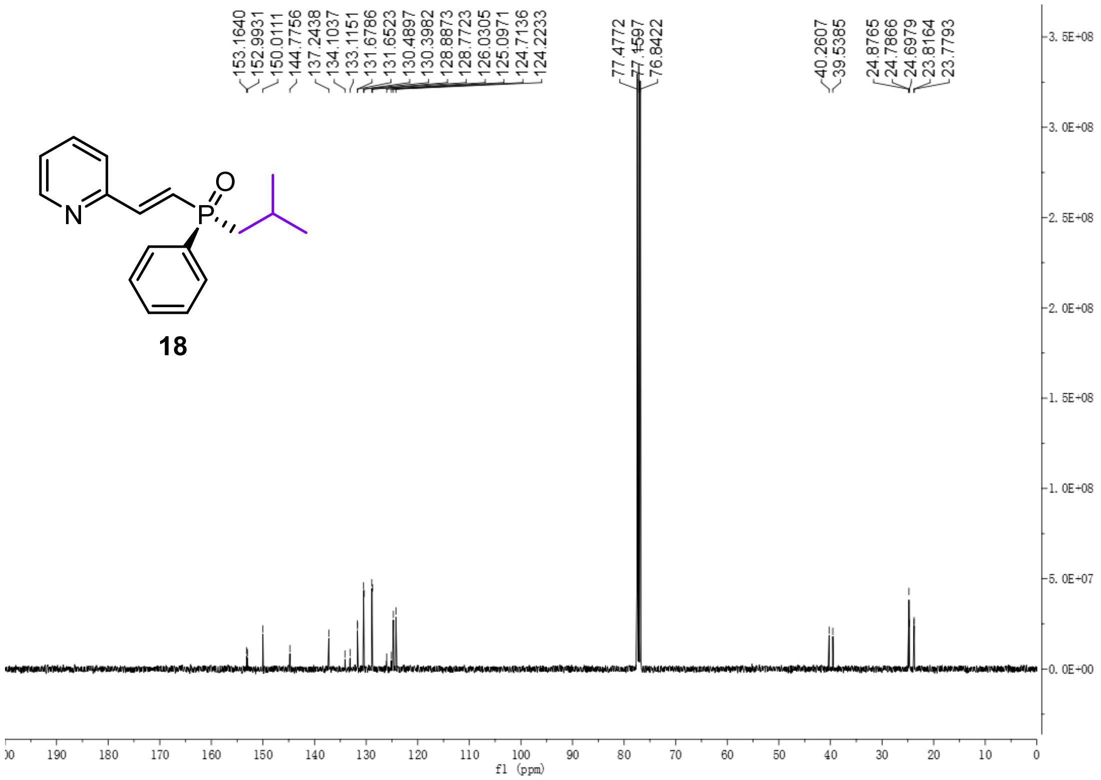
153.1640
152.9931
150.0111
144.7756
137.2438
134.1037
133.1151
131.6786
131.6523
130.4897
130.3982
128.8873
128.7723
126.0305
125.0971
124.7136
124.2233
77.4772
77.1597
76.8422
40.2607
39.5385
24.8765
24.7866
24.6979
23.8164
23.7793
N
O
P
18
f1 (ppm)

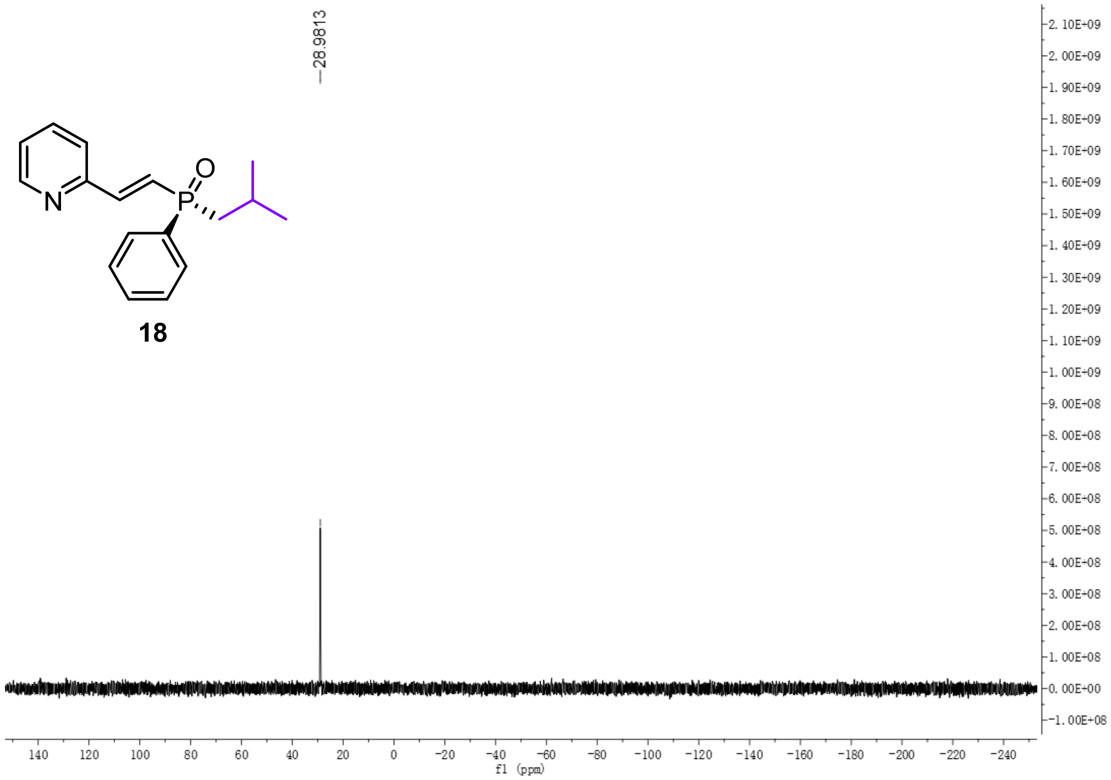
28.9813
N
O
P
18
f1 (ppm)

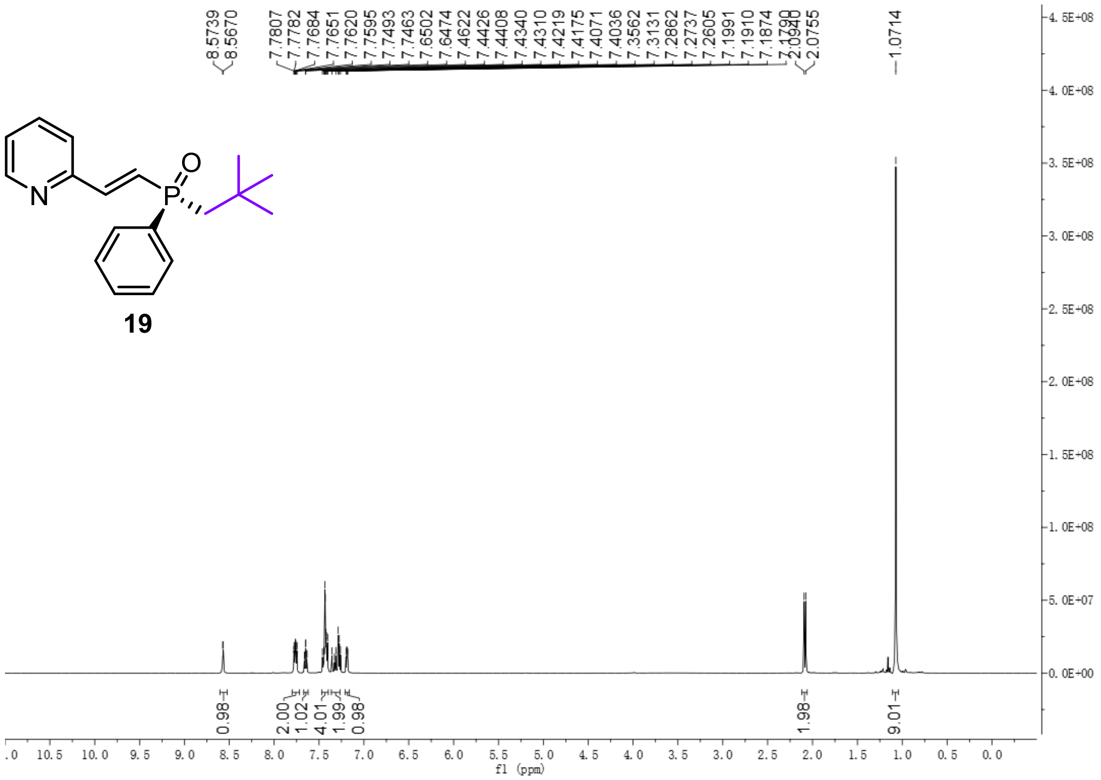

19

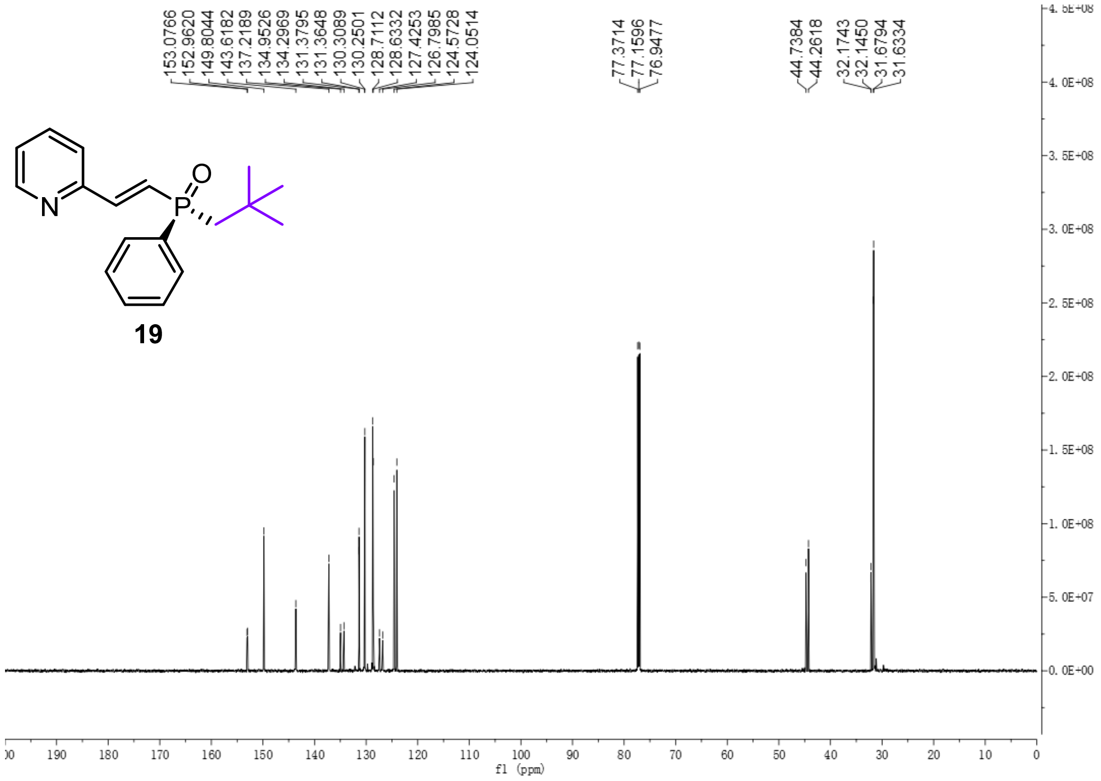

19

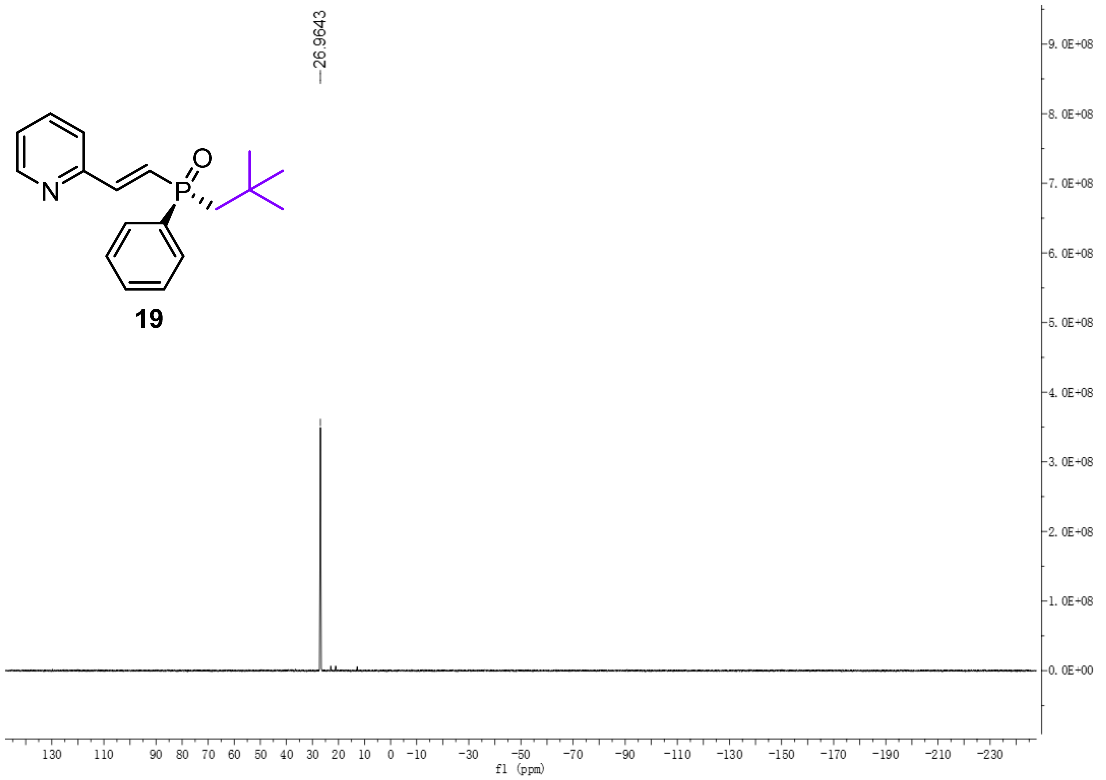

19
—26.9643
f1 (ppm)

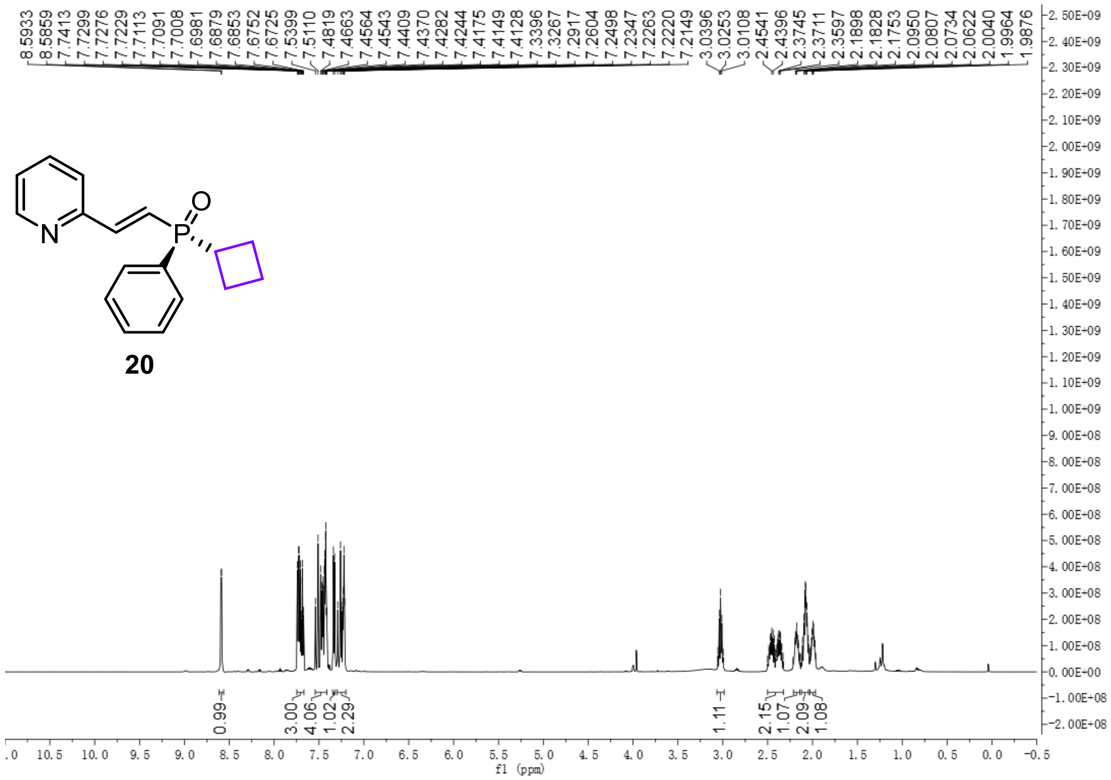

20
8.5933
8.5859
7.7413
7.7299
7.7276
7.7229
7.7113
7.7091
7.7008
7.6981
7.6879
7.6853
7.6752
7.6725
7.5399
7.5110
7.4819
7.4663
7.4564
7.4543
7.4409
7.4370
7.4282
7.4244
7.4175
7.4149
7.4128
7.3396
7.3267
7.2917
7.2604
7.2498
7.2347
7.2263
7.2220
7.2149
3.0396
3.0253
3.0108
2.4541
2.4396
2.3745
2.3711
2.3597
2.1898
2.1828
2.1753
2.0950
2.0807
2.0734
2.0622
2.0040
1.9964
1.9876
0.99
3.00
4.06
1.02
2.29
1.11
2.15
1.07
2.09
1.08
f1 (ppm)

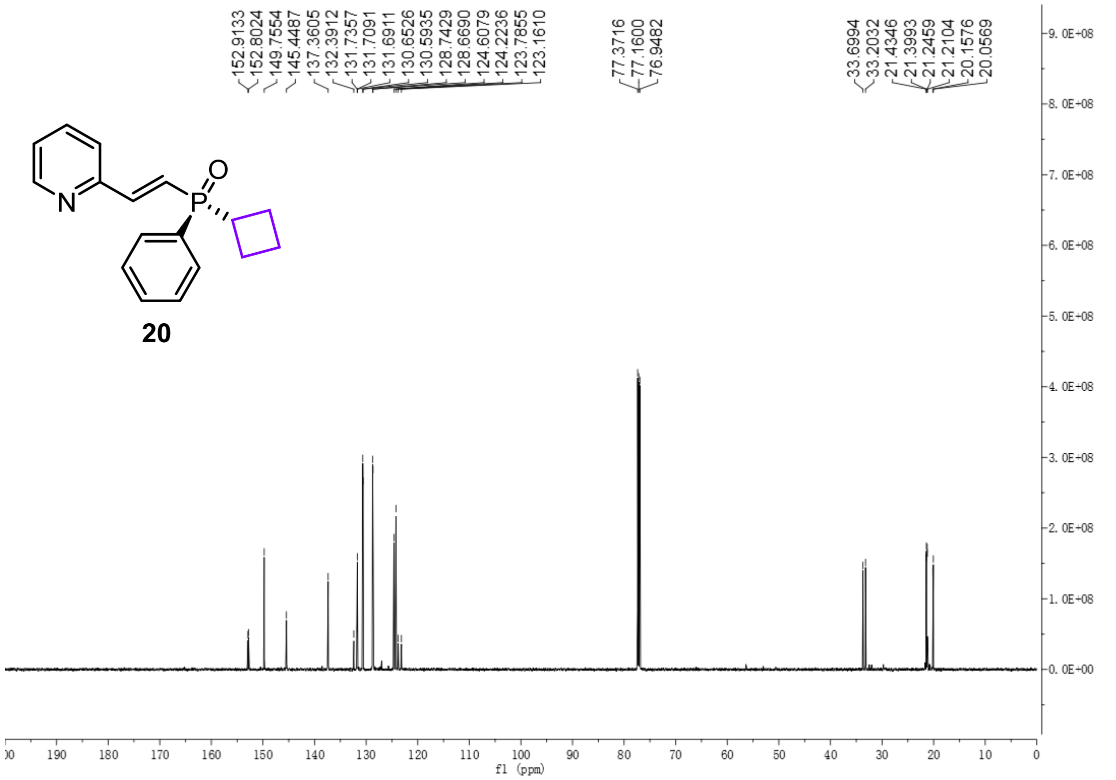

20
152.9133
152.8024
149.7554
145.4487
137.3605
132.3912
131.7357
131.7091
131.6911
130.6526
130.5935
128.7429
128.6690
124.6079
124.2236
123.7855
123.1610
77.3716
77.1600
76.9482
33.6994
33.2032
21.4346
21.3993
21.2459
21.2104
20.1576
20.0569
f1 (ppm)

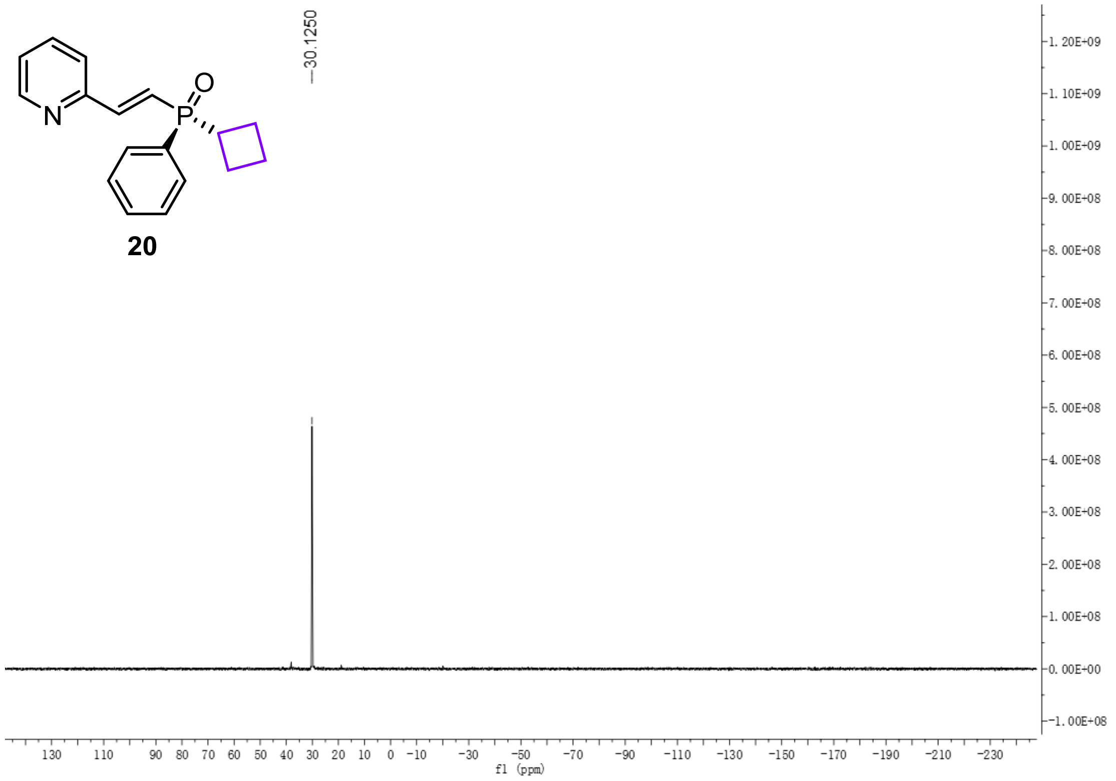

20
30.1250
f1 (ppm)

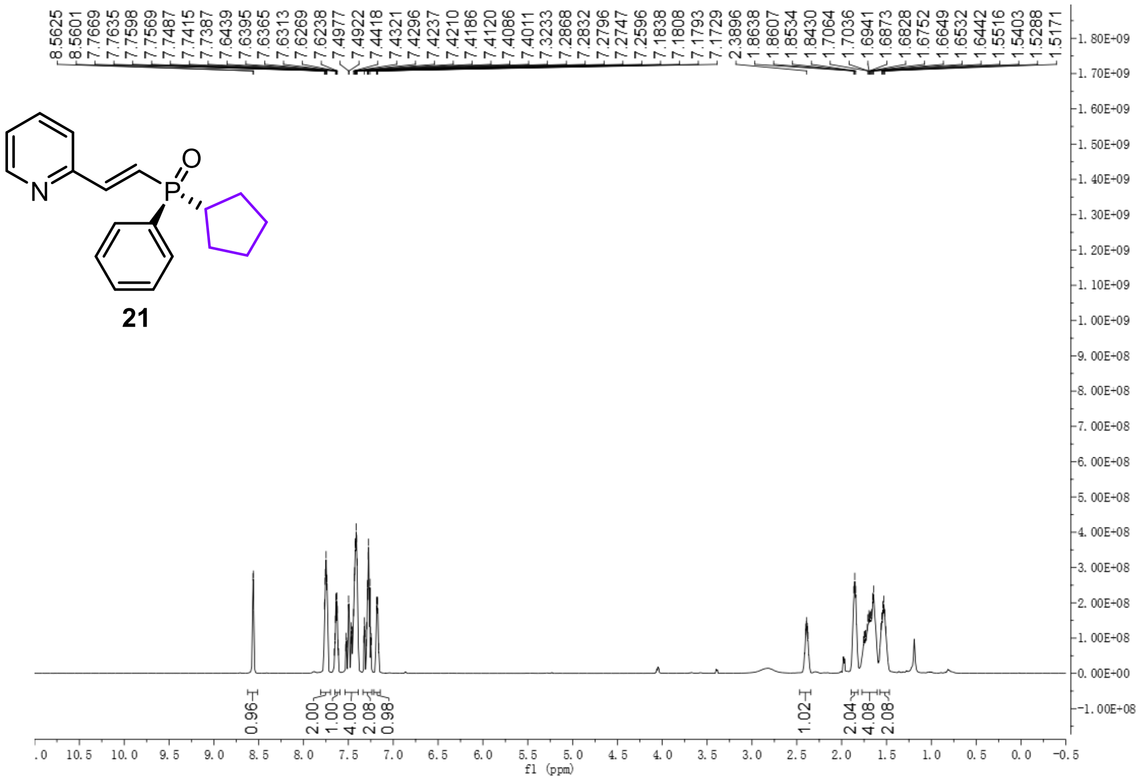

8.5625
8.5601
7.7669
7.7635
7.7598
7.7569
7.7487
7.7415
7.7387
7.6439
7.6395
7.6365
7.6313
7.6269
7.6238
7.4977
7.4922
7.4418
7.4321
7.4296
7.4237
7.4210
7.4186
7.4120
7.4086
7.4011
7.3233
7.2868
7.2832
7.2796
7.2747
7.2596
7.1838
7.1808
7.1793
7.1729
2.3896
1.8638
1.8607
1.8534
1.8430
1.7064
1.7036
1.6941
1.6873
1.6828
1.6752
1.6649
1.6532
1.6442
1.5516
1.5403
1.5288
1.5171
21
0.96
2.00
1.00
4.00
2.08
0.98
1.02
2.04
4.08
2.08
f1 (ppm)

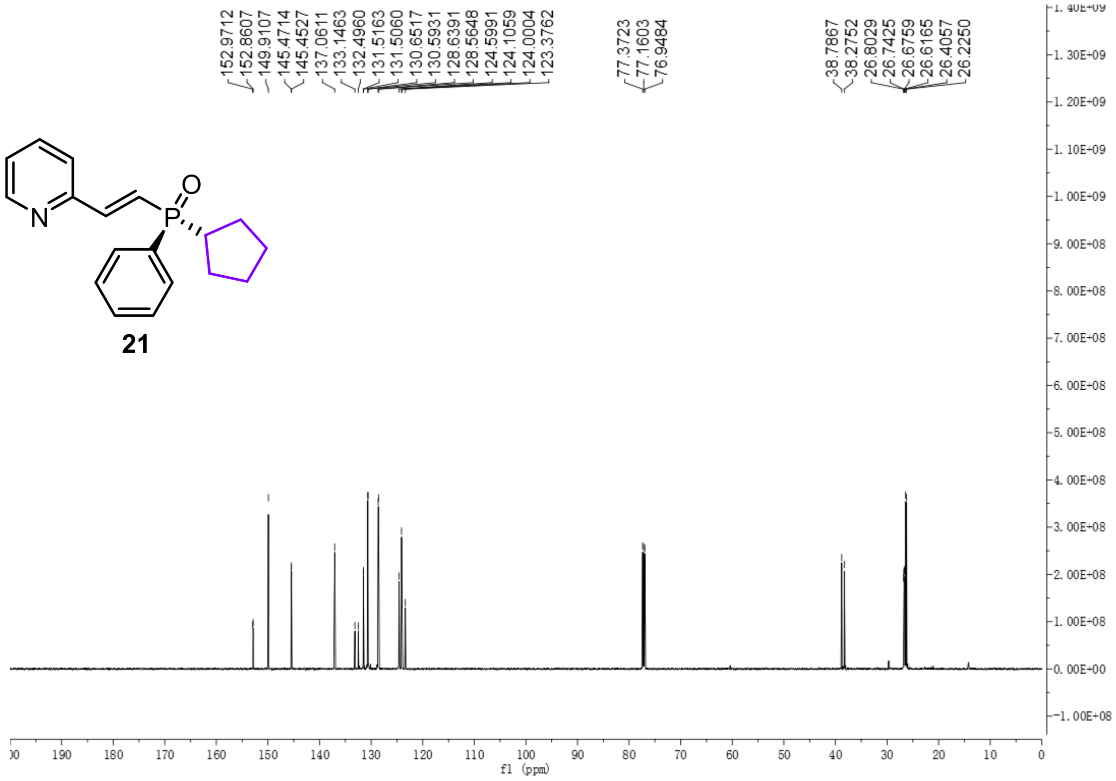

152.9712
152.8607
149.9107
145.4714
145.4527
137.0611
133.1463
132.4960
131.5163
131.5060
130.6517
130.5931
128.6391
128.5648
124.5991
124.1059
124.0004
123.3762
77.3723
77.1603
76.9484
38.7867
38.2752
26.8029
26.7425
26.6759
26.6165
26.4057
26.2250
21
f1 (ppm)

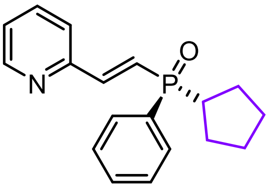
N
O
P

21

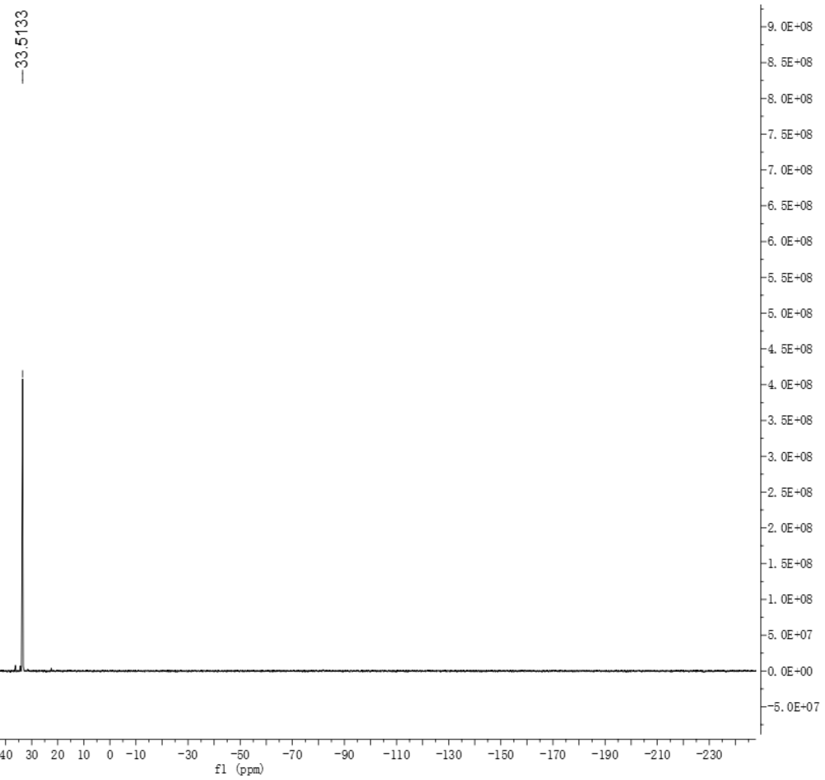
—33.5133
fl (ppm)

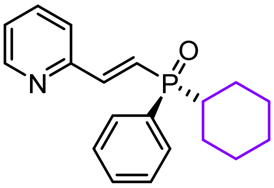
N
O
P

22

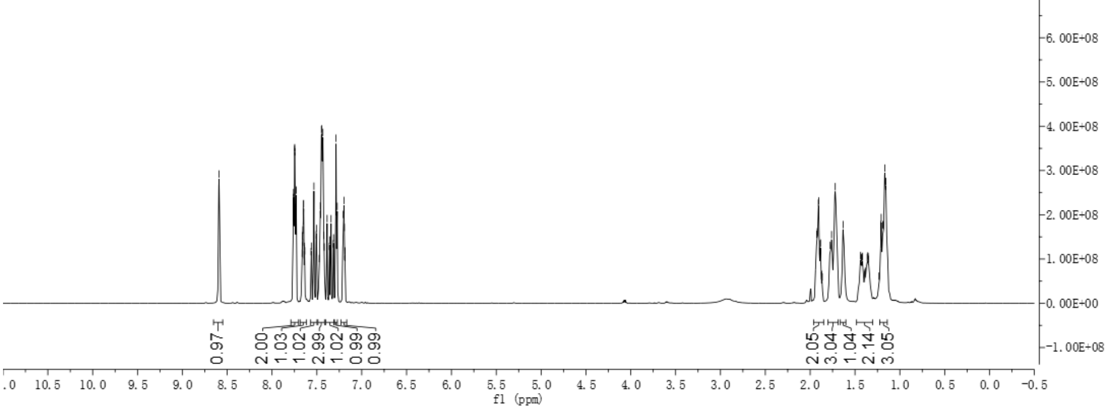
8.5909
7.7617
7.7600
7.7487
7.7475
7.7459
7.7340
7.7309
7.6607
7.6581
7.6551
7.6520
7.6504
7.6480
7.5358
7.5329
7.5075
7.5040
7.4611
7.4592
7.4495
7.4446
7.4404
7.4374
7.4326
7.4223
7.3892
7.3860
7.3580
7.3450
7.3417
7.3170
7.3137
7.2865
7.2730
7.2059
7.1984
7.1935
1.9257
1.9208
1.9085
1.9049
1.8892
1.8849
1.7762
1.7611
1.7204
1.6331
1.2111
1.2077
1.1894
1.1849
1.1677
1.1554
0.97
2.00
1.03
1.02
2.99
1.02
0.99
0.99
2.05
3.04
1.04
2.14
3.05
fl (ppm)

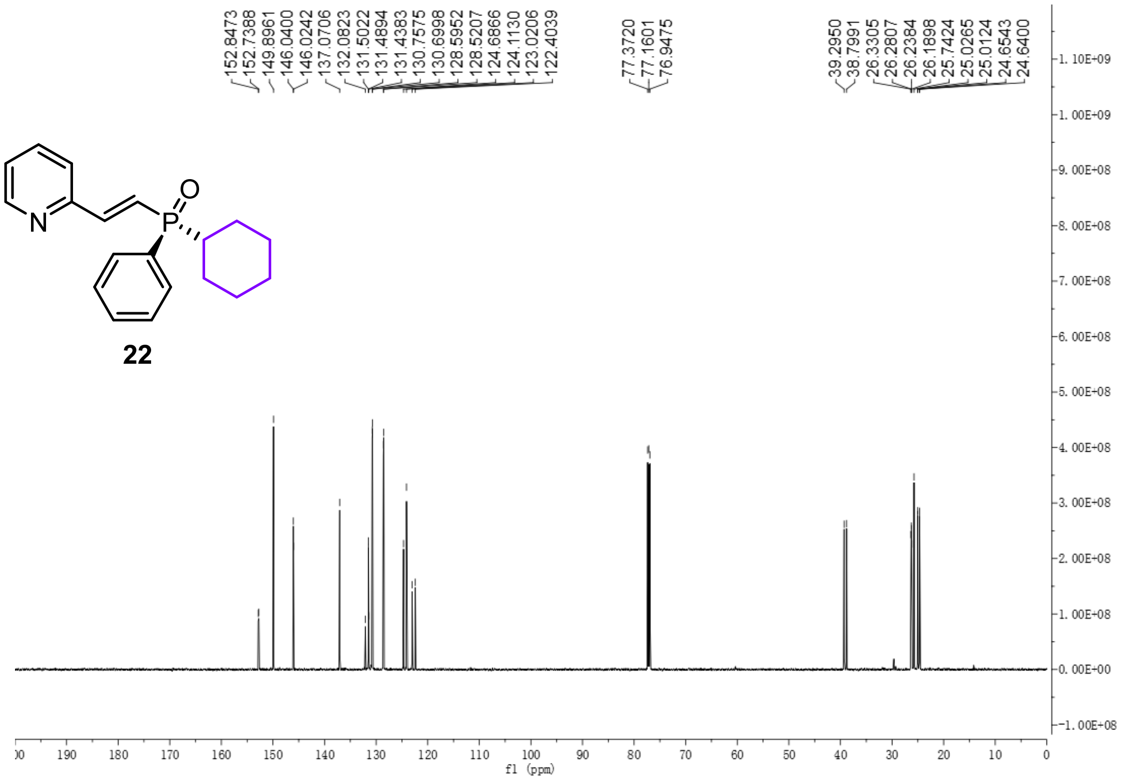
152.8473
152.7388
149.8961
146.0400
146.0242
137.0706
132.0823
131.5022
131.4894
131.4383
130.7575
130.6998
128.5952
128.5207
124.6866
124.1130
123.0206
122.4039
77.3720
77.1601
76.9475
39.2950
38.7991
26.3305
26.2807
26.2384
26.1898
25.7424
25.0265
25.0124
24.6543
24.6400
N
O
P
f1 (ppm)

22

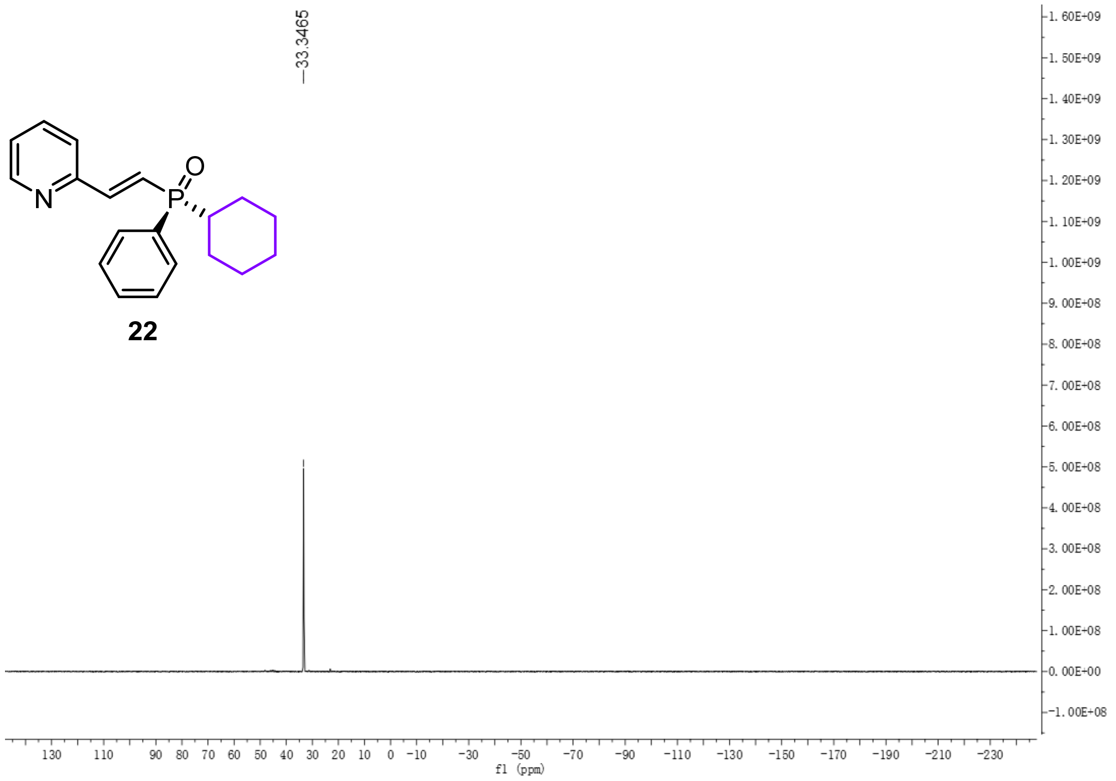
33.3465
N
O
P
f1 (ppm)

22

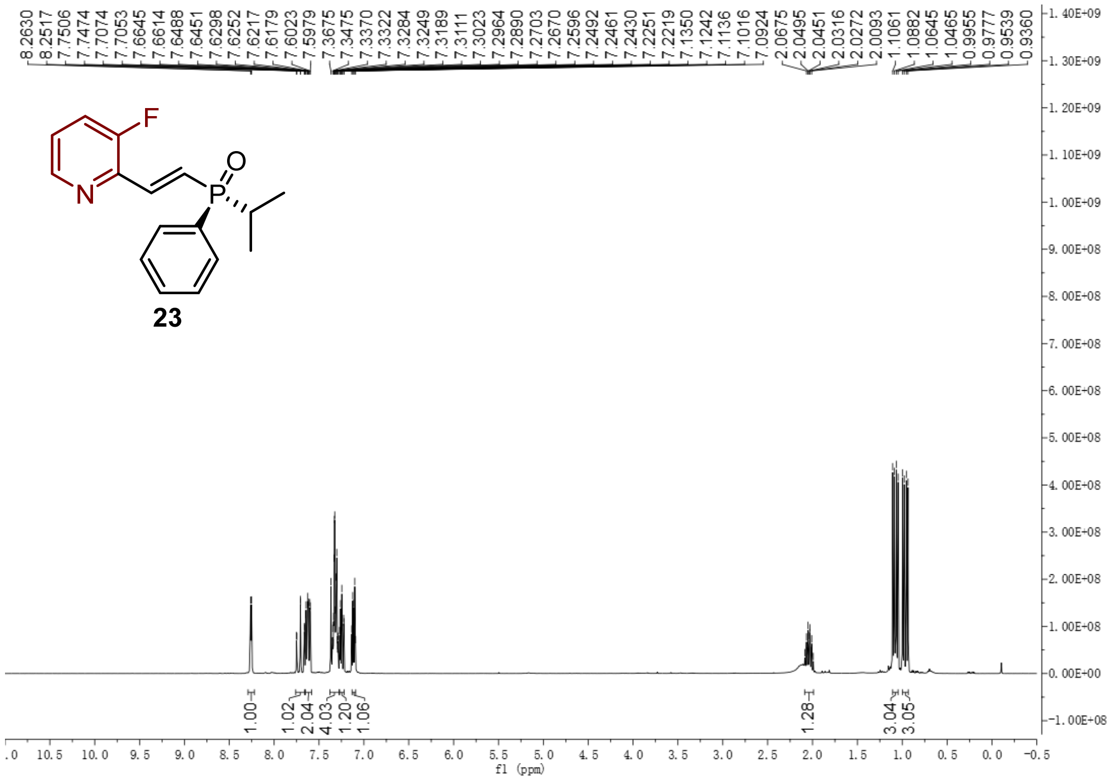
8.2630 8.2517 7.7506 7.7474 7.7074 7.7053 7.6645 7.6614 7.6488 7.6451 7.6298 7.6252 7.6217 7.6179 7.6023 7.5979 7.3675 7.3475 7.3370 7.3322 7.3284 7.3249 7.3189 7.3111 7.3023 7.2964 7.2890 7.2703 7.2670 7.2596 7.2492 7.2461 7.2430 7.2251 7.2219 7.1350 7.1242 7.1136 7.1016 7.0924 2.0675 2.0495 2.0451 2.0316 2.0272 2.0093 1.1061 1.0882 1.0645 1.0465 0.9955 0.9777 0.9539 0.9360
F
O
N
P
23
1.00
1.02
2.04
4.03
1.20
1.06
1.28
3.04
3.05
f1 (ppm)

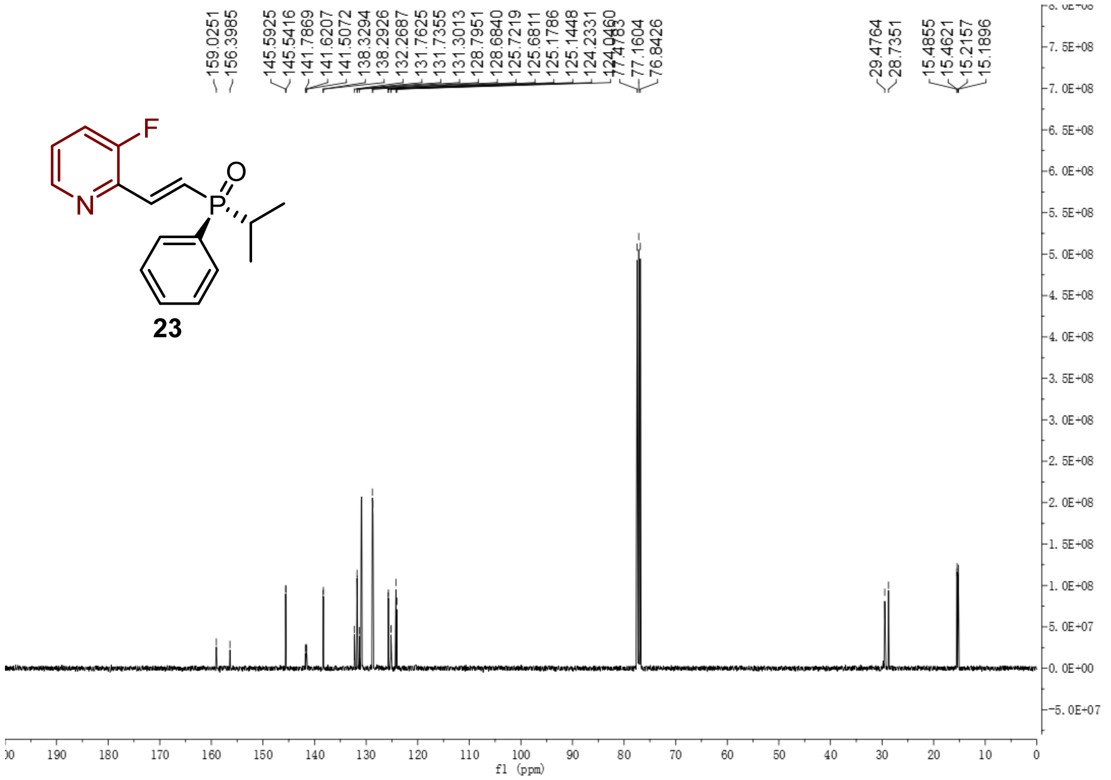
159.0251 156.3985 145.5925 145.5416 141.7869 141.6207 141.5072 138.3294 138.2926 132.2687 131.7625 131.7355 131.3013 128.7951 128.6840 125.7219 125.6811 125.1786 125.1448 124.2331 124.0460 77.4783 77.1604 76.8426 29.4764 28.7351 15.4855 15.4621 15.2157 15.1896
F
O
N
P
23
f1 (ppm)

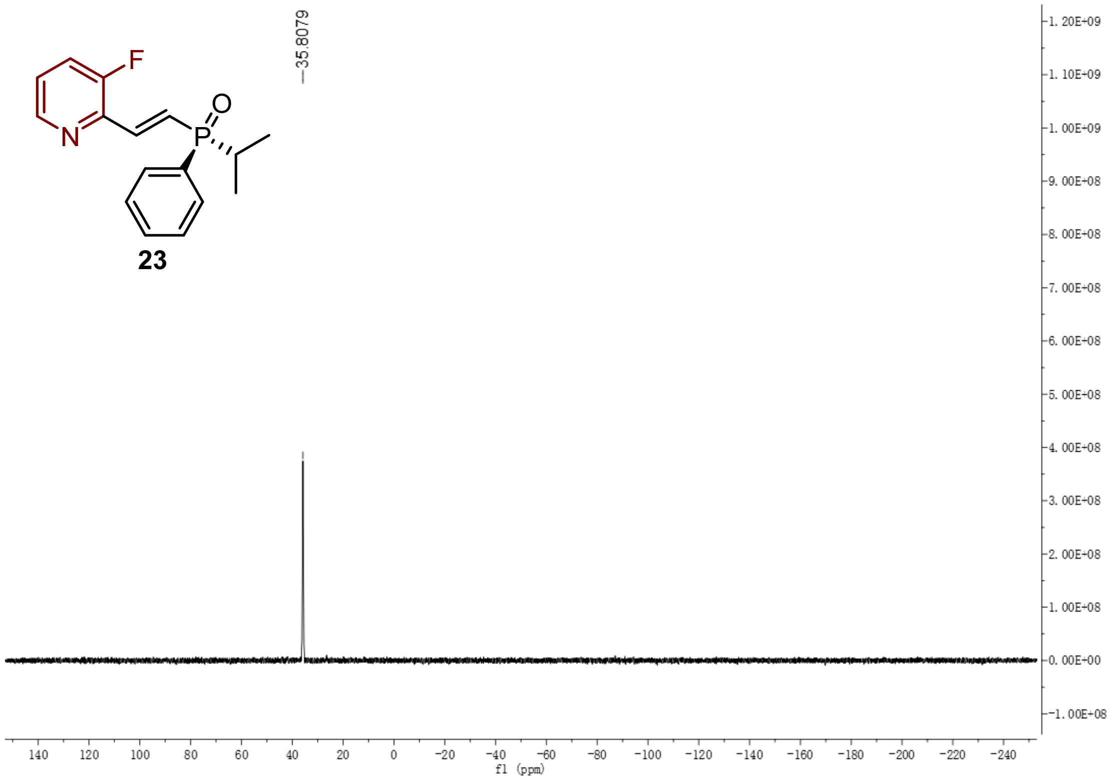

F
N
O
P
23
—35.8079
f1 (ppm)

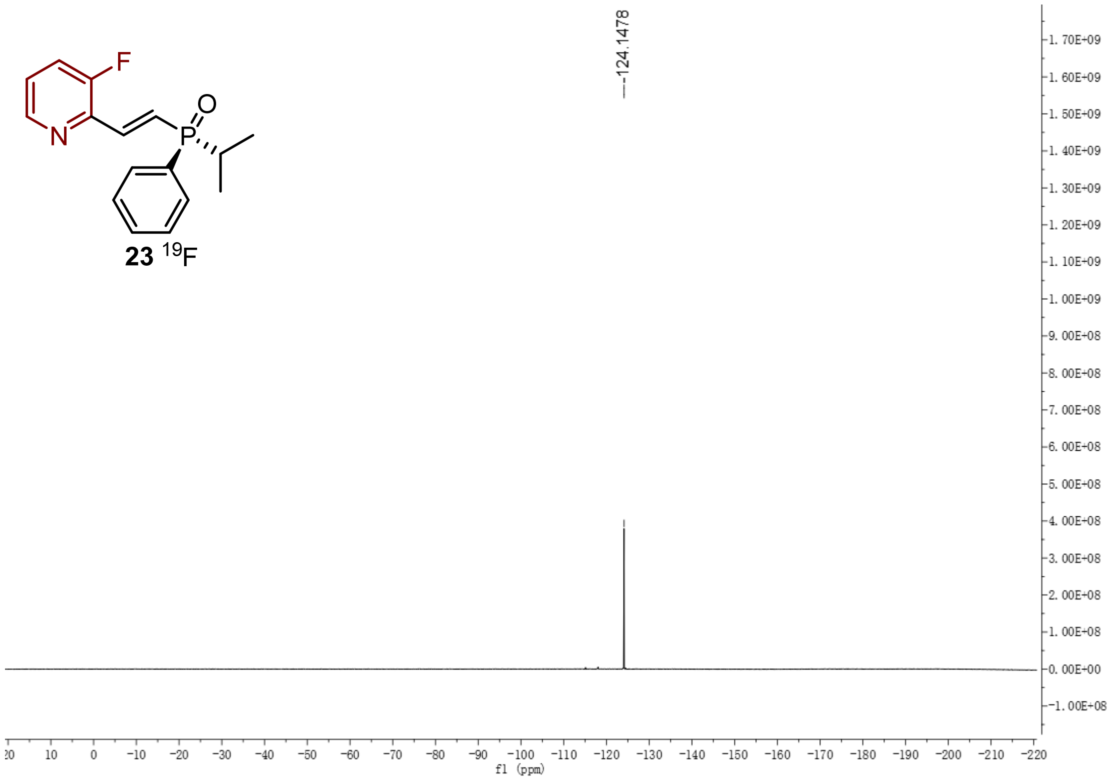

F
N
O
P
23 19F
—-124.1478
f1 (ppm)

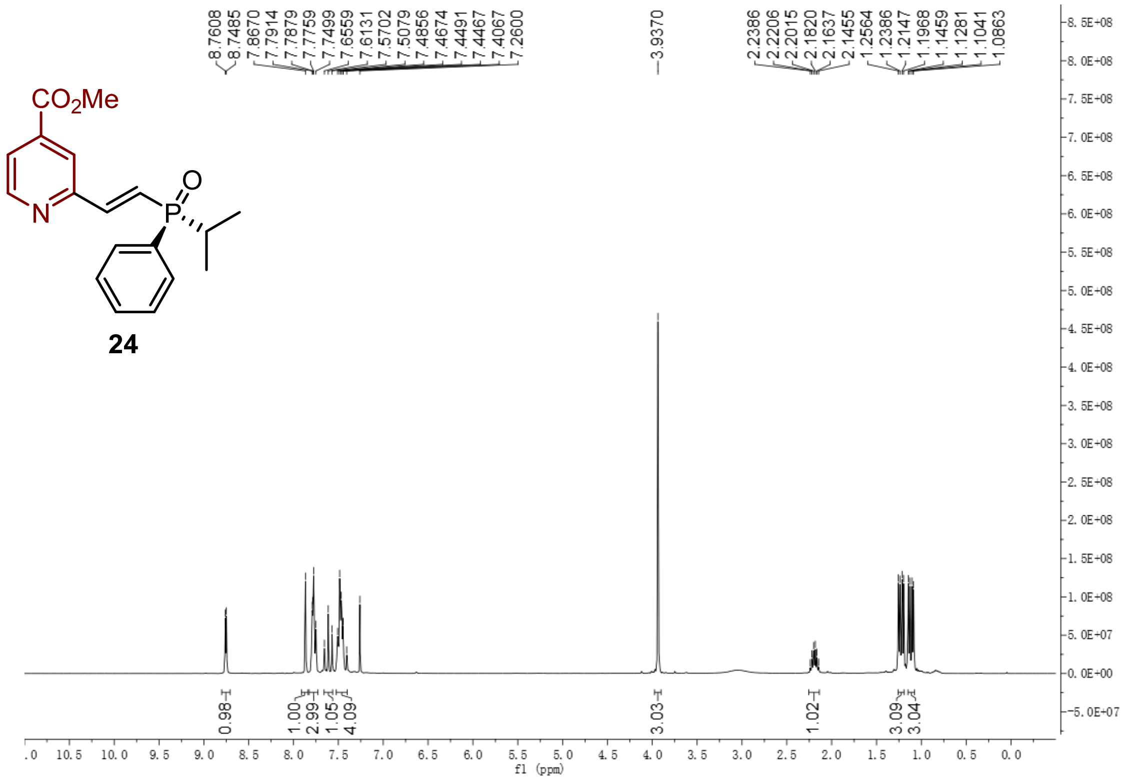

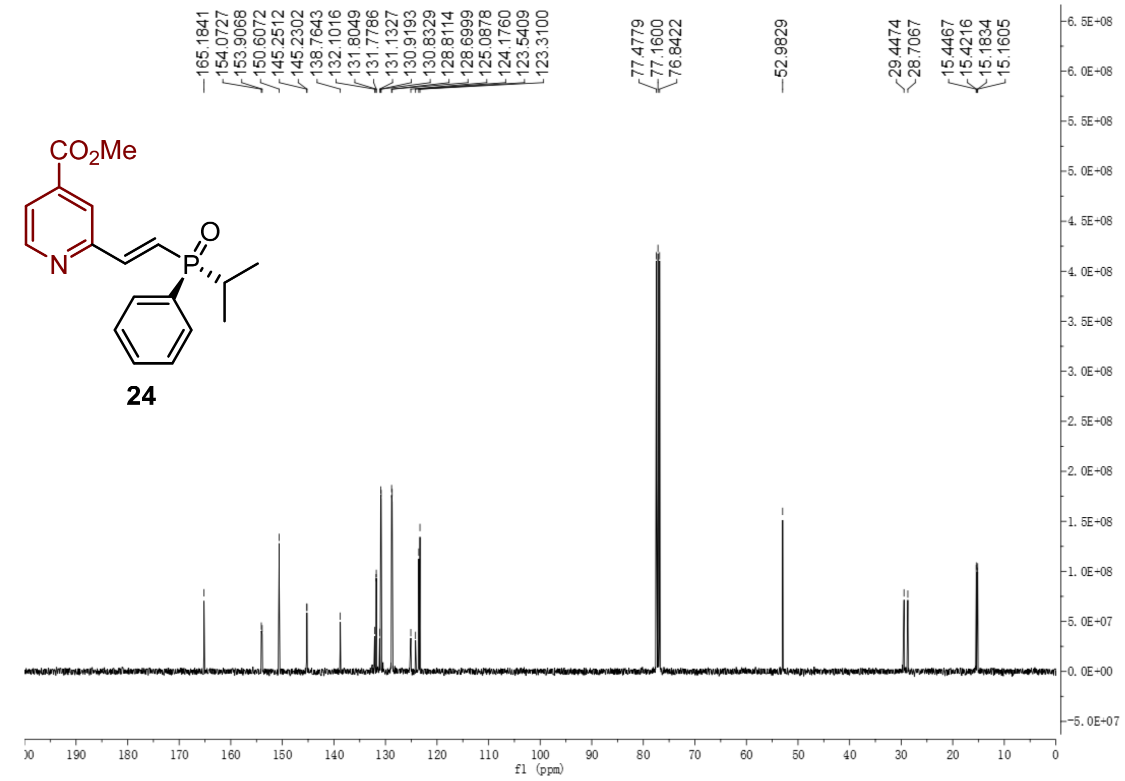

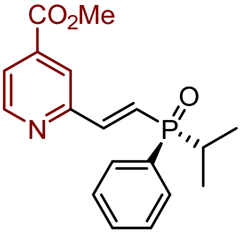
CO2Me
N
P
O

24

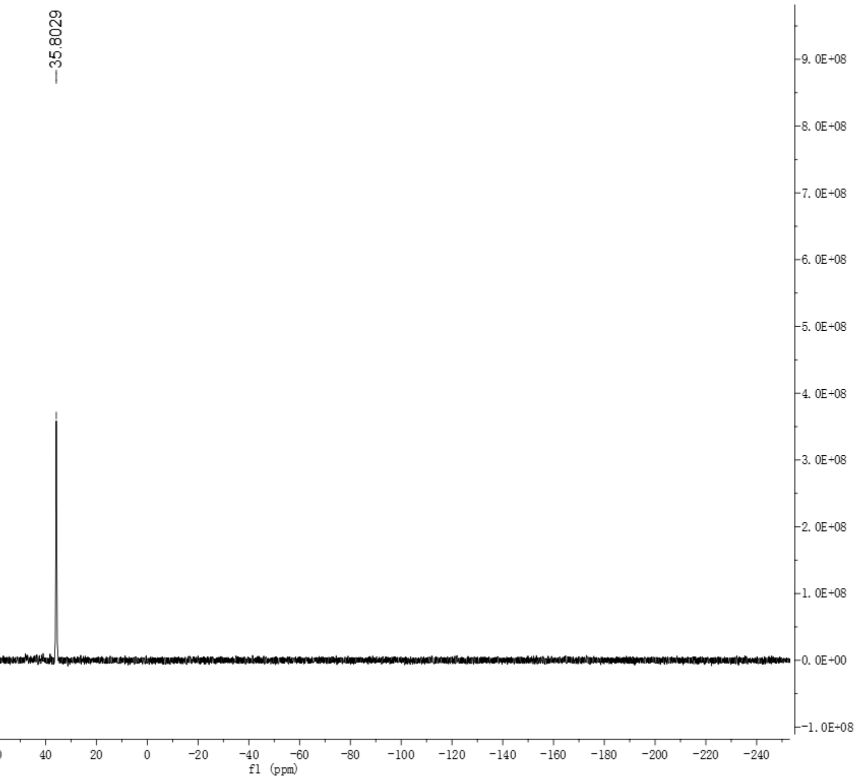
35.8029
f1 (ppm)

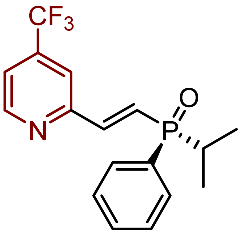
CF3
N
P
O

25

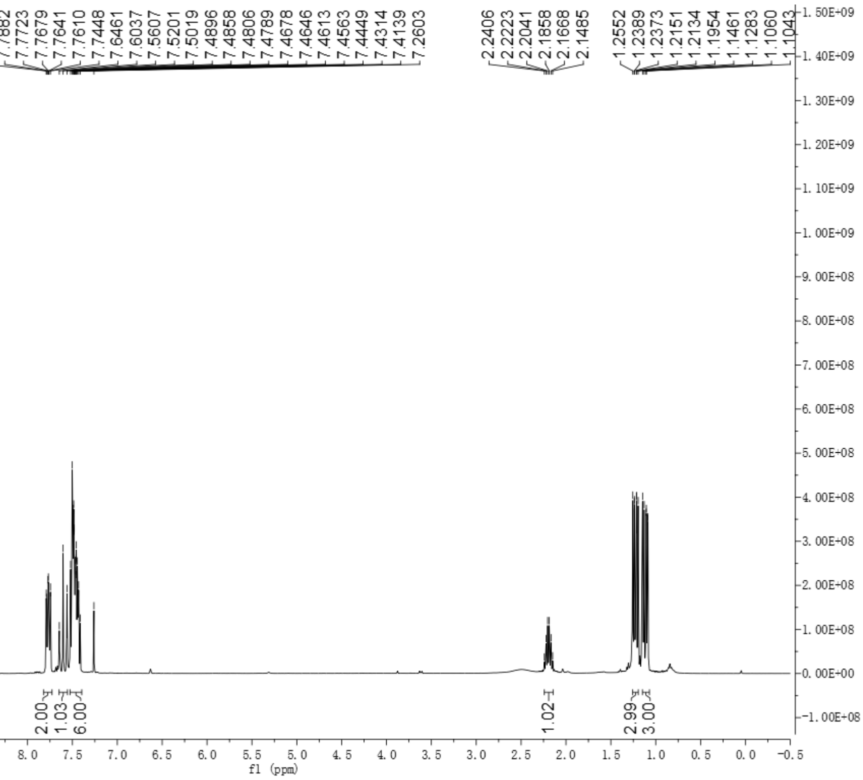
8.7860
8.7736
7.7913
7.7882
7.7723
7.7679
7.7641
7.7610
7.7448
7.6461
7.6037
7.5607
7.5201
7.5019
7.4896
7.4858
7.4806
7.4789
7.4678
7.4646
7.4613
7.4563
7.4449
7.4314
7.4139
7.2603
2.2406
2.2223
2.2041
2.1858
2.1668
2.1485
1.2552
1.2389
1.2373
1.2151
1.2134
1.1954
1.1461
1.1283
1.1060
1.1043
0.93
2.00
1.03
6.00
1.02
2.99
3.00
f1 (ppm)

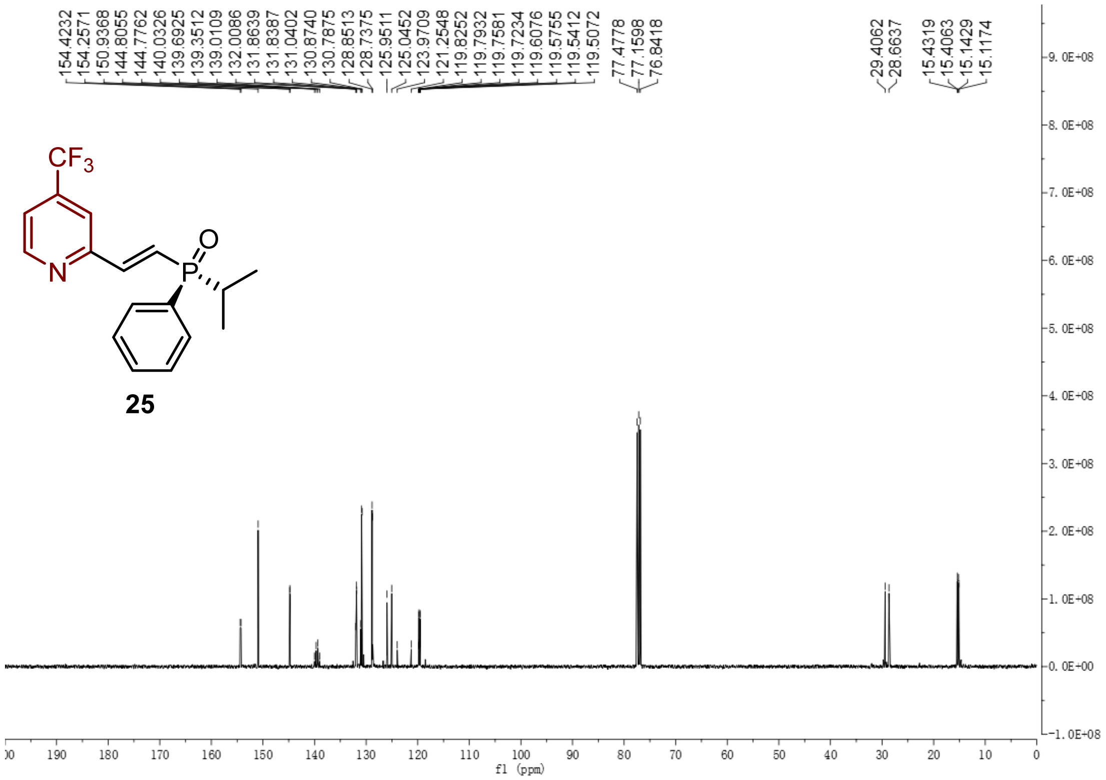
154.4232
154.2571
150.9368
144.8055
144.7762
140.0326
139.6925
139.3512
139.0109
132.0086
131.8639
131.8387
131.0402
130.8740
130.7875
128.8513
128.7375
125.9511
125.0452
123.9709
121.2548
119.8252
119.7932
119.7581
119.7234
119.6076
119.5755
119.5412
119.5072
77.4778
77.1598
76.8418
29.4062
28.6637
15.4319
15.4063
15.1429
15.1174
CF3
N
O
P
25
f1 (ppm)

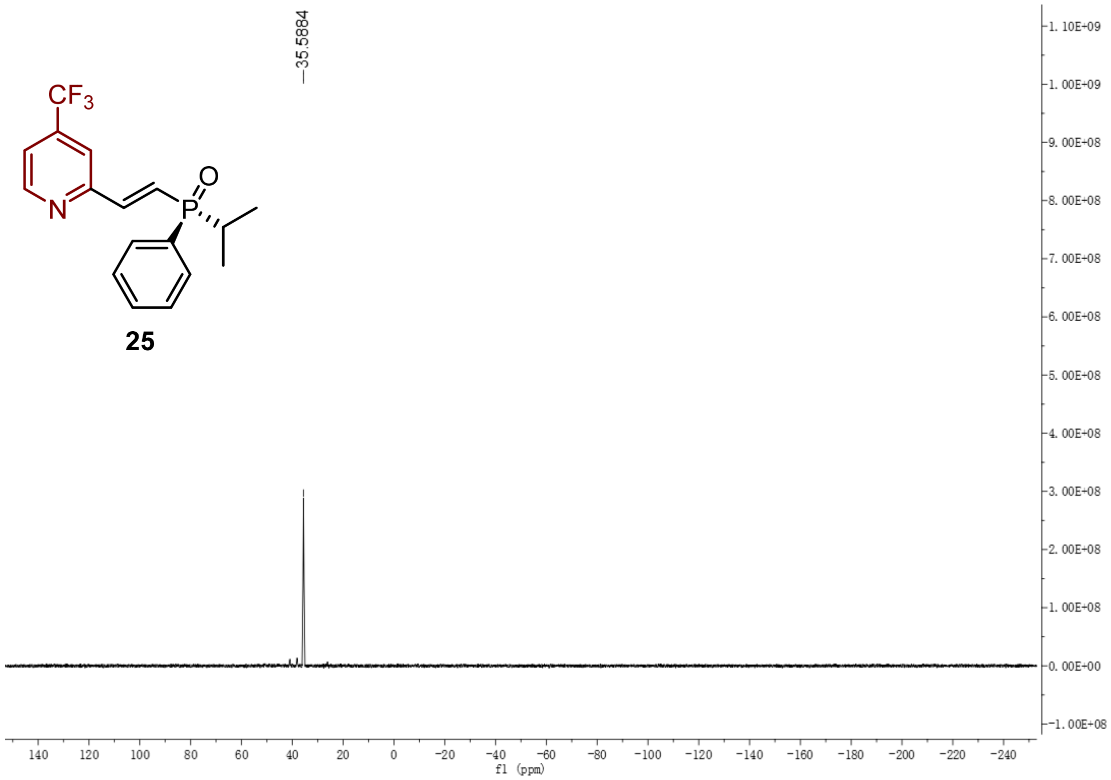
35.5884
CF3
N
O
P
25
f1 (ppm)

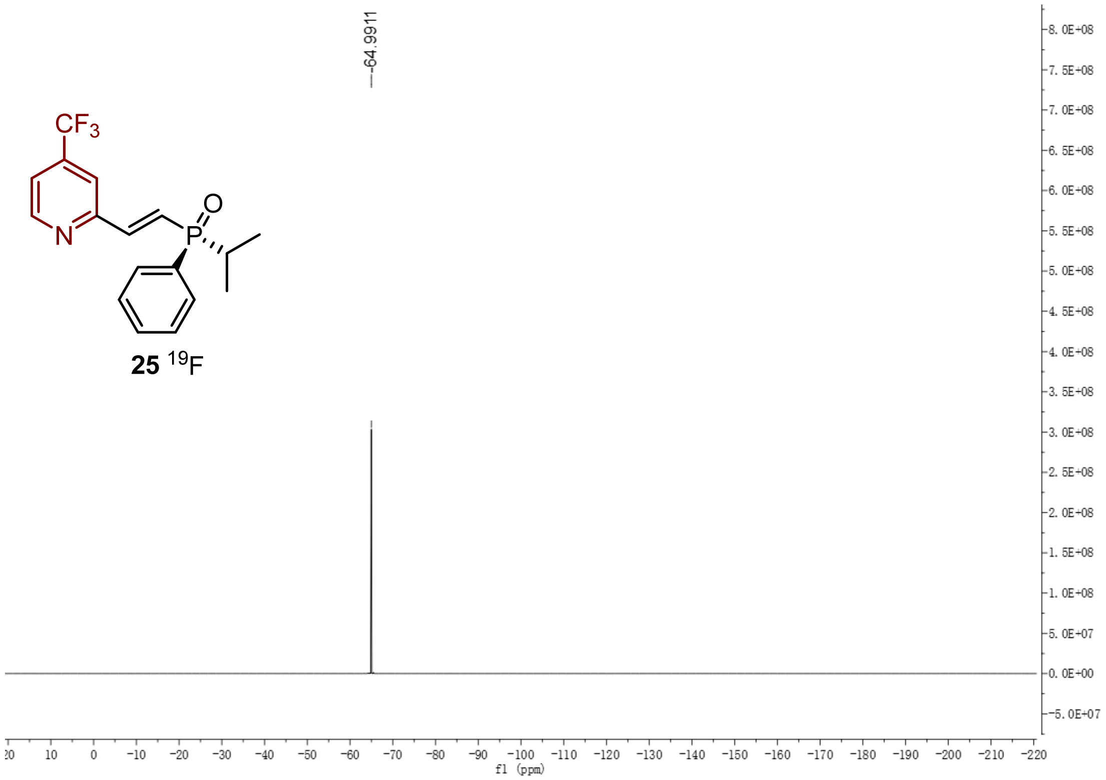
-64.9911
CF3
N
O
P
25 19F
f1 (ppm)

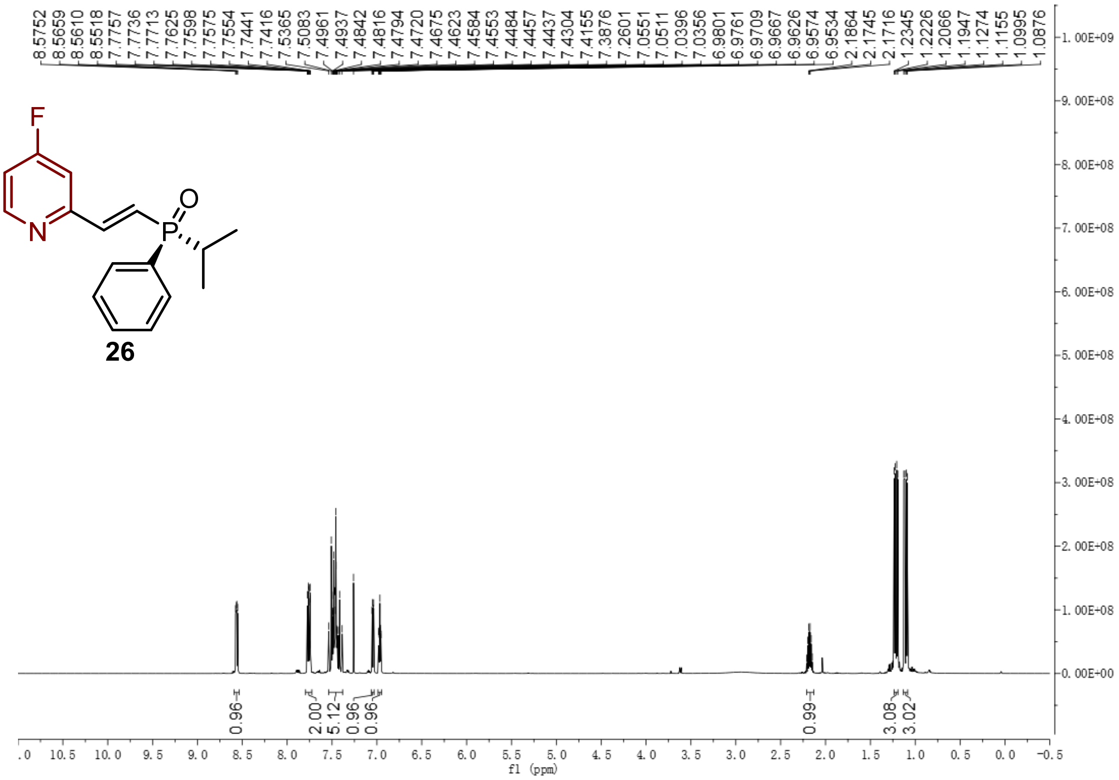
8.5752
8.5659
8.5610
8.5518
7.7757
7.7736
7.7713
7.7625
7.7598
7.7575
7.7554
7.7441
7.7416
7.5365
7.5083
7.4961
7.4937
7.4842
7.4816
7.4794
7.4720
7.4675
7.4623
7.4584
7.4553
7.4484
7.4457
7.4437
7.4304
7.4155
7.3876
7.2601
7.0551
7.0511
7.0396
7.0356
6.9801
6.9761
6.9709
6.9667
6.9626
6.9574
6.9534
2.1864
2.1745
2.1716
1.2345
1.2226
1.2066
1.1947
1.1274
1.1155
1.0995
1.0876
F
N
O
P
26
0.96
2.00
5.12
0.96
0.96
0.99
3.08
3.02
f1 (ppm)

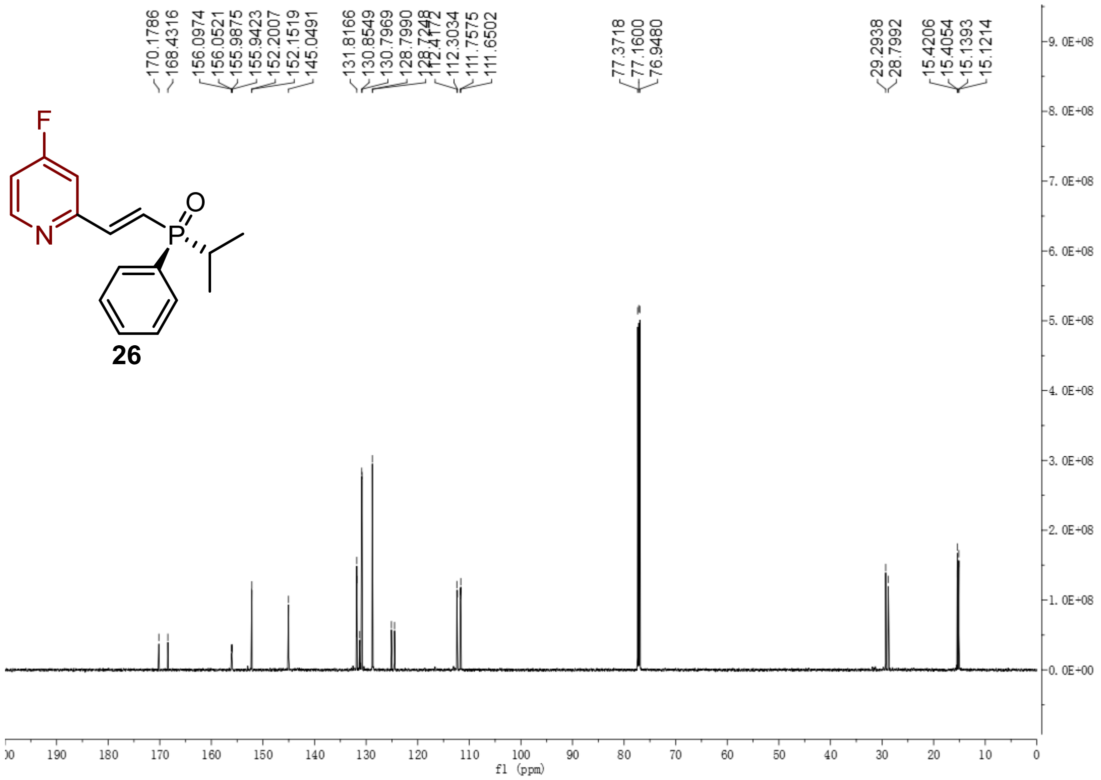

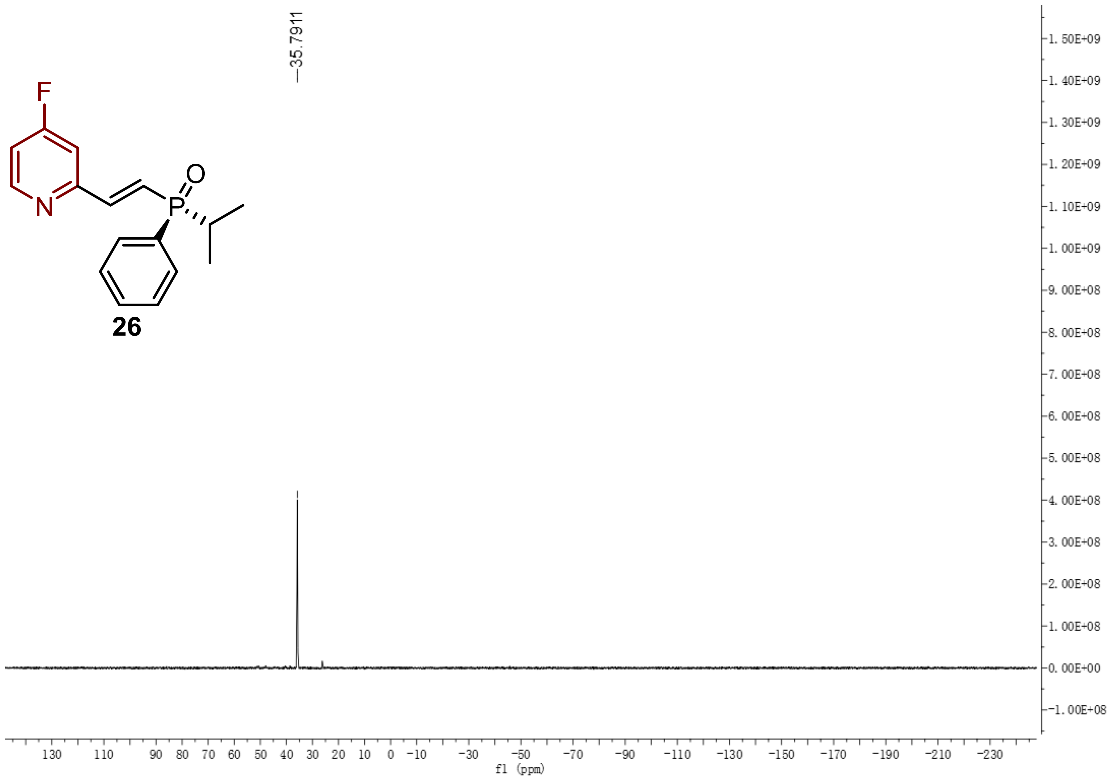

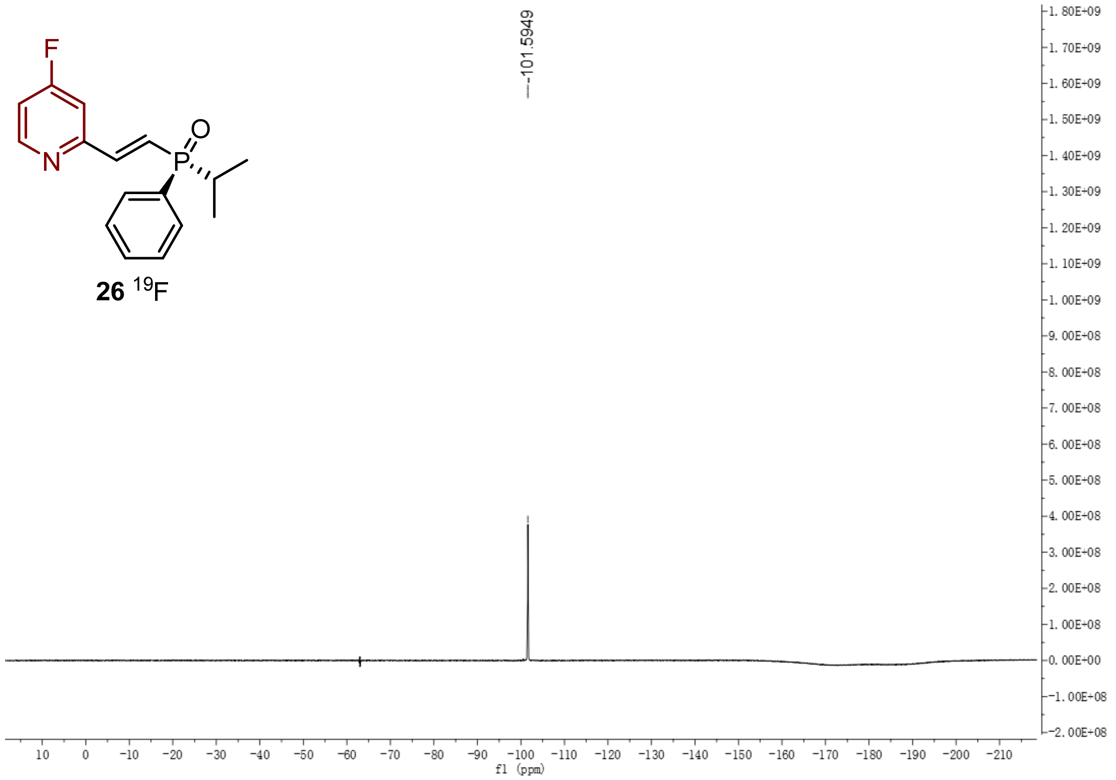

26 19F
-101.5949
f1 (ppm)

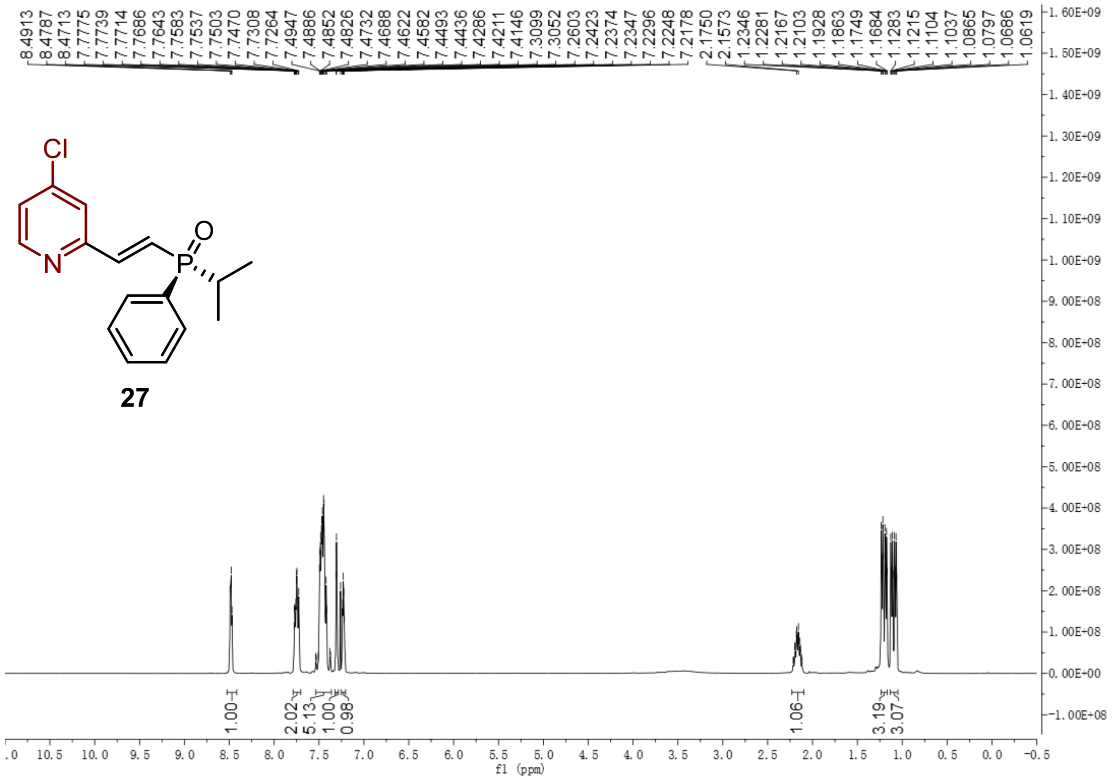

27
8.4913
8.4787
8.4713
7.7775
7.7739
7.7714
7.7686
7.7643
7.7583
7.7537
7.7503
7.7470
7.7308
7.7264
7.4947
7.4886
7.4852
7.4826
7.4732
7.4688
7.4622
7.4582
7.4493
7.4436
7.4286
7.4211
7.4146
7.3099
7.3052
7.2603
7.2423
7.2374
7.2347
7.2296
7.2248
7.2178
2.1750
2.1573
1.2346
1.2281
1.2167
1.2103
1.1928
1.1863
1.1749
1.1684
1.1283
1.1215
1.1104
1.1037
1.0865
1.0797
1.0686
1.0619
1.00
2.02
5.13
1.00
0.98
1.06
3.19
3.07
f1 (ppm)

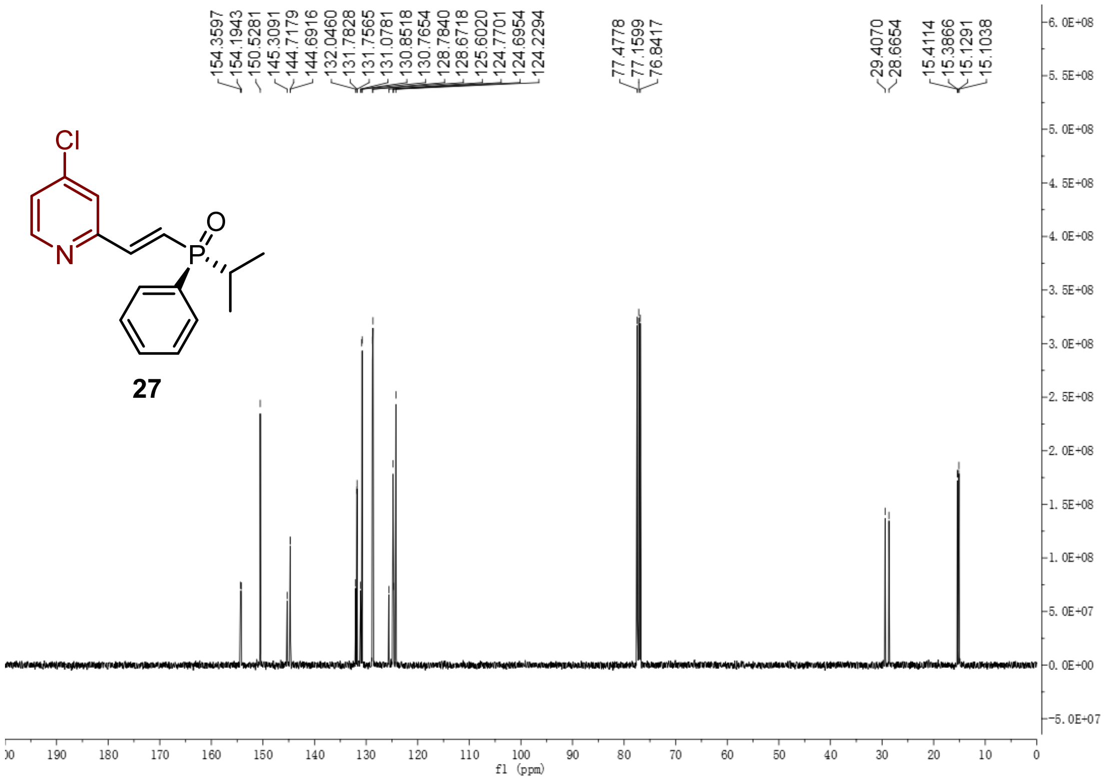
154.3597
154.1943
150.5281
145.3091
144.7179
144.6916
132.0460
131.7828
131.7565
131.0781
130.8518
130.7654
128.7840
128.6718
125.6020
124.7701
124.6954
124.2294
77.4778
77.1599
76.8417
29.4070
28.6654
15.4114
15.3866
15.1291
15.1038
Cl
N
P
O
27
f1 (ppm)

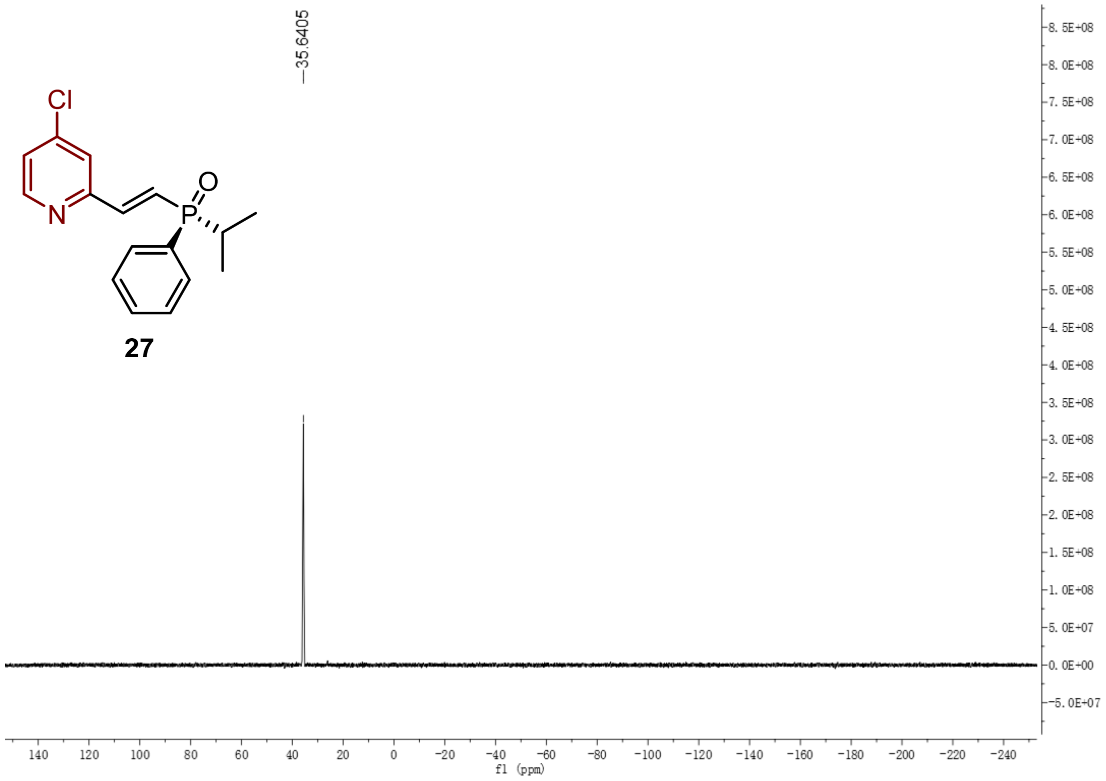
35.6405
Cl
N
P
O
27
f1 (ppm)

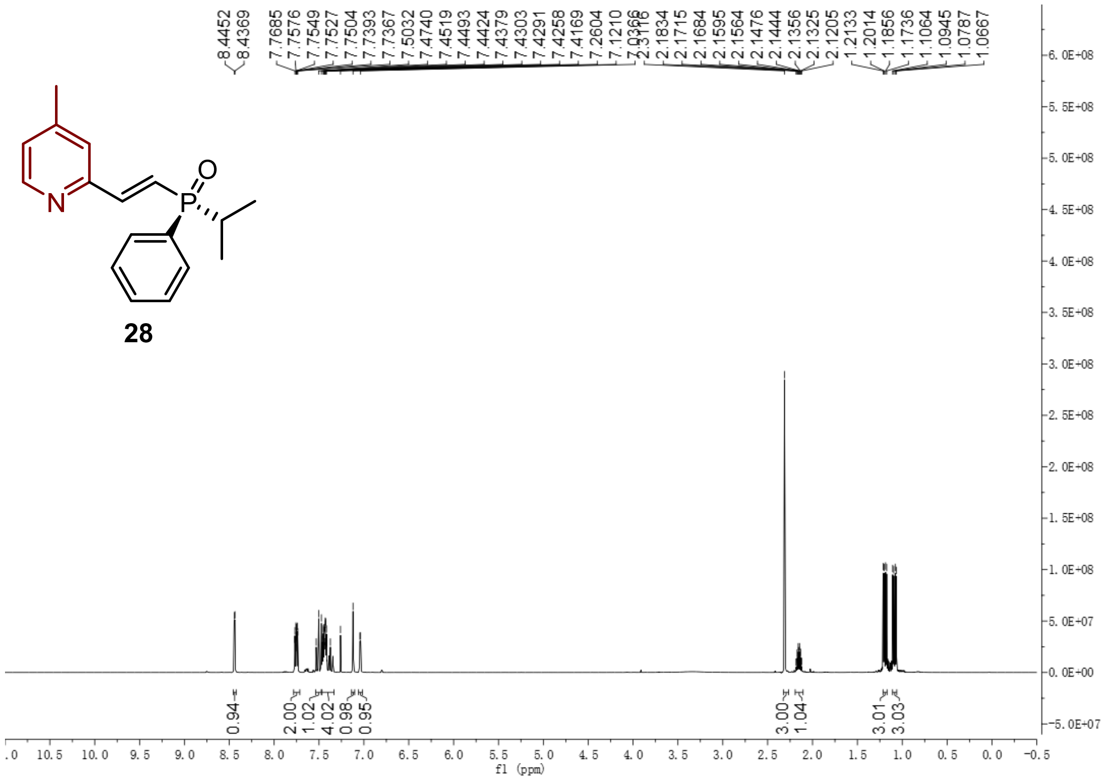
8.4452
8.4369
7.7685
7.7576
7.7549
7.7527
7.7504
7.7393
7.7367
7.5032
7.4740
7.4519
7.4493
7.4424
7.4379
7.4303
7.4291
7.4258
7.4169
7.2604
7.1210
7.0366
2.3116
2.1834
2.1715
2.1684
2.1595
2.1564
2.1476
2.1444
2.1356
2.1325
2.1205
1.2133
1.2014
1.1856
1.1736
1.1064
1.0945
1.0787
1.0667
0.94
2.00
1.02
4.02
0.98
0.95
3.00
1.04
3.01
3.03
fl (ppm)

28

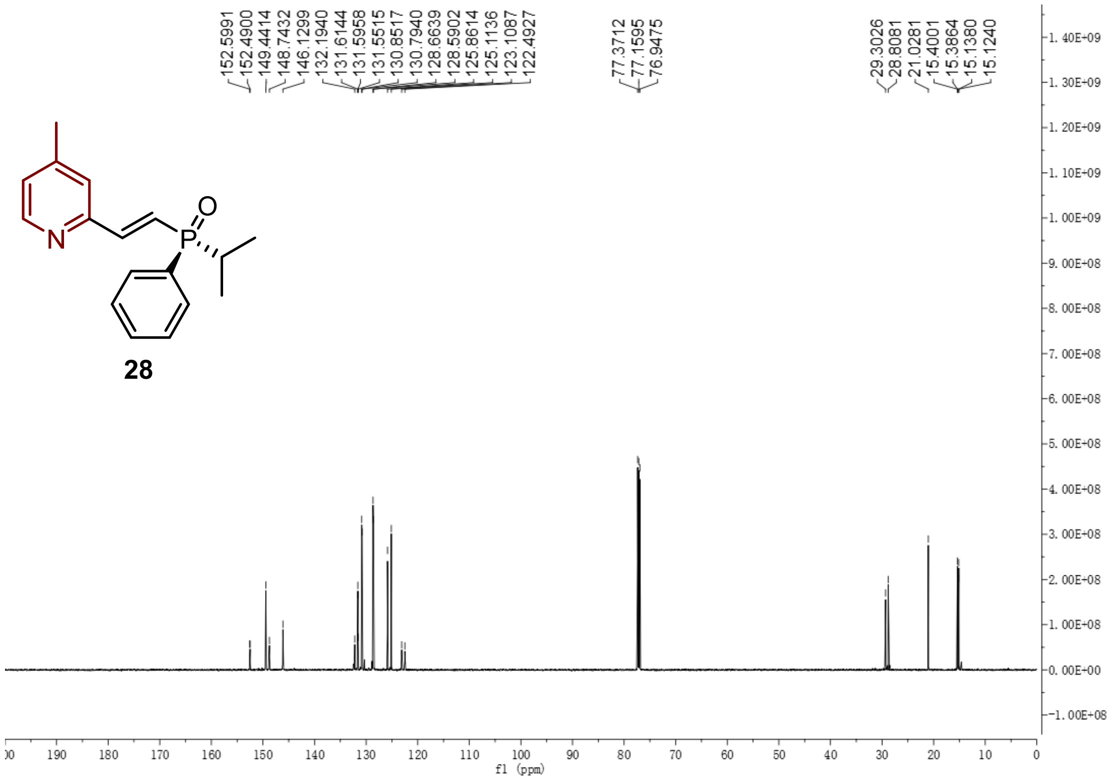
152.5991
152.4900
149.4414
148.7432
146.1299
132.1940
131.6144
131.5958
131.5515
130.8517
130.7940
128.6639
128.5902
125.8614
125.1136
123.1087
122.4927
77.3712
77.1595
76.9475
29.3026
28.8081
21.0281
15.4001
15.3864
15.1380
15.1240
fl (ppm)

28

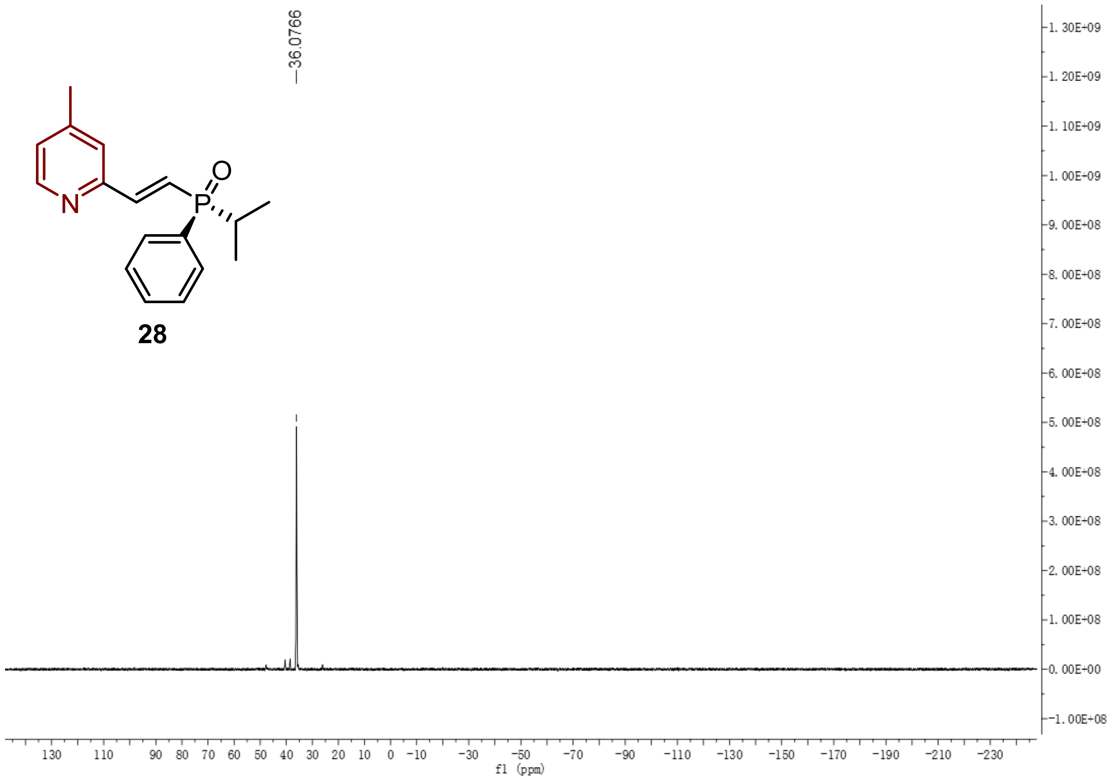

O
P
N
—36.0766
f1 (ppm)

28

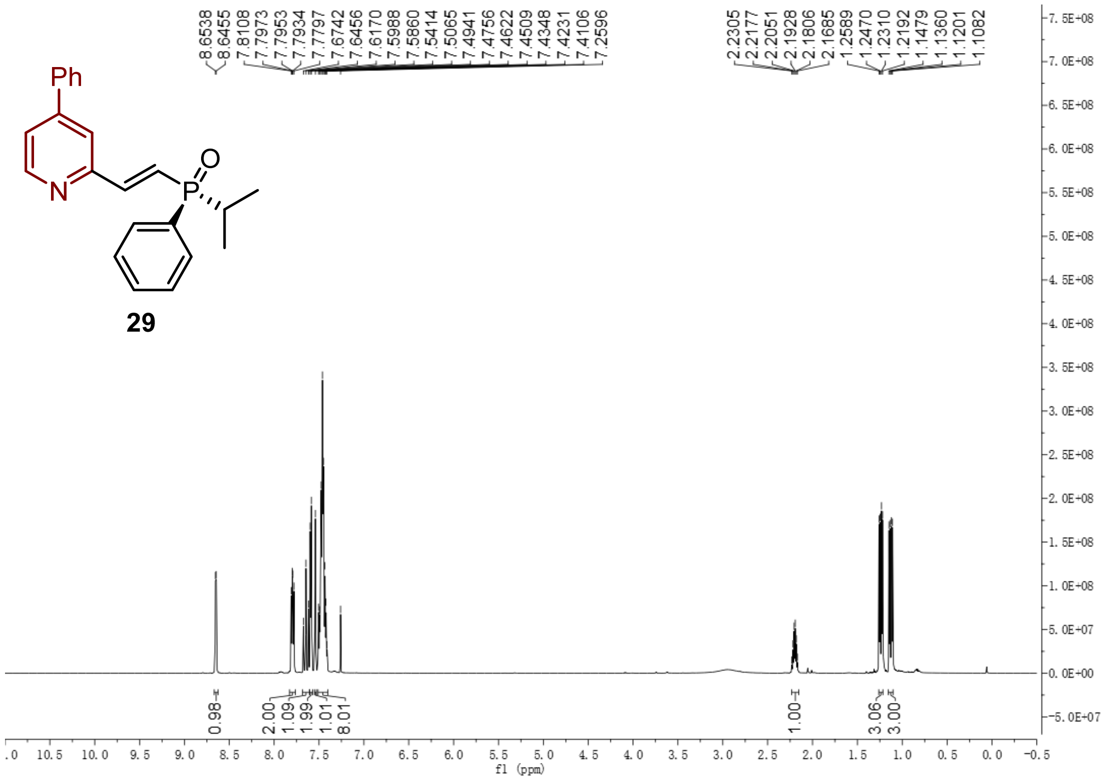

Ph
O
P
N
8.6538
8.6455
7.8108
7.7973
7.7953
7.7934
7.7797
7.6742
7.6456
7.6170
7.5988
7.5860
7.5414
7.5065
7.4941
7.4756
7.4622
7.4509
7.4348
7.4231
7.4106
7.2596
2.2305
2.2177
2.2051
2.1928
2.1806
2.1685
1.2589
1.2470
1.2310
1.2192
1.1479
1.1360
1.1201
1.1082
0.98
2.00
1.09
1.99
1.01
8.01
1.00
3.06
3.00
f1 (ppm)

29

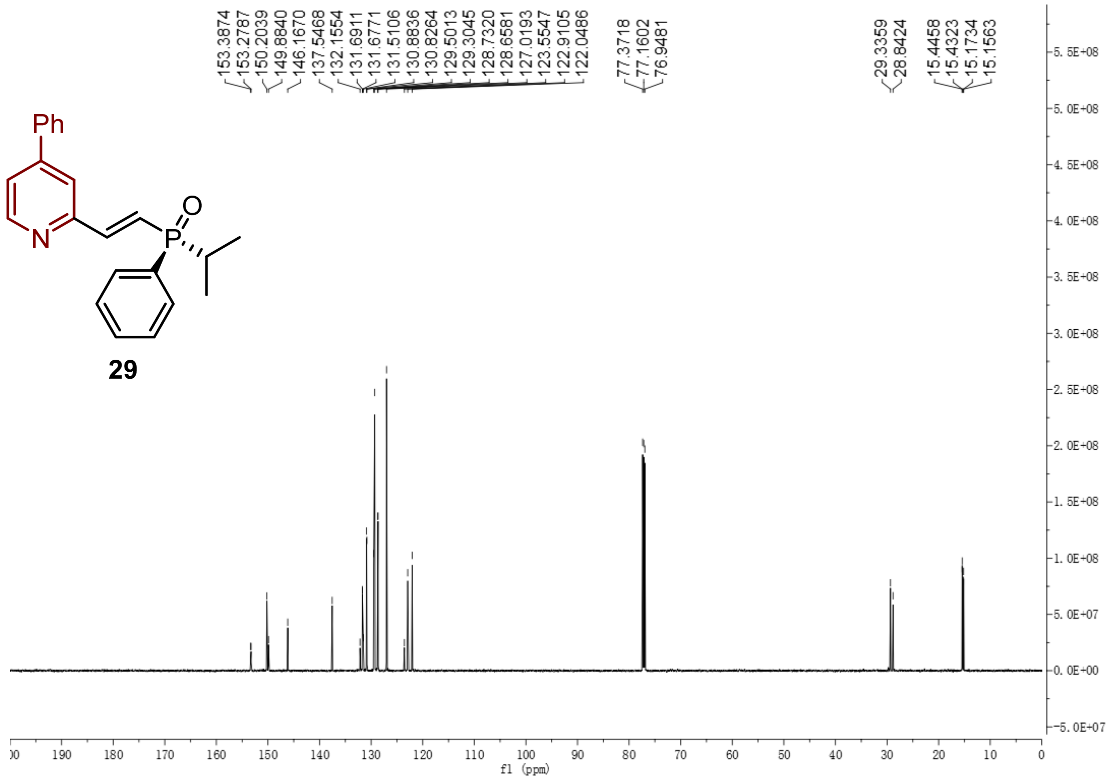

29

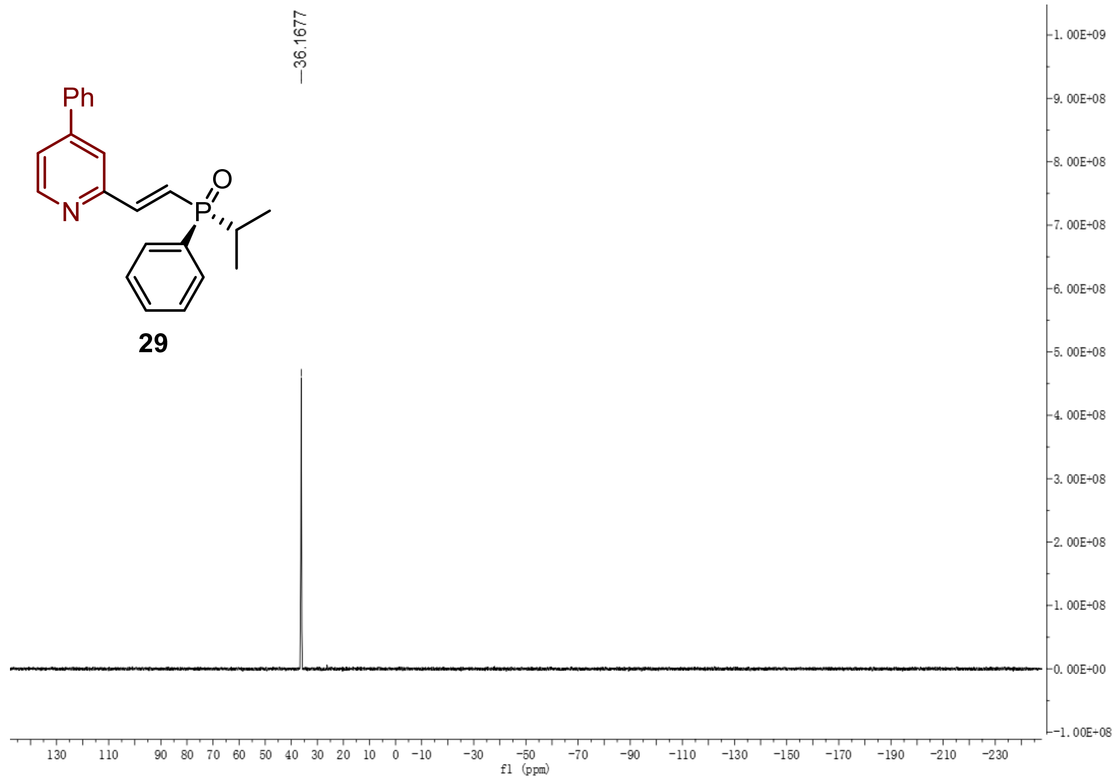

29

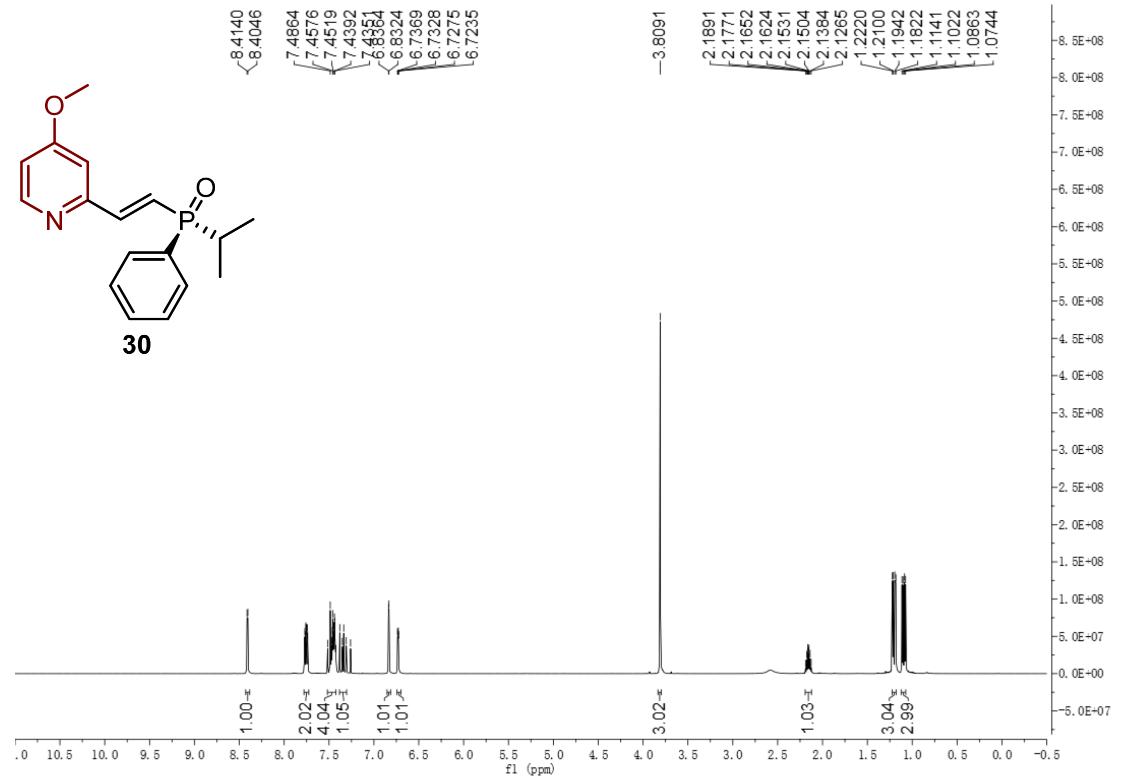
30

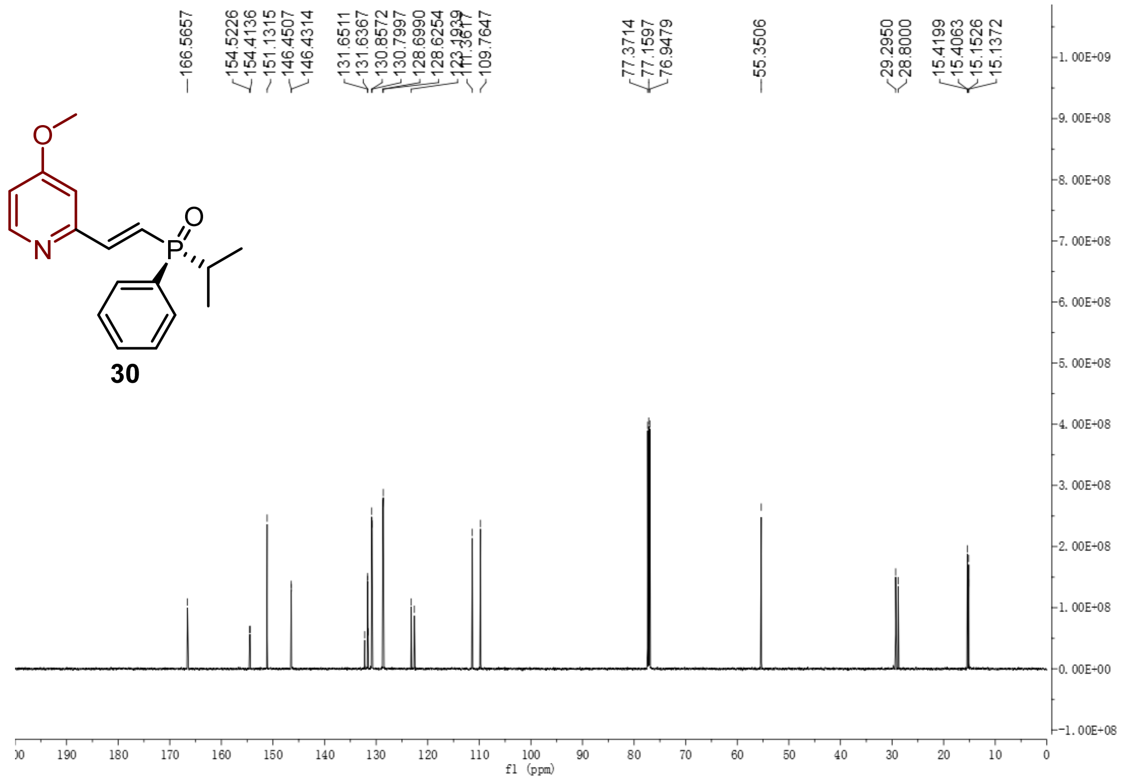
30

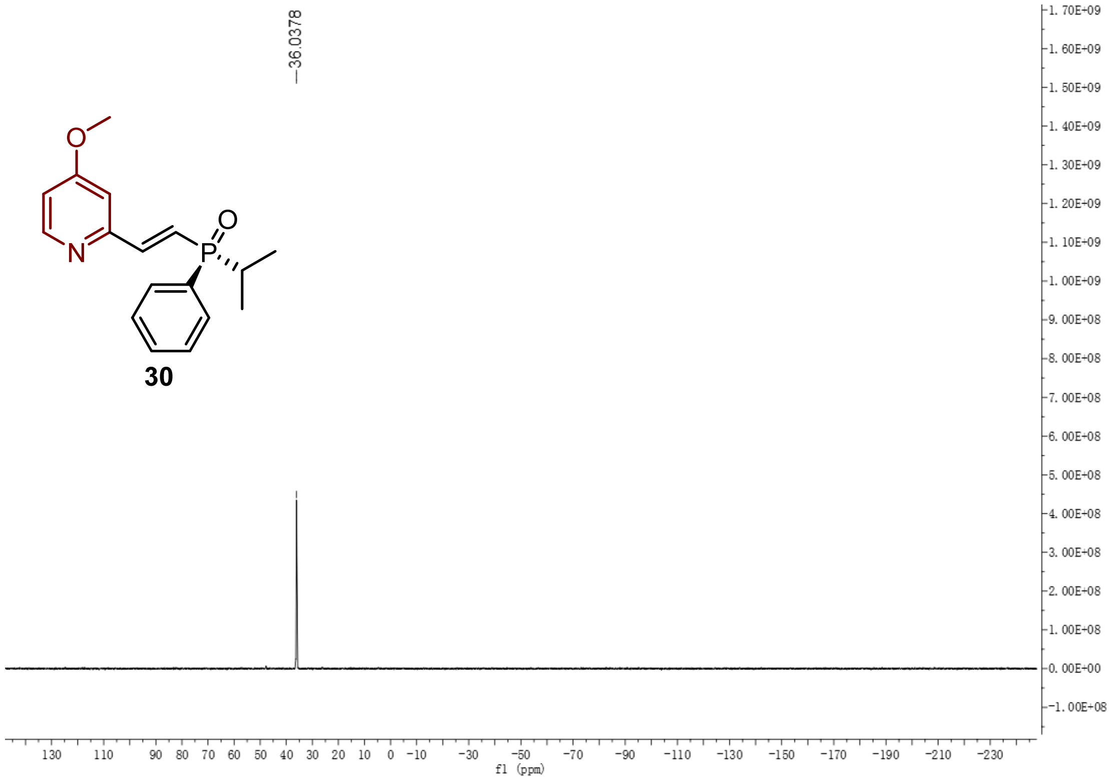

36.0378
30
f1 (ppm)

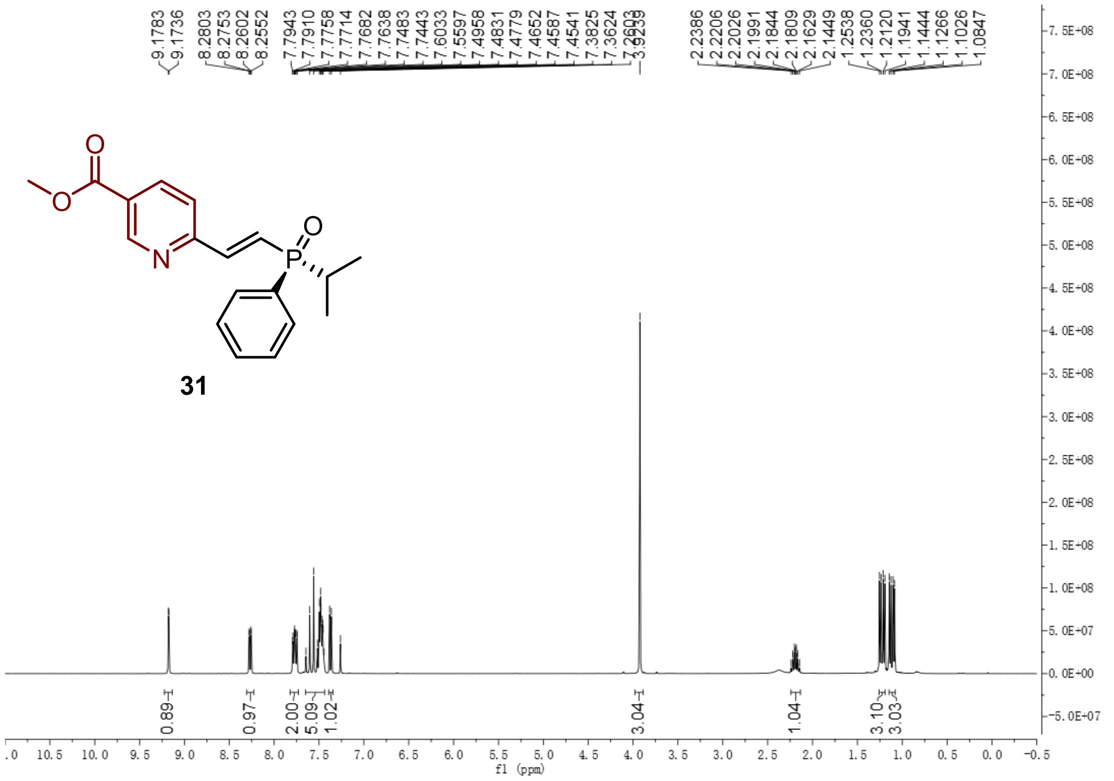

9.1783
9.1736
8.2803
8.2753
8.2602
8.2552
7.7943
7.7910
7.7758
7.7714
7.7682
7.7638
7.7483
7.7443
7.6033
7.5597
7.4958
7.4831
7.4779
7.4652
7.4587
7.4541
7.3825
7.3624
2.2386
2.2206
2.2026
2.1991
2.1844
2.1809
2.1629
2.1449
1.2538
1.2360
1.2120
1.1941
1.1444
1.1266
1.1026
1.0847
31
0.89
0.97
2.00
5.09
1.02
3.04
1.04
3.10
3.03
f1 (ppm)

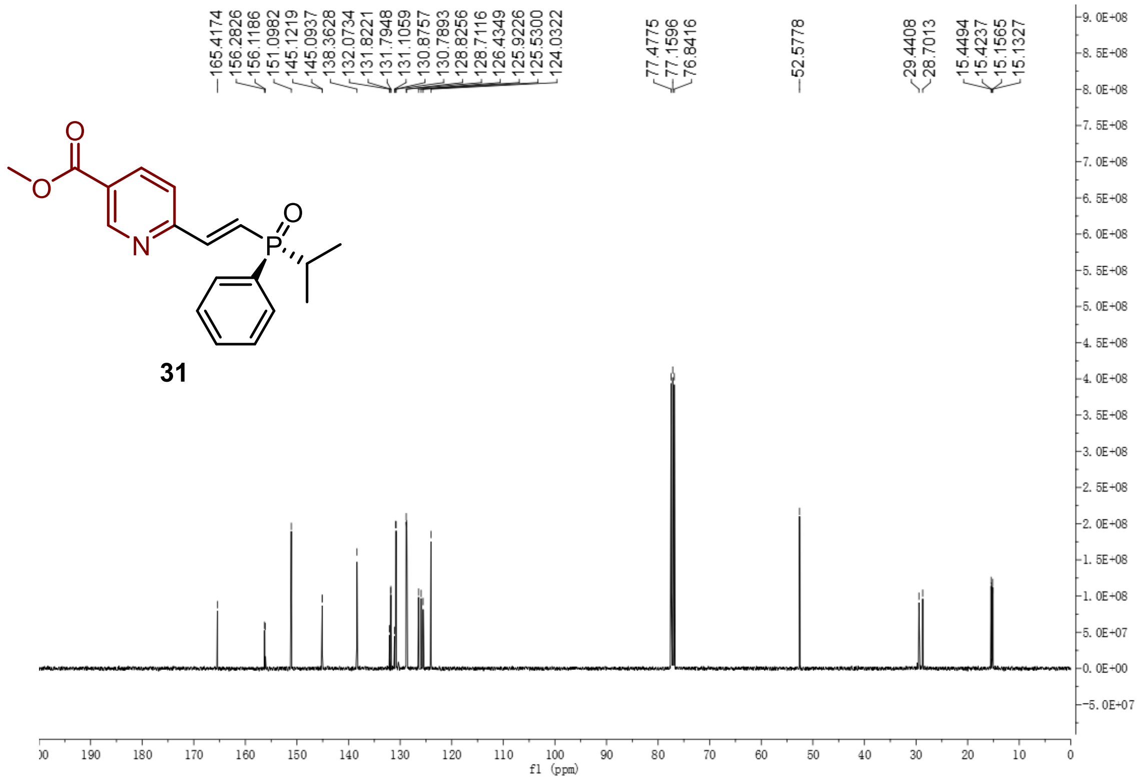
165.4174
156.2826
156.1186
151.0982
145.1219
145.0937
138.3628
132.0734
131.8221
131.7948
131.1059
130.8757
130.7893
128.8256
128.7116
126.4349
125.9226
125.5300
124.0322
77.4775
77.1596
76.8416
52.5778
29.4408
28.7013
15.4494
15.4237
15.1565
15.1327
31
f1 (ppm)

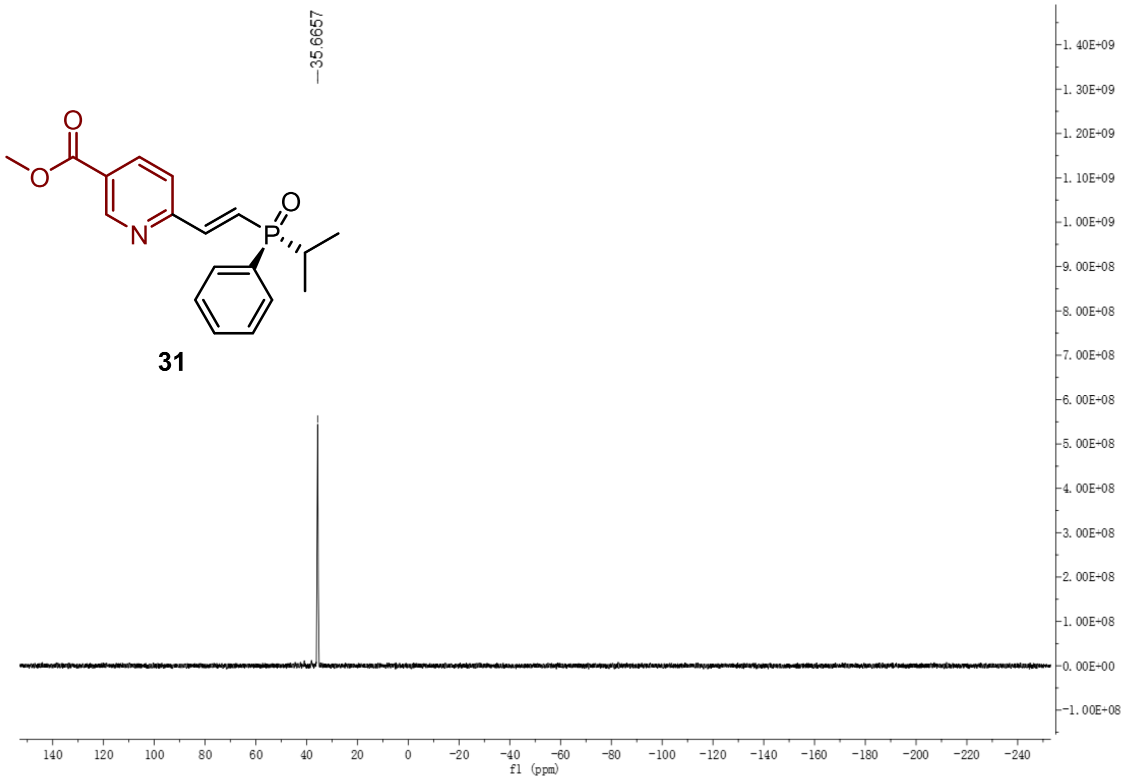
35.6657
31
f1 (ppm)

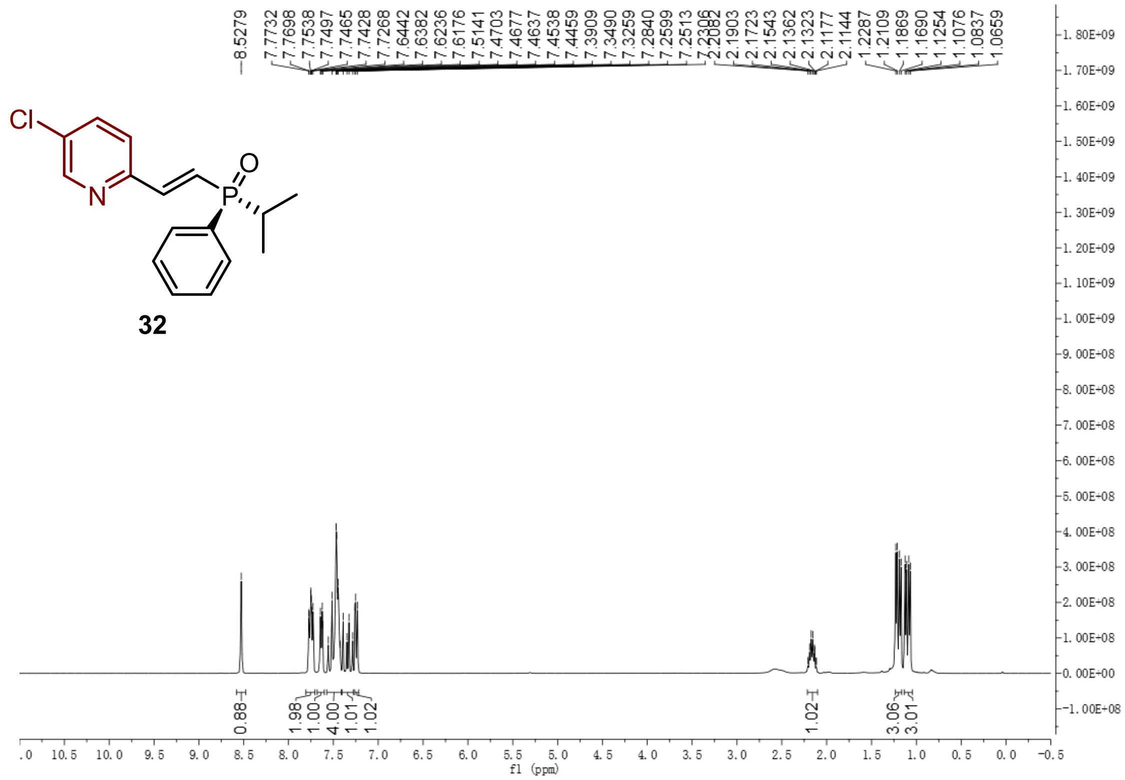
Cl
N
O
P
32
8.5279
7.7732
7.7698
7.7538
7.7497
7.7465
7.7428
7.7268
7.6442
7.6382
7.6236
7.6176
7.5141
7.4703
7.4677
7.4637
7.4538
7.4459
7.3909
7.3490
7.3259
7.2840
7.2599
7.2513
7.2306
7.2082
2.1903
2.1723
2.1543
2.1362
2.1323
2.1177
2.1144
1.2287
1.2109
1.1869
1.1690
1.1254
1.1076
1.0837
1.0659
0.88
1.98
1.00
4.00
1.01
1.02
1.02
3.06
3.01
f1 (ppm)

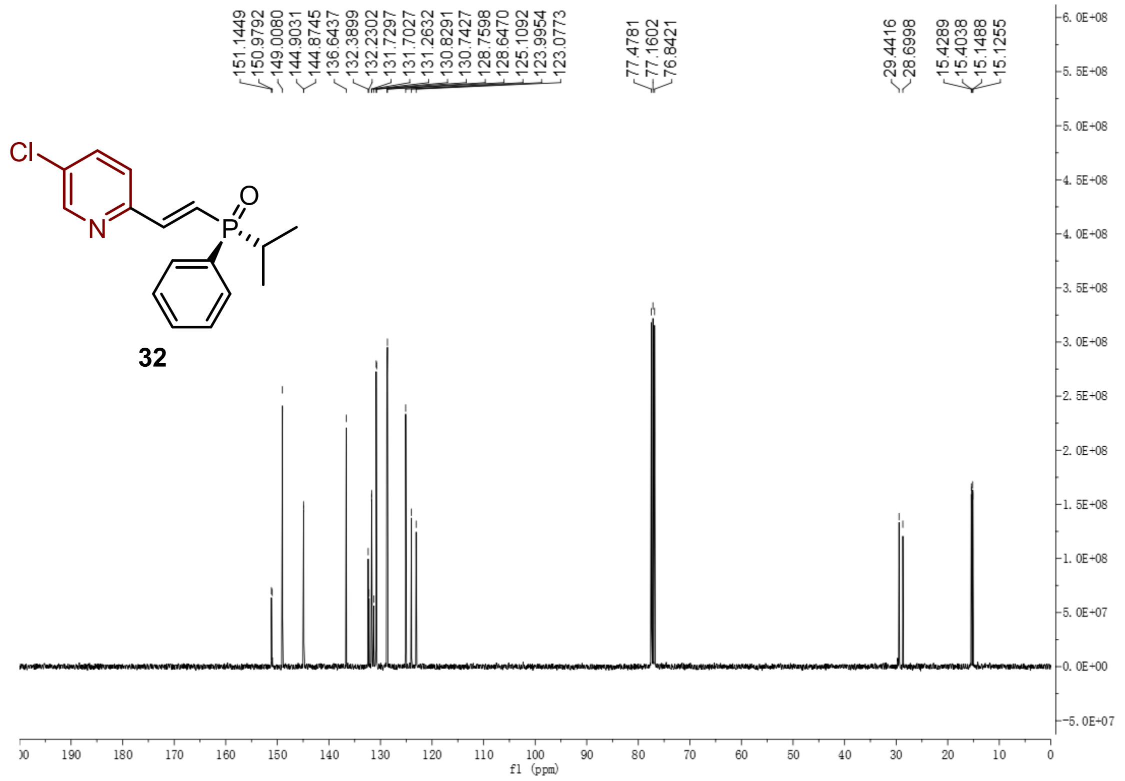
Cl
N
O
P
32
151.1449
150.9792
149.0080
144.9031
144.8745
136.6437
132.3899
132.2302
131.7297
131.7027
131.2632
130.8291
130.7427
128.7598
128.6470
125.1092
123.9954
123.0773
77.4781
77.1602
76.8421
29.4416
28.6998
15.4289
15.4038
15.1488
15.1255
f1 (ppm)

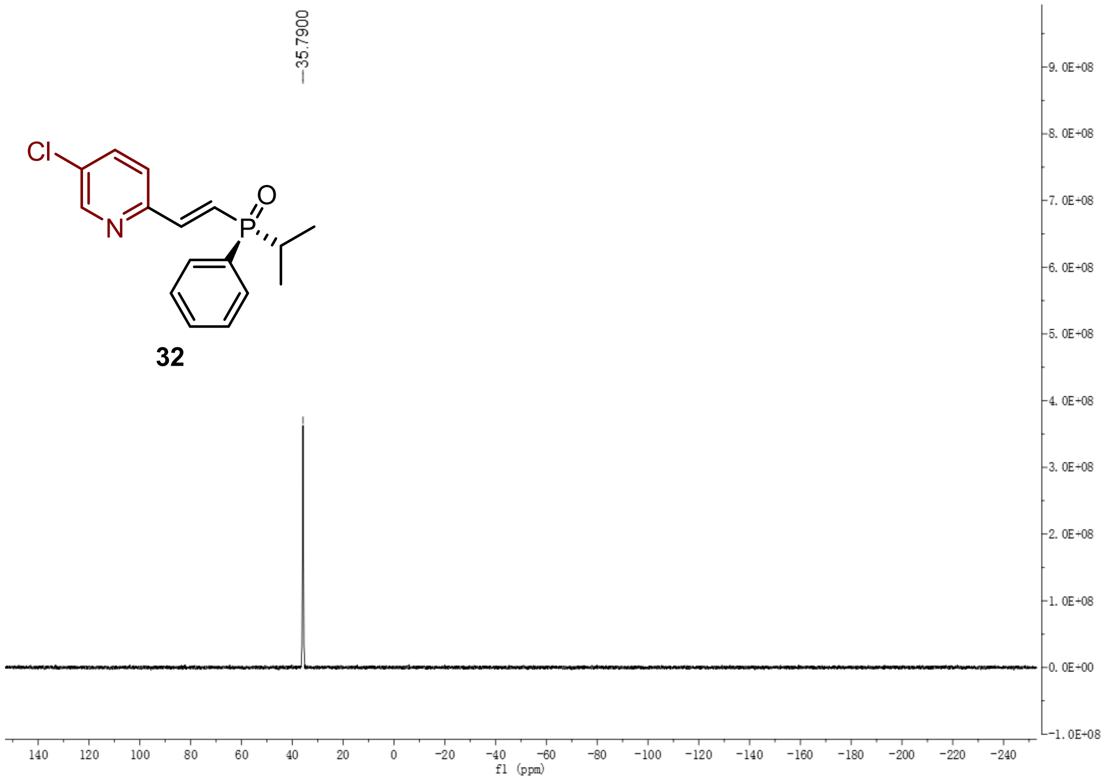

32

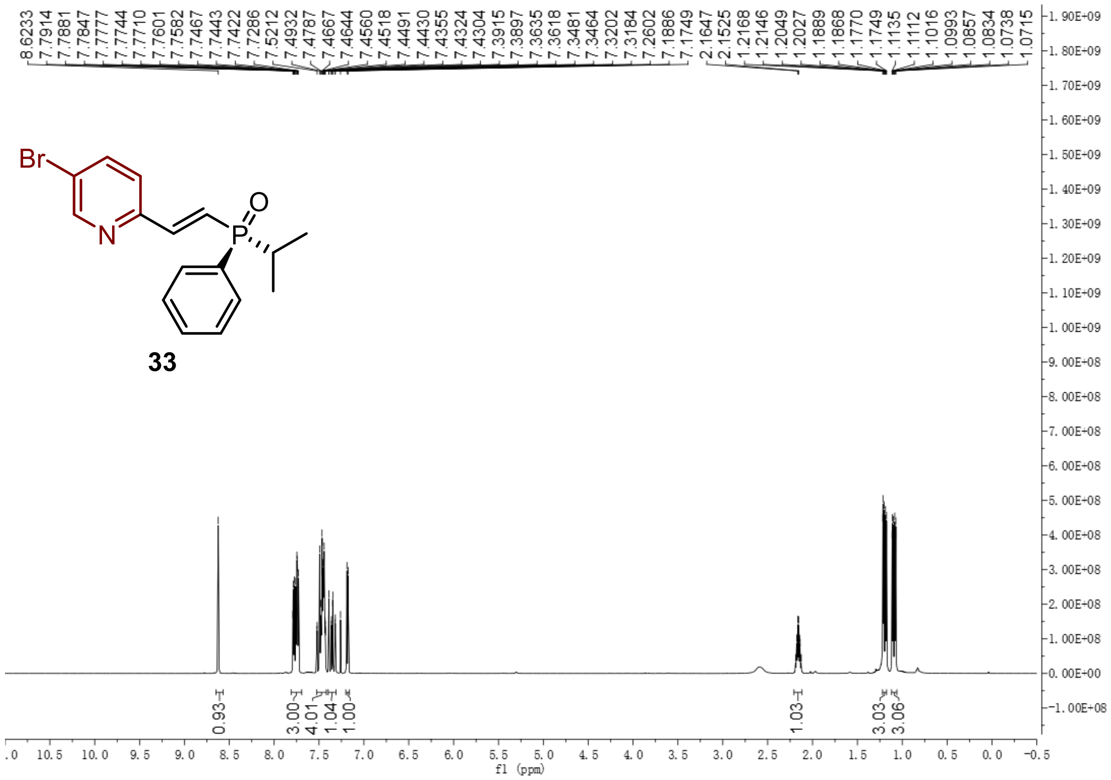

33

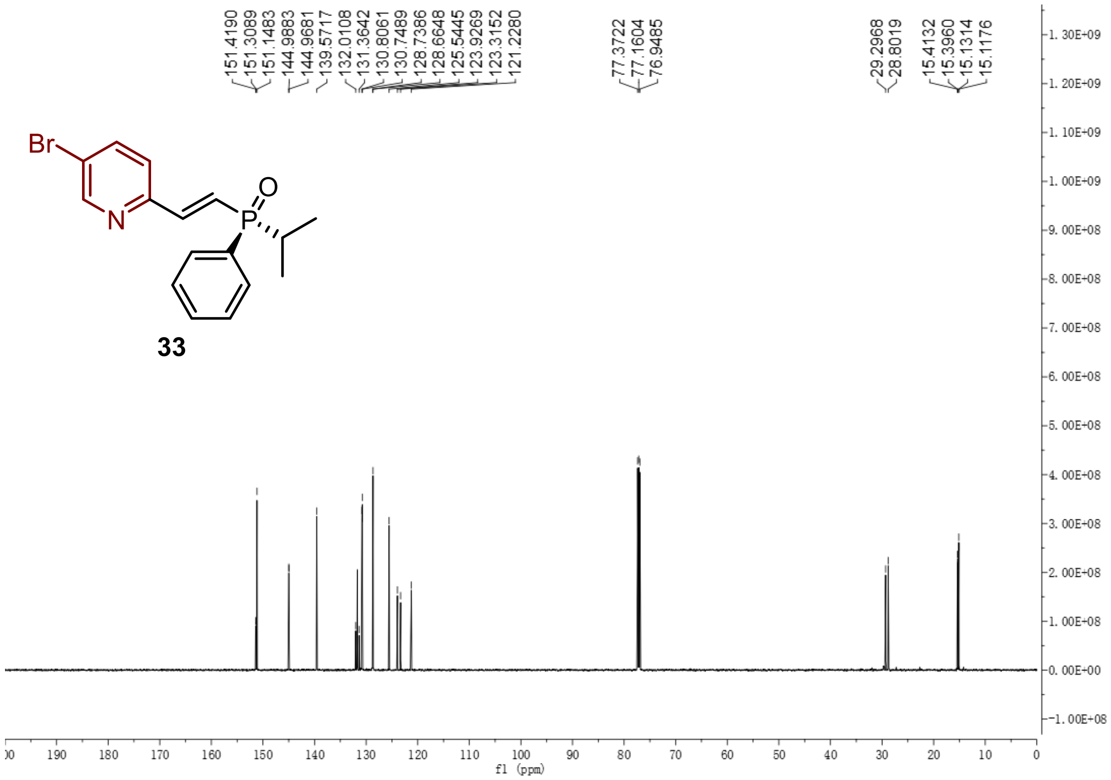
151.4190
151.3089
151.1483
144.9883
144.9681
139.5717
132.0108
131.3642
130.8061
130.7489
128.7386
128.6648
125.5445
123.9269
123.3152
121.2280
77.3722
77.1604
76.9485
29.2968
28.8019
15.4132
15.3960
15.1314
15.1176
Br
N
O
P
33
f1 (ppm)

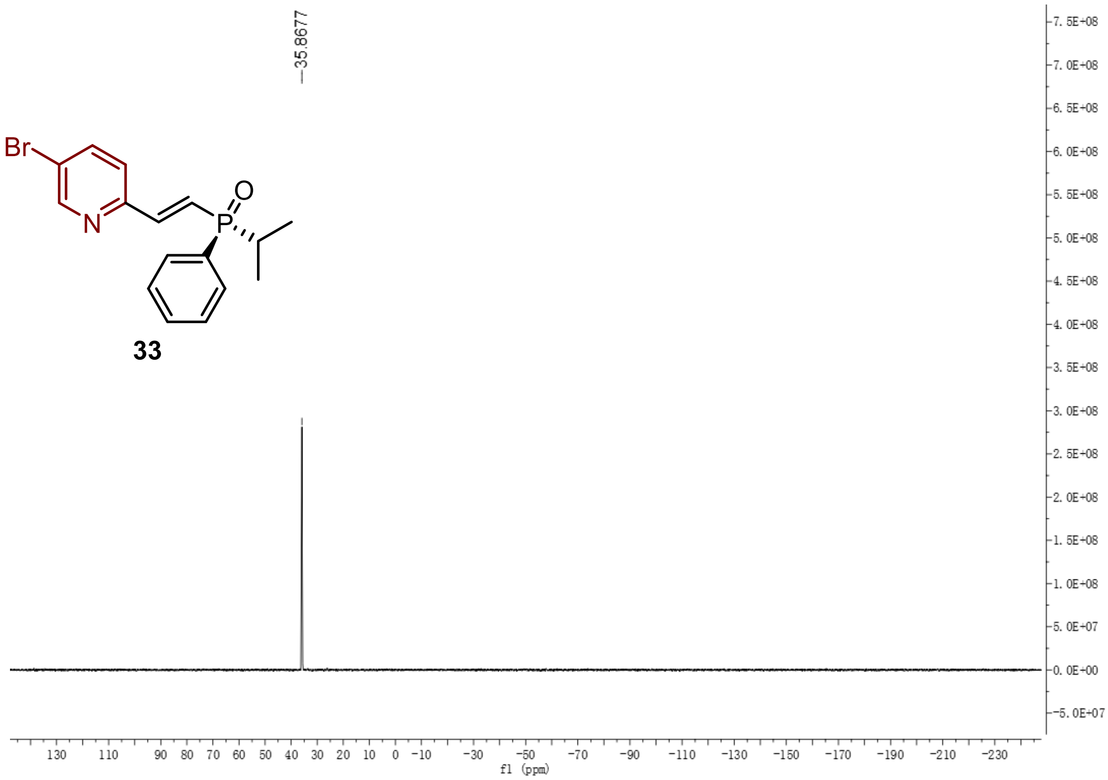
35.8677
Br
N
O
P
33
f1 (ppm)

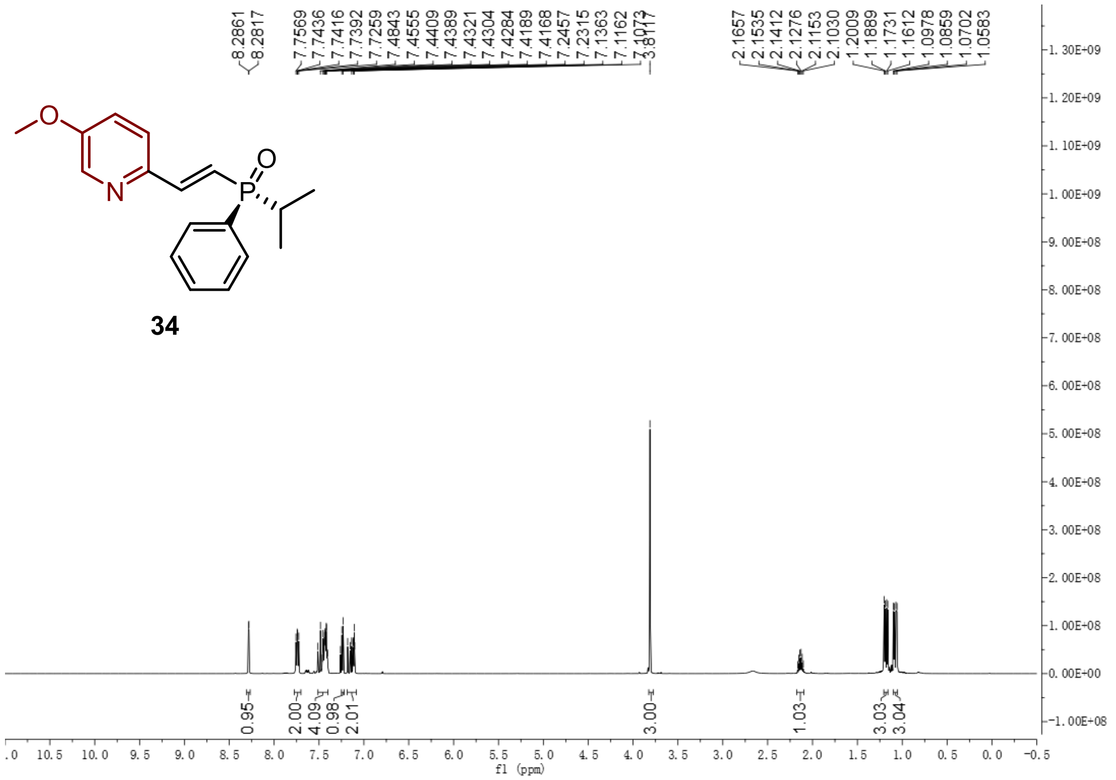
34
8.2861
8.2817
7.7569
7.7436
7.7416
7.7392
7.7259
7.4843
7.4555
7.4409
7.4389
7.4321
7.4304
7.4284
7.4189
7.4168
7.2457
7.2315
7.1363
7.1162
7.1073
3.8117
2.1657
2.1535
2.1412
2.1276
2.1153
2.1030
1.2009
1.1889
1.1731
1.1612
1.0978
1.0859
1.0702
1.0583
0.95
2.00
4.09
0.98
2.01
3.00
1.03
3.03
3.04
f1 (ppm)

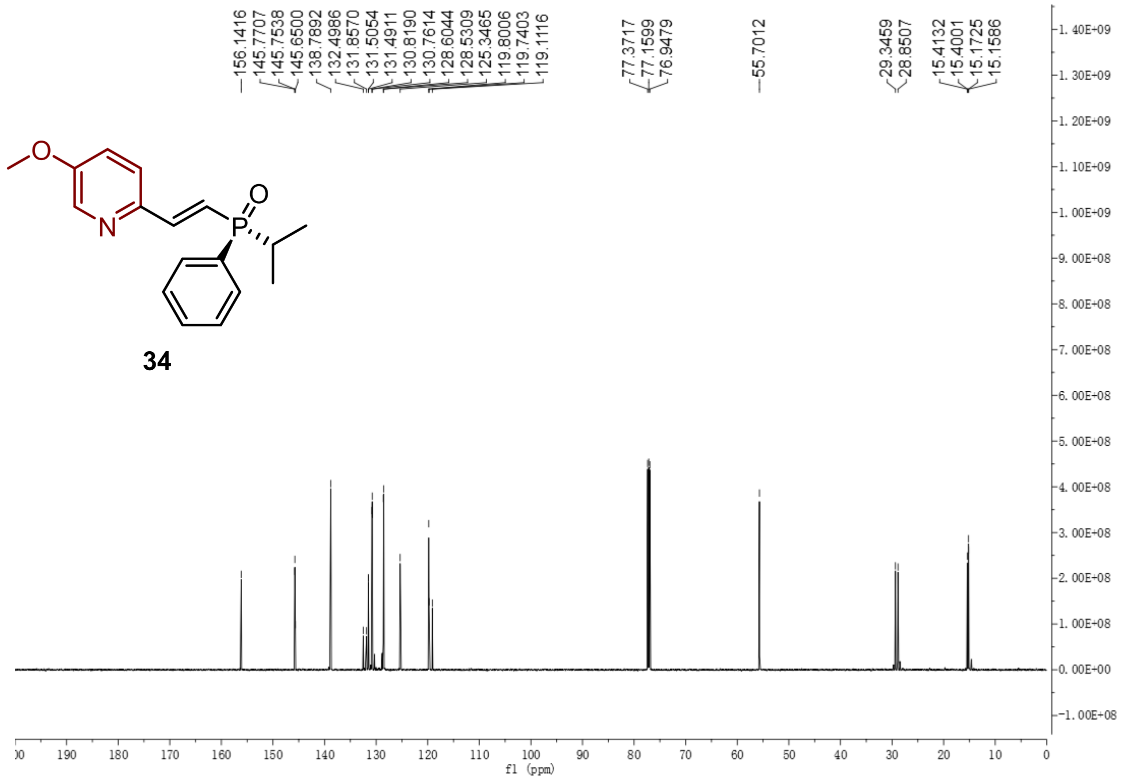
34
156.1416
145.7707
145.7538
145.6500
138.7892
132.4986
131.8570
131.5054
131.4911
130.8190
130.7614
128.6044
128.5309
125.3465
119.8006
119.7403
119.1116
77.3717
77.1599
76.9479
55.7012
29.3459
28.8507
15.4132
15.4001
15.1725
15.1586
f1 (ppm)

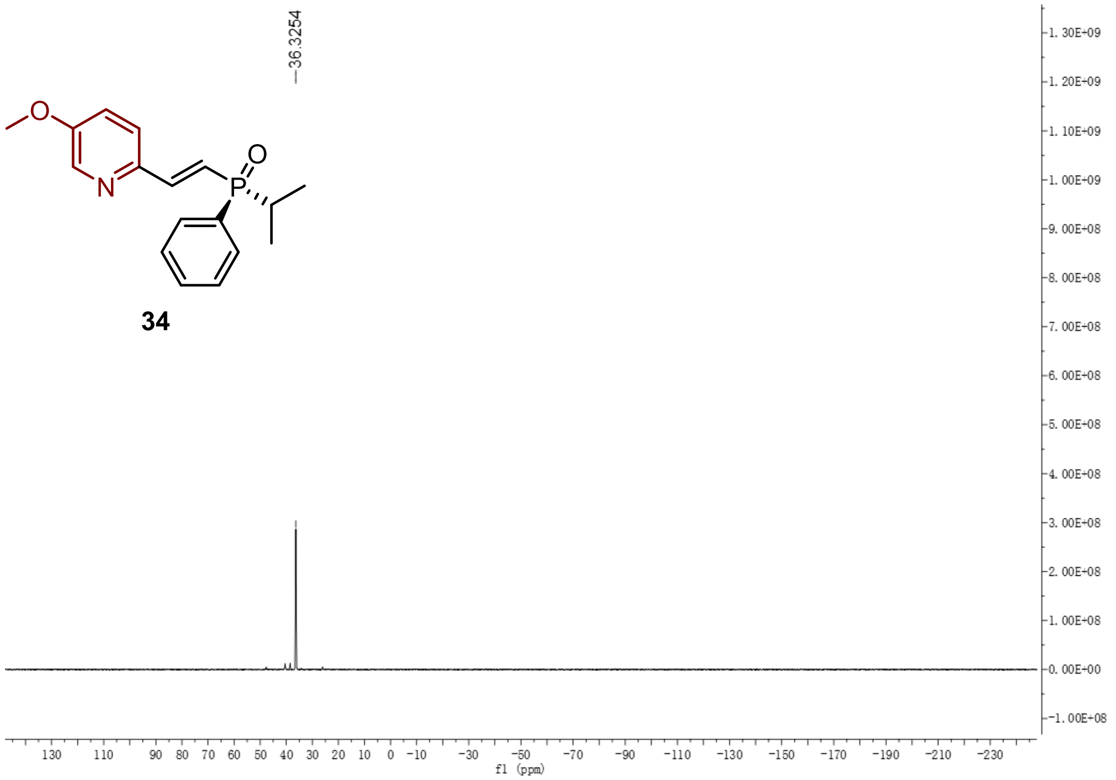

34

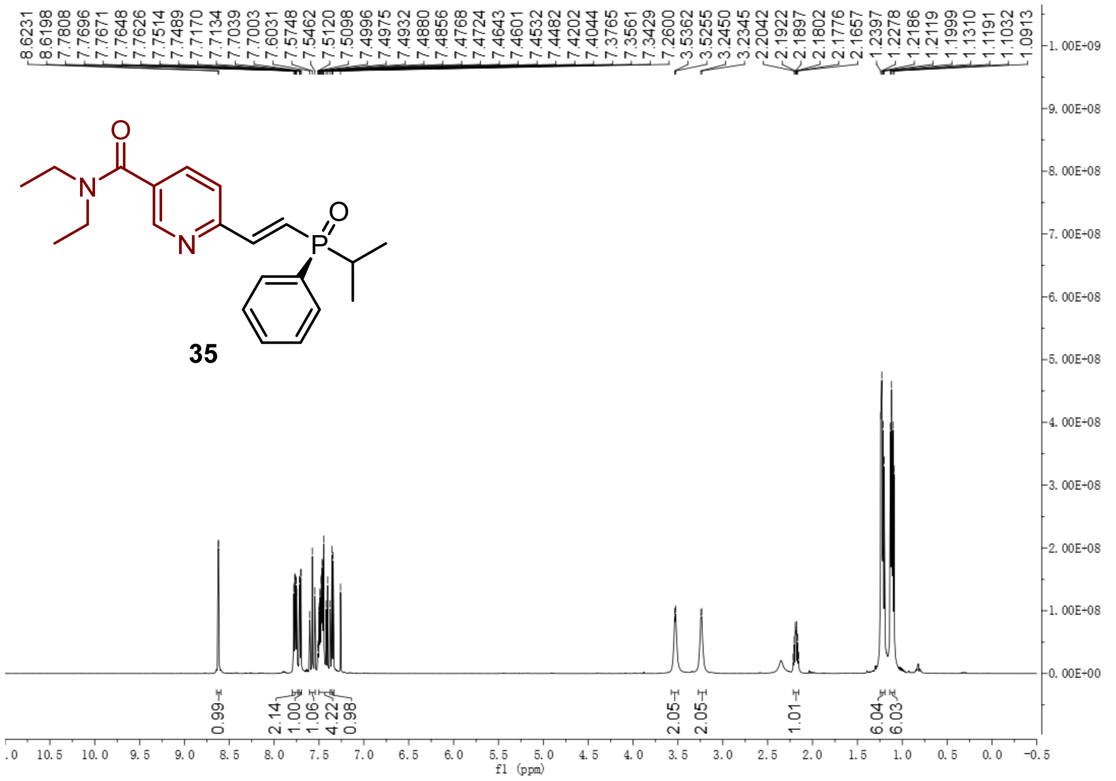

35

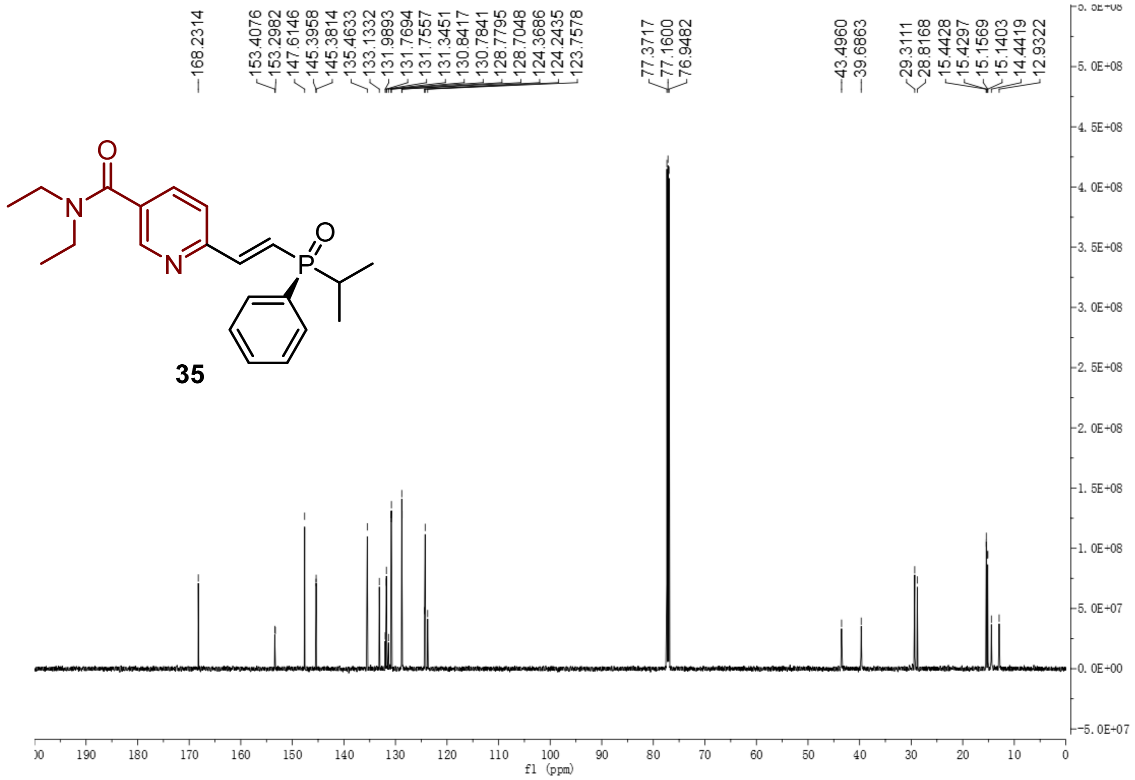
168.2314
153.4076
153.2982
147.6146
145.3958
145.3814
135.4633
133.1332
131.9893
131.7694
131.7557
131.3451
130.8417
130.7841
128.7795
128.7048
124.3686
124.2435
123.7578
77.3717
77.1600
76.9482
43.4960
39.6863
29.3111
28.8168
15.4428
15.4297
15.1569
15.1403
14.4419
12.9322
O
N
N
P
fl (ppm)

35

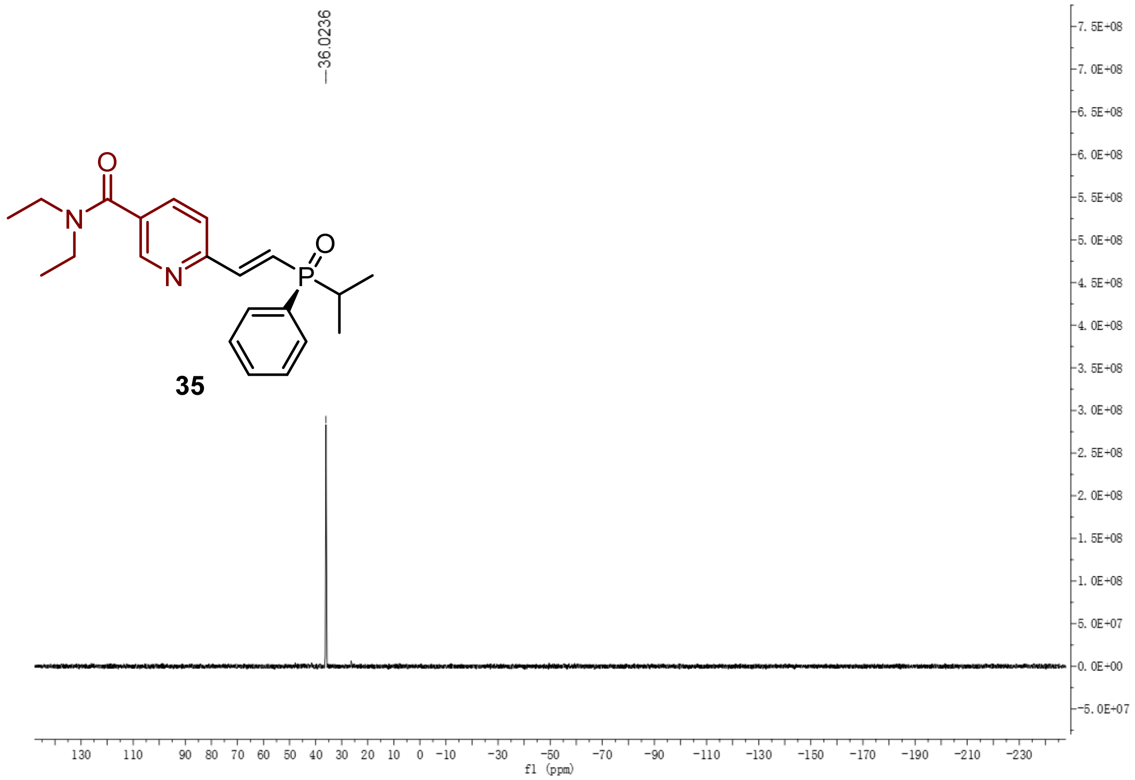
36.0236
O
N
N
P
fl (ppm)

35

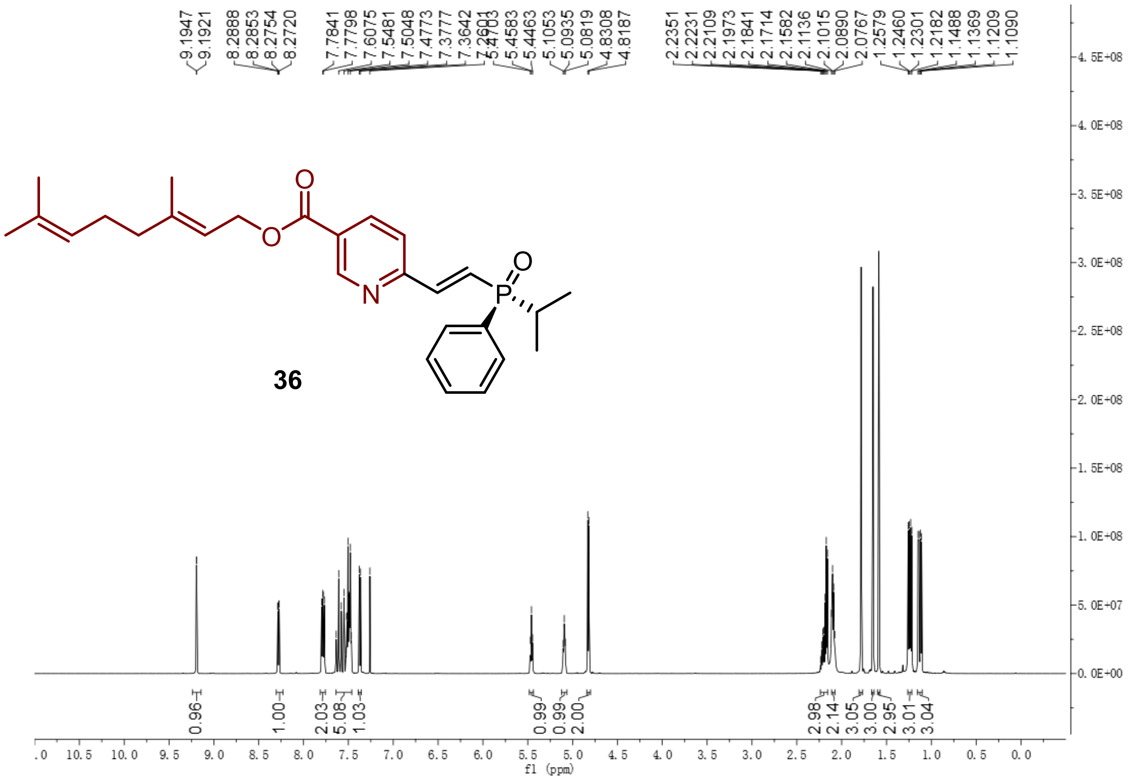

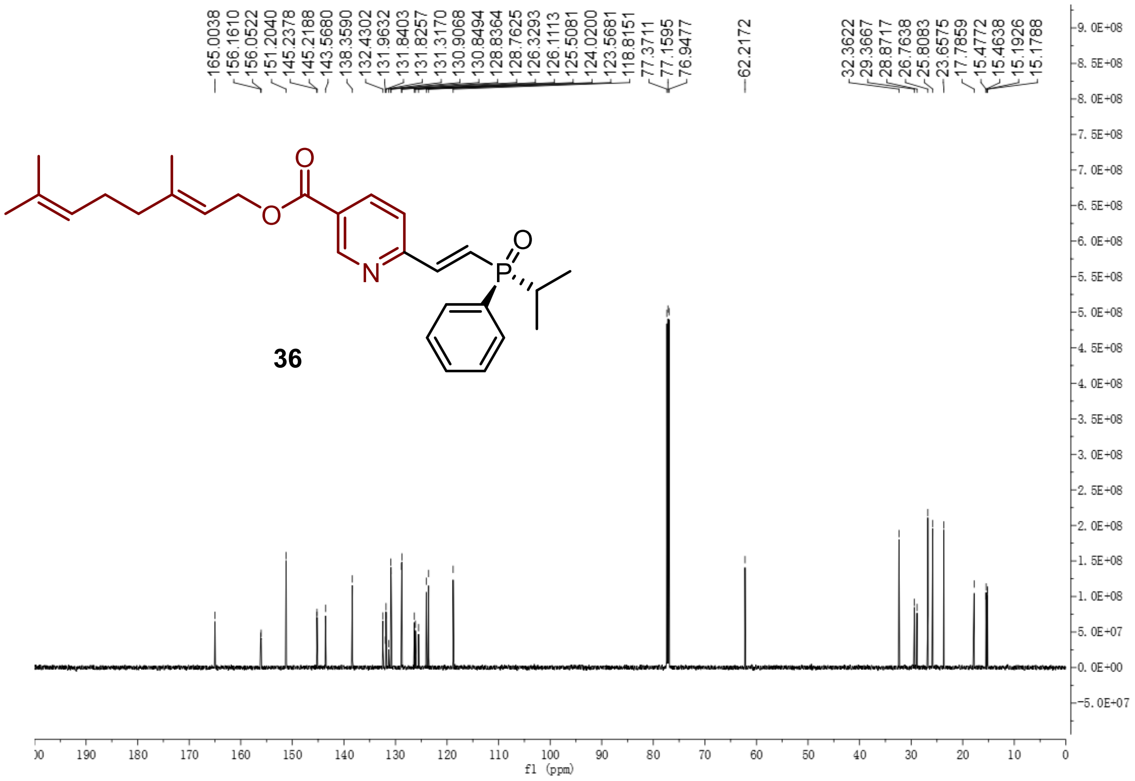

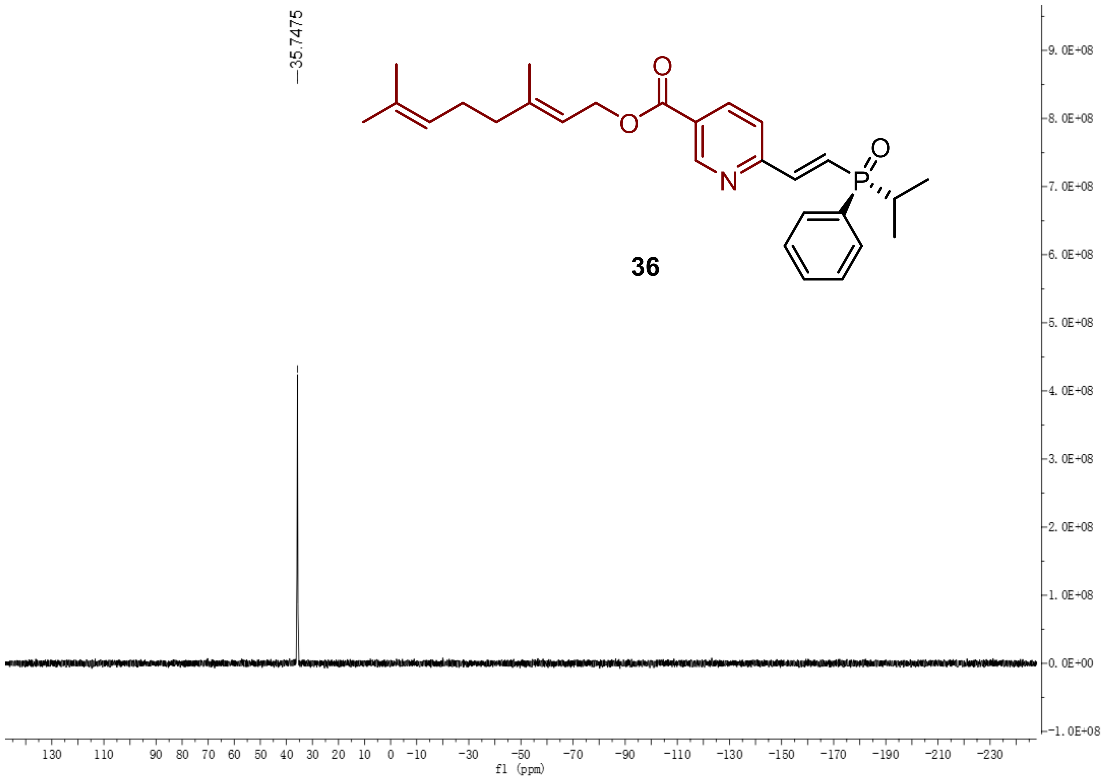
36
f1 (ppm)

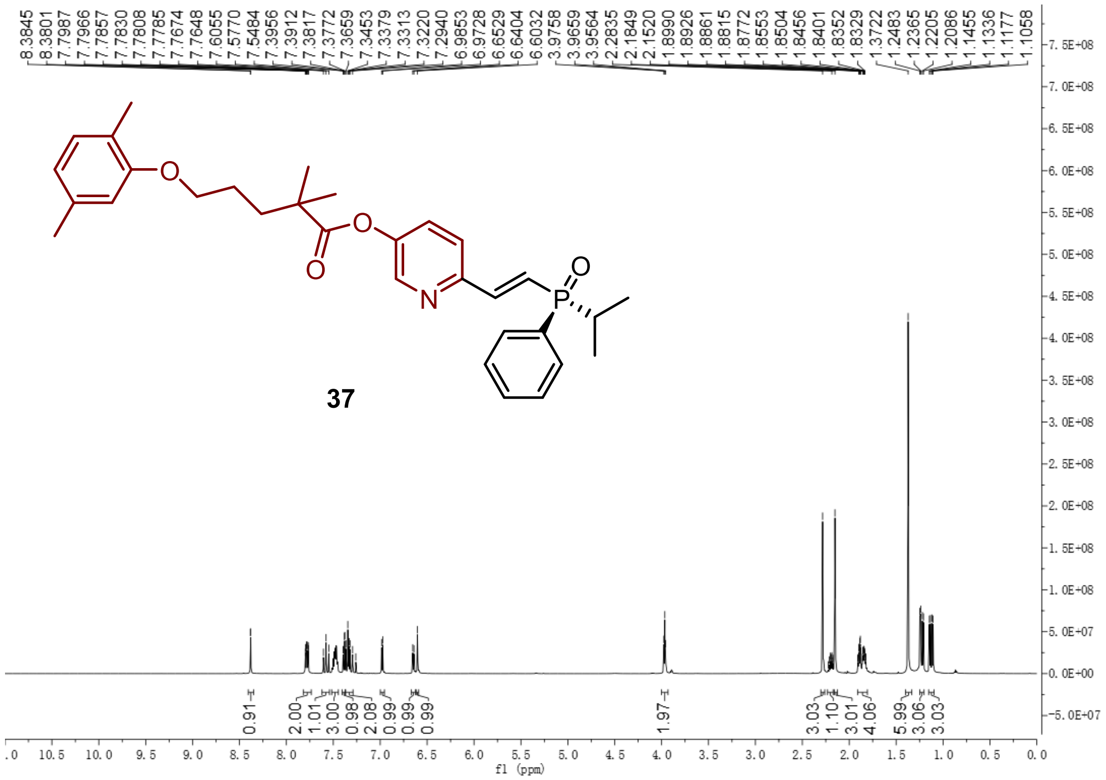
37
f1 (ppm)

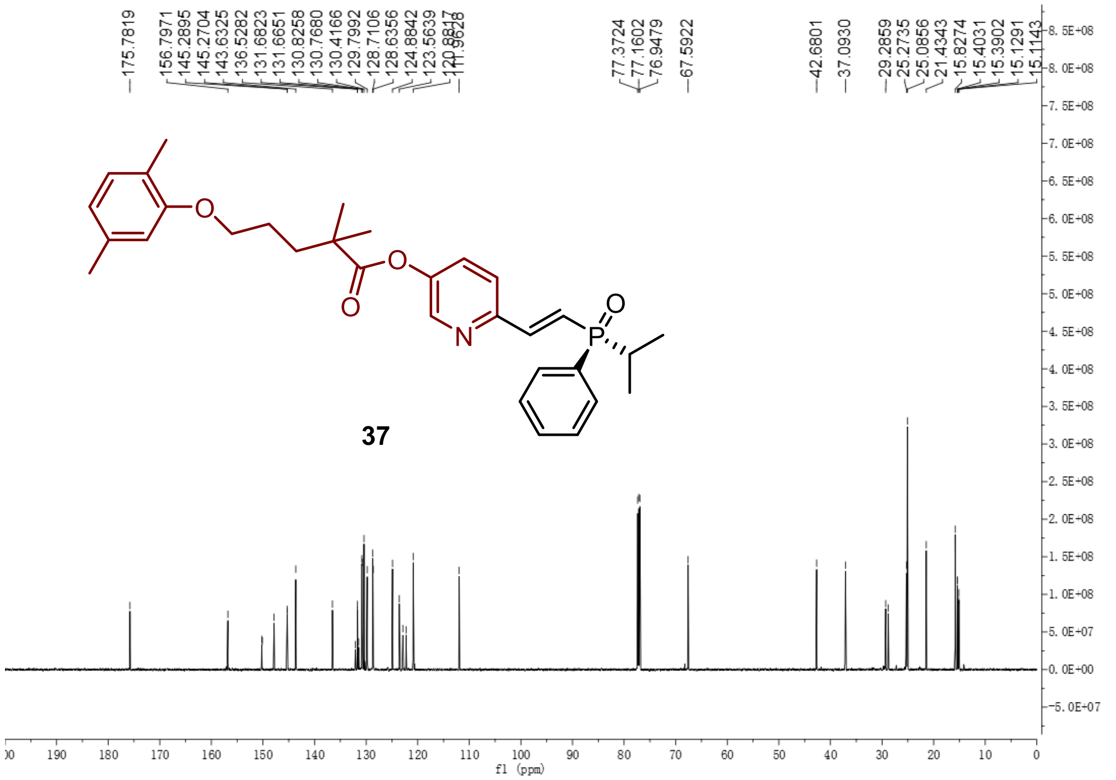

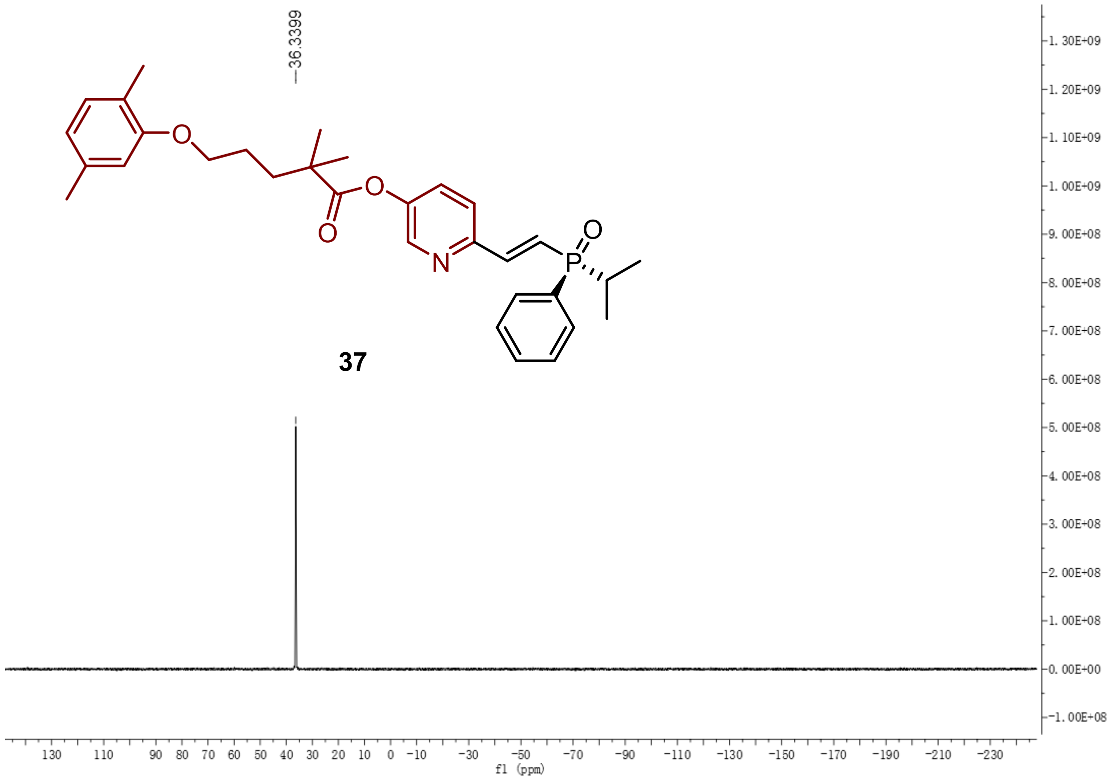

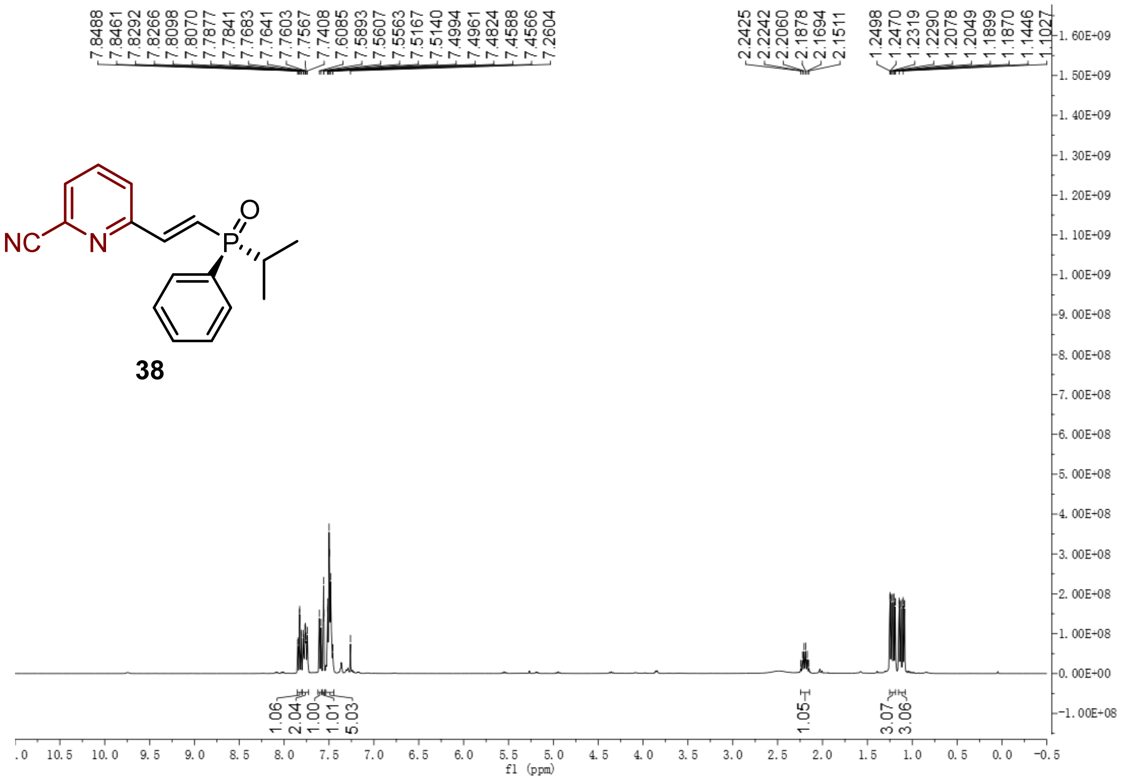

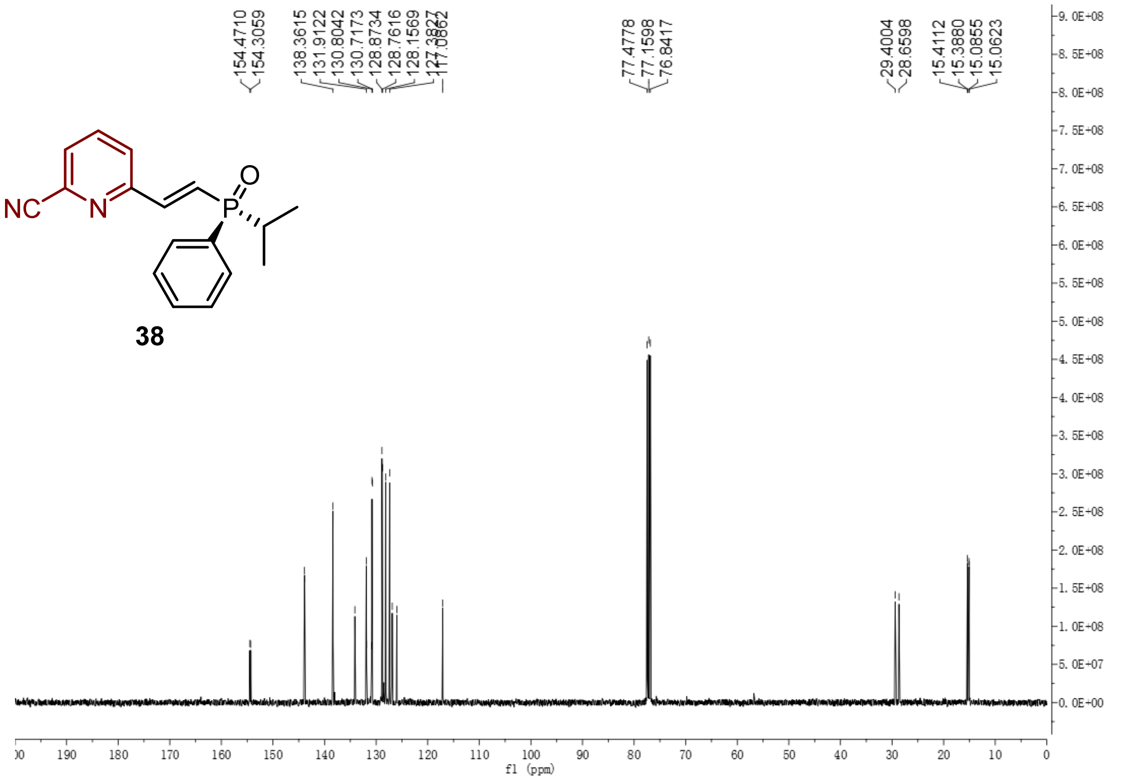

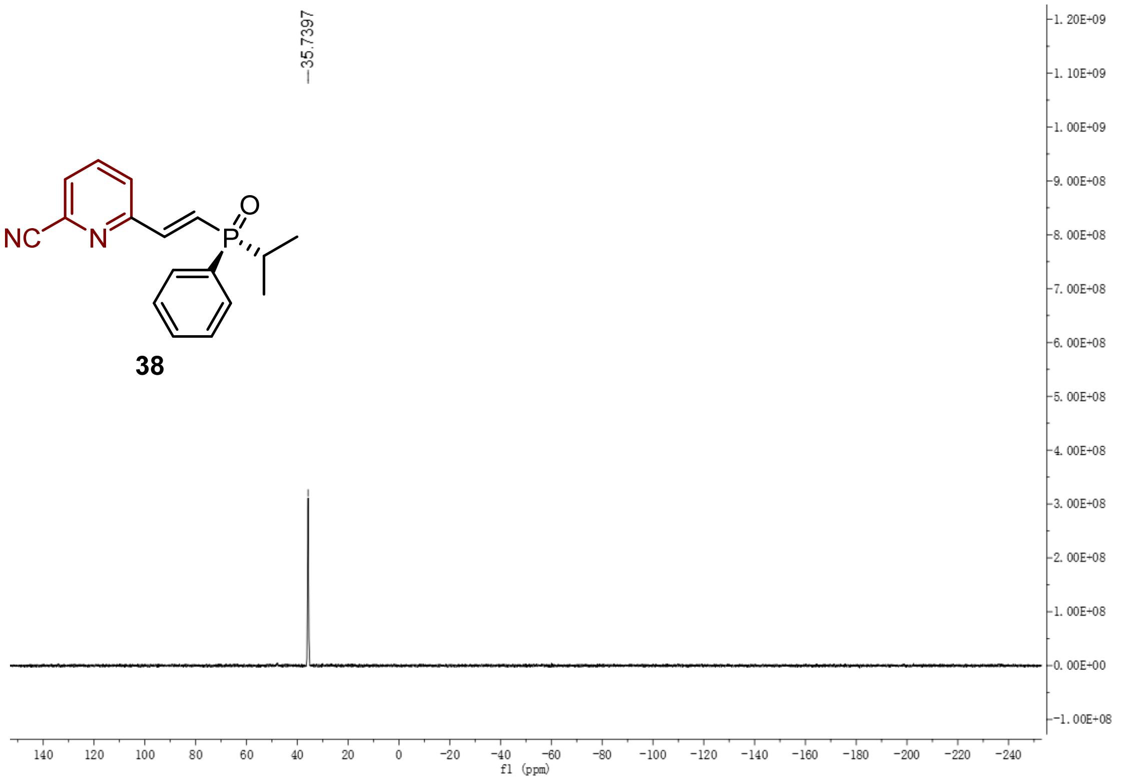

NC
N
O
P
35.7397
f1 (ppm)

38

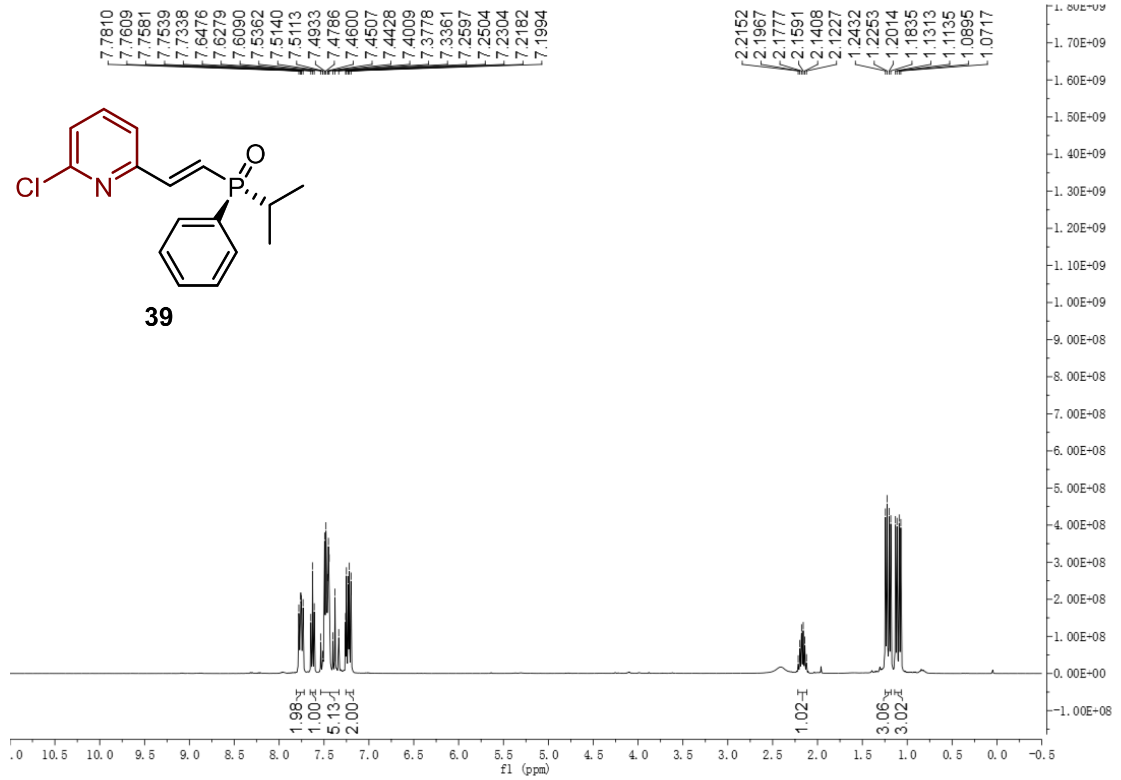

Cl
N
O
P
7.7810 7.7609 7.7581 7.7539 7.7338 7.6476 7.6279 7.6090 7.5362 7.5140 7.5113 7.4933 7.4786 7.4600 7.4507 7.4428 7.4009 7.3778 7.3361 7.2597 7.2504 7.2304 7.2182 7.1994
2.2152 2.1967 2.1777 2.1591 2.1408 2.1227 1.2432 1.2253 1.2014 1.1835 1.1313 1.1135 1.0895 1.0717
1.98 1.00 5.13 2.00 1.02 3.06 3.02
f1 (ppm)

39

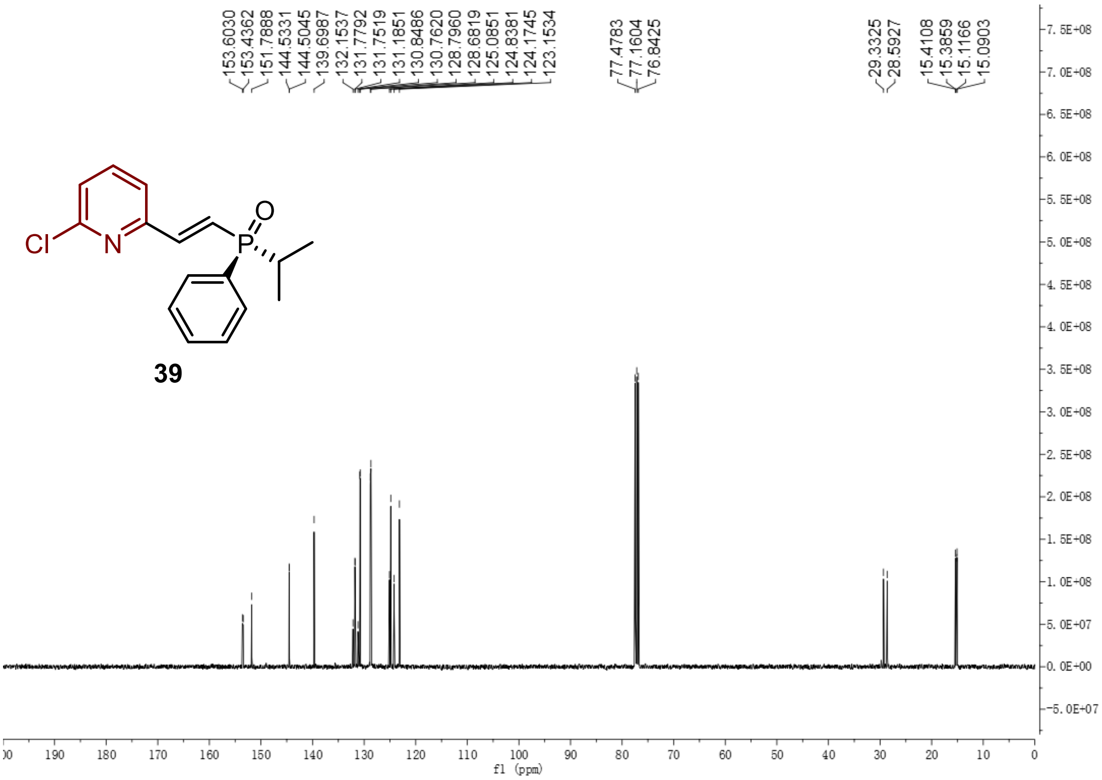
153.6030
153.4362
151.7888
144.5331
144.5045
139.6987
132.1537
131.7792
131.7519
131.1851
130.8486
130.7620
128.7960
128.6819
125.0851
124.8381
124.1745
123.1534
77.4783
77.1604
76.8425
29.3325
28.5927
15.4108
15.3859
15.1166
15.0903
Cl
N
O
P
39
f1 (ppm)

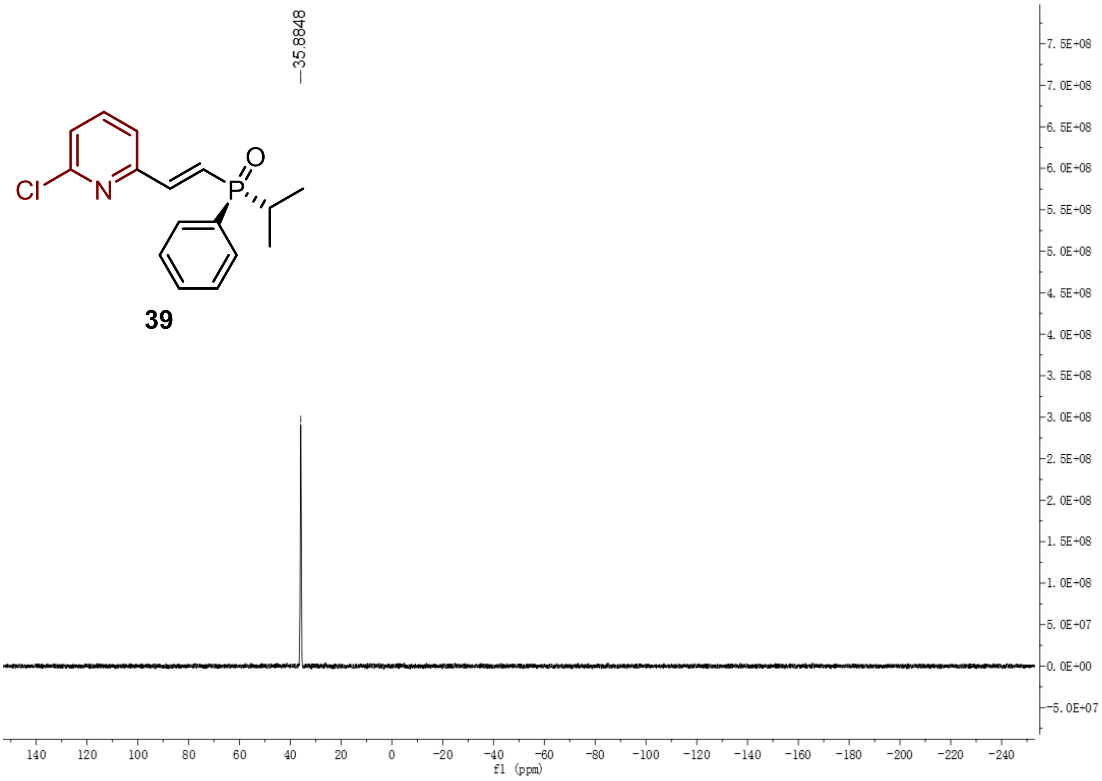
35.8848
Cl
N
O
P
39
f1 (ppm)

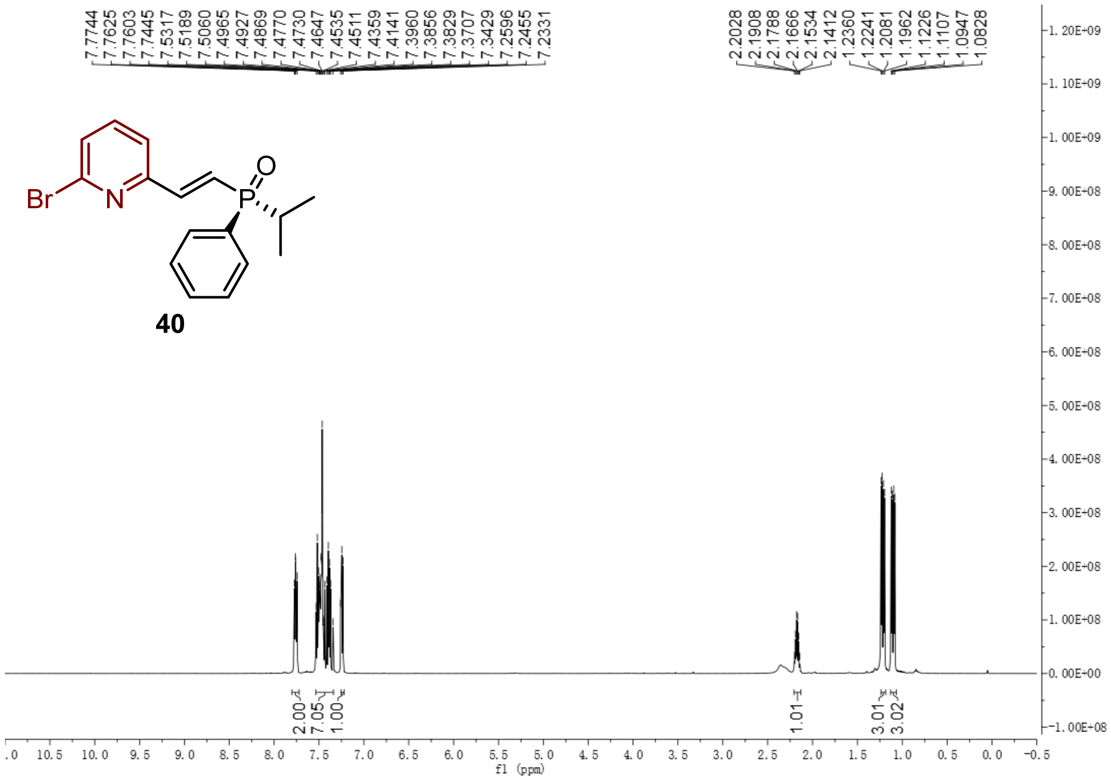
7.7744
7.7625
7.7603
7.7445
7.5317
7.5189
7.5060
7.4965
7.4927
7.4869
7.4770
7.4730
7.4647
7.4535
7.4511
7.4359
7.4141
7.3960
7.3856
7.3829
7.3707
7.3429
7.2596
7.2455
7.2331
2.2028
2.1908
2.1788
2.1666
2.1534
2.1412
1.2360
1.2241
1.2081
1.1962
1.1226
1.1107
1.0947
1.0828
Br
N
P
O
40
2.00
7.05
1.00
1.01
3.01
3.02
f1 (ppm)

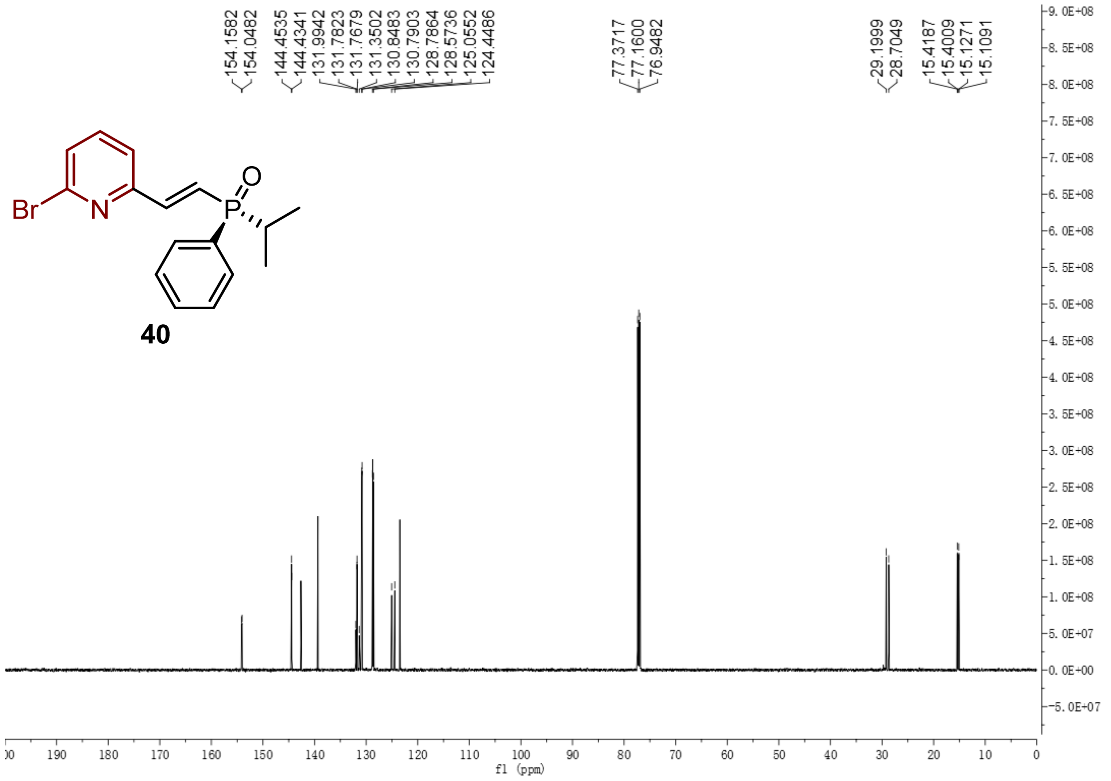
154.1582
154.0482
144.4535
144.4341
131.9942
131.7823
131.7679
131.3502
130.8483
130.7903
128.7864
128.5736
125.0552
124.4486
77.3717
77.1600
76.9482
29.1999
28.7049
15.4187
15.4009
15.1271
15.1091
Br
N
P
O
40
f1 (ppm)

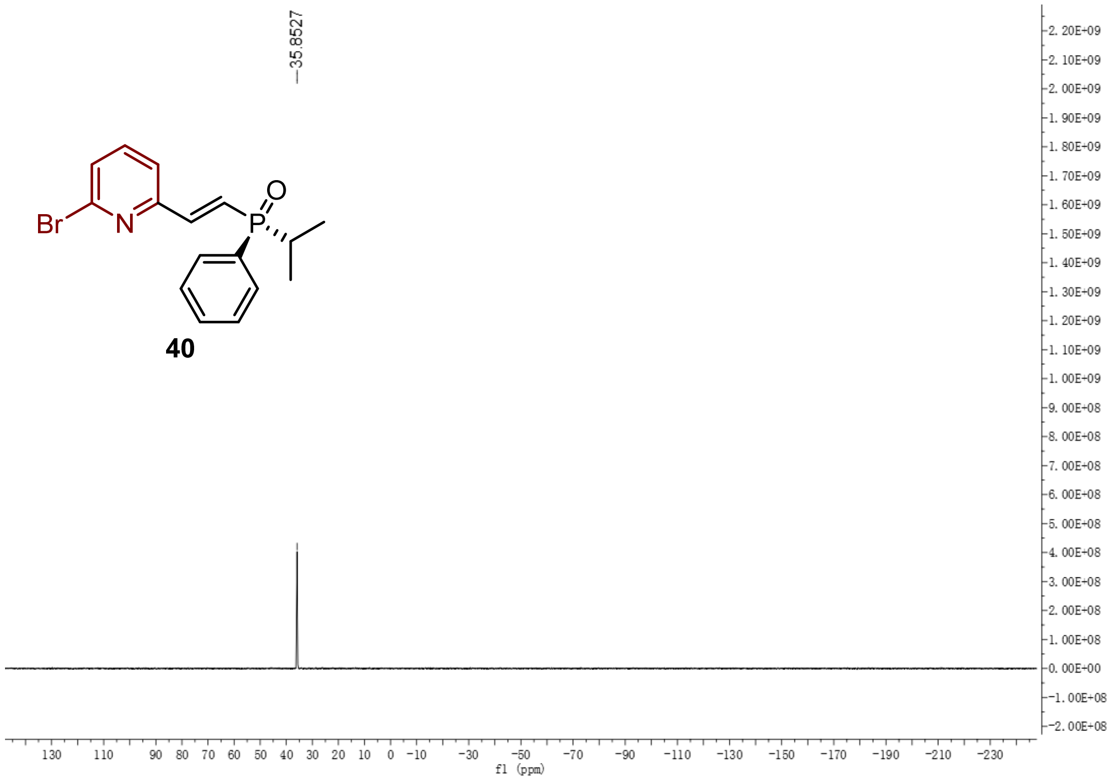

—35.8527
40
f1 (ppm)

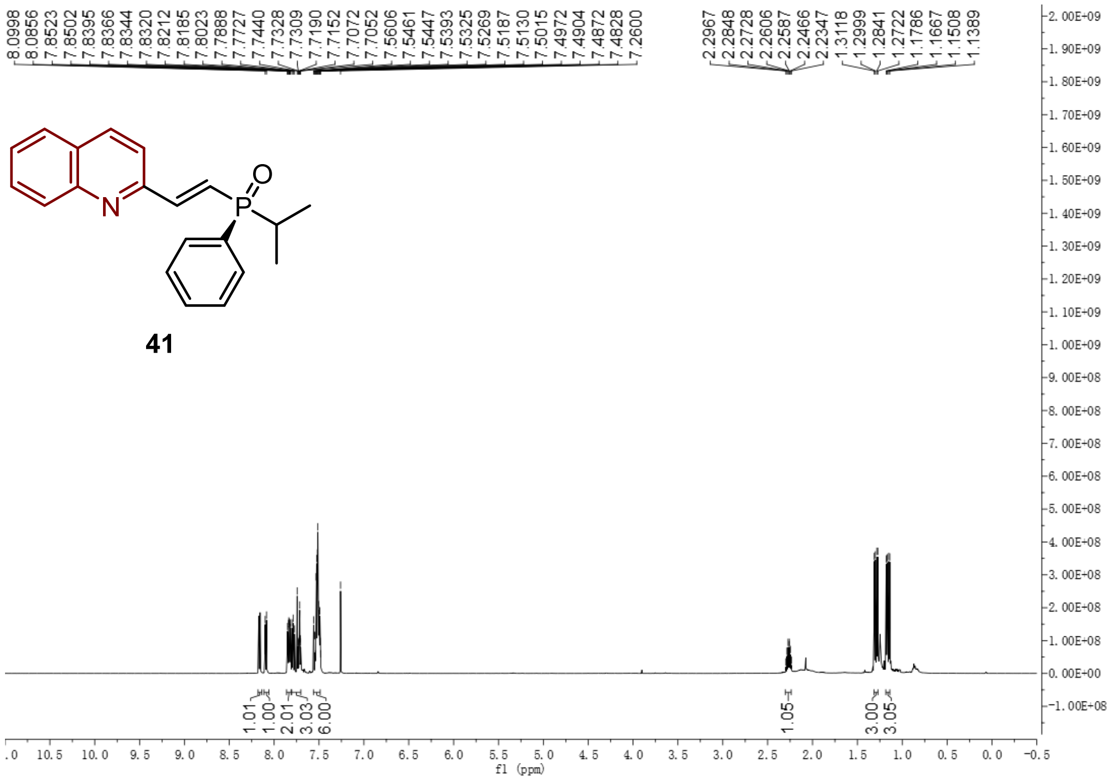

8.0998
8.0856
7.8523
7.8502
7.8395
7.8366
7.8344
7.8320
7.8212
7.8185
7.8023
7.7888
7.7727
7.7440
7.7328
7.7309
7.7190
7.7152
7.7072
7.7052
7.5606
7.5461
7.5447
7.5393
7.5325
7.5269
7.5187
7.5130
7.5015
7.4972
7.4904
7.4872
7.4828
7.2600
2.2967
2.2848
2.2728
2.2606
2.2587
2.2466
2.2347
1.3118
1.2999
1.2841
1.2722
1.1786
1.1667
1.1508
1.1389
41
1.01
1.00
2.01
3.03
6.00
1.05
3.00
3.05
f1 (ppm)

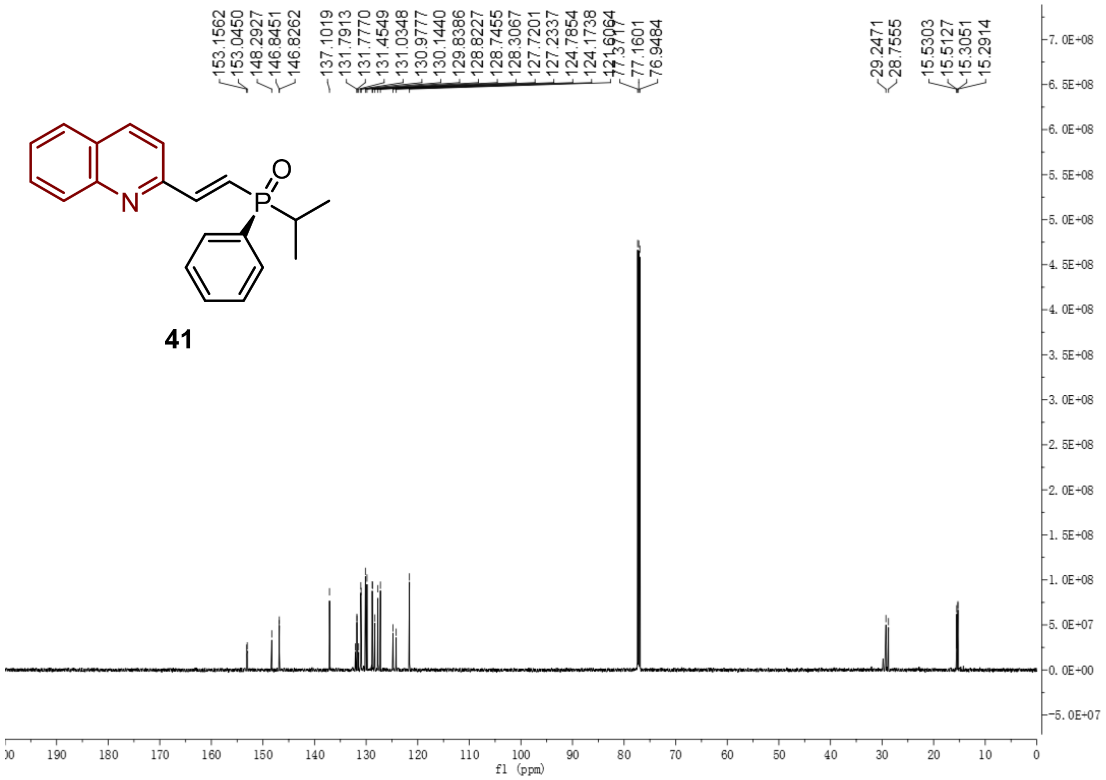

153.1562
153.0450
148.2927
146.8451
146.8262
137.1019
131.7913
131.7770
131.4549
131.0348
130.9777
130.1440
129.8386
128.8227
128.7455
128.3067
127.7201
127.2337
124.7854
124.1738
121.6064
77.3717
77.1601
76.9484
29.2471
28.7555
15.5303
15.5127
15.3051
15.2914
N
O
P
41
f1 (ppm)

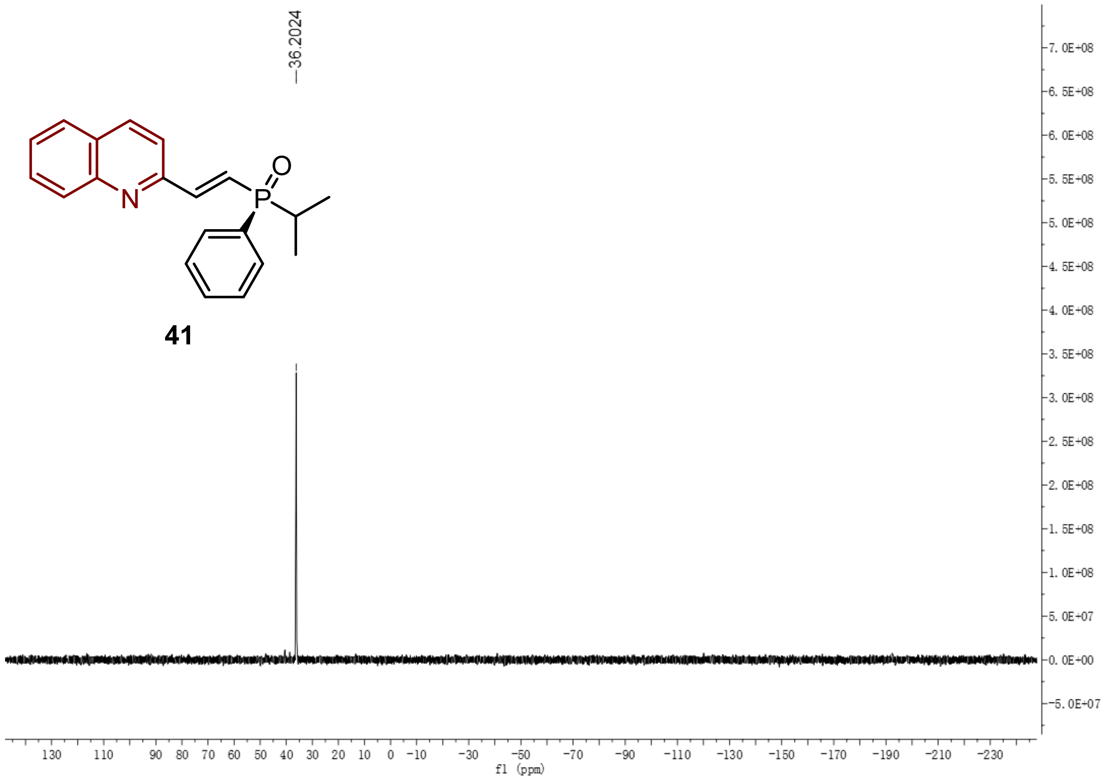

36.2024
N
O
P
41
f1 (ppm)

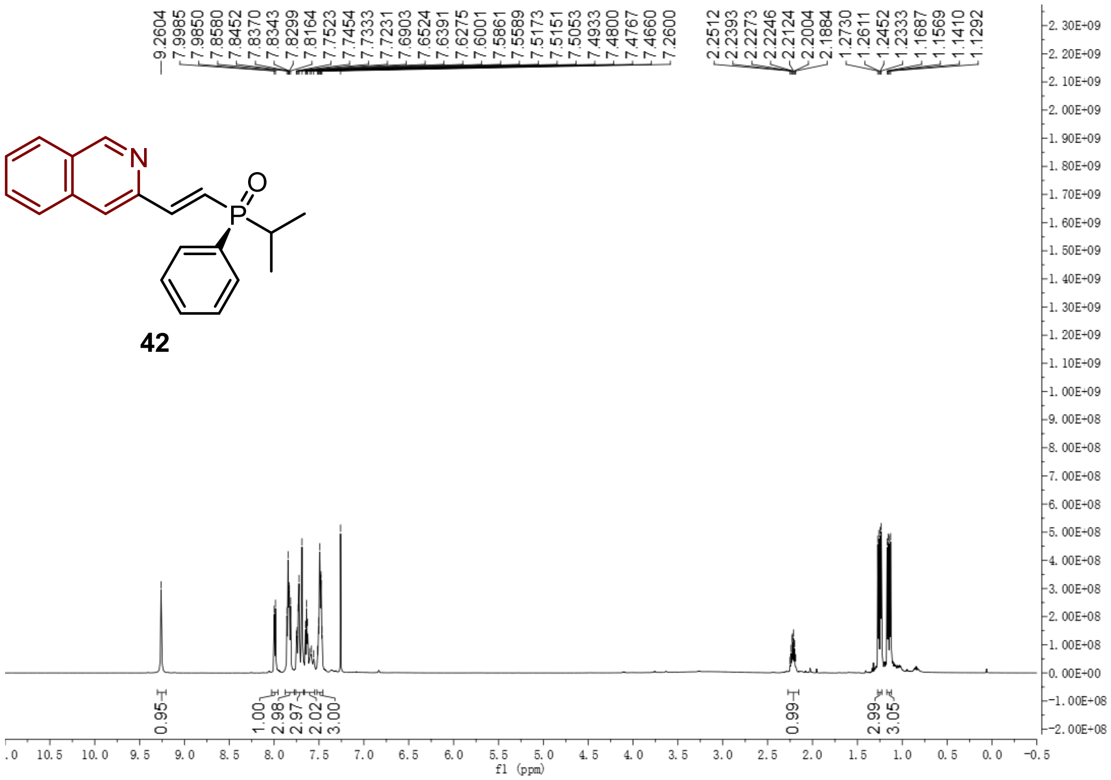

9.2604
7.9985
7.9850
7.8580
7.8452
7.8370
7.8343
7.8299
7.8164
7.7523
7.7454
7.7333
7.7231
7.6903
7.6524
7.6391
7.6275
7.6001
7.5861
7.5589
7.5173
7.5151
7.5053
7.4933
7.4800
7.4767
7.4660
7.2600
2.2512
2.2393
2.2273
2.2246
2.2124
2.2004
2.1884
1.2730
1.2611
1.2452
1.2333
1.1687
1.1569
1.1410
1.1292
N
O
P
42
0.95
1.00
2.98
2.97
2.02
3.00
0.99
2.99
3.05
f1 (ppm)

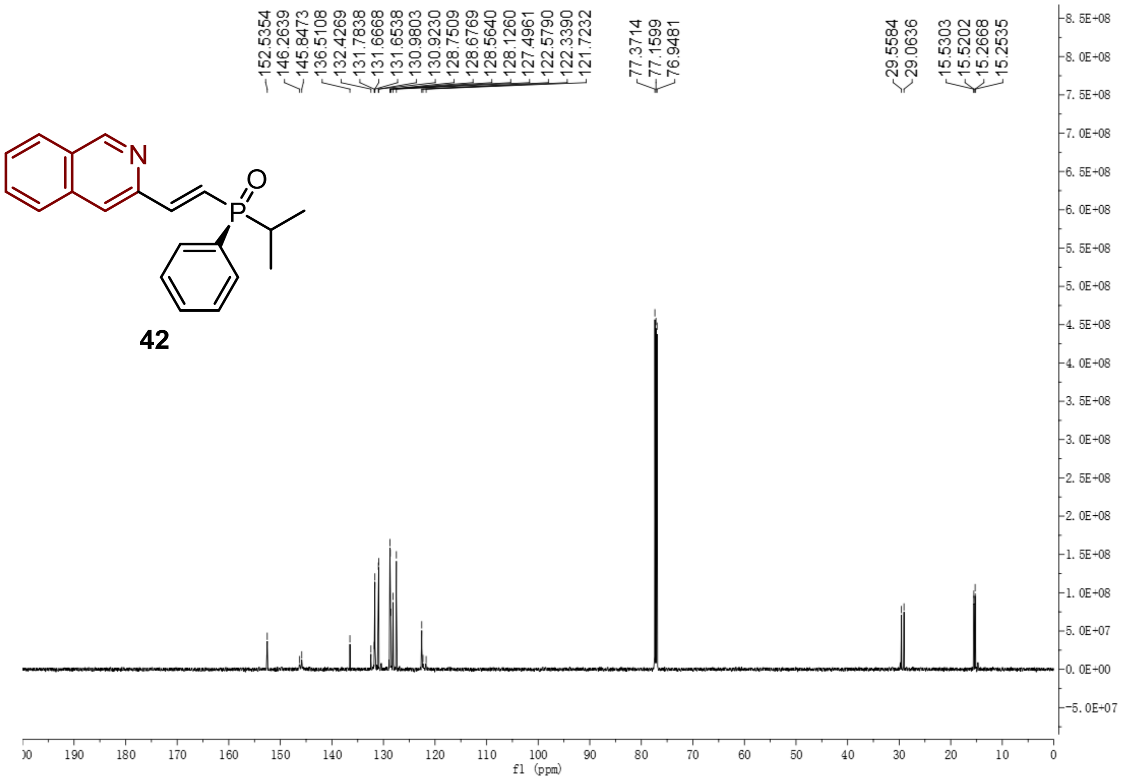

152.5354
146.2639
145.8473
136.5108
132.4269
131.7838
131.6668
131.6538
130.9803
130.9230
128.7509
128.6769
128.5640
128.1260
127.4961
122.5790
122.3390
121.7232
77.3714
77.1599
76.9481
29.5584
29.0636
15.5303
15.5202
15.2668
15.2535
N
O
P
42
f1 (ppm)

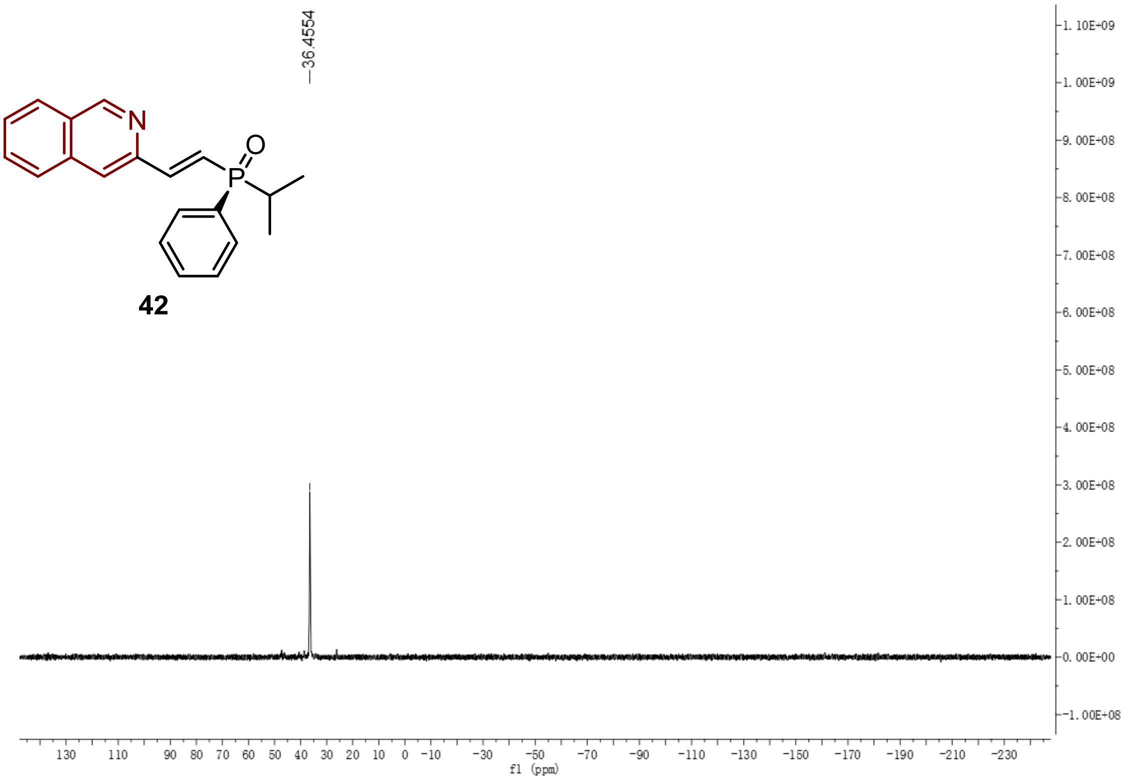

36.4554
f1 (ppm)

42

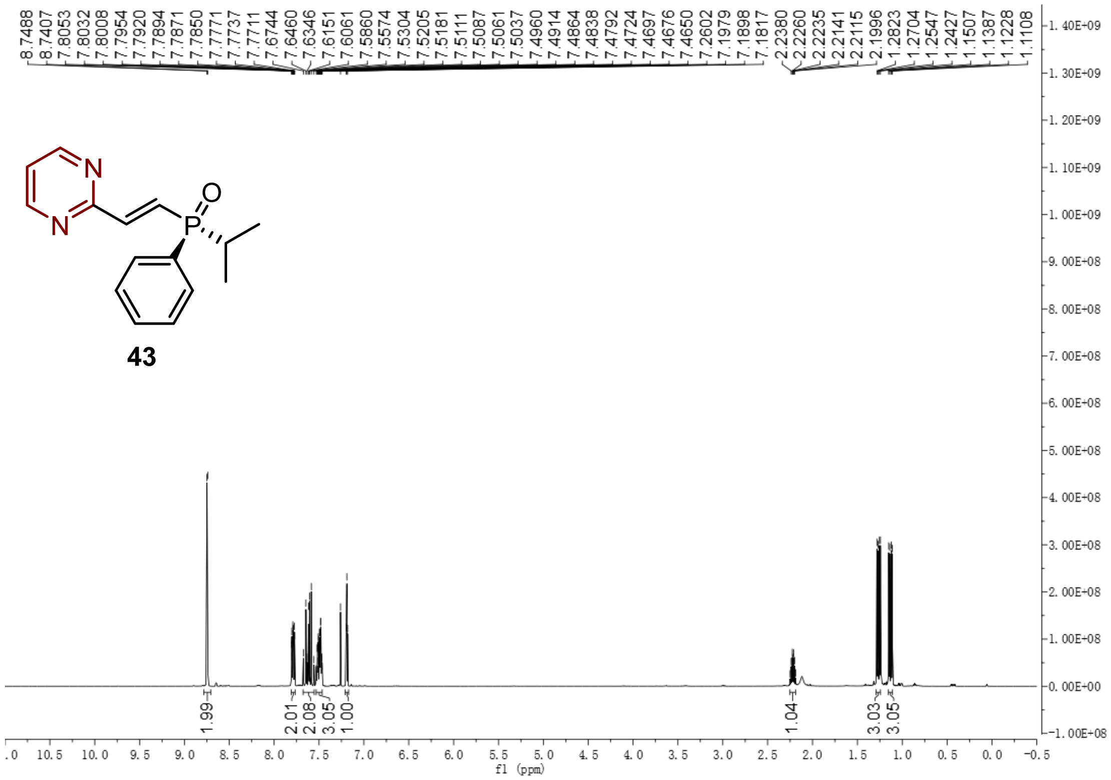

8.7488
8.7407
7.8053
7.8032
7.8008
7.7954
7.7920
7.7894
7.7871
7.7850
7.7771
7.7737
7.7711
7.6744
7.6460
7.6346
7.6151
7.6061
7.5860
7.5574
7.5304
7.5205
7.5181
7.5111
7.5087
7.5061
7.5037
7.4960
7.4914
7.4864
7.4838
7.4792
7.4724
7.4697
7.4676
7.4650
7.2602
7.1979
7.1898
7.1817
2.2380
2.2260
2.2235
2.2141
2.2115
2.1996
1.2823
1.2704
1.2547
1.2427
1.1507
1.1387
1.1228
1.1108
1.99
2.01
2.08
3.05
1.00
1.04
3.03
3.05
f1 (ppm)

43

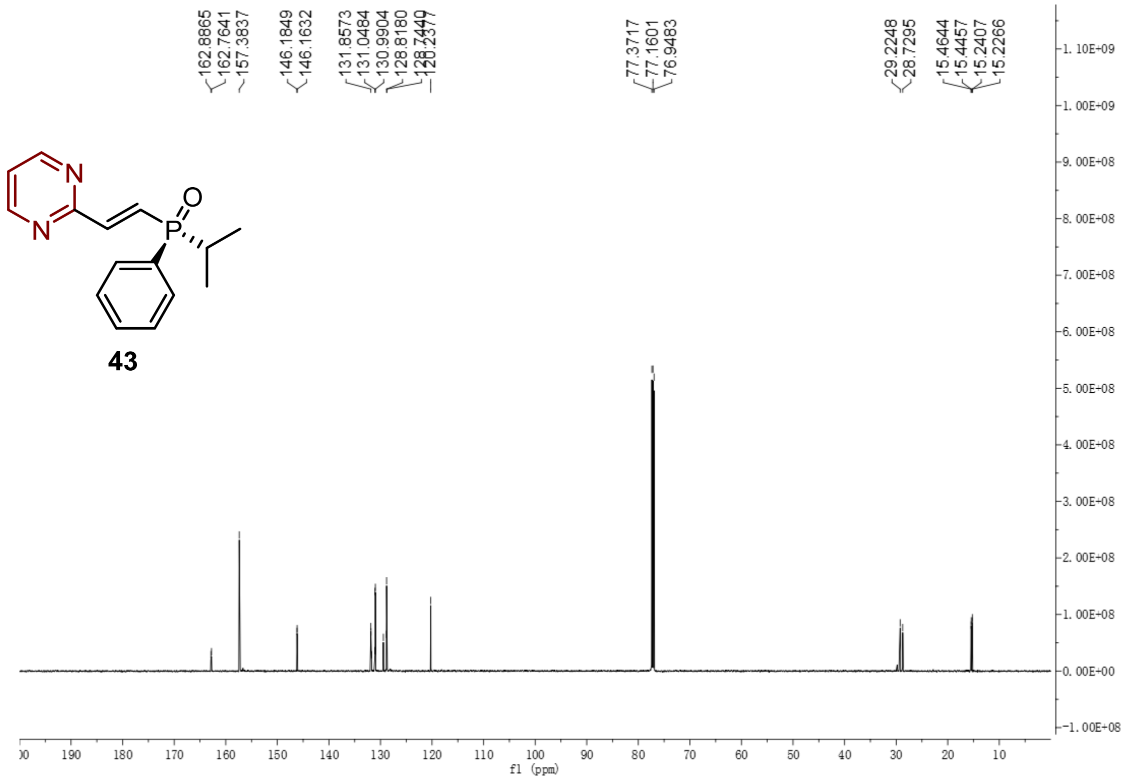
162.8865
162.7641
157.3837
146.1849
146.1632
131.8573
131.0484
130.9904
128.8180
128.7440
120.2377
77.3717
77.1601
76.9483
29.2248
28.7295
15.4644
15.4457
15.2407
15.2266
f1 (ppm)

43

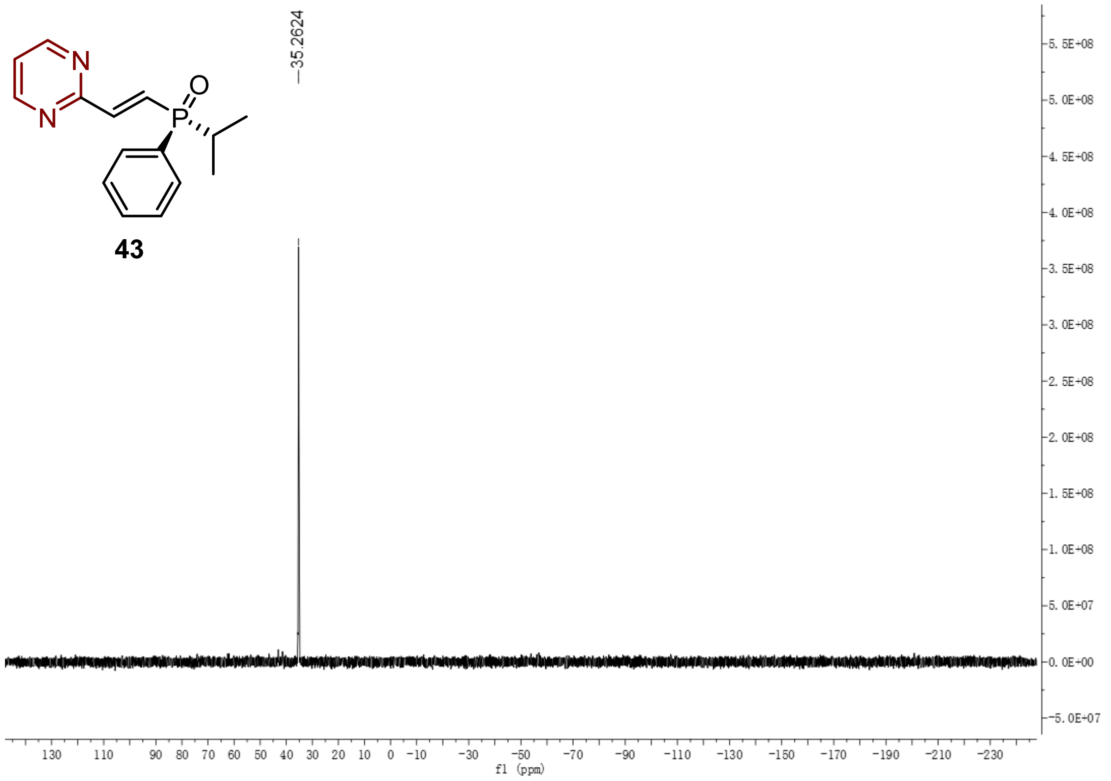
35.2624
f1 (ppm)

43

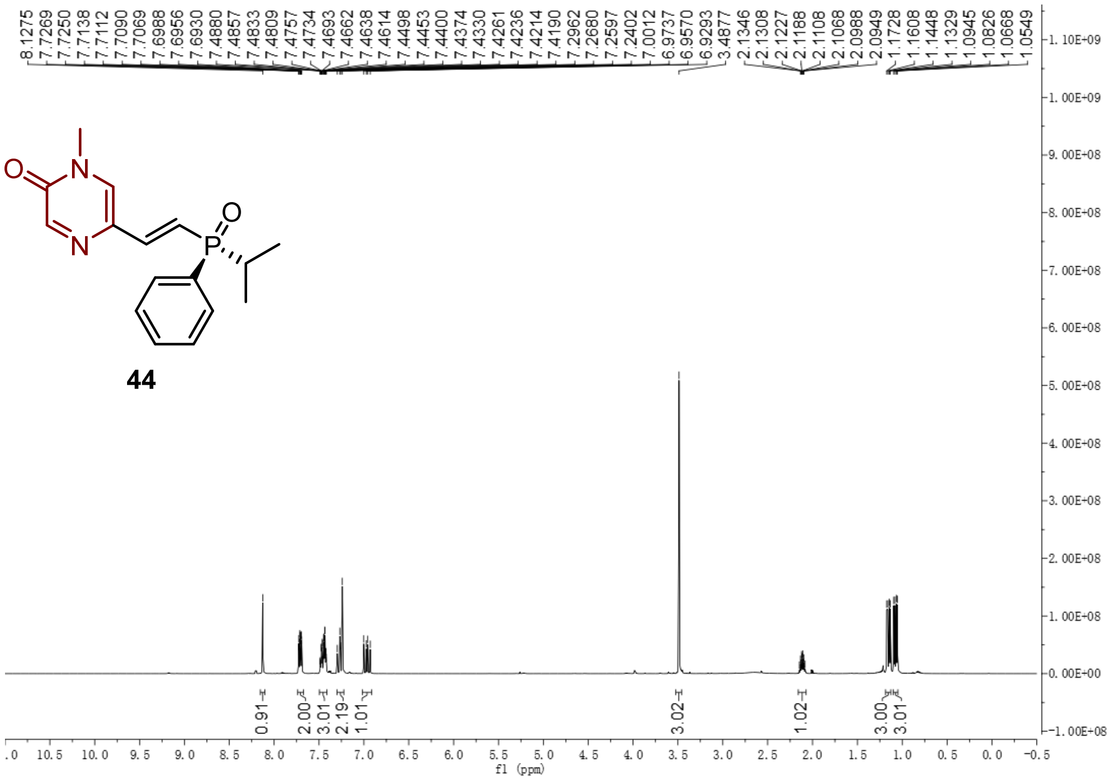

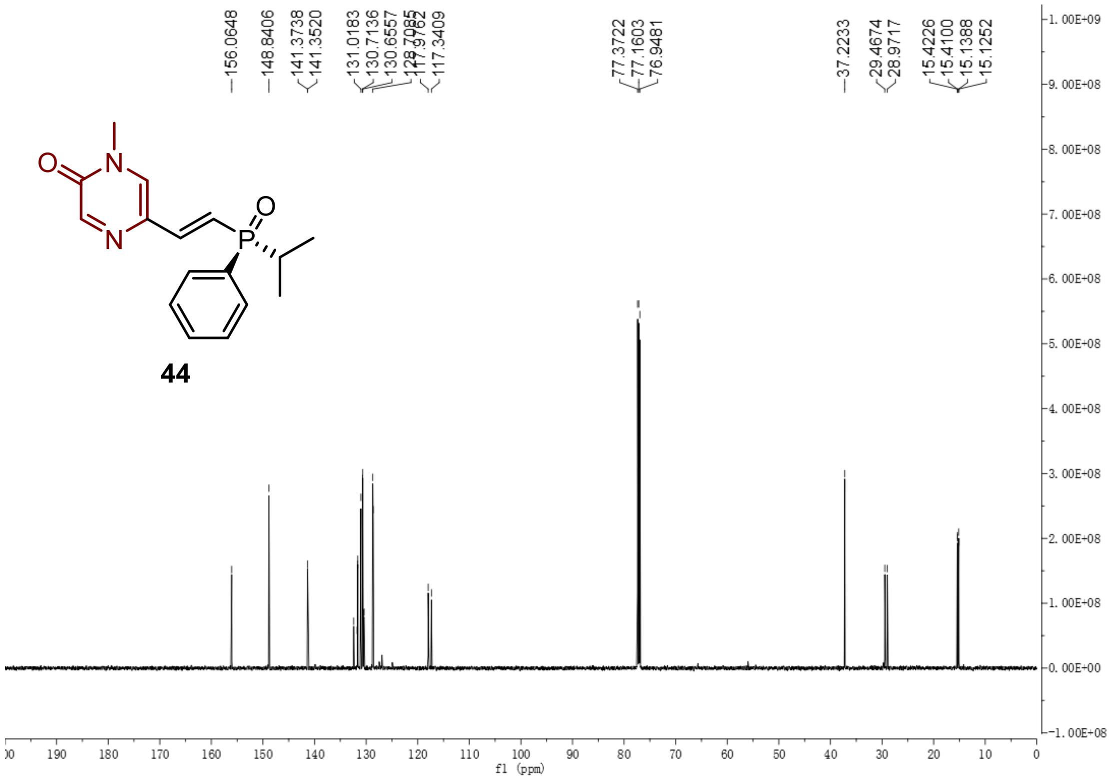

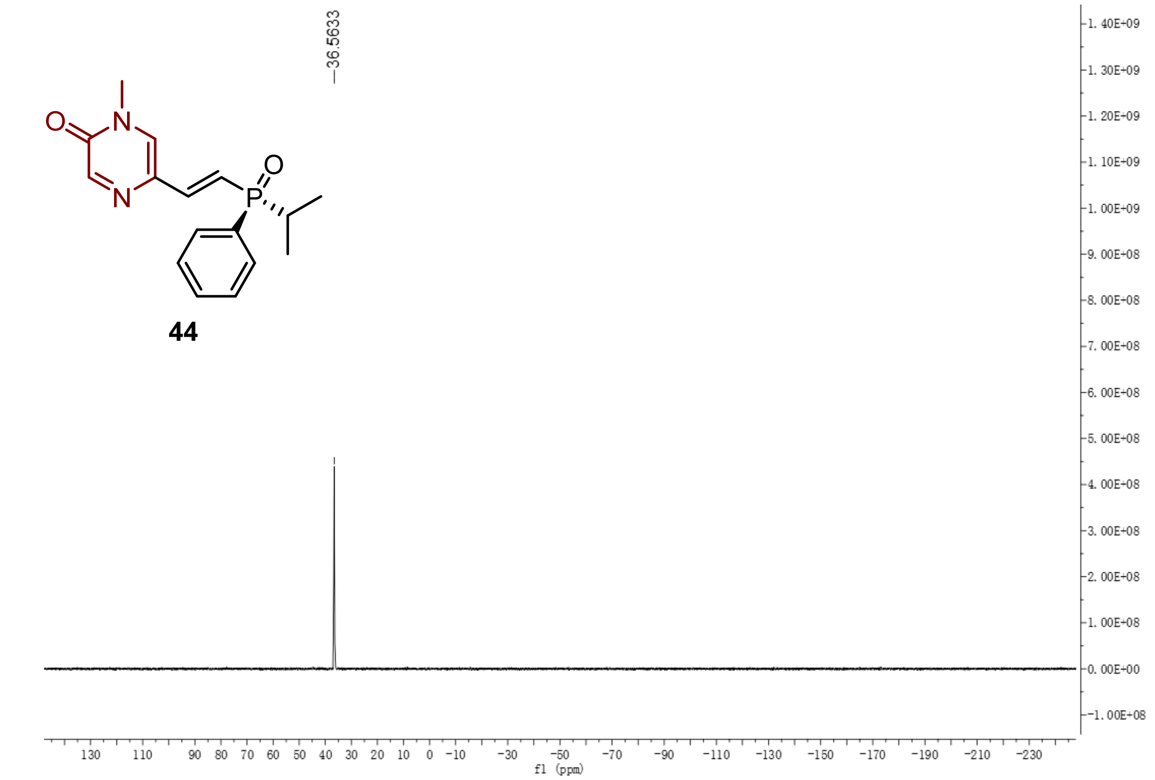

44

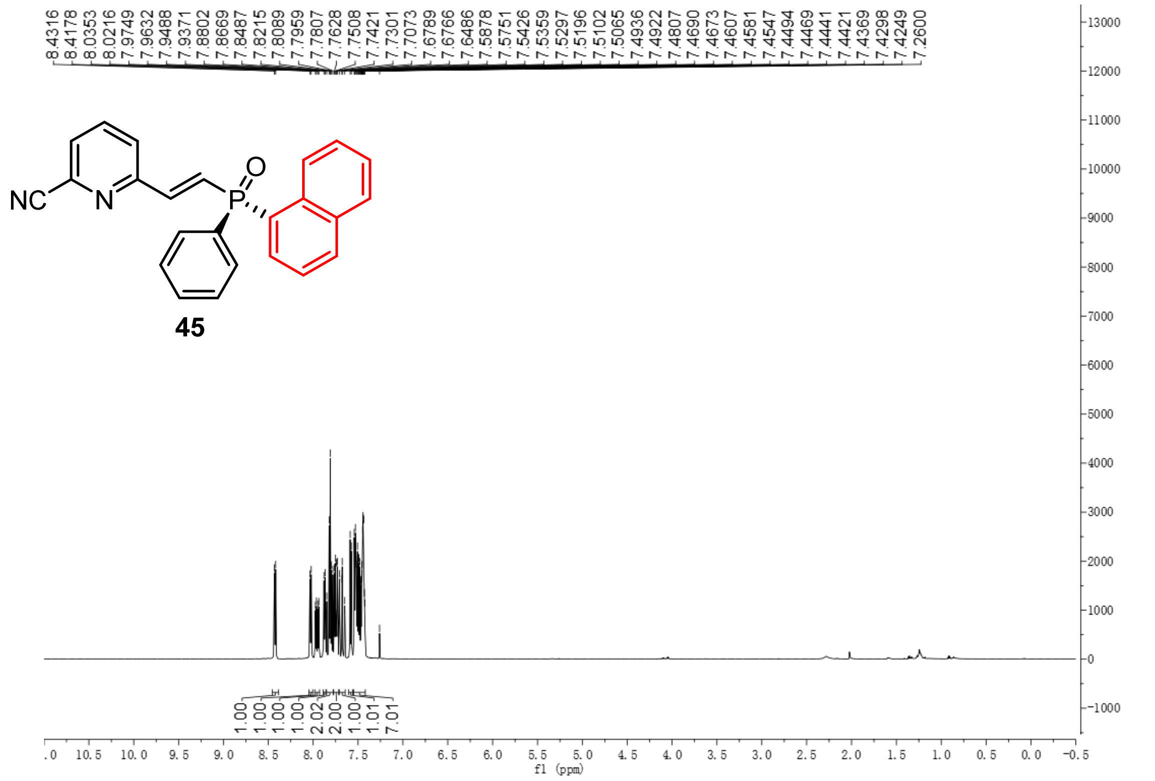

45

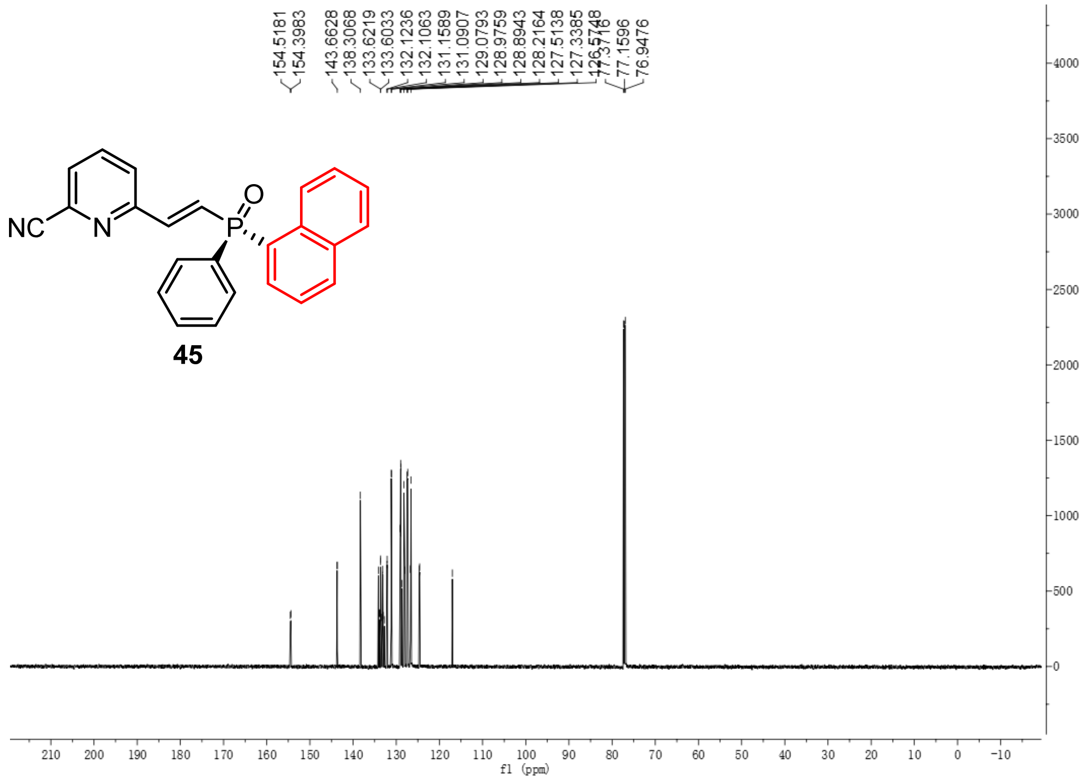

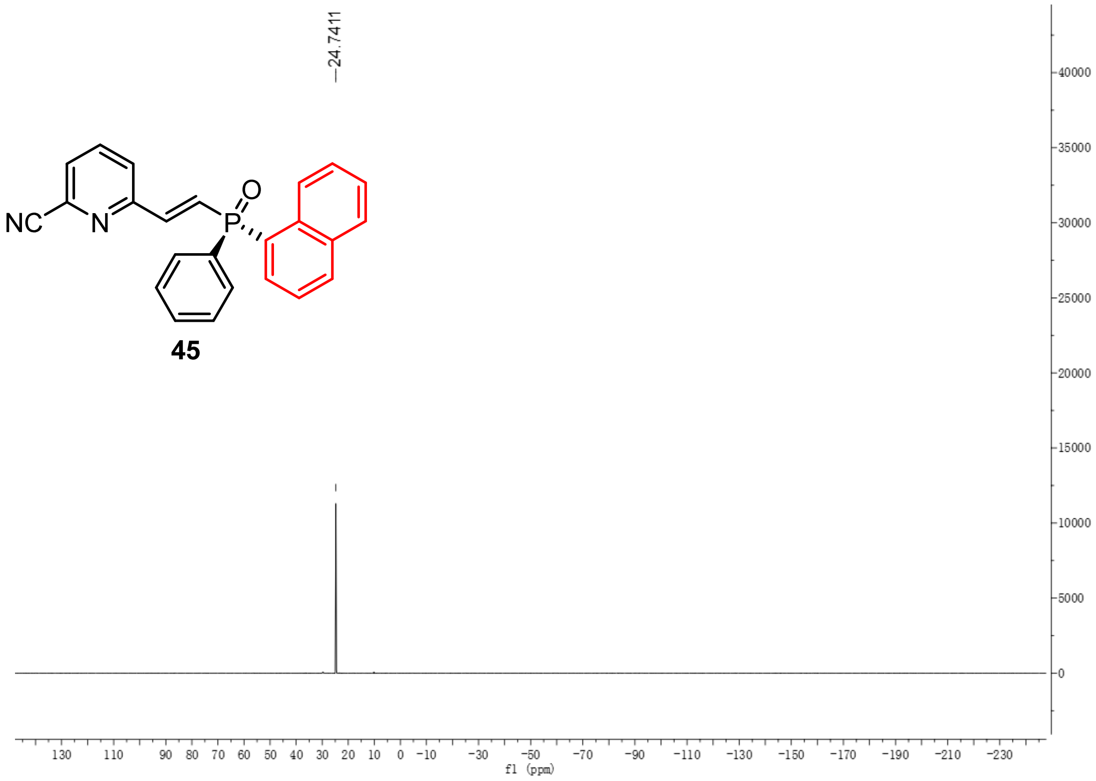

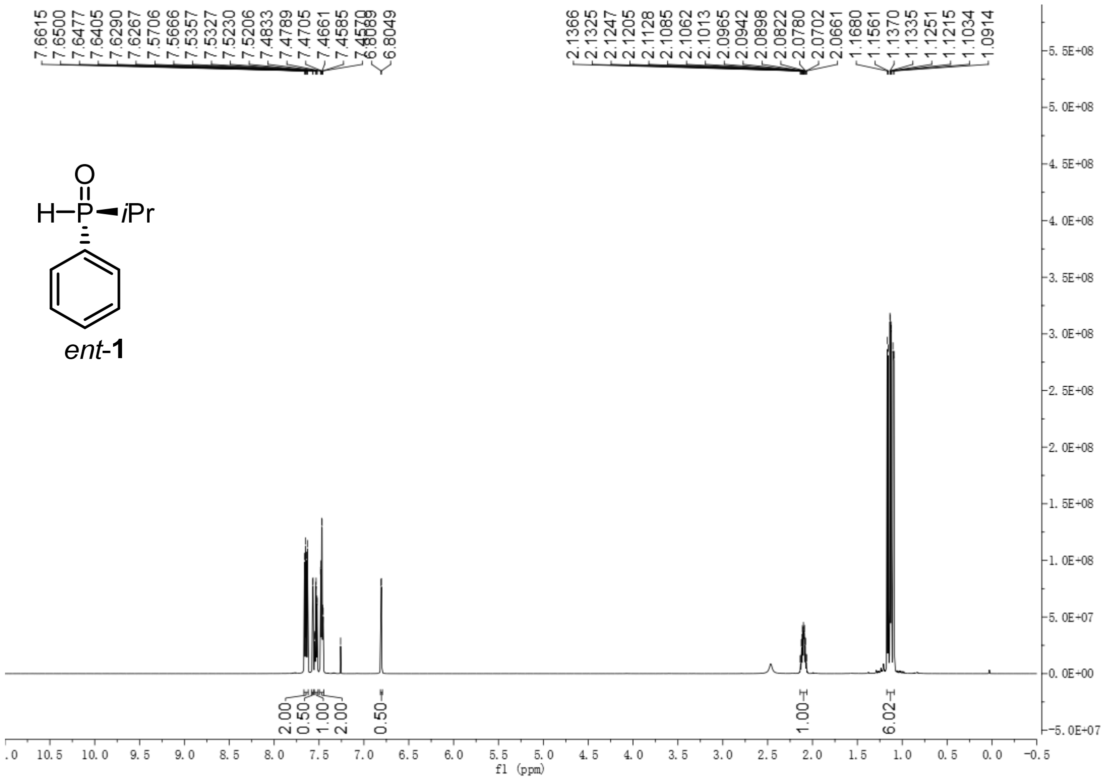

ent-1

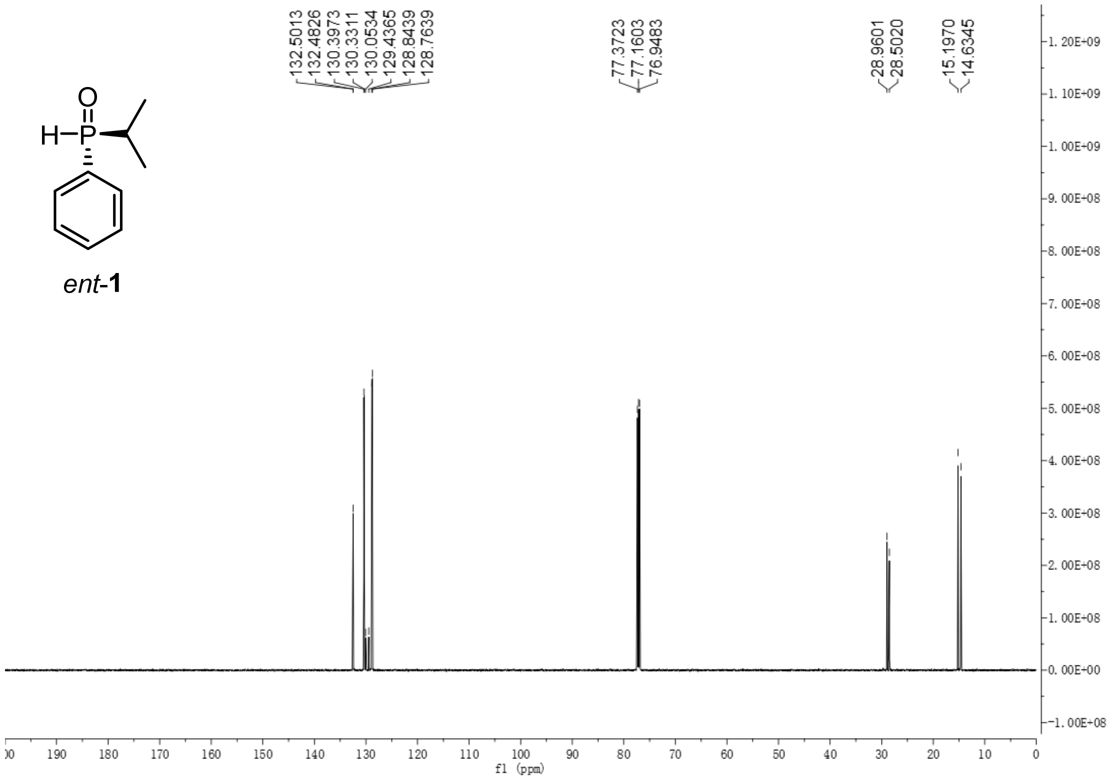

ent-1

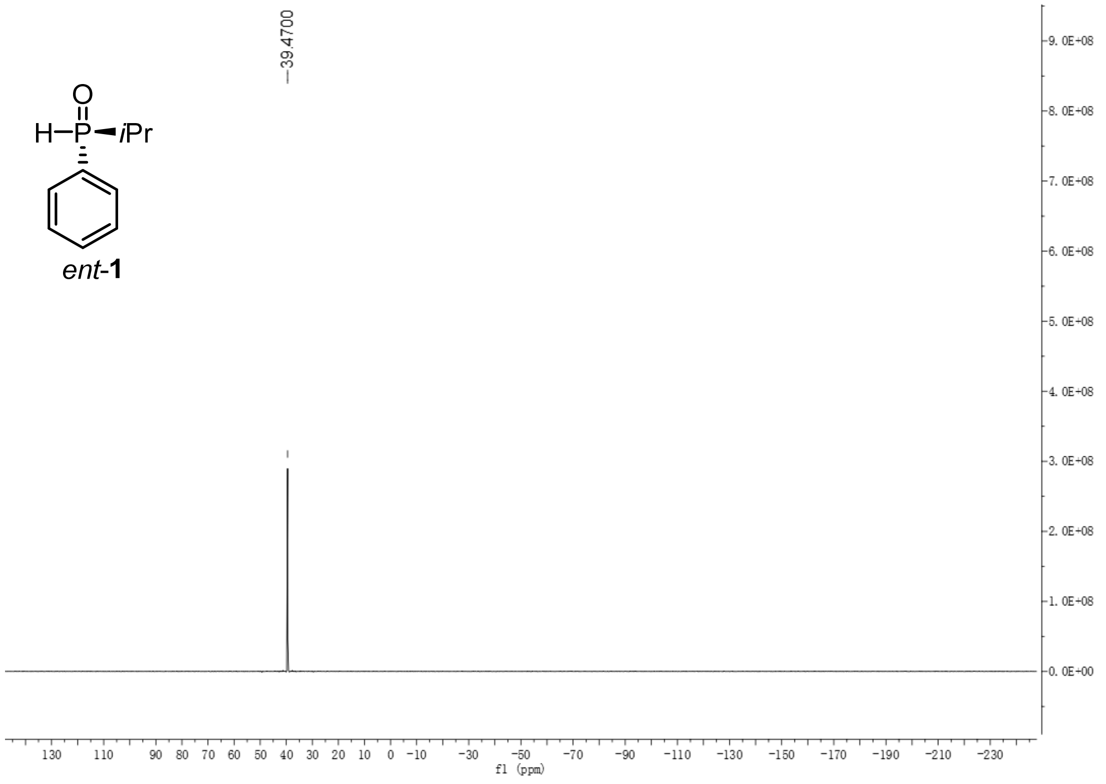

H−P(=O)−iPr
39.4700
9. 0E+08
8. 0E+08
7. 0E+08
6. 0E+08
5. 0E+08
4. 0E+08
3. 0E+08
2. 0E+08
1. 0E+08
0. 0E+00
130 110 90 80 70 60 50 40 30 20 10 0 −10 −30 −50 −70 −90 −110 −130 −150 −170 −190 −210 −230
f1 (ppm)

ent-**1**

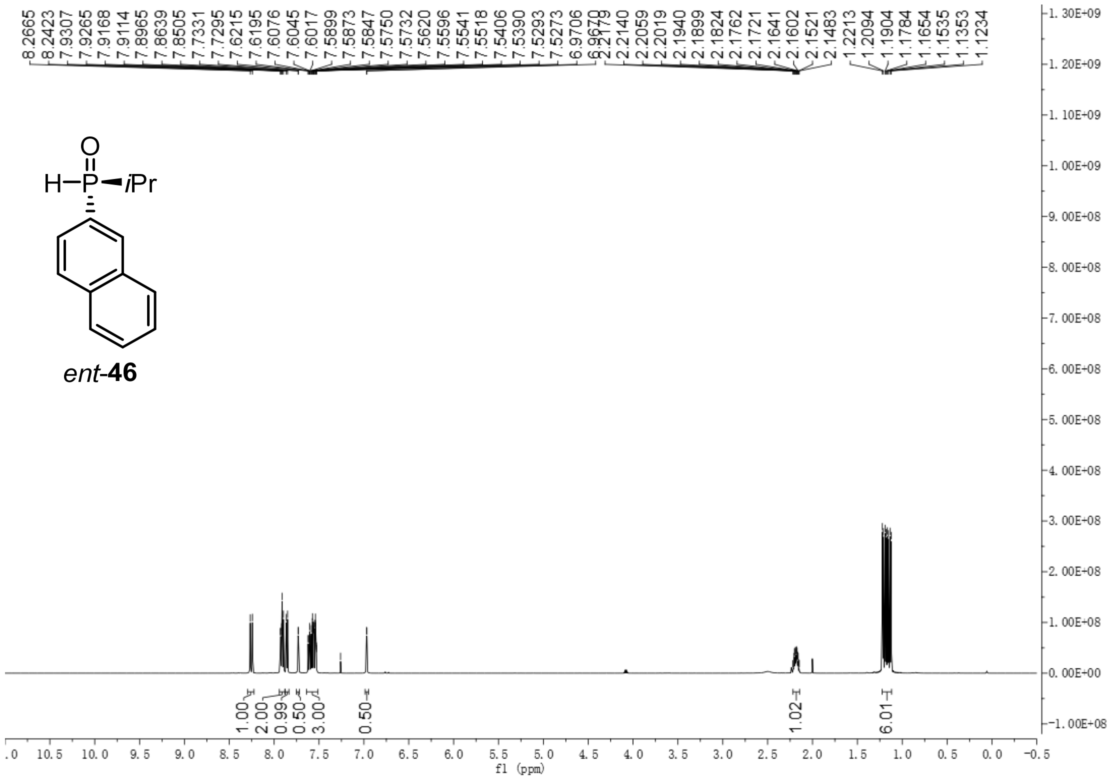

H−P(=O)−iPr
8.2665 8.2423 7.9307 7.9265 7.9168 7.9114 7.8965 7.8639 7.8505 7.7331 7.7295 7.6215 7.6195 7.6076 7.6045 7.6017 7.5899 7.5873 7.5847 7.5750 7.5732 7.5620 7.5596 7.5541 7.5518 7.5406 7.5390 7.5293 7.5273 6.9706 6.9670
2.2179 2.2140 2.2059 2.2019 2.1940 2.1899 2.1824 2.1762 2.1721 2.1641 2.1602 2.1521 2.1483
1.2213 1.2094 1.1904 1.1784 1.1654 1.1535 1.1353 1.1234
1.00 2.00 0.99 0.50 3.00 0.50 1.02 6.01
1. 30E+09
1. 20E+09
1. 10E+09
1. 00E+09
9. 00E+08
8. 00E+08
7. 00E+08
6. 00E+08
5. 00E+08
4. 00E+08
3. 00E+08
2. 00E+08
1. 00E+08
0. 00E+00
−1. 00E+08
.0 10.5 10.0 9.5 9.0 8.5 8.0 7.5 7.0 6.5 6.0 5.5 5.0 4.5 4.0 3.5 3.0 2.5 2.0 1.5 1.0 0.5 0.0 −0.5
f1 (ppm)

ent-**46**

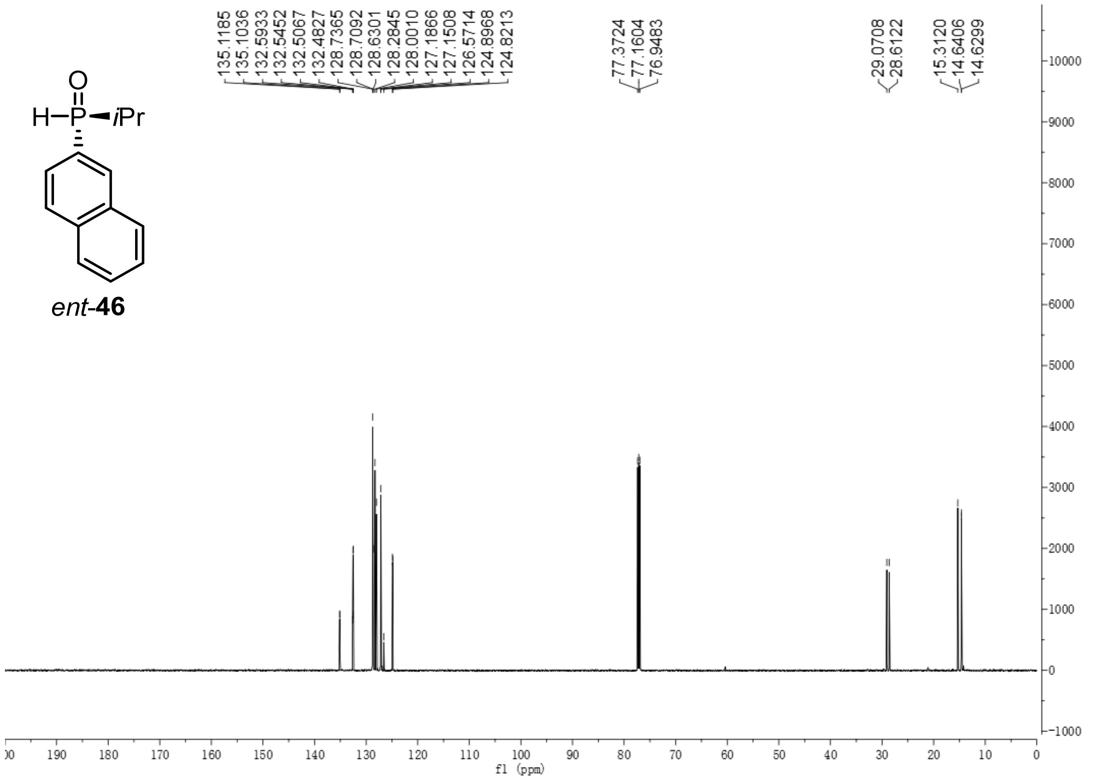

H–P(=O)–iPr
ent-46
135.1185
135.1036
132.5933
132.5452
132.5067
132.4827
128.7365
128.7092
128.6301
128.2845
128.0010
127.1866
127.1508
126.5714
124.8968
124.8213
77.3724
77.1604
76.9483
29.0708
28.6122
15.3120
14.6406
14.6299
f1 (ppm)

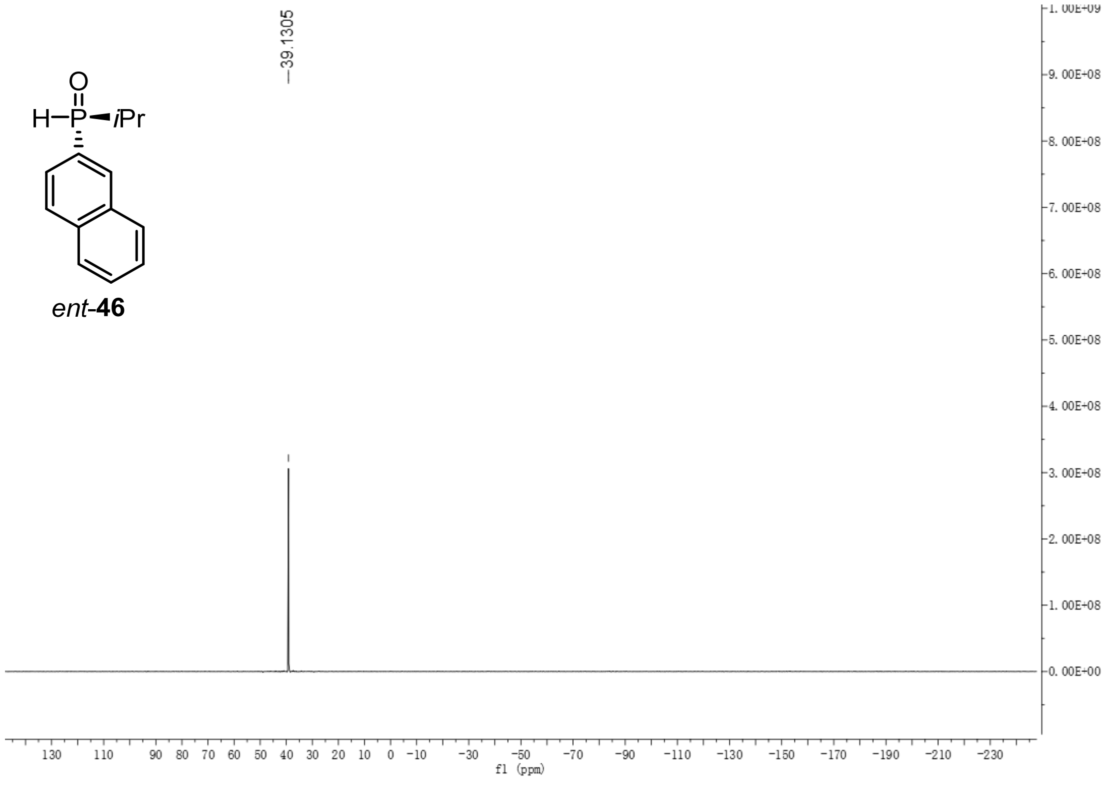

H–P(=O)–iPr
ent-46
39.1305
f1 (ppm)

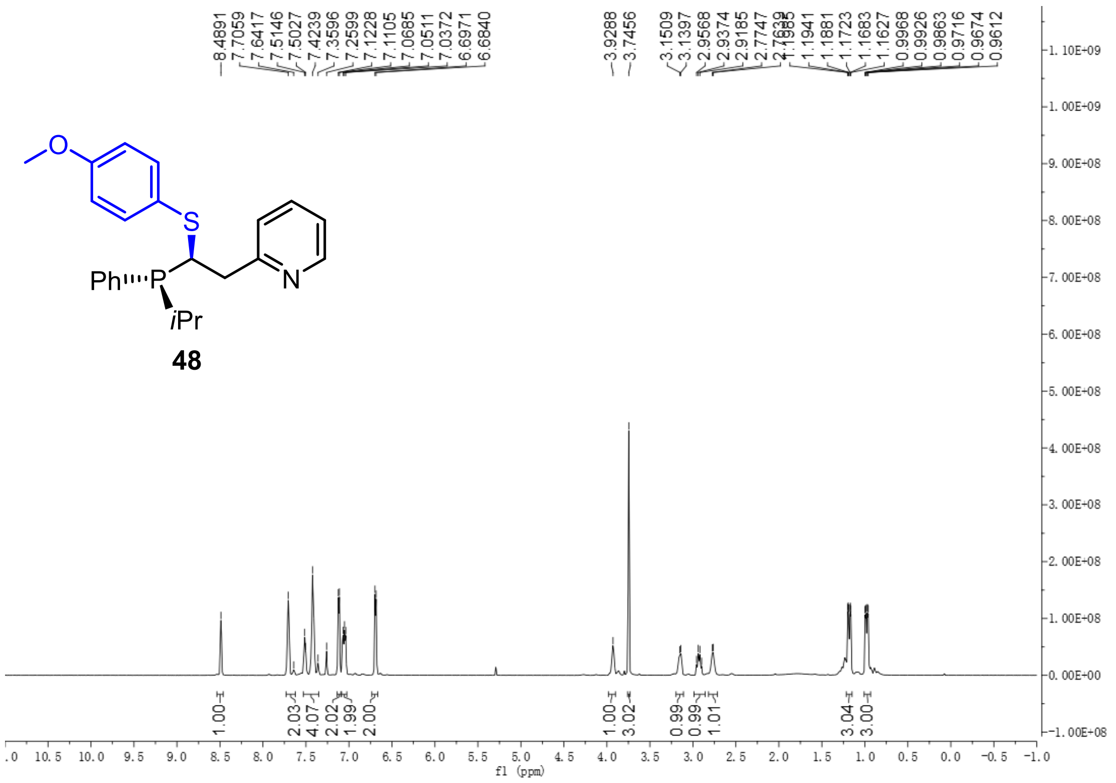

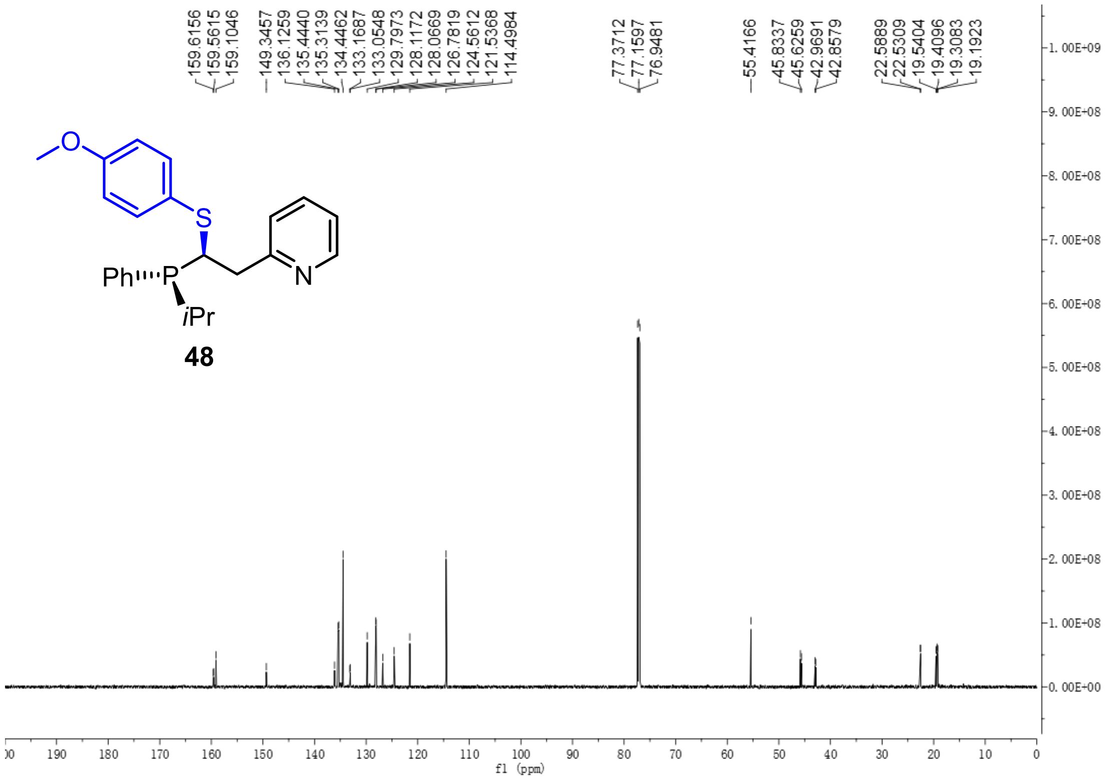

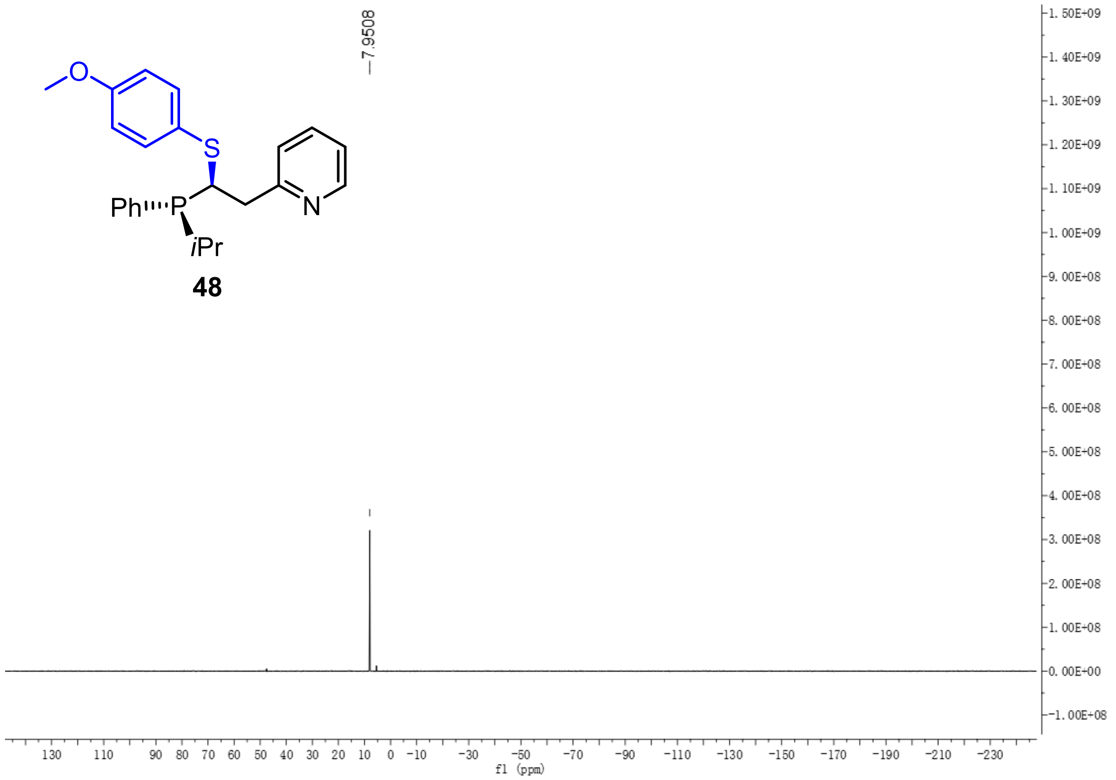

Ph
P
S
N
O
iPr
−7.9508
f1 (ppm)

48

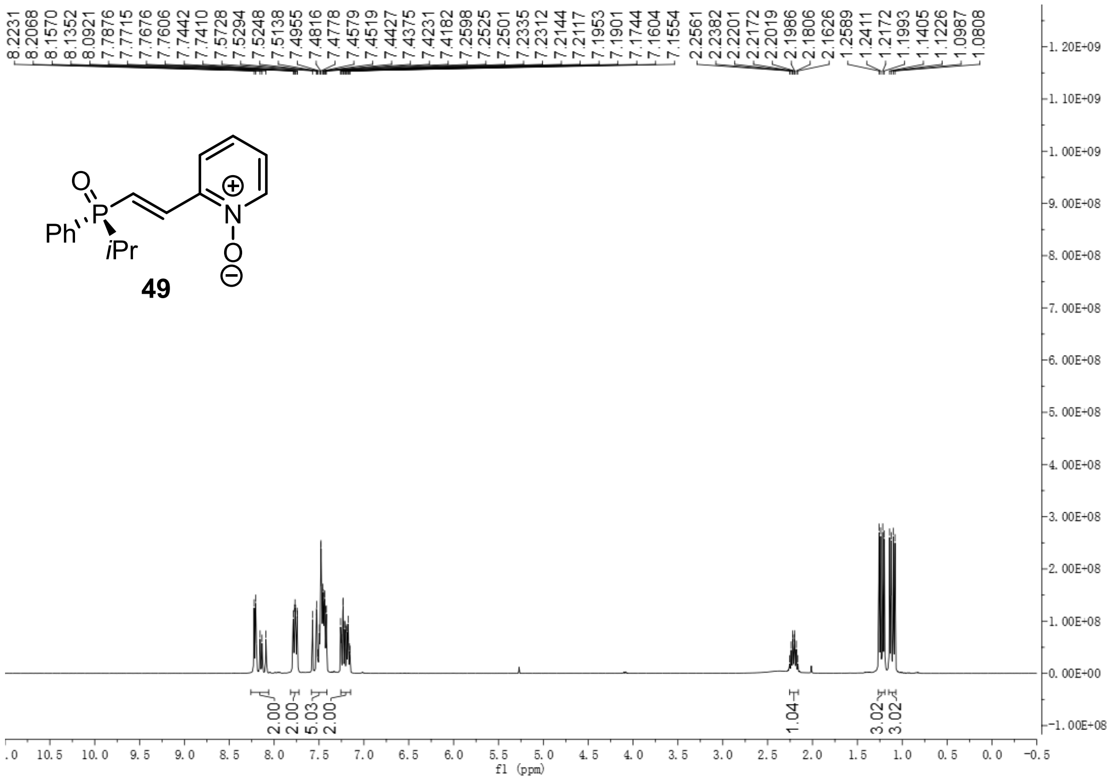

O
P
Ph
iPr
N
O
8.2231
8.2068
8.1570
8.1352
8.0921
7.7876
7.7715
7.7676
7.7606
7.7442
7.7410
7.5728
7.5294
7.5248
7.5138
7.4955
7.4816
7.4778
7.4579
7.4519
7.4427
7.4375
7.4231
7.4182
7.2598
7.2525
7.2501
7.2335
7.2312
7.2144
7.2117
7.1953
7.1901
7.1744
7.1604
7.1554
2.2561
2.2382
2.2201
2.2172
2.2019
2.1986
2.1806
2.1626
1.2589
1.2411
1.2172
1.1993
1.1405
1.1226
1.0987
1.0808
2.00
2.00
5.03
2.00
1.04
3.02
3.02
f1 (ppm)

49

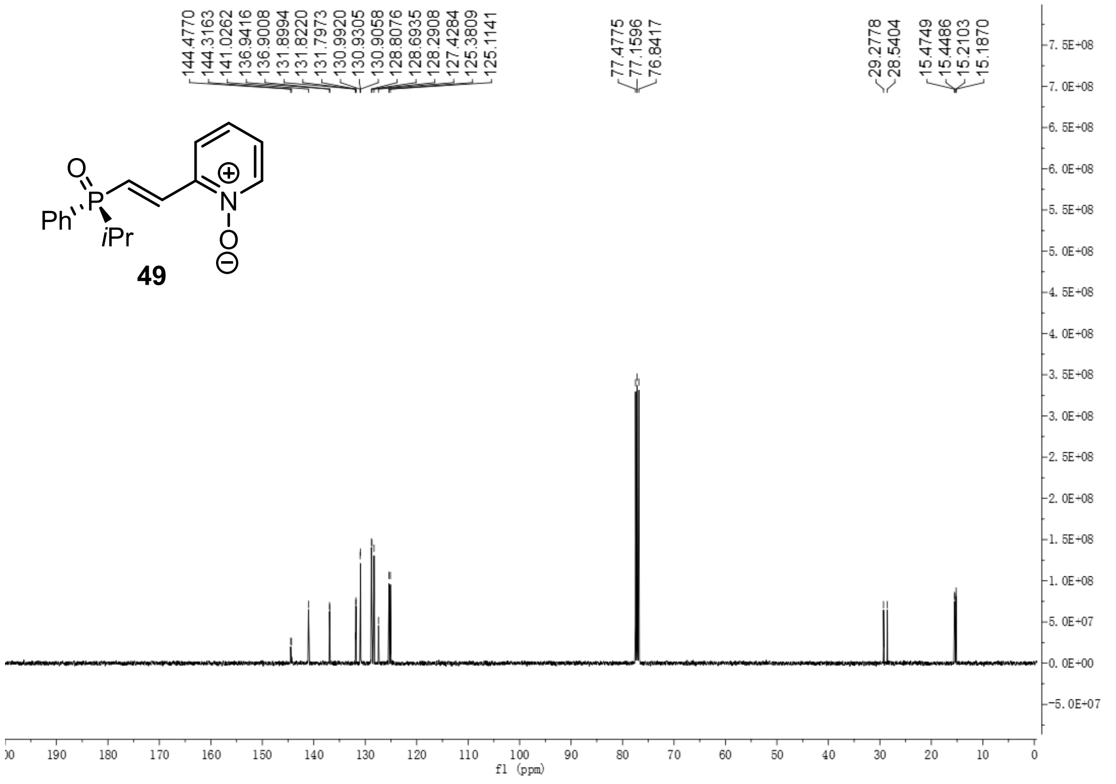
144.4770
144.3163
141.0262
136.9416
136.9008
131.8994
131.8220
131.7973
130.9920
130.9305
130.9058
128.8076
128.6935
128.2908
127.4284
125.3809
125.1141
77.4775
77.1596
76.8417
29.2778
28.5404
15.4749
15.4486
15.2103
15.1870
Ph
O
P
iPr
N
O
49
fl (ppm)

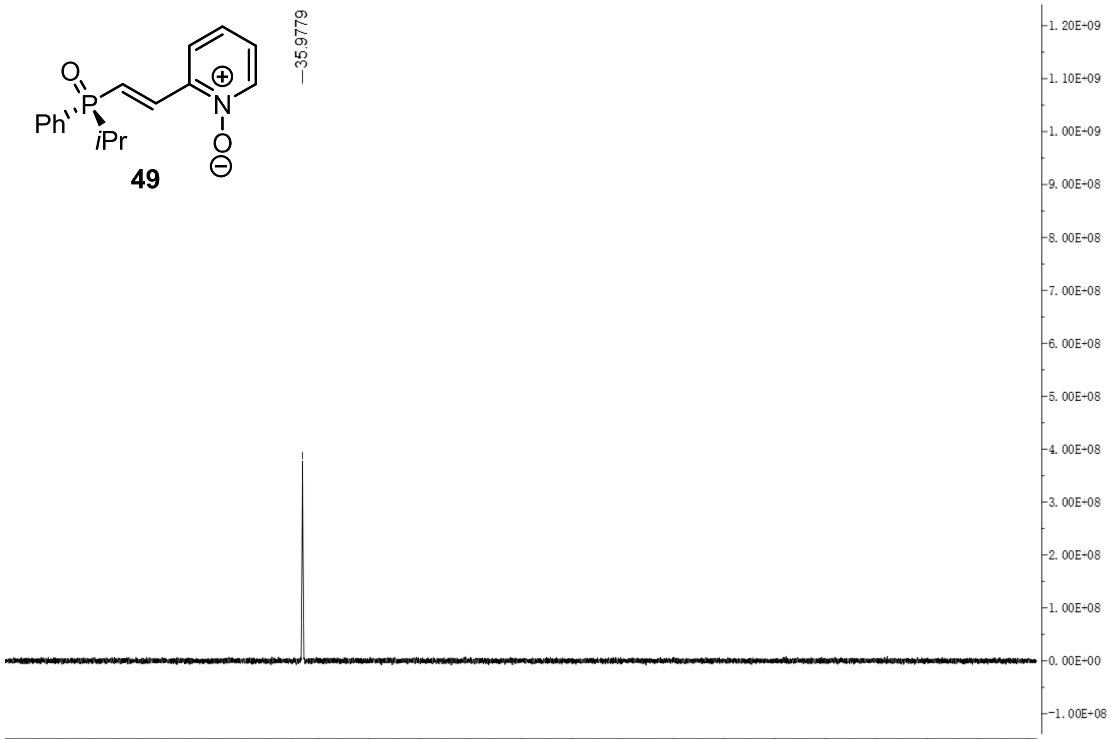
35.9779
Ph
O
P
iPr
N
O
49
fl (ppm)

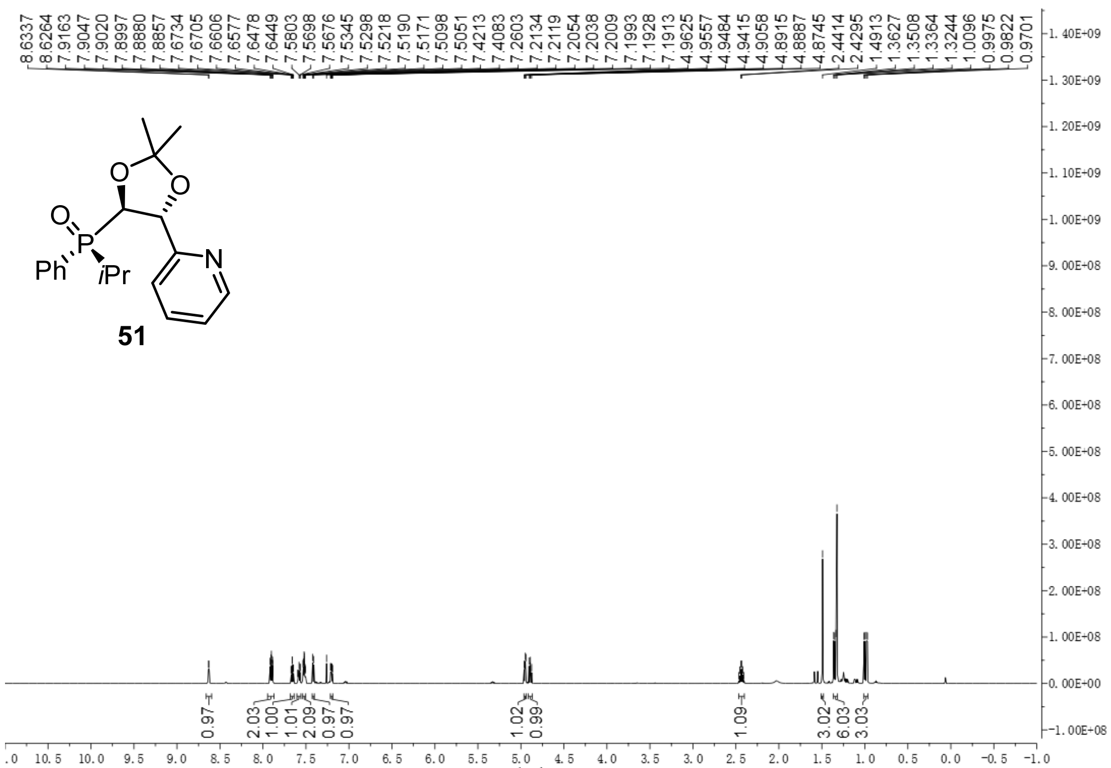
8.6337
8.6264
7.9163
7.9047
7.9020
7.8997
7.8880
7.8857
7.6734
7.6705
7.6606
7.6577
7.6478
7.6449
7.5803
7.5698
7.5676
7.5345
7.5298
7.5218
7.5190
7.5171
7.5098
7.5051
7.4213
7.4083
7.2603
7.2134
7.2119
7.2054
7.2038
7.2009
7.1993
7.1928
7.1913
4.9625
4.9557
4.9484
4.9415
4.9058
4.8915
4.8887
4.8745
2.4414
2.4295
1.4913
1.3627
1.3508
1.3364
1.3244
1.0096
0.9975
0.9822
0.9701
Ph
iPr
51
0.97
2.03
1.00
1.01
2.09
0.97
0.97
1.02
0.99
1.09
3.02
6.03
3.03
f1 (ppm)

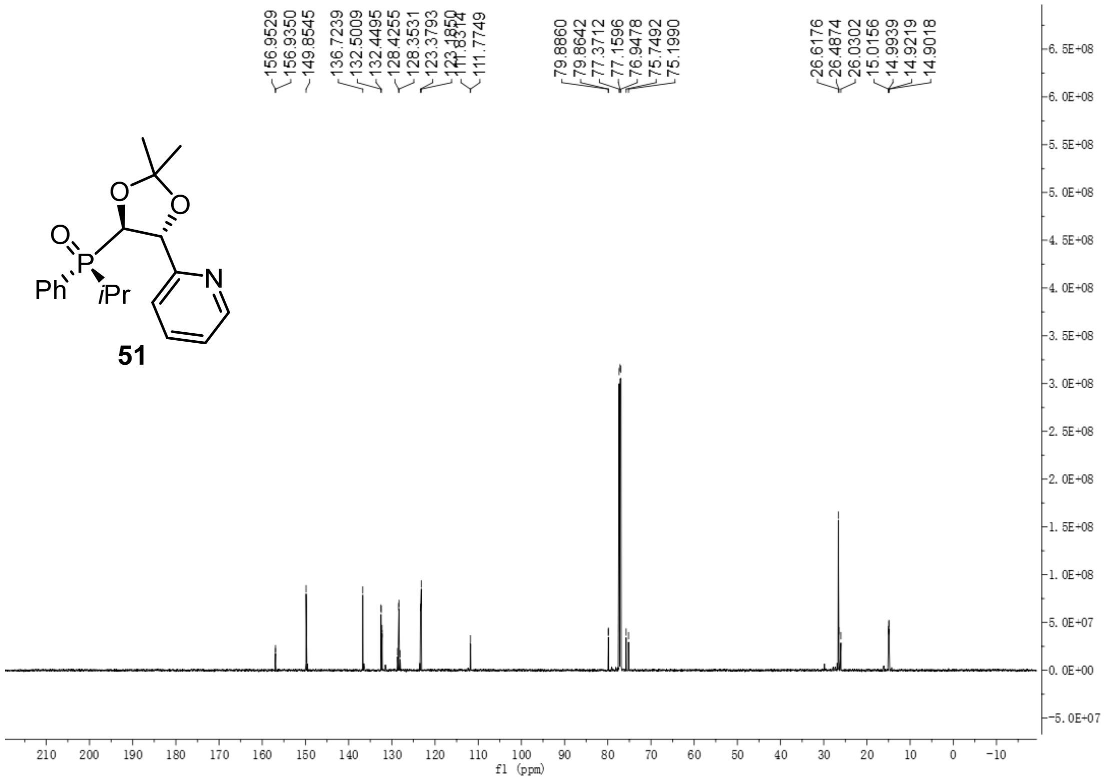
156.9529
156.9350
149.8545
136.7239
132.5009
132.4495
128.4255
128.3531
123.3793
123.1850
111.7749
79.8860
79.8642
77.3712
77.1596
76.9478
75.7492
75.1990
26.6176
26.4874
26.0302
15.0156
14.9939
14.9219
14.9018
Ph
iPr
51
f1 (ppm)

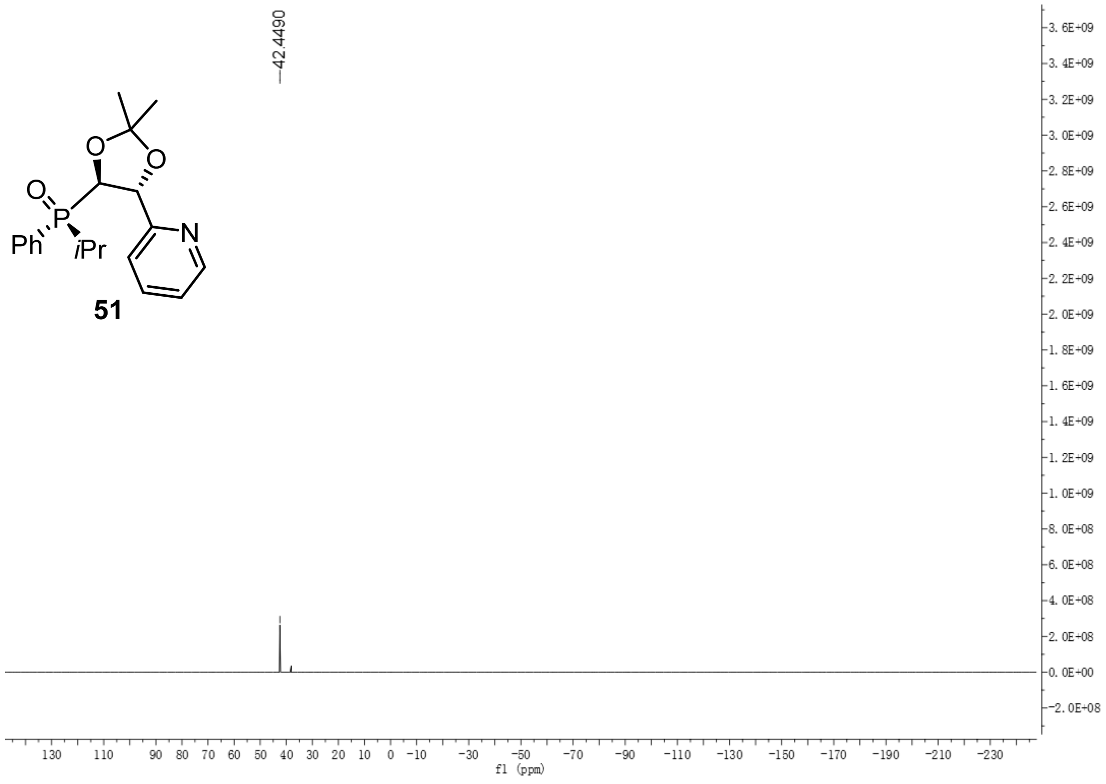

42.4490
51
Ph
iPr
fl (ppm)

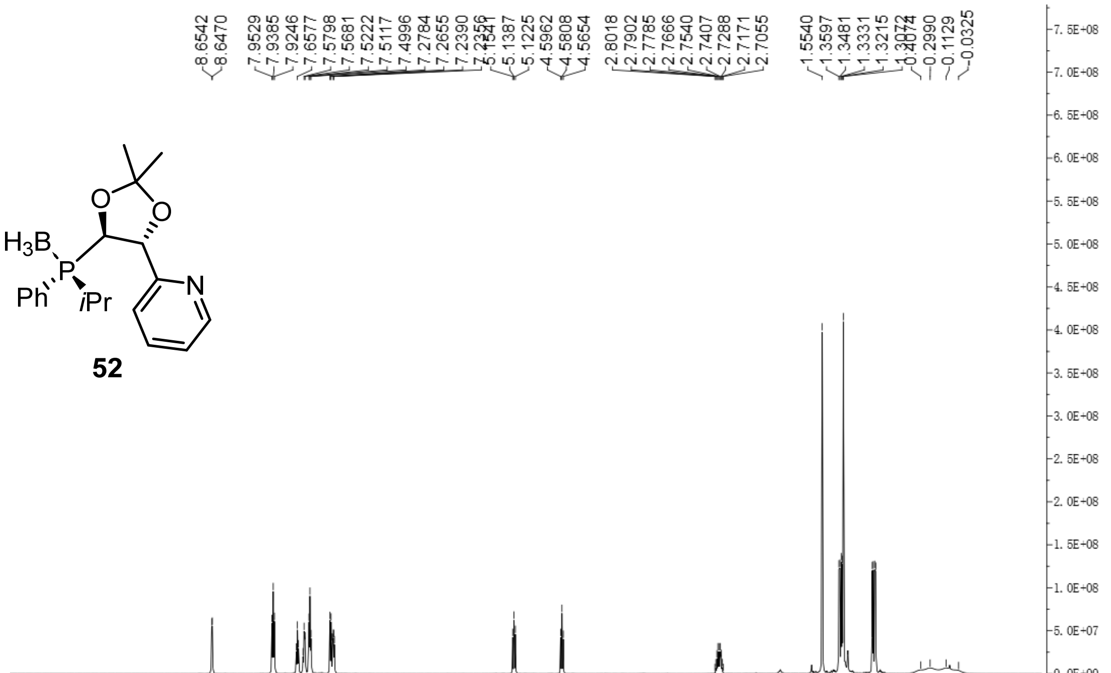

8.6542
8.6470
7.9529
7.9385
7.9246
7.6577
7.5798
7.5681
7.5222
7.5117
7.4996
7.2784
7.2655
7.2390
5.1387
5.1225
4.5962
4.5808
4.5654
2.8018
2.7902
2.7785
2.7666
2.7540
2.7407
2.7288
2.7171
2.7055
1.5540
1.3597
1.3481
1.3331
1.3215
0.2990
0.1129
0.0325
52
Ph
iPr
fl (ppm)

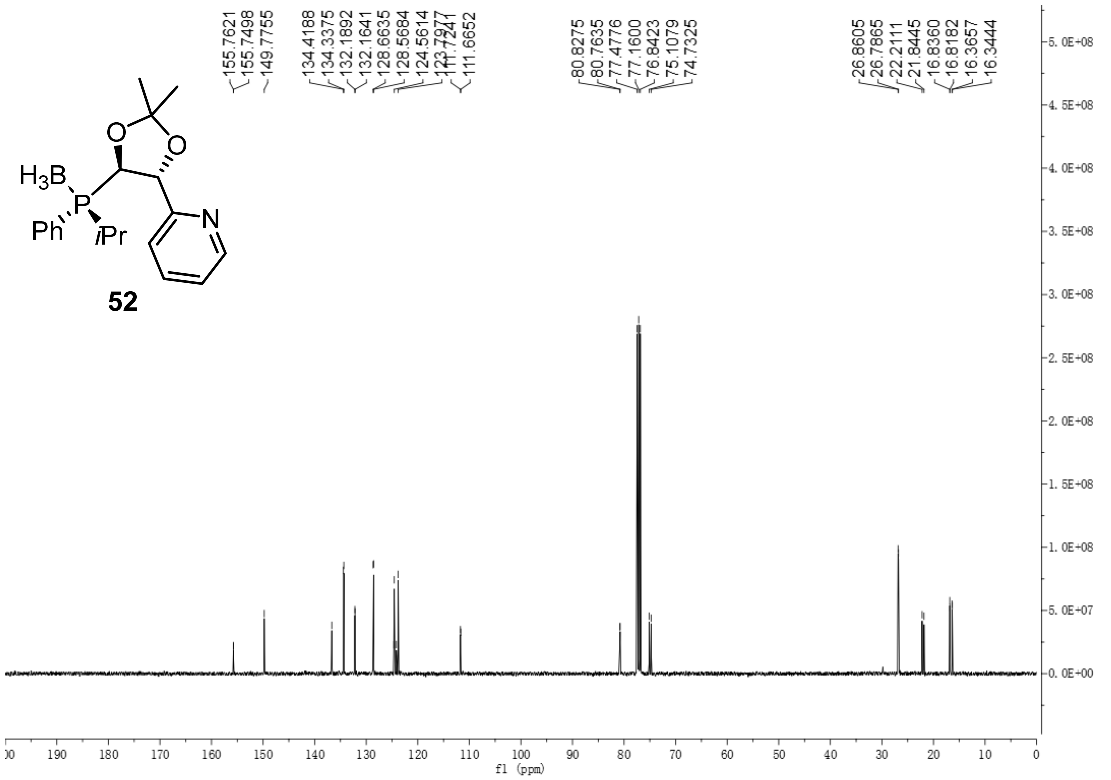

155.7621
155.7498
149.7755
134.4188
134.3375
132.1892
132.1641
128.6635
128.5684
124.5614
111.6652
80.8275
80.7635
77.4776
77.1600
76.8423
75.1079
74.7325
26.8605
26.7865
22.2111
21.8445
16.8360
16.8182
16.3657
16.3444
f1 (ppm)

52

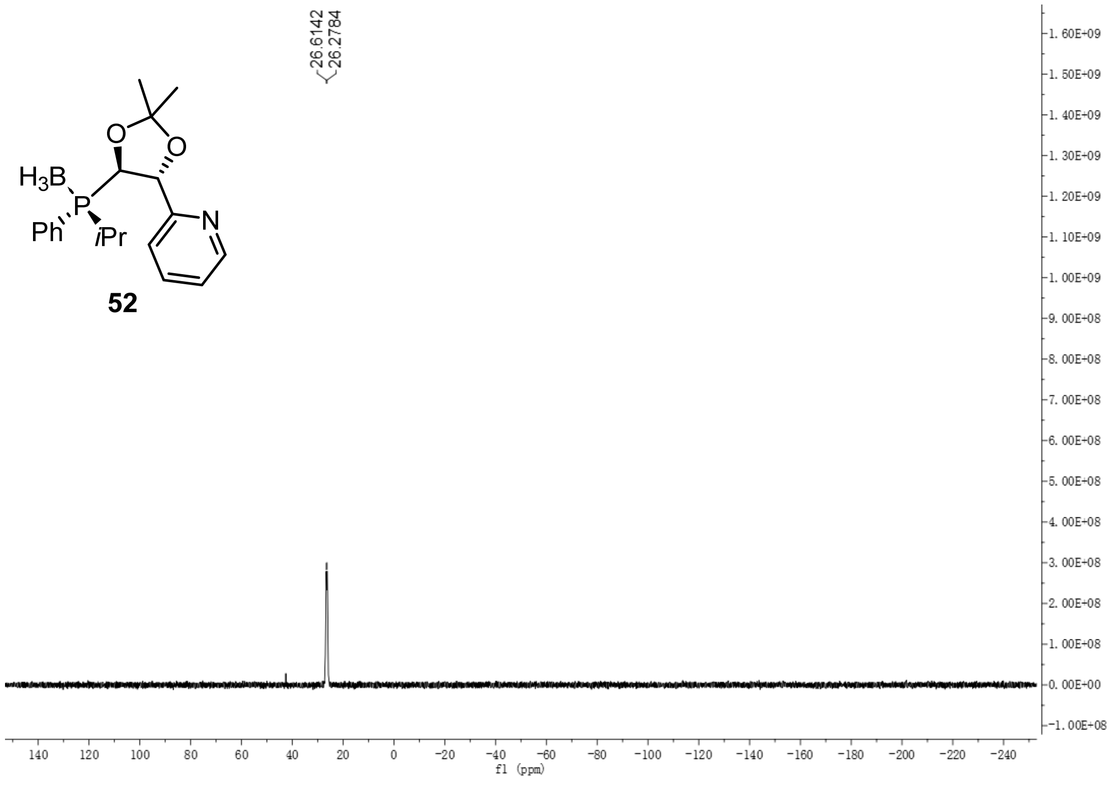

26.6142
26.2784
f1 (ppm)

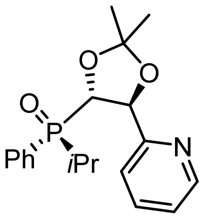
O
P
Ph
iPr
O
O
N

54

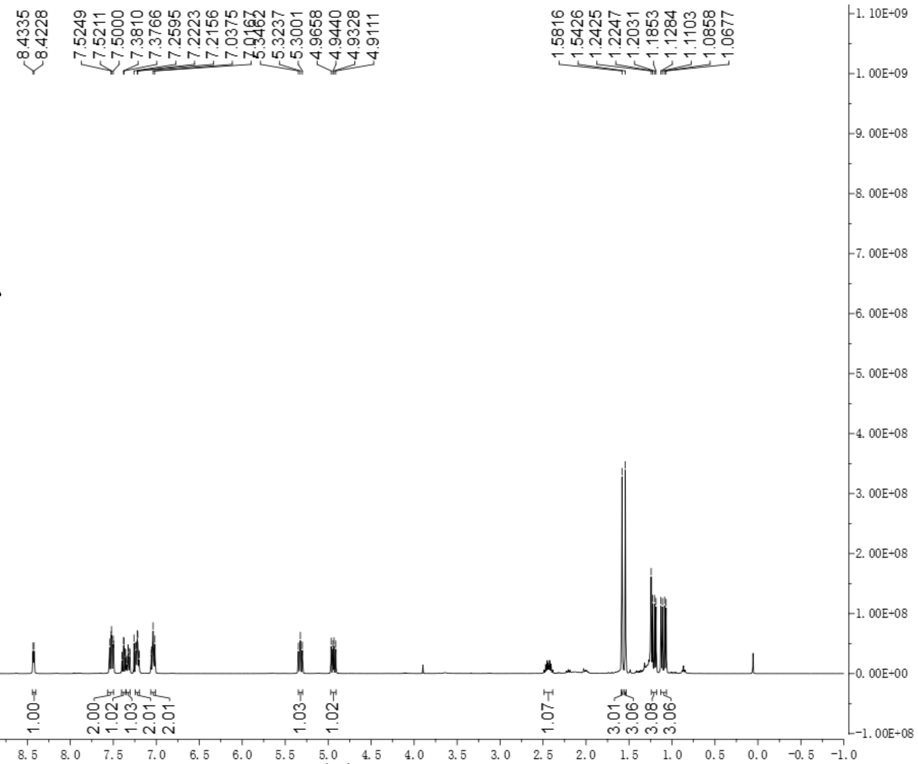
8.4335
8.4228
7.5249
7.5211
7.5000
7.3810
7.3766
7.2595
7.2223
7.2156
7.0375
5.3237
5.3001
4.9658
4.9440
4.9328
4.9111
1.5816
1.5426
1.2425
1.2247
1.2031
1.1853
1.1284
1.1103
1.0858
1.0677
1.00
2.00
1.02
1.03
2.01
2.01
1.03
1.02
1.07
3.01
3.06
3.08
3.06
fl (ppm)

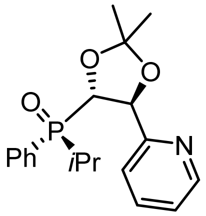
O
P
Ph
iPr
O
O
N

54

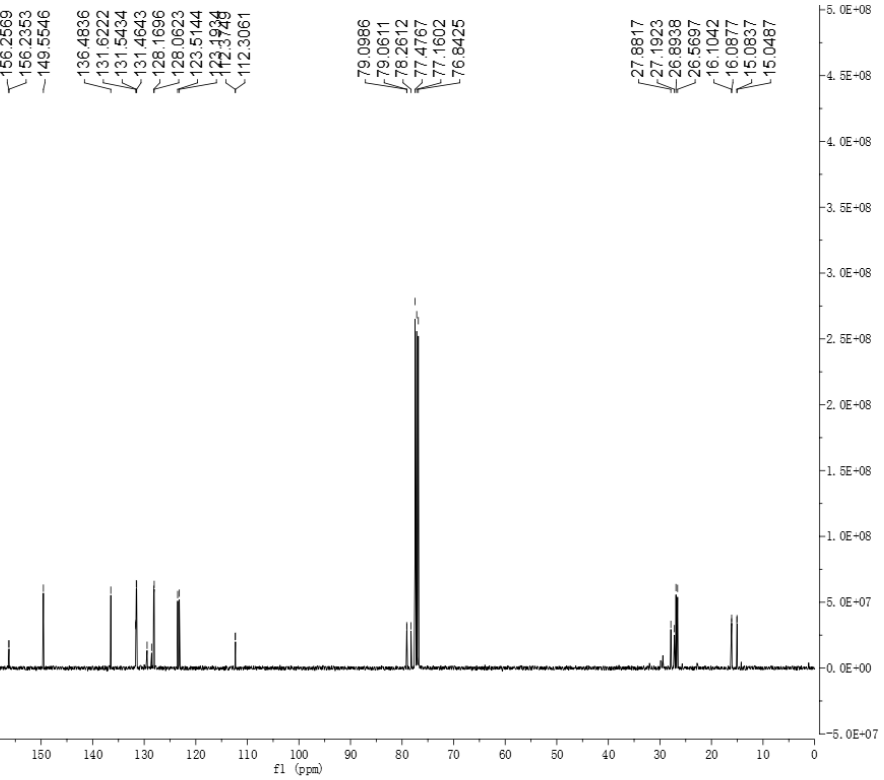
156.2569
156.2353
149.5546
136.4836
131.6222
131.5434
131.4643
128.1696
128.0623
123.5144
112.3061
79.0986
79.0611
78.2612
77.4767
77.1602
76.8425
27.8817
27.1923
26.8938
26.5697
16.1042
16.0877
15.0837
15.0487
fl (ppm)

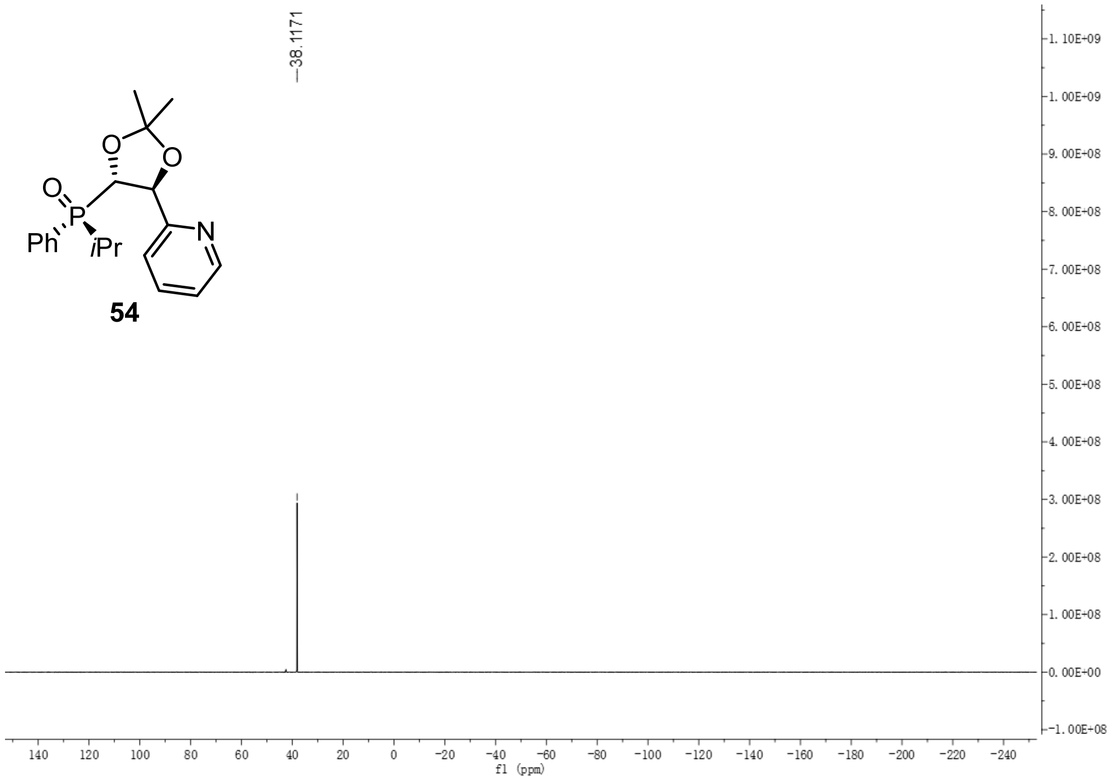

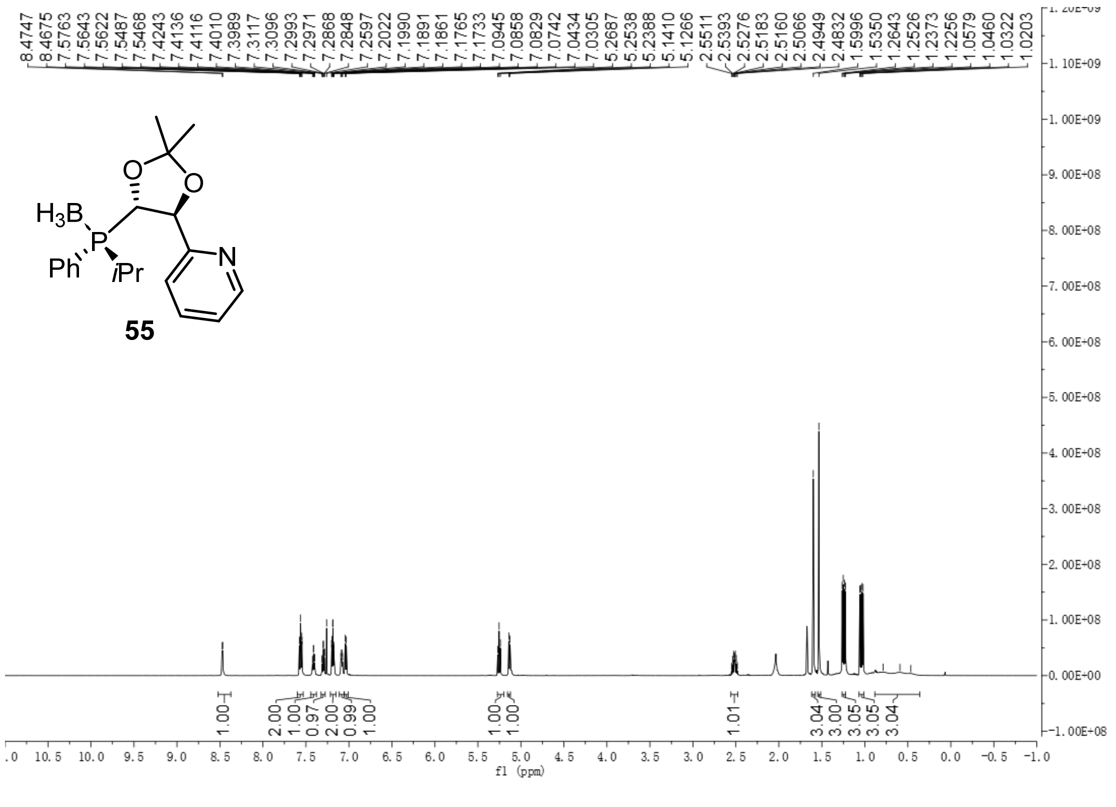

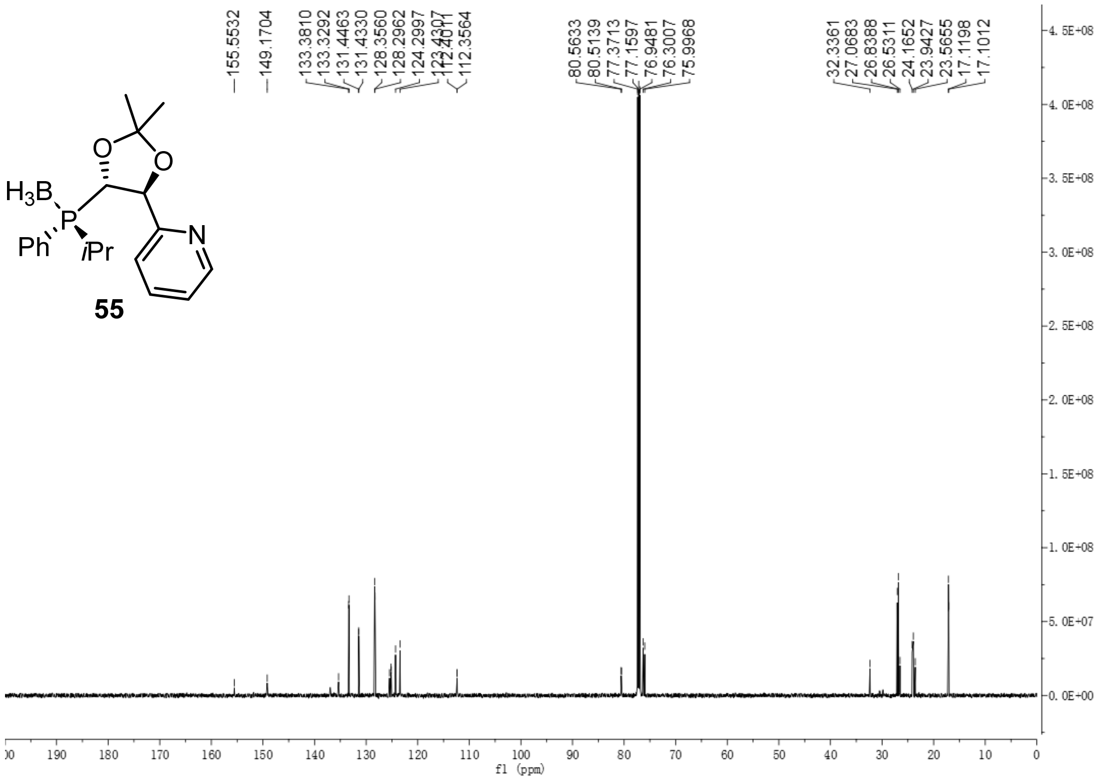
H3B
P
Ph
iPr
O
O
N
55
155.5532
149.1704
133.3810
133.3292
131.4463
131.4330
128.3560
128.2962
124.2997
112.3564
80.5633
80.5139
77.3713
77.1597
76.9481
76.3007
75.9968
32.3361
27.0683
26.8388
26.5311
24.1652
23.9427
23.5655
17.1198
17.1012
f1 (ppm)

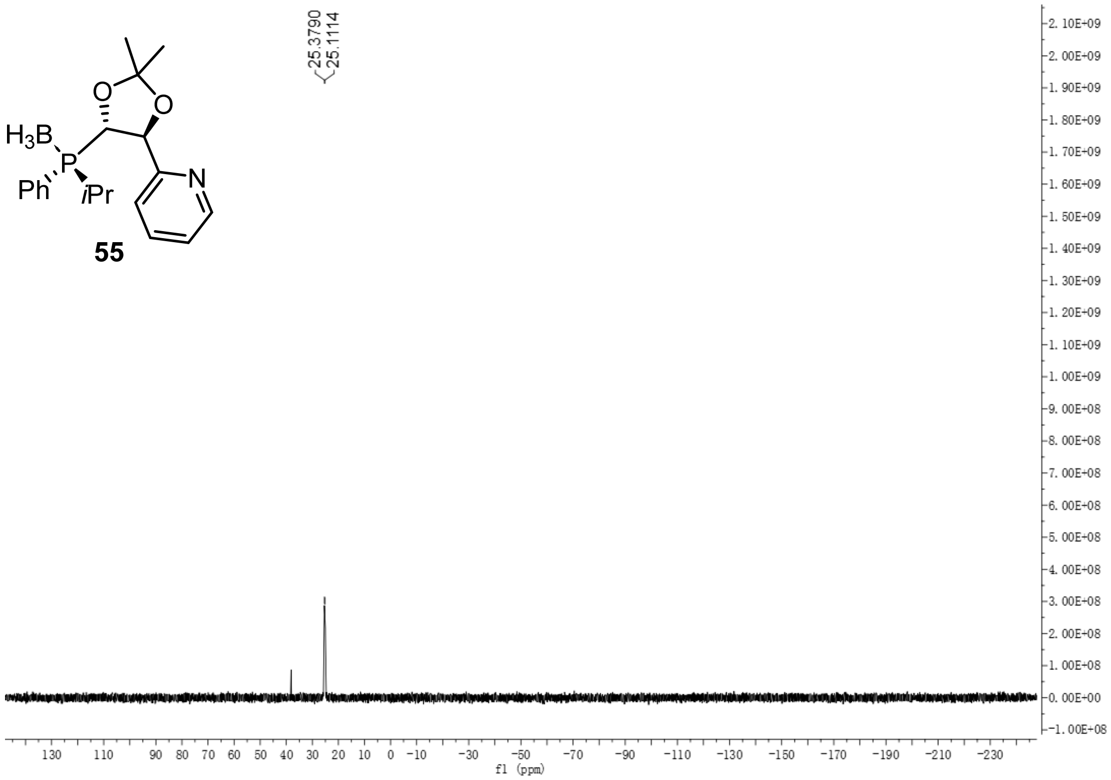
H3B
P
Ph
iPr
O
O
N
55
25.3790
25.1114
f1 (ppm)

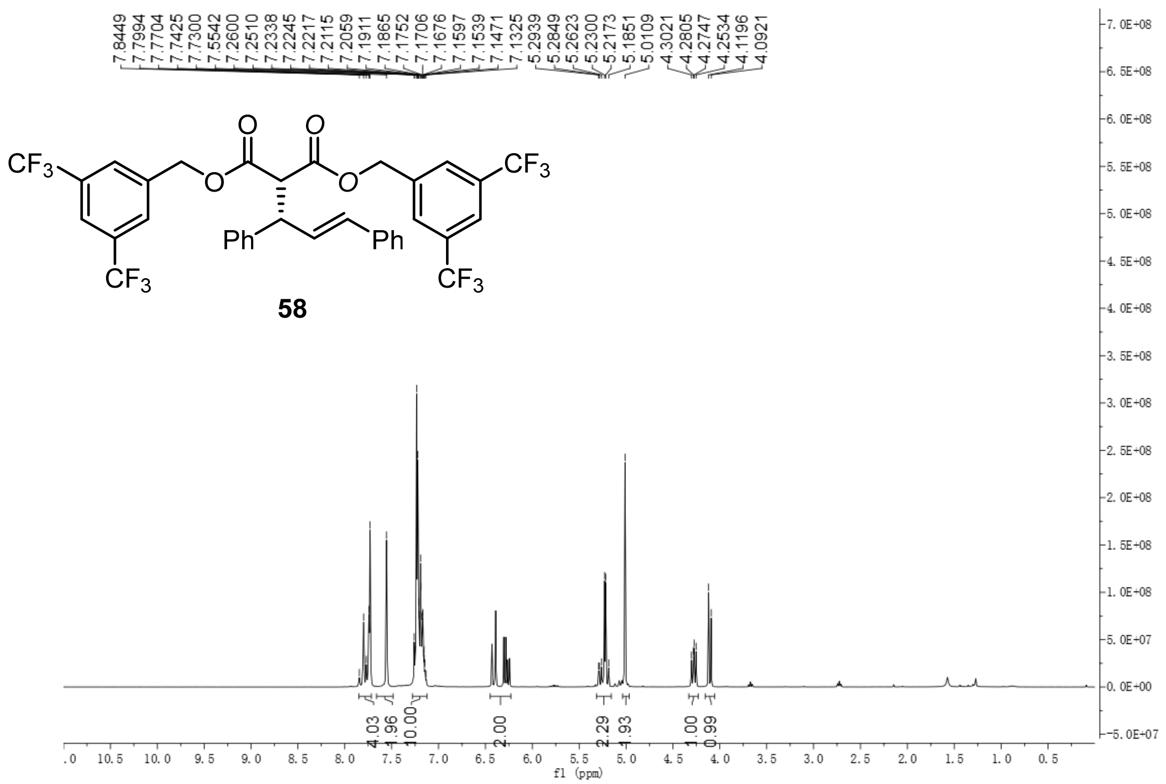
7.8449
7.7994
7.7704
7.7425
7.7300
7.5542
7.2600
7.2510
7.2338
7.2245
7.2217
7.2115
7.2059
7.1911
7.1865
7.1752
7.1706
7.1676
7.1597
7.1539
7.1471
7.1325
5.2939
5.2849
5.2623
5.2300
5.2173
5.1851
5.0109
4.3021
4.2805
4.2747
4.2534
4.1196
4.0921
CF3
O
O
O
O
CF3
Ph
Ph
CF3
CF3
58
4.03
4.96
10.00
2.00
2.29
4.93
1.00
0.99
f1 (ppm)

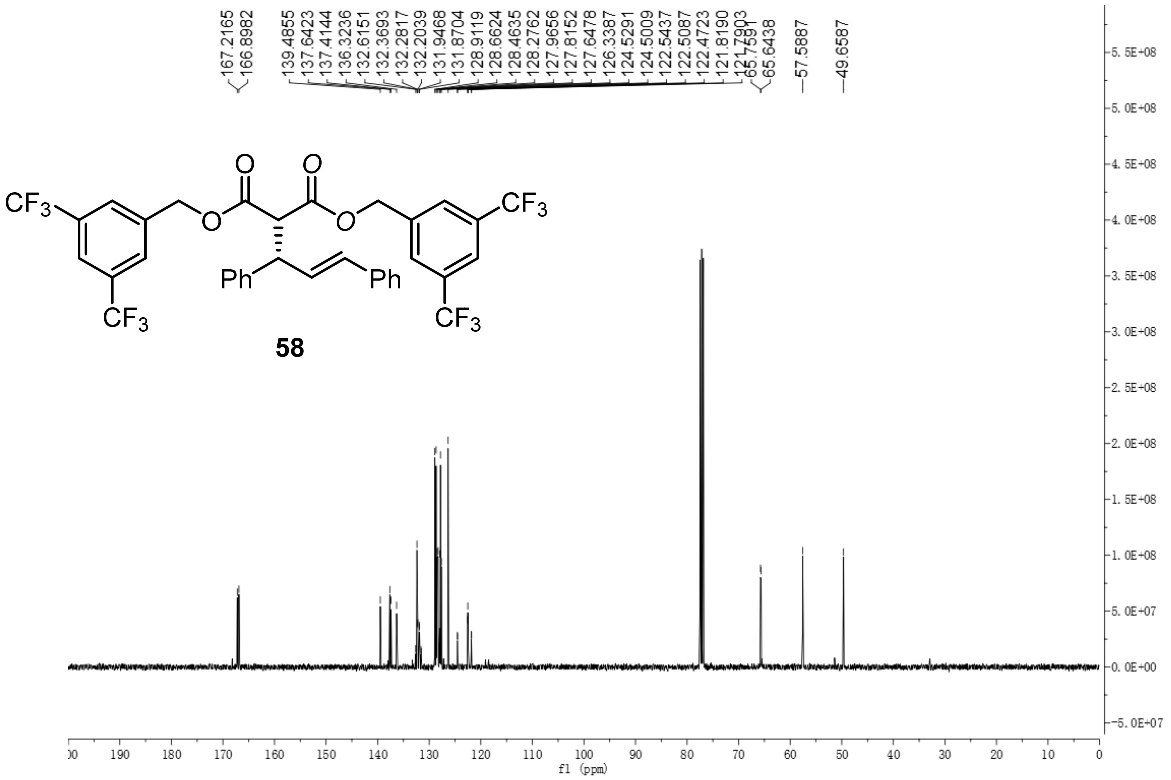
167.2165
166.8982
139.4855
137.6423
137.4144
136.3236
132.6151
132.3693
132.2817
132.2039
131.9468
131.8704
128.9119
128.6624
128.4635
128.2762
127.9656
127.8152
127.6478
126.3387
124.5291
124.5009
122.5437
122.5087
122.4723
121.8190
121.7903
65.7591
65.6438
57.5887
49.6587
CF3
O
O
O
O
CF3
Ph
Ph
CF3
CF3
58
f1 (ppm)

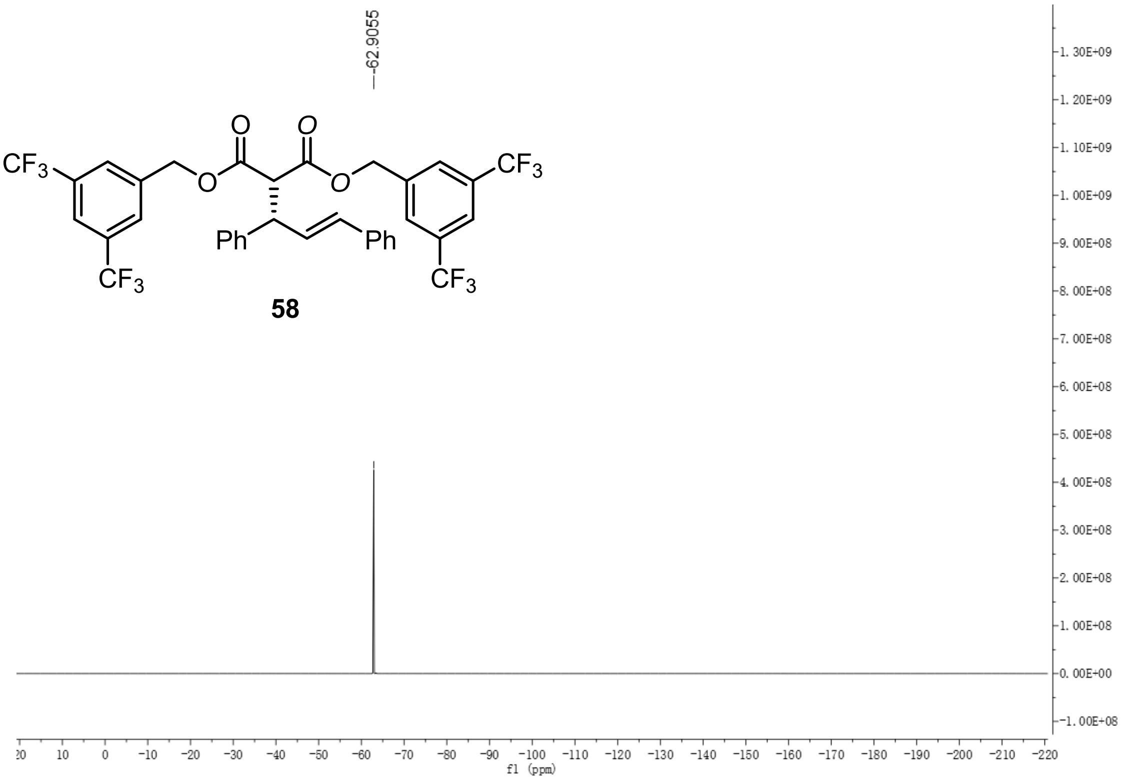

-62.9055
CF3
O
O
CF3
Ph
Ph
CF3
CF3
58
fl (ppm)

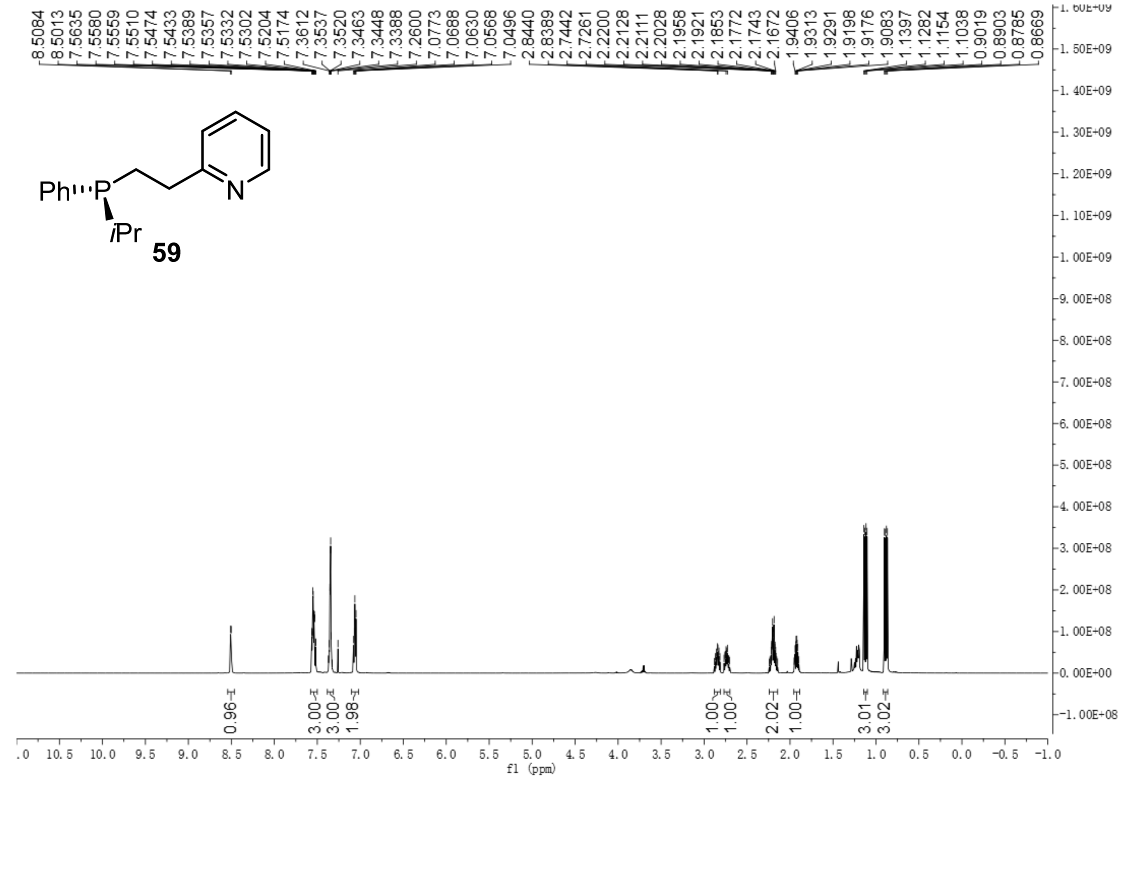

8.5084
8.5013
7.5635
7.5580
7.5559
7.5510
7.5474
7.5433
7.5389
7.5357
7.5332
7.5302
7.5204
7.5174
7.3612
7.3537
7.3520
7.3463
7.3448
7.3388
7.2600
7.0773
7.0688
7.0630
7.0568
7.0496
2.8440
2.8389
2.7442
2.7261
2.2200
2.2128
2.2111
2.2028
2.1958
2.1921
2.1853
2.1772
2.1743
2.1672
1.9406
1.9313
1.9291
1.9198
1.9176
1.9083
1.1397
1.1282
1.1154
1.1038
0.9019
0.8903
0.8785
0.8669
Ph'''P
N
iPr
59
0.96
3.00
3.00
1.98
1.00
1.00
2.02
1.00
3.01
3.02
fl (ppm)

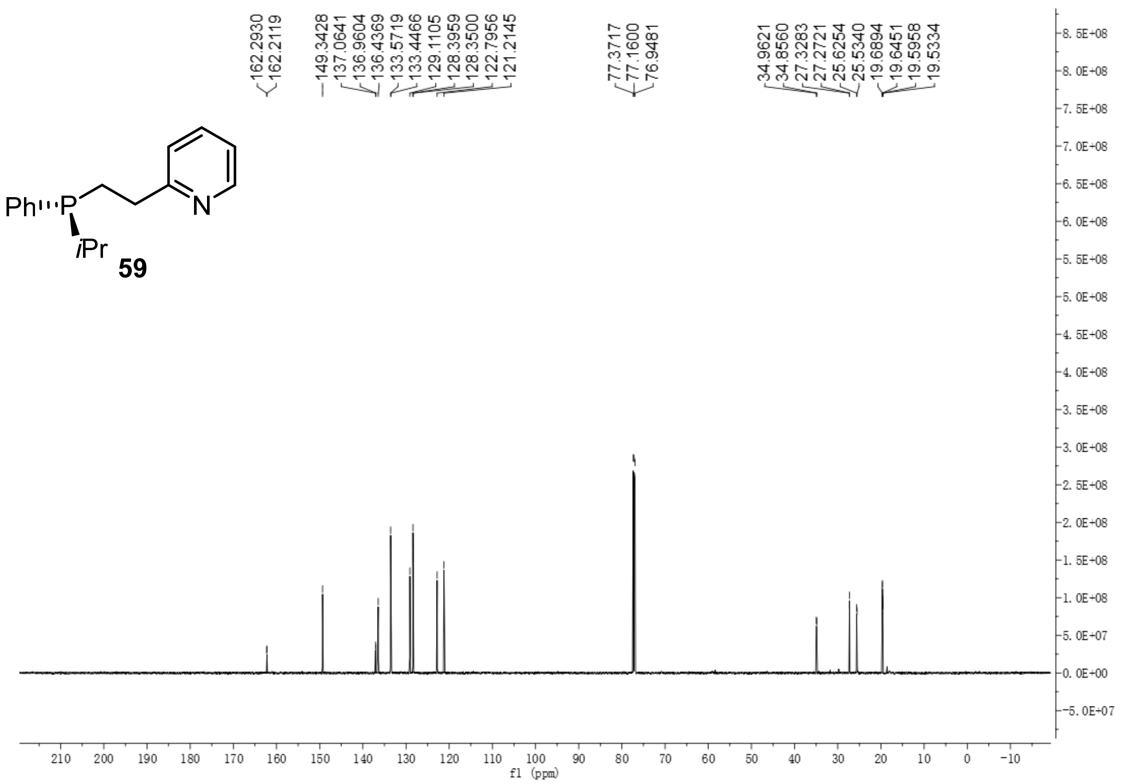

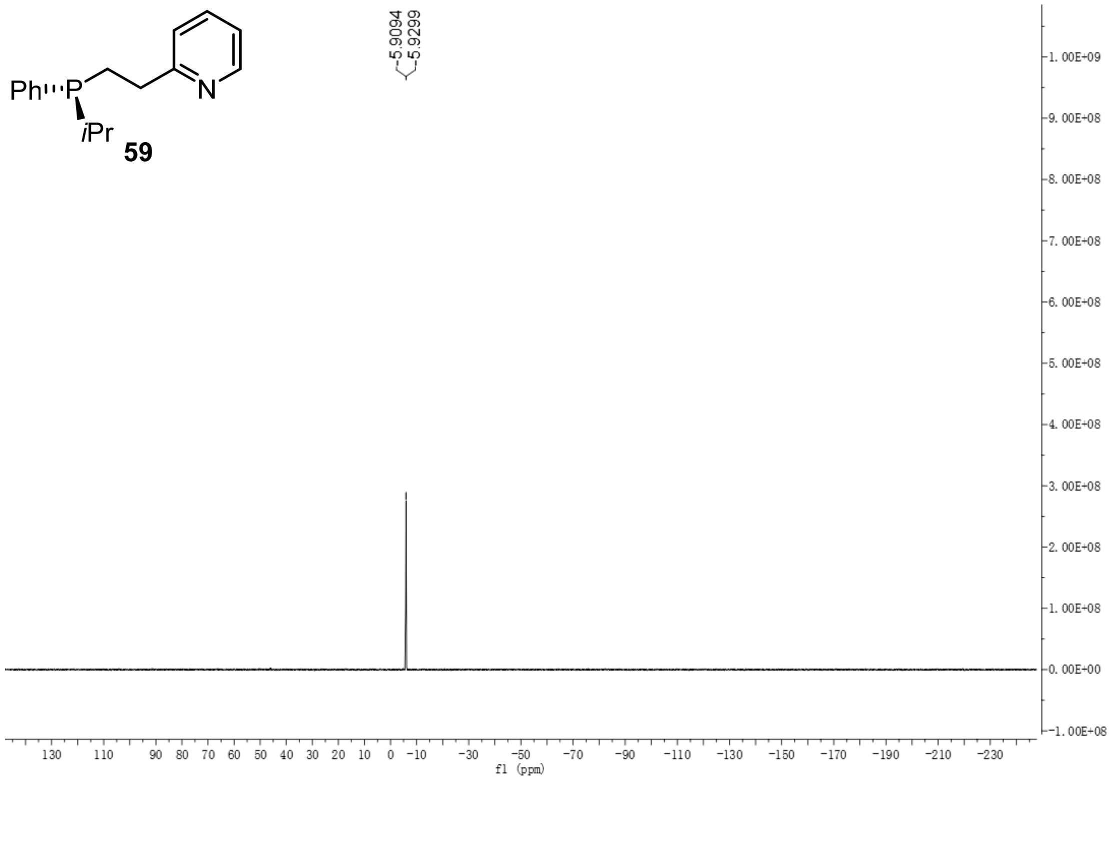

10. References

(1) B. Wang, Y. Liu, C. Jiang, Z. Cao, S. Cao, X. Zhao, X. Ban, Y. Yin, Z. Jiang, *Angew. Chem. Int. Ed.* **2023**, *62*, e202216605.

(2) Y. Liu, L. Zhang, Y. Zhang, S. Cao, X. Ban, Y. Yin, X. Zhao, Z. Jiang, *J. Am. Chem. Soc.* **2023**, *145*, 18307−18315.

(3) X. Bai, G. Zeng, T. Shao, Z. Jiang, *Angew. Chem., Int. Ed.* **2017**, *56*, 3684−3688.

(4) H. Luo, Y. Yang, Y. Fu, *Nat Commun*, **2024**, *15*, 5647-5658.

(5) M. J. Frisch; G. W. Trucks; H. B. Schlegel; G. E. Scuseria; M. A. Robb; J. R. Cheeseman; G. Scalmani; V. Barone; B. Mennucci; G. A. Petersson; H. Nakatsuji; M. Caricato; X. Li; H. P. Hratchian; A. F. Izmaylov; J. Bloino; G. Zheng; J. L. Sonnenberg; M. Hada; M. Ehara; K. Toyota; R. Fukuda; J. Hasegawa; M. Ishida; T. Nakajima; Y. Honda; O. Kitao; H. Nakai; T. Vreven; J. A. Jr. Montgomery; J. E. Peralta; F. Ogliaro; M. Bearpark; J. J. Heyd; E. Brothers; K. N. Kudin; V. N. Staroverov; R. Kobayashi; J. Normand; K. Raghavachari; A. Rendell; J. C. Burant; S. S. Iyengar; J. Tomasi; M. Cossi; N. Rega; N. J. Millam; M. Klene; J. E. Knox; J. B. Cross; V. Bakken; C. Adamo; J. Jaramillo; R. Gomperts; R. E. Stratmann; O. Yazyev; A. J. Austin; R. Cammi; C. Pomelli; J. W. Ochterski; R. L. Martin; K. Morokuma; V. G. Zakrzewski; G. A. Voth; P. Salvador; J. J. Dannenberg; S. Dapprich; A. D. Daniels; Ö. Farkas; J. B. Foresman; J. V. Ortiz; J. Cioslowski; D. J. Fox, Gaussian 16, Revision B.01; Gaussian, Inc.: Wallingford CT, **2016**.

(6) a) A. D. Becke, *Phys. Rev. A* **1988**, *38*, 3098-3100; A. D. Becke; b) *J. Chem. Phys.* **1993**, *98*, 5648-5652; c) C. Lee, W. Yang, R. G. Parr, *Phys. Rev. B* **1988**, *37*, 785-789.

(7) S. Grimme; J. Antony; S. Ehrlich; H. Krieg, *J. Chem. Phys*. **2010**, *132*, 154104.

(8) W. J. Hehre, R. Ditchfield, J. A. Pople, *J. Chem. Phys.* **1972**, *56*, 2257–2261.

(9) M. Dolg, U. Wedig, H. Stoll, H. *J.* Preuss, *Chem. Phys*. **1987**. *86*, 866.

(10) a) K. A. Fukui, *J. Phys. Chem.* **1970**, *74*, 4161-4163; b) K. Fukui, *Acc. Chem. Res.* **1981**, *14*, 363-368.

(11) A. V. Marenich, C. J. Cramer, D. G. Truhlar, *J. Phys. Chem. B*. **2009**, *113*, 6378.

(12) J. Contreras-García, E. R. Johnson, S. Keinan, R. Chaudret, J.-P. Piquemal, D. N. Beratan, W. *J.* Yang, *Chem. Theory Comput.* **2011**, *7 (3)*, 625−632.

(13) C. Y. Legault, CYLview, 1.0b; Université de Sherbrooke: Canada, **2009**; http://www.cylview.org.
